# Gut Microbial Changes Following Fecal Microbiota Transplantation for D-Lactic Acidosis in Two Children

**DOI:** 10.1097/PG9.0000000000000319

**Published:** 2023-06-09

**Authors:** Jordan D. Busing, Farnaz Fouladi, Emily C. Bulik-Sullivan, Ian M. Carroll, Anthony A. Fodor, Kelly F. Thomsen, Ajay S. Gulati, Maribeth R. Nicholson

**Affiliations:** From the * Department of Pediatric Gastroenterology, Hepatology, and Nutrition, Vanderbilt University Medical Center, Nashville, TN; † Department of Bioinformatics and Genomics, University of North Carolina at Charlotte, Charlotte, NC; ‡ School of Medicine, University of North Carolina, Chapel Hill, NC; § Department of Nutrition, The University of North Carolina at Chapel Hill, Chapel Hill, NC; ∥ Department of Pediatric Gastroenterology, Hepatology, and Nutrition, University of North Carolina at Chapel Hill, Chapel Hill, NC.

**Keywords:** gut bacteria, 16S rRNA profiling, metagenomics, stool microbiome

## Abstract

D-lactic acidosis (D-LA) is an uncommon complication of short bowel syndrome characterized by elevated plasma D-lactate and encephalopathy. Treatments include rehydration, dietary carbohydrate restriction, and antibiotics to alter the gut microbiota. Fecal microbiota transplantation (FMT) has recently been used in children to successfully treat D-LA. We compared the clinical course and then utilized metagenomic shotgun sequencing to describe changes in the composition and function of the intestinal microbiome following FMT in 2 patients with recurrent D-LA. FMT altered the composition of the fecal microbiota in these 2 patients with recurrent D-LA, though not necessarily in a consistent manner. Importantly, microbial metabolic pathways were also impacted by FMT, which may be critical for achieving desired clinical outcomes. While sample size limits the generalizability of our results, these findings set the stage for further understanding of the role of microbes in the pathogenesis of recurrent D-LA.

**What Is Known**

-  D-lactic acidosis is an uncommon complication of short bowel syndrome, characterized by elevated plasma D-lactate and encephalopathy.
-  Fecal microbiota transplantation (FMT) has previously been used in children as an efficacious treatment for D-lactic acidosis refractory to conventional therapies, though the mechanism remains unclear.

**What Is New**

- FMT was safe and effective for recurrent D-lactic acidosis in both patients described in this study.
- Despite clinical improvement, there was no significant increase in microbial diversity in two patients treated with FMT.
- Lack of improvement in microbial diversity but successful clinical response in both suggests that FMT efficacy for D-LA occurs via an alternate mechanism.
- Alterations in specific intestinal bacteria, including members of the genera Klebsiella and Veillonella, may provide important mechanistic clues about the pathogenesis and treatment of D-lactic acidosis.
- Translational Impact
- FMT was a safe and effective treatment in these two patients with refractory D-LA, though the mechanism behind their improvement remains unclear.

## INTRODUCTION

Short bowel syndrome (SBS) is a malabsorptive state characterized by innate or acquired shortening of the small intestine (1). D-lactic acidosis (D-LA) is a rare but severe condition that can occur in patients with SBS. In these children, colonic bacteria are exposed to excessive amounts of undigested carbohydrates, resulting in increased production of D-lactate via bacterial fermentation (2). Excessive absorption of D-lactate can then lead to metabolic acidosis and clinical manifestations such as somnolence, weakness, ataxia, slurred speech, irritability, and clumsiness (3). Conventional treatments including aggressive rehydration, dietary carbohydrate restriction, and antibiotics have variable response rates, highlighting the need for novel therapies (4).

Fecal microbiota transplantation (FMT) has been proposed as a treatment modality for D-LA recalcitrant to standard therapies; however, to date, only 2 patients successfully treated with FMT for D-LA have been reported, and the mechanism remains unclear (5,6). In this study, we report a third patient treated with FMT for recurrent D-LA. Importantly, for the first time, we perform shotgun metagenomic sequencing to evaluate the composition and function of the intestinal microbiome before and after FMT for D-LA. These analyses were performed for our newly identified patient and on samples formerly obtained from the child with recurrent D-LA previously published in the study by Bulik-Sullivan et al (6). This has allowed the characterization of the variability of microbiome changes in multiple patients with differing clinical courses.

## METHODS

Investigational new drug applications were approved by the Food and Drug Administration, and institutional review boards at respective centers approved the study. Informed parental consent and patient assent for FMT were obtained. Stool samples were obtained pre-FMT from donor and recipient and approximately 1-week and 1-month post-FMT from the recipients. Samples were stored at −80°C until analysis. Microbial DNA was extracted via enzymatic and physical homogenization, followed by phenol/chloroform/isoamyl alcohol extraction, as previously described (7,8). Sequencing libraries were prepared using the Illumina Nextera DNA Flex library preparation kit, and whole genome metagenomic shotgun sequencing was performed using the Illumina NovaSeq 6000 platform (2 × 150 base pairs). Kneaddata was used to remove human genome reads (http://huttenhower.sph.harvard.edu/kneaddata). The Kraken2 classifier was used for microbial taxonomic assignment (9). To account for different sequencing depths across samples, microbial count reads were normalized as previously described (10). The HUMAnN2 pipeline was used to profile the presence and abundance of metabolic pathways (11).

## RESULTS

### Case Descriptions

The new patient identified for this study was diagnosed with recurrent D-LA at Vanderbilt University Medical Center (VUMC). She was a 12-year-old female with SBS secondary to midgut volvulus associated with a mesenteric lymphangioma occurring at age 4. She had approximately 25 cm of intact small bowel remaining, with a retained ileocecal valve and colon. She had a normal diet without need for parental nutrition. In the 5 years before FMT, she had 3 laboratory-confirmed DLA episodes and additional episodes that were empirically treated based on symptoms. She had limited prior response to several oral antibiotics (metronidazole, gentamicin, sulfamethoxazole-trimethoprim, rifaximin, neomycin, and amoxicillin-clavulanic acid), commercially available *Lactobacillus rhamnosus* GG probiotic, blenderized diets, pancreatic enzyme replacement, and carbohydrate restriction.

The comparator patient was a 7-year-old female with SBS secondary to gastroschisis who was diagnosed at University of North Carolina (UNC) as previously described (6). Her residual small intestinal length was 40–50 cm, with the majority of the colon retained, but no ileocecal valve. Total parenteral nutrition provided approximately 50% of her caloric needs at presentation. She had 5 laboratory-confirmed episodes of D-LA with associated symptoms of ataxia and altered mental status over 4 months before FMT. She had limited response to oral antibiotics (metronidazole, gentamicin, sulfamethoxazole-trimethoprim, rifaximin, neomycin, and fluconazole) and carbohydrate restriction.

For FMT, both patients were admitted to their respective pediatric gastroenterology services, proton-pump inhibitor therapy was initiated, and bowel lavage was completed. Donor FMT material was obtained from OpenBiome (FMT Upper Delivery Microbiota Preparation, FMP30). Given the VUMC patient’s history of intermittent emesis, FMT was administered via nasoduodenal tube. FMT was administered via a nasogastric tube for the UNC patient. FMT was well tolerated with no reported adverse events. The UNC patient had no further recurrences of D-LA after FMT in the 1-year follow-up. The VUMC patient had a recurrence of D-LA 27 days post-FMT with altered mental status and an elevated D-lactate (1.75 mmol/L). The 1-month follow-up stool was collected while the patient was symptomatic with D-LA. Following sample collection, she was treated with a 2-week course of metronidazole and had no further episodes of D-LA in the 4 years following FMT.

### Microbiome Alterations Following FMT

**FIGURE 1.**
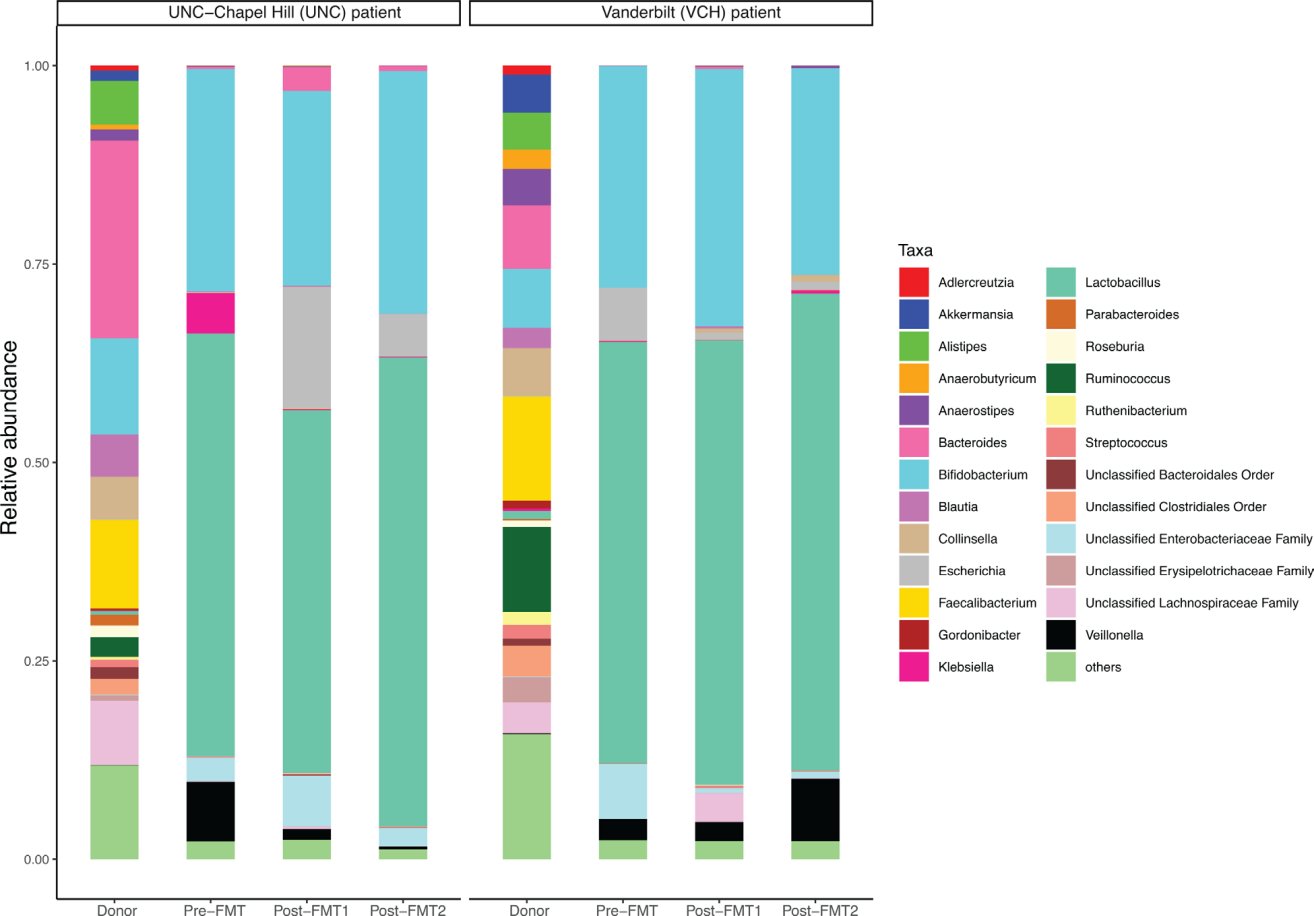
Microbial taxonomic composition of the VUMC and UNC patients’ and donors’ stool samples at the genus level. UNC, University of North Carolina; VUMC = Vanderbilt University Medical Center.

In both cases, the FMT donor material exhibited a higher microbial taxonomic diversity (Shannon diversity index, Supplemental Digital Content Fig. l, http://links.lww.com/PG9/A109) than the recipients’ pre-FMT stool samples. Neither patient had substantial increases in diversity post-FMT. *Lactobacillus* and *Bifidobacterium* genera were dominant in both patients’ stool samples before and after FMT (Fig. 1). In the UNC patient, the relative abundance of *Klebsiella* and *Veillonella* decreased from pre-FMT to post-FMT, and *Escherichia* increased. Alternatively, in the VUMC patient, the relative abundance of *Klebsiella*, which was low before FMT and at 1-week follow-up, increased at the time of D-LA recurrence (1-month post-FMT). In addition, *Veillonella* abundance increased at the time of D-LA recurrence, whereas *Escherichia* decreased post-FMT and remained low at the time of D-LA recurrence. Otherwise, the 2 patients had broadly similar taxonomies in pre- and posttransplant samples (Fig. 1).

**FIGURE 2.**
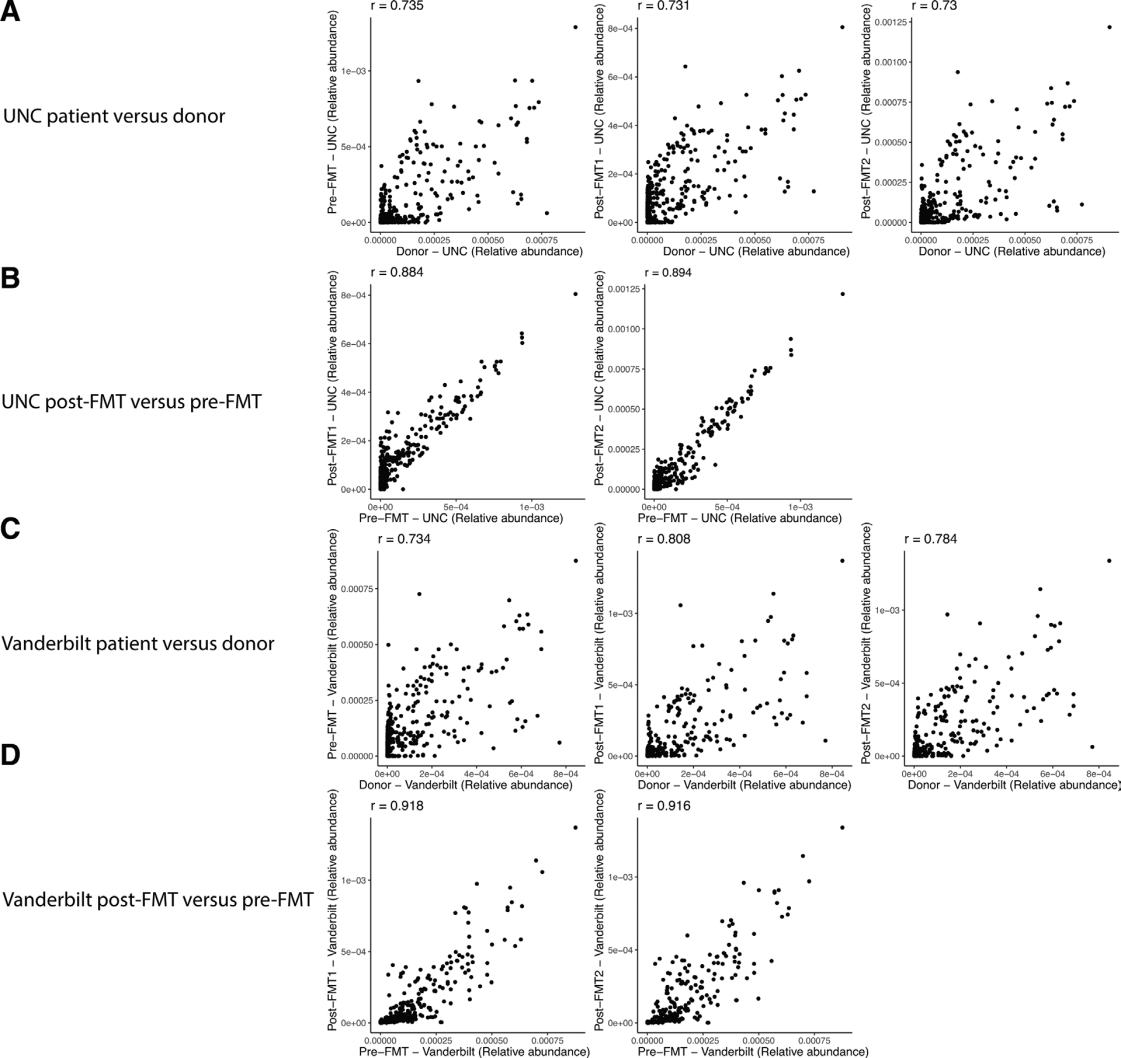
Scatter plots showing the relative abundances of metabolic pathways in patients versus donors and pre-FMT versus post-FMT samples from the UNC and VUMC patients. The Spearman’s rank correlation coefficient is shown for each scatter plot. FMT = fecal microbiota transplantation; VUMC = Vanderbilt University Medical Center.

Functional analysis via HUMAnN2 pipeline revealed that metabolic pathways from FMT recipients pre- and posttransplants were more similar for the same patient when compared to patients versus donors (Fig. 2). The metabolic pathways within a given patient’s microbiome were relatively conserved post-FMT, with a consistent signal in functional gene assignments. Comparing recipients to donors, there were numerous functional assignments similar between the VUMC and UNC samples (see Supplemental Digital Content Fig. 2, http://links.lww.com/PG9/A110). This suggests that the functional profile of SBS patients was similar in the 2 different patients and was stable after the transplant. Metabolic pathways higher in patients relative to donors include genes associated with *Veillonella* and *Bifidobacterium*, and many involve nucleotide biosynthesis pathways (see Supplemental Digital Content Table 1, http://links.lww.com/PG9/A111).

## DISCUSSION

D-LA is associated with significant morbidity and lacks effective therapeutics. FMT may be efficacious and safe in children suffering from recalcitrant D-LA. In this study, we report the temporal microbial composition, metagenomics, and variable clinical course of 2 patients with recurrent D-LA treated with FMT.

Despite ultimately successful outcomes, there was no significant increase in microbial diversity in the 2 patients after FMT. Diversity measures are commonly used as a surrogate of intestinal microbial health and frequently noted to improve in patients treated with FMT for *Clostridioides difficile* infection (12). The lack of improvement in microbial diversity but successful clinical response in our patients suggests that FMT efficacy for D-LA occurs via an alternate mechanism. This does not rely on large numbers of species colonizing and repopulating the SBS gut; in contrast, subtle changes in rare species and metabolites may be more relevant (6).

In both patients, *Lactobacillu*s and *Bifidobacterium* were the predominant bacteria pre- and post-FMT. Prior studies of the intestinal microbiota of patients with SBS have demonstrated variable findings. In a single study of adult patients with SBS, those with a retained ileocecal valve had predominantly *Lactobacillus* and *Prevotella* genera versus predominance of *Proteus*, *Klebsiella*, *Streptococcus*, and *Megasphaera* in those without an ileocecal valve. Our patients had no major differences in predominant genera despite the presence of an ileocecal value in one, and absence in the other (13).

Bulik-Sullivan et al (6) theorized that the reduction of the *Veillonella* genus (a known lactate-fermenting bacteria group) following FMT could play a role in the complex interaction of lactate producers and lactate-fermenting bacteria in the colon leading to the resolution of D-LA. Notably, our VUMC patient displayed an initial decrease in *Veillonella* abundance following FMT, which then bloomed at the time of symptomatic D-LA. *Veillonella* is a unique intestinal bacteria in that it uses lactate or lactic acid as its sole carbon fuel source (14). Hence, it is possible the increased abundance of *Veillonella* is related to increased lactate in the stool as an energy source. In our functional analysis (see Supplemental Digital Content Table 1, http://links.lww.com/PG9/A111), many *Veillonella* genes associated with nucleotide synthesis were consistently higher in the SBS patients. Future studies with larger sample sizes will be needed to determine the mechanistic role of *Veillonella* in the pathogenesis and treatment of recurrent D-LA.

Members of the *Klebsiella* genus also decreased in both patients post-FMT and increased at the time of D-LA recurrence in the VUMC patient. The *Klebsiella* genus includes bacterial species previously associated with both small intestinal bacterial overgrowth and inflammatory bowel disease (15). Its role in the development of D-LA also warrants additional study. Ultimately, larger cohorts of patients will be essential to determine microbial trends in the use of FMT for the treatment of D-LA in patients with SBS.

In conclusion, we present the intestinal microbiomes and metabolic pathways of 2 pediatric patients treated with FMT for D-LA. Alterations in specific intestinal bacteria, including members of the genera *Klebsiella* and *Veillonella*, may provide important mechanistic clues toward developing FMT as a therapeutic option for these complex patients.

## ACKNOWLEDGEMENT

The study participants were consented through IRB-approved studies.

## Supplementary Material
