# Supplementary material for: Gut Microbial Changes Following Fecal Microbiota Transplantation for D-Lactic Acidosis in Two Children
Source: JPGN Rep. 2023 Jun 9;4(3):e319. doi: 10.1097/PG9.0000000000000319 (PMC10435018; doi:10.1097/PG9.0000000000000319)
Supplement: Supplementary file 1 [file pg9-4-e319-s001.pdf]

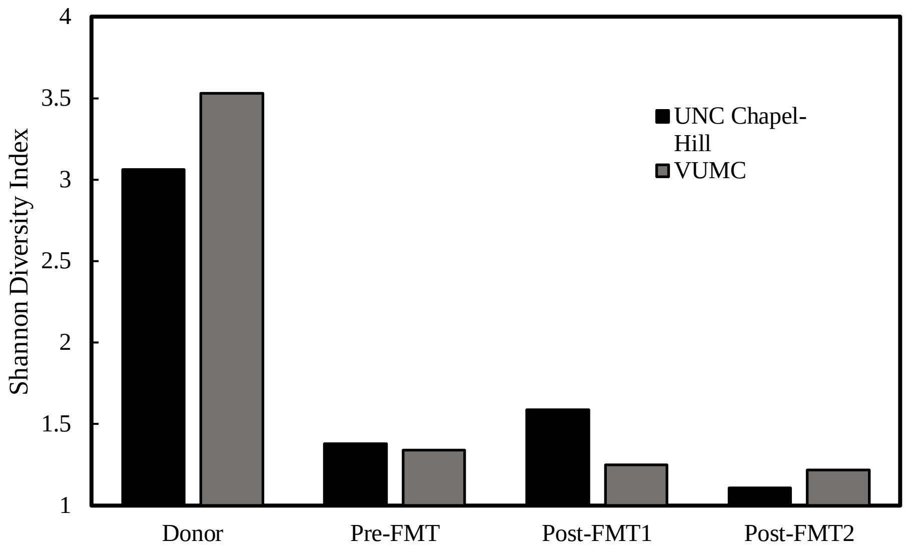

Supplemental Figure 1. Microbial diversity is lower in the D-LA patients' stool microbiota before and after FMT compared to the donors.
