# Supplementary material for: Gut Microbial Changes Following Fecal Microbiota Transplantation for D-Lactic Acidosis in Two Children
Source: JPGN Rep. 2023 Jun 9;4(3):e319. doi: 10.1097/PG9.0000000000000319 (PMC10435018; doi:10.1097/PG9.0000000000000319)
Supplement: Supplementary file 2 [file pg9-4-e319-s002.pdf]

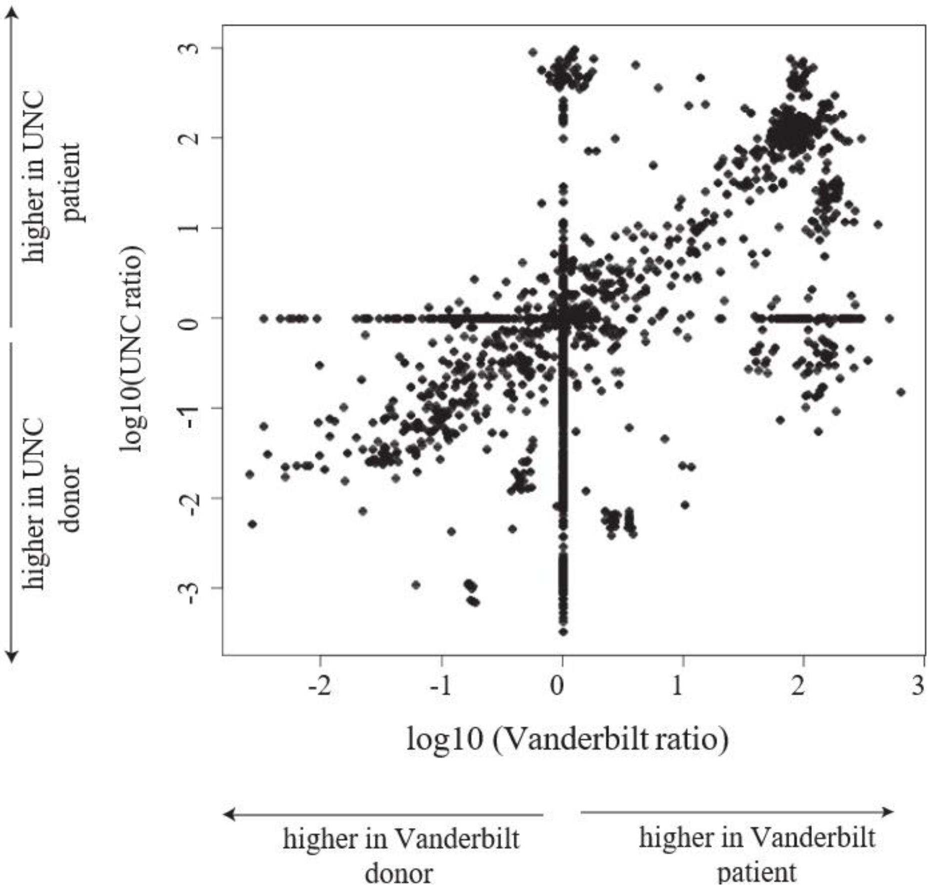

Supplemental Figure 2. For each gene from HumanN (see supplementary Table 1), the log10 of the ratio of the average of all timepoints for each patient divided by the corresponding value for the donor. The x-axis show this ratio for the Vanderbilt patient and the y-axis show this ratio for the Chapel Hill patient.
