# Supplementary material for: Gut Microbial Changes Following Fecal Microbiota Transplantation for D-Lactic Acidosis in Two Children
Source: JPGN Rep. 2023 Jun 9;4(3):e319. doi: 10.1097/PG9.0000000000000319 (PMC10435018; doi:10.1097/PG9.0000000000000319)
Supplement: Supplementary file 3 [file pg9-4-e319-s003.pdf]

Pathway
PWY_6703__preQ0_biosynthesis_g__Veillonella_s__Veillonella_atypica
PWY_6700__queuosine_biosynthesis_g__Veillonella_s__Veillonella_atypica
PWY_5791__1,4_dihydroxy_2_naphthoate_biosynthesis_II_(plants)_g__Veillonella_s__Veillonella_
PWY_5837__1,4_dihydroxy_2_naphthoate_biosynthesis_I_g__Veillonella_s__Veillonella_atypica
PWY_5686__UMP_biosynthesis_g__Veillonella_s__Veillonella_atypica
PWY_4242__pantothenate_and_coenzyme_A_biosynthesis_III_g__Veillonella_s__Veillonella_atyp
PWY_5100__pyruvate_fermentation_to_acetate_and_lactate_II_g__Veillonella_s__Veillonella_aty
PWY_7208__superpathway_of_pyrimidine_nucleobases_salvage_g__Veillonella_s__Veillonella_at
PWY_5686__UMP_biosynthesis_g__Bifidobacterium_s__Bifidobacterium_breve
PWY_5188__tetrapyrrole_biosynthesis_I_(from_glutamate)_g__Veillonella_s__Veillonella_atypica
PWY_6125__superpathway_of_guanosine_nucleotides_de_novo_biosynthesis_II_g__Veillonella_s_
HEMESYN2_PWY__heme_biosynthesis_II_(anaerobic)_g__Veillonella_s__Veillonella_atypica
PWY_5097__L_lysine_biosynthesis_VI_g__Veillonella_s__Veillonella_atypica
PWY_7221__guanosine_ribonucleotides_de_novo_biosynthesis_g__Veillonella_s__Veillonella_aty
GLUTORN_PWY__L_ornithine_biosynthesis_g__Bifidobacterium_s__Bifidobacterium_breve
UNINTEGRATED_g__Veillonella_s__Veillonella_atypica
PWY_6121__5_aminoimidazole_ribonucleotide_biosynthesis_I_g__Veillonella_s__Veillonella_atyp
PWY_7219__adenosine_ribonucleotides_de_novo_biosynthesis_g__Bifidobacterium_s__Bifidobac
ARGSYNBSUB_PWY__L_arginine_biosynthesis_II_(acetyl_cycle)_g__Bifidobacterium_s__Bifidobact
PWY_7111__pyruvate_fermentation_to_isobutanol_(engineered)_g__Bifidobacterium_s__Bifidoba
PWY_7228__superpathway_of_guanosine_nucleotides_de_novo_biosynthesis_I_g__Veillonella_s_
ARGSYN_PWY__L_arginine_biosynthesis_I_(via_L_ornithine)_g__Bifidobacterium_s__Bifidobacteri
PWY_7400__L_arginine_biosynthesis_IV_(archaebacteria)_g__Bifidobacterium_s__Bifidobacterium
PWY_7219__adenosine_ribonucleotides_de_novo_biosynthesis_g__Veillonella_s__Veillonella_aty
PWY_6387__UDP_N_acetylmuramoyl_pentapeptide_biosynthesis_I_(meso_diaminopimelate_con
PWY_6126__superpathway_of_adenosine_nucleotides_de_novo_biosynthesis_II_g__Veillonella_s_
PWY_6122__5_aminoimidazole_ribonucleotide_biosynthesis_II_g__Bifidobacterium_s__Bifidobact
PWY_6277__superpathway_of_5_aminoimidazole_ribonucleotide_biosynthesis_g__Bifidobacteriu
PWY_2942__L_lysine_biosynthesis_III_g__Veillonella_s__Veillonella_atypica
PEPTIDOGLYCANSYN_PWY__peptidoglycan_biosynthesis_I_(meso_diaminopimelate_containing)_g
PWY_7229__superpathway_of_adenosine_nucleotides_de_novo_biosynthesis_I_g__Veillonella_s_
PWY_7111__pyruvate_fermentation_to_isobutanol_(engineered)_g__Veillonella_s__Veillonella_a
VALSYN_PWY__L_valine_biosynthesis_g__Veillonella_s__Veillonella_atypica
PWY_6386__UDP_N_acetylmuramoyl_pentapeptide_biosynthesis_II_(lysine_containing)_g__Veillo
COA_PWY_1__coenzyme_A_biosynthesis_II_(mammalian)_g__Veillonella_s__Veillonella_atypica
UDPNAGSYN_PWY__UDP_N_acetyl_D_glucosamine_biosynthesis_I_g__Bifidobacterium_s__Bifido
HISTSYN_PWY__L_histidine_biosynthesis_g__Veillonella_s__Veillonella_atypica
PWY_6122__5_aminoimidazole_ribonucleotide_biosynthesis_II_g__Veillonella_s__Veillonella_aty
PWY_6277__superpathway_of_5_aminoimidazole_ribonucleotide_biosynthesis_g__Veillonella_s_
PWY_7220__adenosine_deoxyribonucleotides_de_novo_biosynthesis_II_g__Veillonella_s__Veillon
PWY_7222__guanosine_deoxyribonucleotides_de_novo_biosynthesis_II_g__Veillonella_s__Veillon
PWY_6936__seleno_amino_acid_biosynthesis_g__Bifidobacterium_s__Bifidobacterium_breve
PWY_5913__TCA_cycle_VI_(obligate_autotrophs)_g__Escherichia_s__Escherichia_coli
RIBOSYN2_PWY__flavin_biosynthesis_I_(bacteria_and_plants)_g__Escherichia_s__Escherichia_coli
PWY_6123__inosine_5__phosphate_biosynthesis_I_g__Bifidobacterium_s__Bifidobacterium_brev
PWY_6151__S_adenosyl_L_methionine_cycle_I_g__Bifidobacterium_s__Bifidobacterium_breve

PWY_6703__preQ0_biosynthesis_g__Escherichia_s__Escherichia_coli
PWY_7221__guanosine_ribonucleotides_de_novo_biosynthesis_g__Bifidobacterium_s__Bifidobac
PANTO_PWY__phosphopantothenate_biosynthesis_I_g__Escherichia_s__Escherichia_coli
PWY_7254__TCA_cycle_VII_(acetate_producers)_g__Escherichia_s__Escherichia_coli
COA_PWY__coenzyme_A_biosynthesis_I_g__Veillonella_s__Veillonella_atypica
PWY_6737__starch_degradation_V_g__Escherichia_s__Escherichia_coli
PWY_1269__CMP_3_deoxy_D_manno_octulosonate_biosynthesis_I_g__Escherichia_s__Escherichi
PWY_5103__L_isoleucine_biosynthesis_III_g__Bifidobacterium_s__Bifidobacterium_breve
PWY_2723__trehalose_degradation_V
PYRIDNUCSAL_PWY__NAD_salvage_pathway_I_g__Escherichia_s__Escherichia_coli
PWY_5189__tetrapyrrole_biosynthesis_II_(from_glycine)_g__Escherichia_s__Escherichia_coli
ILEUSYN_PWY__L_isoleucine_biosynthesis_I_(from_threonine)_g__Bifidobacterium_s__Bifidobact
VALSYN_PWY__L_valine_biosynthesis_g__Bifidobacterium_s__Bifidobacterium_breve
PWY_6353__purine_nucleotides_degradation_II_(aerobic)_g__Escherichia_s__Escherichia_coli
HEMESYN2_PWY__heme_biosynthesis_II_(anaerobic)_g__Escherichia_s__Escherichia_coli
GLUCUROCAT_PWY__superpathway_of_&beta__D_glucuronide_and_D_glucuronate_degradation_
SULFATE_CYS_PWY__superpathway_of_sulfate_assimilation_and_cysteine_biosynthesis_g__Esche
PYRIDNUCSYN_PWY__NAD_biosynthesis_I_(from_aspartate)_g__Escherichia_s__Escherichia_coli
BRANCHED_CHAIN_AA_SYN_PWY__superpathway_of_branched_amino_acid_biosynthesis_g__Bif
FASYN_INITIAL_PWY__superpathway_of_fatty_acid_biosynthesis_initiation_(E__coli)_g__Escherich
PWY_5861__superpathway_of_demethylmenaquinol_8_biosynthesis_g__Escherichia_s__Escherich
HCAMHPDEG_PWY__3_phenylpropanoate_and_3_(3_hydroxyphenyl)propanoate_degradation_to_
PWY_6690__cinnamate_and_3_hydroxycinnamate_degradation_to_2_oxopent_4_enoate
PWY_5863__superpathway_of_phylloquinol_biosynthesis_g__Escherichia_s__Escherichia_coli
AEROBACTINSYN_PWY__aerobactin_biosynthesis
AEROBACTINSYN_PWY__aerobactin_biosynthesis_g__Escherichia_s__Escherichia_coli
GLCMANNANAUT_PWY__superpathway_of_N_acetylglucosamine,_N_acetylmannosamine_and_N
UBISYN_PWY__superpathway_of_ubiquinol_8_biosynthesis_(prokaryotic)_g__Escherichia_s__Esch
PANTOSYN_PWY__pantothenate_and_coenzyme_A_biosynthesis_I_g__Escherichia_s__Escherichia
PWY_7269__NAD_NADP_NADH_NADPH_mitochondrial_interconversion_(yeast)
PWY_6527__stachyose_degradation_g__Escherichia_s__Escherichia_coli
PWY_6629__superpathway_of_L_tryptophan_biosynthesis_g__Escherichia_s__Escherichia_coli
PWY_6700__queuosine_biosynthesis_g__Escherichia_s__Escherichia_coli
PWY_6507__4_deoxy_L_threo_hex_4_enopyranuronate_degradation_g__Escherichia_s__Escheric
SER_GLYSYN_PWY__superpathway_of_L_serine_and_glycine_biosynthesis_I_g__Escherichia_s__Es
PWY_7242__D_fructuronate_degradation_g__Escherichia_s__Escherichia_coli
UNINTEGRATED_g__Bifidobacterium_s__Bifidobacterium_breve
GLYCOLYSIS_TCA_GLYOX_BYPASS__superpathway_of_glycolysis,_pyruvate_dehydrogenase,_TCA,_
PWY_6282__palmitoleate_biosynthesis_I_(from_(5Z)_dodec_5_enoate)_g__Escherichia_s__Escher
PWY_5838__superpathway_of_menaquinol_8_biosynthesis_I_g__Escherichia_s__Escherichia_coli
PWY_5989__stearate_biosynthesis_II_(bacteria_and_plants)_g__Escherichia_s__Escherichia_coli
PWY_5173__superpathway_of_acetyl_CoA_biosynthesis_g__Escherichia_s__Escherichia_coli
PWY_7269__NAD_NADP_NADH_NADPH_mitochondrial_interconversion_(yeast)_g__Escherichia_s
PWY_6163__chorismate_biosynthesis_from_3_dehydroquinate_g__Escherichia_s__Escherichia_co
GALACT_GLUCUROCAT_PWY__superpathway_of_hexuronide_and_hexuronate_degradation_g__E
HCAMHPDEG_PWY__3_phenylpropanoate_and_3_(3_hydroxyphenyl)propanoate_degradation_to_
PWY_6690__cinnamate_and_3_hydroxycinnamate_degradation_to_2_oxopent_4_enoate_g__Esch

PWY_6386__UDP_N_acetylmuramoyl_pentapeptide_biosynthesis_II_(lysine_containing)_g__Esche
PWY_6317__galactose_degradation_I_(Leloir_pathway)_g__Escherichia_s__Escherichia_coli
PWY_6892__thiazole_biosynthesis_I_(E__coli)_g__Escherichia_s__Escherichia_coli
PWY0_1297__superpathway_of_purine_deoxyribonucleosides_degradation_g__Escherichia_s__Es
PWY_6519__8_amino_7_oxononanoate_biosynthesis_I_g__Escherichia_s__Escherichia_coli
FASYN_ELONG_PWY__fatty_acid_elongation____saturated_g__Escherichia_s__Escherichia_coli
PWY_5897__superpathway_of_menaquinol_11_biosynthesis_g__Escherichia_s__Escherichia_coli
PWY_5898__superpathway_of_menaquinol_12_biosynthesis_g__Escherichia_s__Escherichia_coli
PWY_5899__superpathway_of_menaquinol_13_biosynthesis_g__Escherichia_s__Escherichia_coli
ARO_PWY__chorismate_biosynthesis_I_g__Escherichia_s__Escherichia_coli
COMPLETE_ARO_PWY__superpathway_of_aromatic_amino_acid_biosynthesis_g__Escherichia_s__
BIOTIN_BIOSYNTHESIS_PWY__biotin_biosynthesis_I_g__Escherichia_s__Escherichia_coli
PWY_7409__phospholipid_remodeling_(phosphatidylethanolamine,_yeast)_g__Escherichia_s__Esc
ECASYN_PWY__enterobacterial_common_antigen_biosynthesis_g__Escherichia_s__Escherichia_co
GALACTUROCAT_PWY__D_galacturonate_degradation_I_g__Escherichia_s__Escherichia_coli
PWY_7664__oleate_biosynthesis_IV_(anaerobic)_g__Escherichia_s__Escherichia_coli
PWY0_1533__methylphosphonate_degradation_I
MET_SAM_PWY__superpathway_of_S_adenosyl_L_methionine_biosynthesis_g__Escherichia_s__E
METSYN_PWY__L_homoserine_and_L_methionine_biosynthesis_g__Escherichia_s__Escherichia_co
PWY_6823__molybdenum_cofactor_biosynthesis_g__Escherichia_s__Escherichia_coli
PWY_5347__superpathway_of_L_methionine_biosynthesis_(transsulfuration)_g__Escherichia_s__
PWY0_1533__methylphosphonate_degradation_I_g__Escherichia_s__Escherichia_coli
GLYCOLYSIS_E_D__superpathway_of_glycolysis_and_Entner_Doudoroff_g__Escherichia_s__Escher
PWY_6305__putrescine_biosynthesis_IV_g__Escherichia_s__Escherichia_coli
PWY_5791__1,4_dihydroxy_2_naphthoate_biosynthesis_II_(plants)_g__Escherichia_s__Escherichia
PWY_5837__1,4_dihydroxy_2_naphthoate_biosynthesis_I_g__Escherichia_s__Escherichia_coli
PWY_5840__superpathway_of_menaquinol_7_biosynthesis_g__Escherichia_s__Escherichia_coli
PWY_5845__superpathway_of_menaquinol_9_biosynthesis_g__Escherichia_s__Escherichia_coli
PWY_5850__superpathway_of_menaquinol_6_biosynthesis_I_g__Escherichia_s__Escherichia_coli
PWY_5896__superpathway_of_menaquinol_10_biosynthesis_g__Escherichia_s__Escherichia_coli
PEPTIDOGLYCANSYN_PWY__peptidoglycan_biosynthesis_I_(meso_diaminopimelate_containing)_g
PWY_6385__peptidoglycan_biosynthesis_III_(mycobacteria)_g__Escherichia_s__Escherichia_coli
ECASYN_PWY__enterobacterial_common_antigen_biosynthesis
PWY_1042__glycolysis_IV_(plant_cytosol)_g__Escherichia_s__Escherichia_coli
PWY_5860__superpathway_of_demethylmenaquinol_6_biosynthesis_I_g__Escherichia_s__Escheri
PWY_5862__superpathway_of_demethylmenaquinol_9_biosynthesis_g__Escherichia_s__Escherich
PWY_6387__UDP_N_acetylmuramoyl_pentapeptide_biosynthesis_I_(meso_diaminopimelate_cont
PWY_4041__&gamma__glutamyl_cycle_unclassified
ENTBACSYN_PWY__enterobactin_biosynthesis_g__Escherichia_s__Escherichia_coli
PWY_4702__phytate_degradation_I_g__Escherichia_s__Escherichia_coli
PWY_5083__NAD_NADH_phosphorylation_and_dephosphorylation_g__Escherichia_s__Escherichia
PWY_5686__UMP_biosynthesis_g__Escherichia_s__Escherichia_coli
PWY0_1061__superpathway_of_L_alanine_biosynthesis_g__Escherichia_s__Escherichia_coli
PWY_6823__molybdenum_cofactor_biosynthesis
PWY_621__sucrose_degradation_III_(sucrose_invertase)_g__Escherichia_s__Escherichia_coli
TRPSYN_PWY__L_tryptophan_biosynthesis_g__Escherichia_s__Escherichia_coli
ILEUSYN_PWY__L_isoleucine_biosynthesis_I_(from_threonine)_g__Escherichia_s__Escherichia_col

VALSYN_PWY__L_valine_biosynthesis_g__Escherichia_s__Escherichia_coli
PWY_6630__superpathway_of_L_tyrosine_biosynthesis_g__Escherichia_s__Escherichia_coli
PWY_7315__dTDP_N_acetylthomosamine_biosynthesis_g__Escherichia_s__Escherichia_coli
GLYCOGENSYNTH_PWY__glycogen_biosynthesis_I_(from_ADP_D_Glucose)_g__Escherichia_s__Esc
GLYCOLYSIS__glycolysis_I_(from_glucose_6_phosphate)_g__Escherichia_s__Escherichia_coli
PWY_5188__tetrapyrrole_biosynthesis_I_(from_glutamate)_g__Escherichia_s__Escherichia_coli
PWY0_1586__peptidoglycan_maturation_(meso_diaminopimelate_containing)_g__Escherichia_s__
NAD_BIOSYNTHESIS_II__NAD_salvage_pathway_II_g__Escherichia_s__Escherichia_coli
PWY_5695__urate_biosynthesis_inosine_5__phosphate_degradation_g__Escherichia_s__Escherich
PWY0_1277__3_phenylpropanoate_and_3_(3_hydroxyphenyl)propanoate_degradation_g__Escher
PWY_6628__superpathway_of_L_phenylalanine_biosynthesis_g__Escherichia_s__Escherichia_coli
P461_PWY__hexitol_fermentation_to_lactate,_formate,_ethanol_and_acetate_g__Escherichia_s__
THRESYN_PWY__superpathway_of_L_threonine_biosynthesis_g__Escherichia_s__Escherichia_coli
PHOSLIPSYN_PWY__superpathway_of_phospholipid_biosynthesis_I_(bacteria)_g__Escherichia_s__
PWY66_400__glycolysis_VI_(metazoan)_g__Escherichia_s__Escherichia_coli
PWY_6151__S_adenosyl_L_methionine_cycle_I_g__Escherichia_s__Escherichia_coli
PWY_4242__pantothenate_and_coenzyme_A_biosynthesis_III_g__Escherichia_s__Escherichia_coli
PWY_5855__ubiquinol_7_biosynthesis_(prokaryotic)_g__Escherichia_s__Escherichia_coli
PWY_5856__ubiquinol_9_biosynthesis_(prokaryotic)_g__Escherichia_s__Escherichia_coli
PWY_5857__ubiquinol_10_biosynthesis_(prokaryotic)_g__Escherichia_s__Escherichia_coli
PWY_6708__ubiquinol_8_biosynthesis_(prokaryotic)_g__Escherichia_s__Escherichia_coli
PWY_5484__glycolysis_II_(from_fructose_6_phosphate)_g__Escherichia_s__Escherichia_coli
HOMOSER_METSYN_PWY__L_methionine_biosynthesis_I_g__Escherichia_s__Escherichia_coli
SO4ASSIM_PWY__sulfate_reduction_I_(assimilatory)_g__Escherichia_s__Escherichia_coli
PWY_7219__adenosine_ribonucleotides_de_novo_biosynthesis_g__Escherichia_s__Escherichia_co
PWY_7111__pyruvate_fermentation_to_isobutanol_(engineered)_g__Escherichia_s__Escherichia_
ARGSYN_PWY__L_arginine_biosynthesis_I_(via_L_ornithine)_g__Escherichia_s__Escherichia_coli
PWY_7388__octanoyl_[acyl_carrier_protein]_biosynthesis_(mitochondria,_yeast)_g__Escherichia_
PWY0_862__(5Z)_dodec_5_enoate_biosynthesis_g__Escherichia_s__Escherichia_coli
PWY_6147__6_hydroxymethyl_dihydropterin_diphosphate_biosynthesis_I_g__Escherichia_s__Esc
PWY_7221__guanosine_ribonucleotides_de_novo_biosynthesis_g__Escherichia_s__Escherichia_co
AST_PWY__L_arginine_degradation_II_(AST_pathway)
KDO_NAGLIPASYN_PWY__superpathway_of_(Kdo)2_lipid_A_biosynthesis_g__Escherichia_s__Esch
PWY0_1296__purine_ribonucleosides_degradation_g__Escherichia_s__Escherichia_coli
PWY66_422__D_galactose_degradation_V_(Leloir_pathway)_g__Escherichia_s__Escherichia_coli
GLUTORN_PWY__L_ornithine_biosynthesis_g__Escherichia_s__Escherichia_coli
SALVADEHYPOX_PWY__adenosine_nucleotides_degradation_II_g__Escherichia_s__Escherichia_co
PWY_6608__guanosine_nucleotides_degradation_III_g__Escherichia_s__Escherichia_coli
PWY_7409__phospholipid_remodeling_(phosphatidylethanolamine,_yeast)
PWY_4041__&gamma__glutamyl_cycle_g__Escherichia_s__Escherichia_coli
GLYOXYLATE_BYPASS__glyoxylate_cycle_g__Escherichia_s__Escherichia_coli
PWY_7234__inosine_5__phosphate_biosynthesis_III_g__Escherichia_s__Escherichia_coli
FUCCAT_PWY__fucose_degradation_g__Escherichia_s__Escherichia_coli
PWY0_1261__anhydromuropeptides_recycling_g__Escherichia_s__Escherichia_coli
PWY_5138__unsaturated,_even_numbered_fatty_acid_&beta__oxidation_g__Escherichia_s__Esch
PWY_6124__inosine_5__phosphate_biosynthesis_II_g__Escherichia_s__Escherichia_coli
HISTSYN_PWY__L_histidine_biosynthesis_g__Escherichia_s__Escherichia_coli

PWY_5705__allantoin_degradation_to_glyoxylate_III_g__Escherichia_s__Escherichia_coli
PWY_6123__inosine_5__phosphate_biosynthesis_I_g__Escherichia_s__Escherichia_coli
PWY_7663__gondoate_biosynthesis_(anaerobic)_g__Escherichia_s__Escherichia_coli
PWY_2723__trehalose_degradation_V_g__Escherichia_s__Escherichia_coli
FUC_RHAMCAT_PWY__superpathway_of_fucose_and_rhamnose_degradation_g__Escherichia_s__
PWY_5136__fatty_acid_&beta__oxidation_II_(peroxisome)_g__Escherichia_s__Escherichia_coli
ANAGLYCOLYSIS_PWY__glycolysis_III_(from_glucose)_g__Escherichia_s__Escherichia_coli
TCA_GLYOX_BYPASS__superpathway_of_glyoxylate_bypass_and_TCA_g__Escherichia_s__Escheric
FAO_PWY__fatty_acid_&beta__oxidation_I_g__Escherichia_s__Escherichia_coli
GLYCOL_GLYOXDEG_PWY__superpathway_of_glycol_metabolism_and_degradation_g__Escherichi
PWY_6969__TCA_cycle_V_(2_oxoglutarate_ferredoxin_oxidoreductase)_g__Escherichia_s__Escher
P105_PWY__TCA_cycle_IV_(2_oxoglutarate_decarboxylase)_g__Escherichia_s__Escherichia_coli
PWY0_1241__ADP_L_glycero_&beta__D_manno_heptose_biosynthesis_g__Escherichia_s__Escher
UDPNAGSYN_PWY__UDP_N_acetyl_D_glucosamine_biosynthesis_I_g__Escherichia_s__Escherichia
AST_PWY__L_arginine_degradation_II_(AST_pathway)_g__Escherichia_s__Escherichia_coli
POLYAMSYN_PWY__superpathway_of_polyamine_biosynthesis_I_g__Escherichia_s__Escherichia_
PWY_6609__adenine_and_adenosine_salvage_III_g__Escherichia_s__Escherichia_coli
PWY0_1338__polymyxin_resistance
POLYISOPRENSYN_PWY__polyisoprenoid_biosynthesis_(E__coli)_g__Escherichia_s__Escherichia_co
PWY0_1338__polymyxin_resistance_g__Escherichia_s__Escherichia_coli
TCA__TCA_cycle_I_(prokaryotic)_g__Escherichia_s__Escherichia_coli
PWY_6936__seleno_amino_acid_biosynthesis_g__Escherichia_s__Escherichia_coli
PWY_6612__superpathway_of_tetrahydrofolate_biosynthesis_g__Escherichia_s__Escherichia_coli
PWY_7315__dTDP_N_acetylthomosamine_biosynthesis
PWY_6859__all_trans_farnesol_biosynthesis_g__Escherichia_s__Escherichia_coli
PWY_6897__thiamin_salvage_II_g__Escherichia_s__Escherichia_coli
ORNDEG_PWY__superpathway_of_ornithine_degradation_g__Escherichia_s__Escherichia_coli
PWY_5659__GDP_mannose_biosynthesis_g__Escherichia_s__Escherichia_coli
ARGDEG_PWY__superpathway_of_L_arginine,_putrescine,_and_4_aminobutanoate_degradation_
ORNARGDEG_PWY__superpathway_of_L_arginine_and_L_ornithine_degradation_g__Escherichia_
PWY4FS_7__phosphatidylglycerol_biosynthesis_I_(plastidic)_g__Escherichia_s__Escherichia_coli
PWY4FS_8__phosphatidylglycerol_biosynthesis_II_(non_plastidic)_g__Escherichia_s__Escherichia_
PWY_561__superpathway_of_glyoxylate_cycle_and_fatty_acid_degradation_g__Escherichia_s__Es
GALACTARDEG_PWY__D_galactarate_degradation_I_g__Escherichia_s__Escherichia_coli
GLUCARGALACTSUPER_PWY__superpathway_of_D_glucarate_and_D_galactarate_degradation_g_
PWY_6731__starch_degradation_III
THRESYN_PWY__superpathway_of_L_threonine_biosynthesis_g__Lactobacillus_s__Lactobacillus_v
PWY_5690__TCA_cycle_II_(plants_and_fungi)_g__Escherichia_s__Escherichia_coli
PWY0_1298__superpathway_of_pyrimidine_deoxyribonucleosides_degradation_g__Escherichia_s_
FOLSYN_PWY__superpathway_of_tetrahydrofolate_biosynthesis_and_salvage_g__Escherichia_s__
UNINTEGRATED_g__Lactobacillus_s__Lactobacillus_reuteri
KDO_NAGLIPASYN_PWY__superpathway_of_(Kdo)2_lipid_A_biosynthesis
PWY0_1277__3_phenylpropanoate_and_3_(3_hydroxyphenyl)propanoate_degradation
ASPASN_PWY__superpathway_of_L_aspartate_and_L_asparagine_biosynthesis_g__Escherichia_s_
PWY_3841__folate_transformations_II_g__Escherichia_s__Escherichia_coli
UNINTEGRATED_g__Escherichia_s__Escherichia_coli
PWY_7663__gondoate_biosynthesis_(anaerobic)_g__Lactobacillus_s__Lactobacillus_vaginalis

PWY_5667__CDP_diacylglycerol_biosynthesis_I_g__Escherichia_s__Escherichia_coli
PWY0_1319__CDP_diacylglycerol_biosynthesis_II_g__Escherichia_s__Escherichia_coli
COA_PWY_1__coenzyme_A_biosynthesis_II_(mammalian)_g__Escherichia_s__Escherichia_coli
COBALSYN_PWY__adenosylcobalamin_salvage_from_cobinamide_I_g__Escherichia_s__Escherichia
PWY_6803__phosphatidylcholine_acyl_editing_g__Escherichia_s__Escherichia_coli
PWY_6387__UDP_N_acetylmuramoyl_pentapeptide_biosynthesis_I_(meso_diaminopimelate_con
RHAMCAT_PWY__L_rhamnose_degradation_I_g__Escherichia_s__Escherichia_coli
PWY_5384__sucrose_degradation_IV_(sucrose_phosphorylase)_g__Escherichia_s__Escherichia_co
GLUCOSE1PMETAB_PWY__glucose_and_glucose_1_phosphate_degradation_g__Escherichia_s__Es
PWY_2941__L_lysine_biosynthesis_II_g__Lactobacillus_s__Lactobacillus_vaginalis
PEPTIDOGLYCANSYN_PWY__peptidoglycan_biosynthesis_I_(meso_diaminopimelate_containing)_g
PWY_5097__L_lysine_biosynthesis_VI_g__Lactobacillus_s__Lactobacillus_vaginalis
ORNDEG_PWY__superpathway_of_ornithine_degradation
PWY_7357__thiamin_formation_from_pyrithiamine_and_oxythiamine_(yeast)_g__Escherichia_s__
PWY_6891__thiazole_biosynthesis_II_(Bacillus)
COA_PWY__coenzyme_A_biosynthesis_I_g__Escherichia_s__Escherichia_coli
PWY_6386__UDP_N_acetylmuramoyl_pentapeptide_biosynthesis_II_(lysine_containing)_g__Lacto
1CMET2_PWY__N10_formyl_tetrahydrofolate_biosynthesis_g__Escherichia_s__Escherichia_coli
PWY_2942__L_lysine_biosynthesis_III_g__Lactobacillus_s__Lactobacillus_vaginalis
PWY_5973__cis_vaccenate_biosynthesis_g__Lactobacillus_s__Lactobacillus_vaginalis
PWY_724__superpathway_of_L_lysine,_L_threonine_and_L_methionine_biosynthesis_II_g__Lacto
PWY_6121__5_aminoimidazole_ribonucleotide_biosynthesis_I_g__Escherichia_s__Escherichia_col
PWY_6121__5_aminoimidazole_ribonucleotide_biosynthesis_I_g__Lactobacillus_s__Lactobacillus_
NAGLIPASYN_PWY__lipid_IVA_biosynthesis_g__Escherichia_s__Escherichia_coli
GLYCOL_GLYOXDEG_PWY__superpathway_of_glycol_metabolism_and_degradation
UNINTEGRATED_g__Lactobacillus_s__Lactobacillus_vaginalis
PWY_7357__thiamin_formation_from_pyrithiamine_and_oxythiamine_(yeast)_g__Lactobacillus_s
PWY_3841__folate_transformations_II_g__Lactobacillus_s__Lactobacillus_salivarius
METHGLYUT_PWY__superpathway_of_methylglyoxal_degradation_g__Escherichia_s__Escherichia
PWY_6123__inosine_5__phosphate_biosynthesis_I_g__Lactobacillus_s__Lactobacillus_salivarius
PWY_7663__gondoate_biosynthesis_(anaerobic)_g__Lactobacillus_s__Lactobacillus_salivarius
PWY_6122__5_aminoimidazole_ribonucleotide_biosynthesis_II_g__Lactobacillus_s__Lactobacillus_
PWY_6277__superpathway_of_5_aminoimidazole_ribonucleotide_biosynthesis_g__Lactobacillus_
PWY_7013__L_1,2_propanediol_degradation
PWY_5173__superpathway_of_acetyl_CoA_biosynthesis
PWY_6317__galactose_degradation_I_(Leloir_pathway)_g__Lactobacillus_s__Lactobacillus_salivar
PWY_5686__UMP_biosynthesis_g__Lactobacillus_s__Lactobacillus_vaginalis
PWY_7197__pyrimidine_deoxyribonucleotide_phosphorylation_g__Lactobacillus_s__Lactobacillus_
PWY_4041__&gamma__glutamyl_cycle
COA_PWY_1__coenzyme_A_biosynthesis_II_(mammalian)_g__Lactobacillus_s__Lactobacillus_vagi
PWY_5138__unsaturated,_even_numbered_fatty_acid_&beta__oxidation
PWY_922__mevalonate_pathway_I_g__Lactobacillus_s__Lactobacillus_vaginalis
PWY_7220__adenosine_deoxyribonucleotides_de_novo_biosynthesis_II_g__Lactobacillus_s__Lact
PWY_7222__guanosine_deoxyribonucleotides_de_novo_biosynthesis_II_g__Lactobacillus_s__Lact
PWY0_1241__ADP_L_glycero_&beta__D_manno_heptose_biosynthesis
PWY_6122__5_aminoimidazole_ribonucleotide_biosynthesis_II_g__Escherichia_s__Escherichia_co
PWY_6277__superpathway_of_5_aminoimidazole_ribonucleotide_biosynthesis_g__Escherichia_s_

PWY_7184__pyrimidine_deoxyribonucleotides_de_novo_biosynthesis_I_g__Lactobacillus_s__Lacto
PWY_5656__mannosylglycerate_biosynthesis_I
PWY_6545__pyrimidine_deoxyribonucleotides_de_novo_biosynthesis_III_g__Lactobacillus_s__Lac
KETOGLUCONMET_PWY__ketogluconate_metabolism
PWY_7210__pyrimidine_deoxyribonucleotides_biosynthesis_from_CTP_g__Lactobacillus_s__Lacto
COLANSYN_PWY__colanic_acid_building_blocks_biosynthesis_g__Escherichia_s__Escherichia_coli
PWY_7198__pyrimidine_deoxyribonucleotides_de_novo_biosynthesis_IV_g__Lactobacillus_s__Lac
PWY0_166__superpathway_of_pyrimidine_deoxyribonucleotides_de_novo_biosynthesis_(E__coli)
PWY66_422__D_galactose_degradation_V_(Leloir_pathway)_g__Lactobacillus_s__Lactobacillus_sa
PWY_7197__pyrimidine_deoxyribonucleotide_phosphorylation_g__Lactobacillus_s__Lactobacillus_
PWY_5083__NAD_NADH_phosphorylation_and_dephosphorylation
PWY_6125__superpathway_of_guanosine_nucleotides_de_novo_biosynthesis_II_g__Lactobacillus_
GLYCOGENSYNTH_PWY__glycogen_biosynthesis_I_(from_ADP_D_Glucose)_g__Lactobacillus_s__La
PWY_7228__superpathway_of_guanosine_nucleotides_de_novo_biosynthesis_I_g__Lactobacillus_
GLUCARDEG_PWY__D_glucarate_degradation_I_g__Escherichia_s__Escherichia_coli
PWY_5367__petroselinate_biosynthesis_g__Lactobacillus_s__Lactobacillus_salivarius
PWY_7221__guanosine_ribonucleotides_de_novo_biosynthesis_g__Lactobacillus_s__Lactobacillus
PWY_6859__all_trans_farnesol_biosynthesis
GLCMANNANAUT_PWY__superpathway_of_N_acetylglucosamine,_N_acetylmannosamine_and_N
ARGDEG_PWY__superpathway_of_L_arginine,_putrescine,_and_4_aminobutanoate_degradation
ORNARGDEG_PWY__superpathway_of_L_arginine_and_L_ornithine_degradation
UNINTEGRATED_g__Lactobacillus_s__Lactobacillus_johnsonii
PWY_5100__pyruvate_fermentation_to_acetate_and_lactate_II_g__Lactobacillus_s__Lactobacillus
PWY_3001__superpathway_of_L_isoleucine_biosynthesis_I_g__Escherichia_s__Escherichia_coli
PWY_2941__L_lysine_biosynthesis_II_g__Lactobacillus_s__Lactobacillus_salivarius
PWY_6629__superpathway_of_L_tryptophan_biosynthesis
GLUCOSE1PMETAB_PWY__glucose_and_glucose_1_phosphate_degradation
PWY_7219__adenosine_ribonucleotides_de_novo_biosynthesis_g__Lactobacillus_s__Lactobacillus
UNINTEGRATED_g__Lactobacillus_s__Lactobacillus_helveticus
TCA_GLYOX_BYPASS__superpathway_of_glyoxylate_bypass_and_TCA
METHGLYUT_PWY__superpathway_of_methylglyoxal_degradation
PWY_7219__adenosine_ribonucleotides_de_novo_biosynthesis_g__Lactobacillus_s__Lactobacillus
GLYOXYLATE_BYPASS__glyoxylate_cycle
PWY_5723__Rubisco_shunt
PWY_5860__superpathway_of_demethylmenaquinol_6_biosynthesis_I
PWY_5862__superpathway_of_demethylmenaquinol_9_biosynthesis
POLYISOPRENSYN_PWY__polyisoprenoid_biosynthesis_(E__coli)
BRANCHED_CHAIN_AA_SYN_PWY__superpathway_of_branched_amino_acid_biosynthesis_g__Esc
PWY_7254__TCA_cycle_VII_(acetate_producers)
UNINTEGRATED_g__Lactobacillus_s__Lactobacillus_salivarius
GLUCARDEG_PWY__D_glucarate_degradation_I
UBISYN_PWY__superpathway_of_ubiquinol_8_biosynthesis_(prokaryotic)
PWY_7279__aerobic_respiration_II_(cytochrome_c)_(yeast)
PWY_6121__5_aminoimidazole_ribonucleotide_biosynthesis_I_g__Lactobacillus_s__Lactobacillus_
PWY_6803__phosphatidylcholine_acyl_editing
PWY0_1415__superpathway_of_heme_biosynthesis_from_uroporphyrinogen_III
PWY_5705__allantoin_degradation_to_glyoxylate_III

PWY_5845__superpathway_of_menaquinol_9_biosynthesis
PWY_5850__superpathway_of_menaquinol_6_biosynthesis_I
PWY_5896__superpathway_of_menaquinol_10_biosynthesis
GALACTARDEG_PWY__D_galactarate_degradation_I
GLUCARGALACTSUPER_PWY__superpathway_of_D_glucarate_and_D_galactarate_degradation
PWY_5384__sucrose_degradation_IV_(sucrose_phosphorylase)
PWY_7228__superpathway_of_guanosine_nucleotides_de_novo_biosynthesis_I_g__Lactobacillus_
PWY_5973__cis_vaccenate_biosynthesis_g__Lactobacillus_s__Lactobacillus_salivarius
PWY_6151__S_adenosyl_L_methionine_cycle_I_g__Lactobacillus_s__Lactobacillus_johnsonii
PWY_5103__L_isoleucine_biosynthesis_III_g__Escherichia_s__Escherichia_coli
PWY_6122__5_aminoimidazole_ribonucleotide_biosynthesis_II_g__Lactobacillus_s__Lactobacillus_
PWY_6277__superpathway_of_5_aminoimidazole_ribonucleotide_biosynthesis_g__Lactobacillus_
PWY_922__mevalonate_pathway_I
PWY_7219__adenosine_ribonucleotides_de_novo_biosynthesis_g__Lactobacillus_s__Lactobacillus
COA_PWY_1__coenzyme_A_biosynthesis_II_(mammalian)_g__Lactobacillus_s__Lactobacillus_john
PWY_5861__superpathway_of_demethylmenaquinol_8_biosynthesis
PWY_3781__aerobic_respiration_I_(cytochrome_c)
PWY_5910__superpathway_of_geranylgeranyldiphosphate_biosynthesis_I_(via_mevalonate)
COA_PWY__coenzyme_A_biosynthesis_I_g__Lactobacillus_s__Lactobacillus_johnsonii
GLYCOLYSIS_TCA_GLYOX_BYPASS__superpathway_of_glycolysis,_pyruvate_dehydrogenase,_TCA,_
PWY_5855__ubiquinol_7_biosynthesis_(prokaryotic)
PWY_5856__ubiquinol_9_biosynthesis_(prokaryotic)
PWY_5857__ubiquinol_10_biosynthesis_(prokaryotic)
PWY_6708__ubiquinol_8_biosynthesis_(prokaryotic)
PWY_7221__guanosine_ribonucleotides_de_novo_biosynthesis_g__Lactobacillus_s__Lactobacillus
PWY_7208__superpathway_of_pyrimidine_nucleobases_salvage_g__Lactobacillus_s__Lactobacillu
PWY_6386__UDP_N_acetylmuramoyl_pentapeptide_biosynthesis_II_(lysine_containing)_g__Lacto
UDPNAGSYN_PWY__UDP_N_acetyl_D_glucosamine_biosynthesis_I_g__Lactobacillus_s__Lactobac
PWY_5188__tetrapyrrole_biosynthesis_I_(from_glutamate)_g__Lactobacillus_s__Lactobacillus_reu
PWY_7323__superpathway_of_GDP_mannose_derived_O_antigen_building_blocks_biosynthesis_
PWY_6387__UDP_N_acetylmuramoyl_pentapeptide_biosynthesis_I_(meso_diaminopimelate_cont
PWY_5384__sucrose_degradation_IV_(sucrose_phosphorylase)_unclassified
REDCITCYC__TCA_cycle_VIII_(helicobacter)
P105_PWY__TCA_cycle_IV_(2_oxoglutarate_decarboxylase)
PWY_5838__superpathway_of_menaquinol_8_biosynthesis_I
PEPTIDOGLYCANSYN_PWY__peptidoglycan_biosynthesis_I_(meso_diaminopimelate_containing)_g
PWY_7229__superpathway_of_adenosine_nucleotides_de_novo_biosynthesis_I_g__Lactobacillus_
PWY0_1296__purine_ribonucleosides_degradation_g__Lactobacillus_s__Lactobacillus_reuteri
PWY_5686__UMP_biosynthesis_g__Lactobacillus_s__Lactobacillus_reuteri
PWY_6147__6_hydroxymethyl_dihydropterin_diphosphate_biosynthesis_I_g__Lactobacillus_s__La
PWY_3841__folate_transformations_II_g__Lactobacillus_s__Lactobacillus_reuteri
PWY_6895__superpathway_of_thiamin_diphosphate_biosynthesis_II
PWY_6126__superpathway_of_adenosine_nucleotides_de_novo_biosynthesis_II_g__Lactobacillus_
PWY_7357__thiamin_formation_from_pyrithiamine_and_oxythiamine_(yeast)_g__Lactobacillus_s_
UNINTEGRATED_g__Lactobacillus_s__Lactobacillus_gasseri
PWY_7219__adenosine_ribonucleotides_de_novo_biosynthesis_g__Lactobacillus_s__Lactobacillus
PWY_6859__all_trans_farnesol_biosynthesis_g__Lactobacillus_s__Lactobacillus_reuteri

PWY_2942__L_lysine_biosynthesis_III_g__Lactobacillus_s__Lactobacillus_reuteri
PWY_7219__adenosine_ribonucleotides_de_novo_biosynthesis_g__Lactobacillus_s__Lactobacillus
PWY_6892__thiazole_biosynthesis_I_(E__coli)
COA_PWY_1__coenzyme_A_biosynthesis_II_(mammalian)_g__Lactobacillus_s__Lactobacillus_reut
PWY66_422__D_galactose_degradation_V_(Leloir_pathway)_g__Lactobacillus_s__Lactobacillus_re
PWY_4242__pantothenate_and_coenzyme_A_biosynthesis_III_g__Lactobacillus_s__Lactobacillus_
PWY_7221__guanosine_ribonucleotides_de_novo_biosynthesis_g__Lactobacillus_s__Lactobacillus
COA_PWY__coenzyme_A_biosynthesis_I_g__Lactobacillus_s__Lactobacillus_reuteri
PWY_5791__1,4_dihydroxy_2_naphthoate_biosynthesis_II_(plants)
PWY_5837__1,4_dihydroxy_2_naphthoate_biosynthesis_I
UDPNAGSYN_PWY__UDP_N_acetyl_D_glucosamine_biosynthesis_I_g__Lactobacillus_s__Lactobaci
PWY_841__superpathway_of_purine_nucleotides_de_novo_biosynthesis_I_g__Lactobacillus_s__L
UDPNAGSYN_PWY__UDP_N_acetyl_D_glucosamine_biosynthesis_I_g__Lactobacillus_s__Lactobaci
PWY_6387__UDP_N_acetylmuramoyl_pentapeptide_biosynthesis_I_(meso_diaminopimelate_con
PWY_7234__inosine_5__phosphate_biosynthesis_III_g__Lactobacillus_s__Lactobacillus_delbrueck
PWY_6386__UDP_N_acetylmuramoyl_pentapeptide_biosynthesis_II_(lysine_containing)_g__Lacto
PWY_4981__L_proline_biosynthesis_II_(from_arginine)_g__Lactobacillus_s__Lactobacillus_reuteri
PWY_2941__L_lysine_biosynthesis_II_g__Lactobacillus_s__Lactobacillus_helveticus
PEPTIDOGLYCANSYN_PWY__peptidoglycan_biosynthesis_I_(meso_diaminopimelate_containing)_g
PWY_6609__adenine_and_adenosine_salvage_III_g__Lactobacillus_s__Lactobacillus_reuteri
PWY_6124__inosine_5__phosphate_biosynthesis_II_g__Lactobacillus_s__Lactobacillus_reuteri
PWY_7234__inosine_5__phosphate_biosynthesis_III_g__Lactobacillus_s__Lactobacillus_reuteri
PWY_5100__pyruvate_fermentation_to_acetate_and_lactate_II_g__Lactobacillus_s__Lactobacillus
PWY_2941__L_lysine_biosynthesis_II_g__Lactobacillus_s__Lactobacillus_reuteri
PWY_6125__superpathway_of_guanosine_nucleotides_de_novo_biosynthesis_II_g__Lactobacillus_
PWY_5097__L_lysine_biosynthesis_VI_g__Lactobacillus_s__Lactobacillus_reuteri
PWY_6123__inosine_5__phosphate_biosynthesis_I_g__Lactobacillus_s__Lactobacillus_reuteri
PWY_7228__superpathway_of_guanosine_nucleotides_de_novo_biosynthesis_I_g__Lactobacillus_
PWY_7539__6_hydroxymethyl_dihydropterin_diphosphate_biosynthesis_III_(Chlamydia)_g__Lacto
PWY_7446__sulfoglycolysis
PWY_3841__folate_transformations_II_g__Lactobacillus_s__Lactobacillus_johnsonii
PWY_7220__adenosine_deoxyribonucleotides_de_novo_biosynthesis_II_g__Lactobacillus_s__Lact
PWY_7222__guanosine_deoxyribonucleotides_de_novo_biosynthesis_II_g__Lactobacillus_s__Lact
PWY_6121__5_aminoimidazole_ribonucleotide_biosynthesis_I_g__Lactobacillus_s__Lactobacillus_
PWY_6630__superpathway_of_L_tyrosine_biosynthesis
PWY_6317__galactose_degradation_I_(Leloir_pathway)_g__Lactobacillus_s__Lactobacillus_johnso
PWY66_422__D_galactose_degradation_V_(Leloir_pathway)_g__Lactobacillus_s__Lactobacillus_jo
PWY_5667__CDP_diacylglycerol_biosynthesis_I_g__Lactobacillus_s__Lactobacillus_reuteri
PWY0_1319__CDP_diacylglycerol_biosynthesis_II_g__Lactobacillus_s__Lactobacillus_reuteri
PWY_3841__folate_transformations_II_g__Lactobacillus_s__Lactobacillus_gasseri
PWY_6122__5_aminoimidazole_ribonucleotide_biosynthesis_II_g__Lactobacillus_s__Lactobacillus_
PWY_6277__superpathway_of_5_aminoimidazole_ribonucleotide_biosynthesis_g__Lactobacillus_
PWY_7208__superpathway_of_pyrimidine_nucleobases_salvage_g__Lactobacillus_s__Lactobacillu
PWY_5913__TCA_cycle_VI_(obligate_autotrophs)
PWY_7197__pyrimidine_deoxyribonucleotide_phosphorylation_g__Lactobacillus_s__Lactobacillus_
UDPNAGSYN_PWY__UDP_N_acetyl_D_glucosamine_biosynthesis_I_g__Lactobacillus_s__Lactobaci
PWY_7221__guanosine_ribonucleotides_de_novo_biosynthesis_g__Lactobacillus_s__Lactobacillus

UDPNAGSYN_PWY__UDP_N_acetyl_D_glucosamine_biosynthesis_I_g__Lactobacillus_s__Lactobac
PWY_6123__inosine_5__phosphate_biosynthesis_I_g__Lactobacillus_s__Lactobacillus_delbrueckii
UDPNAGSYN_PWY__UDP_N_acetyl_D_glucosamine_biosynthesis_I_unclassified
CALVIN_PWY__Calvin_Benson_Bassham_cycle_g__Escherichia_s__Escherichia_coli
PWY_6124__inosine_5__phosphate_biosynthesis_II_g__Lactobacillus_s__Lactobacillus_delbrueckii
P163_PWY__L_lysine_fermentation_to_acetate_and_butanoate
PWY_7198__pyrimidine_deoxyribonucleotides_de_novo_biosynthesis_IV
LACTOSECAT_PWY__lactose_and_galactose_degradation_I_g__Lactobacillus_s__Lactobacillus_dell
UDPNAGSYN_PWY__UDP_N_acetyl_D_glucosamine_biosynthesis_I_g__Lactobacillus_s__Lactobac
FERMENTATION_PWY__mixed_acid_fermentation
PWY_5097__L_lysine_biosynthesis_VI_g__Lactobacillus_s__Lactobacillus_crispatus
PWY_5920__superpathway_of_heme_biosynthesis_from_glycine
PWY_621__sucrose_degradation_III_(sucrose_invertase)_g__Lactobacillus_s__Lactobacillus_crispa
PWY_2942__L_lysine_biosynthesis_III_g__Escherichia_s__Escherichia_coli
UNINTEGRATED_g__Lactobacillus_s__Lactobacillus_delbrueckii
PWY_7221__guanosine_ribonucleotides_de_novo_biosynthesis_g__Lactobacillus_s__Lactobacillus
PWY_5154__L_arginine_biosynthesis_III_(via_N_acetyl_L_citrulline)_g__Escherichia_s__Escherichi
PWY_5097__L_lysine_biosynthesis_VI_g__Escherichia_s__Escherichia_coli
PWY_7392__taxadiene_biosynthesis_(engineered)
PWY_6121__5_aminoimidazole_ribonucleotide_biosynthesis_I_g__Lactobacillus_s__Lactobacillus_
PWY_2942__L_lysine_biosynthesis_III_g__Lactobacillus_s__Lactobacillus_crispatus
PWY_2941__L_lysine_biosynthesis_II_g__Lactobacillus_s__Lactobacillus_crispatus
PWY_4981__L_proline_biosynthesis_II_(from_arginine)_g__Lactobacillus_s__Lactobacillus_delbru
PWY_6700__queuosine_biosynthesis_g__Sutterella_s__Sutterella_wadsworthensis
COA_PWY_1__coenzyme_A_biosynthesis_II_(mammalian)_g__Lactobacillus_s__Lactobacillus_crisp
UDPNAGSYN_PWY__UDP_N_acetyl_D_glucosamine_biosynthesis_I_g__Lactobacillus_s__Lactobac
DAPLYSINESYN_PWY__L_lysine_biosynthesis_I_g__Escherichia_s__Escherichia_coli
PWY_7219__adenosine_ribonucleotides_de_novo_biosynthesis_g__Lactobacillus_s__Lactobacillus
PWY_6122__5_aminoimidazole_ribonucleotide_biosynthesis_II_g__Lactobacillus_s__Lactobacillus_
PWY_6277__superpathway_of_5_aminoimidazole_ribonucleotide_biosynthesis_g__Lactobacillus_
PWY_6151__S_adenosyl_L_methionine_cycle_I_g__Lactobacillus_s__Lactobacillus_crispatus
PWY_7117__C4_photosynthetic_carbon_assimilation_cycle,_PEPCK_type
PWY_7219__adenosine_ribonucleotides_de_novo_biosynthesis_g__Lactobacillus_s__Lactobacillus
PWY_5973__cis_vaccenate_biosynthesis_g__Lactobacillus_s__Lactobacillus_delbrueckii
PWY_7219__adenosine_ribonucleotides_de_novo_biosynthesis_g__Lactobacillus_s__Lactobacillus
PWY_7663__gondoate_biosynthesis_(anaerobic)_g__Lactobacillus_s__Lactobacillus_delbrueckii
PWY0_1061__superpathway_of_L_alanine_biosynthesis
PWY_7210__pyrimidine_deoxyribonucleotides_biosynthesis_from_CTP
GLYCOCAT_PWY__glycogen_degradation_I_(bacterial)
P161_PWY__acetylene_degradation
ARG+POLYAMINE_SYN__superpathway_of_arginine_and_polyamine_biosynthesis_g__Escherichia_
UNINTEGRATED_g__Lactobacillus_s__Lactobacillus_crispatus
P4_PWY__superpathway_of_L_lysine,_L_threonine_and_L_methionine_biosynthesis_I_g__Escheri
COA_PWY_1__coenzyme_A_biosynthesis_II_(mammalian)_g__Lactobacillus_s__Lactobacillus_helv
ARGSYNBSUB_PWY__L_arginine_biosynthesis_II_(acetyl_cycle)_g__Escherichia_s__Escherichia_co
PWY_5863__superpathway_of_phylloquinol_biosynthesis
COA_PWY_1__coenzyme_A_biosynthesis_II_(mammalian)_g__Lactobacillus_s__Lactobacillus_brev

PWY_724__superpathway_of_L_lysine,_L_threonine_and_L_methionine_biosynthesis_II_g__Esche
HEME_BIOSYNTHESIS_II__heme_biosynthesis_I_(aerobic)
PWY0_862__(5Z)_dodec_5_enoate_biosynthesis
NAGLIPASYN_PWY__lipid_IVA_biosynthesis
PWY_7400__L_arginine_biosynthesis_IV_(archaebacteria)_g__Escherichia_s__Escherichia_coli
PWY_6151__S_adenosyl_L_methionine_cycle_I_g__Lactobacillus_s__Lactobacillus_acidophilus
PWY_5897__superpathway_of_menaquinol_11_biosynthesis
PWY_5898__superpathway_of_menaquinol_12_biosynthesis
PWY_5899__superpathway_of_menaquinol_13_biosynthesis
UNINTEGRATED_g__Lactobacillus_s__Lactobacillus_brevis
UDPNAGSYN_PWY__UDP_N_acetyl_D_glucosamine_biosynthesis_I_g__Lactobacillus_s__Lactobac
PWY_7388__octanoyl_[acyl_carrier_protein]_biosynthesis_(mitochondria,_yeast)
PWY_7328__superpathway_of_UDP_glucose_derived_O_antigen_building_blocks_biosynthesis
PWY_5723__Rubisco_shunt_g__Escherichia_s__Escherichia_coli
PWY_6168__flavin_biosynthesis_III_(fungi)_g__Escherichia_s__Escherichia_coli
PWY_6703__preQ0_biosynthesis_g__Megasphaera_s__Megasphaera_micronuciformis
PWY_7664__oleate_biosynthesis_IV_(anaerobic)
HEMESYN2_PWY__heme_biosynthesis_II_(anaerobic)
NONOXIPENT_PWY__pentose_phosphate_pathway_(non_oxidative_branch)_g__Escherichia_s__Es
FASYN_INITIAL_PWY__superpathway_of_fatty_acid_biosynthesis_initiation_(E__coli)
PWY_7219__adenosine_ribonucleotides_de_novo_biosynthesis_g__Lactobacillus_s__Lactobacillus
FASYN_ELONG_PWY__fatty_acid_elongation____saturated
PWY_5097__L_lysine_biosynthesis_VI_g__Lactobacillus_s__Lactobacillus_acidophilus
NONOXIPENT_PWY__pentose_phosphate_pathway_(non_oxidative_branch)_g__Lactobacillus_s__
UDPNAGSYN_PWY__UDP_N_acetyl_D_glucosamine_biosynthesis_I_g__Lactobacillus_s__Lactobac
PWY_7446__sulfoglycolysis_g__Escherichia_s__Escherichia_coli
COA_PWY__coenzyme_A_biosynthesis_I_g__Lactobacillus_s__Lactobacillus_brevis
PWY_4242__pantothenate_and_coenzyme_A_biosynthesis_III_g__Megasphaera_s__Megasphaera
PWY_7199__pyrimidine_deoxyribonucleosides_salvage_g__Escherichia_s__Escherichia_coli
PWY_2942__L_lysine_biosynthesis_III_g__Megasphaera_s__Megasphaera_micronuciformis
PWY_7184__pyrimidine_deoxyribonucleotides_de_novo_biosynthesis_I_g__Sutterella_s__Suttere
UNINTEGRATED_g__Lactobacillus_s__Lactobacillus_acidophilus
PWY_7198__pyrimidine_deoxyribonucleotides_de_novo_biosynthesis_IV_unclassified
PWY_6122__5_aminoimidazole_ribonucleotide_biosynthesis_II_g__Megasphaera_s__Megasphaer
PWY_6277__superpathway_of_5_aminoimidazole_ribonucleotide_biosynthesis_g__Megasphaera_
HEMESYN2_PWY__heme_biosynthesis_II_(anaerobic)_g__Sutterella_s__Sutterella_wadsworthens
PWY_5097__L_lysine_biosynthesis_VI_g__Megasphaera_s__Megasphaera_micronuciformis
PWY0_1296__purine_ribonucleosides_degradation_g__Lactobacillus_s__Lactobacillus_brevis
ENTBACSYN_PWY__enterobactin_biosynthesis
PWY_6897__thiamin_salvage_II_g__Megasphaera_s__Megasphaera_micronuciformis
PWY_6147__6_hydroxymethyl_dihydropterin_diphosphate_biosynthesis_I_g__Megasphaera_s__M
COA_PWY_1__coenzyme_A_biosynthesis_II_(mammalian)_g__Megasphaera_s__Megasphaera_m
PWY0_166__superpathway_of_pyrimidine_deoxyribonucleotides_de_novo_biosynthesis_(E__coli)
PEPTIDOGLYCANSYN_PWY__peptidoglycan_biosynthesis_I_(meso_diaminopimelate_containing)_g
PWY_6317__galactose_degradation_I_(Leloir_pathway)_g__Lactobacillus_s__Lactobacillus_acidop
PWY66_422__D_galactose_degradation_V_(Leloir_pathway)_g__Lactobacillus_s__Lactobacillus_ac
PWY_6121__5_aminoimidazole_ribonucleotide_biosynthesis_I_g__Megasphaera_s__Megasphaera

PWY_6282__palmitoleate_biosynthesis_I_(from_(5Z)_dodec_5_enoate)
PWY_6386__UDP_N_acetylmuramoyl_pentapeptide_biosynthesis_II_(lysine_containing)_g__Mega
PWY_5384__sucrose_degradation_IV_(sucrose_phosphorylase)_g__Lactobacillus_s__Lactobacillus_
PWY_6387__UDP_N_acetylmuramoyl_pentapeptide_biosynthesis_I_(meso_diaminopimelate_cont
PWY_7221__guanosine_ribonucleotides_de_novo_biosynthesis_g__Lactobacillus_s__Lactobacillus
PWY_6386__UDP_N_acetylmuramoyl_pentapeptide_biosynthesis_II_(lysine_containing)_g__Lacto
PWY_7357__thiamin_formation_from_pyrithiamine_and_oxythiamine_(yeast)_g__Megasphaera_s
THISYN_PWY__superpathway_of_thiamin_diphosphate_biosynthesis_I
PWY_621__sucrose_degradation_III_(sucrose_invertase)_g__Lactobacillus_s__Lactobacillus_acido
PWY_6385__peptidoglycan_biosynthesis_III_(mycobacteria)_g__Megasphaera_s__Megasphaera_m
PWY_7539__6_hydroxymethyl_dihydropterin_diphosphate_biosynthesis_III_(Chlamydia)_g__Esch
PWY_6387__UDP_N_acetylmuramoyl_pentapeptide_biosynthesis_I_(meso_diaminopimelate_cont
ANAEROFRUCAT_PWY__homolactic_fermentation_g__Escherichia_s__Escherichia_coli
PWY_724__superpathway_of_L_lysine,_L_threonine_and_L_methionine_biosynthesis_II_g__Lacto
PWY_5686__UMP_biosynthesis_g__Megasphaera_s__Megasphaera_micronuciformis
PWY_561__superpathway_of_glyoxylate_cycle_and_fatty_acid_degradation
PWY4FS_7__phosphatidylglycerol_biosynthesis_I_(plastidic)
PWY4FS_8__phosphatidylglycerol_biosynthesis_II_(non_plastidic)
PWY_6519__8_amino_7_oxononanoate_biosynthesis_I
PWY_5667__CDP_diacylglycerol_biosynthesis_I_g__Megasphaera_s__Megasphaera_micronucifor
PWY0_1319__CDP_diacylglycerol_biosynthesis_II_g__Megasphaera_s__Megasphaera_micronucifo
PWY_6121__5_aminoimidazole_ribonucleotide_biosynthesis_I_g__Lactobacillus_s__Lactobacillus_
VALSYN_PWY__L_valine_biosynthesis_g__Megasphaera_s__Megasphaera_micronuciformis
UNINTEGRATED_g__Megasphaera_s__Megasphaera_micronuciformis
PEPTIDOGLYCANSYN_PWY__peptidoglycan_biosynthesis_I_(meso_diaminopimelate_containing)_g
PWY_5918__superpathay_of_heme_biosynthesis_from_glutamate
PWY_7210__pyrimidine_deoxyribonucleotides_biosynthesis_from_CTP_unclassified
PWY_5695__urate_biosynthesis_inosine_5__phosphate_degradation_g__Megasphaera_s__Megas
PWY_6386__UDP_N_acetylmuramoyl_pentapeptide_biosynthesis_II_(lysine_containing)_g__Lacto
PWY_6387__UDP_N_acetylmuramoyl_pentapeptide_biosynthesis_I_(meso_diaminopimelate_cont
PWY_3841__folate_transformations_II_g__Lactobacillus_s__Lactobacillus_acidophilus
PWY_5971__palmitate_biosynthesis_II_(bacteria_and_plants)
PWY_6151__S_adenosyl_L_methionine_cycle_I_g__Lactobacillus_s__Lactobacillus_brevis
NONOXIPENT_PWY__pentose_phosphate_pathway_(non_oxidative_branch)_g__Lactobacillus_s__
PWY_5100__pyruvate_fermentation_to_acetate_and_lactate_II_g__Lactobacillus_s__Lactobacillus
PWY_2942__L_lysine_biosynthesis_III_g__Lactobacillus_s__Lactobacillus_acidophilus
PWY_6608__guanosine_nucleotides_degradation_III_g__Megasphaera_s__Megasphaera_micronu
PWY_2941__L_lysine_biosynthesis_II_g__Lactobacillus_s__Lactobacillus_acidophilus
P125_PWY__superpathway_of_(R,R)_butanediol_biosynthesis
PWY_6163__chorismate_biosynthesis_from_3_dehydroquinate_g__Megasphaera_s__Megasphaer
PWY_5840__superpathway_of_menaquinol_7_biosynthesis
COA_PWY_1__coenzyme_A_biosynthesis_II_(mammalian)_g__Lactobacillus_s__Lactobacillus_acid
PWY_7229__superpathway_of_adenosine_nucleotides_de_novo_biosynthesis_I_g__Sutterella_s__
PEPTIDOGLYCANSYN_PWY__peptidoglycan_biosynthesis_I_(meso_diaminopimelate_containing)_g
PWY_7221__guanosine_ribonucleotides_de_novo_biosynthesis_g__Megasphaera_s__Megasphaer
PWY_6126__superpathway_of_adenosine_nucleotides_de_novo_biosynthesis_II_g__Sutterella_s__
PWY_7234__inosine_5__phosphate_biosynthesis_III_g__Lactobacillus_s__Lactobacillus_acidophilu

COA_PWY__coenzyme_A_biosynthesis_I_g__Megasphaera_s__Megasphaera_micronuciformis
PWY_6122__5_aminoimidazole_ribonucleotide_biosynthesis_II_g__Lactobacillus_s__Lactobacillus_
PWY_6277__superpathway_of_5_aminoimidazole_ribonucleotide_biosynthesis_g__Lactobacillus_
BIOTIN_BIOSYNTHESIS_PWY__biotin_biosynthesis_I
PWY_5188__tetrapyrrole_biosynthesis_I_(from_glutamate)_g__Sutterella_s__Sutterella_wadswor
P122_PWY__heterolactic_fermentation
PWY_7219__adenosine_ribonucleotides_de_novo_biosynthesis_g__Megasphaera_s__Megasphae
FUC_RHAMCAT_PWY__superpathway_of_fucose_and_rhamnose_degradation
PWY_7111__pyruvate_fermentation_to_isobutanol_(engineered)_g__Megasphaera_s__Megaspha
PWY_7208__superpathway_of_pyrimidine_nucleobases_salvage_g__Sutterella_s__Sutterella_wac
OANTIGEN_PWY__O_antigen_building_blocks_biosynthesis_(E__coli)_unclassified
PWY_7197__pyrimidine_deoxyribonucleotide_phosphorylation_g__Escherichia_s__Escherichia_co
PWY_6123__inosine_5__phosphate_biosynthesis_I_g__Lactobacillus_s__Lactobacillus_acidophilus
PWY66_400__glycolysis_VI_(metazoan)
PWY0_1296__purine_ribonucleosides_degradation_g__Megasphaera_s__Megasphaera_micronuc
GLYCOLYSIS__glycolysis_I_(from_glucose_6_phosphate)
PWY_6285__superpathway_of_fatty_acids_biosynthesis_(E__coli)
PWY0_41__allantoin_degradation_IV_(anaerobic)
PWY_6124__inosine_5__phosphate_biosynthesis_II_g__Lactobacillus_s__Lactobacillus_acidophilu
TEICHOICACID_PWY__teichoic_acid_(poly_glycerol)_biosynthesis
DTDPRHAMSYN_PWY__dTDP_L_rhamnose_biosynthesis_I_g__Escherichia_s__Escherichia_coli
THRESYN_PWY__superpathway_of_L_threonine_biosynthesis_g__Lactobacillus_s__Lactobacillus_a
NAD_BIOSYNTHESIS_II__NAD_salvage_pathway_II
PWY_6628__superpathway_of_L_phenylalanine_biosynthesis
PWY_5484__glycolysis_II_(from_fructose_6_phosphate)
GLUTORN_PWY__L_ornithine_biosynthesis
PWY66_389__phytol_degradation
PHOSLIPSYN_PWY__superpathway_of_phospholipid_biosynthesis_I_(bacteria)
UDPNAGSYN_PWY__UDP_N_acetyl_D_glucosamine_biosynthesis_I
PWYG_321__mycolate_biosynthesis
PWY66_409__superpathway_of_purine_nucleotide_salvage_g__Escherichia_s__Escherichia_coli
PWY_7220__adenosine_deoxyribonucleotides_de_novo_biosynthesis_II_g__Sutterella_s__Suttere
PWY_7222__guanosine_deoxyribonucleotides_de_novo_biosynthesis_II_g__Sutterella_s__Suttere
PWY0_321__phenylacetate_degradation_I_(aerobic)
SO4ASSIM_PWY__sulfate_reduction_I_(assimilatory)
ORNDEG_PWY__superpathway_of_ornithine_degradation_unclassified
PWY_7219__adenosine_ribonucleotides_de_novo_biosynthesis_g__Sutterella_s__Sutterella_wads
PEPTIDOGLYCANSYN_PWY__peptidoglycan_biosynthesis_I_(meso_diaminopimelate_containing)_g
PWY_6387__UDP_N_acetylmuramoyl_pentapeptide_biosynthesis_I_(meso_diaminopimelate_con
NAGLIPASYN_PWY__lipid_IVA_biosynthesis_g__Sutterella_s__Sutterella_wadsworthensis
PWY0_166__superpathway_of_pyrimidine_deoxyribonucleotides_de_novo_biosynthesis_(E__coli)
GOLPDLCAT_PWY__superpathway_of_glycerol_degradation_to_1,3_propanediol
LACTOSECAT_PWY__lactose_and_galactose_degradation_I_unclassified
PWY_841__superpathway_of_purine_nucleotides_de_novo_biosynthesis_I_g__Escherichia_s__Esc
ARGSYNBSUB_PWY__L_arginine_biosynthesis_II_(acetyl_cycle)
PWY_6386__UDP_N_acetylmuramoyl_pentapeptide_biosynthesis_II_(lysine_containing)_g__Sutte
METSYN_PWY__L_homoserine_and_L_methionine_biosynthesis_g__Lactobacillus_s__Lactobacillus

PWY_6124__inosine_5__phosphate_biosynthesis_II_g__Lactobacillus_s__Lactobacillus_salivarius
GLCMANNANAUT_PWY__superpathway_of_N_acetylglucosamine,_N_acetylmannosamine_and_N
P441_PWY__superpathway_of_N_acetylneuraminate_degradation
UNINTEGRATED_g__Sutterella_s__Sutterella_wadsworthensis
HEXITOLDEGSUPER_PWY__superpathway_of_hexitol_degradation_(bacteria)
PWY_6125__superpathway_of_guanosine_nucleotides_de_novo_biosynthesis_II_g__Escherichia_s
PWY_6901__superpathway_of_glucose_and_xylose_degradation_g__Escherichia_s__Escherichia_c
PWY_6121__5_aminoimidazole_ribonucleotide_biosynthesis_I_g__Sutterella_s__Sutterella_wadsw
PWY_7184__pyrimidine_deoxyribonucleotides_de_novo_biosynthesis_I_g__Escherichia_s__Escher
PWY_6122__5_aminoimidazole_ribonucleotide_biosynthesis_II_g__Sutterella_s__Sutterella_wads
PWY_6277__superpathway_of_5_aminoimidazole_ribonucleotide_biosynthesis_g__Sutterella_s__
PWY_7220__adenosine_deoxyribonucleotides_de_novo_biosynthesis_II_g__Escherichia_s__Esche
PWY_7222__guanosine_deoxyribonucleotides_de_novo_biosynthesis_II_g__Escherichia_s__Esche
PWY0_1297__superpathway_of_purine_deoxyribonucleosides_degradation
PWY_6125__superpathway_of_guanosine_nucleotides_de_novo_biosynthesis_II_g__Sutterella_s__
PWY_7229__superpathway_of_adenosine_nucleotides_de_novo_biosynthesis_I_g__Escherichia_s
PWY_7228__superpathway_of_guanosine_nucleotides_de_novo_biosynthesis_I_g__Sutterella_s__
PWY_7228__superpathway_of_guanosine_nucleotides_de_novo_biosynthesis_I_g__Escherichia_s
PWY_6628__superpathway_of_L_phenylalanine_biosynthesis_unclassified
P23_PWY__reductive_TCA_cycle_I
PWY_6284__superpathway_of_unsaturated_fatty_acids_biosynthesis_(E__coli)
PWY0_162__superpathway_of_pyrimidine_ribonucleotides_de_novo_biosynthesis_g__Escherichia
PWY_7204__pyridoxal_5__phosphate_salvage_II_(plants)
GLYCOLYSIS_E_D__superpathway_of_glycolysis_and_Entner_Doudoroff
PWY_6595__superpathway_of_guanosine_nucleotides_degradation_(plants)
PWY_6936__seleno_amino_acid_biosynthesis_g__Lactobacillus_s__Lactobacillus_salivarius
PWY0_1298__superpathway_of_pyrimidine_deoxyribonucleosides_degradation
PWY_3781__aerobic_respiration_I_(cytochrome_c)_unclassified
HEMESYN2_PWY__heme_biosynthesis_II_(anaerobic)_unclassified
PWY_5083__NAD_NADH_phosphorylation_and_dephosphorylation_unclassified
PWY_6122__5_aminoimidazole_ribonucleotide_biosynthesis_II_g__Rothia_s__Rothia_mucilaginos
PWY_6277__superpathway_of_5_aminoimidazole_ribonucleotide_biosynthesis_g__Rothia_s__Rot
PWY_6126__superpathway_of_adenosine_nucleotides_de_novo_biosynthesis_II_g__Escherichia_s
SULFATE_CYS_PWY__superpathway_of_sulfate_assimilation_and_cysteine_biosynthesis
PWY_7197__pyrimidine_deoxyribonucleotide_phosphorylation
PWY_7221__guanosine_ribonucleotides_de_novo_biosynthesis_g__Sutterella_s__Sutterella_wads
PWY66_409__superpathway_of_purine_nucleotide_salvage
HOMOSER_METSYN_PWY__L_methionine_biosynthesis_I_g__Lactobacillus_s__Lactobacillus_vagir
PWY_7184__pyrimidine_deoxyribonucleotides_de_novo_biosynthesis_I
ANAEROFRUCAT_PWY__homolactic_fermentation
PWY_7208__superpathway_of_pyrimidine_nucleobases_salvage_g__Escherichia_s__Escherichia_c
PWY_7208__superpathway_of_pyrimidine_nucleobases_salvage
PWY_5675__nitrate_reduction_V_(assimilatory)
PWY_7228__superpathway_of_guanosine_nucleotides_de_novo_biosynthesis_I
PWY0_1261__anhydromuropeptides_recycling
PWY_6125__superpathway_of_guanosine_nucleotides_de_novo_biosynthesis_II
PWY_7220__adenosine_deoxyribonucleotides_de_novo_biosynthesis_II

PWY_7222__guanosine_deoxyribonucleotides_de_novo_biosynthesis_II
PWY4FS_7__phosphatidylglycerol_biosynthesis_I_(plastidic)_unclassified
PWY4FS_8__phosphatidylglycerol_biosynthesis_II_(non_plastidic)_unclassified
PWY_6901__superpathway_of_glucose_and_xylose_degradation
PENTOSE_P_PWY__pentose_phosphate_pathway_g__Escherichia_s__Escherichia_coli
PWY_6803__phosphatidylcholine_acyl_editing_unclassified
PWY_2941__L_lysine_biosynthesis_II
PWY_6606__guanosine_nucleotides_degradation_II
COA_PWY_1__coenzyme_A_biosynthesis_II_(mammalian)_g__Sutterella_s__Sutterella_wadswort
GLUCONEO_PWY__gluconeogenesis_I
PWY_5189__tetrapyrrole_biosynthesis_II_(from_glycine)
PWY66_409__superpathway_of_purine_nucleotide_salvage_unclassified
PWY_7234__inosine_5__phosphate_biosynthesis_III
P185_PWY__formaldehyde_assimilation_III_(dihydroxyacetone_cycle)
PWY_5941__glycogen_degradation_II_(eukaryotic)
GLUCARDEG_PWY__D_glucarate_degradation_I_unclassified
PWY_5005__biotin_biosynthesis_II
PWY_5154__L_arginine_biosynthesis_III_(via_N_acetyl_L_citrulline)
PWY0_166__superpathway_of_pyrimidine_deoxyribonucleotides_de_novo_biosynthesis_(E__coli)
GALACTARDEG_PWY__D_galactarate_degradation_I_unclassified
GLUCARGALACTSUPER_PWY__superpathway_of_D_glucarate_and_D_galactarate_degradation_un
PWY_6936__seleno_amino_acid_biosynthesis
PWY_6126__superpathway_of_adenosine_nucleotides_de_novo_biosynthesis_II
PWY_7560__methylerythritol_phosphate_pathway_II
PWY_5121__superpathway_of_geranylgeranyl_diphosphate_biosynthesis_II_(via_MEP)
PWY_922__mevalonate_pathway_I_unclassified
PWY_7663__gondoate_biosynthesis_(anaerobic)
PWY_3841__folate_transformations_II_unclassified
PWY_841__superpathway_of_purine_nucleotides_de_novo_biosynthesis_I
DAPLYSINESYN_PWY__L_lysine_biosynthesis_I
PWY_6897__thiamin_salvage_II_unclassified
PWY_7229__superpathway_of_adenosine_nucleotides_de_novo_biosynthesis_I
GLUTORN_PWY__L_ornithine_biosynthesis_unclassified
PWY_6387__UDP_N_acetylmuramoyl_pentapeptide_biosynthesis_I_(meso_diaminopimelate_con
ARGSYNBSUB_PWY__L_arginine_biosynthesis_II_(acetyl_cycle)_unclassified
PWY_7388__octanoyl_[acyl_carrier_protein]_biosynthesis_(mitochondria,_yeast)_unclassified
PHOSLIPSYN_PWY__superpathway_of_phospholipid_biosynthesis_I_(bacteria)_unclassified
FASYN_INITIAL_PWY__superpathway_of_fatty_acid_biosynthesis_initiation_(E__coli)_unclassified
PWY_5791__1,4_dihydroxy_2_naphthoate_biosynthesis_II_(plants)_unclassified
PWY_5837__1,4_dihydroxy_2_naphthoate_biosynthesis_I_unclassified
PWY0_862__(5Z)_dodec_5_enoate_biosynthesis_unclassified
PWY_5100__pyruvate_fermentation_to_acetate_and_lactate_II
PWY_6386__UDP_N_acetylmuramoyl_pentapeptide_biosynthesis_II_(lysine_containing)_g__Rothi
VALSYN_PWY__L_valine_biosynthesis_g__Rothia_s__Rothia_mucilaginosa
PWY_7197__pyrimidine_deoxyribonucleotide_phosphorylation_g__Sutterella_s__Sutterella_wads
UNINTEGRATED_g__Rothia_s__Rothia_mucilaginosa
PWY_7664__oleate_biosynthesis_IV_(anaerobic)_unclassified

ARGSYN_PWY__L_arginine_biosynthesis_I_(via_L_ornithine)
PPGPPMET_PWY__ppGpp_biosynthesis
PWY_3841__folate_transformations_II
FASYN_ELONG_PWY__fatty_acid_elongation____saturated_unclassified
PWY_5189__tetrapyrrole_biosynthesis_II_(from_glycine)_g__Rothia_s__Rothia_mucilaginosa
PEPTIDOGLYCANSYN_PWY__peptidoglycan_biosynthesis_I_(meso_diaminopimelate_containing)_g
PWY_5188__tetrapyrrole_biosynthesis_I_(from_glutamate)_g__Rothia_s__Rothia_mucilaginosa
PWY_5675__nitrate_reduction_V_(assimilatory)_unclassified
PWY0_1241__ADP_L_glycero_&beta__D_manno_heptose_biosynthesis_unclassified
PWY_4981__L_proline_biosynthesis_II_(from_arginine)
PWY_6270__isoprene_biosynthesis_I
PWY_6121__5_aminoimidazole_ribonucleotide_biosynthesis_I_g__Rothia_s__Rothia_mucilaginos
PWY_6282__palmitoleate_biosynthesis_I_(from_(5Z)_dodec_5_enoate)_unclassified
PWY_6531__mannitol_cycle
PWY_6612__superpathway_of_tetrahydrofolate_biosynthesis
PWY_5973__cis_vaccenate_biosynthesis
PWY_7400__L_arginine_biosynthesis_IV_(archaebacteria)
PWY_7219__adenosine_ribonucleotides_de_novo_biosynthesis_g__Rothia_s__Rothia_mucilagino
FUCCAT_PWY__fucose_degradation
OANTIGEN_PWY__O_antigen_building_blocks_biosynthesis_(E__coli)
PWY_7279__aerobic_respiration_II_(cytochrome_c)_(yeast)_unclassified
PWY_6737__starch_degradation_V_g__Lactococcus_s__Lactococcus_lactis
FOLSYN_PWY__superpathway_of_tetrahydrofolate_biosynthesis_and_salvage
PWY_6151__S_adenosyl_L_methionine_cycle_I_g__Rothia_s__Rothia_mucilaginosa
PWY_6123__inosine_5__phosphate_biosynthesis_I
PWY_6124__inosine_5__phosphate_biosynthesis_II
P461_PWY__hexitol_fermentation_to_lactate,_formate,_ethanol_and_acetate
P461_PWY__hexitol_fermentation_to_lactate,_formate,_ethanol_and_acetate_unclassified
PWY_4242__pantothenate_and_coenzyme_A_biosynthesis_III
PWY_2942__L_lysine_biosynthesis_III_g__Rothia_s__Rothia_mucilaginosa
PWY_5723__Rubisco_shunt_unclassified
PWY490_3__nitrate_reduction_VI_(assimilatory)
PWY_6606__guanosine_nucleotides_degradation_II_g__Escherichia_s__Escherichia_coli
PWY_5097__L_lysine_biosynthesis_VI_g__Rothia_s__Rothia_mucilaginosa
PWY_5971__palmitate_biosynthesis_II_(bacteria_and_plants)_unclassified
PWY_7039__phosphatidate_metabolism,_as_a_signaling_molecule
PWY_6353__purine_nucleotides_degradation_II_(aerobic)
BRANCHED_CHAIN_AA_SYN_PWY__superpathway_of_branched_amino_acid_biosynthesis_g__Bif
PWY_6471__peptidoglycan_biosynthesis_IV_(Enterococcus_faecium)_unclassified
3_HYDROXYPHENYLACETATE_DEGRADATION_PWY__4_hydroxyphenylacetate_degradation
LACTOSECAT_PWY__lactose_and_galactose_degradation_I
PANTO_PWY__phosphopantothenate_biosynthesis_I_g__Rothia_s__Rothia_mucilaginosa
PWY_7221__guanosine_ribonucleotides_de_novo_biosynthesis
COLANSYN_PWY__colanic_acid_building_blocks_biosynthesis
PWY_5686__UMP_biosynthesis_g__Rothia_s__Rothia_mucilaginosa
PWY_7220__adenosine_deoxyribonucleotides_de_novo_biosynthesis_II_unclassified
PWY_7222__guanosine_deoxyribonucleotides_de_novo_biosynthesis_II_unclassified

UNINTEGRATED_g__Bifidobacterium_s__Bifidobacterium_longum
PWY_5103__L_isoleucine_biosynthesis_III_g__Bifidobacterium_s__Bifidobacterium_longum
PWY_5154__L_arginine_biosynthesis_III_(via_N_acetyl_L_citrulline)_unclassified
1CMET2_PWY__N10_formyl_tetrahydrofolate_biosynthesis
PWY_6876__isopropanol_biosynthesis
COA_PWY__coenzyme_A_biosynthesis_I
FAO_PWY__fatty_acid_&beta__oxidation_I
RIBOSYN2_PWY__flavin_biosynthesis_I_(bacteria_and_plants)
PWY_5690__TCA_cycle_II_(plants_and_fungi)
PWY_5676__acetyl_CoA_fermentation_to_butanoate_II
ASPASN_PWY__superpathway_of_L_aspartate_and_L_asparagine_biosynthesis
PWY_6608__guanosine_nucleotides_degradation_III
PWY_7111__pyruvate_fermentation_to_isobutanol_(engineered)_g__Lactococcus_s__Lactococcus
DTDPRHAMSYN_PWY__dTDP_L_rhamnose_biosynthesis_I_g__Bifidobacterium_s__Bifidobacteriur
PWY_7539__6_hydroxymethyl_dihydropterin_diphosphate_biosynthesis_III_(Chlamydia)
PWY_5100__pyruvate_fermentation_to_acetate_and_lactate_II_g__Actinomyces_s__Actinomyces
ILEUSYN_PWY__L_isoleucine_biosynthesis_I_(from_threonine)_g__Bifidobacterium_s__Bifidobact
VALSYN_PWY__L_valine_biosynthesis_g__Bifidobacterium_s__Bifidobacterium_longum
PWY_4702__phytate_degradation_I
PWY_5863__superpathway_of_phylloquinol_biosynthesis_unclassified
PENTOSE_P_PWY__pentose_phosphate_pathway
PWYG_321__mycolate_biosynthesis_unclassified
PWY_6936__seleno_amino_acid_biosynthesis_g__Lactococcus_s__Lactococcus_lactis
GLYOXYLATE_BYPASS__glyoxylate_cycle_unclassified
ANAGLYCOLYSIS_PWY__glycolysis_III_(from_glucose)
TCA_GLYOX_BYPASS__superpathway_of_glyoxylate_bypass_and_TCA_unclassified
PWY_5913__TCA_cycle_VI_(obligate_autotrophs)_unclassified
PWY_2942__L_lysine_biosynthesis_III
P221_PWY__octane_oxidation
PWY_7219__adenosine_ribonucleotides_de_novo_biosynthesis
PWY_5136__fatty_acid_&beta__oxidation_II_(peroxisome)
UNINTEGRATED
LACTOSECAT_PWY__lactose_and_galactose_degradation_I_g__Lactococcus_s__Lactococcus_lactis
SO4ASSIM_PWY__sulfate_reduction_I_(assimilatory)_g__Haemophilus_s__Haemophilus_parainflu
PYRIDNUCSAL_PWY__NAD_salvage_pathway_I_unclassified
COA_PWY_1__coenzyme_A_biosynthesis_II_(mammalian)
PWY_7199__pyrimidine_deoxyribonucleosides_salvage
NONMEVIPP_PWY__methylerythritol_phosphate_pathway_I_g__Actinomyces_s__Actinomyces_gr
PWY_7323__superpathway_of_GDP_mannose_derived_O_antigen_building_blocks_biosynthesis
PWY66_389__phytol_degradation_unclassified
PWY_5692__allantoin_degradation_to_glyoxylate_II
URDEGR_PWY__superpathway_of_allantoin_degradation_in_plants
PWY_6969__TCA_cycle_V_(2_oxoglutarate_ferredoxin_oxidoreductase)
PWY_7117__C4_photosynthetic_carbon_assimilation_cycle,_PEPCK_type_unclassified
GLYCOLYSIS_TCA_GLYOX_BYPASS__superpathway_of_glycolysis,_pyruvate_dehydrogenase,_TCA,_
1CMET2_PWY__N10_formyl_tetrahydrofolate_biosynthesis_unclassified
PWY_5989__stearate_biosynthesis_II_(bacteria_and_plants)

P221_PWY__octane_oxidation_unclassified
PWY_6151__S_adenosyl_L_methionine_cycle_I
COA_PWY_1__coenzyme_A_biosynthesis_II_(mammalian)_g__Bifidobacterium_s__Bifidobacteriu
PWY_5103__L_isoleucine_biosynthesis_III_g__Lactococcus_s__Lactococcus_lactis
CALVIN_PWY__Calvin_Benson_Bassham_cycle_g__Lactococcus_s__Lactococcus_lactis
BRANCHED_CHAIN_AA_SYN_PWY__superpathway_of_branched_amino_acid_biosynthesis_g__Lac
PWY_6318__L_phenylalanine_degradation_IV_(mammalian,_via_side_chain)
PWY_7663__gondoate_biosynthesis_(anaerobic)_g__Haemophilus_s__Haemophilus_parainfluenz
PWY_6318__L_phenylalanine_degradation_IV_(mammalian,_via_side_chain)_unclassified
UDPNAGSYN_PWY__UDP_N_acetyl_D_glucosamine_biosynthesis_I_g__Bifidobacterium_s__Bifido
TCA__TCA_cycle_I_(prokaryotic)
PWY_6609__adenine_and_adenosine_salvage_III_g__Lactococcus_s__Lactococcus_lactis
PWY_7663__gondoate_biosynthesis_(anaerobic)_unclassified
PWY0_1297__superpathway_of_purine_deoxyribonucleosides_degradation_unclassified
DENOVOPURINE2_PWY__superpathway_of_purine_nucleotides_de_novo_biosynthesis_II
ARGSYN_PWY__L_arginine_biosynthesis_I_(via_L_ornithine)_unclassified
PWY_5686__UMP_biosynthesis
PWY0_1296__purine_ribonucleosides_degradation_g__Lactococcus_s__Lactococcus_lactis
PWY_5973__cis_vaccenate_biosynthesis_unclassified
PWY_6700__queuosine_biosynthesis_g__Bacteroides_s__Bacteroides_caccae
PWY_5910__superpathway_of_geranylgeranyldiphosphate_biosynthesis_I_(via_mevalonate)_uncl
PWY_5897__superpathway_of_menaquinol_11_biosynthesis_unclassified
PWY_5898__superpathway_of_menaquinol_12_biosynthesis_unclassified
PWY_5899__superpathway_of_menaquinol_13_biosynthesis_unclassified
PWY_5097__L_lysine_biosynthesis_VI
PEPTIDOGLYCANSYN_PWY__peptidoglycan_biosynthesis_I_(meso_diaminopimelate_containing)
PWY_4981__L_proline_biosynthesis_II_(from_arginine)_g__Lactococcus_s__Lactococcus_lactis
PWY_5097__L_lysine_biosynthesis_VI_g__Bacteroides_s__Bacteroides_caccae
PWY_6113__superpathway_of_mycolate_biosynthesis
PWY_1269__CMP_3_deoxy_D_manno_octulosonate_biosynthesis_I_g__Bacteroides_s__Bacteroid
PWY_7219__adenosine_ribonucleotides_de_novo_biosynthesis_g__Bifidobacterium_s__Bifidobac
PWY_2942__L_lysine_biosynthesis_III_g__Bacteroides_s__Bacteroides_caccae
PWY_6123__inosine_5__phosphate_biosynthesis_I_g__Bifidobacterium_s__Bifidobacterium_long
PWY_7357__thiamin_formation_from_pyrithiamine_and_oxythiamine_(yeast)
PWY_7221__guanosine_ribonucleotides_de_novo_biosynthesis_g__Bifidobacterium_s__Bifidobac
PWY0_1297__superpathway_of_purine_deoxyribonucleosides_degradation_g__Lactococcus_s__La
PWY_6121__5_aminoimidazole_ribonucleotide_biosynthesis_I_g__Bifidobacterium_s__Bifidobact
PWY_6122__5_aminoimidazole_ribonucleotide_biosynthesis_II_g__Bifidobacterium_s__Bifidobact
PWY_6277__superpathway_of_5_aminoimidazole_ribonucleotide_biosynthesis_g__Bifidobacteriu
PWY_7219__adenosine_ribonucleotides_de_novo_biosynthesis_g__Actinomyces_s__Actinomyces
PWY_6386__UDP_N_acetylmuramoyl_pentapeptide_biosynthesis_II_(lysine_containing)
PWY_6387__UDP_N_acetylmuramoyl_pentapeptide_biosynthesis_I_(meso_diaminopimelate_con
UNINTEGRATED_g__Haemophilus_s__Haemophilus_parainfluenzae
PANTO_PWY__phosphopantothenate_biosynthesis_I_g__Bacteroides_s__Bacteroides_caccae
PWY_7111__pyruvate_fermentation_to_isobutanol_(engineered)_g__Actinomyces_s__Actinomyc
VALSYN_PWY__L_valine_biosynthesis_g__Actinomyces_s__Actinomyces_graevenitzii
UNINTEGRATED_g__Lactococcus_s__Lactococcus_lactis

PWY_5667__CDP_diacylglycerol_biosynthesis_I_g__Bacteroides_s__Bacteroides_caccae
PWY0_1319__CDP_diacylglycerol_biosynthesis_II_g__Bacteroides_s__Bacteroides_caccae
PWY_6151__S_adenosyl_L_methionine_cycle_I_g__Bifidobacterium_s__Bifidobacterium_longum
COA_PWY_1__coenzyme_A_biosynthesis_II_(mammalian)_g__Bacteroides_s__Bacteroides_caccae
PWY_7219__adenosine_ribonucleotides_de_novo_biosynthesis_g__Bacteroides_s__Bacteroides_c
PWY_621__sucrose_degradation_III_(sucrose_invertase)
PWY_5100__pyruvate_fermentation_to_acetate_and_lactate_II_g__Lactococcus_s__Lactococcus_l
PWY_6147__6_hydroxymethyl_dihydropterin_diphosphate_biosynthesis_I
RHAMCAT_PWY__L_rhamnose_degradation_I_g__Bacteroides_s__Bacteroides_caccae
PWY_724__superpathway_of_L_lysine,_L_threonine_and_L_methionine_biosynthesis_II
PWY_5686__UMP_biosynthesis_g__Bacteroides_s__Bacteroides_caccae
PWY_7400__L_arginine_biosynthesis_IV_(archaebacteria)_unclassified
PWY_6609__adenine_and_adenosine_salvage_III_g__Bacteroides_s__Bacteroides_caccae
PWY_6892__thiazole_biosynthesis_I_(E__coli)_unclassified
ARGSYNBSUB_PWY__L_arginine_biosynthesis_II_(acetyl_cycle)_g__Bifidobacterium_s__Bifidobact
PWY_7221__guanosine_ribonucleotides_de_novo_biosynthesis_g__Bacteroides_s__Bacteroides_c
NAGLIPASYN_PWY__lipid_IVA_biosynthesis_g__Haemophilus_s__Haemophilus_parainfluenzae
PWY_7039__phosphatidate_metabolism,_as_a_signaling_molecule_g__Escherichia_s__Escherichia
HOMOSER_METSYN_PWY__L_methionine_biosynthesis_I_g__Lactococcus_s__Lactococcus_lactis
PWY_5973__cis_vaccenate_biosynthesis_g__Lactococcus_s__Lactococcus_lactis
DTDPRHAMSYN_PWY__dTDP_L_rhamnose_biosynthesis_I_g__Bacteroides_s__Bacteroides_caccae
PWY_5695__urate_biosynthesis_inosine_5__phosphate_degradation_g__Bacteroides_s__Bacteroi
PWY_7111__pyruvate_fermentation_to_isobutanol_(engineered)_g__Bifidobacterium_s__Bifidoba
ARGININE_SYN4_PWY__L_ornithine_de_novo__biosynthesis_g__Bacteroides_s__Bacteroides_cacc
GLUTORN_PWY__L_ornithine_biosynthesis_g__Bifidobacterium_s__Bifidobacterium_longum
DAPLYSINESYN_PWY__L_lysine_biosynthesis_I_g__Haemophilus_s__Haemophilus_parainfluenzae
PWY_7663__gondoate_biosynthesis_(anaerobic)_g__Bacteroides_s__Bacteroides_caccae
PWY_7115__C4_photosynthetic_carbon_assimilation_cycle,_NAD_ME_type
PWY_7111__pyruvate_fermentation_to_isobutanol_(engineered)_g__Bacteroides_s__Bacteroides
VALSYN_PWY__L_valine_biosynthesis_g__Bacteroides_s__Bacteroides_caccae
THRESYN_PWY__superpathway_of_L_threonine_biosynthesis_g__Actinomyces_s__Actinomyces_g
PWY_5686__UMP_biosynthesis_g__Actinomyces_s__Actinomyces_graevenitzii
PWY66_422__D_galactose_degradation_V_(Leloir_pathway)
PWY_6122__5_aminoimidazole_ribonucleotide_biosynthesis_II
PWY_6277__superpathway_of_5_aminoimidazole_ribonucleotide_biosynthesis
HISTSYN_PWY__L_histidine_biosynthesis_g__Bifidobacterium_s__Bifidobacterium_longum
PWY_5345__superpathway_of_L_methionine_biosynthesis_(by_sulfhydrylation)
PWY_1042__glycolysis_IV_(plant_cytosol)_g__Bacteroides_s__Bacteroides_caccae
ARGSYN_PWY__L_arginine_biosynthesis_I_(via_L_ornithine)_g__Bifidobacterium_s__Bifidobacteri
PWY_7400__L_arginine_biosynthesis_IV_(archaebacteria)_g__Bifidobacterium_s__Bifidobacteriun
UNINTEGRATED_g__Adlercreutzia_s__Adlercreutzia_equolifaciens
UNINTEGRATED_g__Akkermansia_s__Akkermansia_muciniphila
UNINTEGRATED_g__Alistipes_s__Alistipes_finegoldii
UNINTEGRATED_g__Alistipes_s__Alistipes_indistinctus
UNINTEGRATED_g__Alistipes_s__Alistipes_onderdonkii
UNINTEGRATED_g__Alistipes_s__Alistipes_putredinis
UNINTEGRATED_g__Alistipes_s__Alistipes_senegalensis

UNINTEGRATED_g__Alistipes_s__Alistipes_shahii
UNINTEGRATED_g__Alistipes_s__Alistipes_sp_HGB5
UNINTEGRATED_g__Anaerofustis_s__Anaerofustis_stercorihominis
UNINTEGRATED_g__Anaerostipes_s__Anaerostipes_hadrus
UNINTEGRATED_g__Anaerotruncus_s__Anaerotruncus_colihominis
UNINTEGRATED_g__Bacteroides_s__Bacteroides_cellulosilyticus
UNINTEGRATED_g__Bacteroides_s__Bacteroides_clarus
UNINTEGRATED_g__Bacteroides_s__Bacteroides_coprocola
UNINTEGRATED_g__Bacteroides_s__Bacteroides_dorei
UNINTEGRATED_g__Bacteroides_s__Bacteroides_eggerthii
UNINTEGRATED_g__Bacteroides_s__Bacteroides_faecis
UNINTEGRATED_g__Bacteroides_s__Bacteroides_finegoldii
UNINTEGRATED_g__Bacteroides_s__Bacteroides_intestinalis
UNINTEGRATED_g__Bacteroides_s__Bacteroides_massiliensis
UNINTEGRATED_g__Bacteroides_s__Bacteroides_ovatus
UNINTEGRATED_g__Bacteroides_s__Bacteroides_plebeius
UNINTEGRATED_g__Bacteroides_s__Bacteroides_salyersiae
UNINTEGRATED_g__Bacteroides_s__Bacteroides_sp_1_1_30
UNINTEGRATED_g__Bacteroides_s__Bacteroides_sp_1_1_6
UNINTEGRATED_g__Bacteroides_s__Bacteroides_sp_2_1_22
UNINTEGRATED_g__Bacteroides_s__Bacteroides_sp_2_2_4
UNINTEGRATED_g__Bacteroides_s__Bacteroides_sp_3_1_19
UNINTEGRATED_g__Bacteroides_s__Bacteroides_sp_3_1_23
UNINTEGRATED_g__Bacteroides_s__Bacteroides_sp_3_1_33FAA
UNINTEGRATED_g__Bacteroides_s__Bacteroides_sp_3_1_40A
UNINTEGRATED_g__Bacteroides_s__Bacteroides_sp_4_3_47FAA
UNINTEGRATED_g__Bacteroides_s__Bacteroides_sp_9_1_42FAA
UNINTEGRATED_g__Bacteroides_s__Bacteroides_sp_D20
UNINTEGRATED_g__Bacteroides_s__Bacteroides_stercoris
UNINTEGRATED_g__Bacteroides_s__Bacteroides_thetaiotaomicron
UNINTEGRATED_g__Bacteroides_s__Bacteroides_uniformis
UNINTEGRATED_g__Bacteroides_s__Bacteroides_xylanisolvens
UNINTEGRATED_g__Barnesiella_s__Barnesiella_intestinihominis
UNINTEGRATED_g__Bifidobacterium_s__Bifidobacterium_angulatum
UNINTEGRATED_g__Bifidobacterium_s__Bifidobacterium_animalis
UNINTEGRATED_g__Bifidobacterium_s__Bifidobacterium_bifidum
UNINTEGRATED_g__Bifidobacterium_s__Bifidobacterium_catenulatum
UNINTEGRATED_g__Bifidobacterium_s__Bifidobacterium_dentium
UNINTEGRATED_g__Bifidobacterium_s__Bifidobacterium_pseudocatenulatum
UNINTEGRATED_g__Bilophila_s__Bilophila_wadsworthia
UNINTEGRATED_g__Blautia_s__Blautia_hydrogenotrophica
UNINTEGRATED_g__Blautia_s__Blautia_sp_KLE_1732
UNINTEGRATED_g__Blautia_s__Ruminococcus_gnavus
UNINTEGRATED_g__Blautia_s__Ruminococcus_obeum
UNINTEGRATED_g__Burkholderiales_noname_s__Burkholderiales_bacterium_1_1_47
UNINTEGRATED_g__Butyrivibrio_s__Butyrivibrio_crossotus
UNINTEGRATED_g__Catenibacterium_s__Catenibacterium_mitsuokai

UNINTEGRATED_g__Citrobacter_s__Citrobacter_freundii
UNINTEGRATED_g__Clostridiales_noname_s__Bacteroides_pectinophilus
UNINTEGRATED_g__Clostridiales_noname_s__Clostridiales_bacterium_1_7_47FAA
UNINTEGRATED_g__Clostridium_s__Clostridium_asparagiforme
UNINTEGRATED_g__Clostridium_s__Clostridium_bolteae
UNINTEGRATED_g__Clostridium_s__Clostridium_citroniae
UNINTEGRATED_g__Clostridium_s__Clostridium_clostridioforme
UNINTEGRATED_g__Clostridium_s__Clostridium_hathewayi
UNINTEGRATED_g__Clostridium_s__Clostridium_leptum
UNINTEGRATED_g__Clostridium_s__Clostridium_perfringens
UNINTEGRATED_g__Clostridium_s__Clostridium_sp_L2_50
UNINTEGRATED_g__Clostridium_s__Clostridium_sp_SS2_1
UNINTEGRATED_g__Clostridium_s__Clostridium_symbiosum
UNINTEGRATED_g__Collinsella_s__Collinsella_intestinalis
UNINTEGRATED_g__Collinsella_s__Collinsella_tanakaei
UNINTEGRATED_g__Coprobacter_s__Coprobacter_fastidiosus
UNINTEGRATED_g__Coprococcus_s__Coprococcus_catus
UNINTEGRATED_g__Coprococcus_s__Coprococcus_eutactus
UNINTEGRATED_g__Coprococcus_s__Coprococcus_sp_ART55_1
UNINTEGRATED_g__Dialister_s__Dialister_invisus
UNINTEGRATED_g__Dorea_s__Dorea_formicigenerans
UNINTEGRATED_g__Eggerthella_s__Eggerthella_lenta
UNINTEGRATED_g__Eggerthella_s__Eggerthella_sp_1_3_56FAA
UNINTEGRATED_g__Eggerthella_s__Eggerthella_sp_HGA1
UNINTEGRATED_g__Enterobacter_s__Enterobacter_cloacae
UNINTEGRATED_g__Enterococcus_s__Enterococcus_durans
UNINTEGRATED_g__Enterococcus_s__Enterococcus_faecalis
UNINTEGRATED_g__Enterococcus_s__Enterococcus_faecium
UNINTEGRATED_g__Enterorhabdus_s__Enterorhabdus_caecimuris
UNINTEGRATED_g__Erysipelotrichaceae_noname_s__Clostridium_spiroforme
UNINTEGRATED_g__Erysipelotrichaceae_noname_s__Erysipelotrichaceae_bacterium_21_3
UNINTEGRATED_g__Erysipelotrichaceae_noname_s__Erysipelotrichaceae_bacterium_2_2_44A
UNINTEGRATED_g__Erysipelotrichaceae_noname_s__Eubacterium_biforme
UNINTEGRATED_g__Erysipelotrichaceae_noname_s__Eubacterium_cylindroides
UNINTEGRATED_g__Eubacterium_s__Eubacterium_eligens
UNINTEGRATED_g__Eubacterium_s__Eubacterium_hallii
UNINTEGRATED_g__Eubacterium_s__Eubacterium_ramulus
UNINTEGRATED_g__Eubacterium_s__Eubacterium_siraeum
UNINTEGRATED_g__Eubacterium_s__Eubacterium_sp_3_1_31
UNINTEGRATED_g__Eubacterium_s__Eubacterium_ventriosum
UNINTEGRATED_g__Fusobacterium_s__Fusobacterium_nucleatum
UNINTEGRATED_g__Gemella_s__Gemella_sanguinis
UNINTEGRATED_g__Gordonibacter_s__Gordonibacter_pamelaeae
UNINTEGRATED_g__Holdemania_s__Holdemania_filiformis
UNINTEGRATED_g__Klebsiella_s__Klebsiella_pneumoniae
UNINTEGRATED_g__Lachnospiraceae_noname_s__Lachnospiraceae_bacterium_1_1_57FAA
UNINTEGRATED_g__Lachnospiraceae_noname_s__Lachnospiraceae_bacterium_1_4_56FAA

UNINTEGRATED_g__Lachnospiraceae_noname_s__Lachnospiraceae_bacterium_2_1_58FAA
UNINTEGRATED_g__Lachnospiraceae_noname_s__Lachnospiraceae_bacterium_3_1_46FAA
UNINTEGRATED_g__Lachnospiraceae_noname_s__Lachnospiraceae_bacterium_3_1_57FAA_CT1
UNINTEGRATED_g__Lachnospiraceae_noname_s__Lachnospiraceae_bacterium_5_1_63FAA
UNINTEGRATED_g__Lachnospiraceae_noname_s__Lachnospiraceae_bacterium_7_1_58FAA
UNINTEGRATED_g__Lachnospiraceae_noname_s__Lachnospiraceae_bacterium_9_1_43BFAA
UNINTEGRATED_g__Lactobacillus_s__Lactobacillus_casei_paracasei
UNINTEGRATED_g__Lactobacillus_s__Lactobacillus_fermentum
UNINTEGRATED_g__Lactobacillus_s__Lactobacillus_rhamnosus
UNINTEGRATED_g__Lactobacillus_s__Lactobacillus_ruminis
UNINTEGRATED_g__Nakaseomyces_s__Candida_glabrata
UNINTEGRATED_g__Odoribacter_s__Odoribacter_laneus
UNINTEGRATED_g__Odoribacter_s__Odoribacter_splanchnicus
UNINTEGRATED_g__Oxalobacter_s__Oxalobacter_formigenes
UNINTEGRATED_g__Parabacteroides_s__Parabacteroides_distasonis
UNINTEGRATED_g__Parabacteroides_s__Parabacteroides_goldsteinii
UNINTEGRATED_g__Parabacteroides_s__Parabacteroides_johnsonii
UNINTEGRATED_g__Parabacteroides_s__Parabacteroides_merdae
UNINTEGRATED_g__Parabacteroides_s__Parabacteroides_sp_20_3
UNINTEGRATED_g__Parabacteroides_s__Parabacteroides_sp_D13
UNINTEGRATED_g__Paraprevotella_s__Paraprevotella_clara
UNINTEGRATED_g__Paraprevotella_s__Paraprevotella_xylaniphila
UNINTEGRATED_g__Parasutterella_s__Parasutterella_excrementihominis
UNINTEGRATED_g__Pediococcus_s__Pediococcus_acidilactici
UNINTEGRATED_g__Pediococcus_s__Pediococcus_lolii
UNINTEGRATED_g__Peptostreptococcaceae_noname_s__Clostridium_bartlettii
UNINTEGRATED_g__Phascolarctobacterium_s__Phascolarctobacterium_succinatutens
UNINTEGRATED_g__Prevotella_s__Prevotella_copri
UNINTEGRATED_g__Propionibacterium_s__Propionibacterium_freudenreichii
UNINTEGRATED_g__Roseburia_s__Roseburia_hominis
UNINTEGRATED_g__Roseburia_s__Roseburia_inulinivorans
UNINTEGRATED_g__Ruminococcaceae_noname_s__Ruminococcaceae_bacterium_D16
UNINTEGRATED_g__Ruminococcus_s__Ruminococcus_callidus
UNINTEGRATED_g__Ruminococcus_s__Ruminococcus_champanellensis
UNINTEGRATED_g__Ruminococcus_s__Ruminococcus_sp
UNINTEGRATED_g__Ruminococcus_s__Ruminococcus_sp_JC304
UNINTEGRATED_g__Slackia_s__Slackia_piriformis
UNINTEGRATED_g__Solobacterium_s__Solobacterium_moorei
UNINTEGRATED_g__Streptococcus_s__Streptococcus_australis
UNINTEGRATED_g__Streptococcus_s__Streptococcus_mitis_oralis_pneumoniae
UNINTEGRATED_g__Streptococcus_s__Streptococcus_sanguinis
UNINTEGRATED_g__Subdoligranulum_s__Subdoligranulum_sp_4_3_54A2FAA
UNINTEGRATED_g__Veillonella_s__Veillonella_dispar
UNINTEGRATED_g__Veillonella_s__Veillonella_parvula
1CMET2_PWY__N10_formyl_tetrahydrofolate_biosynthesis_g__Akkermansia_s__Akkermansia_mu
1CMET2_PWY__N10_formyl_tetrahydrofolate_biosynthesis_g__Bacteroides_s__Bacteroides_cellu
1CMET2_PWY__N10_formyl_tetrahydrofolate_biosynthesis_g__Bacteroides_s__Bacteroides_claru

1CMET2_PWY__N10_formyl_tetrahydrofolate_biosynthesis_g__Bacteroides_s__Bacteroides_copr
1CMET2_PWY__N10_formyl_tetrahydrofolate_biosynthesis_g__Bacteroides_s__Bacteroides_faeci
1CMET2_PWY__N10_formyl_tetrahydrofolate_biosynthesis_g__Bacteroides_s__Bacteroides_fineg
1CMET2_PWY__N10_formyl_tetrahydrofolate_biosynthesis_g__Bacteroides_s__Bacteroides_fragil
1CMET2_PWY__N10_formyl_tetrahydrofolate_biosynthesis_g__Bacteroides_s__Bacteroides_intes
1CMET2_PWY__N10_formyl_tetrahydrofolate_biosynthesis_g__Bacteroides_s__Bacteroides_mass
1CMET2_PWY__N10_formyl_tetrahydrofolate_biosynthesis_g__Bacteroides_s__Bacteroides_ovatu
1CMET2_PWY__N10_formyl_tetrahydrofolate_biosynthesis_g__Bacteroides_s__Bacteroides_sp_1_
1CMET2_PWY__N10_formyl_tetrahydrofolate_biosynthesis_g__Bacteroides_s__Bacteroides_sp_1_
1CMET2_PWY__N10_formyl_tetrahydrofolate_biosynthesis_g__Bacteroides_s__Bacteroides_sp_2_
1CMET2_PWY__N10_formyl_tetrahydrofolate_biosynthesis_g__Bacteroides_s__Bacteroides_sp_3_
1CMET2_PWY__N10_formyl_tetrahydrofolate_biosynthesis_g__Bacteroides_s__Bacteroides_sp_D
1CMET2_PWY__N10_formyl_tetrahydrofolate_biosynthesis_g__Bacteroides_s__Bacteroides_sterc
1CMET2_PWY__N10_formyl_tetrahydrofolate_biosynthesis_g__Bacteroides_s__Bacteroides_theta
1CMET2_PWY__N10_formyl_tetrahydrofolate_biosynthesis_g__Bacteroides_s__Bacteroides_unifo
1CMET2_PWY__N10_formyl_tetrahydrofolate_biosynthesis_g__Bacteroides_s__Bacteroides_xylar
1CMET2_PWY__N10_formyl_tetrahydrofolate_biosynthesis_g__Citrobacter_s__Citrobacter_freund
1CMET2_PWY__N10_formyl_tetrahydrofolate_biosynthesis_g__Klebsiella_s__Klebsiella_pneumon
1CMET2_PWY__N10_formyl_tetrahydrofolate_biosynthesis_g__Lactococcus_s__Lactococcus_lactis
1CMET2_PWY__N10_formyl_tetrahydrofolate_biosynthesis_g__Parabacteroides_s__Parabacteroid
1CMET2_PWY__N10_formyl_tetrahydrofolate_biosynthesis_g__Parabacteroides_s__Parabacteroid
1CMET2_PWY__N10_formyl_tetrahydrofolate_biosynthesis_g__Parabacteroides_s__Parabacteroid
1CMET2_PWY__N10_formyl_tetrahydrofolate_biosynthesis_g__Parabacteroides_s__Parabacteroid
1CMET2_PWY__N10_formyl_tetrahydrofolate_biosynthesis_g__Parabacteroides_s__Parabacteroid
1CMET2_PWY__N10_formyl_tetrahydrofolate_biosynthesis_g__Parabacteroides_s__Parabacteroid
3_HYDROXYPHENYLACETATE_DEGRADATION_PWY__4_hydroxyphenylacetate_degradation_g__Esc
ALL_CHORISMATE_PWY__superpathway_of_chorismate_metabolism
ALLANTOINDEG_PWY__superpathway_of_allantoin_degradation_in_yeast
ALLANTOINDEG_PWY__superpathway_of_allantoin_degradation_in_yeast_unclassified
ANAEROFRUCAT_PWY__homolactic_fermentation_g__Lactococcus_s__Lactococcus_lactis
ANAGLYCOLYSIS_PWY__glycolysis_III_(from_glucose)_g__Alistipes_s__Alistipes_senegalensis
ANAGLYCOLYSIS_PWY__glycolysis_III_(from_glucose)_g__Alistipes_s__Alistipes_shahii
ANAGLYCOLYSIS_PWY__glycolysis_III_(from_glucose)_g__Bacteroides_s__Bacteroides_ovatus
ANAGLYCOLYSIS_PWY__glycolysis_III_(from_glucose)_g__Bacteroides_s__Bacteroides_sp_1_1_30
ANAGLYCOLYSIS_PWY__glycolysis_III_(from_glucose)_g__Bacteroides_s__Bacteroides_sp_2_1_22
ANAGLYCOLYSIS_PWY__glycolysis_III_(from_glucose)_g__Bacteroides_s__Bacteroides_sp_2_2_4
ANAGLYCOLYSIS_PWY__glycolysis_III_(from_glucose)_g__Bacteroides_s__Bacteroides_sp_3_1_23
ANAGLYCOLYSIS_PWY__glycolysis_III_(from_glucose)_g__Bacteroides_s__Bacteroides_xylanisolve
ANAGLYCOLYSIS_PWY__glycolysis_III_(from_glucose)_g__Citrobacter_s__Citrobacter_freundii
ANAGLYCOLYSIS_PWY__glycolysis_III_(from_glucose)_g__Fusobacterium_s__Fusobacterium_nucle
ANAGLYCOLYSIS_PWY__glycolysis_III_(from_glucose)_g__Klebsiella_s__Klebsiella_pneumoniae
ANAGLYCOLYSIS_PWY__glycolysis_III_(from_glucose)_g__Lactococcus_s__Lactococcus_lactis
ANAGLYCOLYSIS_PWY__glycolysis_III_(from_glucose)_g__Odoribacter_s__Odoribacter_splanchnic
ANAGLYCOLYSIS_PWY__glycolysis_III_(from_glucose)_g__Pediococcus_s__Pediococcus_acidilactici
ANAGLYCOLYSIS_PWY__glycolysis_III_(from_glucose)_g__Pediococcus_s__Pediococcus_lolii
ANAGLYCOLYSIS_PWY__glycolysis_III_(from_glucose)_g__Streptococcus_s__Streptococcus_austral
ANAGLYCOLYSIS_PWY__glycolysis_III_(from_glucose)_g__Streptococcus_s__Streptococcus_mitis_

ARGDEG_PWY__superpathway_of_L_arginine,_putrescine,_and_4_aminobutanoate_degradation_
ARGININE_SYN4_PWY__L_ornithine_de_novo__biosynthesis_g__Bacteroides_s__Bacteroides_cell
ARGININE_SYN4_PWY__L_ornithine_de_novo__biosynthesis_g__Bacteroides_s__Bacteroides_clar
ARGININE_SYN4_PWY__L_ornithine_de_novo__biosynthesis_g__Bacteroides_s__Bacteroides_faec
ARGININE_SYN4_PWY__L_ornithine_de_novo__biosynthesis_g__Bacteroides_s__Bacteroides_fine
ARGININE_SYN4_PWY__L_ornithine_de_novo__biosynthesis_g__Bacteroides_s__Bacteroides_inte
ARGININE_SYN4_PWY__L_ornithine_de_novo__biosynthesis_g__Bacteroides_s__Bacteroides_mas
ARGININE_SYN4_PWY__L_ornithine_de_novo__biosynthesis_g__Bacteroides_s__Bacteroides_ova
ARGININE_SYN4_PWY__L_ornithine_de_novo__biosynthesis_g__Bacteroides_s__Bacteroides_saly
ARGININE_SYN4_PWY__L_ornithine_de_novo__biosynthesis_g__Bacteroides_s__Bacteroides_sp_
ARGININE_SYN4_PWY__L_ornithine_de_novo__biosynthesis_g__Bacteroides_s__Bacteroides_sp_
ARGININE_SYN4_PWY__L_ornithine_de_novo__biosynthesis_g__Bacteroides_s__Bacteroides_sp_
ARGININE_SYN4_PWY__L_ornithine_de_novo__biosynthesis_g__Bacteroides_s__Bacteroides_sp_
ARGININE_SYN4_PWY__L_ornithine_de_novo__biosynthesis_g__Bacteroides_s__Bacteroides_sp_
ARGININE_SYN4_PWY__L_ornithine_de_novo__biosynthesis_g__Bacteroides_s__Bacteroides_sp_
ARGININE_SYN4_PWY__L_ornithine_de_novo__biosynthesis_g__Bacteroides_s__Bacteroides_ster
ARGININE_SYN4_PWY__L_ornithine_de_novo__biosynthesis_g__Bacteroides_s__Bacteroides_thet
ARGININE_SYN4_PWY__L_ornithine_de_novo__biosynthesis_g__Bacteroides_s__Bacteroides_unif
ARGININE_SYN4_PWY__L_ornithine_de_novo__biosynthesis_g__Bacteroides_s__Bacteroides_xyla
ARGORNPROST_PWY__arginine,_ornithine_and_proline_interconversion_g__Enterococcus_s__Ent
ARGSYN_PWY__L_arginine_biosynthesis_I_(via_L_ornithine)_g__Burkholderiales_noname_s__Bur
ARGSYN_PWY__L_arginine_biosynthesis_I_(via_L_ornithine)_g__Citrobacter_s__Citrobacter_freur
ARGSYN_PWY__L_arginine_biosynthesis_I_(via_L_ornithine)_g__Coprococcus_s__Coprococcus_ca
ARGSYN_PWY__L_arginine_biosynthesis_I_(via_L_ornithine)_g__Eubacterium_s__Eubacterium_sir
ARGSYN_PWY__L_arginine_biosynthesis_I_(via_L_ornithine)_g__Klebsiella_s__Klebsiella_pneumo
ARGSYN_PWY__L_arginine_biosynthesis_I_(via_L_ornithine)_g__Lactococcus_s__Lactococcus_lact
ARGSYN_PWY__L_arginine_biosynthesis_I_(via_L_ornithine)_g__Oxalobacter_s__Oxalobacter_forr
ARGSYN_PWY__L_arginine_biosynthesis_I_(via_L_ornithine)_g__Parasutterella_s__Parasutterella_
ARGSYN_PWY__L_arginine_biosynthesis_I_(via_L_ornithine)_g__Ruminococcus_s__Ruminococcus
ARGSYN_PWY__L_arginine_biosynthesis_I_(via_L_ornithine)_g__Veillonella_s__Veillonella_dispar
ARGSYN_PWY__L_arginine_biosynthesis_I_(via_L_ornithine)_g__Veillonella_s__Veillonella_parvul
ARGSYNBSUB_PWY__L_arginine_biosynthesis_II_(acetyl_cycle)_g__Burkholderiales_noname_s__B
ARGSYNBSUB_PWY__L_arginine_biosynthesis_II_(acetyl_cycle)_g__Coprococcus_s__Coprococcus_
ARGSYNBSUB_PWY__L_arginine_biosynthesis_II_(acetyl_cycle)_g__Eubacterium_s__Eubacterium_
ARGSYNBSUB_PWY__L_arginine_biosynthesis_II_(acetyl_cycle)_g__Lactococcus_s__Lactococcus_la
ARGSYNBSUB_PWY__L_arginine_biosynthesis_II_(acetyl_cycle)_g__Oxalobacter_s__Oxalobacter_f
ARGSYNBSUB_PWY__L_arginine_biosynthesis_II_(acetyl_cycle)_g__Parasutterella_s__Parasutterel
ARGSYNBSUB_PWY__L_arginine_biosynthesis_II_(acetyl_cycle)_g__Ruminococcus_s__Ruminococc
ARO_PWY__chorismate_biosynthesis_I_g__Akkermansia_s__Akkermansia_muciniphila
ARO_PWY__chorismate_biosynthesis_I_g__Alistipes_s__Alistipes_finegoldii
ARO_PWY__chorismate_biosynthesis_I_g__Alistipes_s__Alistipes_sp_HGB5
ARO_PWY__chorismate_biosynthesis_I_g__Anaerostipes_s__Anaerostipes_hadrus
ARO_PWY__chorismate_biosynthesis_I_g__Anaerotruncus_s__Anaerotruncus_colihominis
ARO_PWY__chorismate_biosynthesis_I_g__Bacteroides_s__Bacteroides_fragilis
ARO_PWY__chorismate_biosynthesis_I_g__Blautia_s__Blautia_sp_KLE_1732
ARO_PWY__chorismate_biosynthesis_I_g__Blautia_s__Ruminococcus_gnavus
ARO_PWY__chorismate_biosynthesis_I_g__Blautia_s__Ruminococcus_obeum

ARO_PWY__chorismate_biosynthesis_I_g__Burkholderiales_noname_s__Burkholderiales_bacteriu
ARO_PWY__chorismate_biosynthesis_I_g__Butyrivibrio_s__Butyrivibrio_crossotus
ARO_PWY__chorismate_biosynthesis_I_g__Citrobacter_s__Citrobacter_freundii
ARO_PWY__chorismate_biosynthesis_I_g__Clostridiales_noname_s__Clostridiales_bacterium_1_7
ARO_PWY__chorismate_biosynthesis_I_g__Clostridium_s__Clostridium_asparagiforme
ARO_PWY__chorismate_biosynthesis_I_g__Clostridium_s__Clostridium_bolteae
ARO_PWY__chorismate_biosynthesis_I_g__Clostridium_s__Clostridium_clostridioforme
ARO_PWY__chorismate_biosynthesis_I_g__Clostridium_s__Clostridium_hathewayi
ARO_PWY__chorismate_biosynthesis_I_g__Clostridium_s__Clostridium_sp_L2_50
ARO_PWY__chorismate_biosynthesis_I_g__Clostridium_s__Clostridium_sp_SS2_1
ARO_PWY__chorismate_biosynthesis_I_g__Clostridium_s__Clostridium_symbiosum
ARO_PWY__chorismate_biosynthesis_I_g__Dorea_s__Dorea_formicigenerans
ARO_PWY__chorismate_biosynthesis_I_g__Dorea_s__Dorea_longicatena
ARO_PWY__chorismate_biosynthesis_I_g__Erysipelotrichaceae_noname_s__Eubacterium_biforme
ARO_PWY__chorismate_biosynthesis_I_g__Eubacterium_s__Eubacterium_eligens
ARO_PWY__chorismate_biosynthesis_I_g__Eubacterium_s__Eubacterium_siraeum
ARO_PWY__chorismate_biosynthesis_I_g__Eubacterium_s__Eubacterium_ventriosum
ARO_PWY__chorismate_biosynthesis_I_g__Klebsiella_s__Klebsiella_pneumoniae
ARO_PWY__chorismate_biosynthesis_I_g__Lachnospiraceae_noname_s__Lachnospiraceae_bacter
ARO_PWY__chorismate_biosynthesis_I_g__Lachnospiraceae_noname_s__Lachnospiraceae_bacter
ARO_PWY__chorismate_biosynthesis_I_g__Lachnospiraceae_noname_s__Lachnospiraceae_bacter
ARO_PWY__chorismate_biosynthesis_I_g__Lachnospiraceae_noname_s__Lachnospiraceae_bacter
ARO_PWY__chorismate_biosynthesis_I_g__Lactococcus_s__Lactococcus_lactis
ARO_PWY__chorismate_biosynthesis_I_g__Parasutterella_s__Parasutterella_excrementihominis
ARO_PWY__chorismate_biosynthesis_I_g__Peptostreptococcaceae_noname_s__Clostridium_bartl
ARO_PWY__chorismate_biosynthesis_I_g__Phascolarctobacterium_s__Phascolarctobacterium_suc
ARO_PWY__chorismate_biosynthesis_I_g__Propionibacterium_s__Propionibacterium_freudenreic
ARO_PWY__chorismate_biosynthesis_I_g__Roseburia_s__Roseburia_hominis
ARO_PWY__chorismate_biosynthesis_I_g__Roseburia_s__Roseburia_inulinivorans
ARO_PWY__chorismate_biosynthesis_I_g__Ruminococcus_s__Ruminococcus_champanellensis
ASPASN_PWY__superpathway_of_L_aspartate_and_L_asparagine_biosynthesis_g__Bacteroides_s_
ASPASN_PWY__superpathway_of_L_aspartate_and_L_asparagine_biosynthesis_g__Bacteroides_s_
ASPASN_PWY__superpathway_of_L_aspartate_and_L_asparagine_biosynthesis_g__Bacteroides_s_
ASPASN_PWY__superpathway_of_L_aspartate_and_L_asparagine_biosynthesis_g__Bacteroides_s_
ASPASN_PWY__superpathway_of_L_aspartate_and_L_asparagine_biosynthesis_g__Bacteroides_s_
ASPASN_PWY__superpathway_of_L_aspartate_and_L_asparagine_biosynthesis_g__Bacteroides_s_
ASPASN_PWY__superpathway_of_L_aspartate_and_L_asparagine_biosynthesis_g__Bacteroides_s_
ASPASN_PWY__superpathway_of_L_aspartate_and_L_asparagine_biosynthesis_g__Bacteroides_s_
ASPASN_PWY__superpathway_of_L_aspartate_and_L_asparagine_biosynthesis_g__Bacteroides_s_
ASPASN_PWY__superpathway_of_L_aspartate_and_L_asparagine_biosynthesis_g__Bacteroides_s_
ASPASN_PWY__superpathway_of_L_aspartate_and_L_asparagine_biosynthesis_g__Citrobacter_s_
ASPASN_PWY__superpathway_of_L_aspartate_and_L_asparagine_biosynthesis_g__Enterobacter_
ASPASN_PWY__superpathway_of_L_aspartate_and_L_asparagine_biosynthesis_g__Lactobacillus_
ASPASN_PWY__superpathway_of_L_aspartate_and_L_asparagine_biosynthesis_g__Odoribacter_s
ASPASN_PWY__superpathway_of_L_aspartate_and_L_asparagine_biosynthesis_g__Propionibacter
AST_PWY__L_arginine_degradation_II_(AST_pathway)_unclassified
BIOTIN_BIOSYNTHESIS_PWY__biotin_biosynthesis_I_g__Citrobacter_s__Citrobacter_freundii

BIOTIN_BIOSYNTHESIS_PWY__biotin_biosynthesis_I_g__Haemophilus_s__Haemophilus_parainflue
BIOTIN_BIOSYNTHESIS_PWY__biotin_biosynthesis_I_g__Klebsiella_s__Klebsiella_pneumoniae
BRANCHED_CHAIN_AA_SYN_PWY__superpathway_of_branched_amino_acid_biosynthesis_g__Ak
BRANCHED_CHAIN_AA_SYN_PWY__superpathway_of_branched_amino_acid_biosynthesis_g__An
BRANCHED_CHAIN_AA_SYN_PWY__superpathway_of_branched_amino_acid_biosynthesis_g__Bla
BRANCHED_CHAIN_AA_SYN_PWY__superpathway_of_branched_amino_acid_biosynthesis_g__Bla
BRANCHED_CHAIN_AA_SYN_PWY__superpathway_of_branched_amino_acid_biosynthesis_g__Clo
BRANCHED_CHAIN_AA_SYN_PWY__superpathway_of_branched_amino_acid_biosynthesis_g__Clo
BRANCHED_CHAIN_AA_SYN_PWY__superpathway_of_branched_amino_acid_biosynthesis_g__Co
BRANCHED_CHAIN_AA_SYN_PWY__superpathway_of_branched_amino_acid_biosynthesis_g__Co
BRANCHED_CHAIN_AA_SYN_PWY__superpathway_of_branched_amino_acid_biosynthesis_g__Co
BRANCHED_CHAIN_AA_SYN_PWY__superpathway_of_branched_amino_acid_biosynthesis_g__Eul
BRANCHED_CHAIN_AA_SYN_PWY__superpathway_of_branched_amino_acid_biosynthesis_g__Fus
BRANCHED_CHAIN_AA_SYN_PWY__superpathway_of_branched_amino_acid_biosynthesis_g__Od
BRANCHED_CHAIN_AA_SYN_PWY__superpathway_of_branched_amino_acid_biosynthesis_g__Od
BRANCHED_CHAIN_AA_SYN_PWY__superpathway_of_branched_amino_acid_biosynthesis_g__Pro
BRANCHED_CHAIN_AA_SYN_PWY__superpathway_of_branched_amino_acid_biosynthesis_g__Ru
BRANCHED_CHAIN_AA_SYN_PWY__superpathway_of_branched_amino_acid_biosynthesis_g__Str
BRANCHED_CHAIN_AA_SYN_PWY__superpathway_of_branched_amino_acid_biosynthesis_g__Str
BRANCHED_CHAIN_AA_SYN_PWY__superpathway_of_branched_amino_acid_biosynthesis_g__Str
BRANCHED_CHAIN_AA_SYN_PWY__superpathway_of_branched_amino_acid_biosynthesis_g__Sul
CALVIN_PWY__Calvin_Benson_Bassham_cycle_g__Anaerostipes_s__Anaerostipes_hadrus
CALVIN_PWY__Calvin_Benson_Bassham_cycle_g__Blautia_s__Ruminococcus_torques
CALVIN_PWY__Calvin_Benson_Bassham_cycle_g__Citrobacter_s__Citrobacter_freundii
CALVIN_PWY__Calvin_Benson_Bassham_cycle_g__Clostridium_s__Clostridium_asparagiforme
CALVIN_PWY__Calvin_Benson_Bassham_cycle_g__Clostridium_s__Clostridium_sp_SS2_1
CALVIN_PWY__Calvin_Benson_Bassham_cycle_g__Coprococcus_s__Coprococcus_catus
CALVIN_PWY__Calvin_Benson_Bassham_cycle_g__Enterobacter_s__Enterobacter_cloacae
CALVIN_PWY__Calvin_Benson_Bassham_cycle_g__Fusobacterium_s__Fusobacterium_nucleatum
CALVIN_PWY__Calvin_Benson_Bassham_cycle_g__Klebsiella_s__Klebsiella_pneumoniae
CALVIN_PWY__Calvin_Benson_Bassham_cycle_g__Lachnospiraceae_noname_s__Lachnospiraceae
CALVIN_PWY__Calvin_Benson_Bassham_cycle_g__Lactobacillus_s__Lactobacillus_rhamnosus
CALVIN_PWY__Calvin_Benson_Bassham_cycle_g__Odoribacter_s__Odoribacter_splanchnicus
CALVIN_PWY__Calvin_Benson_Bassham_cycle_g__Peptostreptococcaceae_noname_s__Clostridiur
CATECHOL_ORTHO_CLEAVAGE_PWY__catechol_degradation_to_&beta__ketoadipate
CENTFERM_PWY__pyruvate_fermentation_to_butanoate_unclassified
COA_PWY_1__coenzyme_A_biosynthesis_II_(mammalian)_g__Actinomyces_s__Actinomyces_grae
COA_PWY_1__coenzyme_A_biosynthesis_II_(mammalian)_g__Adlercreutzia_s__Adlercreutzia_equ
COA_PWY_1__coenzyme_A_biosynthesis_II_(mammalian)_g__Akkermansia_s__Akkermansia_muc
COA_PWY_1__coenzyme_A_biosynthesis_II_(mammalian)_g__Alistipes_s__Alistipes_finegoldii
COA_PWY_1__coenzyme_A_biosynthesis_II_(mammalian)_g__Alistipes_s__Alistipes_indistinctus
COA_PWY_1__coenzyme_A_biosynthesis_II_(mammalian)_g__Alistipes_s__Alistipes_putredinis
COA_PWY_1__coenzyme_A_biosynthesis_II_(mammalian)_g__Anaerofustis_s__Anaerofustis_stero
COA_PWY_1__coenzyme_A_biosynthesis_II_(mammalian)_g__Anaerostipes_s__Anaerostipes_had
COA_PWY_1__coenzyme_A_biosynthesis_II_(mammalian)_g__Anaerotruncus_s__Anaerotruncus_
COA_PWY_1__coenzyme_A_biosynthesis_II_(mammalian)_g__Bacteroides_s__Bacteroides_cellulo
COA_PWY_1__coenzyme_A_biosynthesis_II_(mammalian)_g__Bacteroides_s__Bacteroides_copro

COA_PWY_1__coenzyme_A_biosynthesis_II_(mammalian)_g__Bacteroides_s__Bacteroides_dorei
COA_PWY_1__coenzyme_A_biosynthesis_II_(mammalian)_g__Bacteroides_s__Bacteroides_egger
COA_PWY_1__coenzyme_A_biosynthesis_II_(mammalian)_g__Bacteroides_s__Bacteroides_faecis
COA_PWY_1__coenzyme_A_biosynthesis_II_(mammalian)_g__Bacteroides_s__Bacteroides_finegc
COA_PWY_1__coenzyme_A_biosynthesis_II_(mammalian)_g__Bacteroides_s__Bacteroides_intest
COA_PWY_1__coenzyme_A_biosynthesis_II_(mammalian)_g__Bacteroides_s__Bacteroides_ovatu
COA_PWY_1__coenzyme_A_biosynthesis_II_(mammalian)_g__Bacteroides_s__Bacteroides_salyer
COA_PWY_1__coenzyme_A_biosynthesis_II_(mammalian)_g__Bacteroides_s__Bacteroides_sp_1_
COA_PWY_1__coenzyme_A_biosynthesis_II_(mammalian)_g__Bacteroides_s__Bacteroides_sp_1_
COA_PWY_1__coenzyme_A_biosynthesis_II_(mammalian)_g__Bacteroides_s__Bacteroides_sp_2_
COA_PWY_1__coenzyme_A_biosynthesis_II_(mammalian)_g__Bacteroides_s__Bacteroides_sp_2_
COA_PWY_1__coenzyme_A_biosynthesis_II_(mammalian)_g__Bacteroides_s__Bacteroides_sp_3_
COA_PWY_1__coenzyme_A_biosynthesis_II_(mammalian)_g__Bacteroides_s__Bacteroides_sp_3_
COA_PWY_1__coenzyme_A_biosynthesis_II_(mammalian)_g__Bacteroides_s__Bacteroides_sp_3_
COA_PWY_1__coenzyme_A_biosynthesis_II_(mammalian)_g__Bacteroides_s__Bacteroides_sp_3_
COA_PWY_1__coenzyme_A_biosynthesis_II_(mammalian)_g__Bacteroides_s__Bacteroides_sp_3_
COA_PWY_1__coenzyme_A_biosynthesis_II_(mammalian)_g__Bacteroides_s__Bacteroides_sp_4_
COA_PWY_1__coenzyme_A_biosynthesis_II_(mammalian)_g__Bacteroides_s__Bacteroides_sp_9_
COA_PWY_1__coenzyme_A_biosynthesis_II_(mammalian)_g__Bacteroides_s__Bacteroides_sp_D2
COA_PWY_1__coenzyme_A_biosynthesis_II_(mammalian)_g__Bacteroides_s__Bacteroides_thetai
COA_PWY_1__coenzyme_A_biosynthesis_II_(mammalian)_g__Bacteroides_s__Bacteroides_unifor
COA_PWY_1__coenzyme_A_biosynthesis_II_(mammalian)_g__Bacteroides_s__Bacteroides_xylani
COA_PWY_1__coenzyme_A_biosynthesis_II_(mammalian)_g__Barnesiella_s__Barnesiella_intestin
COA_PWY_1__coenzyme_A_biosynthesis_II_(mammalian)_g__Bifidobacterium_s__Bifidobacteriur
COA_PWY_1__coenzyme_A_biosynthesis_II_(mammalian)_g__Bifidobacterium_s__Bifidobacteriur
COA_PWY_1__coenzyme_A_biosynthesis_II_(mammalian)_g__Bifidobacterium_s__Bifidobacteriur
COA_PWY_1__coenzyme_A_biosynthesis_II_(mammalian)_g__Bifidobacterium_s__Bifidobacteriur
COA_PWY_1__coenzyme_A_biosynthesis_II_(mammalian)_g__Bifidobacterium_s__Bifidobacteriur
COA_PWY_1__coenzyme_A_biosynthesis_II_(mammalian)_g__Blautia_s__Blautia_hydrogenotroph
COA_PWY_1__coenzyme_A_biosynthesis_II_(mammalian)_g__Blautia_s__Blautia_sp_KLE_1732
COA_PWY_1__coenzyme_A_biosynthesis_II_(mammalian)_g__Blautia_s__Ruminococcus_gnavus
COA_PWY_1__coenzyme_A_biosynthesis_II_(mammalian)_g__Blautia_s__Ruminococcus_obeum
COA_PWY_1__coenzyme_A_biosynthesis_II_(mammalian)_g__Butyrivibrio_s__Butyrivibrio_crossc
COA_PWY_1__coenzyme_A_biosynthesis_II_(mammalian)_g__Citrobacter_s__Citrobacter_freundi
COA_PWY_1__coenzyme_A_biosynthesis_II_(mammalian)_g__Clostridiales_noname_s__Clostridia
COA_PWY_1__coenzyme_A_biosynthesis_II_(mammalian)_g__Clostridium_s__Clostridium_boltea
COA_PWY_1__coenzyme_A_biosynthesis_II_(mammalian)_g__Clostridium_s__Clostridium_citroni
COA_PWY_1__coenzyme_A_biosynthesis_II_(mammalian)_g__Clostridium_s__Clostridium_clostri
COA_PWY_1__coenzyme_A_biosynthesis_II_(mammalian)_g__Clostridium_s__Clostridium_hathe
COA_PWY_1__coenzyme_A_biosynthesis_II_(mammalian)_g__Clostridium_s__Clostridium_leptun
COA_PWY_1__coenzyme_A_biosynthesis_II_(mammalian)_g__Clostridium_s__Clostridium_perfrir
COA_PWY_1__coenzyme_A_biosynthesis_II_(mammalian)_g__Clostridium_s__Clostridium_sp_SS2
COA_PWY_1__coenzyme_A_biosynthesis_II_(mammalian)_g__Collinsella_s__Collinsella_intestinal
COA_PWY_1__coenzyme_A_biosynthesis_II_(mammalian)_g__Collinsella_s__Collinsella_tanakaei
COA_PWY_1__coenzyme_A_biosynthesis_II_(mammalian)_g__Coprobacter_s__Coprobacter_fastic
COA_PWY_1__coenzyme_A_biosynthesis_II_(mammalian)_g__Coprococcus_s__Coprococcus_catu
COA_PWY_1__coenzyme_A_biosynthesis_II_(mammalian)_g__Coprococcus_s__Coprococcus_euta

COA_PWY_1__coenzyme_A_biosynthesis_II_(mammalian)_g__Dialister_s__Dialister_invisus
COA_PWY_1__coenzyme_A_biosynthesis_II_(mammalian)_g__Dorea_s__Dorea_formicigenerans
COA_PWY_1__coenzyme_A_biosynthesis_II_(mammalian)_g__Eggerthella_s__Eggerthella_lenta
COA_PWY_1__coenzyme_A_biosynthesis_II_(mammalian)_g__Eggerthella_s__Eggerthella_sp_1_3
COA_PWY_1__coenzyme_A_biosynthesis_II_(mammalian)_g__Eggerthella_s__Eggerthella_sp_HG/
COA_PWY_1__coenzyme_A_biosynthesis_II_(mammalian)_g__Enterococcus_s__Enterococcus_fae
COA_PWY_1__coenzyme_A_biosynthesis_II_(mammalian)_g__Enterococcus_s__Enterococcus_fae
COA_PWY_1__coenzyme_A_biosynthesis_II_(mammalian)_g__Erysipelotrichaceae_noname_s__Cl
COA_PWY_1__coenzyme_A_biosynthesis_II_(mammalian)_g__Erysipelotrichaceae_noname_s__Er
COA_PWY_1__coenzyme_A_biosynthesis_II_(mammalian)_g__Erysipelotrichaceae_noname_s__Er
COA_PWY_1__coenzyme_A_biosynthesis_II_(mammalian)_g__Erysipelotrichaceae_noname_s__Eu
COA_PWY_1__coenzyme_A_biosynthesis_II_(mammalian)_g__Eubacterium_s__Eubacterium_elige
COA_PWY_1__coenzyme_A_biosynthesis_II_(mammalian)_g__Eubacterium_s__Eubacterium_halli
COA_PWY_1__coenzyme_A_biosynthesis_II_(mammalian)_g__Eubacterium_s__Eubacterium_sirae
COA_PWY_1__coenzyme_A_biosynthesis_II_(mammalian)_g__Eubacterium_s__Eubacterium_sp_3
COA_PWY_1__coenzyme_A_biosynthesis_II_(mammalian)_g__Eubacterium_s__Eubacterium_vent
COA_PWY_1__coenzyme_A_biosynthesis_II_(mammalian)_g__Fusobacterium_s__Fusobacterium_
COA_PWY_1__coenzyme_A_biosynthesis_II_(mammalian)_g__Gemella_s__Gemella_sanguinis
COA_PWY_1__coenzyme_A_biosynthesis_II_(mammalian)_g__Gordonibacter_s__Gordonibacter_p
COA_PWY_1__coenzyme_A_biosynthesis_II_(mammalian)_g__Holdemania_s__Holdemania_filifor
COA_PWY_1__coenzyme_A_biosynthesis_II_(mammalian)_g__Klebsiella_s__Klebsiella_pneumoni
COA_PWY_1__coenzyme_A_biosynthesis_II_(mammalian)_g__Lachnospiraceae_noname_s__Lach
COA_PWY_1__coenzyme_A_biosynthesis_II_(mammalian)_g__Lachnospiraceae_noname_s__Lach
COA_PWY_1__coenzyme_A_biosynthesis_II_(mammalian)_g__Lachnospiraceae_noname_s__Lach
COA_PWY_1__coenzyme_A_biosynthesis_II_(mammalian)_g__Lachnospiraceae_noname_s__Lach
COA_PWY_1__coenzyme_A_biosynthesis_II_(mammalian)_g__Lachnospiraceae_noname_s__Lach
COA_PWY_1__coenzyme_A_biosynthesis_II_(mammalian)_g__Lachnospiraceae_noname_s__Lach
COA_PWY_1__coenzyme_A_biosynthesis_II_(mammalian)_g__Lachnospiraceae_noname_s__Lach
COA_PWY_1__coenzyme_A_biosynthesis_II_(mammalian)_g__Lachnospiraceae_noname_s__Lach
COA_PWY_1__coenzyme_A_biosynthesis_II_(mammalian)_g__Lactobacillus_s__Lactobacillus_case
COA_PWY_1__coenzyme_A_biosynthesis_II_(mammalian)_g__Lactobacillus_s__Lactobacillus_ferm
COA_PWY_1__coenzyme_A_biosynthesis_II_(mammalian)_g__Odoribacter_s__Odoribacter_laneu
COA_PWY_1__coenzyme_A_biosynthesis_II_(mammalian)_g__Odoribacter_s__Odoribacter_splan
COA_PWY_1__coenzyme_A_biosynthesis_II_(mammalian)_g__Oxalobacter_s__Oxalobacter_formi
COA_PWY_1__coenzyme_A_biosynthesis_II_(mammalian)_g__Parabacteroides_s__Parabacteroide
COA_PWY_1__coenzyme_A_biosynthesis_II_(mammalian)_g__Parabacteroides_s__Parabacteroide
COA_PWY_1__coenzyme_A_biosynthesis_II_(mammalian)_g__Parabacteroides_s__Parabacteroide
COA_PWY_1__coenzyme_A_biosynthesis_II_(mammalian)_g__Parabacteroides_s__Parabacteroide
COA_PWY_1__coenzyme_A_biosynthesis_II_(mammalian)_g__Parabacteroides_s__Parabacteroide
COA_PWY_1__coenzyme_A_biosynthesis_II_(mammalian)_g__Parabacteroides_s__Parabacteroide
COA_PWY_1__coenzyme_A_biosynthesis_II_(mammalian)_g__Paraprevotella_s__Paraprevotella_
COA_PWY_1__coenzyme_A_biosynthesis_II_(mammalian)_g__Parasutterella_s__Parasutterella_e
COA_PWY_1__coenzyme_A_biosynthesis_II_(mammalian)_g__Pediococcus_s__Pediococcus_acidil
COA_PWY_1__coenzyme_A_biosynthesis_II_(mammalian)_g__Pediococcus_s__Pediococcus_lolii
COA_PWY_1__coenzyme_A_biosynthesis_II_(mammalian)_g__Peptostreptococcaceae_noname_s_
COA_PWY_1__coenzyme_A_biosynthesis_II_(mammalian)_g__Phascolarctobacterium_s__Phascol
COA_PWY_1__coenzyme_A_biosynthesis_II_(mammalian)_g__Roseburia_s__Roseburia_hominis

COA_PWY_1__coenzyme_A_biosynthesis_II_(mammalian)_g__Roseburia_s__Roseburia_inulinivor
COA_PWY_1__coenzyme_A_biosynthesis_II_(mammalian)_g__Rothia_s__Rothia_mucilaginosa
COA_PWY_1__coenzyme_A_biosynthesis_II_(mammalian)_g__Ruminococcus_s__Ruminococcus_c
COA_PWY_1__coenzyme_A_biosynthesis_II_(mammalian)_g__Ruminococcus_s__Ruminococcus_l
COA_PWY_1__coenzyme_A_biosynthesis_II_(mammalian)_g__Ruminococcus_s__Ruminococcus_s
COA_PWY_1__coenzyme_A_biosynthesis_II_(mammalian)_g__Slackia_s__Slackia_piriformis
COA_PWY_1__coenzyme_A_biosynthesis_II_(mammalian)_g__Streptococcus_s__Streptococcus_au
COA_PWY_1__coenzyme_A_biosynthesis_II_(mammalian)_g__Streptococcus_s__Streptococcus_m
COA_PWY_1__coenzyme_A_biosynthesis_II_(mammalian)_g__Subdoligranulum_s__Subdoligranul
COA_PWY_1__coenzyme_A_biosynthesis_II_(mammalian)_g__Veillonella_s__Veillonella_dispar
COA_PWY_1__coenzyme_A_biosynthesis_II_(mammalian)_g__Veillonella_s__Veillonella_parvula
COA_PWY__coenzyme_A_biosynthesis_I_g__Akkermansia_s__Akkermansia_muciniphila
COA_PWY__coenzyme_A_biosynthesis_I_g__Anaerofustis_s__Anaerofustis_stercorihominis
COA_PWY__coenzyme_A_biosynthesis_I_g__Anaerostipes_s__Anaerostipes_hadrus
COA_PWY__coenzyme_A_biosynthesis_I_g__Anaerotruncus_s__Anaerotruncus_colihominis
COA_PWY__coenzyme_A_biosynthesis_I_g__Bacteroides_s__Bacteroides_ovatus
COA_PWY__coenzyme_A_biosynthesis_I_g__Bacteroides_s__Bacteroides_sp_1_1_30
COA_PWY__coenzyme_A_biosynthesis_I_g__Bacteroides_s__Bacteroides_sp_2_1_22
COA_PWY__coenzyme_A_biosynthesis_I_g__Bacteroides_s__Bacteroides_sp_2_2_4
COA_PWY__coenzyme_A_biosynthesis_I_g__Bacteroides_s__Bacteroides_sp_3_1_23
COA_PWY__coenzyme_A_biosynthesis_I_g__Bacteroides_s__Bacteroides_xylanisolvens
COA_PWY__coenzyme_A_biosynthesis_I_g__Bifidobacterium_s__Bifidobacterium_dentium
COA_PWY__coenzyme_A_biosynthesis_I_g__Citrobacter_s__Citrobacter_freundii
COA_PWY__coenzyme_A_biosynthesis_I_g__Clostridiales_noname_s__Clostridiales_bacterium_1_
COA_PWY__coenzyme_A_biosynthesis_I_g__Clostridium_s__Clostridium_sp_SS2_1
COA_PWY__coenzyme_A_biosynthesis_I_g__Coprococcus_s__Coprococcus_eutactus
COA_PWY__coenzyme_A_biosynthesis_I_g__Eggerthella_s__Eggerthella_lenta
COA_PWY__coenzyme_A_biosynthesis_I_g__Eggerthella_s__Eggerthella_sp_1_3_56FAA
COA_PWY__coenzyme_A_biosynthesis_I_g__Eggerthella_s__Eggerthella_sp_HGA1
COA_PWY__coenzyme_A_biosynthesis_I_g__Enterococcus_s__Enterococcus_faecium
COA_PWY__coenzyme_A_biosynthesis_I_g__Erysipelotrichaceae_noname_s__Clostridium_spirofo
COA_PWY__coenzyme_A_biosynthesis_I_g__Eubacterium_s__Eubacterium_ventriosum
COA_PWY__coenzyme_A_biosynthesis_I_g__Lachnospiraceae_noname_s__Lachnospiraceae_bact
COA_PWY__coenzyme_A_biosynthesis_I_g__Odoribacter_s__Odoribacter_splanchnicus
COA_PWY__coenzyme_A_biosynthesis_I_g__Oxalobacter_s__Oxalobacter_formigenes
COA_PWY__coenzyme_A_biosynthesis_I_g__Pediococcus_s__Pediococcus_acidilactici
COA_PWY__coenzyme_A_biosynthesis_I_g__Pediococcus_s__Pediococcus_lolii
COA_PWY__coenzyme_A_biosynthesis_I_g__Phascolarctobacterium_s__Phascolarctobacterium_su
COA_PWY__coenzyme_A_biosynthesis_I_g__Streptococcus_s__Streptococcus_australis
COA_PWY__coenzyme_A_biosynthesis_I_g__Streptococcus_s__Streptococcus_mitis_oralis_pneum
COA_PWY__coenzyme_A_biosynthesis_I_g__Veillonella_s__Veillonella_dispar
COA_PWY__coenzyme_A_biosynthesis_I_g__Veillonella_s__Veillonella_parvula
COBALSYN_PWY__adenosylcobalamin_salvage_from_cobinamide_I_g__Blautia_s__Blautia_sp_KLE
COBALSYN_PWY__adenosylcobalamin_salvage_from_cobinamide_I_g__Blautia_s__Ruminococcus_
COBALSYN_PWY__adenosylcobalamin_salvage_from_cobinamide_I_g__Citrobacter_s__Citrobacte
COBALSYN_PWY__adenosylcobalamin_salvage_from_cobinamide_I_g__Coprococcus_s__Coprococ
COBALSYN_PWY__adenosylcobalamin_salvage_from_cobinamide_I_g__Phascolarctobacterium_s__

COBALSYN_PWY__adenosylcobalamin_salvage_from_cobinamide_I_g__Roseburia_s__Roseburia_i
COBALSYN_PWY__adenosylcobalamin_salvage_from_cobinamide_I_g__Ruminococcus_s__Rumino
COMPLETE_ARO_PWY__superpathway_of_aromatic_amino_acid_biosynthesis_g__Akkermansia_s
COMPLETE_ARO_PWY__superpathway_of_aromatic_amino_acid_biosynthesis_g__Anaerostipes_s
COMPLETE_ARO_PWY__superpathway_of_aromatic_amino_acid_biosynthesis_g__Bacteroides_s_
COMPLETE_ARO_PWY__superpathway_of_aromatic_amino_acid_biosynthesis_g__Blautia_s__Blau
COMPLETE_ARO_PWY__superpathway_of_aromatic_amino_acid_biosynthesis_g__Blautia_s__Rum
COMPLETE_ARO_PWY__superpathway_of_aromatic_amino_acid_biosynthesis_g__Blautia_s__Rum
COMPLETE_ARO_PWY__superpathway_of_aromatic_amino_acid_biosynthesis_g__Butyrivibrio_s_
COMPLETE_ARO_PWY__superpathway_of_aromatic_amino_acid_biosynthesis_g__Citrobacter_s_
COMPLETE_ARO_PWY__superpathway_of_aromatic_amino_acid_biosynthesis_g__Clostridiales_no
COMPLETE_ARO_PWY__superpathway_of_aromatic_amino_acid_biosynthesis_g__Clostridium_s_
COMPLETE_ARO_PWY__superpathway_of_aromatic_amino_acid_biosynthesis_g__Clostridium_s_
COMPLETE_ARO_PWY__superpathway_of_aromatic_amino_acid_biosynthesis_g__Clostridium_s_
COMPLETE_ARO_PWY__superpathway_of_aromatic_amino_acid_biosynthesis_g__Clostridium_s_
COMPLETE_ARO_PWY__superpathway_of_aromatic_amino_acid_biosynthesis_g__Clostridium_s_
COMPLETE_ARO_PWY__superpathway_of_aromatic_amino_acid_biosynthesis_g__Clostridium_s_
COMPLETE_ARO_PWY__superpathway_of_aromatic_amino_acid_biosynthesis_g__Clostridium_s_
COMPLETE_ARO_PWY__superpathway_of_aromatic_amino_acid_biosynthesis_g__Eubacterium_s_
COMPLETE_ARO_PWY__superpathway_of_aromatic_amino_acid_biosynthesis_g__Klebsiella_s__K
COMPLETE_ARO_PWY__superpathway_of_aromatic_amino_acid_biosynthesis_g__Lachnospiracea
COMPLETE_ARO_PWY__superpathway_of_aromatic_amino_acid_biosynthesis_g__Lachnospiracea
COMPLETE_ARO_PWY__superpathway_of_aromatic_amino_acid_biosynthesis_g__Lachnospiracea
COMPLETE_ARO_PWY__superpathway_of_aromatic_amino_acid_biosynthesis_g__Lachnospiracea
COMPLETE_ARO_PWY__superpathway_of_aromatic_amino_acid_biosynthesis_g__Lactococcus_s_
COMPLETE_ARO_PWY__superpathway_of_aromatic_amino_acid_biosynthesis_g__Phascolarctoba
COMPLETE_ARO_PWY__superpathway_of_aromatic_amino_acid_biosynthesis_g__Propionibacteri
COMPLETE_ARO_PWY__superpathway_of_aromatic_amino_acid_biosynthesis_g__Roseburia_s__R
COMPLETE_ARO_PWY__superpathway_of_aromatic_amino_acid_biosynthesis_g__Ruminococcus_
DAPLYSINESYN_PWY__L_lysine_biosynthesis_I_g__Bifidobacterium_s__Bifidobacterium_animalis
DAPLYSINESYN_PWY__L_lysine_biosynthesis_I_g__Citrobacter_s__Citrobacter_freundii
DAPLYSINESYN_PWY__L_lysine_biosynthesis_I_g__Enterobacter_s__Enterobacter_cloacae
DAPLYSINESYN_PWY__L_lysine_biosynthesis_I_g__Klebsiella_s__Klebsiella_pneumoniae
DAPLYSINESYN_PWY__L_lysine_biosynthesis_I_g__Oxalobacter_s__Oxalobacter_formigenes
DTDPRHAMSYN_PWY__dTDP_L_rhamnose_biosynthesis_I_g__Alistipes_s__Alistipes_shahii
DTDPRHAMSYN_PWY__dTDP_L_rhamnose_biosynthesis_I_g__Anaerostipes_s__Anaerostipes_had
DTDPRHAMSYN_PWY__dTDP_L_rhamnose_biosynthesis_I_g__Bacteroides_s__Bacteroides_finego
DTDPRHAMSYN_PWY__dTDP_L_rhamnose_biosynthesis_I_g__Bacteroides_s__Bacteroides_fragilis
DTDPRHAMSYN_PWY__dTDP_L_rhamnose_biosynthesis_I_g__Bacteroides_s__Bacteroides_ovatus
DTDPRHAMSYN_PWY__dTDP_L_rhamnose_biosynthesis_I_g__Bacteroides_s__Bacteroides_sp_1_
DTDPRHAMSYN_PWY__dTDP_L_rhamnose_biosynthesis_I_g__Bacteroides_s__Bacteroides_sp_2_
DTDPRHAMSYN_PWY__dTDP_L_rhamnose_biosynthesis_I_g__Bacteroides_s__Bacteroides_sp_2_
DTDPRHAMSYN_PWY__dTDP_L_rhamnose_biosynthesis_I_g__Bacteroides_s__Bacteroides_sp_3_
DTDPRHAMSYN_PWY__dTDP_L_rhamnose_biosynthesis_I_g__Bacteroides_s__Bacteroides_xylanis
DTDPRHAMSYN_PWY__dTDP_L_rhamnose_biosynthesis_I_g__Bifidobacterium_s__Bifidobacteriur
DTDPRHAMSYN_PWY__dTDP_L_rhamnose_biosynthesis_I_g__Blautia_s__Blautia_sp_KLE_1732
DTDPRHAMSYN_PWY__dTDP_L_rhamnose_biosynthesis_I_g__Blautia_s__Ruminococcus_obeum

DTDPRHAMSYN_PWY__dTDP_L_rhamnose_biosynthesis_I_g__Blautia_s__Ruminococcus_torques
DTDPRHAMSYN_PWY__dTDP_L_rhamnose_biosynthesis_I_g__Clostridium_s__Clostridium_aspara
DTDPRHAMSYN_PWY__dTDP_L_rhamnose_biosynthesis_I_g__Clostridium_s__Clostridium_clostric
DTDPRHAMSYN_PWY__dTDP_L_rhamnose_biosynthesis_I_g__Clostridium_s__Clostridium_sp_L2_
DTDPRHAMSYN_PWY__dTDP_L_rhamnose_biosynthesis_I_g__Clostridium_s__Clostridium_sp_SS2
DTDPRHAMSYN_PWY__dTDP_L_rhamnose_biosynthesis_I_g__Coprococcus_s__Coprococcus_catus
DTDPRHAMSYN_PWY__dTDP_L_rhamnose_biosynthesis_I_g__Eggerthella_s__Eggerthella_lenta
DTDPRHAMSYN_PWY__dTDP_L_rhamnose_biosynthesis_I_g__Eggerthella_s__Eggerthella_sp_1_3
DTDPRHAMSYN_PWY__dTDP_L_rhamnose_biosynthesis_I_g__Eggerthella_s__Eggerthella_sp_HGA
DTDPRHAMSYN_PWY__dTDP_L_rhamnose_biosynthesis_I_g__Enterococcus_s__Enterococcus_faec
DTDPRHAMSYN_PWY__dTDP_L_rhamnose_biosynthesis_I_g__Erysipelotrichaceae_noname_s__Eu
DTDPRHAMSYN_PWY__dTDP_L_rhamnose_biosynthesis_I_g__Fusobacterium_s__Fusobacterium_
DTDPRHAMSYN_PWY__dTDP_L_rhamnose_biosynthesis_I_g__Lachnospiraceae_noname_s__Lachn
DTDPRHAMSYN_PWY__dTDP_L_rhamnose_biosynthesis_I_g__Odoribacter_s__Odoribacter_splanc
DTDPRHAMSYN_PWY__dTDP_L_rhamnose_biosynthesis_I_g__Oxalobacter_s__Oxalobacter_formi
DTDPRHAMSYN_PWY__dTDP_L_rhamnose_biosynthesis_I_g__Propionibacterium_s__Propionibact
DTDPRHAMSYN_PWY__dTDP_L_rhamnose_biosynthesis_I_g__Roseburia_s__Roseburia_intestinali
DTDPRHAMSYN_PWY__dTDP_L_rhamnose_biosynthesis_I_g__Ruminococcus_s__Ruminococcus_c
DTDPRHAMSYN_PWY__dTDP_L_rhamnose_biosynthesis_I_g__Ruminococcus_s__Ruminococcus_s
DTDPRHAMSYN_PWY__dTDP_L_rhamnose_biosynthesis_I_g__Streptococcus_s__Streptococcus_au
ECASYN_PWY__enterobacterial_common_antigen_biosynthesis_g__Citrobacter_s__Citrobacter_fr
ECASYN_PWY__enterobacterial_common_antigen_biosynthesis_unclassified
ENTBACSYN_PWY__enterobactin_biosynthesis_unclassified
FAO_PWY__fatty_acid_&beta__oxidation_I_g__Citrobacter_s__Citrobacter_freundii
FASYN_ELONG_PWY__fatty_acid_elongation____saturated_g__Bacteroides_s__Bacteroides_sp_2_
FASYN_ELONG_PWY__fatty_acid_elongation____saturated_g__Bacteroides_s__Bacteroides_xylan
FASYN_ELONG_PWY__fatty_acid_elongation____saturated_g__Citrobacter_s__Citrobacter_freunc
FASYN_ELONG_PWY__fatty_acid_elongation____saturated_g__Enterobacter_s__Enterobacter_clo
FASYN_ELONG_PWY__fatty_acid_elongation____saturated_g__Haemophilus_s__Haemophilus_pa
FASYN_ELONG_PWY__fatty_acid_elongation____saturated_g__Klebsiella_s__Klebsiella_pneumon
FASYN_INITIAL_PWY__superpathway_of_fatty_acid_biosynthesis_initiation_(E__coli)_g__Bacteroi
FASYN_INITIAL_PWY__superpathway_of_fatty_acid_biosynthesis_initiation_(E__coli)_g__Bacteroi
FASYN_INITIAL_PWY__superpathway_of_fatty_acid_biosynthesis_initiation_(E__coli)_g__Citrobact
FASYN_INITIAL_PWY__superpathway_of_fatty_acid_biosynthesis_initiation_(E__coli)_g__Klebsiell
FASYN_INITIAL_PWY__superpathway_of_fatty_acid_biosynthesis_initiation_(E__coli)_g__Streptoc
FERMENTATION_PWY__mixed_acid_fermentation_g__Citrobacter_s__Citrobacter_freundii
FOLSYN_PWY__superpathway_of_tetrahydrofolate_biosynthesis_and_salvage_g__Lactococcus_s__
FUC_RHAMCAT_PWY__superpathway_of_fucose_and_rhamnose_degradation_g__Citrobacter_s__
FUCCAT_PWY__fucose_degradation_g__Citrobacter_s__Citrobacter_freundii
FUCCAT_PWY__fucose_degradation_g__Klebsiella_s__Klebsiella_pneumoniae
GALACTARDEG_PWY__D_galactarate_degradation_I_g__Citrobacter_s__Citrobacter_freundii
GALACTARDEG_PWY__D_galactarate_degradation_I_g__Enterobacter_s__Enterobacter_cloacae
GALACTARDEG_PWY__D_galactarate_degradation_I_g__Klebsiella_s__Klebsiella_pneumoniae
GALACTUROCAT_PWY__D_galacturonate_degradation_I_g__Alistipes_s__Alistipes_shahii
GALACTUROCAT_PWY__D_galacturonate_degradation_I_g__Citrobacter_s__Citrobacter_freundii
GLCMANNANAUT_PWY__superpathway_of_N_acetylglucosamine,_N_acetylmannosamine_and_N
GLCMANNANAUT_PWY__superpathway_of_N_acetylglucosamine,_N_acetylmannosamine_and_N

GLCMANNANAUT_PWY__superpathway_of_N_acetylglucosamine,_N_acetylmannosamine_and_N
GLUCARDEG_PWY__D_glucarate_degradation_I_g__Citrobacter_s__Citrobacter_freundii
GLUCARDEG_PWY__D_glucarate_degradation_I_g__Enterobacter_s__Enterobacter_cloacae
GLUCARGALACTSUPER_PWY__superpathway_of_D_glucarate_and_D_galactarate_degradation_g_
GLUCARGALACTSUPER_PWY__superpathway_of_D_glucarate_and_D_galactarate_degradation_g_
GLUCARGALACTSUPER_PWY__superpathway_of_D_glucarate_and_D_galactarate_degradation_g_
GLUCONEO_PWY__gluconeogenesis_I_g__Citrobacter_s__Citrobacter_freundii
GLUCONEO_PWY__gluconeogenesis_I_g__Enterobacter_s__Enterobacter_cloacae
GLUCOSE1PMETAB_PWY__glucose_and_glucose_1_phosphate_degradation_g__Klebsiella_s__Kle
GLUCOSE1PMETAB_PWY__glucose_and_glucose_1_phosphate_degradation_unclassified
GLUDEG_I_PWY__GABA_shunt_unclassified
GLUTORN_PWY__L_ornithine_biosynthesis_g__Burkholderiales_noname_s__Burkholderiales_bact
GLUTORN_PWY__L_ornithine_biosynthesis_g__Citrobacter_s__Citrobacter_freundii
GLUTORN_PWY__L_ornithine_biosynthesis_g__Coprococcus_s__Coprococcus_catus
GLUTORN_PWY__L_ornithine_biosynthesis_g__Eubacterium_s__Eubacterium_siraeum
GLUTORN_PWY__L_ornithine_biosynthesis_g__Klebsiella_s__Klebsiella_pneumoniae
GLUTORN_PWY__L_ornithine_biosynthesis_g__Oxalobacter_s__Oxalobacter_formigenes
GLUTORN_PWY__L_ornithine_biosynthesis_g__Parasutterella_s__Parasutterella_excrementihomi
GLUTORN_PWY__L_ornithine_biosynthesis_g__Ruminococcus_s__Ruminococcus_champanellensis
GLUTORN_PWY__L_ornithine_biosynthesis_g__Veillonella_s__Veillonella_dispar
GLUTORN_PWY__L_ornithine_biosynthesis_g__Veillonella_s__Veillonella_parvula
GLYCOCAT_PWY__glycogen_degradation_I_(bacterial)_unclassified
GLYCOGENSYNTH_PWY__glycogen_biosynthesis_I_(from_ADP_D_Glucose)_g__Anaerostipes_s__A
GLYCOGENSYNTH_PWY__glycogen_biosynthesis_I_(from_ADP_D_Glucose)_g__Blautia_s__Blautia_
GLYCOGENSYNTH_PWY__glycogen_biosynthesis_I_(from_ADP_D_Glucose)_g__Blautia_s__Rumin
GLYCOGENSYNTH_PWY__glycogen_biosynthesis_I_(from_ADP_D_Glucose)_g__Citrobacter_s__Cit
GLYCOGENSYNTH_PWY__glycogen_biosynthesis_I_(from_ADP_D_Glucose)_g__Clostridium_s__Cl
GLYCOGENSYNTH_PWY__glycogen_biosynthesis_I_(from_ADP_D_Glucose)_g__Coprococcus_s__C
GLYCOGENSYNTH_PWY__glycogen_biosynthesis_I_(from_ADP_D_Glucose)_g__Coprococcus_s__C
GLYCOGENSYNTH_PWY__glycogen_biosynthesis_I_(from_ADP_D_Glucose)_g__Coprococcus_s__C
GLYCOGENSYNTH_PWY__glycogen_biosynthesis_I_(from_ADP_D_Glucose)_g__Enterobacter_s__E
GLYCOGENSYNTH_PWY__glycogen_biosynthesis_I_(from_ADP_D_Glucose)_g__Erysipelotrichaceae
GLYCOGENSYNTH_PWY__glycogen_biosynthesis_I_(from_ADP_D_Glucose)_g__Eubacterium_s__E
GLYCOGENSYNTH_PWY__glycogen_biosynthesis_I_(from_ADP_D_Glucose)_g__Fusobacterium_s__
GLYCOGENSYNTH_PWY__glycogen_biosynthesis_I_(from_ADP_D_Glucose)_g__Klebsiella_s__Kleb
GLYCOGENSYNTH_PWY__glycogen_biosynthesis_I_(from_ADP_D_Glucose)_g__Lachnospiraceae_r
GLYCOGENSYNTH_PWY__glycogen_biosynthesis_I_(from_ADP_D_Glucose)_g__Lactobacillus_s__L
GLYCOGENSYNTH_PWY__glycogen_biosynthesis_I_(from_ADP_D_Glucose)_g__Ruminococcus_s__
GLYCOL_GLYOXDEG_PWY__superpathway_of_glycol_metabolism_and_degradation_unclassified
GLYCOLYSIS_E_D__superpathway_of_glycolysis_and_Entner_Doudoroff_g__Citrobacter_s__Citrob
GLYCOLYSIS_TCA_GLYOX_BYPASS__superpathway_of_glycolysis,_pyruvate_dehydrogenase,_TCA,_
GLYCOLYSIS__glycolysis_I_(from_glucose_6_phosphate)_g__Citrobacter_s__Citrobacter_freundii
GLYCOLYSIS__glycolysis_I_(from_glucose_6_phosphate)_g__Lactococcus_s__Lactococcus_lactis
GLYOXYLATE_BYPASS__glyoxylate_cycle_g__Citrobacter_s__Citrobacter_freundii
GLYOXYLATE_BYPASS__glyoxylate_cycle_g__Enterobacter_s__Enterobacter_cloacae
GLYOXYLATE_BYPASS__glyoxylate_cycle_g__Klebsiella_s__Klebsiella_pneumoniae
GLYOXYLATE_BYPASS__glyoxylate_cycle_g__Nakaseomyces_s__Candida_glabrata

GOLPDLCAT_PWY__superpathway_of_glycerol_degradation_to_1,3_propanediol_g__Citrobacter_s
GOLPDLCAT_PWY__superpathway_of_glycerol_degradation_to_1,3_propanediol_g__Escherichia_s
GOLPDLCAT_PWY__superpathway_of_glycerol_degradation_to_1,3_propanediol_g__Klebsiella_s_
GOLPDLCAT_PWY__superpathway_of_glycerol_degradation_to_1,3_propanediol_g__Streptococcu
HCAMHPDEG_PWY__3_phenylpropanoate_and_3_(3_hydroxyphenyl)propanoate_degradation_to_
HCAMHPDEG_PWY__3_phenylpropanoate_and_3_(3_hydroxyphenyl)propanoate_degradation_to_
HEME_BIOSYNTHESIS_II__heme_biosynthesis_I_(aerobic)_g__Citrobacter_s__Citrobacter_freundii
HEME_BIOSYNTHESIS_II__heme_biosynthesis_I_(aerobic)_g__Enterobacter_s__Enterobacter_cloa
HEME_BIOSYNTHESIS_II__heme_biosynthesis_I_(aerobic)_g__Propionibacterium_s__Propionibact
HEME_BIOSYNTHESIS_II__heme_biosynthesis_I_(aerobic)_g__Veillonella_s__Veillonella_dispar
HEME_BIOSYNTHESIS_II__heme_biosynthesis_I_(aerobic)_unclassified
HEMESYN2_PWY__heme_biosynthesis_II_(anaerobic)_g__Enterobacter_s__Enterobacter_cloacae
HEXITOLDEGSUPER_PWY__superpathway_of_hexitol_degradation_(bacteria)_g__Citrobacter_s__C
HISDEG_PWY__L_histidine_degradation_I_g__Alistipes_s__Alistipes_shahii
HISDEG_PWY__L_histidine_degradation_I_g__Citrobacter_s__Citrobacter_freundii
HISDEG_PWY__L_histidine_degradation_I_g__Clostridium_s__Clostridium_symbiosum
HISDEG_PWY__L_histidine_degradation_I_g__Enterobacter_s__Enterobacter_cloacae
HISDEG_PWY__L_histidine_degradation_I_g__Fusobacterium_s__Fusobacterium_nucleatum
HISDEG_PWY__L_histidine_degradation_I_g__Klebsiella_s__Klebsiella_pneumoniae
HISDEG_PWY__L_histidine_degradation_I_g__Streptococcus_s__Streptococcus_australis
HISDEG_PWY__L_histidine_degradation_I_g__Streptococcus_s__Streptococcus_sanguinis
HISTSYN_PWY__L_histidine_biosynthesis_g__Anaerostipes_s__Anaerostipes_hadrus
HISTSYN_PWY__L_histidine_biosynthesis_g__Bacteroides_s__Bacteroides_faecis
HISTSYN_PWY__L_histidine_biosynthesis_g__Bacteroides_s__Bacteroides_sp_1_1_6
HISTSYN_PWY__L_histidine_biosynthesis_g__Bacteroides_s__Bacteroides_thetaiotaomicron
HISTSYN_PWY__L_histidine_biosynthesis_g__Bifidobacterium_s__Bifidobacterium_bifidum
HISTSYN_PWY__L_histidine_biosynthesis_g__Bifidobacterium_s__Bifidobacterium_dentium
HISTSYN_PWY__L_histidine_biosynthesis_g__Bifidobacterium_s__Bifidobacterium_pseudocatenul
HISTSYN_PWY__L_histidine_biosynthesis_g__Burkholderiales_noname_s__Burkholderiales_bacter
HISTSYN_PWY__L_histidine_biosynthesis_g__Citrobacter_s__Citrobacter_freundii
HISTSYN_PWY__L_histidine_biosynthesis_g__Clostridium_s__Clostridium_sp_SS2_1
HISTSYN_PWY__L_histidine_biosynthesis_g__Collinsella_s__Collinsella_tanakaei
HISTSYN_PWY__L_histidine_biosynthesis_g__Eubacterium_s__Eubacterium_siraeum
HISTSYN_PWY__L_histidine_biosynthesis_g__Klebsiella_s__Klebsiella_pneumoniae
HISTSYN_PWY__L_histidine_biosynthesis_g__Lachnospiraceae_noname_s__Lachnospiraceae_bact
HISTSYN_PWY__L_histidine_biosynthesis_g__Lachnospiraceae_noname_s__Lachnospiraceae_bact
HISTSYN_PWY__L_histidine_biosynthesis_g__Lactobacillus_s__Lactobacillus_casei_paracasei
HISTSYN_PWY__L_histidine_biosynthesis_g__Lactobacillus_s__Lactobacillus_fermentum
HISTSYN_PWY__L_histidine_biosynthesis_g__Lactococcus_s__Lactococcus_lactis
HISTSYN_PWY__L_histidine_biosynthesis_g__Parasutterella_s__Parasutterella_excrementihominis
HISTSYN_PWY__L_histidine_biosynthesis_g__Ruminococcaceae_noname_s__Ruminococcaceae_ba
HISTSYN_PWY__L_histidine_biosynthesis_g__Ruminococcus_s__Ruminococcus_champanellensis
HISTSYN_PWY__L_histidine_biosynthesis_g__Veillonella_s__Veillonella_dispar
HISTSYN_PWY__L_histidine_biosynthesis_g__Veillonella_s__Veillonella_parvula
HOMOSER_METSYN_PWY__L_methionine_biosynthesis_I_g__Anaerostipes_s__Anaerostipes_hadr
HOMOSER_METSYN_PWY__L_methionine_biosynthesis_I_g__Clostridium_s__Clostridium_sp_SS2_
HOMOSER_METSYN_PWY__L_methionine_biosynthesis_I_g__Coprococcus_s__Coprococcus_catus

HOMOSER_METSYN_PWY__L_methionine_biosynthesis_I_g__Enterobacter_s__Enterobacter_cloac
HOMOSER_METSYN_PWY__L_methionine_biosynthesis_I_g__Klebsiella_s__Klebsiella_pneumonia
HOMOSER_METSYN_PWY__L_methionine_biosynthesis_I_g__Lachnospiraceae_noname_s__Lachn
HOMOSER_METSYN_PWY__L_methionine_biosynthesis_I_g__Streptococcus_s__Streptococcus_mi
HOMOSER_METSYN_PWY__L_methionine_biosynthesis_I_g__Streptococcus_s__Streptococcus_sa
ILEUSYN_PWY__L_isoleucine_biosynthesis_I_(from_threonine)_g__Akkermansia_s__Akkermansia_
ILEUSYN_PWY__L_isoleucine_biosynthesis_I_(from_threonine)_g__Anaerofustis_s__Anaerofustis_
ILEUSYN_PWY__L_isoleucine_biosynthesis_I_(from_threonine)_g__Blautia_s__Blautia_sp_KLE_173
ILEUSYN_PWY__L_isoleucine_biosynthesis_I_(from_threonine)_g__Blautia_s__Ruminococcus_obe
ILEUSYN_PWY__L_isoleucine_biosynthesis_I_(from_threonine)_g__Clostridium_s__Clostridium_as
ILEUSYN_PWY__L_isoleucine_biosynthesis_I_(from_threonine)_g__Clostridium_s__Clostridium_le
ILEUSYN_PWY__L_isoleucine_biosynthesis_I_(from_threonine)_g__Coprococcus_s__Coprococcus_
ILEUSYN_PWY__L_isoleucine_biosynthesis_I_(from_threonine)_g__Coprococcus_s__Coprococcus_
ILEUSYN_PWY__L_isoleucine_biosynthesis_I_(from_threonine)_g__Coprococcus_s__Coprococcus_
ILEUSYN_PWY__L_isoleucine_biosynthesis_I_(from_threonine)_g__Eubacterium_s__Eubacterium_
ILEUSYN_PWY__L_isoleucine_biosynthesis_I_(from_threonine)_g__Fusobacterium_s__Fusobacteri
ILEUSYN_PWY__L_isoleucine_biosynthesis_I_(from_threonine)_g__Odoribacter_s__Odoribacter_la
ILEUSYN_PWY__L_isoleucine_biosynthesis_I_(from_threonine)_g__Odoribacter_s__Odoribacter_s
ILEUSYN_PWY__L_isoleucine_biosynthesis_I_(from_threonine)_g__Propionibacterium_s__Propion
ILEUSYN_PWY__L_isoleucine_biosynthesis_I_(from_threonine)_g__Ruminococcus_s__Ruminococc
ILEUSYN_PWY__L_isoleucine_biosynthesis_I_(from_threonine)_g__Streptococcus_s__Streptococcu
ILEUSYN_PWY__L_isoleucine_biosynthesis_I_(from_threonine)_g__Streptococcus_s__Streptococcu
ILEUSYN_PWY__L_isoleucine_biosynthesis_I_(from_threonine)_g__Streptococcus_s__Streptococcu
ILEUSYN_PWY__L_isoleucine_biosynthesis_I_(from_threonine)_g__Subdoligranulum_s__Subdoligr
KETOGLUCONMET_PWY__ketogluconate_metabolism_g__Escherichia_s__Escherichia_coli
KETOGLUCONMET_PWY__ketogluconate_metabolism_unclassified
LACTOSECAT_PWY__lactose_and_galactose_degradation_I_g__Enterococcus_s__Enterococcus_du
LACTOSECAT_PWY__lactose_and_galactose_degradation_I_g__Enterococcus_s__Enterococcus_fae
LACTOSECAT_PWY__lactose_and_galactose_degradation_I_g__Lactobacillus_s__Lactobacillus_cas
LACTOSECAT_PWY__lactose_and_galactose_degradation_I_g__Lactobacillus_s__Lactobacillus_rha
LACTOSECAT_PWY__lactose_and_galactose_degradation_I_g__Lactobacillus_s__Lactobacillus_rum
LACTOSECAT_PWY__lactose_and_galactose_degradation_I_g__Streptococcus_s__Streptococcus_a
LACTOSECAT_PWY__lactose_and_galactose_degradation_I_g__Streptococcus_s__Streptococcus_n
LACTOSECAT_PWY__lactose_and_galactose_degradation_I_g__Streptococcus_s__Streptococcus_s
LACTOSECAT_PWY__lactose_and_galactose_degradation_I_g__Streptococcus_s__Streptococcus_s
LIPASYN_PWY__phospholipases
MET_SAM_PWY__superpathway_of_S_adenosyl_L_methionine_biosynthesis_g__Anaerostipes_s_
MET_SAM_PWY__superpathway_of_S_adenosyl_L_methionine_biosynthesis_g__Clostridium_s__
MET_SAM_PWY__superpathway_of_S_adenosyl_L_methionine_biosynthesis_g__Enterobacter_s_
MET_SAM_PWY__superpathway_of_S_adenosyl_L_methionine_biosynthesis_g__Klebsiella_s__Kle
MET_SAM_PWY__superpathway_of_S_adenosyl_L_methionine_biosynthesis_g__Lachnospiraceae_
MET_SAM_PWY__superpathway_of_S_adenosyl_L_methionine_biosynthesis_g__Lactococcus_s__
MET_SAM_PWY__superpathway_of_S_adenosyl_L_methionine_biosynthesis_g__Streptococcus_s_
METSYN_PWY__L_homoserine_and_L_methionine_biosynthesis_g__Anaerostipes_s__Anaerostipe
METSYN_PWY__L_homoserine_and_L_methionine_biosynthesis_g__Clostridium_s__Clostridium_s
METSYN_PWY__L_homoserine_and_L_methionine_biosynthesis_g__Enterobacter_s__Enterobacte
METSYN_PWY__L_homoserine_and_L_methionine_biosynthesis_g__Klebsiella_s__Klebsiella_pneu

METSYN_PWY__L_homoserine_and_L_methionine_biosynthesis_g__Lachnospiraceae_noname_s_
METSYN_PWY__L_homoserine_and_L_methionine_biosynthesis_g__Lactococcus_s__Lactococcus_
METSYN_PWY__L_homoserine_and_L_methionine_biosynthesis_g__Streptococcus_s__Streptococ
NAD_BIOSYNTHESIS_II__NAD_salvage_pathway_II_g__Citrobacter_s__Citrobacter_freundii
NAD_BIOSYNTHESIS_II__NAD_salvage_pathway_II_g__Enterobacter_s__Enterobacter_cloacae
NAD_BIOSYNTHESIS_II__NAD_salvage_pathway_II_g__Klebsiella_s__Klebsiella_pneumoniae
NAD_BIOSYNTHESIS_II__NAD_salvage_pathway_II_g__Lactococcus_s__Lactococcus_lactis
NAD_BIOSYNTHESIS_II__NAD_salvage_pathway_II_unclassified
NAGLIPASYN_PWY__lipid_IVA_biosynthesis_g__Burkholderiales_noname_s__Burkholderiales_bac
NAGLIPASYN_PWY__lipid_IVA_biosynthesis_g__Citrobacter_s__Citrobacter_freundii
NAGLIPASYN_PWY__lipid_IVA_biosynthesis_g__Klebsiella_s__Klebsiella_pneumoniae
NAGLIPASYN_PWY__lipid_IVA_biosynthesis_g__Oxalobacter_s__Oxalobacter_formigenes
NAGLIPASYN_PWY__lipid_IVA_biosynthesis_g__Parasutterella_s__Parasutterella_excrementihomi
NAGLIPASYN_PWY__lipid_IVA_biosynthesis_unclassified
NONMEVIPP_PWY__methylerythritol_phosphate_pathway_I_g__Adlercreutzia_s__Adlercreutzia_e
NONMEVIPP_PWY__methylerythritol_phosphate_pathway_I_g__Akkermansia_s__Akkermansia_m
NONMEVIPP_PWY__methylerythritol_phosphate_pathway_I_g__Alistipes_s__Alistipes_finegoldii
NONMEVIPP_PWY__methylerythritol_phosphate_pathway_I_g__Alistipes_s__Alistipes_indistinctu
NONMEVIPP_PWY__methylerythritol_phosphate_pathway_I_g__Alistipes_s__Alistipes_putredinis
NONMEVIPP_PWY__methylerythritol_phosphate_pathway_I_g__Alistipes_s__Alistipes_sp_HGB5
NONMEVIPP_PWY__methylerythritol_phosphate_pathway_I_g__Anaerostipes_s__Anaerostipes_h
NONMEVIPP_PWY__methylerythritol_phosphate_pathway_I_g__Anaerotruncus_s__Anaerotruncu
NONMEVIPP_PWY__methylerythritol_phosphate_pathway_I_g__Bacteroides_s__Bacteroides_egg
NONMEVIPP_PWY__methylerythritol_phosphate_pathway_I_g__Bacteroides_s__Bacteroides_ova
NONMEVIPP_PWY__methylerythritol_phosphate_pathway_I_g__Bacteroides_s__Bacteroides_sp_
NONMEVIPP_PWY__methylerythritol_phosphate_pathway_I_g__Bacteroides_s__Bacteroides_sp_
NONMEVIPP_PWY__methylerythritol_phosphate_pathway_I_g__Bacteroides_s__Bacteroides_sp_
NONMEVIPP_PWY__methylerythritol_phosphate_pathway_I_g__Bacteroides_s__Bacteroides_sp_
NONMEVIPP_PWY__methylerythritol_phosphate_pathway_I_g__Bacteroides_s__Bacteroides_xyla
NONMEVIPP_PWY__methylerythritol_phosphate_pathway_I_g__Bifidobacterium_s__Bifidobacter
NONMEVIPP_PWY__methylerythritol_phosphate_pathway_I_g__Bifidobacterium_s__Bifidobacter
NONMEVIPP_PWY__methylerythritol_phosphate_pathway_I_g__Blautia_s__Blautia_sp_KLE_1732
NONMEVIPP_PWY__methylerythritol_phosphate_pathway_I_g__Blautia_s__Ruminococcus_gnavu
NONMEVIPP_PWY__methylerythritol_phosphate_pathway_I_g__Blautia_s__Ruminococcus_obeur
NONMEVIPP_PWY__methylerythritol_phosphate_pathway_I_g__Blautia_s__Ruminococcus_torque
NONMEVIPP_PWY__methylerythritol_phosphate_pathway_I_g__Burkholderiales_noname_s__Bur
NONMEVIPP_PWY__methylerythritol_phosphate_pathway_I_g__Butyrivibrio_s__Butyrivibrio_cros
NONMEVIPP_PWY__methylerythritol_phosphate_pathway_I_g__Citrobacter_s__Citrobacter_freur
NONMEVIPP_PWY__methylerythritol_phosphate_pathway_I_g__Clostridium_s__Clostridium_aspa
NONMEVIPP_PWY__methylerythritol_phosphate_pathway_I_g__Clostridium_s__Clostridium_hath
NONMEVIPP_PWY__methylerythritol_phosphate_pathway_I_g__Clostridium_s__Clostridium_perf
NONMEVIPP_PWY__methylerythritol_phosphate_pathway_I_g__Clostridium_s__Clostridium_sp_L
NONMEVIPP_PWY__methylerythritol_phosphate_pathway_I_g__Clostridium_s__Clostridium_sp_S
NONMEVIPP_PWY__methylerythritol_phosphate_pathway_I_g__Collinsella_s__Collinsella_intestir
NONMEVIPP_PWY__methylerythritol_phosphate_pathway_I_g__Collinsella_s__Collinsella_tanaka
NONMEVIPP_PWY__methylerythritol_phosphate_pathway_I_g__Coprobacter_s__Coprobacter_fas
NONMEVIPP_PWY__methylerythritol_phosphate_pathway_I_g__Dorea_s__Dorea_formicigeneran

NONMEVIPP_PWY__methylerythritol_phosphate_pathway_I_g__Eggerthella_s__Eggerthella_lenta
NONMEVIPP_PWY__methylerythritol_phosphate_pathway_I_g__Eggerthella_s__Eggerthella_sp_1
NONMEVIPP_PWY__methylerythritol_phosphate_pathway_I_g__Eggerthella_s__Eggerthella_sp_H
NONMEVIPP_PWY__methylerythritol_phosphate_pathway_I_g__Erysipelotrichaceae_noname_s__
NONMEVIPP_PWY__methylerythritol_phosphate_pathway_I_g__Erysipelotrichaceae_noname_s__
NONMEVIPP_PWY__methylerythritol_phosphate_pathway_I_g__Eubacterium_s__Eubacterium_ra
NONMEVIPP_PWY__methylerythritol_phosphate_pathway_I_g__Eubacterium_s__Eubacterium_sp
NONMEVIPP_PWY__methylerythritol_phosphate_pathway_I_g__Fusobacterium_s__Fusobacteriur
NONMEVIPP_PWY__methylerythritol_phosphate_pathway_I_g__Holdemania_s__Holdemania_filif
NONMEVIPP_PWY__methylerythritol_phosphate_pathway_I_g__Klebsiella_s__Klebsiella_pneumo
NONMEVIPP_PWY__methylerythritol_phosphate_pathway_I_g__Lachnospiraceae_noname_s__La
NONMEVIPP_PWY__methylerythritol_phosphate_pathway_I_g__Lachnospiraceae_noname_s__La
NONMEVIPP_PWY__methylerythritol_phosphate_pathway_I_g__Lachnospiraceae_noname_s__La
NONMEVIPP_PWY__methylerythritol_phosphate_pathway_I_g__Lachnospiraceae_noname_s__La
NONMEVIPP_PWY__methylerythritol_phosphate_pathway_I_g__Lachnospiraceae_noname_s__La
NONMEVIPP_PWY__methylerythritol_phosphate_pathway_I_g__Odoribacter_s__Odoribacter_lan
NONMEVIPP_PWY__methylerythritol_phosphate_pathway_I_g__Odoribacter_s__Odoribacter_spl
NONMEVIPP_PWY__methylerythritol_phosphate_pathway_I_g__Paraprevotella_s__Paraprevotella
NONMEVIPP_PWY__methylerythritol_phosphate_pathway_I_g__Parasutterella_s__Parasutterella_
NONMEVIPP_PWY__methylerythritol_phosphate_pathway_I_g__Peptostreptococcaceae_noname_
NONMEVIPP_PWY__methylerythritol_phosphate_pathway_I_g__Phascolarctobacterium_s__Phasc
NONMEVIPP_PWY__methylerythritol_phosphate_pathway_I_g__Propionibacterium_s__Propionib
NONMEVIPP_PWY__methylerythritol_phosphate_pathway_I_g__Roseburia_s__Roseburia_homini
NONMEVIPP_PWY__methylerythritol_phosphate_pathway_I_g__Ruminococcus_s__Ruminococcus
NONMEVIPP_PWY__methylerythritol_phosphate_pathway_I_g__Slackia_s__Slackia_piriformis
NONOXIPENT_PWY__pentose_phosphate_pathway_(non_oxidative_branch)_g__Alistipes_s__Alist
NONOXIPENT_PWY__pentose_phosphate_pathway_(non_oxidative_branch)_g__Anaerostipes_s__
NONOXIPENT_PWY__pentose_phosphate_pathway_(non_oxidative_branch)_g__Bacteroides_s__E
NONOXIPENT_PWY__pentose_phosphate_pathway_(non_oxidative_branch)_g__Bacteroides_s__E
NONOXIPENT_PWY__pentose_phosphate_pathway_(non_oxidative_branch)_g__Blautia_s__Rumir
NONOXIPENT_PWY__pentose_phosphate_pathway_(non_oxidative_branch)_g__Blautia_s__Rumir
NONOXIPENT_PWY__pentose_phosphate_pathway_(non_oxidative_branch)_g__Citrobacter_s__C
NONOXIPENT_PWY__pentose_phosphate_pathway_(non_oxidative_branch)_g__Clostridiales_non
NONOXIPENT_PWY__pentose_phosphate_pathway_(non_oxidative_branch)_g__Clostridium_s__C
NONOXIPENT_PWY__pentose_phosphate_pathway_(non_oxidative_branch)_g__Clostridium_s__C
NONOXIPENT_PWY__pentose_phosphate_pathway_(non_oxidative_branch)_g__Coprococcus_s__
NONOXIPENT_PWY__pentose_phosphate_pathway_(non_oxidative_branch)_g__Enterobacter_s__
NONOXIPENT_PWY__pentose_phosphate_pathway_(non_oxidative_branch)_g__Erysipelotrichace
NONOXIPENT_PWY__pentose_phosphate_pathway_(non_oxidative_branch)_g__Erysipelotrichace
NONOXIPENT_PWY__pentose_phosphate_pathway_(non_oxidative_branch)_g__Eubacterium_s__
NONOXIPENT_PWY__pentose_phosphate_pathway_(non_oxidative_branch)_g__Eubacterium_s__
NONOXIPENT_PWY__pentose_phosphate_pathway_(non_oxidative_branch)_g__Fusobacterium_s
NONOXIPENT_PWY__pentose_phosphate_pathway_(non_oxidative_branch)_g__Klebsiella_s__Kle
NONOXIPENT_PWY__pentose_phosphate_pathway_(non_oxidative_branch)_g__Lachnospiraceae_
NONOXIPENT_PWY__pentose_phosphate_pathway_(non_oxidative_branch)_g__Lachnospiraceae_
NONOXIPENT_PWY__pentose_phosphate_pathway_(non_oxidative_branch)_g__Lactobacillus_s__
NONOXIPENT_PWY__pentose_phosphate_pathway_(non_oxidative_branch)_g__Lactobacillus_s__

NONOXIPENT_PWY__pentose_phosphate_pathway_(non_oxidative_branch)_g__Lactococcus_s__L
NONOXIPENT_PWY__pentose_phosphate_pathway_(non_oxidative_branch)_g__Odoribacter_s__(
NONOXIPENT_PWY__pentose_phosphate_pathway_(non_oxidative_branch)_g__Oxalobacter_s__(
NONOXIPENT_PWY__pentose_phosphate_pathway_(non_oxidative_branch)_g__Peptostreptococc
NONOXIPENT_PWY__pentose_phosphate_pathway_(non_oxidative_branch)_g__Phascolarctobact
NONOXIPENT_PWY__pentose_phosphate_pathway_(non_oxidative_branch)_g__Propionibacteriur
NONOXIPENT_PWY__pentose_phosphate_pathway_(non_oxidative_branch)_g__Veillonella_s__Ve
NONOXIPENT_PWY__pentose_phosphate_pathway_(non_oxidative_branch)_g__Veillonella_s__Ve
OANTIGEN_PWY__O_antigen_building_blocks_biosynthesis_(E__coli)_g__Lactococcus_s__Lactoco
OANTIGEN_PWY__O_antigen_building_blocks_biosynthesis_(E__coli)_g__Ruminococcus_s__Rumi
ORNARGDEG_PWY__superpathway_of_L_arginine_and_L_ornithine_degradation_unclassified
P105_PWY__TCA_cycle_IV_(2_oxoglutarate_decarboxylase)_g__Citrobacter_s__Citrobacter_freun
P108_PWY__pyruvate_fermentation_to_propanoate_I_g__Propionibacterium_s__Propionibacteriu
P125_PWY__superpathway_of_(R,R)_butanediol_biosynthesis_g__Lactococcus_s__Lactococcus_la
P125_PWY__superpathway_of_(R,R)_butanediol_biosynthesis_unclassified
P161_PWY__acetylene_degradation_g__Citrobacter_s__Citrobacter_freundii
P161_PWY__acetylene_degradation_g__Enterobacter_s__Enterobacter_cloacae
P161_PWY__acetylene_degradation_g__Klebsiella_s__Klebsiella_pneumoniae
P163_PWY__L_lysine_fermentation_to_acetate_and_butanoate_g__Fusobacterium_s__Fusobacte
P185_PWY__formaldehyde_assimilation_III_(dihydroxyacetone_cycle)_g__Citrobacter_s__Citroba
P185_PWY__formaldehyde_assimilation_III_(dihydroxyacetone_cycle)_g__Escherichia_s__Escheri
P23_PWY__reductive_TCA_cycle_I_unclassified
P441_PWY__superpathway_of_N_acetylneuraminate_degradation_g__Citrobacter_s__Citrobacter
P461_PWY__hexitol_fermentation_to_lactate,_formate,_ethanol_and_acetate_g__Anaerostipes_s
P461_PWY__hexitol_fermentation_to_lactate,_formate,_ethanol_and_acetate_g__Citrobacter_s__
P461_PWY__hexitol_fermentation_to_lactate,_formate,_ethanol_and_acetate_g__Clostridium_s__
P461_PWY__hexitol_fermentation_to_lactate,_formate,_ethanol_and_acetate_g__Enterobacter_s
P461_PWY__hexitol_fermentation_to_lactate,_formate,_ethanol_and_acetate_g__Klebsiella_s__K
P461_PWY__hexitol_fermentation_to_lactate,_formate,_ethanol_and_acetate_g__Lachnospiracea
P461_PWY__hexitol_fermentation_to_lactate,_formate,_ethanol_and_acetate_g__Lactobacillus_s
P461_PWY__hexitol_fermentation_to_lactate,_formate,_ethanol_and_acetate_g__Lactobacillus_s
PANTO_PWY__phosphopantothenate_biosynthesis_I_g__Adlercreutzia_s__Adlercreutzia_equolifa
PANTO_PWY__phosphopantothenate_biosynthesis_I_g__Akkermansia_s__Akkermansia_muciniph
PANTO_PWY__phosphopantothenate_biosynthesis_I_g__Alistipes_s__Alistipes_finegoldii
PANTO_PWY__phosphopantothenate_biosynthesis_I_g__Alistipes_s__Alistipes_indistinctus
PANTO_PWY__phosphopantothenate_biosynthesis_I_g__Alistipes_s__Alistipes_putredinis
PANTO_PWY__phosphopantothenate_biosynthesis_I_g__Alistipes_s__Alistipes_sp_HGB5
PANTO_PWY__phosphopantothenate_biosynthesis_I_g__Anaerofustis_s__Anaerofustis_stercorihc
PANTO_PWY__phosphopantothenate_biosynthesis_I_g__Anaerostipes_s__Anaerostipes_hadrus
PANTO_PWY__phosphopantothenate_biosynthesis_I_g__Bacteroides_s__Bacteroides_cellulosilyti
PANTO_PWY__phosphopantothenate_biosynthesis_I_g__Bacteroides_s__Bacteroides_clarus
PANTO_PWY__phosphopantothenate_biosynthesis_I_g__Bacteroides_s__Bacteroides_coprocola
PANTO_PWY__phosphopantothenate_biosynthesis_I_g__Bacteroides_s__Bacteroides_dorei
PANTO_PWY__phosphopantothenate_biosynthesis_I_g__Bacteroides_s__Bacteroides_eggerthii
PANTO_PWY__phosphopantothenate_biosynthesis_I_g__Bacteroides_s__Bacteroides_faecis
PANTO_PWY__phosphopantothenate_biosynthesis_I_g__Bacteroides_s__Bacteroides_finegoldii
PANTO_PWY__phosphopantothenate_biosynthesis_I_g__Bacteroides_s__Bacteroides_intestinalis

PANTO_PWY__phosphopantothenate_biosynthesis_I_g__Bacteroides_s__Bacteroides_massiliensi
PANTO_PWY__phosphopantothenate_biosynthesis_I_g__Bacteroides_s__Bacteroides_ovatus
PANTO_PWY__phosphopantothenate_biosynthesis_I_g__Bacteroides_s__Bacteroides_plebeius
PANTO_PWY__phosphopantothenate_biosynthesis_I_g__Bacteroides_s__Bacteroides_sp_1_1_30
PANTO_PWY__phosphopantothenate_biosynthesis_I_g__Bacteroides_s__Bacteroides_sp_1_1_6
PANTO_PWY__phosphopantothenate_biosynthesis_I_g__Bacteroides_s__Bacteroides_sp_2_1_22
PANTO_PWY__phosphopantothenate_biosynthesis_I_g__Bacteroides_s__Bacteroides_sp_2_2_4
PANTO_PWY__phosphopantothenate_biosynthesis_I_g__Bacteroides_s__Bacteroides_sp_3_1_19
PANTO_PWY__phosphopantothenate_biosynthesis_I_g__Bacteroides_s__Bacteroides_sp_3_1_23
PANTO_PWY__phosphopantothenate_biosynthesis_I_g__Bacteroides_s__Bacteroides_sp_3_1_33
PANTO_PWY__phosphopantothenate_biosynthesis_I_g__Bacteroides_s__Bacteroides_sp_3_1_40
PANTO_PWY__phosphopantothenate_biosynthesis_I_g__Bacteroides_s__Bacteroides_sp_4_3_47
PANTO_PWY__phosphopantothenate_biosynthesis_I_g__Bacteroides_s__Bacteroides_sp_9_1_42
PANTO_PWY__phosphopantothenate_biosynthesis_I_g__Bacteroides_s__Bacteroides_sp_D20
PANTO_PWY__phosphopantothenate_biosynthesis_I_g__Bacteroides_s__Bacteroides_stercoris
PANTO_PWY__phosphopantothenate_biosynthesis_I_g__Bacteroides_s__Bacteroides_thetaiotaon
PANTO_PWY__phosphopantothenate_biosynthesis_I_g__Bacteroides_s__Bacteroides_uniformis
PANTO_PWY__phosphopantothenate_biosynthesis_I_g__Bacteroides_s__Bacteroides_xylanisolve
PANTO_PWY__phosphopantothenate_biosynthesis_I_g__Barnesiella_s__Barnesiella_intestinihom
PANTO_PWY__phosphopantothenate_biosynthesis_I_g__Bilophila_s__Bilophila_wadsworthia
PANTO_PWY__phosphopantothenate_biosynthesis_I_g__Blautia_s__Blautia_hydrogenotrophica
PANTO_PWY__phosphopantothenate_biosynthesis_I_g__Blautia_s__Blautia_sp_KLE_1732
PANTO_PWY__phosphopantothenate_biosynthesis_I_g__Blautia_s__Ruminococcus_gnavus
PANTO_PWY__phosphopantothenate_biosynthesis_I_g__Burkholderiales_noname_s__Burkholder
PANTO_PWY__phosphopantothenate_biosynthesis_I_g__Butyrivibrio_s__Butyrivibrio_crossotus
PANTO_PWY__phosphopantothenate_biosynthesis_I_g__Citrobacter_s__Citrobacter_freundii
PANTO_PWY__phosphopantothenate_biosynthesis_I_g__Clostridium_s__Clostridium_sp_L2_50
PANTO_PWY__phosphopantothenate_biosynthesis_I_g__Clostridium_s__Clostridium_sp_SS2_1
PANTO_PWY__phosphopantothenate_biosynthesis_I_g__Coprobacter_s__Coprobacter_fastidiosu
PANTO_PWY__phosphopantothenate_biosynthesis_I_g__Coprococcus_s__Coprococcus_eutactus
PANTO_PWY__phosphopantothenate_biosynthesis_I_g__Coprococcus_s__Coprococcus_sp_ART55
PANTO_PWY__phosphopantothenate_biosynthesis_I_g__Eggerthella_s__Eggerthella_lenta
PANTO_PWY__phosphopantothenate_biosynthesis_I_g__Eggerthella_s__Eggerthella_sp_1_3_56F
PANTO_PWY__phosphopantothenate_biosynthesis_I_g__Eggerthella_s__Eggerthella_sp_HGA1
PANTO_PWY__phosphopantothenate_biosynthesis_I_g__Enterobacter_s__Enterobacter_cloacae
PANTO_PWY__phosphopantothenate_biosynthesis_I_g__Enterococcus_s__Enterococcus_faecalis
PANTO_PWY__phosphopantothenate_biosynthesis_I_g__Enterorhabdus_s__Enterorhabdus_caeci
PANTO_PWY__phosphopantothenate_biosynthesis_I_g__Eubacterium_s__Eubacterium_hallii
PANTO_PWY__phosphopantothenate_biosynthesis_I_g__Eubacterium_s__Eubacterium_ramulus
PANTO_PWY__phosphopantothenate_biosynthesis_I_g__Eubacterium_s__Eubacterium_ventriosu
PANTO_PWY__phosphopantothenate_biosynthesis_I_g__Klebsiella_s__Klebsiella_pneumoniae
PANTO_PWY__phosphopantothenate_biosynthesis_I_g__Lachnospiraceae_noname_s__Lachnospi
PANTO_PWY__phosphopantothenate_biosynthesis_I_g__Lachnospiraceae_noname_s__Lachnospi
PANTO_PWY__phosphopantothenate_biosynthesis_I_g__Lachnospiraceae_noname_s__Lachnospi
PANTO_PWY__phosphopantothenate_biosynthesis_I_g__Lachnospiraceae_noname_s__Lachnospi
PANTO_PWY__phosphopantothenate_biosynthesis_I_g__Odoribacter_s__Odoribacter_laneus
PANTO_PWY__phosphopantothenate_biosynthesis_I_g__Odoribacter_s__Odoribacter_splanchnic

PANTO_PWY__phosphopantothenate_biosynthesis_I_g__Oxalobacter_s__Oxalobacter_formigenes
PANTO_PWY__phosphopantothenate_biosynthesis_I_g__Parabacteroides_s__Parabacteroides_dis
PANTO_PWY__phosphopantothenate_biosynthesis_I_g__Parabacteroides_s__Parabacteroides_go
PANTO_PWY__phosphopantothenate_biosynthesis_I_g__Parabacteroides_s__Parabacteroides_joh
PANTO_PWY__phosphopantothenate_biosynthesis_I_g__Parabacteroides_s__Parabacteroides_me
PANTO_PWY__phosphopantothenate_biosynthesis_I_g__Parabacteroides_s__Parabacteroides_sp
PANTO_PWY__phosphopantothenate_biosynthesis_I_g__Parabacteroides_s__Parabacteroides_sp
PANTO_PWY__phosphopantothenate_biosynthesis_I_g__Paraprevotella_s__Paraprevotella_clara
PANTO_PWY__phosphopantothenate_biosynthesis_I_g__Parasutterella_s__Parasutterella_excrem
PANTO_PWY__phosphopantothenate_biosynthesis_I_g__Phascolarctobacterium_s__Phascolarctob
PANTO_PWY__phosphopantothenate_biosynthesis_I_g__Roseburia_s__Roseburia_hominis
PANTO_PWY__phosphopantothenate_biosynthesis_I_g__Ruminococcus_s__Ruminococcus_callidu
PANTO_PWY__phosphopantothenate_biosynthesis_I_g__Ruminococcus_s__Ruminococcus_sp
PANTOSYN_PWY__pantothenate_and_coenzyme_A_biosynthesis_I_g__Actinomyces_s__Actinomy
PANTOSYN_PWY__pantothenate_and_coenzyme_A_biosynthesis_I_g__Akkermansia_s__Akkerma
PANTOSYN_PWY__pantothenate_and_coenzyme_A_biosynthesis_I_g__Anaerofustis_s__Anaerofu
PANTOSYN_PWY__pantothenate_and_coenzyme_A_biosynthesis_I_g__Anaerostipes_s__Anaerost
PANTOSYN_PWY__pantothenate_and_coenzyme_A_biosynthesis_I_g__Bacteroides_s__Bacteroide
PANTOSYN_PWY__pantothenate_and_coenzyme_A_biosynthesis_I_g__Bacteroides_s__Bacteroide
PANTOSYN_PWY__pantothenate_and_coenzyme_A_biosynthesis_I_g__Bacteroides_s__Bacteroide
PANTOSYN_PWY__pantothenate_and_coenzyme_A_biosynthesis_I_g__Bacteroides_s__Bacteroide
PANTOSYN_PWY__pantothenate_and_coenzyme_A_biosynthesis_I_g__Bacteroides_s__Bacteroide
PANTOSYN_PWY__pantothenate_and_coenzyme_A_biosynthesis_I_g__Bacteroides_s__Bacteroide
PANTOSYN_PWY__pantothenate_and_coenzyme_A_biosynthesis_I_g__Citrobacter_s__Citrobacter
PANTOSYN_PWY__pantothenate_and_coenzyme_A_biosynthesis_I_g__Clostridium_s__Clostridiur
PANTOSYN_PWY__pantothenate_and_coenzyme_A_biosynthesis_I_g__Eggerthella_s__Eggerthella
PANTOSYN_PWY__pantothenate_and_coenzyme_A_biosynthesis_I_g__Eggerthella_s__Eggerthella
PANTOSYN_PWY__pantothenate_and_coenzyme_A_biosynthesis_I_g__Eubacterium_s__Eubacteri
PANTOSYN_PWY__pantothenate_and_coenzyme_A_biosynthesis_I_g__Lachnospiraceae_noname_
PANTOSYN_PWY__pantothenate_and_coenzyme_A_biosynthesis_I_g__Odoribacter_s__Odoribact
PANTOSYN_PWY__pantothenate_and_coenzyme_A_biosynthesis_I_g__Phascolarctobacterium_s_
PENTOSE_P_PWY__pentose_phosphate_pathway_g__Citrobacter_s__Citrobacter_freundii
PENTOSE_P_PWY__pentose_phosphate_pathway_g__Enterobacter_s__Enterobacter_cloacae
PENTOSE_P_PWY__pentose_phosphate_pathway_g__Klebsiella_s__Klebsiella_pneumoniae
PENTOSE_P_PWY__pentose_phosphate_pathway_g__Odoribacter_s__Odoribacter_splanchnicus
PENTOSE_P_PWY__pentose_phosphate_pathway_g__Propionibacterium_s__Propionibacterium_fr
PEPTIDOGLYCANSYN_PWY__peptidoglycan_biosynthesis_I_(meso_diaminopimelate_containing)_g
PEPTIDOGLYCANSYN_PWY__peptidoglycan_biosynthesis_I_(meso_diaminopimelate_containing)_g
PEPTIDOGLYCANSYN_PWY__peptidoglycan_biosynthesis_I_(meso_diaminopimelate_containing)_g
PEPTIDOGLYCANSYN_PWY__peptidoglycan_biosynthesis_I_(meso_diaminopimelate_containing)_g
PEPTIDOGLYCANSYN_PWY__peptidoglycan_biosynthesis_I_(meso_diaminopimelate_containing)_g
PEPTIDOGLYCANSYN_PWY__peptidoglycan_biosynthesis_I_(meso_diaminopimelate_containing)_g
PEPTIDOGLYCANSYN_PWY__peptidoglycan_biosynthesis_I_(meso_diaminopimelate_containing)_g
PEPTIDOGLYCANSYN_PWY__peptidoglycan_biosynthesis_I_(meso_diaminopimelate_containing)_g
PEPTIDOGLYCANSYN_PWY__peptidoglycan_biosynthesis_I_(meso_diaminopimelate_containing)_g
PEPTIDOGLYCANSYN_PWY__peptidoglycan_biosynthesis_I_(meso_diaminopimelate_containing)_g
PEPTIDOGLYCANSYN_PWY__peptidoglycan_biosynthesis_I_(meso_diaminopimelate_containing)_g

PEPTIDOGLYCANSYN_PWY__peptidoglycan_biosynthesis_I_(meso_diaminopimelate_containing)_g
PEPTIDOGLYCANSYN_PWY__peptidoglycan_biosynthesis_I_(meso_diaminopimelate_containing)_g
PEPTIDOGLYCANSYN_PWY__peptidoglycan_biosynthesis_I_(meso_diaminopimelate_containing)_g
PEPTIDOGLYCANSYN_PWY__peptidoglycan_biosynthesis_I_(meso_diaminopimelate_containing)_g
PEPTIDOGLYCANSYN_PWY__peptidoglycan_biosynthesis_I_(meso_diaminopimelate_containing)_g
PEPTIDOGLYCANSYN_PWY__peptidoglycan_biosynthesis_I_(meso_diaminopimelate_containing)_g
PEPTIDOGLYCANSYN_PWY__peptidoglycan_biosynthesis_I_(meso_diaminopimelate_containing)_g
PEPTIDOGLYCANSYN_PWY__peptidoglycan_biosynthesis_I_(meso_diaminopimelate_containing)_g
PEPTIDOGLYCANSYN_PWY__peptidoglycan_biosynthesis_I_(meso_diaminopimelate_containing)_g
PEPTIDOGLYCANSYN_PWY__peptidoglycan_biosynthesis_I_(meso_diaminopimelate_containing)_g
PEPTIDOGLYCANSYN_PWY__peptidoglycan_biosynthesis_I_(meso_diaminopimelate_containing)_g
PEPTIDOGLYCANSYN_PWY__peptidoglycan_biosynthesis_I_(meso_diaminopimelate_containing)_g
PEPTIDOGLYCANSYN_PWY__peptidoglycan_biosynthesis_I_(meso_diaminopimelate_containing)_g
PEPTIDOGLYCANSYN_PWY__peptidoglycan_biosynthesis_I_(meso_diaminopimelate_containing)_g
PEPTIDOGLYCANSYN_PWY__peptidoglycan_biosynthesis_I_(meso_diaminopimelate_containing)_g
PEPTIDOGLYCANSYN_PWY__peptidoglycan_biosynthesis_I_(meso_diaminopimelate_containing)_g
PEPTIDOGLYCANSYN_PWY__peptidoglycan_biosynthesis_I_(meso_diaminopimelate_containing)_g
PEPTIDOGLYCANSYN_PWY__peptidoglycan_biosynthesis_I_(meso_diaminopimelate_containing)_g
PEPTIDOGLYCANSYN_PWY__peptidoglycan_biosynthesis_I_(meso_diaminopimelate_containing)_g
PEPTIDOGLYCANSYN_PWY__peptidoglycan_biosynthesis_I_(meso_diaminopimelate_containing)_g
PEPTIDOGLYCANSYN_PWY__peptidoglycan_biosynthesis_I_(meso_diaminopimelate_containing)_g
PEPTIDOGLYCANSYN_PWY__peptidoglycan_biosynthesis_I_(meso_diaminopimelate_containing)_g
PEPTIDOGLYCANSYN_PWY__peptidoglycan_biosynthesis_I_(meso_diaminopimelate_containing)_g
PEPTIDOGLYCANSYN_PWY__peptidoglycan_biosynthesis_I_(meso_diaminopimelate_containing)_g
PEPTIDOGLYCANSYN_PWY__peptidoglycan_biosynthesis_I_(meso_diaminopimelate_containing)_g
PEPTIDOGLYCANSYN_PWY__peptidoglycan_biosynthesis_I_(meso_diaminopimelate_containing)_g
PEPTIDOGLYCANSYN_PWY__peptidoglycan_biosynthesis_I_(meso_diaminopimelate_containing)_g
PEPTIDOGLYCANSYN_PWY__peptidoglycan_biosynthesis_I_(meso_diaminopimelate_containing)_g
PEPTIDOGLYCANSYN_PWY__peptidoglycan_biosynthesis_I_(meso_diaminopimelate_containing)_g
PEPTIDOGLYCANSYN_PWY__peptidoglycan_biosynthesis_I_(meso_diaminopimelate_containing)_g
PEPTIDOGLYCANSYN_PWY__peptidoglycan_biosynthesis_I_(meso_diaminopimelate_containing)_g
PEPTIDOGLYCANSYN_PWY__peptidoglycan_biosynthesis_I_(meso_diaminopimelate_containing)_g
PEPTIDOGLYCANSYN_PWY__peptidoglycan_biosynthesis_I_(meso_diaminopimelate_containing)_g
PEPTIDOGLYCANSYN_PWY__peptidoglycan_biosynthesis_I_(meso_diaminopimelate_containing)_g
PEPTIDOGLYCANSYN_PWY__peptidoglycan_biosynthesis_I_(meso_diaminopimelate_containing)_g
PEPTIDOGLYCANSYN_PWY__peptidoglycan_biosynthesis_I_(meso_diaminopimelate_containing)_g
PEPTIDOGLYCANSYN_PWY__peptidoglycan_biosynthesis_I_(meso_diaminopimelate_containing)_g
PEPTIDOGLYCANSYN_PWY__peptidoglycan_biosynthesis_I_(meso_diaminopimelate_containing)_g
PEPTIDOGLYCANSYN_PWY__peptidoglycan_biosynthesis_I_(meso_diaminopimelate_containing)_g
PEPTIDOGLYCANSYN_PWY__peptidoglycan_biosynthesis_I_(meso_diaminopimelate_containing)_g
PEPTIDOGLYCANSYN_PWY__peptidoglycan_biosynthesis_I_(meso_diaminopimelate_containing)_g
PEPTIDOGLYCANSYN_PWY__peptidoglycan_biosynthesis_I_(meso_diaminopimelate_containing)_g
PEPTIDOGLYCANSYN_PWY__peptidoglycan_biosynthesis_I_(meso_diaminopimelate_containing)_g
PEPTIDOGLYCANSYN_PWY__peptidoglycan_biosynthesis_I_(meso_diaminopimelate_containing)_g
PEPTIDOGLYCANSYN_PWY__peptidoglycan_biosynthesis_I_(meso_diaminopimelate_containing)_g
PEPTIDOGLYCANSYN_PWY__peptidoglycan_biosynthesis_I_(meso_diaminopimelate_containing)_g
PEPTIDOGLYCANSYN_PWY__peptidoglycan_biosynthesis_I_(meso_diaminopimelate_containing)_g

PEPTIDOGLYCANSYN_PWY__peptidoglycan_biosynthesis_I_(meso_diaminopimelate_containing)_g
PEPTIDOGLYCANSYN_PWY__peptidoglycan_biosynthesis_I_(meso_diaminopimelate_containing)_g
PEPTIDOGLYCANSYN_PWY__peptidoglycan_biosynthesis_I_(meso_diaminopimelate_containing)_g
PEPTIDOGLYCANSYN_PWY__peptidoglycan_biosynthesis_I_(meso_diaminopimelate_containing)_g
PEPTIDOGLYCANSYN_PWY__peptidoglycan_biosynthesis_I_(meso_diaminopimelate_containing)_g
PEPTIDOGLYCANSYN_PWY__peptidoglycan_biosynthesis_I_(meso_diaminopimelate_containing)_g
PEPTIDOGLYCANSYN_PWY__peptidoglycan_biosynthesis_I_(meso_diaminopimelate_containing)_g
PEPTIDOGLYCANSYN_PWY__peptidoglycan_biosynthesis_I_(meso_diaminopimelate_containing)_g
PEPTIDOGLYCANSYN_PWY__peptidoglycan_biosynthesis_I_(meso_diaminopimelate_containing)_g
PEPTIDOGLYCANSYN_PWY__peptidoglycan_biosynthesis_I_(meso_diaminopimelate_containing)_g
PEPTIDOGLYCANSYN_PWY__peptidoglycan_biosynthesis_I_(meso_diaminopimelate_containing)_g
PEPTIDOGLYCANSYN_PWY__peptidoglycan_biosynthesis_I_(meso_diaminopimelate_containing)_g
PEPTIDOGLYCANSYN_PWY__peptidoglycan_biosynthesis_I_(meso_diaminopimelate_containing)_g
PEPTIDOGLYCANSYN_PWY__peptidoglycan_biosynthesis_I_(meso_diaminopimelate_containing)_g
PEPTIDOGLYCANSYN_PWY__peptidoglycan_biosynthesis_I_(meso_diaminopimelate_containing)_g
PEPTIDOGLYCANSYN_PWY__peptidoglycan_biosynthesis_I_(meso_diaminopimelate_containing)_g
PEPTIDOGLYCANSYN_PWY__peptidoglycan_biosynthesis_I_(meso_diaminopimelate_containing)_g
PEPTIDOGLYCANSYN_PWY__peptidoglycan_biosynthesis_I_(meso_diaminopimelate_containing)_g
PEPTIDOGLYCANSYN_PWY__peptidoglycan_biosynthesis_I_(meso_diaminopimelate_containing)_g
PEPTIDOGLYCANSYN_PWY__peptidoglycan_biosynthesis_I_(meso_diaminopimelate_containing)_g
PEPTIDOGLYCANSYN_PWY__peptidoglycan_biosynthesis_I_(meso_diaminopimelate_containing)_g
PEPTIDOGLYCANSYN_PWY__peptidoglycan_biosynthesis_I_(meso_diaminopimelate_containing)_g
PEPTIDOGLYCANSYN_PWY__peptidoglycan_biosynthesis_I_(meso_diaminopimelate_containing)_g
PEPTIDOGLYCANSYN_PWY__peptidoglycan_biosynthesis_I_(meso_diaminopimelate_containing)_g
PEPTIDOGLYCANSYN_PWY__peptidoglycan_biosynthesis_I_(meso_diaminopimelate_containing)_g
PEPTIDOGLYCANSYN_PWY__peptidoglycan_biosynthesis_I_(meso_diaminopimelate_containing)_g
PEPTIDOGLYCANSYN_PWY__peptidoglycan_biosynthesis_I_(meso_diaminopimelate_containing)_g
PEPTIDOGLYCANSYN_PWY__peptidoglycan_biosynthesis_I_(meso_diaminopimelate_containing)_g
PEPTIDOGLYCANSYN_PWY__peptidoglycan_biosynthesis_I_(meso_diaminopimelate_containing)_g
PEPTIDOGLYCANSYN_PWY__peptidoglycan_biosynthesis_I_(meso_diaminopimelate_containing)_g
PEPTIDOGLYCANSYN_PWY__peptidoglycan_biosynthesis_I_(meso_diaminopimelate_containing)_g
PEPTIDOGLYCANSYN_PWY__peptidoglycan_biosynthesis_I_(meso_diaminopimelate_containing)_g
PEPTIDOGLYCANSYN_PWY__peptidoglycan_biosynthesis_I_(meso_diaminopimelate_containing)_g
PEPTIDOGLYCANSYN_PWY__peptidoglycan_biosynthesis_I_(meso_diaminopimelate_containing)_g
PHOSLIPSYN_PWY__superpathway_of_phospholipid_biosynthesis_I_(bacteria)_g__Citrobacter_s__
PHOSLIPSYN_PWY__superpathway_of_phospholipid_biosynthesis_I_(bacteria)_g__Enterobacter_s
PHOSLIPSYN_PWY__superpathway_of_phospholipid_biosynthesis_I_(bacteria)_g__Klebsiella_s__K
POLYAMINSYN3_PWY__superpathway_of_polyamine_biosynthesis_II_unclassified
POLYAMSYN_PWY__superpathway_of_polyamine_biosynthesis_I_g__Veillonella_s__Veillonella_pa
PPGPPMET_PWY__ppGpp_biosynthesis_g__Citrobacter_s__Citrobacter_freundii
PPGPPMET_PWY__ppGpp_biosynthesis_g__Enterobacter_s__Enterobacter_cloacae
PPGPPMET_PWY__ppGpp_biosynthesis_g__Klebsiella_s__Klebsiella_pneumoniae
PROTOCATECHUATE_ORTHO_CLEAVAGE_PWY__protocatechuate_degradation_II_(ortho_cleavage
PROTOCATECHUATE_ORTHO_CLEAVAGE_PWY__protocatechuate_degradation_II_(ortho_cleavage
PROTOCATECHUATE_ORTHO_CLEAVAGE_PWY__protocatechuate_degradation_II_(ortho_cleavage
PWY_101__photosynthesis_light_reactions
PWY_101__photosynthesis_light_reactions_unclassified

PWY_1042__glycolysis_IV_(plant_cytosol)_g__Akkermansia_s__Akkermansia_muciniphila
PWY_1042__glycolysis_IV_(plant_cytosol)_g__Alistipes_s__Alistipes_senegalensis
PWY_1042__glycolysis_IV_(plant_cytosol)_g__Alistipes_s__Alistipes_shahii
PWY_1042__glycolysis_IV_(plant_cytosol)_g__Anaerofustis_s__Anaerofustis_stercorihominis
PWY_1042__glycolysis_IV_(plant_cytosol)_g__Anaerostipes_s__Anaerostipes_hadrus
PWY_1042__glycolysis_IV_(plant_cytosol)_g__Anaerotruncus_s__Anaerotruncus_colihominis
PWY_1042__glycolysis_IV_(plant_cytosol)_g__Bacteroides_s__Bacteroides_coprocola
PWY_1042__glycolysis_IV_(plant_cytosol)_g__Bacteroides_s__Bacteroides_faecis
PWY_1042__glycolysis_IV_(plant_cytosol)_g__Bacteroides_s__Bacteroides_finegoldii
PWY_1042__glycolysis_IV_(plant_cytosol)_g__Bacteroides_s__Bacteroides_ovatus
PWY_1042__glycolysis_IV_(plant_cytosol)_g__Bacteroides_s__Bacteroides_sp_1_1_30
PWY_1042__glycolysis_IV_(plant_cytosol)_g__Bacteroides_s__Bacteroides_sp_1_1_6
PWY_1042__glycolysis_IV_(plant_cytosol)_g__Bacteroides_s__Bacteroides_sp_2_1_22
PWY_1042__glycolysis_IV_(plant_cytosol)_g__Bacteroides_s__Bacteroides_sp_2_2_4
PWY_1042__glycolysis_IV_(plant_cytosol)_g__Bacteroides_s__Bacteroides_sp_3_1_23
PWY_1042__glycolysis_IV_(plant_cytosol)_g__Bacteroides_s__Bacteroides_thetaiotaomicron
PWY_1042__glycolysis_IV_(plant_cytosol)_g__Bacteroides_s__Bacteroides_xylanisolvens
PWY_1042__glycolysis_IV_(plant_cytosol)_g__Barnesiella_s__Barnesiella_intestinihominis
PWY_1042__glycolysis_IV_(plant_cytosol)_g__Blautia_s__Blautia_sp_KLE_1732
PWY_1042__glycolysis_IV_(plant_cytosol)_g__Blautia_s__Ruminococcus_obeum
PWY_1042__glycolysis_IV_(plant_cytosol)_g__Citrobacter_s__Citrobacter_freundii
PWY_1042__glycolysis_IV_(plant_cytosol)_g__Clostridiales_noname_s__Clostridiales_bacterium_1
PWY_1042__glycolysis_IV_(plant_cytosol)_g__Clostridium_s__Clostridium_asparagiforme
PWY_1042__glycolysis_IV_(plant_cytosol)_g__Clostridium_s__Clostridium_perfringens
PWY_1042__glycolysis_IV_(plant_cytosol)_g__Clostridium_s__Clostridium_sp_SS2_1
PWY_1042__glycolysis_IV_(plant_cytosol)_g__Coprococcus_s__Coprococcus_catus
PWY_1042__glycolysis_IV_(plant_cytosol)_g__Eggerthella_s__Eggerthella_lenta
PWY_1042__glycolysis_IV_(plant_cytosol)_g__Eggerthella_s__Eggerthella_sp_1_3_56FAA
PWY_1042__glycolysis_IV_(plant_cytosol)_g__Eggerthella_s__Eggerthella_sp_HGA1
PWY_1042__glycolysis_IV_(plant_cytosol)_g__Enterococcus_s__Enterococcus_faecium
PWY_1042__glycolysis_IV_(plant_cytosol)_g__Holdemania_s__Holdemania_filiformis
PWY_1042__glycolysis_IV_(plant_cytosol)_g__Klebsiella_s__Klebsiella_pneumoniae
PWY_1042__glycolysis_IV_(plant_cytosol)_g__Lachnospiraceae_noname_s__Lachnospiraceae_bac
PWY_1042__glycolysis_IV_(plant_cytosol)_g__Lachnospiraceae_noname_s__Lachnospiraceae_bac
PWY_1042__glycolysis_IV_(plant_cytosol)_g__Lachnospiraceae_noname_s__Lachnospiraceae_bac
PWY_1042__glycolysis_IV_(plant_cytosol)_g__Odoribacter_s__Odoribacter_splanchnicus
PWY_1042__glycolysis_IV_(plant_cytosol)_g__Pediococcus_s__Pediococcus_acidilactici
PWY_1042__glycolysis_IV_(plant_cytosol)_g__Pediococcus_s__Pediococcus_lolii
PWY_1042__glycolysis_IV_(plant_cytosol)_g__Peptostreptococcaceae_noname_s__Clostridium_ba
PWY_1042__glycolysis_IV_(plant_cytosol)_g__Ruminococcus_s__Ruminococcus_champanellensis
PWY_1042__glycolysis_IV_(plant_cytosol)_g__Ruminococcus_s__Ruminococcus_sp
PWY_1042__glycolysis_IV_(plant_cytosol)_g__Streptococcus_s__Streptococcus_australis
PWY_1042__glycolysis_IV_(plant_cytosol)_g__Streptococcus_s__Streptococcus_mitis_oralis_pneu
PWY_1269__CMP_3_deoxy_D_manno_octulosonate_biosynthesis_I_g__Akkermansia_s__Akkerma
PWY_1269__CMP_3_deoxy_D_manno_octulosonate_biosynthesis_I_g__Alistipes_s__Alistipes_ser
PWY_1269__CMP_3_deoxy_D_manno_octulosonate_biosynthesis_I_g__Alistipes_s__Alistipes_sha
PWY_1269__CMP_3_deoxy_D_manno_octulosonate_biosynthesis_I_g__Bacteroides_s__Bacteroid

PWY_1269__CMP_3_deoxy_D_manno_octulosonate_biosynthesis_I_g__Bacteroides_s__Bacteroid
PWY_1269__CMP_3_deoxy_D_manno_octulosonate_biosynthesis_I_g__Bacteroides_s__Bacteroid
PWY_1269__CMP_3_deoxy_D_manno_octulosonate_biosynthesis_I_g__Bacteroides_s__Bacteroid
PWY_1269__CMP_3_deoxy_D_manno_octulosonate_biosynthesis_I_g__Bacteroides_s__Bacteroid
PWY_1269__CMP_3_deoxy_D_manno_octulosonate_biosynthesis_I_g__Bacteroides_s__Bacteroid
PWY_1269__CMP_3_deoxy_D_manno_octulosonate_biosynthesis_I_g__Bacteroides_s__Bacteroid
PWY_1269__CMP_3_deoxy_D_manno_octulosonate_biosynthesis_I_g__Bacteroides_s__Bacteroid
PWY_1269__CMP_3_deoxy_D_manno_octulosonate_biosynthesis_I_g__Bacteroides_s__Bacteroid
PWY_1269__CMP_3_deoxy_D_manno_octulosonate_biosynthesis_I_g__Bacteroides_s__Bacteroid
PWY_1269__CMP_3_deoxy_D_manno_octulosonate_biosynthesis_I_g__Citrobacter_s__Citrobacte
PWY_1269__CMP_3_deoxy_D_manno_octulosonate_biosynthesis_I_g__Odoribacter_s__Odoribac
PWY_1269__CMP_3_deoxy_D_manno_octulosonate_biosynthesis_I_g__Oxalobacter_s__Oxalobac
PWY_1861__formaldehyde_assimilation_II_(RuMP_Cycle)_g__Blautia_s__Ruminococcus_obeum
PWY_1861__formaldehyde_assimilation_II_(RuMP_Cycle)_unclassified
PWY_2941__L_lysine_biosynthesis_II_g__Catenibacterium_s__Catenibacterium_mitsuokai
PWY_2941__L_lysine_biosynthesis_II_g__Enterococcus_s__Enterococcus_durans
PWY_2941__L_lysine_biosynthesis_II_g__Enterococcus_s__Enterococcus_faecalis
PWY_2941__L_lysine_biosynthesis_II_g__Erysipelotrichaceae_noname_s__Clostridium_spiroforme
PWY_2941__L_lysine_biosynthesis_II_g__Erysipelotrichaceae_noname_s__Erysipelotrichaceae_ba
PWY_2941__L_lysine_biosynthesis_II_g__Lactobacillus_s__Lactobacillus_casei_paracasei
PWY_2941__L_lysine_biosynthesis_II_g__Lactobacillus_s__Lactobacillus_fermentum
PWY_2941__L_lysine_biosynthesis_II_g__Lactococcus_s__Lactococcus_lactis
PWY_2941__L_lysine_biosynthesis_II_g__Odoribacter_s__Odoribacter_splanchnicus
PWY_2941__L_lysine_biosynthesis_II_g__Peptostreptococcaceae_noname_s__Clostridium_bartlet
PWY_2942__L_lysine_biosynthesis_III_g__Adlercreutzia_s__Adlercreutzia_equolifaciens
PWY_2942__L_lysine_biosynthesis_III_g__Akkermansia_s__Akkermansia_muciniphila
PWY_2942__L_lysine_biosynthesis_III_g__Alistipes_s__Alistipes_indistinctus
PWY_2942__L_lysine_biosynthesis_III_g__Anaerostipes_s__Anaerostipes_hadrus
PWY_2942__L_lysine_biosynthesis_III_g__Anaerotruncus_s__Anaerotruncus_colihominis
PWY_2942__L_lysine_biosynthesis_III_g__Bacteroides_s__Bacteroides_cellulosilyticus
PWY_2942__L_lysine_biosynthesis_III_g__Bacteroides_s__Bacteroides_clarus
PWY_2942__L_lysine_biosynthesis_III_g__Bacteroides_s__Bacteroides_coprocola
PWY_2942__L_lysine_biosynthesis_III_g__Bacteroides_s__Bacteroides_dorei
PWY_2942__L_lysine_biosynthesis_III_g__Bacteroides_s__Bacteroides_eggerthii
PWY_2942__L_lysine_biosynthesis_III_g__Bacteroides_s__Bacteroides_faecis
PWY_2942__L_lysine_biosynthesis_III_g__Bacteroides_s__Bacteroides_finegoldii
PWY_2942__L_lysine_biosynthesis_III_g__Bacteroides_s__Bacteroides_intestinalis
PWY_2942__L_lysine_biosynthesis_III_g__Bacteroides_s__Bacteroides_massiliensis
PWY_2942__L_lysine_biosynthesis_III_g__Bacteroides_s__Bacteroides_ovatus
PWY_2942__L_lysine_biosynthesis_III_g__Bacteroides_s__Bacteroides_plebeius
PWY_2942__L_lysine_biosynthesis_III_g__Bacteroides_s__Bacteroides_salyersiae
PWY_2942__L_lysine_biosynthesis_III_g__Bacteroides_s__Bacteroides_sp_1_1_30
PWY_2942__L_lysine_biosynthesis_III_g__Bacteroides_s__Bacteroides_sp_1_1_6
PWY_2942__L_lysine_biosynthesis_III_g__Bacteroides_s__Bacteroides_sp_2_1_22
PWY_2942__L_lysine_biosynthesis_III_g__Bacteroides_s__Bacteroides_sp_2_2_4
PWY_2942__L_lysine_biosynthesis_III_g__Bacteroides_s__Bacteroides_sp_3_1_19
PWY_2942__L_lysine_biosynthesis_III_g__Bacteroides_s__Bacteroides_sp_3_1_23

PWY_2942__L_lysine_biosynthesis_III_g__Bacteroides_s__Bacteroides_sp_3_1_33FAA
PWY_2942__L_lysine_biosynthesis_III_g__Bacteroides_s__Bacteroides_sp_3_1_40A
PWY_2942__L_lysine_biosynthesis_III_g__Bacteroides_s__Bacteroides_sp_4_3_47FAA
PWY_2942__L_lysine_biosynthesis_III_g__Bacteroides_s__Bacteroides_sp_9_1_42FAA
PWY_2942__L_lysine_biosynthesis_III_g__Bacteroides_s__Bacteroides_sp_D20
PWY_2942__L_lysine_biosynthesis_III_g__Bacteroides_s__Bacteroides_stercoris
PWY_2942__L_lysine_biosynthesis_III_g__Bacteroides_s__Bacteroides_thetaiotaomicron
PWY_2942__L_lysine_biosynthesis_III_g__Bacteroides_s__Bacteroides_uniformis
PWY_2942__L_lysine_biosynthesis_III_g__Bacteroides_s__Bacteroides_xylanisolvens
PWY_2942__L_lysine_biosynthesis_III_g__Barnesiella_s__Barnesiella_intestinihominis
PWY_2942__L_lysine_biosynthesis_III_g__Bifidobacterium_s__Bifidobacterium_animalis
PWY_2942__L_lysine_biosynthesis_III_g__Bilophila_s__Bilophila_wadsworthia
PWY_2942__L_lysine_biosynthesis_III_g__Blautia_s__Ruminococcus_gnavus
PWY_2942__L_lysine_biosynthesis_III_g__Blautia_s__Ruminococcus_obeum
PWY_2942__L_lysine_biosynthesis_III_g__Burkholderiales_noname_s__Burkholderiales_bacteriun
PWY_2942__L_lysine_biosynthesis_III_g__Catenibacterium_s__Catenibacterium_mitsuokai
PWY_2942__L_lysine_biosynthesis_III_g__Citrobacter_s__Citrobacter_freundii
PWY_2942__L_lysine_biosynthesis_III_g__Clostridium_s__Clostridium_asparagiforme
PWY_2942__L_lysine_biosynthesis_III_g__Clostridium_s__Clostridium_hathewayi
PWY_2942__L_lysine_biosynthesis_III_g__Clostridium_s__Clostridium_sp_SS2_1
PWY_2942__L_lysine_biosynthesis_III_g__Coprobacter_s__Coprobacter_fastidiosus
PWY_2942__L_lysine_biosynthesis_III_g__Coprococcus_s__Coprococcus_catus
PWY_2942__L_lysine_biosynthesis_III_g__Coprococcus_s__Coprococcus_eutactus
PWY_2942__L_lysine_biosynthesis_III_g__Coprococcus_s__Coprococcus_sp_ART55_1
PWY_2942__L_lysine_biosynthesis_III_g__Dialister_s__Dialister_invisus
PWY_2942__L_lysine_biosynthesis_III_g__Eggerthella_s__Eggerthella_lenta
PWY_2942__L_lysine_biosynthesis_III_g__Eggerthella_s__Eggerthella_sp_1_3_56FAA
PWY_2942__L_lysine_biosynthesis_III_g__Eggerthella_s__Eggerthella_sp_HGA1
PWY_2942__L_lysine_biosynthesis_III_g__Enterococcus_s__Enterococcus_durans
PWY_2942__L_lysine_biosynthesis_III_g__Enterorhabdus_s__Enterorhabdus_caecimuris
PWY_2942__L_lysine_biosynthesis_III_g__Erysipelotrichaceae_noname_s__Clostridium_spiroform
PWY_2942__L_lysine_biosynthesis_III_g__Erysipelotrichaceae_noname_s__Erysipelotrichaceae_ba
PWY_2942__L_lysine_biosynthesis_III_g__Eubacterium_s__Eubacterium_siraeum
PWY_2942__L_lysine_biosynthesis_III_g__Eubacterium_s__Eubacterium_sp_3_1_31
PWY_2942__L_lysine_biosynthesis_III_g__Faecalibacterium_s__Faecalibacterium_prausnitzii
PWY_2942__L_lysine_biosynthesis_III_g__Gemella_s__Gemella_sanguinis
PWY_2942__L_lysine_biosynthesis_III_g__Gordonibacter_s__Gordonibacter_pamelaeae
PWY_2942__L_lysine_biosynthesis_III_g__Lachnospiraceae_noname_s__Lachnospiraceae_bacteriu
PWY_2942__L_lysine_biosynthesis_III_g__Lachnospiraceae_noname_s__Lachnospiraceae_bacteriu
PWY_2942__L_lysine_biosynthesis_III_g__Lachnospiraceae_noname_s__Lachnospiraceae_bacteriu
PWY_2942__L_lysine_biosynthesis_III_g__Lachnospiraceae_noname_s__Lachnospiraceae_bacteriu
PWY_2942__L_lysine_biosynthesis_III_g__Lactobacillus_s__Lactobacillus_casei_paracasei
PWY_2942__L_lysine_biosynthesis_III_g__Lactobacillus_s__Lactobacillus_fermentum
PWY_2942__L_lysine_biosynthesis_III_g__Lactococcus_s__Lactococcus_lactis
PWY_2942__L_lysine_biosynthesis_III_g__Odoribacter_s__Odoribacter_laneus
PWY_2942__L_lysine_biosynthesis_III_g__Odoribacter_s__Odoribacter_splanchnicus
PWY_2942__L_lysine_biosynthesis_III_g__Oxalobacter_s__Oxalobacter_formigenes

PWY_2942__L_lysine_biosynthesis_III_g__Parabacteroides_s__Parabacteroides_distasonis
PWY_2942__L_lysine_biosynthesis_III_g__Parabacteroides_s__Parabacteroides_goldsteinii
PWY_2942__L_lysine_biosynthesis_III_g__Parabacteroides_s__Parabacteroides_johnsonii
PWY_2942__L_lysine_biosynthesis_III_g__Parabacteroides_s__Parabacteroides_merdae
PWY_2942__L_lysine_biosynthesis_III_g__Parabacteroides_s__Parabacteroides_sp_20_3
PWY_2942__L_lysine_biosynthesis_III_g__Parabacteroides_s__Parabacteroides_sp_D13
PWY_2942__L_lysine_biosynthesis_III_g__Paraprevotella_s__Paraprevotella_clara
PWY_2942__L_lysine_biosynthesis_III_g__Paraprevotella_s__Paraprevotella_xylaniphila
PWY_2942__L_lysine_biosynthesis_III_g__Peptostreptococcaceae_noname_s__Clostridium_bartle
PWY_2942__L_lysine_biosynthesis_III_g__Prevotella_s__Prevotella_copri
PWY_2942__L_lysine_biosynthesis_III_g__Propionibacterium_s__Propionibacterium_freudenreich
PWY_2942__L_lysine_biosynthesis_III_g__Ruminococcus_s__Ruminococcus_champanellensis
PWY_2942__L_lysine_biosynthesis_III_g__Ruminococcus_s__Ruminococcus_lactaris
PWY_2942__L_lysine_biosynthesis_III_g__Veillonella_s__Veillonella_dispar
PWY_2942__L_lysine_biosynthesis_III_g__Veillonella_s__Veillonella_parvula
PWY_3001__superpathway_of_L_isoleucine_biosynthesis_I_g__Citrobacter_s__Citrobacter_freund
PWY_3001__superpathway_of_L_isoleucine_biosynthesis_I_g__Coprococcus_s__Coprococcus_cat
PWY_3001__superpathway_of_L_isoleucine_biosynthesis_I_g__Coprococcus_s__Coprococcus_eut
PWY_3001__superpathway_of_L_isoleucine_biosynthesis_I_g__Coprococcus_s__Coprococcus_sp_
PWY_3001__superpathway_of_L_isoleucine_biosynthesis_I_g__Enterobacter_s__Enterobacter_clo
PWY_3001__superpathway_of_L_isoleucine_biosynthesis_I_g__Lactococcus_s__Lactococcus_lactis
PWY_3001__superpathway_of_L_isoleucine_biosynthesis_I_g__Propionibacterium_s__Propioniba
PWY_3001__superpathway_of_L_isoleucine_biosynthesis_I_g__Streptococcus_s__Streptococcus_
PWY_3781__aerobic_respiration_I_(cytochrome_c)_g__Nakaseomyces_s__Candida_glabrata
PWY_3801__sucrose_degradation_II_(sucrose_synthase)
PWY_3801__sucrose_degradation_II_(sucrose_synthase)_unclassified
PWY_3841__folate_transformations_II_g__Akkermansia_s__Akkermansia_muciniphila
PWY_3841__folate_transformations_II_g__Bacteroides_s__Bacteroides_cellulosilyticus
PWY_3841__folate_transformations_II_g__Bacteroides_s__Bacteroides_clarus
PWY_3841__folate_transformations_II_g__Bacteroides_s__Bacteroides_coprocola
PWY_3841__folate_transformations_II_g__Bacteroides_s__Bacteroides_dorei
PWY_3841__folate_transformations_II_g__Bacteroides_s__Bacteroides_faecis
PWY_3841__folate_transformations_II_g__Bacteroides_s__Bacteroides_finegoldii
PWY_3841__folate_transformations_II_g__Bacteroides_s__Bacteroides_fragilis
PWY_3841__folate_transformations_II_g__Bacteroides_s__Bacteroides_intestinalis
PWY_3841__folate_transformations_II_g__Bacteroides_s__Bacteroides_massiliensis
PWY_3841__folate_transformations_II_g__Bacteroides_s__Bacteroides_ovatus
PWY_3841__folate_transformations_II_g__Bacteroides_s__Bacteroides_sp_1_1_30
PWY_3841__folate_transformations_II_g__Bacteroides_s__Bacteroides_sp_1_1_6
PWY_3841__folate_transformations_II_g__Bacteroides_s__Bacteroides_sp_2_1_22
PWY_3841__folate_transformations_II_g__Bacteroides_s__Bacteroides_sp_2_2_4
PWY_3841__folate_transformations_II_g__Bacteroides_s__Bacteroides_sp_3_1_19
PWY_3841__folate_transformations_II_g__Bacteroides_s__Bacteroides_sp_3_1_23
PWY_3841__folate_transformations_II_g__Bacteroides_s__Bacteroides_sp_3_1_33FAA
PWY_3841__folate_transformations_II_g__Bacteroides_s__Bacteroides_sp_3_1_40A
PWY_3841__folate_transformations_II_g__Bacteroides_s__Bacteroides_sp_4_3_47FAA
PWY_3841__folate_transformations_II_g__Bacteroides_s__Bacteroides_sp_9_1_42FAA

PWY_3841__folate_transformations_II_g__Bacteroides_s__Bacteroides_sp_D20
PWY_3841__folate_transformations_II_g__Bacteroides_s__Bacteroides_stercoris
PWY_3841__folate_transformations_II_g__Bacteroides_s__Bacteroides_thetaiotaomicron
PWY_3841__folate_transformations_II_g__Bacteroides_s__Bacteroides_uniformis
PWY_3841__folate_transformations_II_g__Bacteroides_s__Bacteroides_xylanisolvens
PWY_3841__folate_transformations_II_g__Citrobacter_s__Citrobacter_freundii
PWY_3841__folate_transformations_II_g__Enterobacter_s__Enterobacter_cloacae
PWY_3841__folate_transformations_II_g__Enterococcus_s__Enterococcus_faecalis
PWY_3841__folate_transformations_II_g__Enterococcus_s__Enterococcus_faecium
PWY_3841__folate_transformations_II_g__Klebsiella_s__Klebsiella_pneumoniae
PWY_3841__folate_transformations_II_g__Lactobacillus_s__Lactobacillus_brevis
PWY_3841__folate_transformations_II_g__Lactobacillus_s__Lactobacillus_casei_paracasei
PWY_3841__folate_transformations_II_g__Lactobacillus_s__Lactobacillus_fermentum
PWY_3841__folate_transformations_II_g__Lactococcus_s__Lactococcus_lactis
PWY_3841__folate_transformations_II_g__Parabacteroides_s__Parabacteroides_distasonis
PWY_3841__folate_transformations_II_g__Parabacteroides_s__Parabacteroides_goldsteinii
PWY_3841__folate_transformations_II_g__Parabacteroides_s__Parabacteroides_johnsonii
PWY_3841__folate_transformations_II_g__Parabacteroides_s__Parabacteroides_merdae
PWY_3841__folate_transformations_II_g__Parabacteroides_s__Parabacteroides_sp_20_3
PWY_3841__folate_transformations_II_g__Parabacteroides_s__Parabacteroides_sp_D13
PWY_3841__folate_transformations_II_g__Streptococcus_s__Streptococcus_mitis_oralis_pneumo
PWY_3841__folate_transformations_II_g__Streptococcus_s__Streptococcus_sanguinis
PWY_4041__&gamma__glutamyl_cycle_g__Citrobacter_s__Citrobacter_freundii
PWY_4041__&gamma__glutamyl_cycle_g__Klebsiella_s__Klebsiella_pneumoniae
PWY_4242__pantothenate_and_coenzyme_A_biosynthesis_III_g__Actinomyces_s__Actinomyces_
PWY_4242__pantothenate_and_coenzyme_A_biosynthesis_III_g__Akkermansia_s__Akkermansia_
PWY_4242__pantothenate_and_coenzyme_A_biosynthesis_III_g__Anaerofustis_s__Anaerofustis_
PWY_4242__pantothenate_and_coenzyme_A_biosynthesis_III_g__Anaerotruncus_s__Anaerotrunc
PWY_4242__pantothenate_and_coenzyme_A_biosynthesis_III_g__Bacteroides_s__Bacteroides_ov
PWY_4242__pantothenate_and_coenzyme_A_biosynthesis_III_g__Bacteroides_s__Bacteroides_sp
PWY_4242__pantothenate_and_coenzyme_A_biosynthesis_III_g__Bacteroides_s__Bacteroides_sp
PWY_4242__pantothenate_and_coenzyme_A_biosynthesis_III_g__Bacteroides_s__Bacteroides_sp
PWY_4242__pantothenate_and_coenzyme_A_biosynthesis_III_g__Bacteroides_s__Bacteroides_sp
PWY_4242__pantothenate_and_coenzyme_A_biosynthesis_III_g__Bacteroides_s__Bacteroides_xy
PWY_4242__pantothenate_and_coenzyme_A_biosynthesis_III_g__Citrobacter_s__Citrobacter_fre
PWY_4242__pantothenate_and_coenzyme_A_biosynthesis_III_g__Clostridiales_noname_s__Clost
PWY_4242__pantothenate_and_coenzyme_A_biosynthesis_III_g__Eggerthella_s__Eggerthella_len
PWY_4242__pantothenate_and_coenzyme_A_biosynthesis_III_g__Eggerthella_s__Eggerthella_sp_
PWY_4242__pantothenate_and_coenzyme_A_biosynthesis_III_g__Eggerthella_s__Eggerthella_sp_
PWY_4242__pantothenate_and_coenzyme_A_biosynthesis_III_g__Erysipelotrichaceae_noname_s
PWY_4242__pantothenate_and_coenzyme_A_biosynthesis_III_g__Eubacterium_s__Eubacterium_
PWY_4242__pantothenate_and_coenzyme_A_biosynthesis_III_g__Lactobacillus_s__Lactobacillus_
PWY_4242__pantothenate_and_coenzyme_A_biosynthesis_III_g__Lactococcus_s__Lactococcus_la
PWY_4242__pantothenate_and_coenzyme_A_biosynthesis_III_g__Oxalobacter_s__Oxalobacter_fo
PWY_4242__pantothenate_and_coenzyme_A_biosynthesis_III_g__Pediococcus_s__Pediococcus_a
PWY_4242__pantothenate_and_coenzyme_A_biosynthesis_III_g__Pediococcus_s__Pediococcus_lo
PWY_4242__pantothenate_and_coenzyme_A_biosynthesis_III_g__Phascolarctobacterium_s__Pha

PWY_4242__pantothenate_and_coenzyme_A_biosynthesis_III_g__Streptococcus_s__Streptococcu
PWY_4242__pantothenate_and_coenzyme_A_biosynthesis_III_g__Streptococcus_s__Streptococcu
PWY_4242__pantothenate_and_coenzyme_A_biosynthesis_III_g__Veillonella_s__Veillonella_dispa
PWY_4242__pantothenate_and_coenzyme_A_biosynthesis_III_g__Veillonella_s__Veillonella_parv
PWY_4321__L_glutamate_degradation_IV
PWY_4321__L_glutamate_degradation_IV_unclassified
PWY_4361__S_methyl_5_thio_&alpha__D_ribose_1_phosphate_degradation
PWY_4361__S_methyl_5_thio_&alpha__D_ribose_1_phosphate_degradation_unclassified
PWY_4702__phytate_degradation_I_g__Akkermansia_s__Akkermansia_muciniphila
PWY_4702__phytate_degradation_I_unclassified
PWY_4981__L_proline_biosynthesis_II_(from_arginine)_g__Adlercreutzia_s__Adlercreutzia_equol
PWY_4981__L_proline_biosynthesis_II_(from_arginine)_g__Anaerofustis_s__Anaerofustis_stercor
PWY_4981__L_proline_biosynthesis_II_(from_arginine)_g__Clostridiales_noname_s__Clostridiales
PWY_4981__L_proline_biosynthesis_II_(from_arginine)_g__Clostridium_s__Clostridium_asparagif
PWY_4981__L_proline_biosynthesis_II_(from_arginine)_g__Clostridium_s__Clostridium_citroniae
PWY_4981__L_proline_biosynthesis_II_(from_arginine)_g__Clostridium_s__Clostridium_perfringe
PWY_4981__L_proline_biosynthesis_II_(from_arginine)_g__Collinsella_s__Collinsella_tanakaei
PWY_4981__L_proline_biosynthesis_II_(from_arginine)_g__Eggerthella_s__Eggerthella_lenta
PWY_4981__L_proline_biosynthesis_II_(from_arginine)_g__Eggerthella_s__Eggerthella_sp_1_3_5
PWY_4981__L_proline_biosynthesis_II_(from_arginine)_g__Eggerthella_s__Eggerthella_sp_HGA1
PWY_4981__L_proline_biosynthesis_II_(from_arginine)_g__Enterobacter_s__Enterobacter_cloaca
PWY_4981__L_proline_biosynthesis_II_(from_arginine)_g__Enterococcus_s__Enterococcus_faeciu
PWY_4981__L_proline_biosynthesis_II_(from_arginine)_g__Enterorhabdus_s__Enterorhabdus_cae
PWY_4981__L_proline_biosynthesis_II_(from_arginine)_g__Escherichia_s__Escherichia_coli
PWY_4981__L_proline_biosynthesis_II_(from_arginine)_g__Eubacterium_s__Eubacterium_ramulu
PWY_4981__L_proline_biosynthesis_II_(from_arginine)_g__Lactobacillus_s__Lactobacillus_brevis
PWY_4981__L_proline_biosynthesis_II_(from_arginine)_g__Lactobacillus_s__Lactobacillus_fermer
PWY_4981__L_proline_biosynthesis_II_(from_arginine)_g__Odoribacter_s__Odoribacter_splanchr
PWY_4981__L_proline_biosynthesis_II_(from_arginine)_g__Pediococcus_s__Pediococcus_acidilact
PWY_4981__L_proline_biosynthesis_II_(from_arginine)_g__Pediococcus_s__Pediococcus_lolii
PWY_4981__L_proline_biosynthesis_II_(from_arginine)_g__Slackia_s__Slackia_piriformis
PWY_4984__urea_cycle_g__Coprococcus_s__Coprococcus_catus
PWY_4984__urea_cycle_g__Lachnospiraceae_noname_s__Lachnospiraceae_bacterium_7_1_58FA
PWY_5004__superpathway_of_L_citrulline_metabolism
PWY_5005__biotin_biosynthesis_II_g__Veillonella_s__Veillonella_dispar
PWY_5005__biotin_biosynthesis_II_g__Veillonella_s__Veillonella_parvula
PWY_5022__4_aminobutanoate_degradation_V_g__Klebsiella_s__Klebsiella_pneumoniae
PWY_5022__4_aminobutanoate_degradation_V_unclassified
PWY_5083__NAD_NADH_phosphorylation_and_dephosphorylation_g__Citrobacter_s__Citrobacte
PWY_5083__NAD_NADH_phosphorylation_and_dephosphorylation_g__Enterobacter_s__Enteroba
PWY_5083__NAD_NADH_phosphorylation_and_dephosphorylation_g__Klebsiella_s__Klebsiella_p
PWY_5088__L_glutamate_degradation_VIII_(to_propanoate)
PWY_5097__L_lysine_biosynthesis_VI_g__Adlercreutzia_s__Adlercreutzia_equolifaciens
PWY_5097__L_lysine_biosynthesis_VI_g__Akkermansia_s__Akkermansia_muciniphila
PWY_5097__L_lysine_biosynthesis_VI_g__Alistipes_s__Alistipes_indistinctus
PWY_5097__L_lysine_biosynthesis_VI_g__Anaerostipes_s__Anaerostipes_hadrus
PWY_5097__L_lysine_biosynthesis_VI_g__Anaerotruncus_s__Anaerotruncus_colihominis

PWY_5097__L_lysine_biosynthesis_VI_g__Bacteroides_s__Bacteroides_cellulosilyticus
PWY_5097__L_lysine_biosynthesis_VI_g__Bacteroides_s__Bacteroides_clarus
PWY_5097__L_lysine_biosynthesis_VI_g__Bacteroides_s__Bacteroides_coprocola
PWY_5097__L_lysine_biosynthesis_VI_g__Bacteroides_s__Bacteroides_eggerthii
PWY_5097__L_lysine_biosynthesis_VI_g__Bacteroides_s__Bacteroides_faecis
PWY_5097__L_lysine_biosynthesis_VI_g__Bacteroides_s__Bacteroides_finegoldii
PWY_5097__L_lysine_biosynthesis_VI_g__Bacteroides_s__Bacteroides_intestinalis
PWY_5097__L_lysine_biosynthesis_VI_g__Bacteroides_s__Bacteroides_massiliensis
PWY_5097__L_lysine_biosynthesis_VI_g__Bacteroides_s__Bacteroides_ovatus
PWY_5097__L_lysine_biosynthesis_VI_g__Bacteroides_s__Bacteroides_plebeius
PWY_5097__L_lysine_biosynthesis_VI_g__Bacteroides_s__Bacteroides_salyersiae
PWY_5097__L_lysine_biosynthesis_VI_g__Bacteroides_s__Bacteroides_sp_1_1_30
PWY_5097__L_lysine_biosynthesis_VI_g__Bacteroides_s__Bacteroides_sp_1_1_6
PWY_5097__L_lysine_biosynthesis_VI_g__Bacteroides_s__Bacteroides_sp_2_1_22
PWY_5097__L_lysine_biosynthesis_VI_g__Bacteroides_s__Bacteroides_sp_2_2_4
PWY_5097__L_lysine_biosynthesis_VI_g__Bacteroides_s__Bacteroides_sp_3_1_19
PWY_5097__L_lysine_biosynthesis_VI_g__Bacteroides_s__Bacteroides_sp_3_1_23
PWY_5097__L_lysine_biosynthesis_VI_g__Bacteroides_s__Bacteroides_sp_3_1_40A
PWY_5097__L_lysine_biosynthesis_VI_g__Bacteroides_s__Bacteroides_sp_4_3_47FAA
PWY_5097__L_lysine_biosynthesis_VI_g__Bacteroides_s__Bacteroides_sp_D20
PWY_5097__L_lysine_biosynthesis_VI_g__Bacteroides_s__Bacteroides_stercoris
PWY_5097__L_lysine_biosynthesis_VI_g__Bacteroides_s__Bacteroides_thetaiotaomicron
PWY_5097__L_lysine_biosynthesis_VI_g__Bacteroides_s__Bacteroides_uniformis
PWY_5097__L_lysine_biosynthesis_VI_g__Bacteroides_s__Bacteroides_xylanisolvens
PWY_5097__L_lysine_biosynthesis_VI_g__Barnesiella_s__Barnesiella_intestinihominis
PWY_5097__L_lysine_biosynthesis_VI_g__Bifidobacterium_s__Bifidobacterium_animalis
PWY_5097__L_lysine_biosynthesis_VI_g__Bilophila_s__Bilophila_wadsworthia
PWY_5097__L_lysine_biosynthesis_VI_g__Blautia_s__Ruminococcus_gnavus
PWY_5097__L_lysine_biosynthesis_VI_g__Blautia_s__Ruminococcus_obeum
PWY_5097__L_lysine_biosynthesis_VI_g__Burkholderiales_noname_s__Burkholderiales_bacteriu
PWY_5097__L_lysine_biosynthesis_VI_g__Catenibacterium_s__Catenibacterium_mitsuokai
PWY_5097__L_lysine_biosynthesis_VI_g__Citrobacter_s__Citrobacter_freundii
PWY_5097__L_lysine_biosynthesis_VI_g__Clostridium_s__Clostridium_asparagiforme
PWY_5097__L_lysine_biosynthesis_VI_g__Clostridium_s__Clostridium_hathewayi
PWY_5097__L_lysine_biosynthesis_VI_g__Clostridium_s__Clostridium_sp_SS2_1
PWY_5097__L_lysine_biosynthesis_VI_g__Coprobacter_s__Coprobacter_fastidiosus
PWY_5097__L_lysine_biosynthesis_VI_g__Coprococcus_s__Coprococcus_catus
PWY_5097__L_lysine_biosynthesis_VI_g__Coprococcus_s__Coprococcus_eutactus
PWY_5097__L_lysine_biosynthesis_VI_g__Coprococcus_s__Coprococcus_sp_ART55_1
PWY_5097__L_lysine_biosynthesis_VI_g__Dialister_s__Dialister_invisus
PWY_5097__L_lysine_biosynthesis_VI_g__Enterococcus_s__Enterococcus_durans
PWY_5097__L_lysine_biosynthesis_VI_g__Enterorhabdus_s__Enterorhabdus_caecimuris
PWY_5097__L_lysine_biosynthesis_VI_g__Erysipelotrichaceae_noname_s__Clostridium_spiroform
PWY_5097__L_lysine_biosynthesis_VI_g__Erysipelotrichaceae_noname_s__Erysipelotrichaceae_ba
PWY_5097__L_lysine_biosynthesis_VI_g__Eubacterium_s__Eubacterium_siraeum
PWY_5097__L_lysine_biosynthesis_VI_g__Gordonibacter_s__Gordonibacter_pamelaeae
PWY_5097__L_lysine_biosynthesis_VI_g__Lachnospiraceae_noname_s__Lachnospiraceae_bacteri

PWY_5097__L_lysine_biosynthesis_VI_g__Lachnospiraceae_noname_s__Lachnospiraceae_bacteriu
PWY_5097__L_lysine_biosynthesis_VI_g__Lachnospiraceae_noname_s__Lachnospiraceae_bacteriu
PWY_5097__L_lysine_biosynthesis_VI_g__Lactobacillus_s__Lactobacillus_casei_paracasei
PWY_5097__L_lysine_biosynthesis_VI_g__Lactobacillus_s__Lactobacillus_fermentum
PWY_5097__L_lysine_biosynthesis_VI_g__Odoribacter_s__Odoribacter_laneus
PWY_5097__L_lysine_biosynthesis_VI_g__Odoribacter_s__Odoribacter_splanchnicus
PWY_5097__L_lysine_biosynthesis_VI_g__Oxalobacter_s__Oxalobacter_formigenes
PWY_5097__L_lysine_biosynthesis_VI_g__Parabacteroides_s__Parabacteroides_distasonis
PWY_5097__L_lysine_biosynthesis_VI_g__Parabacteroides_s__Parabacteroides_goldsteinii
PWY_5097__L_lysine_biosynthesis_VI_g__Parabacteroides_s__Parabacteroides_johnsonii
PWY_5097__L_lysine_biosynthesis_VI_g__Parabacteroides_s__Parabacteroides_merdae
PWY_5097__L_lysine_biosynthesis_VI_g__Parabacteroides_s__Parabacteroides_sp_20_3
PWY_5097__L_lysine_biosynthesis_VI_g__Parabacteroides_s__Parabacteroides_sp_D13
PWY_5097__L_lysine_biosynthesis_VI_g__Paraprevotella_s__Paraprevotella_clara
PWY_5097__L_lysine_biosynthesis_VI_g__Paraprevotella_s__Paraprevotella_xylaniphila
PWY_5097__L_lysine_biosynthesis_VI_g__Peptostreptococcaceae_noname_s__Clostridium_bartle
PWY_5097__L_lysine_biosynthesis_VI_g__Phascolarctobacterium_s__Phascolarctobacterium_succ
PWY_5097__L_lysine_biosynthesis_VI_g__Prevotella_s__Prevotella_copri
PWY_5097__L_lysine_biosynthesis_VI_g__Roseburia_s__Roseburia_hominis
PWY_5097__L_lysine_biosynthesis_VI_g__Ruminococcus_s__Ruminococcus_callidus
PWY_5097__L_lysine_biosynthesis_VI_g__Ruminococcus_s__Ruminococcus_champanellensis
PWY_5097__L_lysine_biosynthesis_VI_g__Ruminococcus_s__Ruminococcus_lactaris
PWY_5097__L_lysine_biosynthesis_VI_g__Veillonella_s__Veillonella_dispar
PWY_5097__L_lysine_biosynthesis_VI_g__Veillonella_s__Veillonella_parvula
PWY_5100__pyruvate_fermentation_to_acetate_and_lactate_II_g__Anaerostipes_s__Anaerostipe
PWY_5100__pyruvate_fermentation_to_acetate_and_lactate_II_g__Bifidobacterium_s__Bifidobac
PWY_5100__pyruvate_fermentation_to_acetate_and_lactate_II_g__Clostridium_s__Clostridium_s
PWY_5100__pyruvate_fermentation_to_acetate_and_lactate_II_g__Clostridium_s__Clostridium_s
PWY_5100__pyruvate_fermentation_to_acetate_and_lactate_II_g__Collinsella_s__Collinsella_inte
PWY_5100__pyruvate_fermentation_to_acetate_and_lactate_II_g__Collinsella_s__Collinsella_tan
PWY_5100__pyruvate_fermentation_to_acetate_and_lactate_II_g__Coprococcus_s__Coprococcus
PWY_5100__pyruvate_fermentation_to_acetate_and_lactate_II_g__Coprococcus_s__Coprococcus
PWY_5100__pyruvate_fermentation_to_acetate_and_lactate_II_g__Eggerthella_s__Eggerthella_le
PWY_5100__pyruvate_fermentation_to_acetate_and_lactate_II_g__Eggerthella_s__Eggerthella_sp
PWY_5100__pyruvate_fermentation_to_acetate_and_lactate_II_g__Eggerthella_s__Eggerthella_sp
PWY_5100__pyruvate_fermentation_to_acetate_and_lactate_II_g__Enterococcus_s__Enterococcu
PWY_5100__pyruvate_fermentation_to_acetate_and_lactate_II_g__Enterococcus_s__Enterococcu
PWY_5100__pyruvate_fermentation_to_acetate_and_lactate_II_g__Erysipelotrichaceae_noname_
PWY_5100__pyruvate_fermentation_to_acetate_and_lactate_II_g__Erysipelotrichaceae_noname_
PWY_5100__pyruvate_fermentation_to_acetate_and_lactate_II_g__Eubacterium_s__Eubacterium
PWY_5100__pyruvate_fermentation_to_acetate_and_lactate_II_g__Gemella_s__Gemella_sanguir
PWY_5100__pyruvate_fermentation_to_acetate_and_lactate_II_g__Klebsiella_s__Klebsiella_pneu
PWY_5100__pyruvate_fermentation_to_acetate_and_lactate_II_g__Lachnospiraceae_noname_s__
PWY_5100__pyruvate_fermentation_to_acetate_and_lactate_II_g__Propionibacterium_s__Propio
PWY_5100__pyruvate_fermentation_to_acetate_and_lactate_II_g__Roseburia_s__Roseburia_hom
PWY_5100__pyruvate_fermentation_to_acetate_and_lactate_II_g__Solobacterium_s__Solobacter
PWY_5100__pyruvate_fermentation_to_acetate_and_lactate_II_g__Streptococcus_s__Streptococ

PWY_5100__pyruvate_fermentation_to_acetate_and_lactate_II_g__Streptococcus_s__Streptococ
PWY_5100__pyruvate_fermentation_to_acetate_and_lactate_II_g__Streptococcus_s__Streptococ
PWY_5100__pyruvate_fermentation_to_acetate_and_lactate_II_g__Veillonella_s__Veillonella_dis
PWY_5103__L_isoleucine_biosynthesis_III_g__Akkermansia_s__Akkermansia_muciniphila
PWY_5103__L_isoleucine_biosynthesis_III_g__Anaerofustis_s__Anaerofustis_stercorihominis
PWY_5103__L_isoleucine_biosynthesis_III_g__Blautia_s__Blautia_sp_KLE_1732
PWY_5103__L_isoleucine_biosynthesis_III_g__Blautia_s__Ruminococcus_obeum
PWY_5103__L_isoleucine_biosynthesis_III_g__Citrobacter_s__Citrobacter_freundii
PWY_5103__L_isoleucine_biosynthesis_III_g__Clostridium_s__Clostridium_asparagiforme
PWY_5103__L_isoleucine_biosynthesis_III_g__Clostridium_s__Clostridium_leptum
PWY_5103__L_isoleucine_biosynthesis_III_g__Coprococcus_s__Coprococcus_catus
PWY_5103__L_isoleucine_biosynthesis_III_g__Coprococcus_s__Coprococcus_eutactus
PWY_5103__L_isoleucine_biosynthesis_III_g__Coprococcus_s__Coprococcus_sp_ART55_1
PWY_5103__L_isoleucine_biosynthesis_III_g__Eubacterium_s__Eubacterium_siraeum
PWY_5103__L_isoleucine_biosynthesis_III_g__Fusobacterium_s__Fusobacterium_nucleatum
PWY_5103__L_isoleucine_biosynthesis_III_g__Odoribacter_s__Odoribacter_laneus
PWY_5103__L_isoleucine_biosynthesis_III_g__Odoribacter_s__Odoribacter_splanchnicus
PWY_5103__L_isoleucine_biosynthesis_III_g__Propionibacterium_s__Propionibacterium_freudenr
PWY_5103__L_isoleucine_biosynthesis_III_g__Ruminococcus_s__Ruminococcus_sp
PWY_5103__L_isoleucine_biosynthesis_III_g__Streptococcus_s__Streptococcus_australis
PWY_5103__L_isoleucine_biosynthesis_III_g__Streptococcus_s__Streptococcus_mitis_oralis_pneu
PWY_5103__L_isoleucine_biosynthesis_III_g__Streptococcus_s__Streptococcus_sanguinis
PWY_5103__L_isoleucine_biosynthesis_III_g__Subdoligranulum_s__Subdoligranulum_sp_4_3_54A
PWY_5104__L_isoleucine_biosynthesis_IV_g__Anaerostipes_s__Anaerostipes_hadrus
PWY_5104__L_isoleucine_biosynthesis_IV_g__Clostridium_s__Clostridium_sp_SS2_1
PWY_5104__L_isoleucine_biosynthesis_IV_g__Coprococcus_s__Coprococcus_eutactus
PWY_5104__L_isoleucine_biosynthesis_IV_g__Coprococcus_s__Coprococcus_sp_ART55_1
PWY_5104__L_isoleucine_biosynthesis_IV_g__Lachnospiraceae_noname_s__Lachnospiraceae_bac
PWY_5104__L_isoleucine_biosynthesis_IV_g__Propionibacterium_s__Propionibacterium_freudenr
PWY_5136__fatty_acid_&beta__oxidation_II_(peroxisome)_g__Citrobacter_s__Citrobacter_freunc
PWY_5138__unsaturated,_even_numbered_fatty_acid_&beta__oxidation_g__Citrobacter_s__Citro
PWY_5138__unsaturated,_even_numbered_fatty_acid_&beta__oxidation_unclassified
PWY_5154__L_arginine_biosynthesis_III_(via_N_acetyl_L_citrulline)_g__Coprococcus_s__Coproco
PWY_5154__L_arginine_biosynthesis_III_(via_N_acetyl_L_citrulline)_g__Lactococcus_s__Lactococ
PWY_5173__superpathway_of_acetyl_CoA_biosynthesis_g__Citrobacter_s__Citrobacter_freundii
PWY_5173__superpathway_of_acetyl_CoA_biosynthesis_g__Enterobacter_s__Enterobacter_cloac
PWY_5173__superpathway_of_acetyl_CoA_biosynthesis_g__Klebsiella_s__Klebsiella_pneumoniae
PWY_5173__superpathway_of_acetyl_CoA_biosynthesis_g__Nakaseomyces_s__Candida_glabrata
PWY_5173__superpathway_of_acetyl_CoA_biosynthesis_g__Streptococcus_s__Streptococcus_san
PWY_5173__superpathway_of_acetyl_CoA_biosynthesis_unclassified
PWY_5177__glutaryl_CoA_degradation_g__Coprococcus_s__Coprococcus_catus
PWY_5180__toluene_degradation_I_(aerobic)_(via_o_cresol)
PWY_5182__toluene_degradation_II_(aerobic)_(via_4_methylcatechol)
PWY_5188__tetrapyrrole_biosynthesis_I_(from_glutamate)_g__Actinomyces_s__Actinomyces_gra
PWY_5188__tetrapyrrole_biosynthesis_I_(from_glutamate)_g__Akkermansia_s__Akkermansia_mu
PWY_5188__tetrapyrrole_biosynthesis_I_(from_glutamate)_g__Anaerostipes_s__Anaerostipes_ha
PWY_5188__tetrapyrrole_biosynthesis_I_(from_glutamate)_g__Anaerotruncus_s__Anaerotruncus

PWY_5188__tetrapyrrole_biosynthesis_I_(from_glutamate)_g__Bilophila_s__Bilophila_wadsworth
PWY_5188__tetrapyrrole_biosynthesis_I_(from_glutamate)_g__Blautia_s__Blautia_sp_KLE_1732
PWY_5188__tetrapyrrole_biosynthesis_I_(from_glutamate)_g__Blautia_s__Ruminococcus_obeum
PWY_5188__tetrapyrrole_biosynthesis_I_(from_glutamate)_g__Burkholderiales_noname_s__Burk
PWY_5188__tetrapyrrole_biosynthesis_I_(from_glutamate)_g__Citrobacter_s__Citrobacter_freund
PWY_5188__tetrapyrrole_biosynthesis_I_(from_glutamate)_g__Clostridium_s__Clostridium_aspar
PWY_5188__tetrapyrrole_biosynthesis_I_(from_glutamate)_g__Clostridium_s__Clostridium_perfri
PWY_5188__tetrapyrrole_biosynthesis_I_(from_glutamate)_g__Clostridium_s__Clostridium_sp_SS
PWY_5188__tetrapyrrole_biosynthesis_I_(from_glutamate)_g__Coprococcus_s__Coprococcus_cat
PWY_5188__tetrapyrrole_biosynthesis_I_(from_glutamate)_g__Coprococcus_s__Coprococcus_eut
PWY_5188__tetrapyrrole_biosynthesis_I_(from_glutamate)_g__Dialister_s__Dialister_invisus
PWY_5188__tetrapyrrole_biosynthesis_I_(from_glutamate)_g__Eggerthella_s__Eggerthella_lenta
PWY_5188__tetrapyrrole_biosynthesis_I_(from_glutamate)_g__Enterobacter_s__Enterobacter_clo
PWY_5188__tetrapyrrole_biosynthesis_I_(from_glutamate)_g__Eubacterium_s__Eubacterium_sp_
PWY_5188__tetrapyrrole_biosynthesis_I_(from_glutamate)_g__Fusobacterium_s__Fusobacterium
PWY_5188__tetrapyrrole_biosynthesis_I_(from_glutamate)_g__Klebsiella_s__Klebsiella_pneumor
PWY_5188__tetrapyrrole_biosynthesis_I_(from_glutamate)_g__Lachnospiraceae_noname_s__Lac
PWY_5188__tetrapyrrole_biosynthesis_I_(from_glutamate)_g__Lachnospiraceae_noname_s__Lac
PWY_5188__tetrapyrrole_biosynthesis_I_(from_glutamate)_g__Lachnospiraceae_noname_s__Lac
PWY_5188__tetrapyrrole_biosynthesis_I_(from_glutamate)_g__Parasutterella_s__Parasutterella_
PWY_5188__tetrapyrrole_biosynthesis_I_(from_glutamate)_g__Peptostreptococcaceae_noname_
PWY_5188__tetrapyrrole_biosynthesis_I_(from_glutamate)_g__Propionibacterium_s__Propioniba
PWY_5188__tetrapyrrole_biosynthesis_I_(from_glutamate)_g__Ruminococcus_s__Ruminococcus_
PWY_5188__tetrapyrrole_biosynthesis_I_(from_glutamate)_g__Ruminococcus_s__Ruminococcus_
PWY_5188__tetrapyrrole_biosynthesis_I_(from_glutamate)_g__Ruminococcus_s__Ruminococcus_
PWY_5188__tetrapyrrole_biosynthesis_I_(from_glutamate)_g__Veillonella_s__Veillonella_dispar
PWY_5188__tetrapyrrole_biosynthesis_I_(from_glutamate)_g__Veillonella_s__Veillonella_parvula
PWY_5189__tetrapyrrole_biosynthesis_II_(from_glycine)_g__Blautia_s__Blautia_sp_KLE_1732
PWY_5189__tetrapyrrole_biosynthesis_II_(from_glycine)_g__Blautia_s__Ruminococcus_obeum
PWY_5189__tetrapyrrole_biosynthesis_II_(from_glycine)_g__Citrobacter_s__Citrobacter_freundii
PWY_5189__tetrapyrrole_biosynthesis_II_(from_glycine)_g__Clostridium_s__Clostridium_asparag
PWY_5189__tetrapyrrole_biosynthesis_II_(from_glycine)_g__Enterobacter_s__Enterobacter_cloac
PWY_5189__tetrapyrrole_biosynthesis_II_(from_glycine)_g__Nakaseomyces_s__Candida_glabrata
PWY_5189__tetrapyrrole_biosynthesis_II_(from_glycine)_g__Propionibacterium_s__Propionibacte
PWY_5189__tetrapyrrole_biosynthesis_II_(from_glycine)_g__Ruminococcus_s__Ruminococcus_sp
PWY_5189__tetrapyrrole_biosynthesis_II_(from_glycine)_g__Streptococcus_s__Streptococcus_sar
PWY_5304__superpathway_of_sulfur_oxidation_(Acidianus_ambivalens)_g__Anaerostipes_s__An
PWY_5304__superpathway_of_sulfur_oxidation_(Acidianus_ambivalens)_g__Clostridium_s__Clos
PWY_5304__superpathway_of_sulfur_oxidation_(Acidianus_ambivalens)_g__Coprococcus_s__Cop
PWY_5304__superpathway_of_sulfur_oxidation_(Acidianus_ambivalens)_g__Coprococcus_s__Cop
PWY_5304__superpathway_of_sulfur_oxidation_(Acidianus_ambivalens)_g__Lachnospiraceae_no
PWY_5347__superpathway_of_L_methionine_biosynthesis_(transsulfuration)_g__Anaerostipes_s_
PWY_5347__superpathway_of_L_methionine_biosynthesis_(transsulfuration)_g__Clostridium_s__
PWY_5347__superpathway_of_L_methionine_biosynthesis_(transsulfuration)_g__Enterobacter_s_
PWY_5347__superpathway_of_L_methionine_biosynthesis_(transsulfuration)_g__Klebsiella_s__Kl
PWY_5347__superpathway_of_L_methionine_biosynthesis_(transsulfuration)_g__Lachnospiraceae
PWY_5347__superpathway_of_L_methionine_biosynthesis_(transsulfuration)_g__Lactococcus_s__

PWY_5367__petroselinate_biosynthesis_g__Lactococcus_s__Lactococcus_lactis
PWY_5415__catechol_degradation_I_(meta_cleavage_pathway)
PWY_5415__catechol_degradation_I_(meta_cleavage_pathway)_g__Klebsiella_s__Klebsiella_pneu
PWY_5484__glycolysis_II_(from_fructose_6_phosphate)_g__Citrobacter_s__Citrobacter_freundii
PWY_5484__glycolysis_II_(from_fructose_6_phosphate)_g__Enterobacter_s__Enterobacter_cloaca
PWY_5484__glycolysis_II_(from_fructose_6_phosphate)_g__Klebsiella_s__Klebsiella_pneumoniae
PWY_5484__glycolysis_II_(from_fructose_6_phosphate)_g__Lactococcus_s__Lactococcus_lactis
PWY_561__superpathway_of_glyoxylate_cycle_and_fatty_acid_degradation_g__Citrobacter_s__C
PWY_5656__mannosylglycerate_biosynthesis_I_unclassified
PWY_5659__GDP_mannose_biosynthesis_g__Alistipes_s__Alistipes_finegoldii
PWY_5659__GDP_mannose_biosynthesis_g__Alistipes_s__Alistipes_onderdonkii
PWY_5659__GDP_mannose_biosynthesis_g__Alistipes_s__Alistipes_shahii
PWY_5659__GDP_mannose_biosynthesis_g__Alistipes_s__Alistipes_sp_HGB5
PWY_5659__GDP_mannose_biosynthesis_g__Anaerostipes_s__Anaerostipes_hadrus
PWY_5659__GDP_mannose_biosynthesis_g__Bacteroides_s__Bacteroides_finegoldii
PWY_5659__GDP_mannose_biosynthesis_g__Bacteroides_s__Bacteroides_ovatus
PWY_5659__GDP_mannose_biosynthesis_g__Bacteroides_s__Bacteroides_sp_1_1_30
PWY_5659__GDP_mannose_biosynthesis_g__Bacteroides_s__Bacteroides_sp_2_1_22
PWY_5659__GDP_mannose_biosynthesis_g__Bacteroides_s__Bacteroides_sp_2_2_4
PWY_5659__GDP_mannose_biosynthesis_g__Bacteroides_s__Bacteroides_sp_3_1_23
PWY_5659__GDP_mannose_biosynthesis_g__Bacteroides_s__Bacteroides_xylanisolvens
PWY_5659__GDP_mannose_biosynthesis_g__Clostridium_s__Clostridium_sp_SS2_1
PWY_5659__GDP_mannose_biosynthesis_g__Coprococcus_s__Coprococcus_eutactus
PWY_5659__GDP_mannose_biosynthesis_g__Coprococcus_s__Coprococcus_sp_ART55_1
PWY_5659__GDP_mannose_biosynthesis_g__Eubacterium_s__Eubacterium_siraeum
PWY_5659__GDP_mannose_biosynthesis_g__Lachnospiraceae_noname_s__Lachnospiraceae_bact
PWY_5659__GDP_mannose_biosynthesis_g__Nakaseomyces_s__Candida_glabrata
PWY_5659__GDP_mannose_biosynthesis_g__Odoribacter_s__Odoribacter_splanchnicus
PWY_5659__GDP_mannose_biosynthesis_g__Propionibacterium_s__Propionibacterium_freudenre
PWY_5659__GDP_mannose_biosynthesis_g__Ruminococcus_s__Ruminococcus_sp
PWY_5667__CDP_diacylglycerol_biosynthesis_I_g__Alistipes_s__Alistipes_finegoldii
PWY_5667__CDP_diacylglycerol_biosynthesis_I_g__Alistipes_s__Alistipes_indistinctus
PWY_5667__CDP_diacylglycerol_biosynthesis_I_g__Alistipes_s__Alistipes_sp_HGB5
PWY_5667__CDP_diacylglycerol_biosynthesis_I_g__Anaerostipes_s__Anaerostipes_hadrus
PWY_5667__CDP_diacylglycerol_biosynthesis_I_g__Bacteroides_s__Bacteroides_cellulosilyticus
PWY_5667__CDP_diacylglycerol_biosynthesis_I_g__Bacteroides_s__Bacteroides_clarus
PWY_5667__CDP_diacylglycerol_biosynthesis_I_g__Bacteroides_s__Bacteroides_coprocola
PWY_5667__CDP_diacylglycerol_biosynthesis_I_g__Bacteroides_s__Bacteroides_eggerthii
PWY_5667__CDP_diacylglycerol_biosynthesis_I_g__Bacteroides_s__Bacteroides_faecis
PWY_5667__CDP_diacylglycerol_biosynthesis_I_g__Bacteroides_s__Bacteroides_finegoldii
PWY_5667__CDP_diacylglycerol_biosynthesis_I_g__Bacteroides_s__Bacteroides_intestinalis
PWY_5667__CDP_diacylglycerol_biosynthesis_I_g__Bacteroides_s__Bacteroides_ovatus
PWY_5667__CDP_diacylglycerol_biosynthesis_I_g__Bacteroides_s__Bacteroides_plebeius
PWY_5667__CDP_diacylglycerol_biosynthesis_I_g__Bacteroides_s__Bacteroides_salyersiae
PWY_5667__CDP_diacylglycerol_biosynthesis_I_g__Bacteroides_s__Bacteroides_sp_1_1_30
PWY_5667__CDP_diacylglycerol_biosynthesis_I_g__Bacteroides_s__Bacteroides_sp_1_1_6
PWY_5667__CDP_diacylglycerol_biosynthesis_I_g__Bacteroides_s__Bacteroides_sp_2_1_22

PWY_5667__CDP_diacylglycerol_biosynthesis_I_g__Bacteroides_s__Bacteroides_sp_2_2_4
PWY_5667__CDP_diacylglycerol_biosynthesis_I_g__Bacteroides_s__Bacteroides_sp_3_1_19
PWY_5667__CDP_diacylglycerol_biosynthesis_I_g__Bacteroides_s__Bacteroides_sp_3_1_23
PWY_5667__CDP_diacylglycerol_biosynthesis_I_g__Bacteroides_s__Bacteroides_sp_D20
PWY_5667__CDP_diacylglycerol_biosynthesis_I_g__Bacteroides_s__Bacteroides_stercoris
PWY_5667__CDP_diacylglycerol_biosynthesis_I_g__Bacteroides_s__Bacteroides_thetaiotaomicror
PWY_5667__CDP_diacylglycerol_biosynthesis_I_g__Bacteroides_s__Bacteroides_uniformis
PWY_5667__CDP_diacylglycerol_biosynthesis_I_g__Bacteroides_s__Bacteroides_xylanisolvens
PWY_5667__CDP_diacylglycerol_biosynthesis_I_g__Barnesiella_s__Barnesiella_intestinihominis
PWY_5667__CDP_diacylglycerol_biosynthesis_I_g__Blautia_s__Blautia_hydrogenotrophica
PWY_5667__CDP_diacylglycerol_biosynthesis_I_g__Blautia_s__Ruminococcus_gnavus
PWY_5667__CDP_diacylglycerol_biosynthesis_I_g__Blautia_s__Ruminococcus_obeum
PWY_5667__CDP_diacylglycerol_biosynthesis_I_g__Butyrivibrio_s__Butyrivibrio_crossotus
PWY_5667__CDP_diacylglycerol_biosynthesis_I_g__Citrobacter_s__Citrobacter_freundii
PWY_5667__CDP_diacylglycerol_biosynthesis_I_g__Clostridiales_noname_s__Clostridiales_bacter
PWY_5667__CDP_diacylglycerol_biosynthesis_I_g__Clostridium_s__Clostridium_asparagiforme
PWY_5667__CDP_diacylglycerol_biosynthesis_I_g__Clostridium_s__Clostridium_bolteae
PWY_5667__CDP_diacylglycerol_biosynthesis_I_g__Clostridium_s__Clostridium_clostridioforme
PWY_5667__CDP_diacylglycerol_biosynthesis_I_g__Clostridium_s__Clostridium_hathewayi
PWY_5667__CDP_diacylglycerol_biosynthesis_I_g__Clostridium_s__Clostridium_leptum
PWY_5667__CDP_diacylglycerol_biosynthesis_I_g__Clostridium_s__Clostridium_sp_L2_50
PWY_5667__CDP_diacylglycerol_biosynthesis_I_g__Clostridium_s__Clostridium_sp_SS2_1
PWY_5667__CDP_diacylglycerol_biosynthesis_I_g__Coprobacter_s__Coprobacter_fastidiosus
PWY_5667__CDP_diacylglycerol_biosynthesis_I_g__Coprococcus_s__Coprococcus_eutactus
PWY_5667__CDP_diacylglycerol_biosynthesis_I_g__Dialister_s__Dialister_invisus
PWY_5667__CDP_diacylglycerol_biosynthesis_I_g__Dorea_s__Dorea_formicigenerans
PWY_5667__CDP_diacylglycerol_biosynthesis_I_g__Dorea_s__Dorea_longicatena
PWY_5667__CDP_diacylglycerol_biosynthesis_I_g__Enterobacter_s__Enterobacter_cloacae
PWY_5667__CDP_diacylglycerol_biosynthesis_I_g__Erysipelotrichaceae_noname_s__Clostridium_
PWY_5667__CDP_diacylglycerol_biosynthesis_I_g__Eubacterium_s__Eubacterium_eligens
PWY_5667__CDP_diacylglycerol_biosynthesis_I_g__Eubacterium_s__Eubacterium_hallii
PWY_5667__CDP_diacylglycerol_biosynthesis_I_g__Eubacterium_s__Eubacterium_ramulus
PWY_5667__CDP_diacylglycerol_biosynthesis_I_g__Eubacterium_s__Eubacterium_siraeum
PWY_5667__CDP_diacylglycerol_biosynthesis_I_g__Eubacterium_s__Eubacterium_ventriosum
PWY_5667__CDP_diacylglycerol_biosynthesis_I_g__Gordonibacter_s__Gordonibacter_pamelaeae
PWY_5667__CDP_diacylglycerol_biosynthesis_I_g__Klebsiella_s__Klebsiella_pneumoniae
PWY_5667__CDP_diacylglycerol_biosynthesis_I_g__Lachnospiraceae_noname_s__Lachnospiracea
PWY_5667__CDP_diacylglycerol_biosynthesis_I_g__Lachnospiraceae_noname_s__Lachnospiracea
PWY_5667__CDP_diacylglycerol_biosynthesis_I_g__Lachnospiraceae_noname_s__Lachnospiracea
PWY_5667__CDP_diacylglycerol_biosynthesis_I_g__Lachnospiraceae_noname_s__Lachnospiracea
PWY_5667__CDP_diacylglycerol_biosynthesis_I_g__Lachnospiraceae_noname_s__Lachnospiracea
PWY_5667__CDP_diacylglycerol_biosynthesis_I_g__Lachnospiraceae_noname_s__Lachnospiracea
PWY_5667__CDP_diacylglycerol_biosynthesis_I_g__Lachnospiraceae_noname_s__Lachnospiracea
PWY_5667__CDP_diacylglycerol_biosynthesis_I_g__Lachnospiraceae_noname_s__Lachnospiracea
PWY_5667__CDP_diacylglycerol_biosynthesis_I_g__Oxalobacter_s__Oxalobacter_formigenes
PWY_5667__CDP_diacylglycerol_biosynthesis_I_g__Parabacteroides_s__Parabacteroides_distason
PWY_5667__CDP_diacylglycerol_biosynthesis_I_g__Parabacteroides_s__Parabacteroides_johnson

PWY_5667__CDP_diacylglycerol_biosynthesis_I_g__Parabacteroides_s__Parabacteroides_merdae
PWY_5667__CDP_diacylglycerol_biosynthesis_I_g__Parabacteroides_s__Parabacteroides_sp_D13
PWY_5667__CDP_diacylglycerol_biosynthesis_I_g__Paraprevotella_s__Paraprevotella_clara
PWY_5667__CDP_diacylglycerol_biosynthesis_I_g__Peptostreptococcaceae_noname_s__Clostridiu
PWY_5667__CDP_diacylglycerol_biosynthesis_I_g__Propionibacterium_s__Propionibacterium_freu
PWY_5667__CDP_diacylglycerol_biosynthesis_I_g__Roseburia_s__Roseburia_hominis
PWY_5667__CDP_diacylglycerol_biosynthesis_I_g__Roseburia_s__Roseburia_inulinivorans
PWY_5667__CDP_diacylglycerol_biosynthesis_I_g__Ruminococcus_s__Ruminococcus_champanell
PWY_5667__CDP_diacylglycerol_biosynthesis_I_g__Ruminococcus_s__Ruminococcus_sp
PWY_5667__CDP_diacylglycerol_biosynthesis_I_g__Streptococcus_s__Streptococcus_mitis_oralis_
PWY_5667__CDP_diacylglycerol_biosynthesis_I_g__Subdoligranulum_s__Subdoligranulum_sp_4_
PWY_5667__CDP_diacylglycerol_biosynthesis_I_g__Veillonella_s__Veillonella_dispar
PWY_5667__CDP_diacylglycerol_biosynthesis_I_g__Veillonella_s__Veillonella_parvula
PWY_5686__UMP_biosynthesis_g__Adlercreutzia_s__Adlercreutzia_equolifaciens
PWY_5686__UMP_biosynthesis_g__Akkermansia_s__Akkermansia_muciniphila
PWY_5686__UMP_biosynthesis_g__Alistipes_s__Alistipes_finegoldii
PWY_5686__UMP_biosynthesis_g__Alistipes_s__Alistipes_indistinctus
PWY_5686__UMP_biosynthesis_g__Alistipes_s__Alistipes_onderdonkii
PWY_5686__UMP_biosynthesis_g__Alistipes_s__Alistipes_putredinis
PWY_5686__UMP_biosynthesis_g__Alistipes_s__Alistipes_sp_HGB5
PWY_5686__UMP_biosynthesis_g__Anaerostipes_s__Anaerostipes_hadrus
PWY_5686__UMP_biosynthesis_g__Bacteroides_s__Bacteroides_cellulosilyticus
PWY_5686__UMP_biosynthesis_g__Bacteroides_s__Bacteroides_clarus
PWY_5686__UMP_biosynthesis_g__Bacteroides_s__Bacteroides_coprocola
PWY_5686__UMP_biosynthesis_g__Bacteroides_s__Bacteroides_eggerthii
PWY_5686__UMP_biosynthesis_g__Bacteroides_s__Bacteroides_faecis
PWY_5686__UMP_biosynthesis_g__Bacteroides_s__Bacteroides_finegoldii
PWY_5686__UMP_biosynthesis_g__Bacteroides_s__Bacteroides_intestinalis
PWY_5686__UMP_biosynthesis_g__Bacteroides_s__Bacteroides_massiliensis
PWY_5686__UMP_biosynthesis_g__Bacteroides_s__Bacteroides_ovatus
PWY_5686__UMP_biosynthesis_g__Bacteroides_s__Bacteroides_plebeius
PWY_5686__UMP_biosynthesis_g__Bacteroides_s__Bacteroides_sp_1_1_6
PWY_5686__UMP_biosynthesis_g__Bacteroides_s__Bacteroides_sp_2_2_4
PWY_5686__UMP_biosynthesis_g__Bacteroides_s__Bacteroides_sp_3_1_19
PWY_5686__UMP_biosynthesis_g__Bacteroides_s__Bacteroides_sp_3_1_23
PWY_5686__UMP_biosynthesis_g__Bacteroides_s__Bacteroides_sp_D20
PWY_5686__UMP_biosynthesis_g__Bacteroides_s__Bacteroides_stercoris
PWY_5686__UMP_biosynthesis_g__Bacteroides_s__Bacteroides_thetaiotaomicron
PWY_5686__UMP_biosynthesis_g__Bacteroides_s__Bacteroides_uniformis
PWY_5686__UMP_biosynthesis_g__Barnesiella_s__Barnesiella_intestinihominis
PWY_5686__UMP_biosynthesis_g__Bifidobacterium_s__Bifidobacterium_animalis
PWY_5686__UMP_biosynthesis_g__Bifidobacterium_s__Bifidobacterium_catenulatum
PWY_5686__UMP_biosynthesis_g__Bifidobacterium_s__Bifidobacterium_dentium
PWY_5686__UMP_biosynthesis_g__Bifidobacterium_s__Bifidobacterium_pseudocatenulatum
PWY_5686__UMP_biosynthesis_g__Bilophila_s__Bilophila_wadsworthia
PWY_5686__UMP_biosynthesis_g__Blautia_s__Blautia_sp_KLE_1732
PWY_5686__UMP_biosynthesis_g__Blautia_s__Ruminococcus_gnavus

PWY_5686__UMP_biosynthesis_g__Blautia_s__Ruminococcus_obeum
PWY_5686__UMP_biosynthesis_g__Butyrivibrio_s__Butyrivibrio_crossotus
PWY_5686__UMP_biosynthesis_g__Citrobacter_s__Citrobacter_freundii
PWY_5686__UMP_biosynthesis_g__Clostridiales_noname_s__Bacteroides_pectinophilus
PWY_5686__UMP_biosynthesis_g__Clostridium_s__Clostridium_hathewayi
PWY_5686__UMP_biosynthesis_g__Clostridium_s__Clostridium_leptum
PWY_5686__UMP_biosynthesis_g__Clostridium_s__Clostridium_perfringens
PWY_5686__UMP_biosynthesis_g__Clostridium_s__Clostridium_sp_SS2_1
PWY_5686__UMP_biosynthesis_g__Clostridium_s__Clostridium_symbiosum
PWY_5686__UMP_biosynthesis_g__Collinsella_s__Collinsella_intestinalis
PWY_5686__UMP_biosynthesis_g__Collinsella_s__Collinsella_tanakaei
PWY_5686__UMP_biosynthesis_g__Coprococcus_s__Coprococcus_catus
PWY_5686__UMP_biosynthesis_g__Coprococcus_s__Coprococcus_eutactus
PWY_5686__UMP_biosynthesis_g__Dialister_s__Dialister_invisus
PWY_5686__UMP_biosynthesis_g__Eggerthella_s__Eggerthella_lenta
PWY_5686__UMP_biosynthesis_g__Eggerthella_s__Eggerthella_sp_1_3_56FAA
PWY_5686__UMP_biosynthesis_g__Eggerthella_s__Eggerthella_sp_HGA1
PWY_5686__UMP_biosynthesis_g__Enterobacter_s__Enterobacter_cloacae
PWY_5686__UMP_biosynthesis_g__Erysipelotrichaceae_noname_s__Clostridium_spiroforme
PWY_5686__UMP_biosynthesis_g__Erysipelotrichaceae_noname_s__Erysipelotrichaceae_bacteriu
PWY_5686__UMP_biosynthesis_g__Erysipelotrichaceae_noname_s__Erysipelotrichaceae_bacteriu
PWY_5686__UMP_biosynthesis_g__Erysipelotrichaceae_noname_s__Eubacterium_biforme
PWY_5686__UMP_biosynthesis_g__Erysipelotrichaceae_noname_s__Eubacterium_cylindroides
PWY_5686__UMP_biosynthesis_g__Eubacterium_s__Eubacterium_ramulus
PWY_5686__UMP_biosynthesis_g__Eubacterium_s__Eubacterium_siraeum
PWY_5686__UMP_biosynthesis_g__Eubacterium_s__Eubacterium_sp_3_1_31
PWY_5686__UMP_biosynthesis_g__Eubacterium_s__Eubacterium_ventriosum
PWY_5686__UMP_biosynthesis_g__Fusobacterium_s__Fusobacterium_nucleatum
PWY_5686__UMP_biosynthesis_g__Gemella_s__Gemella_sanguinis
PWY_5686__UMP_biosynthesis_g__Holdemania_s__Holdemania_filiformis
PWY_5686__UMP_biosynthesis_g__Klebsiella_s__Klebsiella_pneumoniae
PWY_5686__UMP_biosynthesis_g__Lachnospiraceae_noname_s__Lachnospiraceae_bacterium_1_
PWY_5686__UMP_biosynthesis_g__Lachnospiraceae_noname_s__Lachnospiraceae_bacterium_2_
PWY_5686__UMP_biosynthesis_g__Lachnospiraceae_noname_s__Lachnospiraceae_bacterium_3_
PWY_5686__UMP_biosynthesis_g__Lachnospiraceae_noname_s__Lachnospiraceae_bacterium_5_
PWY_5686__UMP_biosynthesis_g__Lachnospiraceae_noname_s__Lachnospiraceae_bacterium_7_
PWY_5686__UMP_biosynthesis_g__Lactobacillus_s__Lactobacillus_fermentum
PWY_5686__UMP_biosynthesis_g__Lactococcus_s__Lactococcus_lactis
PWY_5686__UMP_biosynthesis_g__Methanobrevibacter_s__Methanobrevibacter_smithii
PWY_5686__UMP_biosynthesis_g__Odoribacter_s__Odoribacter_laneus
PWY_5686__UMP_biosynthesis_g__Odoribacter_s__Odoribacter_splanchnicus
PWY_5686__UMP_biosynthesis_g__Oxalobacter_s__Oxalobacter_formigenes
PWY_5686__UMP_biosynthesis_g__Parabacteroides_s__Parabacteroides_distasonis
PWY_5686__UMP_biosynthesis_g__Parabacteroides_s__Parabacteroides_goldsteinii
PWY_5686__UMP_biosynthesis_g__Parabacteroides_s__Parabacteroides_johnsonii
PWY_5686__UMP_biosynthesis_g__Parabacteroides_s__Parabacteroides_merdae
PWY_5686__UMP_biosynthesis_g__Parabacteroides_s__Parabacteroides_sp_20_3

PWY_5686__UMP_biosynthesis_g__Parabacteroides_s__Parabacteroides_sp_D13
PWY_5686__UMP_biosynthesis_g__Pediococcus_s__Pediococcus_acidilactici
PWY_5686__UMP_biosynthesis_g__Pediococcus_s__Pediococcus_lolii
PWY_5686__UMP_biosynthesis_g__Peptostreptococcaceae_noname_s__Clostridium_bartlettii
PWY_5686__UMP_biosynthesis_g__Phascolarctobacterium_s__Phascolarctobacterium_succinatut
PWY_5686__UMP_biosynthesis_g__Prevotella_s__Prevotella_copri
PWY_5686__UMP_biosynthesis_g__Propionibacterium_s__Propionibacterium_freudenreichii
PWY_5686__UMP_biosynthesis_g__Roseburia_s__Roseburia_hominis
PWY_5686__UMP_biosynthesis_g__Ruminococcaceae_noname_s__Ruminococcaceae_bacterium_
PWY_5686__UMP_biosynthesis_g__Ruminococcus_s__Ruminococcus_champanellensis
PWY_5686__UMP_biosynthesis_g__Ruminococcus_s__Ruminococcus_sp
PWY_5686__UMP_biosynthesis_g__Streptococcus_s__Streptococcus_australis
PWY_5686__UMP_biosynthesis_g__Streptococcus_s__Streptococcus_mitis_oralis_pneumoniae
PWY_5686__UMP_biosynthesis_g__Subdoligranulum_s__Subdoligranulum_sp_4_3_54A2FAA
PWY_5686__UMP_biosynthesis_g__Veillonella_s__Veillonella_dispar
PWY_5686__UMP_biosynthesis_g__Veillonella_s__Veillonella_parvula
PWY_5690__TCA_cycle_II_(plants_and_fungi)_g__Citrobacter_s__Citrobacter_freundii
PWY_5695__urate_biosynthesis_inosine_5__phosphate_degradation_g__Alistipes_s__Alistipes_in
PWY_5695__urate_biosynthesis_inosine_5__phosphate_degradation_g__Alistipes_s__Alistipes_sh
PWY_5695__urate_biosynthesis_inosine_5__phosphate_degradation_g__Bacteroides_s__Bacteroi
PWY_5695__urate_biosynthesis_inosine_5__phosphate_degradation_g__Bacteroides_s__Bacteroi
PWY_5695__urate_biosynthesis_inosine_5__phosphate_degradation_g__Bacteroides_s__Bacteroi
PWY_5695__urate_biosynthesis_inosine_5__phosphate_degradation_g__Bacteroides_s__Bacteroi
PWY_5695__urate_biosynthesis_inosine_5__phosphate_degradation_g__Bacteroides_s__Bacteroi
PWY_5695__urate_biosynthesis_inosine_5__phosphate_degradation_g__Bacteroides_s__Bacteroi
PWY_5695__urate_biosynthesis_inosine_5__phosphate_degradation_g__Bacteroides_s__Bacteroi
PWY_5695__urate_biosynthesis_inosine_5__phosphate_degradation_g__Bacteroides_s__Bacteroi
PWY_5695__urate_biosynthesis_inosine_5__phosphate_degradation_g__Bacteroides_s__Bacteroi
PWY_5695__urate_biosynthesis_inosine_5__phosphate_degradation_g__Bacteroides_s__Bacteroi
PWY_5695__urate_biosynthesis_inosine_5__phosphate_degradation_g__Bacteroides_s__Bacteroi
PWY_5695__urate_biosynthesis_inosine_5__phosphate_degradation_g__Bacteroides_s__Bacteroi
PWY_5695__urate_biosynthesis_inosine_5__phosphate_degradation_g__Bacteroides_s__Bacteroi
PWY_5695__urate_biosynthesis_inosine_5__phosphate_degradation_g__Bacteroides_s__Bacteroi
PWY_5695__urate_biosynthesis_inosine_5__phosphate_degradation_g__Bacteroides_s__Bacteroi
PWY_5695__urate_biosynthesis_inosine_5__phosphate_degradation_g__Bacteroides_s__Bacteroi
PWY_5695__urate_biosynthesis_inosine_5__phosphate_degradation_g__Bacteroides_s__Bacteroi
PWY_5695__urate_biosynthesis_inosine_5__phosphate_degradation_g__Bacteroides_s__Bacteroi
PWY_5695__urate_biosynthesis_inosine_5__phosphate_degradation_g__Bacteroides_s__Bacteroi
PWY_5695__urate_biosynthesis_inosine_5__phosphate_degradation_g__Bacteroides_s__Bacteroi
PWY_5695__urate_biosynthesis_inosine_5__phosphate_degradation_g__Barnesiella_s__Barnesie
PWY_5695__urate_biosynthesis_inosine_5__phosphate_degradation_g__Blautia_s__Ruminococcu
PWY_5695__urate_biosynthesis_inosine_5__phosphate_degradation_g__Citrobacter_s__Citrobact
PWY_5695__urate_biosynthesis_inosine_5__phosphate_degradation_g__Coprobacter_s__Coprob
PWY_5695__urate_biosynthesis_inosine_5__phosphate_degradation_g__Coprococcus_s__Coproc
PWY_5695__urate_biosynthesis_inosine_5__phosphate_degradation_g__Enterobacter_s__Entero
PWY_5695__urate_biosynthesis_inosine_5__phosphate_degradation_g__Eubacterium_s__Eubacte

PWY_5695__urate_biosynthesis_inosine_5__phosphate_degradation_g__Klebsiella_s__Klebsiella_
PWY_5695__urate_biosynthesis_inosine_5__phosphate_degradation_g__Lactococcus_s__Lactococ
PWY_5695__urate_biosynthesis_inosine_5__phosphate_degradation_g__Odoribacter_s__Odoriba
PWY_5695__urate_biosynthesis_inosine_5__phosphate_degradation_g__Parabacteroides_s__Para
PWY_5695__urate_biosynthesis_inosine_5__phosphate_degradation_g__Parabacteroides_s__Para
PWY_5695__urate_biosynthesis_inosine_5__phosphate_degradation_g__Parabacteroides_s__Para
PWY_5695__urate_biosynthesis_inosine_5__phosphate_degradation_g__Parabacteroides_s__Para
PWY_5695__urate_biosynthesis_inosine_5__phosphate_degradation_g__Parabacteroides_s__Para
PWY_5695__urate_biosynthesis_inosine_5__phosphate_degradation_g__Peptostreptococcaceae_
PWY_5695__urate_biosynthesis_inosine_5__phosphate_degradation_g__Roseburia_s__Roseburia
PWY_5695__urate_biosynthesis_inosine_5__phosphate_degradation_g__Ruminococcus_s__Rumi
PWY_5705__allantoin_degradation_to_glyoxylate_III_unclassified
PWY_5723__Rubisco_shunt_g__Citrobacter_s__Citrobacter_freundii
PWY_5747__2_methylcitrate_cycle_II
PWY_5747__2_methylcitrate_cycle_II_g__Citrobacter_s__Citrobacter_freundii
PWY_5747__2_methylcitrate_cycle_II_unclassified
PWY_5791__1,4_dihydroxy_2_naphthoate_biosynthesis_II_(plants)_g__Lactococcus_s__Lactococc
PWY_5837__1,4_dihydroxy_2_naphthoate_biosynthesis_I_g__Lactococcus_s__Lactococcus_lactis
PWY_5838__superpathway_of_menaquinol_8_biosynthesis_I_unclassified
PWY_5845__superpathway_of_menaquinol_9_biosynthesis_unclassified
PWY_5850__superpathway_of_menaquinol_6_biosynthesis_I_unclassified
PWY_5855__ubiquinol_7_biosynthesis_(prokaryotic)_g__Citrobacter_s__Citrobacter_freundii
PWY_5855__ubiquinol_7_biosynthesis_(prokaryotic)_g__Enterobacter_s__Enterobacter_cloacae
PWY_5855__ubiquinol_7_biosynthesis_(prokaryotic)_g__Klebsiella_s__Klebsiella_pneumoniae
PWY_5855__ubiquinol_7_biosynthesis_(prokaryotic)_unclassified
PWY_5856__ubiquinol_9_biosynthesis_(prokaryotic)_g__Citrobacter_s__Citrobacter_freundii
PWY_5856__ubiquinol_9_biosynthesis_(prokaryotic)_g__Enterobacter_s__Enterobacter_cloacae
PWY_5856__ubiquinol_9_biosynthesis_(prokaryotic)_g__Klebsiella_s__Klebsiella_pneumoniae
PWY_5856__ubiquinol_9_biosynthesis_(prokaryotic)_unclassified
PWY_5857__ubiquinol_10_biosynthesis_(prokaryotic)_g__Citrobacter_s__Citrobacter_freundii
PWY_5857__ubiquinol_10_biosynthesis_(prokaryotic)_g__Enterobacter_s__Enterobacter_cloacae
PWY_5857__ubiquinol_10_biosynthesis_(prokaryotic)_g__Klebsiella_s__Klebsiella_pneumoniae
PWY_5857__ubiquinol_10_biosynthesis_(prokaryotic)_unclassified
PWY_5860__superpathway_of_demethylmenaquinol_6_biosynthesis_I_unclassified
PWY_5861__superpathway_of_demethylmenaquinol_8_biosynthesis_unclassified
PWY_5862__superpathway_of_demethylmenaquinol_9_biosynthesis_unclassified
PWY_5896__superpathway_of_menaquinol_10_biosynthesis_unclassified
PWY_5913__TCA_cycle_VI_(obligate_autotrophs)_g__Citrobacter_s__Citrobacter_freundii
PWY_5918__superpathay_of_heme_biosynthesis_from_glutamate_g__Citrobacter_s__Citrobacter
PWY_5918__superpathay_of_heme_biosynthesis_from_glutamate_g__Enterobacter_s__Enteroba
PWY_5918__superpathay_of_heme_biosynthesis_from_glutamate_g__Propionibacterium_s__Pro
PWY_5918__superpathay_of_heme_biosynthesis_from_glutamate_unclassified
PWY_5920__superpathway_of_heme_biosynthesis_from_glycine_g__Propionibacterium_s__Propi
PWY_5920__superpathway_of_heme_biosynthesis_from_glycine_unclassified
PWY_5989__stearate_biosynthesis_II_(bacteria_and_plants)_g__Bacteroides_s__Bacteroides_sp_
PWY_5989__stearate_biosynthesis_II_(bacteria_and_plants)_g__Bacteroides_s__Bacteroides_xyla
PWY_5989__stearate_biosynthesis_II_(bacteria_and_plants)_g__Citrobacter_s__Citrobacter_freur

PWY_5989__stearate_biosynthesis_II_(bacteria_and_plants)_g__Enterobacter_s__Enterobacter_cl
PWY_5989__stearate_biosynthesis_II_(bacteria_and_plants)_g__Haemophilus_s__Haemophilus_p
PWY_5989__stearate_biosynthesis_II_(bacteria_and_plants)_g__Klebsiella_s__Klebsiella_pneumo
PWY_5994__palmitate_biosynthesis_I_(animals_and_fungi)
PWY_6121__5_aminoimidazole_ribonucleotide_biosynthesis_I_g__Adlercreutzia_s__Adlercreutzia
PWY_6121__5_aminoimidazole_ribonucleotide_biosynthesis_I_g__Akkermansia_s__Akkermansia_
PWY_6121__5_aminoimidazole_ribonucleotide_biosynthesis_I_g__Alistipes_s__Alistipes_finegold
PWY_6121__5_aminoimidazole_ribonucleotide_biosynthesis_I_g__Alistipes_s__Alistipes_onderdo
PWY_6121__5_aminoimidazole_ribonucleotide_biosynthesis_I_g__Alistipes_s__Alistipes_putredir
PWY_6121__5_aminoimidazole_ribonucleotide_biosynthesis_I_g__Alistipes_s__Alistipes_sp_HGB
PWY_6121__5_aminoimidazole_ribonucleotide_biosynthesis_I_g__Anaerofustis_s__Anaerofustis_
PWY_6121__5_aminoimidazole_ribonucleotide_biosynthesis_I_g__Anaerostipes_s__Anaerostipes_
PWY_6121__5_aminoimidazole_ribonucleotide_biosynthesis_I_g__Anaerotruncus_s__Anaerotrun
PWY_6121__5_aminoimidazole_ribonucleotide_biosynthesis_I_g__Bacteroides_s__Bacteroides_sp
PWY_6121__5_aminoimidazole_ribonucleotide_biosynthesis_I_g__Bilophila_s__Bilophila_wadswo
PWY_6121__5_aminoimidazole_ribonucleotide_biosynthesis_I_g__Blautia_s__Blautia_hydrogeno
PWY_6121__5_aminoimidazole_ribonucleotide_biosynthesis_I_g__Blautia_s__Blautia_sp_KLE_17
PWY_6121__5_aminoimidazole_ribonucleotide_biosynthesis_I_g__Blautia_s__Ruminococcus_gna
PWY_6121__5_aminoimidazole_ribonucleotide_biosynthesis_I_g__Blautia_s__Ruminococcus_obe
PWY_6121__5_aminoimidazole_ribonucleotide_biosynthesis_I_g__Blautia_s__Ruminococcus_torc
PWY_6121__5_aminoimidazole_ribonucleotide_biosynthesis_I_g__Burkholderiales_noname_s__B
PWY_6121__5_aminoimidazole_ribonucleotide_biosynthesis_I_g__Butyrivibrio_s__Butyrivibrio_cr
PWY_6121__5_aminoimidazole_ribonucleotide_biosynthesis_I_g__Catenibacterium_s__Catenibac
PWY_6121__5_aminoimidazole_ribonucleotide_biosynthesis_I_g__Clostridiales_noname_s__Bact
PWY_6121__5_aminoimidazole_ribonucleotide_biosynthesis_I_g__Clostridiales_noname_s__Clost
PWY_6121__5_aminoimidazole_ribonucleotide_biosynthesis_I_g__Clostridium_s__Clostridium_as
PWY_6121__5_aminoimidazole_ribonucleotide_biosynthesis_I_g__Clostridium_s__Clostridium_cl
PWY_6121__5_aminoimidazole_ribonucleotide_biosynthesis_I_g__Clostridium_s__Clostridium_ha
PWY_6121__5_aminoimidazole_ribonucleotide_biosynthesis_I_g__Clostridium_s__Clostridium_le
PWY_6121__5_aminoimidazole_ribonucleotide_biosynthesis_I_g__Clostridium_s__Clostridium_sp
PWY_6121__5_aminoimidazole_ribonucleotide_biosynthesis_I_g__Clostridium_s__Clostridium_sp
PWY_6121__5_aminoimidazole_ribonucleotide_biosynthesis_I_g__Collinsella_s__Collinsella_intes
PWY_6121__5_aminoimidazole_ribonucleotide_biosynthesis_I_g__Collinsella_s__Collinsella_tanal
PWY_6121__5_aminoimidazole_ribonucleotide_biosynthesis_I_g__Coprococcus_s__Coprococcus_
PWY_6121__5_aminoimidazole_ribonucleotide_biosynthesis_I_g__Coprococcus_s__Coprococcus_
PWY_6121__5_aminoimidazole_ribonucleotide_biosynthesis_I_g__Dialister_s__Dialister_invisus
PWY_6121__5_aminoimidazole_ribonucleotide_biosynthesis_I_g__Dorea_s__Dorea_formicigener
PWY_6121__5_aminoimidazole_ribonucleotide_biosynthesis_I_g__Eggerthella_s__Eggerthella_ler
PWY_6121__5_aminoimidazole_ribonucleotide_biosynthesis_I_g__Eggerthella_s__Eggerthella_sp_
PWY_6121__5_aminoimidazole_ribonucleotide_biosynthesis_I_g__Eggerthella_s__Eggerthella_sp_
PWY_6121__5_aminoimidazole_ribonucleotide_biosynthesis_I_g__Enterococcus_s__Enterococcus
PWY_6121__5_aminoimidazole_ribonucleotide_biosynthesis_I_g__Enterococcus_s__Enterococcus
PWY_6121__5_aminoimidazole_ribonucleotide_biosynthesis_I_g__Enterococcus_s__Enterococcus
PWY_6121__5_aminoimidazole_ribonucleotide_biosynthesis_I_g__Erysipelotrichaceae_noname_s
PWY_6121__5_aminoimidazole_ribonucleotide_biosynthesis_I_g__Erysipelotrichaceae_noname_s
PWY_6121__5_aminoimidazole_ribonucleotide_biosynthesis_I_g__Erysipelotrichaceae_noname_s
PWY_6121__5_aminoimidazole_ribonucleotide_biosynthesis_I_g__Eubacterium_s__Eubacterium_

PWY_6121__5_aminoimidazole_ribonucleotide_biosynthesis_I_g__Eubacterium_s__Eubacterium_
PWY_6121__5_aminoimidazole_ribonucleotide_biosynthesis_I_g__Eubacterium_s__Eubacterium_
PWY_6121__5_aminoimidazole_ribonucleotide_biosynthesis_I_g__Eubacterium_s__Eubacterium_
PWY_6121__5_aminoimidazole_ribonucleotide_biosynthesis_I_g__Fusobacterium_s__Fusobacteri
PWY_6121__5_aminoimidazole_ribonucleotide_biosynthesis_I_g__Gemella_s__Gemella_sanguini
PWY_6121__5_aminoimidazole_ribonucleotide_biosynthesis_I_g__Holdemania_s__Holdemania_f
PWY_6121__5_aminoimidazole_ribonucleotide_biosynthesis_I_g__Klebsiella_s__Klebsiella_pneum
PWY_6121__5_aminoimidazole_ribonucleotide_biosynthesis_I_g__Lachnospiraceae_noname_s__
PWY_6121__5_aminoimidazole_ribonucleotide_biosynthesis_I_g__Lachnospiraceae_noname_s__
PWY_6121__5_aminoimidazole_ribonucleotide_biosynthesis_I_g__Lachnospiraceae_noname_s__
PWY_6121__5_aminoimidazole_ribonucleotide_biosynthesis_I_g__Lachnospiraceae_noname_s__
PWY_6121__5_aminoimidazole_ribonucleotide_biosynthesis_I_g__Lachnospiraceae_noname_s__
PWY_6121__5_aminoimidazole_ribonucleotide_biosynthesis_I_g__Lactobacillus_s__Lactobacillus_
PWY_6121__5_aminoimidazole_ribonucleotide_biosynthesis_I_g__Lactobacillus_s__Lactobacillus_
PWY_6121__5_aminoimidazole_ribonucleotide_biosynthesis_I_g__Lactococcus_s__Lactococcus_la
PWY_6121__5_aminoimidazole_ribonucleotide_biosynthesis_I_g__Odoribacter_s__Odoribacter_la
PWY_6121__5_aminoimidazole_ribonucleotide_biosynthesis_I_g__Odoribacter_s__Odoribacter_s
PWY_6121__5_aminoimidazole_ribonucleotide_biosynthesis_I_g__Oxalobacter_s__Oxalobacter_f
PWY_6121__5_aminoimidazole_ribonucleotide_biosynthesis_I_g__Parasutterella_s__Parasutterel
PWY_6121__5_aminoimidazole_ribonucleotide_biosynthesis_I_g__Pediococcus_s__Pediococcus_a
PWY_6121__5_aminoimidazole_ribonucleotide_biosynthesis_I_g__Pediococcus_s__Pediococcus_
PWY_6121__5_aminoimidazole_ribonucleotide_biosynthesis_I_g__Peptostreptococcaceae_nonam
PWY_6121__5_aminoimidazole_ribonucleotide_biosynthesis_I_g__Phascolarctobacterium_s__Pha
PWY_6121__5_aminoimidazole_ribonucleotide_biosynthesis_I_g__Propionibacterium_s__Propion
PWY_6121__5_aminoimidazole_ribonucleotide_biosynthesis_I_g__Roseburia_s__Roseburia_homi
PWY_6121__5_aminoimidazole_ribonucleotide_biosynthesis_I_g__Roseburia_s__Roseburia_inulir
PWY_6121__5_aminoimidazole_ribonucleotide_biosynthesis_I_g__Ruminococcaceae_noname_s__
PWY_6121__5_aminoimidazole_ribonucleotide_biosynthesis_I_g__Ruminococcus_s__Ruminococc
PWY_6121__5_aminoimidazole_ribonucleotide_biosynthesis_I_g__Ruminococcus_s__Ruminococc
PWY_6121__5_aminoimidazole_ribonucleotide_biosynthesis_I_g__Ruminococcus_s__Ruminococc
PWY_6121__5_aminoimidazole_ribonucleotide_biosynthesis_I_g__Slackia_s__Slackia_piriformis
PWY_6121__5_aminoimidazole_ribonucleotide_biosynthesis_I_g__Solobacterium_s__Solobacteriu
PWY_6121__5_aminoimidazole_ribonucleotide_biosynthesis_I_g__Subdoligranulum_s__Subdoligr
PWY_6121__5_aminoimidazole_ribonucleotide_biosynthesis_I_g__Veillonella_s__Veillonella_disp
PWY_6121__5_aminoimidazole_ribonucleotide_biosynthesis_I_g__Veillonella_s__Veillonella_parv
PWY_6122__5_aminoimidazole_ribonucleotide_biosynthesis_II_g__Adlercreutzia_s__Adlercreutzi
PWY_6122__5_aminoimidazole_ribonucleotide_biosynthesis_II_g__Akkermansia_s__Akkermansia
PWY_6122__5_aminoimidazole_ribonucleotide_biosynthesis_II_g__Alistipes_s__Alistipes_finegolc
PWY_6122__5_aminoimidazole_ribonucleotide_biosynthesis_II_g__Alistipes_s__Alistipes_onderdc
PWY_6122__5_aminoimidazole_ribonucleotide_biosynthesis_II_g__Alistipes_s__Alistipes_putredir
PWY_6122__5_aminoimidazole_ribonucleotide_biosynthesis_II_g__Alistipes_s__Alistipes_sp_HGB
PWY_6122__5_aminoimidazole_ribonucleotide_biosynthesis_II_g__Anaerofustis_s__Anaerofustis_
PWY_6122__5_aminoimidazole_ribonucleotide_biosynthesis_II_g__Anaerostipes_s__Anaerostipes
PWY_6122__5_aminoimidazole_ribonucleotide_biosynthesis_II_g__Anaerotruncus_s__Anaerotrur
PWY_6122__5_aminoimidazole_ribonucleotide_biosynthesis_II_g__Bacteroides_s__Bacteroides_s
PWY_6122__5_aminoimidazole_ribonucleotide_biosynthesis_II_g__Bifidobacterium_s__Bifidobact
PWY_6122__5_aminoimidazole_ribonucleotide_biosynthesis_II_g__Bifidobacterium_s__Bifidobact

PWY_6122__5_aminoimidazole_ribonucleotide_biosynthesis_II_g__Bifidobacterium_s__Bifidobact
PWY_6122__5_aminoimidazole_ribonucleotide_biosynthesis_II_g__Bifidobacterium_s__Bifidobact
PWY_6122__5_aminoimidazole_ribonucleotide_biosynthesis_II_g__Bifidobacterium_s__Bifidobact
PWY_6122__5_aminoimidazole_ribonucleotide_biosynthesis_II_g__Bilophila_s__Bilophila_wadsw
PWY_6122__5_aminoimidazole_ribonucleotide_biosynthesis_II_g__Blautia_s__Blautia_hydrogeno
PWY_6122__5_aminoimidazole_ribonucleotide_biosynthesis_II_g__Blautia_s__Blautia_sp_KLE_17
PWY_6122__5_aminoimidazole_ribonucleotide_biosynthesis_II_g__Blautia_s__Ruminococcus_gna
PWY_6122__5_aminoimidazole_ribonucleotide_biosynthesis_II_g__Blautia_s__Ruminococcus_obe
PWY_6122__5_aminoimidazole_ribonucleotide_biosynthesis_II_g__Blautia_s__Ruminococcus_tor
PWY_6122__5_aminoimidazole_ribonucleotide_biosynthesis_II_g__Burkholderiales_noname_s__
PWY_6122__5_aminoimidazole_ribonucleotide_biosynthesis_II_g__Butyrivibrio_s__Butyrivibrio_c
PWY_6122__5_aminoimidazole_ribonucleotide_biosynthesis_II_g__Catenibacterium_s__Catenibac
PWY_6122__5_aminoimidazole_ribonucleotide_biosynthesis_II_g__Clostridiales_noname_s__Bact
PWY_6122__5_aminoimidazole_ribonucleotide_biosynthesis_II_g__Clostridiales_noname_s__Clos
PWY_6122__5_aminoimidazole_ribonucleotide_biosynthesis_II_g__Clostridium_s__Clostridium_a
PWY_6122__5_aminoimidazole_ribonucleotide_biosynthesis_II_g__Clostridium_s__Clostridium_c
PWY_6122__5_aminoimidazole_ribonucleotide_biosynthesis_II_g__Clostridium_s__Clostridium_h
PWY_6122__5_aminoimidazole_ribonucleotide_biosynthesis_II_g__Clostridium_s__Clostridium_le
PWY_6122__5_aminoimidazole_ribonucleotide_biosynthesis_II_g__Clostridium_s__Clostridium_p
PWY_6122__5_aminoimidazole_ribonucleotide_biosynthesis_II_g__Clostridium_s__Clostridium_s
PWY_6122__5_aminoimidazole_ribonucleotide_biosynthesis_II_g__Clostridium_s__Clostridium_s
PWY_6122__5_aminoimidazole_ribonucleotide_biosynthesis_II_g__Collinsella_s__Collinsella_inte
PWY_6122__5_aminoimidazole_ribonucleotide_biosynthesis_II_g__Collinsella_s__Collinsella_tana
PWY_6122__5_aminoimidazole_ribonucleotide_biosynthesis_II_g__Coprococcus_s__Coprococcus_
PWY_6122__5_aminoimidazole_ribonucleotide_biosynthesis_II_g__Coprococcus_s__Coprococcus_
PWY_6122__5_aminoimidazole_ribonucleotide_biosynthesis_II_g__Dialister_s__Dialister_invisus
PWY_6122__5_aminoimidazole_ribonucleotide_biosynthesis_II_g__Dorea_s__Dorea_formicigener
PWY_6122__5_aminoimidazole_ribonucleotide_biosynthesis_II_g__Eggerthella_s__Eggerthella_le
PWY_6122__5_aminoimidazole_ribonucleotide_biosynthesis_II_g__Eggerthella_s__Eggerthella_sp
PWY_6122__5_aminoimidazole_ribonucleotide_biosynthesis_II_g__Eggerthella_s__Eggerthella_sp
PWY_6122__5_aminoimidazole_ribonucleotide_biosynthesis_II_g__Enterobacter_s__Enterobacter
PWY_6122__5_aminoimidazole_ribonucleotide_biosynthesis_II_g__Enterococcus_s__Enterococcu
PWY_6122__5_aminoimidazole_ribonucleotide_biosynthesis_II_g__Enterococcus_s__Enterococcu
PWY_6122__5_aminoimidazole_ribonucleotide_biosynthesis_II_g__Enterococcus_s__Enterococcu
PWY_6122__5_aminoimidazole_ribonucleotide_biosynthesis_II_g__Enterorhabdus_s__Enterorhab
PWY_6122__5_aminoimidazole_ribonucleotide_biosynthesis_II_g__Erysipelotrichaceae_noname_
PWY_6122__5_aminoimidazole_ribonucleotide_biosynthesis_II_g__Erysipelotrichaceae_noname_
PWY_6122__5_aminoimidazole_ribonucleotide_biosynthesis_II_g__Erysipelotrichaceae_noname_
PWY_6122__5_aminoimidazole_ribonucleotide_biosynthesis_II_g__Eubacterium_s__Eubacterium_
PWY_6122__5_aminoimidazole_ribonucleotide_biosynthesis_II_g__Eubacterium_s__Eubacterium_
PWY_6122__5_aminoimidazole_ribonucleotide_biosynthesis_II_g__Eubacterium_s__Eubacterium_
PWY_6122__5_aminoimidazole_ribonucleotide_biosynthesis_II_g__Eubacterium_s__Eubacterium_
PWY_6122__5_aminoimidazole_ribonucleotide_biosynthesis_II_g__Fusobacterium_s__Fusobacter
PWY_6122__5_aminoimidazole_ribonucleotide_biosynthesis_II_g__Gemella_s__Gemella_sanguin
PWY_6122__5_aminoimidazole_ribonucleotide_biosynthesis_II_g__Holdemania_s__Holdemania_
PWY_6122__5_aminoimidazole_ribonucleotide_biosynthesis_II_g__Klebsiella_s__Klebsiella_pneu
PWY_6122__5_aminoimidazole_ribonucleotide_biosynthesis_II_g__Lachnospiraceae_noname_s__

PWY_6122__5_aminoimidazole_ribonucleotide_biosynthesis_II_g__Lachnospiraceae_noname_s__
PWY_6122__5_aminoimidazole_ribonucleotide_biosynthesis_II_g__Lachnospiraceae_noname_s__
PWY_6122__5_aminoimidazole_ribonucleotide_biosynthesis_II_g__Lachnospiraceae_noname_s__
PWY_6122__5_aminoimidazole_ribonucleotide_biosynthesis_II_g__Lachnospiraceae_noname_s__
PWY_6122__5_aminoimidazole_ribonucleotide_biosynthesis_II_g__Lachnospiraceae_noname_s__
PWY_6122__5_aminoimidazole_ribonucleotide_biosynthesis_II_g__Lactobacillus_s__Lactobacillus_
PWY_6122__5_aminoimidazole_ribonucleotide_biosynthesis_II_g__Lactobacillus_s__Lactobacillus_
PWY_6122__5_aminoimidazole_ribonucleotide_biosynthesis_II_g__Lactobacillus_s__Lactobacillus_
PWY_6122__5_aminoimidazole_ribonucleotide_biosynthesis_II_g__Odoribacter_s__Odoribacter_l
PWY_6122__5_aminoimidazole_ribonucleotide_biosynthesis_II_g__Odoribacter_s__Odoribacter_s
PWY_6122__5_aminoimidazole_ribonucleotide_biosynthesis_II_g__Oxalobacter_s__Oxalobacter_f
PWY_6122__5_aminoimidazole_ribonucleotide_biosynthesis_II_g__Parasutterella_s__Parasuttere
PWY_6122__5_aminoimidazole_ribonucleotide_biosynthesis_II_g__Pediococcus_s__Pediococcus_
PWY_6122__5_aminoimidazole_ribonucleotide_biosynthesis_II_g__Pediococcus_s__Pediococcus_
PWY_6122__5_aminoimidazole_ribonucleotide_biosynthesis_II_g__Peptostreptococcaceae_nonar
PWY_6122__5_aminoimidazole_ribonucleotide_biosynthesis_II_g__Phascolarctobacterium_s__Pha
PWY_6122__5_aminoimidazole_ribonucleotide_biosynthesis_II_g__Propionibacterium_s__Propion
PWY_6122__5_aminoimidazole_ribonucleotide_biosynthesis_II_g__Roseburia_s__Roseburia_hom
PWY_6122__5_aminoimidazole_ribonucleotide_biosynthesis_II_g__Roseburia_s__Roseburia_inuli
PWY_6122__5_aminoimidazole_ribonucleotide_biosynthesis_II_g__Ruminococcaceae_noname_s_
PWY_6122__5_aminoimidazole_ribonucleotide_biosynthesis_II_g__Ruminococcus_s__Ruminococ
PWY_6122__5_aminoimidazole_ribonucleotide_biosynthesis_II_g__Ruminococcus_s__Ruminococ
PWY_6122__5_aminoimidazole_ribonucleotide_biosynthesis_II_g__Ruminococcus_s__Ruminococ
PWY_6122__5_aminoimidazole_ribonucleotide_biosynthesis_II_g__Slackia_s__Slackia_piriformis
PWY_6122__5_aminoimidazole_ribonucleotide_biosynthesis_II_g__Solobacterium_s__Solobacteri
PWY_6122__5_aminoimidazole_ribonucleotide_biosynthesis_II_g__Streptococcus_s__Streptococc
PWY_6122__5_aminoimidazole_ribonucleotide_biosynthesis_II_g__Subdoligranulum_s__Subdolig
PWY_6122__5_aminoimidazole_ribonucleotide_biosynthesis_II_g__Veillonella_s__Veillonella_disp
PWY_6122__5_aminoimidazole_ribonucleotide_biosynthesis_II_g__Veillonella_s__Veillonella_par
PWY_6123__inosine_5__phosphate_biosynthesis_I_g__Bacteroides_s__Bacteroides_faecis
PWY_6123__inosine_5__phosphate_biosynthesis_I_g__Bacteroides_s__Bacteroides_finegoldii
PWY_6123__inosine_5__phosphate_biosynthesis_I_g__Bacteroides_s__Bacteroides_fragilis
PWY_6123__inosine_5__phosphate_biosynthesis_I_g__Bacteroides_s__Bacteroides_ovatus
PWY_6123__inosine_5__phosphate_biosynthesis_I_g__Bacteroides_s__Bacteroides_salyersiae
PWY_6123__inosine_5__phosphate_biosynthesis_I_g__Bacteroides_s__Bacteroides_sp_1_1_30
PWY_6123__inosine_5__phosphate_biosynthesis_I_g__Bacteroides_s__Bacteroides_sp_1_1_6
PWY_6123__inosine_5__phosphate_biosynthesis_I_g__Bacteroides_s__Bacteroides_sp_2_1_22
PWY_6123__inosine_5__phosphate_biosynthesis_I_g__Bacteroides_s__Bacteroides_sp_2_2_4
PWY_6123__inosine_5__phosphate_biosynthesis_I_g__Bacteroides_s__Bacteroides_sp_3_1_23
PWY_6123__inosine_5__phosphate_biosynthesis_I_g__Bacteroides_s__Bacteroides_thetaiotaomi
PWY_6123__inosine_5__phosphate_biosynthesis_I_g__Bacteroides_s__Bacteroides_xylanisolvens
PWY_6123__inosine_5__phosphate_biosynthesis_I_g__Citrobacter_s__Citrobacter_freundii
PWY_6123__inosine_5__phosphate_biosynthesis_I_g__Clostridium_s__Clostridium_perfringens
PWY_6123__inosine_5__phosphate_biosynthesis_I_g__Coprococcus_s__Coprococcus_catus
PWY_6123__inosine_5__phosphate_biosynthesis_I_g__Coprococcus_s__Coprococcus_eutactus
PWY_6123__inosine_5__phosphate_biosynthesis_I_g__Coprococcus_s__Coprococcus_sp_ART55_
PWY_6123__inosine_5__phosphate_biosynthesis_I_g__Enterobacter_s__Enterobacter_cloacae

PWY_6123__inosine_5__phosphate_biosynthesis_I_g__Enterococcus_s__Enterococcus_faecium
PWY_6123__inosine_5__phosphate_biosynthesis_I_g__Eubacterium_s__Eubacterium_siraeum
PWY_6123__inosine_5__phosphate_biosynthesis_I_g__Klebsiella_s__Klebsiella_pneumoniae
PWY_6123__inosine_5__phosphate_biosynthesis_I_g__Lachnospiraceae_noname_s__Lachnospira
PWY_6123__inosine_5__phosphate_biosynthesis_I_g__Lactobacillus_s__Lactobacillus_casei_parac
PWY_6123__inosine_5__phosphate_biosynthesis_I_g__Lactococcus_s__Lactococcus_lactis
PWY_6123__inosine_5__phosphate_biosynthesis_I_g__Ruminococcus_s__Ruminococcus_champa
PWY_6123__inosine_5__phosphate_biosynthesis_I_g__Streptococcus_s__Streptococcus_mitis_ora
PWY_6124__inosine_5__phosphate_biosynthesis_II_g__Bacteroides_s__Bacteroides_faecis
PWY_6124__inosine_5__phosphate_biosynthesis_II_g__Bacteroides_s__Bacteroides_finegoldii
PWY_6124__inosine_5__phosphate_biosynthesis_II_g__Bacteroides_s__Bacteroides_ovatus
PWY_6124__inosine_5__phosphate_biosynthesis_II_g__Bacteroides_s__Bacteroides_salyersiae
PWY_6124__inosine_5__phosphate_biosynthesis_II_g__Bacteroides_s__Bacteroides_sp_1_1_30
PWY_6124__inosine_5__phosphate_biosynthesis_II_g__Bacteroides_s__Bacteroides_sp_1_1_6
PWY_6124__inosine_5__phosphate_biosynthesis_II_g__Bacteroides_s__Bacteroides_sp_2_1_22
PWY_6124__inosine_5__phosphate_biosynthesis_II_g__Bacteroides_s__Bacteroides_sp_2_2_4
PWY_6124__inosine_5__phosphate_biosynthesis_II_g__Bacteroides_s__Bacteroides_sp_3_1_23
PWY_6124__inosine_5__phosphate_biosynthesis_II_g__Bacteroides_s__Bacteroides_thetaiotaom
PWY_6124__inosine_5__phosphate_biosynthesis_II_g__Bacteroides_s__Bacteroides_xylanisolvens
PWY_6124__inosine_5__phosphate_biosynthesis_II_g__Citrobacter_s__Citrobacter_freundii
PWY_6124__inosine_5__phosphate_biosynthesis_II_g__Clostridium_s__Clostridium_perfringens
PWY_6124__inosine_5__phosphate_biosynthesis_II_g__Clostridium_s__Clostridium_sp_L2_50
PWY_6124__inosine_5__phosphate_biosynthesis_II_g__Coprococcus_s__Coprococcus_catus
PWY_6124__inosine_5__phosphate_biosynthesis_II_g__Coprococcus_s__Coprococcus_eutactus
PWY_6124__inosine_5__phosphate_biosynthesis_II_g__Coprococcus_s__Coprococcus_sp_ART55_
PWY_6124__inosine_5__phosphate_biosynthesis_II_g__Enterobacter_s__Enterobacter_cloacae
PWY_6124__inosine_5__phosphate_biosynthesis_II_g__Enterococcus_s__Enterococcus_faecium
PWY_6124__inosine_5__phosphate_biosynthesis_II_g__Eubacterium_s__Eubacterium_siraeum
PWY_6124__inosine_5__phosphate_biosynthesis_II_g__Fusobacterium_s__Fusobacterium_nuclea
PWY_6124__inosine_5__phosphate_biosynthesis_II_g__Klebsiella_s__Klebsiella_pneumoniae
PWY_6124__inosine_5__phosphate_biosynthesis_II_g__Lachnospiraceae_noname_s__Lachnospira
PWY_6124__inosine_5__phosphate_biosynthesis_II_g__Lactobacillus_s__Lactobacillus_casei_para
PWY_6124__inosine_5__phosphate_biosynthesis_II_g__Lactobacillus_s__Lactobacillus_rhamnosus
PWY_6124__inosine_5__phosphate_biosynthesis_II_g__Lactococcus_s__Lactococcus_lactis
PWY_6124__inosine_5__phosphate_biosynthesis_II_g__Ruminococcus_s__Ruminococcus_champa
PWY_6124__inosine_5__phosphate_biosynthesis_II_g__Streptococcus_s__Streptococcus_mitis_or
PWY_6125__superpathway_of_guanosine_nucleotides_de_novo_biosynthesis_II_g__Actinomyces_
PWY_6125__superpathway_of_guanosine_nucleotides_de_novo_biosynthesis_II_g__Bacteroides_
PWY_6125__superpathway_of_guanosine_nucleotides_de_novo_biosynthesis_II_g__Bacteroides_
PWY_6125__superpathway_of_guanosine_nucleotides_de_novo_biosynthesis_II_g__Bacteroides_
PWY_6125__superpathway_of_guanosine_nucleotides_de_novo_biosynthesis_II_g__Bacteroides_
PWY_6125__superpathway_of_guanosine_nucleotides_de_novo_biosynthesis_II_g__Bacteroides_
PWY_6125__superpathway_of_guanosine_nucleotides_de_novo_biosynthesis_II_g__Burkholderia
PWY_6125__superpathway_of_guanosine_nucleotides_de_novo_biosynthesis_II_g__Citrobacter_s
PWY_6125__superpathway_of_guanosine_nucleotides_de_novo_biosynthesis_II_g__Clostridium_
PWY_6125__superpathway_of_guanosine_nucleotides_de_novo_biosynthesis_II_g__Eggerthella_s
PWY_6125__superpathway_of_guanosine_nucleotides_de_novo_biosynthesis_II_g__Eggerthella_s

PWY_6125__superpathway_of_guanosine_nucleotides_de_novo_biosynthesis_II_g__Eggerthella_s
PWY_6125__superpathway_of_guanosine_nucleotides_de_novo_biosynthesis_II_g__Klebsiella_s_
PWY_6125__superpathway_of_guanosine_nucleotides_de_novo_biosynthesis_II_g__Parasutterell
PWY_6125__superpathway_of_guanosine_nucleotides_de_novo_biosynthesis_II_g__Streptococcu
PWY_6125__superpathway_of_guanosine_nucleotides_de_novo_biosynthesis_II_g__Streptococcu
PWY_6125__superpathway_of_guanosine_nucleotides_de_novo_biosynthesis_II_g__Veillonella_s
PWY_6125__superpathway_of_guanosine_nucleotides_de_novo_biosynthesis_II_g__Veillonella_s
PWY_6126__superpathway_of_adenosine_nucleotides_de_novo_biosynthesis_II_g__Bacteroides_
PWY_6126__superpathway_of_adenosine_nucleotides_de_novo_biosynthesis_II_g__Bacteroides_
PWY_6126__superpathway_of_adenosine_nucleotides_de_novo_biosynthesis_II_g__Bacteroides_
PWY_6126__superpathway_of_adenosine_nucleotides_de_novo_biosynthesis_II_g__Bacteroides_
PWY_6126__superpathway_of_adenosine_nucleotides_de_novo_biosynthesis_II_g__Bacteroides_
PWY_6126__superpathway_of_adenosine_nucleotides_de_novo_biosynthesis_II_g__Bacteroides_
PWY_6126__superpathway_of_adenosine_nucleotides_de_novo_biosynthesis_II_g__Citrobacter_s
PWY_6126__superpathway_of_adenosine_nucleotides_de_novo_biosynthesis_II_g__Clostridiales_
PWY_6126__superpathway_of_adenosine_nucleotides_de_novo_biosynthesis_II_g__Clostridium_
PWY_6126__superpathway_of_adenosine_nucleotides_de_novo_biosynthesis_II_g__Clostridium_
PWY_6126__superpathway_of_adenosine_nucleotides_de_novo_biosynthesis_II_g__Collinsella_s_
PWY_6126__superpathway_of_adenosine_nucleotides_de_novo_biosynthesis_II_g__Collinsella_s_
PWY_6126__superpathway_of_adenosine_nucleotides_de_novo_biosynthesis_II_g__Eggerthella_s
PWY_6126__superpathway_of_adenosine_nucleotides_de_novo_biosynthesis_II_g__Eggerthella_s
PWY_6126__superpathway_of_adenosine_nucleotides_de_novo_biosynthesis_II_g__Eggerthella_s
PWY_6126__superpathway_of_adenosine_nucleotides_de_novo_biosynthesis_II_g__Faecalibacter
PWY_6126__superpathway_of_adenosine_nucleotides_de_novo_biosynthesis_II_g__Holdemania_
PWY_6126__superpathway_of_adenosine_nucleotides_de_novo_biosynthesis_II_g__Klebsiella_s_
PWY_6126__superpathway_of_adenosine_nucleotides_de_novo_biosynthesis_II_g__Oxalobacter_
PWY_6126__superpathway_of_adenosine_nucleotides_de_novo_biosynthesis_II_g__Slackia_s__Sl
PWY_6126__superpathway_of_adenosine_nucleotides_de_novo_biosynthesis_II_g__Solobacteriu
PWY_6126__superpathway_of_adenosine_nucleotides_de_novo_biosynthesis_II_g__Streptococcu
PWY_6126__superpathway_of_adenosine_nucleotides_de_novo_biosynthesis_II_g__Streptococcu
PWY_6126__superpathway_of_adenosine_nucleotides_de_novo_biosynthesis_II_g__Veillonella_s
PWY_6126__superpathway_of_adenosine_nucleotides_de_novo_biosynthesis_II_g__Veillonella_s
PWY_6147__6_hydroxymethyl_dihydropterin_diphosphate_biosynthesis_I_g__Bacteroides_s__Ba
PWY_6147__6_hydroxymethyl_dihydropterin_diphosphate_biosynthesis_I_g__Bacteroides_s__Ba
PWY_6147__6_hydroxymethyl_dihydropterin_diphosphate_biosynthesis_I_g__Bacteroides_s__Ba
PWY_6147__6_hydroxymethyl_dihydropterin_diphosphate_biosynthesis_I_g__Bacteroides_s__Ba
PWY_6147__6_hydroxymethyl_dihydropterin_diphosphate_biosynthesis_I_g__Bacteroides_s__Ba
PWY_6147__6_hydroxymethyl_dihydropterin_diphosphate_biosynthesis_I_g__Bacteroides_s__Ba
PWY_6147__6_hydroxymethyl_dihydropterin_diphosphate_biosynthesis_I_g__Citrobacter_s__Citr
PWY_6147__6_hydroxymethyl_dihydropterin_diphosphate_biosynthesis_I_g__Streptococcus_s__S
PWY_6147__6_hydroxymethyl_dihydropterin_diphosphate_biosynthesis_I_g__Veillonella_s__Veil
PWY_6147__6_hydroxymethyl_dihydropterin_diphosphate_biosynthesis_I_g__Veillonella_s__Veil
PWY_6151__S_adenosyl_L_methionine_cycle_I_g__Actinomyces_s__Actinomyces_odontolyticus
PWY_6151__S_adenosyl_L_methionine_cycle_I_g__Anaerostipes_s__Anaerostipes_hadrus
PWY_6151__S_adenosyl_L_methionine_cycle_I_g__Anaerotruncus_s__Anaerotruncus_colihominis
PWY_6151__S_adenosyl_L_methionine_cycle_I_g__Bacteroides_s__Bacteroides_dorei
PWY_6151__S_adenosyl_L_methionine_cycle_I_g__Bacteroides_s__Bacteroides_eggerthii

PWY_6151__S_adenosyl_L_methionine_cycle_I_g__Bacteroides_s__Bacteroides_massiliensis
PWY_6151__S_adenosyl_L_methionine_cycle_I_g__Bacteroides_s__Bacteroides_sp_3_1_33FAA
PWY_6151__S_adenosyl_L_methionine_cycle_I_g__Bacteroides_s__Bacteroides_sp_3_1_40A
PWY_6151__S_adenosyl_L_methionine_cycle_I_g__Bacteroides_s__Bacteroides_sp_4_3_47FAA
PWY_6151__S_adenosyl_L_methionine_cycle_I_g__Bacteroides_s__Bacteroides_sp_9_1_42FAA
PWY_6151__S_adenosyl_L_methionine_cycle_I_g__Bacteroides_s__Bacteroides_sp_D20
PWY_6151__S_adenosyl_L_methionine_cycle_I_g__Bacteroides_s__Bacteroides_stercoris
PWY_6151__S_adenosyl_L_methionine_cycle_I_g__Bacteroides_s__Bacteroides_uniformis
PWY_6151__S_adenosyl_L_methionine_cycle_I_g__Barnesiella_s__Barnesiella_intestinihominis
PWY_6151__S_adenosyl_L_methionine_cycle_I_g__Bifidobacterium_s__Bifidobacterium_angulatu
PWY_6151__S_adenosyl_L_methionine_cycle_I_g__Bifidobacterium_s__Bifidobacterium_animalis
PWY_6151__S_adenosyl_L_methionine_cycle_I_g__Bifidobacterium_s__Bifidobacterium_dentium
PWY_6151__S_adenosyl_L_methionine_cycle_I_g__Blautia_s__Blautia_hydrogenotrophica
PWY_6151__S_adenosyl_L_methionine_cycle_I_g__Blautia_s__Blautia_sp_KLE_1732
PWY_6151__S_adenosyl_L_methionine_cycle_I_g__Blautia_s__Ruminococcus_gnavus
PWY_6151__S_adenosyl_L_methionine_cycle_I_g__Blautia_s__Ruminococcus_obeum
PWY_6151__S_adenosyl_L_methionine_cycle_I_g__Butyrivibrio_s__Butyrivibrio_crossotus
PWY_6151__S_adenosyl_L_methionine_cycle_I_g__Catenibacterium_s__Catenibacterium_mitsuol
PWY_6151__S_adenosyl_L_methionine_cycle_I_g__Citrobacter_s__Citrobacter_freundii
PWY_6151__S_adenosyl_L_methionine_cycle_I_g__Clostridiales_noname_s__Bacteroides_pectinc
PWY_6151__S_adenosyl_L_methionine_cycle_I_g__Clostridiales_noname_s__Clostridiales_bacter
PWY_6151__S_adenosyl_L_methionine_cycle_I_g__Clostridium_s__Clostridium_asparagiforme
PWY_6151__S_adenosyl_L_methionine_cycle_I_g__Clostridium_s__Clostridium_citroniae
PWY_6151__S_adenosyl_L_methionine_cycle_I_g__Clostridium_s__Clostridium_clostridioforme
PWY_6151__S_adenosyl_L_methionine_cycle_I_g__Clostridium_s__Clostridium_hathewayi
PWY_6151__S_adenosyl_L_methionine_cycle_I_g__Clostridium_s__Clostridium_leptum
PWY_6151__S_adenosyl_L_methionine_cycle_I_g__Clostridium_s__Clostridium_perfringens
PWY_6151__S_adenosyl_L_methionine_cycle_I_g__Clostridium_s__Clostridium_sp_L2_50
PWY_6151__S_adenosyl_L_methionine_cycle_I_g__Clostridium_s__Clostridium_sp_SS2_1
PWY_6151__S_adenosyl_L_methionine_cycle_I_g__Coprobacter_s__Coprobacter_fastidiosus
PWY_6151__S_adenosyl_L_methionine_cycle_I_g__Coprococcus_s__Coprococcus_catus
PWY_6151__S_adenosyl_L_methionine_cycle_I_g__Coprococcus_s__Coprococcus_eutactus
PWY_6151__S_adenosyl_L_methionine_cycle_I_g__Coprococcus_s__Coprococcus_sp_ART55_1
PWY_6151__S_adenosyl_L_methionine_cycle_I_g__Dorea_s__Dorea_formicigenerans
PWY_6151__S_adenosyl_L_methionine_cycle_I_g__Erysipelotrichaceae_noname_s__Clostridium_
PWY_6151__S_adenosyl_L_methionine_cycle_I_g__Erysipelotrichaceae_noname_s__Erysipelotricl
PWY_6151__S_adenosyl_L_methionine_cycle_I_g__Erysipelotrichaceae_noname_s__Eubacterium_
PWY_6151__S_adenosyl_L_methionine_cycle_I_g__Erysipelotrichaceae_noname_s__Eubacterium_
PWY_6151__S_adenosyl_L_methionine_cycle_I_g__Eubacterium_s__Eubacterium_eligens
PWY_6151__S_adenosyl_L_methionine_cycle_I_g__Eubacterium_s__Eubacterium_hallii
PWY_6151__S_adenosyl_L_methionine_cycle_I_g__Eubacterium_s__Eubacterium_sp_3_1_31
PWY_6151__S_adenosyl_L_methionine_cycle_I_g__Eubacterium_s__Eubacterium_ventriosum
PWY_6151__S_adenosyl_L_methionine_cycle_I_g__Fusobacterium_s__Fusobacterium_nucleatum
PWY_6151__S_adenosyl_L_methionine_cycle_I_g__Gemella_s__Gemella_sanguinis
PWY_6151__S_adenosyl_L_methionine_cycle_I_g__Klebsiella_s__Klebsiella_pneumoniae
PWY_6151__S_adenosyl_L_methionine_cycle_I_g__Lachnospiraceae_noname_s__Lachnospiracea
PWY_6151__S_adenosyl_L_methionine_cycle_I_g__Lachnospiraceae_noname_s__Lachnospiracea

PWY_6151__S_adenosyl_L_methionine_cycle_I_g__Lachnospiraceae_noname_s__Lachnospiracea
PWY_6151__S_adenosyl_L_methionine_cycle_I_g__Lactobacillus_s__Lactobacillus_casei_paracase
PWY_6151__S_adenosyl_L_methionine_cycle_I_g__Lactobacillus_s__Lactobacillus_fermentum
PWY_6151__S_adenosyl_L_methionine_cycle_I_g__Lactobacillus_s__Lactobacillus_rhamnosus
PWY_6151__S_adenosyl_L_methionine_cycle_I_g__Lactobacillus_s__Lactobacillus_ruminis
PWY_6151__S_adenosyl_L_methionine_cycle_I_g__Lactobacillus_s__Lactobacillus_salivarius
PWY_6151__S_adenosyl_L_methionine_cycle_I_g__Odoribacter_s__Odoribacter_laneus
PWY_6151__S_adenosyl_L_methionine_cycle_I_g__Odoribacter_s__Odoribacter_splanchnicus
PWY_6151__S_adenosyl_L_methionine_cycle_I_g__Paraprevotella_s__Paraprevotella_clara
PWY_6151__S_adenosyl_L_methionine_cycle_I_g__Peptostreptococcaceae_noname_s__Clostridiu
PWY_6151__S_adenosyl_L_methionine_cycle_I_g__Prevotella_s__Prevotella_copri
PWY_6151__S_adenosyl_L_methionine_cycle_I_g__Propionibacterium_s__Propionibacterium_freu
PWY_6151__S_adenosyl_L_methionine_cycle_I_g__Roseburia_s__Roseburia_inulinivorans
PWY_6151__S_adenosyl_L_methionine_cycle_I_g__Ruminococcus_s__Ruminococcus_callidus
PWY_6151__S_adenosyl_L_methionine_cycle_I_g__Ruminococcus_s__Ruminococcus_champanell
PWY_6151__S_adenosyl_L_methionine_cycle_I_g__Ruminococcus_s__Ruminococcus_sp
PWY_6151__S_adenosyl_L_methionine_cycle_I_g__Slackia_s__Slackia_piriformis
PWY_6151__S_adenosyl_L_methionine_cycle_I_g__Streptococcus_s__Streptococcus_australis
PWY_6151__S_adenosyl_L_methionine_cycle_I_g__Streptococcus_s__Streptococcus_mitis_oralis_
PWY_6151__S_adenosyl_L_methionine_cycle_I_g__Streptococcus_s__Streptococcus_sanguinis
PWY_6151__S_adenosyl_L_methionine_cycle_I_g__Subdoligranulum_s__Subdoligranulum_sp_4_
PWY_6163__chorismate_biosynthesis_from_3_dehydroquinate_g__Akkermansia_s__Akkermansia
PWY_6163__chorismate_biosynthesis_from_3_dehydroquinate_g__Alistipes_s__Alistipes_finegol
PWY_6163__chorismate_biosynthesis_from_3_dehydroquinate_g__Alistipes_s__Alistipes_shahii
PWY_6163__chorismate_biosynthesis_from_3_dehydroquinate_g__Alistipes_s__Alistipes_sp_HGE
PWY_6163__chorismate_biosynthesis_from_3_dehydroquinate_g__Anaerostipes_s__Anaerostipes
PWY_6163__chorismate_biosynthesis_from_3_dehydroquinate_g__Anaerotruncus_s__Anaerotrur
PWY_6163__chorismate_biosynthesis_from_3_dehydroquinate_g__Bacteroides_s__Bacteroides_f
PWY_6163__chorismate_biosynthesis_from_3_dehydroquinate_g__Bilophila_s__Bilophila_wadsw
PWY_6163__chorismate_biosynthesis_from_3_dehydroquinate_g__Blautia_s__Blautia_sp_KLE_17
PWY_6163__chorismate_biosynthesis_from_3_dehydroquinate_g__Blautia_s__Ruminococcus_gna
PWY_6163__chorismate_biosynthesis_from_3_dehydroquinate_g__Blautia_s__Ruminococcus_obe
PWY_6163__chorismate_biosynthesis_from_3_dehydroquinate_g__Burkholderiales_noname_s__
PWY_6163__chorismate_biosynthesis_from_3_dehydroquinate_g__Butyrivibrio_s__Butyrivibrio_c
PWY_6163__chorismate_biosynthesis_from_3_dehydroquinate_g__Citrobacter_s__Citrobacter_fr
PWY_6163__chorismate_biosynthesis_from_3_dehydroquinate_g__Clostridiales_noname_s__Clos
PWY_6163__chorismate_biosynthesis_from_3_dehydroquinate_g__Clostridium_s__Clostridium_a
PWY_6163__chorismate_biosynthesis_from_3_dehydroquinate_g__Clostridium_s__Clostridium_b
PWY_6163__chorismate_biosynthesis_from_3_dehydroquinate_g__Clostridium_s__Clostridium_c
PWY_6163__chorismate_biosynthesis_from_3_dehydroquinate_g__Clostridium_s__Clostridium_h
PWY_6163__chorismate_biosynthesis_from_3_dehydroquinate_g__Clostridium_s__Clostridium_s
PWY_6163__chorismate_biosynthesis_from_3_dehydroquinate_g__Clostridium_s__Clostridium_s
PWY_6163__chorismate_biosynthesis_from_3_dehydroquinate_g__Clostridium_s__Clostridium_s
PWY_6163__chorismate_biosynthesis_from_3_dehydroquinate_g__Coprococcus_s__Coprococcus
PWY_6163__chorismate_biosynthesis_from_3_dehydroquinate_g__Dorea_s__Dorea_formicigener
PWY_6163__chorismate_biosynthesis_from_3_dehydroquinate_g__Dorea_s__Dorea_longicatena
PWY_6163__chorismate_biosynthesis_from_3_dehydroquinate_g__Enterococcus_s__Enterococcu

PWY_6163__chorismate_biosynthesis_from_3_dehydroquinate_g__Erysipelotrichaceae_noname_
PWY_6163__chorismate_biosynthesis_from_3_dehydroquinate_g__Erysipelotrichaceae_noname_
PWY_6163__chorismate_biosynthesis_from_3_dehydroquinate_g__Eubacterium_s__Eubacterium_
PWY_6163__chorismate_biosynthesis_from_3_dehydroquinate_g__Eubacterium_s__Eubacterium_
PWY_6163__chorismate_biosynthesis_from_3_dehydroquinate_g__Eubacterium_s__Eubacterium_
PWY_6163__chorismate_biosynthesis_from_3_dehydroquinate_g__Eubacterium_s__Eubacterium_
PWY_6163__chorismate_biosynthesis_from_3_dehydroquinate_g__Fusobacterium_s__Fusobacter
PWY_6163__chorismate_biosynthesis_from_3_dehydroquinate_g__Klebsiella_s__Klebsiella_pneu
PWY_6163__chorismate_biosynthesis_from_3_dehydroquinate_g__Lachnospiraceae_noname_s__
PWY_6163__chorismate_biosynthesis_from_3_dehydroquinate_g__Lachnospiraceae_noname_s__
PWY_6163__chorismate_biosynthesis_from_3_dehydroquinate_g__Lachnospiraceae_noname_s__
PWY_6163__chorismate_biosynthesis_from_3_dehydroquinate_g__Lachnospiraceae_noname_s__
PWY_6163__chorismate_biosynthesis_from_3_dehydroquinate_g__Lachnospiraceae_noname_s__
PWY_6163__chorismate_biosynthesis_from_3_dehydroquinate_g__Lactobacillus_s__Lactobacillus
PWY_6163__chorismate_biosynthesis_from_3_dehydroquinate_g__Lactococcus_s__Lactococcus_l
PWY_6163__chorismate_biosynthesis_from_3_dehydroquinate_g__Parasutterella_s__Parasuttere
PWY_6163__chorismate_biosynthesis_from_3_dehydroquinate_g__Peptostreptococcaceae_nonar
PWY_6163__chorismate_biosynthesis_from_3_dehydroquinate_g__Phascolarctobacterium_s__Ph
PWY_6163__chorismate_biosynthesis_from_3_dehydroquinate_g__Propionibacterium_s__Propior
PWY_6163__chorismate_biosynthesis_from_3_dehydroquinate_g__Roseburia_s__Roseburia_hom
PWY_6163__chorismate_biosynthesis_from_3_dehydroquinate_g__Roseburia_s__Roseburia_inuli
PWY_6163__chorismate_biosynthesis_from_3_dehydroquinate_g__Ruminococcus_s__Ruminococ
PWY_6163__chorismate_biosynthesis_from_3_dehydroquinate_g__Ruminococcus_s__Ruminococ
PWY_6163__chorismate_biosynthesis_from_3_dehydroquinate_g__Ruminococcus_s__Ruminococ
PWY_6163__chorismate_biosynthesis_from_3_dehydroquinate_g__Streptococcus_s__Streptococc
PWY_6163__chorismate_biosynthesis_from_3_dehydroquinate_g__Streptococcus_s__Streptococc
PWY_6168__flavin_biosynthesis_III_(fungi)_g__Anaerostipes_s__Anaerostipes_hadrus
PWY_6168__flavin_biosynthesis_III_(fungi)_g__Bacteroides_s__Bacteroides_faecis
PWY_6168__flavin_biosynthesis_III_(fungi)_g__Bacteroides_s__Bacteroides_sp_1_1_6
PWY_6168__flavin_biosynthesis_III_(fungi)_g__Bacteroides_s__Bacteroides_thetaiotaomicron
PWY_6168__flavin_biosynthesis_III_(fungi)_g__Citrobacter_s__Citrobacter_freundii
PWY_6168__flavin_biosynthesis_III_(fungi)_g__Clostridium_s__Clostridium_sp_SS2_1
PWY_6168__flavin_biosynthesis_III_(fungi)_g__Lachnospiraceae_noname_s__Lachnospiraceae_ba
PWY_6185__4_methylcatechol_degradation_(ortho_cleavage)
PWY_621__sucrose_degradation_III_(sucrose_invertase)_g__Anaerostipes_s__Anaerostipes_hadru
PWY_621__sucrose_degradation_III_(sucrose_invertase)_g__Bifidobacterium_s__Bifidobacterium_
PWY_621__sucrose_degradation_III_(sucrose_invertase)_g__Blautia_s__Blautia_sp_KLE_1732
PWY_621__sucrose_degradation_III_(sucrose_invertase)_g__Blautia_s__Ruminococcus_obeum
PWY_621__sucrose_degradation_III_(sucrose_invertase)_g__Clostridiales_noname_s__Clostridiale
PWY_621__sucrose_degradation_III_(sucrose_invertase)_g__Clostridium_s__Clostridium_sp_L2_5
PWY_621__sucrose_degradation_III_(sucrose_invertase)_g__Clostridium_s__Clostridium_sp_SS2_
PWY_621__sucrose_degradation_III_(sucrose_invertase)_g__Coprococcus_s__Coprococcus_eutact
PWY_621__sucrose_degradation_III_(sucrose_invertase)_g__Coprococcus_s__Coprococcus_sp_AR
PWY_621__sucrose_degradation_III_(sucrose_invertase)_g__Enterococcus_s__Enterococcus_faeci
PWY_621__sucrose_degradation_III_(sucrose_invertase)_g__Klebsiella_s__Klebsiella_pneumoniae
PWY_621__sucrose_degradation_III_(sucrose_invertase)_g__Lachnospiraceae_noname_s__Lachno
PWY_621__sucrose_degradation_III_(sucrose_invertase)_g__Ruminococcus_s__Ruminococcus_sp

PWY_621__sucrose_degradation_III_(sucrose_invertase)_g__Streptococcus_s__Streptococcus_mit
PWY_622__starch_biosynthesis
PWY_6263__superpathway_of_menaquinol_8_biosynthesis_II
PWY_6270__isoprene_biosynthesis_I_g__Citrobacter_s__Citrobacter_freundii
PWY_6270__isoprene_biosynthesis_I_g__Klebsiella_s__Klebsiella_pneumoniae
PWY_6270__isoprene_biosynthesis_I_g__Phascolarctobacterium_s__Phascolarctobacterium_succi
PWY_6277__superpathway_of_5_aminoimidazole_ribonucleotide_biosynthesis_g__Adlercreutzia_
PWY_6277__superpathway_of_5_aminoimidazole_ribonucleotide_biosynthesis_g__Akkermansia_
PWY_6277__superpathway_of_5_aminoimidazole_ribonucleotide_biosynthesis_g__Alistipes_s__A
PWY_6277__superpathway_of_5_aminoimidazole_ribonucleotide_biosynthesis_g__Alistipes_s__A
PWY_6277__superpathway_of_5_aminoimidazole_ribonucleotide_biosynthesis_g__Alistipes_s__A
PWY_6277__superpathway_of_5_aminoimidazole_ribonucleotide_biosynthesis_g__Alistipes_s__A
PWY_6277__superpathway_of_5_aminoimidazole_ribonucleotide_biosynthesis_g__Anaerofustis_
PWY_6277__superpathway_of_5_aminoimidazole_ribonucleotide_biosynthesis_g__Anaerostipes_
PWY_6277__superpathway_of_5_aminoimidazole_ribonucleotide_biosynthesis_g__Anaerotruncu
PWY_6277__superpathway_of_5_aminoimidazole_ribonucleotide_biosynthesis_g__Bacteroides_
PWY_6277__superpathway_of_5_aminoimidazole_ribonucleotide_biosynthesis_g__Bifidobacteriu
PWY_6277__superpathway_of_5_aminoimidazole_ribonucleotide_biosynthesis_g__Bifidobacteriu
PWY_6277__superpathway_of_5_aminoimidazole_ribonucleotide_biosynthesis_g__Bifidobacteriu
PWY_6277__superpathway_of_5_aminoimidazole_ribonucleotide_biosynthesis_g__Bifidobacteriu
PWY_6277__superpathway_of_5_aminoimidazole_ribonucleotide_biosynthesis_g__Bifidobacteriu
PWY_6277__superpathway_of_5_aminoimidazole_ribonucleotide_biosynthesis_g__Bilophila_s__
PWY_6277__superpathway_of_5_aminoimidazole_ribonucleotide_biosynthesis_g__Blautia_s__Bl
PWY_6277__superpathway_of_5_aminoimidazole_ribonucleotide_biosynthesis_g__Blautia_s__Bl
PWY_6277__superpathway_of_5_aminoimidazole_ribonucleotide_biosynthesis_g__Blautia_s__Ru
PWY_6277__superpathway_of_5_aminoimidazole_ribonucleotide_biosynthesis_g__Blautia_s__Ru
PWY_6277__superpathway_of_5_aminoimidazole_ribonucleotide_biosynthesis_g__Blautia_s__Ru
PWY_6277__superpathway_of_5_aminoimidazole_ribonucleotide_biosynthesis_g__Burkholderial
PWY_6277__superpathway_of_5_aminoimidazole_ribonucleotide_biosynthesis_g__Butyrivibrio_s
PWY_6277__superpathway_of_5_aminoimidazole_ribonucleotide_biosynthesis_g__Catenibacteri
PWY_6277__superpathway_of_5_aminoimidazole_ribonucleotide_biosynthesis_g__Clostridiales_
PWY_6277__superpathway_of_5_aminoimidazole_ribonucleotide_biosynthesis_g__Clostridiales_
PWY_6277__superpathway_of_5_aminoimidazole_ribonucleotide_biosynthesis_g__Clostridium_s
PWY_6277__superpathway_of_5_aminoimidazole_ribonucleotide_biosynthesis_g__Clostridium_s
PWY_6277__superpathway_of_5_aminoimidazole_ribonucleotide_biosynthesis_g__Clostridium_s
PWY_6277__superpathway_of_5_aminoimidazole_ribonucleotide_biosynthesis_g__Clostridium_s
PWY_6277__superpathway_of_5_aminoimidazole_ribonucleotide_biosynthesis_g__Clostridium_s
PWY_6277__superpathway_of_5_aminoimidazole_ribonucleotide_biosynthesis_g__Clostridium_s
PWY_6277__superpathway_of_5_aminoimidazole_ribonucleotide_biosynthesis_g__Clostridium_s
PWY_6277__superpathway_of_5_aminoimidazole_ribonucleotide_biosynthesis_g__Collinsella_s__
PWY_6277__superpathway_of_5_aminoimidazole_ribonucleotide_biosynthesis_g__Collinsella_s__
PWY_6277__superpathway_of_5_aminoimidazole_ribonucleotide_biosynthesis_g__Coprococcus_
PWY_6277__superpathway_of_5_aminoimidazole_ribonucleotide_biosynthesis_g__Coprococcus_
PWY_6277__superpathway_of_5_aminoimidazole_ribonucleotide_biosynthesis_g__Dialister_s__D
PWY_6277__superpathway_of_5_aminoimidazole_ribonucleotide_biosynthesis_g__Dorea_s__Dor
PWY_6277__superpathway_of_5_aminoimidazole_ribonucleotide_biosynthesis_g__Eggerthella_s_
PWY_6277__superpathway_of_5_aminoimidazole_ribonucleotide_biosynthesis_g__Eggerthella_s_

PWY_6277__superpathway_of_5_aminoimidazole_ribonucleotide_biosynthesis_g__Eggerthella_s_
PWY_6277__superpathway_of_5_aminoimidazole_ribonucleotide_biosynthesis_g__Enterobacter_
PWY_6277__superpathway_of_5_aminoimidazole_ribonucleotide_biosynthesis_g__Enterococcus_
PWY_6277__superpathway_of_5_aminoimidazole_ribonucleotide_biosynthesis_g__Enterococcus_
PWY_6277__superpathway_of_5_aminoimidazole_ribonucleotide_biosynthesis_g__Enterococcus_
PWY_6277__superpathway_of_5_aminoimidazole_ribonucleotide_biosynthesis_g__Enterorhabdu
PWY_6277__superpathway_of_5_aminoimidazole_ribonucleotide_biosynthesis_g__Erysipelotricha
PWY_6277__superpathway_of_5_aminoimidazole_ribonucleotide_biosynthesis_g__Erysipelotricha
PWY_6277__superpathway_of_5_aminoimidazole_ribonucleotide_biosynthesis_g__Erysipelotricha
PWY_6277__superpathway_of_5_aminoimidazole_ribonucleotide_biosynthesis_g__Eubacterium_
PWY_6277__superpathway_of_5_aminoimidazole_ribonucleotide_biosynthesis_g__Eubacterium_
PWY_6277__superpathway_of_5_aminoimidazole_ribonucleotide_biosynthesis_g__Eubacterium_
PWY_6277__superpathway_of_5_aminoimidazole_ribonucleotide_biosynthesis_g__Eubacterium_
PWY_6277__superpathway_of_5_aminoimidazole_ribonucleotide_biosynthesis_g__Fusobacterium
PWY_6277__superpathway_of_5_aminoimidazole_ribonucleotide_biosynthesis_g__Gemella_s__G
PWY_6277__superpathway_of_5_aminoimidazole_ribonucleotide_biosynthesis_g__Holdemania_s
PWY_6277__superpathway_of_5_aminoimidazole_ribonucleotide_biosynthesis_g__Klebsiella_s__
PWY_6277__superpathway_of_5_aminoimidazole_ribonucleotide_biosynthesis_g__Lachnospirace
PWY_6277__superpathway_of_5_aminoimidazole_ribonucleotide_biosynthesis_g__Lachnospirace
PWY_6277__superpathway_of_5_aminoimidazole_ribonucleotide_biosynthesis_g__Lachnospirace
PWY_6277__superpathway_of_5_aminoimidazole_ribonucleotide_biosynthesis_g__Lachnospirace
PWY_6277__superpathway_of_5_aminoimidazole_ribonucleotide_biosynthesis_g__Lachnospirace
PWY_6277__superpathway_of_5_aminoimidazole_ribonucleotide_biosynthesis_g__Lachnospirace
PWY_6277__superpathway_of_5_aminoimidazole_ribonucleotide_biosynthesis_g__Lactobacillus_
PWY_6277__superpathway_of_5_aminoimidazole_ribonucleotide_biosynthesis_g__Lactobacillus_
PWY_6277__superpathway_of_5_aminoimidazole_ribonucleotide_biosynthesis_g__Lactobacillus_
PWY_6277__superpathway_of_5_aminoimidazole_ribonucleotide_biosynthesis_g__Odoribacter_s
PWY_6277__superpathway_of_5_aminoimidazole_ribonucleotide_biosynthesis_g__Odoribacter_s
PWY_6277__superpathway_of_5_aminoimidazole_ribonucleotide_biosynthesis_g__Oxalobacter_s
PWY_6277__superpathway_of_5_aminoimidazole_ribonucleotide_biosynthesis_g__Parasutterella_
PWY_6277__superpathway_of_5_aminoimidazole_ribonucleotide_biosynthesis_g__Pediococcus_s
PWY_6277__superpathway_of_5_aminoimidazole_ribonucleotide_biosynthesis_g__Pediococcus_s
PWY_6277__superpathway_of_5_aminoimidazole_ribonucleotide_biosynthesis_g__Peptostreptoc
PWY_6277__superpathway_of_5_aminoimidazole_ribonucleotide_biosynthesis_g__Phascolarctob
PWY_6277__superpathway_of_5_aminoimidazole_ribonucleotide_biosynthesis_g__Propionibacte
PWY_6277__superpathway_of_5_aminoimidazole_ribonucleotide_biosynthesis_g__Roseburia_s__
PWY_6277__superpathway_of_5_aminoimidazole_ribonucleotide_biosynthesis_g__Roseburia_s__
PWY_6277__superpathway_of_5_aminoimidazole_ribonucleotide_biosynthesis_g__Ruminococcac
PWY_6277__superpathway_of_5_aminoimidazole_ribonucleotide_biosynthesis_g__Ruminococcus
PWY_6277__superpathway_of_5_aminoimidazole_ribonucleotide_biosynthesis_g__Ruminococcus
PWY_6277__superpathway_of_5_aminoimidazole_ribonucleotide_biosynthesis_g__Ruminococcus
PWY_6277__superpathway_of_5_aminoimidazole_ribonucleotide_biosynthesis_g__Slackia_s__Sla
PWY_6277__superpathway_of_5_aminoimidazole_ribonucleotide_biosynthesis_g__Solobacterium
PWY_6277__superpathway_of_5_aminoimidazole_ribonucleotide_biosynthesis_g__Streptococcus_
PWY_6277__superpathway_of_5_aminoimidazole_ribonucleotide_biosynthesis_g__Subdoligranulu
PWY_6277__superpathway_of_5_aminoimidazole_ribonucleotide_biosynthesis_g__Veillonella_s_
PWY_6277__superpathway_of_5_aminoimidazole_ribonucleotide_biosynthesis_g__Veillonella_s_

PWY_6282__palmitoleate_biosynthesis_I_(from_(5Z)_dodec_5_enoate)_g__Bacteroides_s__Bacte
PWY_6282__palmitoleate_biosynthesis_I_(from_(5Z)_dodec_5_enoate)_g__Bacteroides_s__Bacte
PWY_6282__palmitoleate_biosynthesis_I_(from_(5Z)_dodec_5_enoate)_g__Citrobacter_s__Citrob
PWY_6282__palmitoleate_biosynthesis_I_(from_(5Z)_dodec_5_enoate)_g__Enterobacter_s__Ente
PWY_6282__palmitoleate_biosynthesis_I_(from_(5Z)_dodec_5_enoate)_g__Haemophilus_s__Hae
PWY_6282__palmitoleate_biosynthesis_I_(from_(5Z)_dodec_5_enoate)_g__Klebsiella_s__Klebsiel
PWY_6285__superpathway_of_fatty_acids_biosynthesis_(E__coli)_unclassified
PWY_6305__putrescine_biosynthesis_IV_g__Blautia_s__Ruminococcus_obeum
PWY_6305__putrescine_biosynthesis_IV_g__Citrobacter_s__Citrobacter_freundii
PWY_6305__putrescine_biosynthesis_IV_g__Enterobacter_s__Enterobacter_cloacae
PWY_6305__putrescine_biosynthesis_IV_g__Faecalibacterium_s__Faecalibacterium_prausnitzii
PWY_6305__putrescine_biosynthesis_IV_g__Klebsiella_s__Klebsiella_pneumoniae
PWY_6305__putrescine_biosynthesis_IV_g__Odoribacter_s__Odoribacter_splanchnicus
PWY_6305__putrescine_biosynthesis_IV_g__Ruminococcus_s__Ruminococcus_champanellensis
PWY_6307__L_tryptophan_degradation_X_(mammalian,_via_tryptamine)
PWY_6313__serotonin_degradation
PWY_6317__galactose_degradation_I_(Leloir_pathway)_g__Anaerostipes_s__Anaerostipes_hadru
PWY_6317__galactose_degradation_I_(Leloir_pathway)_g__Blautia_s__Blautia_sp_KLE_1732
PWY_6317__galactose_degradation_I_(Leloir_pathway)_g__Blautia_s__Ruminococcus_obeum
PWY_6317__galactose_degradation_I_(Leloir_pathway)_g__Citrobacter_s__Citrobacter_freundii
PWY_6317__galactose_degradation_I_(Leloir_pathway)_g__Clostridium_s__Clostridium_asparagif
PWY_6317__galactose_degradation_I_(Leloir_pathway)_g__Clostridium_s__Clostridium_sp_SS2_1
PWY_6317__galactose_degradation_I_(Leloir_pathway)_g__Enterococcus_s__Enterococcus_duran
PWY_6317__galactose_degradation_I_(Leloir_pathway)_g__Enterococcus_s__Enterococcus_faeciu
PWY_6317__galactose_degradation_I_(Leloir_pathway)_g__Eubacterium_s__Eubacterium_siraeur
PWY_6317__galactose_degradation_I_(Leloir_pathway)_g__Fusobacterium_s__Fusobacterium_nu
PWY_6317__galactose_degradation_I_(Leloir_pathway)_g__Lachnospiraceae_noname_s__Lachnos
PWY_6317__galactose_degradation_I_(Leloir_pathway)_g__Lachnospiraceae_noname_s__Lachnos
PWY_6317__galactose_degradation_I_(Leloir_pathway)_g__Lactococcus_s__Lactococcus_lactis
PWY_6317__galactose_degradation_I_(Leloir_pathway)_g__Ruminococcus_s__Ruminococcus_sp
PWY_6317__galactose_degradation_I_(Leloir_pathway)_g__Streptococcus_s__Streptococcus_para
PWY_6317__galactose_degradation_I_(Leloir_pathway)_g__Streptococcus_s__Streptococcus_sang
PWY_6385__peptidoglycan_biosynthesis_III_(mycobacteria)_g__Anaerofustis_s__Anaerofustis_ste
PWY_6385__peptidoglycan_biosynthesis_III_(mycobacteria)_g__Bacteroides_s__Bacteroides_fineg
PWY_6385__peptidoglycan_biosynthesis_III_(mycobacteria)_g__Bacteroides_s__Bacteroides_ovat
PWY_6385__peptidoglycan_biosynthesis_III_(mycobacteria)_g__Bacteroides_s__Bacteroides_sp_1
PWY_6385__peptidoglycan_biosynthesis_III_(mycobacteria)_g__Bacteroides_s__Bacteroides_sp_2
PWY_6385__peptidoglycan_biosynthesis_III_(mycobacteria)_g__Bacteroides_s__Bacteroides_sp_3
PWY_6385__peptidoglycan_biosynthesis_III_(mycobacteria)_g__Bacteroides_s__Bacteroides_sp_3
PWY_6385__peptidoglycan_biosynthesis_III_(mycobacteria)_g__Bacteroides_s__Bacteroides_sp_D
PWY_6385__peptidoglycan_biosynthesis_III_(mycobacteria)_g__Bacteroides_s__Bacteroides_xylan
PWY_6385__peptidoglycan_biosynthesis_III_(mycobacteria)_g__Bifidobacterium_s__Bifidobacteri
PWY_6385__peptidoglycan_biosynthesis_III_(mycobacteria)_g__Citrobacter_s__Citrobacter_freun
PWY_6385__peptidoglycan_biosynthesis_III_(mycobacteria)_g__Clostridium_s__Clostridium_perfr
PWY_6385__peptidoglycan_biosynthesis_III_(mycobacteria)_g__Eggerthella_s__Eggerthella_lenta
PWY_6385__peptidoglycan_biosynthesis_III_(mycobacteria)_g__Eggerthella_s__Eggerthella_sp_1_
PWY_6385__peptidoglycan_biosynthesis_III_(mycobacteria)_g__Eggerthella_s__Eggerthella_sp_H

PWY_6385__peptidoglycan_biosynthesis_III_(mycobacteria)_g__Enterobacter_s__Enterobacter_cl
PWY_6385__peptidoglycan_biosynthesis_III_(mycobacteria)_g__Eubacterium_s__Eubacterium_ve
PWY_6385__peptidoglycan_biosynthesis_III_(mycobacteria)_g__Lactobacillus_s__Lactobacillus_ac
PWY_6385__peptidoglycan_biosynthesis_III_(mycobacteria)_g__Odoribacter_s__Odoribacter_spla
PWY_6385__peptidoglycan_biosynthesis_III_(mycobacteria)_g__Parabacteroides_s__Parabacteroi
PWY_6385__peptidoglycan_biosynthesis_III_(mycobacteria)_g__Parabacteroides_s__Parabacteroi
PWY_6385__peptidoglycan_biosynthesis_III_(mycobacteria)_g__Parabacteroides_s__Parabacteroi
PWY_6385__peptidoglycan_biosynthesis_III_(mycobacteria)_g__Phascolarctobacterium_s__Phasco
PWY_6385__peptidoglycan_biosynthesis_III_(mycobacteria)_g__Streptococcus_s__Streptococcus_
PWY_6385__peptidoglycan_biosynthesis_III_(mycobacteria)_g__Veillonella_s__Veillonella_parvula
PWY_6386__UDP_N_acetylmuramoyl_pentapeptide_biosynthesis_II_(lysine_containing)_g__Adler
PWY_6386__UDP_N_acetylmuramoyl_pentapeptide_biosynthesis_II_(lysine_containing)_g__Alisti
PWY_6386__UDP_N_acetylmuramoyl_pentapeptide_biosynthesis_II_(lysine_containing)_g__Alisti
PWY_6386__UDP_N_acetylmuramoyl_pentapeptide_biosynthesis_II_(lysine_containing)_g__Alisti
PWY_6386__UDP_N_acetylmuramoyl_pentapeptide_biosynthesis_II_(lysine_containing)_g__Alisti
PWY_6386__UDP_N_acetylmuramoyl_pentapeptide_biosynthesis_II_(lysine_containing)_g__Anae
PWY_6386__UDP_N_acetylmuramoyl_pentapeptide_biosynthesis_II_(lysine_containing)_g__Anae
PWY_6386__UDP_N_acetylmuramoyl_pentapeptide_biosynthesis_II_(lysine_containing)_g__Anae
PWY_6386__UDP_N_acetylmuramoyl_pentapeptide_biosynthesis_II_(lysine_containing)_g__Bacte
PWY_6386__UDP_N_acetylmuramoyl_pentapeptide_biosynthesis_II_(lysine_containing)_g__Bacte
PWY_6386__UDP_N_acetylmuramoyl_pentapeptide_biosynthesis_II_(lysine_containing)_g__Bacte
PWY_6386__UDP_N_acetylmuramoyl_pentapeptide_biosynthesis_II_(lysine_containing)_g__Bacte
PWY_6386__UDP_N_acetylmuramoyl_pentapeptide_biosynthesis_II_(lysine_containing)_g__Bacte
PWY_6386__UDP_N_acetylmuramoyl_pentapeptide_biosynthesis_II_(lysine_containing)_g__Bacte
PWY_6386__UDP_N_acetylmuramoyl_pentapeptide_biosynthesis_II_(lysine_containing)_g__Bacte
PWY_6386__UDP_N_acetylmuramoyl_pentapeptide_biosynthesis_II_(lysine_containing)_g__Bacte
PWY_6386__UDP_N_acetylmuramoyl_pentapeptide_biosynthesis_II_(lysine_containing)_g__Bacte
PWY_6386__UDP_N_acetylmuramoyl_pentapeptide_biosynthesis_II_(lysine_containing)_g__Bacte
PWY_6386__UDP_N_acetylmuramoyl_pentapeptide_biosynthesis_II_(lysine_containing)_g__Bacte
PWY_6386__UDP_N_acetylmuramoyl_pentapeptide_biosynthesis_II_(lysine_containing)_g__Bacte
PWY_6386__UDP_N_acetylmuramoyl_pentapeptide_biosynthesis_II_(lysine_containing)_g__Barne
PWY_6386__UDP_N_acetylmuramoyl_pentapeptide_biosynthesis_II_(lysine_containing)_g__Bifido
PWY_6386__UDP_N_acetylmuramoyl_pentapeptide_biosynthesis_II_(lysine_containing)_g__Bifido
PWY_6386__UDP_N_acetylmuramoyl_pentapeptide_biosynthesis_II_(lysine_containing)_g__Bifido
PWY_6386__UDP_N_acetylmuramoyl_pentapeptide_biosynthesis_II_(lysine_containing)_g__Bifido
PWY_6386__UDP_N_acetylmuramoyl_pentapeptide_biosynthesis_II_(lysine_containing)_g__Bilop
PWY_6386__UDP_N_acetylmuramoyl_pentapeptide_biosynthesis_II_(lysine_containing)_g__Blaut
PWY_6386__UDP_N_acetylmuramoyl_pentapeptide_biosynthesis_II_(lysine_containing)_g__Blaut
PWY_6386__UDP_N_acetylmuramoyl_pentapeptide_biosynthesis_II_(lysine_containing)_g__Blaut
PWY_6386__UDP_N_acetylmuramoyl_pentapeptide_biosynthesis_II_(lysine_containing)_g__Blaut
PWY_6386__UDP_N_acetylmuramoyl_pentapeptide_biosynthesis_II_(lysine_containing)_g__Burkh
PWY_6386__UDP_N_acetylmuramoyl_pentapeptide_biosynthesis_II_(lysine_containing)_g__Butyr
PWY_6386__UDP_N_acetylmuramoyl_pentapeptide_biosynthesis_II_(lysine_containing)_g__Citrob
PWY_6386__UDP_N_acetylmuramoyl_pentapeptide_biosynthesis_II_(lysine_containing)_g__Clost
PWY_6386__UDP_N_acetylmuramoyl_pentapeptide_biosynthesis_II_(lysine_containing)_g__Clost
PWY_6386__UDP_N_acetylmuramoyl_pentapeptide_biosynthesis_II_(lysine_containing)_g__Clost
PWY_6386__UDP_N_acetylmuramoyl_pentapeptide_biosynthesis_II_(lysine_containing)_g__Clost

PWY_6386__UDP_N_acetylmuramoyl_pentapeptide_biosynthesis_II_(lysine_containing)_g__Clost
PWY_6386__UDP_N_acetylmuramoyl_pentapeptide_biosynthesis_II_(lysine_containing)_g__Collir
PWY_6386__UDP_N_acetylmuramoyl_pentapeptide_biosynthesis_II_(lysine_containing)_g__Collir
PWY_6386__UDP_N_acetylmuramoyl_pentapeptide_biosynthesis_II_(lysine_containing)_g__Copro
PWY_6386__UDP_N_acetylmuramoyl_pentapeptide_biosynthesis_II_(lysine_containing)_g__Copro
PWY_6386__UDP_N_acetylmuramoyl_pentapeptide_biosynthesis_II_(lysine_containing)_g__Dorea
PWY_6386__UDP_N_acetylmuramoyl_pentapeptide_biosynthesis_II_(lysine_containing)_g__Egger
PWY_6386__UDP_N_acetylmuramoyl_pentapeptide_biosynthesis_II_(lysine_containing)_g__Egger
PWY_6386__UDP_N_acetylmuramoyl_pentapeptide_biosynthesis_II_(lysine_containing)_g__Egger
PWY_6386__UDP_N_acetylmuramoyl_pentapeptide_biosynthesis_II_(lysine_containing)_g__Enter
PWY_6386__UDP_N_acetylmuramoyl_pentapeptide_biosynthesis_II_(lysine_containing)_g__Enter
PWY_6386__UDP_N_acetylmuramoyl_pentapeptide_biosynthesis_II_(lysine_containing)_g__Enter
PWY_6386__UDP_N_acetylmuramoyl_pentapeptide_biosynthesis_II_(lysine_containing)_g__Erysip
PWY_6386__UDP_N_acetylmuramoyl_pentapeptide_biosynthesis_II_(lysine_containing)_g__Erysip
PWY_6386__UDP_N_acetylmuramoyl_pentapeptide_biosynthesis_II_(lysine_containing)_g__Erysip
PWY_6386__UDP_N_acetylmuramoyl_pentapeptide_biosynthesis_II_(lysine_containing)_g__Euba
PWY_6386__UDP_N_acetylmuramoyl_pentapeptide_biosynthesis_II_(lysine_containing)_g__Euba
PWY_6386__UDP_N_acetylmuramoyl_pentapeptide_biosynthesis_II_(lysine_containing)_g__Euba
PWY_6386__UDP_N_acetylmuramoyl_pentapeptide_biosynthesis_II_(lysine_containing)_g__Euba
PWY_6386__UDP_N_acetylmuramoyl_pentapeptide_biosynthesis_II_(lysine_containing)_g__Lachr
PWY_6386__UDP_N_acetylmuramoyl_pentapeptide_biosynthesis_II_(lysine_containing)_g__Lachr
PWY_6386__UDP_N_acetylmuramoyl_pentapeptide_biosynthesis_II_(lysine_containing)_g__Lachr
PWY_6386__UDP_N_acetylmuramoyl_pentapeptide_biosynthesis_II_(lysine_containing)_g__Lachr
PWY_6386__UDP_N_acetylmuramoyl_pentapeptide_biosynthesis_II_(lysine_containing)_g__Lachr
PWY_6386__UDP_N_acetylmuramoyl_pentapeptide_biosynthesis_II_(lysine_containing)_g__Lachr
PWY_6386__UDP_N_acetylmuramoyl_pentapeptide_biosynthesis_II_(lysine_containing)_g__Lachr
PWY_6386__UDP_N_acetylmuramoyl_pentapeptide_biosynthesis_II_(lysine_containing)_g__Lachr
PWY_6386__UDP_N_acetylmuramoyl_pentapeptide_biosynthesis_II_(lysine_containing)_g__Lacto
PWY_6386__UDP_N_acetylmuramoyl_pentapeptide_biosynthesis_II_(lysine_containing)_g__Lacto
PWY_6386__UDP_N_acetylmuramoyl_pentapeptide_biosynthesis_II_(lysine_containing)_g__Lacto
PWY_6386__UDP_N_acetylmuramoyl_pentapeptide_biosynthesis_II_(lysine_containing)_g__Odor
PWY_6386__UDP_N_acetylmuramoyl_pentapeptide_biosynthesis_II_(lysine_containing)_g__Odor
PWY_6386__UDP_N_acetylmuramoyl_pentapeptide_biosynthesis_II_(lysine_containing)_g__Oxalo
PWY_6386__UDP_N_acetylmuramoyl_pentapeptide_biosynthesis_II_(lysine_containing)_g__Parab
PWY_6386__UDP_N_acetylmuramoyl_pentapeptide_biosynthesis_II_(lysine_containing)_g__Parab
PWY_6386__UDP_N_acetylmuramoyl_pentapeptide_biosynthesis_II_(lysine_containing)_g__Parab
PWY_6386__UDP_N_acetylmuramoyl_pentapeptide_biosynthesis_II_(lysine_containing)_g__Parab
PWY_6386__UDP_N_acetylmuramoyl_pentapeptide_biosynthesis_II_(lysine_containing)_g__Parab
PWY_6386__UDP_N_acetylmuramoyl_pentapeptide_biosynthesis_II_(lysine_containing)_g__Parab
PWY_6386__UDP_N_acetylmuramoyl_pentapeptide_biosynthesis_II_(lysine_containing)_g__Parap
PWY_6386__UDP_N_acetylmuramoyl_pentapeptide_biosynthesis_II_(lysine_containing)_g__Paras
PWY_6386__UDP_N_acetylmuramoyl_pentapeptide_biosynthesis_II_(lysine_containing)_g__Pedic
PWY_6386__UDP_N_acetylmuramoyl_pentapeptide_biosynthesis_II_(lysine_containing)_g__Pedic
PWY_6386__UDP_N_acetylmuramoyl_pentapeptide_biosynthesis_II_(lysine_containing)_g__Pepto
PWY_6386__UDP_N_acetylmuramoyl_pentapeptide_biosynthesis_II_(lysine_containing)_g__Phaso
PWY_6386__UDP_N_acetylmuramoyl_pentapeptide_biosynthesis_II_(lysine_containing)_g__Rosel
PWY_6386__UDP_N_acetylmuramoyl_pentapeptide_biosynthesis_II_(lysine_containing)_g__Rumi

PWY_6386__UDP_N_acetylmuramoyl_pentapeptide_biosynthesis_II_(lysine_containing)_g__Slack
PWY_6386__UDP_N_acetylmuramoyl_pentapeptide_biosynthesis_II_(lysine_containing)_g__Strep
PWY_6386__UDP_N_acetylmuramoyl_pentapeptide_biosynthesis_II_(lysine_containing)_g__Strep
PWY_6386__UDP_N_acetylmuramoyl_pentapeptide_biosynthesis_II_(lysine_containing)_g__Strep
PWY_6386__UDP_N_acetylmuramoyl_pentapeptide_biosynthesis_II_(lysine_containing)_g__Subd
PWY_6386__UDP_N_acetylmuramoyl_pentapeptide_biosynthesis_II_(lysine_containing)_g__Veill
PWY_6386__UDP_N_acetylmuramoyl_pentapeptide_biosynthesis_II_(lysine_containing)_g__Veill
PWY_6387__UDP_N_acetylmuramoyl_pentapeptide_biosynthesis_I_(meso_diaminopimelate_con
PWY_6387__UDP_N_acetylmuramoyl_pentapeptide_biosynthesis_I_(meso_diaminopimelate_con
PWY_6387__UDP_N_acetylmuramoyl_pentapeptide_biosynthesis_I_(meso_diaminopimelate_con
PWY_6387__UDP_N_acetylmuramoyl_pentapeptide_biosynthesis_I_(meso_diaminopimelate_con
PWY_6387__UDP_N_acetylmuramoyl_pentapeptide_biosynthesis_I_(meso_diaminopimelate_con
PWY_6387__UDP_N_acetylmuramoyl_pentapeptide_biosynthesis_I_(meso_diaminopimelate_con
PWY_6387__UDP_N_acetylmuramoyl_pentapeptide_biosynthesis_I_(meso_diaminopimelate_con
PWY_6387__UDP_N_acetylmuramoyl_pentapeptide_biosynthesis_I_(meso_diaminopimelate_con
PWY_6387__UDP_N_acetylmuramoyl_pentapeptide_biosynthesis_I_(meso_diaminopimelate_con
PWY_6387__UDP_N_acetylmuramoyl_pentapeptide_biosynthesis_I_(meso_diaminopimelate_con
PWY_6387__UDP_N_acetylmuramoyl_pentapeptide_biosynthesis_I_(meso_diaminopimelate_con
PWY_6387__UDP_N_acetylmuramoyl_pentapeptide_biosynthesis_I_(meso_diaminopimelate_con
PWY_6387__UDP_N_acetylmuramoyl_pentapeptide_biosynthesis_I_(meso_diaminopimelate_con
PWY_6387__UDP_N_acetylmuramoyl_pentapeptide_biosynthesis_I_(meso_diaminopimelate_con
PWY_6387__UDP_N_acetylmuramoyl_pentapeptide_biosynthesis_I_(meso_diaminopimelate_con
PWY_6387__UDP_N_acetylmuramoyl_pentapeptide_biosynthesis_I_(meso_diaminopimelate_con
PWY_6387__UDP_N_acetylmuramoyl_pentapeptide_biosynthesis_I_(meso_diaminopimelate_con
PWY_6387__UDP_N_acetylmuramoyl_pentapeptide_biosynthesis_I_(meso_diaminopimelate_con
PWY_6387__UDP_N_acetylmuramoyl_pentapeptide_biosynthesis_I_(meso_diaminopimelate_con
PWY_6387__UDP_N_acetylmuramoyl_pentapeptide_biosynthesis_I_(meso_diaminopimelate_con
PWY_6387__UDP_N_acetylmuramoyl_pentapeptide_biosynthesis_I_(meso_diaminopimelate_con
PWY_6387__UDP_N_acetylmuramoyl_pentapeptide_biosynthesis_I_(meso_diaminopimelate_con
PWY_6387__UDP_N_acetylmuramoyl_pentapeptide_biosynthesis_I_(meso_diaminopimelate_con
PWY_6387__UDP_N_acetylmuramoyl_pentapeptide_biosynthesis_I_(meso_diaminopimelate_con
PWY_6387__UDP_N_acetylmuramoyl_pentapeptide_biosynthesis_I_(meso_diaminopimelate_con
PWY_6387__UDP_N_acetylmuramoyl_pentapeptide_biosynthesis_I_(meso_diaminopimelate_con
PWY_6387__UDP_N_acetylmuramoyl_pentapeptide_biosynthesis_I_(meso_diaminopimelate_con
PWY_6387__UDP_N_acetylmuramoyl_pentapeptide_biosynthesis_I_(meso_diaminopimelate_con
PWY_6387__UDP_N_acetylmuramoyl_pentapeptide_biosynthesis_I_(meso_diaminopimelate_con
PWY_6387__UDP_N_acetylmuramoyl_pentapeptide_biosynthesis_I_(meso_diaminopimelate_con
PWY_6387__UDP_N_acetylmuramoyl_pentapeptide_biosynthesis_I_(meso_diaminopimelate_con
PWY_6387__UDP_N_acetylmuramoyl_pentapeptide_biosynthesis_I_(meso_diaminopimelate_con
PWY_6387__UDP_N_acetylmuramoyl_pentapeptide_biosynthesis_I_(meso_diaminopimelate_con
PWY_6387__UDP_N_acetylmuramoyl_pentapeptide_biosynthesis_I_(meso_diaminopimelate_con
PWY_6387__UDP_N_acetylmuramoyl_pentapeptide_biosynthesis_I_(meso_diaminopimelate_con
PWY_6387__UDP_N_acetylmuramoyl_pentapeptide_biosynthesis_I_(meso_diaminopimelate_con
PWY_6387__UDP_N_acetylmuramoyl_pentapeptide_biosynthesis_I_(meso_diaminopimelate_con
PWY_6387__UDP_N_acetylmuramoyl_pentapeptide_biosynthesis_I_(meso_diaminopimelate_con
PWY_6387__UDP_N_acetylmuramoyl_pentapeptide_biosynthesis_I_(meso_diaminopimelate_con
PWY_6387__UDP_N_acetylmuramoyl_pentapeptide_biosynthesis_I_(meso_diaminopimelate_con

PWY_6387__UDP_N_acetylmuramoyl_pentapeptide_biosynthesis_I_(meso_diaminopimelate_con
PWY_6387__UDP_N_acetylmuramoyl_pentapeptide_biosynthesis_I_(meso_diaminopimelate_con
PWY_6387__UDP_N_acetylmuramoyl_pentapeptide_biosynthesis_I_(meso_diaminopimelate_con
PWY_6387__UDP_N_acetylmuramoyl_pentapeptide_biosynthesis_I_(meso_diaminopimelate_con
PWY_6387__UDP_N_acetylmuramoyl_pentapeptide_biosynthesis_I_(meso_diaminopimelate_con
PWY_6387__UDP_N_acetylmuramoyl_pentapeptide_biosynthesis_I_(meso_diaminopimelate_con
PWY_6387__UDP_N_acetylmuramoyl_pentapeptide_biosynthesis_I_(meso_diaminopimelate_con
PWY_6387__UDP_N_acetylmuramoyl_pentapeptide_biosynthesis_I_(meso_diaminopimelate_con
PWY_6387__UDP_N_acetylmuramoyl_pentapeptide_biosynthesis_I_(meso_diaminopimelate_con
PWY_6387__UDP_N_acetylmuramoyl_pentapeptide_biosynthesis_I_(meso_diaminopimelate_con
PWY_6387__UDP_N_acetylmuramoyl_pentapeptide_biosynthesis_I_(meso_diaminopimelate_con
PWY_6387__UDP_N_acetylmuramoyl_pentapeptide_biosynthesis_I_(meso_diaminopimelate_con
PWY_6387__UDP_N_acetylmuramoyl_pentapeptide_biosynthesis_I_(meso_diaminopimelate_con
PWY_6387__UDP_N_acetylmuramoyl_pentapeptide_biosynthesis_I_(meso_diaminopimelate_con
PWY_6387__UDP_N_acetylmuramoyl_pentapeptide_biosynthesis_I_(meso_diaminopimelate_con
PWY_6387__UDP_N_acetylmuramoyl_pentapeptide_biosynthesis_I_(meso_diaminopimelate_con
PWY_6387__UDP_N_acetylmuramoyl_pentapeptide_biosynthesis_I_(meso_diaminopimelate_con
PWY_6387__UDP_N_acetylmuramoyl_pentapeptide_biosynthesis_I_(meso_diaminopimelate_con
PWY_6387__UDP_N_acetylmuramoyl_pentapeptide_biosynthesis_I_(meso_diaminopimelate_con
PWY_6387__UDP_N_acetylmuramoyl_pentapeptide_biosynthesis_I_(meso_diaminopimelate_con
PWY_6387__UDP_N_acetylmuramoyl_pentapeptide_biosynthesis_I_(meso_diaminopimelate_con
PWY_6387__UDP_N_acetylmuramoyl_pentapeptide_biosynthesis_I_(meso_diaminopimelate_con
PWY_6387__UDP_N_acetylmuramoyl_pentapeptide_biosynthesis_I_(meso_diaminopimelate_con
PWY_6387__UDP_N_acetylmuramoyl_pentapeptide_biosynthesis_I_(meso_diaminopimelate_con
PWY_6387__UDP_N_acetylmuramoyl_pentapeptide_biosynthesis_I_(meso_diaminopimelate_con
PWY_6387__UDP_N_acetylmuramoyl_pentapeptide_biosynthesis_I_(meso_diaminopimelate_con
PWY_6387__UDP_N_acetylmuramoyl_pentapeptide_biosynthesis_I_(meso_diaminopimelate_con
PWY_6387__UDP_N_acetylmuramoyl_pentapeptide_biosynthesis_I_(meso_diaminopimelate_con
PWY_6387__UDP_N_acetylmuramoyl_pentapeptide_biosynthesis_I_(meso_diaminopimelate_con
PWY_6387__UDP_N_acetylmuramoyl_pentapeptide_biosynthesis_I_(meso_diaminopimelate_con
PWY_6387__UDP_N_acetylmuramoyl_pentapeptide_biosynthesis_I_(meso_diaminopimelate_con
PWY_6387__UDP_N_acetylmuramoyl_pentapeptide_biosynthesis_I_(meso_diaminopimelate_con
PWY_6387__UDP_N_acetylmuramoyl_pentapeptide_biosynthesis_I_(meso_diaminopimelate_con
PWY_6387__UDP_N_acetylmuramoyl_pentapeptide_biosynthesis_I_(meso_diaminopimelate_con
PWY_6387__UDP_N_acetylmuramoyl_pentapeptide_biosynthesis_I_(meso_diaminopimelate_con
PWY_6387__UDP_N_acetylmuramoyl_pentapeptide_biosynthesis_I_(meso_diaminopimelate_con
PWY_6387__UDP_N_acetylmuramoyl_pentapeptide_biosynthesis_I_(meso_diaminopimelate_con
PWY_6387__UDP_N_acetylmuramoyl_pentapeptide_biosynthesis_I_(meso_diaminopimelate_con
PWY_6387__UDP_N_acetylmuramoyl_pentapeptide_biosynthesis_I_(meso_diaminopimelate_con
PWY_6387__UDP_N_acetylmuramoyl_pentapeptide_biosynthesis_I_(meso_diaminopimelate_con
PWY_6387__UDP_N_acetylmuramoyl_pentapeptide_biosynthesis_I_(meso_diaminopimelate_con
PWY_6387__UDP_N_acetylmuramoyl_pentapeptide_biosynthesis_I_(meso_diaminopimelate_con
PWY_6387__UDP_N_acetylmuramoyl_pentapeptide_biosynthesis_I_(meso_diaminopimelate_con
PWY_6387__UDP_N_acetylmuramoyl_pentapeptide_biosynthesis_I_(meso_diaminopimelate_con
PWY_6387__UDP_N_acetylmuramoyl_pentapeptide_biosynthesis_I_(meso_diaminopimelate_con
PWY_6387__UDP_N_acetylmuramoyl_pentapeptide_biosynthesis_I_(meso_diaminopimelate_con
PWY_6387__UDP_N_acetylmuramoyl_pentapeptide_biosynthesis_I_(meso_diaminopimelate_con

PWY_6387__UDP_N_acetylmuramoyl_pentapeptide_biosynthesis_I_(meso_diaminopimelate_cont
PWY_6387__UDP_N_acetylmuramoyl_pentapeptide_biosynthesis_I_(meso_diaminopimelate_cont
PWY_6387__UDP_N_acetylmuramoyl_pentapeptide_biosynthesis_I_(meso_diaminopimelate_cont
PWY_6387__UDP_N_acetylmuramoyl_pentapeptide_biosynthesis_I_(meso_diaminopimelate_cont
PWY_6387__UDP_N_acetylmuramoyl_pentapeptide_biosynthesis_I_(meso_diaminopimelate_cont
PWY_6387__UDP_N_acetylmuramoyl_pentapeptide_biosynthesis_I_(meso_diaminopimelate_cont
PWY_6387__UDP_N_acetylmuramoyl_pentapeptide_biosynthesis_I_(meso_diaminopimelate_cont
PWY_6387__UDP_N_acetylmuramoyl_pentapeptide_biosynthesis_I_(meso_diaminopimelate_cont
PWY_6387__UDP_N_acetylmuramoyl_pentapeptide_biosynthesis_I_(meso_diaminopimelate_cont
PWY_6387__UDP_N_acetylmuramoyl_pentapeptide_biosynthesis_I_(meso_diaminopimelate_cont
PWY_6396__superpathway_of_2,3_butanediol_biosynthesis_unclassified
PWY_6435__4_hydroxybenzoate_biosynthesis_V
PWY_6435__4_hydroxybenzoate_biosynthesis_V_unclassified
PWY_6471__peptidoglycan_biosynthesis_IV_(Enterococcus_faecium)_g__Streptococcus_s__Strept
PWY_6507__4_deoxy_L_threo_hex_4_enopyranuronate_degradation_g__Citrobacter_s__Citrobac
PWY_6507__4_deoxy_L_threo_hex_4_enopyranuronate_degradation_g__Clostridiales_noname_s
PWY_6507__4_deoxy_L_threo_hex_4_enopyranuronate_degradation_g__Enterobacter_s__Entero
PWY_6507__4_deoxy_L_threo_hex_4_enopyranuronate_degradation_g__Lactobacillus_s__Lactob
PWY_6519__8_amino_7_oxononanoate_biosynthesis_I_g__Bacteroides_s__Bacteroides_sp_2_2_
PWY_6519__8_amino_7_oxononanoate_biosynthesis_I_g__Bacteroides_s__Bacteroides_xylanisol
PWY_6519__8_amino_7_oxononanoate_biosynthesis_I_g__Citrobacter_s__Citrobacter_freundii
PWY_6519__8_amino_7_oxononanoate_biosynthesis_I_g__Enterobacter_s__Enterobacter_cloaca
PWY_6519__8_amino_7_oxononanoate_biosynthesis_I_g__Haemophilus_s__Haemophilus_parain
PWY_6519__8_amino_7_oxononanoate_biosynthesis_I_g__Klebsiella_s__Klebsiella_pneumoniae
PWY_6527__stachyose_degradation_g__Blautia_s__Blautia_sp_KLE_1732
PWY_6527__stachyose_degradation_g__Citrobacter_s__Citrobacter_freundii
PWY_6527__stachyose_degradation_g__Coprococcus_s__Coprococcus_eutactus
PWY_6527__stachyose_degradation_g__Coprococcus_s__Coprococcus_sp_ART55_1
PWY_6527__stachyose_degradation_g__Eubacterium_s__Eubacterium_siraeum
PWY_6527__stachyose_degradation_g__Lachnospiraceae_noname_s__Lachnospiraceae_bacteriur
PWY_6527__stachyose_degradation_g__Lactobacillus_s__Lactobacillus_casei_paracasei
PWY_6527__stachyose_degradation_g__Lactobacillus_s__Lactobacillus_rhamnosus
PWY_6527__stachyose_degradation_g__Roseburia_s__Roseburia_intestinalis
PWY_6527__stachyose_degradation_g__Ruminococcus_s__Ruminococcus_sp
PWY_6527__stachyose_degradation_g__Streptococcus_s__Streptococcus_parasanguinis
PWY_6531__mannitol_cycle_g__Escherichia_s__Escherichia_coli
PWY_6531__mannitol_cycle_unclassified
PWY_6545__pyrimidine_deoxyribonucleotides_de_novo_biosynthesis_III_g__Eggerthella_s__Egge
PWY_6545__pyrimidine_deoxyribonucleotides_de_novo_biosynthesis_III_g__Eggerthella_s__Egge
PWY_6545__pyrimidine_deoxyribonucleotides_de_novo_biosynthesis_III_g__Eggerthella_s__Egge
PWY_6545__pyrimidine_deoxyribonucleotides_de_novo_biosynthesis_III_g__Haemophilus_s__Ha
PWY_6545__pyrimidine_deoxyribonucleotides_de_novo_biosynthesis_III_g__Slackia_s__Slackia_p
PWY_6588__pyruvate_fermentation_to_acetone_g__Anaerostipes_s__Anaerostipes_hadrus
PWY_6588__pyruvate_fermentation_to_acetone_g__Clostridium_s__Clostridium_sp_SS2_1
PWY_6588__pyruvate_fermentation_to_acetone_g__Lachnospiraceae_noname_s__Lachnospirace
PWY_6590__superpathway_of_Clostridium_acetobutylicum_acidogenic_fermentation_unclassifiec
PWY_6595__superpathway_of_guanosine_nucleotides_degradation_(plants)_g__Blautia_s__Rumi

PWY_6606__guanosine_nucleotides_degradation_II_g__Blautia_s__Ruminococcus_obeum
PWY_6608__guanosine_nucleotides_degradation_III_g__Blautia_s__Ruminococcus_obeum
PWY_6608__guanosine_nucleotides_degradation_III_g__Coprococcus_s__Coprococcus_catus
PWY_6608__guanosine_nucleotides_degradation_III_g__Klebsiella_s__Klebsiella_pneumoniae
PWY_6608__guanosine_nucleotides_degradation_III_g__Odoribacter_s__Odoribacter_splanchnicu
PWY_6608__guanosine_nucleotides_degradation_III_g__Parabacteroides_s__Parabacteroides_joh
PWY_6609__adenine_and_adenosine_salvage_III_g__Alistipes_s__Alistipes_senegalensis
PWY_6609__adenine_and_adenosine_salvage_III_g__Alistipes_s__Alistipes_shahii
PWY_6609__adenine_and_adenosine_salvage_III_g__Anaerofustis_s__Anaerofustis_stercorihomir
PWY_6609__adenine_and_adenosine_salvage_III_g__Anaerotruncus_s__Anaerotruncus_colihomir
PWY_6609__adenine_and_adenosine_salvage_III_g__Bacteroides_s__Bacteroides_faecis
PWY_6609__adenine_and_adenosine_salvage_III_g__Bacteroides_s__Bacteroides_finegoldii
PWY_6609__adenine_and_adenosine_salvage_III_g__Bacteroides_s__Bacteroides_ovatus
PWY_6609__adenine_and_adenosine_salvage_III_g__Bacteroides_s__Bacteroides_sp_1_1_30
PWY_6609__adenine_and_adenosine_salvage_III_g__Bacteroides_s__Bacteroides_sp_1_1_6
PWY_6609__adenine_and_adenosine_salvage_III_g__Bacteroides_s__Bacteroides_sp_2_1_22
PWY_6609__adenine_and_adenosine_salvage_III_g__Bacteroides_s__Bacteroides_sp_2_2_4
PWY_6609__adenine_and_adenosine_salvage_III_g__Bacteroides_s__Bacteroides_sp_3_1_23
PWY_6609__adenine_and_adenosine_salvage_III_g__Bacteroides_s__Bacteroides_thetaiotaomicr
PWY_6609__adenine_and_adenosine_salvage_III_g__Bacteroides_s__Bacteroides_xylanisolvens
PWY_6609__adenine_and_adenosine_salvage_III_g__Blautia_s__Blautia_sp_KLE_1732
PWY_6609__adenine_and_adenosine_salvage_III_g__Blautia_s__Ruminococcus_gnavus
PWY_6609__adenine_and_adenosine_salvage_III_g__Citrobacter_s__Citrobacter_freundii
PWY_6609__adenine_and_adenosine_salvage_III_g__Clostridiales_noname_s__Clostridiales_bact
PWY_6609__adenine_and_adenosine_salvage_III_g__Clostridium_s__Clostridium_asparagiforme
PWY_6609__adenine_and_adenosine_salvage_III_g__Clostridium_s__Clostridium_perfringens
PWY_6609__adenine_and_adenosine_salvage_III_g__Collinsella_s__Collinsella_intestinalis
PWY_6609__adenine_and_adenosine_salvage_III_g__Coprococcus_s__Coprococcus_catus
PWY_6609__adenine_and_adenosine_salvage_III_g__Eggerthella_s__Eggerthella_lenta
PWY_6609__adenine_and_adenosine_salvage_III_g__Eggerthella_s__Eggerthella_sp_1_3_56FAA
PWY_6609__adenine_and_adenosine_salvage_III_g__Eggerthella_s__Eggerthella_sp_HGA1
PWY_6609__adenine_and_adenosine_salvage_III_g__Enterobacter_s__Enterobacter_cloacae
PWY_6609__adenine_and_adenosine_salvage_III_g__Erysipelotrichaceae_noname_s__Clostridium
PWY_6609__adenine_and_adenosine_salvage_III_g__Erysipelotrichaceae_noname_s__Eubacteriu
PWY_6609__adenine_and_adenosine_salvage_III_g__Erysipelotrichaceae_noname_s__Eubacteriu
PWY_6609__adenine_and_adenosine_salvage_III_g__Holdemania_s__Holdemania_filiformis
PWY_6609__adenine_and_adenosine_salvage_III_g__Klebsiella_s__Klebsiella_pneumoniae
PWY_6609__adenine_and_adenosine_salvage_III_g__Lachnospiraceae_noname_s__Lachnospirace
PWY_6609__adenine_and_adenosine_salvage_III_g__Lachnospiraceae_noname_s__Lachnospirace
PWY_6609__adenine_and_adenosine_salvage_III_g__Lactobacillus_s__Lactobacillus_casei_paraca
PWY_6609__adenine_and_adenosine_salvage_III_g__Odoribacter_s__Odoribacter_splanchnicus
PWY_6609__adenine_and_adenosine_salvage_III_g__Pediococcus_s__Pediococcus_acidilactici
PWY_6609__adenine_and_adenosine_salvage_III_g__Ruminococcus_s__Ruminococcus_champane
PWY_6609__adenine_and_adenosine_salvage_III_g__Streptococcus_s__Streptococcus_australis
PWY_6609__adenine_and_adenosine_salvage_III_g__Streptococcus_s__Streptococcus_mitis_orali
PWY_6609__adenine_and_adenosine_salvage_III_g__Streptococcus_s__Streptococcus_sanguinis
PWY_6612__superpathway_of_tetrahydrofolate_biosynthesis_g__Lactococcus_s__Lactococcus_lac

PWY_6628__superpathway_of_L_phenylalanine_biosynthesis_g__Citrobacter_s__Citrobacter_freu
PWY_6628__superpathway_of_L_phenylalanine_biosynthesis_g__Klebsiella_s__Klebsiella_pneum
PWY_6628__superpathway_of_L_phenylalanine_biosynthesis_g__Propionibacterium_s__Propionil
PWY_6629__superpathway_of_L_tryptophan_biosynthesis_g__Citrobacter_s__Citrobacter_freund
PWY_6629__superpathway_of_L_tryptophan_biosynthesis_unclassified
PWY_6630__superpathway_of_L_tyrosine_biosynthesis_g__Citrobacter_s__Citrobacter_freundii
PWY_6630__superpathway_of_L_tyrosine_biosynthesis_g__Klebsiella_s__Klebsiella_pneumoniae
PWY_6630__superpathway_of_L_tyrosine_biosynthesis_g__Propionibacterium_s__Propionibacter
PWY_6690__cinnamate_and_3_hydroxycinnamate_degradation_to_2_oxopent_4_enoate_g__Citr
PWY_6690__cinnamate_and_3_hydroxycinnamate_degradation_to_2_oxopent_4_enoate_g__Kleb
PWY_6700__queuosine_biosynthesis_g__Adlercreutzia_s__Adlercreutzia_equolifaciens
PWY_6700__queuosine_biosynthesis_g__Akkermansia_s__Akkermansia_muciniphila
PWY_6700__queuosine_biosynthesis_g__Anaerostipes_s__Anaerostipes_hadrus
PWY_6700__queuosine_biosynthesis_g__Bacteroides_s__Bacteroides_cellulosilyticus
PWY_6700__queuosine_biosynthesis_g__Bacteroides_s__Bacteroides_coprocola
PWY_6700__queuosine_biosynthesis_g__Bacteroides_s__Bacteroides_dorei
PWY_6700__queuosine_biosynthesis_g__Bacteroides_s__Bacteroides_eggerthii
PWY_6700__queuosine_biosynthesis_g__Bacteroides_s__Bacteroides_faecis
PWY_6700__queuosine_biosynthesis_g__Bacteroides_s__Bacteroides_finegoldii
PWY_6700__queuosine_biosynthesis_g__Bacteroides_s__Bacteroides_fragilis
PWY_6700__queuosine_biosynthesis_g__Bacteroides_s__Bacteroides_massiliensis
PWY_6700__queuosine_biosynthesis_g__Bacteroides_s__Bacteroides_ovatus
PWY_6700__queuosine_biosynthesis_g__Bacteroides_s__Bacteroides_plebeius
PWY_6700__queuosine_biosynthesis_g__Bacteroides_s__Bacteroides_sp_1_1_30
PWY_6700__queuosine_biosynthesis_g__Bacteroides_s__Bacteroides_sp_1_1_6
PWY_6700__queuosine_biosynthesis_g__Bacteroides_s__Bacteroides_sp_2_1_22
PWY_6700__queuosine_biosynthesis_g__Bacteroides_s__Bacteroides_sp_2_2_4
PWY_6700__queuosine_biosynthesis_g__Bacteroides_s__Bacteroides_sp_3_1_19
PWY_6700__queuosine_biosynthesis_g__Bacteroides_s__Bacteroides_sp_3_1_23
PWY_6700__queuosine_biosynthesis_g__Bacteroides_s__Bacteroides_sp_3_1_33FAA
PWY_6700__queuosine_biosynthesis_g__Bacteroides_s__Bacteroides_sp_3_1_40A
PWY_6700__queuosine_biosynthesis_g__Bacteroides_s__Bacteroides_sp_4_3_47FAA
PWY_6700__queuosine_biosynthesis_g__Bacteroides_s__Bacteroides_sp_9_1_42FAA
PWY_6700__queuosine_biosynthesis_g__Bacteroides_s__Bacteroides_sp_D20
PWY_6700__queuosine_biosynthesis_g__Bacteroides_s__Bacteroides_stercoris
PWY_6700__queuosine_biosynthesis_g__Bacteroides_s__Bacteroides_thetaiotaomicron
PWY_6700__queuosine_biosynthesis_g__Bacteroides_s__Bacteroides_uniformis
PWY_6700__queuosine_biosynthesis_g__Bacteroides_s__Bacteroides_xylanisolvens
PWY_6700__queuosine_biosynthesis_g__Barnesiella_s__Barnesiella_intestinihominis
PWY_6700__queuosine_biosynthesis_g__Bilophila_s__Bilophila_wadsworthia
PWY_6700__queuosine_biosynthesis_g__Blautia_s__Blautia_hydrogenotrophica
PWY_6700__queuosine_biosynthesis_g__Blautia_s__Blautia_sp_KLE_1732
PWY_6700__queuosine_biosynthesis_g__Blautia_s__Ruminococcus_torques
PWY_6700__queuosine_biosynthesis_g__Burkholderiales_noname_s__Burkholderiales_bacterium
PWY_6700__queuosine_biosynthesis_g__Butyrivibrio_s__Butyrivibrio_crossotus
PWY_6700__queuosine_biosynthesis_g__Citrobacter_s__Citrobacter_freundii
PWY_6700__queuosine_biosynthesis_g__Clostridium_s__Clostridium_sp_L2_50

PWY_6700__queuosine_biosynthesis_g__Clostridium_s__Clostridium_sp_SS2_1
PWY_6700__queuosine_biosynthesis_g__Coprobacter_s__Coprobacter_fastidiosus
PWY_6700__queuosine_biosynthesis_g__Coprococcus_s__Coprococcus_catus
PWY_6700__queuosine_biosynthesis_g__Coprococcus_s__Coprococcus_eutactus
PWY_6700__queuosine_biosynthesis_g__Coprococcus_s__Coprococcus_sp_ART55_1
PWY_6700__queuosine_biosynthesis_g__Dialister_s__Dialister_invisus
PWY_6700__queuosine_biosynthesis_g__Enterobacter_s__Enterobacter_cloacae
PWY_6700__queuosine_biosynthesis_g__Enterococcus_s__Enterococcus_faecalis
PWY_6700__queuosine_biosynthesis_g__Enterococcus_s__Enterococcus_faecium
PWY_6700__queuosine_biosynthesis_g__Enterorhabdus_s__Enterorhabdus_caecimuris
PWY_6700__queuosine_biosynthesis_g__Eubacterium_s__Eubacterium_hallii
PWY_6700__queuosine_biosynthesis_g__Klebsiella_s__Klebsiella_pneumoniae
PWY_6700__queuosine_biosynthesis_g__Lachnospiraceae_noname_s__Lachnospiraceae_bacteriu
PWY_6700__queuosine_biosynthesis_g__Megasphaera_s__Megasphaera_micronuciformis
PWY_6700__queuosine_biosynthesis_g__Odoribacter_s__Odoribacter_laneus
PWY_6700__queuosine_biosynthesis_g__Odoribacter_s__Odoribacter_splanchnicus
PWY_6700__queuosine_biosynthesis_g__Oxalobacter_s__Oxalobacter_formigenes
PWY_6700__queuosine_biosynthesis_g__Parabacteroides_s__Parabacteroides_distasonis
PWY_6700__queuosine_biosynthesis_g__Parabacteroides_s__Parabacteroides_johnsonii
PWY_6700__queuosine_biosynthesis_g__Parabacteroides_s__Parabacteroides_sp_20_3
PWY_6700__queuosine_biosynthesis_g__Parabacteroides_s__Parabacteroides_sp_D13
PWY_6700__queuosine_biosynthesis_g__Paraprevotella_s__Paraprevotella_clara
PWY_6700__queuosine_biosynthesis_g__Parasutterella_s__Parasutterella_excrementihominis
PWY_6700__queuosine_biosynthesis_g__Peptostreptococcaceae_noname_s__Clostridium_bartlet
PWY_6700__queuosine_biosynthesis_g__Phascolarctobacterium_s__Phascolarctobacterium_succi
PWY_6700__queuosine_biosynthesis_g__Prevotella_s__Prevotella_copri
PWY_6700__queuosine_biosynthesis_g__Ruminococcus_s__Ruminococcus_callidus
PWY_6700__queuosine_biosynthesis_g__Ruminococcus_s__Ruminococcus_sp
PWY_6700__queuosine_biosynthesis_g__Streptococcus_s__Streptococcus_mitis_oralis_pneumoni
PWY_6700__queuosine_biosynthesis_g__Veillonella_s__Veillonella_dispar
PWY_6700__queuosine_biosynthesis_g__Veillonella_s__Veillonella_parvula
PWY_6703__preQ0_biosynthesis_g__Adlercreutzia_s__Adlercreutzia_equolifaciens
PWY_6703__preQ0_biosynthesis_g__Akkermansia_s__Akkermansia_muciniphila
PWY_6703__preQ0_biosynthesis_g__Alistipes_s__Alistipes_putredinis
PWY_6703__preQ0_biosynthesis_g__Anaerostipes_s__Anaerostipes_hadrus
PWY_6703__preQ0_biosynthesis_g__Bacteroides_s__Bacteroides_cellulosilyticus
PWY_6703__preQ0_biosynthesis_g__Bacteroides_s__Bacteroides_clarus
PWY_6703__preQ0_biosynthesis_g__Bacteroides_s__Bacteroides_dorei
PWY_6703__preQ0_biosynthesis_g__Bacteroides_s__Bacteroides_eggerthii
PWY_6703__preQ0_biosynthesis_g__Bacteroides_s__Bacteroides_faecis
PWY_6703__preQ0_biosynthesis_g__Bacteroides_s__Bacteroides_fragilis
PWY_6703__preQ0_biosynthesis_g__Bacteroides_s__Bacteroides_massiliensis
PWY_6703__preQ0_biosynthesis_g__Bacteroides_s__Bacteroides_ovatus
PWY_6703__preQ0_biosynthesis_g__Bacteroides_s__Bacteroides_plebeius
PWY_6703__preQ0_biosynthesis_g__Bacteroides_s__Bacteroides_sp_1_1_30
PWY_6703__preQ0_biosynthesis_g__Bacteroides_s__Bacteroides_sp_1_1_6
PWY_6703__preQ0_biosynthesis_g__Bacteroides_s__Bacteroides_sp_2_1_22

PWY_6703__preQ0_biosynthesis_g__Bacteroides_s__Bacteroides_sp_2_2_4
PWY_6703__preQ0_biosynthesis_g__Bacteroides_s__Bacteroides_sp_3_1_19
PWY_6703__preQ0_biosynthesis_g__Bacteroides_s__Bacteroides_sp_3_1_23
PWY_6703__preQ0_biosynthesis_g__Bacteroides_s__Bacteroides_sp_3_1_33FAA
PWY_6703__preQ0_biosynthesis_g__Bacteroides_s__Bacteroides_sp_3_1_40A
PWY_6703__preQ0_biosynthesis_g__Bacteroides_s__Bacteroides_sp_4_3_47FAA
PWY_6703__preQ0_biosynthesis_g__Bacteroides_s__Bacteroides_sp_9_1_42FAA
PWY_6703__preQ0_biosynthesis_g__Bacteroides_s__Bacteroides_sp_D20
PWY_6703__preQ0_biosynthesis_g__Bacteroides_s__Bacteroides_stercoris
PWY_6703__preQ0_biosynthesis_g__Bacteroides_s__Bacteroides_thetaiotaomicron
PWY_6703__preQ0_biosynthesis_g__Bacteroides_s__Bacteroides_uniformis
PWY_6703__preQ0_biosynthesis_g__Bacteroides_s__Bacteroides_xylanisolvens
PWY_6703__preQ0_biosynthesis_g__Barnesiella_s__Barnesiella_intestinihominis
PWY_6703__preQ0_biosynthesis_g__Bilophila_s__Bilophila_wadsworthia
PWY_6703__preQ0_biosynthesis_g__Blautia_s__Blautia_sp_KLE_1732
PWY_6703__preQ0_biosynthesis_g__Butyrivibrio_s__Butyrivibrio_crossotus
PWY_6703__preQ0_biosynthesis_g__Citrobacter_s__Citrobacter_freundii
PWY_6703__preQ0_biosynthesis_g__Clostridium_s__Clostridium_sp_L2_50
PWY_6703__preQ0_biosynthesis_g__Clostridium_s__Clostridium_sp_SS2_1
PWY_6703__preQ0_biosynthesis_g__Coprobacter_s__Coprobacter_fastidiosus
PWY_6703__preQ0_biosynthesis_g__Coprococcus_s__Coprococcus_catus
PWY_6703__preQ0_biosynthesis_g__Coprococcus_s__Coprococcus_eutactus
PWY_6703__preQ0_biosynthesis_g__Dialister_s__Dialister_invisus
PWY_6703__preQ0_biosynthesis_g__Enterobacter_s__Enterobacter_cloacae
PWY_6703__preQ0_biosynthesis_g__Eubacterium_s__Eubacterium_hallii
PWY_6703__preQ0_biosynthesis_g__Eubacterium_s__Eubacterium_ramulus
PWY_6703__preQ0_biosynthesis_g__Klebsiella_s__Klebsiella_pneumoniae
PWY_6703__preQ0_biosynthesis_g__Lachnospiraceae_noname_s__Lachnospiraceae_bacterium_5
PWY_6703__preQ0_biosynthesis_g__Odoribacter_s__Odoribacter_laneus
PWY_6703__preQ0_biosynthesis_g__Odoribacter_s__Odoribacter_splanchnicus
PWY_6703__preQ0_biosynthesis_g__Parabacteroides_s__Parabacteroides_distasonis
PWY_6703__preQ0_biosynthesis_g__Parabacteroides_s__Parabacteroides_merdae
PWY_6703__preQ0_biosynthesis_g__Parabacteroides_s__Parabacteroides_sp_20_3
PWY_6703__preQ0_biosynthesis_g__Parabacteroides_s__Parabacteroides_sp_D13
PWY_6703__preQ0_biosynthesis_g__Peptostreptococcaceae_noname_s__Clostridium_bartlettii
PWY_6703__preQ0_biosynthesis_g__Ruminococcus_s__Ruminococcus_callidus
PWY_6703__preQ0_biosynthesis_g__Ruminococcus_s__Ruminococcus_lactaris
PWY_6703__preQ0_biosynthesis_g__Streptococcus_s__Streptococcus_mitis_oralis_pneumoniae
PWY_6703__preQ0_biosynthesis_g__Veillonella_s__Veillonella_dispar
PWY_6703__preQ0_biosynthesis_g__Veillonella_s__Veillonella_parvula
PWY_6708__ubiquinol_8_biosynthesis_(prokaryotic)_g__Citrobacter_s__Citrobacter_freundii
PWY_6708__ubiquinol_8_biosynthesis_(prokaryotic)_g__Enterobacter_s__Enterobacter_cloacae
PWY_6708__ubiquinol_8_biosynthesis_(prokaryotic)_g__Klebsiella_s__Klebsiella_pneumoniae
PWY_6708__ubiquinol_8_biosynthesis_(prokaryotic)_unclassified
PWY_6737__starch_degradation_V_g__Akkermansia_s__Akkermansia_muciniphila
PWY_6737__starch_degradation_V_g__Alistipes_s__Alistipes_finegoldii
PWY_6737__starch_degradation_V_g__Alistipes_s__Alistipes_sp_HGB5

PWY_6737__starch_degradation_V_g__Anaerostipes_s__Anaerostipes_hadrus
PWY_6737__starch_degradation_V_g__Anaerotruncus_s__Anaerotruncus_colihominis
PWY_6737__starch_degradation_V_g__Bifidobacterium_s__Bifidobacterium_dentium
PWY_6737__starch_degradation_V_g__Blautia_s__Blautia_sp_KLE_1732
PWY_6737__starch_degradation_V_g__Blautia_s__Ruminococcus_gnavus
PWY_6737__starch_degradation_V_g__Blautia_s__Ruminococcus_obeum
PWY_6737__starch_degradation_V_g__Butyrivibrio_s__Butyrivibrio_crossotus
PWY_6737__starch_degradation_V_g__Catenibacterium_s__Catenibacterium_mitsuokai
PWY_6737__starch_degradation_V_g__Clostridiales_noname_s__Clostridiales_bacterium_1_7_47
PWY_6737__starch_degradation_V_g__Clostridium_s__Clostridium_asparagiforme
PWY_6737__starch_degradation_V_g__Clostridium_s__Clostridium_bolteae
PWY_6737__starch_degradation_V_g__Clostridium_s__Clostridium_citroniae
PWY_6737__starch_degradation_V_g__Clostridium_s__Clostridium_clostridioforme
PWY_6737__starch_degradation_V_g__Clostridium_s__Clostridium_hathewayi
PWY_6737__starch_degradation_V_g__Clostridium_s__Clostridium_leptum
PWY_6737__starch_degradation_V_g__Clostridium_s__Clostridium_perfringens
PWY_6737__starch_degradation_V_g__Clostridium_s__Clostridium_sp_SS2_1
PWY_6737__starch_degradation_V_g__Clostridium_s__Clostridium_symbiosum
PWY_6737__starch_degradation_V_g__Coprococcus_s__Coprococcus_catus
PWY_6737__starch_degradation_V_g__Dorea_s__Dorea_formicigenerans
PWY_6737__starch_degradation_V_g__Erysipelotrichaceae_noname_s__Eubacterium_biforme
PWY_6737__starch_degradation_V_g__Eubacterium_s__Eubacterium_hallii
PWY_6737__starch_degradation_V_g__Eubacterium_s__Eubacterium_ramulus
PWY_6737__starch_degradation_V_g__Eubacterium_s__Eubacterium_siraeum
PWY_6737__starch_degradation_V_g__Eubacterium_s__Eubacterium_ventriosum
PWY_6737__starch_degradation_V_g__Fusobacterium_s__Fusobacterium_nucleatum
PWY_6737__starch_degradation_V_g__Lachnospiraceae_noname_s__Lachnospiraceae_bacterium
PWY_6737__starch_degradation_V_g__Lachnospiraceae_noname_s__Lachnospiraceae_bacterium
PWY_6737__starch_degradation_V_g__Lachnospiraceae_noname_s__Lachnospiraceae_bacterium
PWY_6737__starch_degradation_V_g__Lachnospiraceae_noname_s__Lachnospiraceae_bacterium
PWY_6737__starch_degradation_V_g__Lachnospiraceae_noname_s__Lachnospiraceae_bacterium
PWY_6737__starch_degradation_V_g__Lachnospiraceae_noname_s__Lachnospiraceae_bacterium
PWY_6737__starch_degradation_V_g__Lachnospiraceae_noname_s__Lachnospiraceae_bacterium
PWY_6737__starch_degradation_V_g__Roseburia_s__Roseburia_hominis
PWY_6737__starch_degradation_V_g__Roseburia_s__Roseburia_inulinivorans
PWY_6737__starch_degradation_V_g__Ruminococcaceae_noname_s__Ruminococcaceae_bacteri
PWY_6737__starch_degradation_V_g__Ruminococcus_s__Ruminococcus_sp
PWY_6737__starch_degradation_V_g__Streptococcus_s__Streptococcus_australis
PWY_6737__starch_degradation_V_g__Streptococcus_s__Streptococcus_sanguinis
PWY_6737__starch_degradation_V_g__Subdoligranulum_s__Subdoligranulum_sp_4_3_54A2FAA
PWY_6785__hydrogen_production_VIII
PWY_6785__hydrogen_production_VIII_unclassified
PWY_6803__phosphatidylcholine_acyl_editing_g__Citrobacter_s__Citrobacter_freundii
PWY_6803__phosphatidylcholine_acyl_editing_g__Enterobacter_s__Enterobacter_cloacae
PWY_6803__phosphatidylcholine_acyl_editing_g__Klebsiella_s__Klebsiella_pneumoniae
PWY_6823__molybdenum_cofactor_biosynthesis_g__Citrobacter_s__Citrobacter_freundii
PWY_6823__molybdenum_cofactor_biosynthesis_unclassified

PWY_6837__fatty_acid_beta_oxidation_V_(unsaturated,_odd_number,_di_isomerase_dependent)
PWY_6837__fatty_acid_beta_oxidation_V_(unsaturated,_odd_number,_di_isomerase_dependent)
PWY_6859__all_trans_farnesol_biosynthesis_g__Streptococcus_s__Streptococcus_sanguinis
PWY_6891__thiazole_biosynthesis_II_(Bacillus)_g__Klebsiella_s__Klebsiella_pneumoniae
PWY_6891__thiazole_biosynthesis_II_(Bacillus)_unclassified
PWY_6892__thiazole_biosynthesis_I_(E__coli)_g__Klebsiella_s__Klebsiella_pneumoniae
PWY_6895__superpathway_of_thiamin_diphosphate_biosynthesis_II_unclassified
PWY_6897__thiamin_salvage_II_g__Bifidobacterium_s__Bifidobacterium_animalis
PWY_6897__thiamin_salvage_II_g__Citrobacter_s__Citrobacter_freundii
PWY_6897__thiamin_salvage_II_g__Klebsiella_s__Klebsiella_pneumoniae
PWY_6897__thiamin_salvage_II_g__Propionibacterium_s__Propionibacterium_freudenreichii
PWY_6901__superpathway_of_glucose_and_xylose_degradation_g__Klebsiella_s__Klebsiella_pne
PWY_6936__seleno_amino_acid_biosynthesis_g__Citrobacter_s__Citrobacter_freundii
PWY_6936__seleno_amino_acid_biosynthesis_g__Clostridium_s__Clostridium_asparagiforme
PWY_6936__seleno_amino_acid_biosynthesis_g__Clostridium_s__Clostridium_sp_SS2_1
PWY_6936__seleno_amino_acid_biosynthesis_g__Collinsella_s__Collinsella_intestinalis
PWY_6936__seleno_amino_acid_biosynthesis_g__Dorea_s__Dorea_formicigenerans
PWY_6936__seleno_amino_acid_biosynthesis_g__Erysipelotrichaceae_noname_s__Clostridium_sp
PWY_6936__seleno_amino_acid_biosynthesis_g__Erysipelotrichaceae_noname_s__Eubacterium_
PWY_6936__seleno_amino_acid_biosynthesis_g__Eubacterium_s__Eubacterium_ventriosum
PWY_6936__seleno_amino_acid_biosynthesis_g__Klebsiella_s__Klebsiella_pneumoniae
PWY_6936__seleno_amino_acid_biosynthesis_g__Lachnospiraceae_noname_s__Lachnospiraceae_
PWY_6936__seleno_amino_acid_biosynthesis_g__Lactobacillus_s__Lactobacillus_casei_paracasei
PWY_6936__seleno_amino_acid_biosynthesis_g__Oxalobacter_s__Oxalobacter_formigenes
PWY_6936__seleno_amino_acid_biosynthesis_g__Propionibacterium_s__Propionibacterium_freu
PWY_6936__seleno_amino_acid_biosynthesis_g__Rothia_s__Rothia_mucilaginosa
PWY_6936__seleno_amino_acid_biosynthesis_g__Streptococcus_s__Streptococcus_mitis_oralis_p
PWY_6969__TCA_cycle_V_(2_oxoglutarate_ferredoxin_oxidoreductase)_g__Citrobacter_s__Citrob
PWY_7007__methyl_ketone_biosynthesis
PWY_7007__methyl_ketone_biosynthesis_unclassified
PWY_7013__L_1,2_propanediol_degradation_g__Citrobacter_s__Citrobacter_freundii
PWY_7013__L_1,2_propanediol_degradation_g__Fusobacterium_s__Fusobacterium_nucleatum
PWY_7013__L_1,2_propanediol_degradation_g__Klebsiella_s__Klebsiella_pneumoniae
PWY_7013__L_1,2_propanediol_degradation_unclassified
PWY_7094__fatty_acid_salvage
PWY_7094__fatty_acid_salvage_unclassified
PWY_7111__pyruvate_fermentation_to_isobutanol_(engineered)_g__Akkermansia_s__Akkermans
PWY_7111__pyruvate_fermentation_to_isobutanol_(engineered)_g__Alistipes_s__Alistipes_indist
PWY_7111__pyruvate_fermentation_to_isobutanol_(engineered)_g__Alistipes_s__Alistipes_seneg
PWY_7111__pyruvate_fermentation_to_isobutanol_(engineered)_g__Anaerofustis_s__Anaerofust
PWY_7111__pyruvate_fermentation_to_isobutanol_(engineered)_g__Bacteroides_s__Bacteroides
PWY_7111__pyruvate_fermentation_to_isobutanol_(engineered)_g__Bacteroides_s__Bacteroides
PWY_7111__pyruvate_fermentation_to_isobutanol_(engineered)_g__Bacteroides_s__Bacteroides
PWY_7111__pyruvate_fermentation_to_isobutanol_(engineered)_g__Bacteroides_s__Bacteroides
PWY_7111__pyruvate_fermentation_to_isobutanol_(engineered)_g__Bacteroides_s__Bacteroides
PWY_7111__pyruvate_fermentation_to_isobutanol_(engineered)_g__Bacteroides_s__Bacteroides
PWY_7111__pyruvate_fermentation_to_isobutanol_(engineered)_g__Bacteroides_s__Bacteroides

PWY_7111__pyruvate_fermentation_to_isobutanol_(engineered)_g__Bacteroides_s__Bacteroides
PWY_7111__pyruvate_fermentation_to_isobutanol_(engineered)_g__Bacteroides_s__Bacteroides
PWY_7111__pyruvate_fermentation_to_isobutanol_(engineered)_g__Bacteroides_s__Bacteroides
PWY_7111__pyruvate_fermentation_to_isobutanol_(engineered)_g__Bacteroides_s__Bacteroides
PWY_7111__pyruvate_fermentation_to_isobutanol_(engineered)_g__Bacteroides_s__Bacteroides
PWY_7111__pyruvate_fermentation_to_isobutanol_(engineered)_g__Bacteroides_s__Bacteroides
PWY_7111__pyruvate_fermentation_to_isobutanol_(engineered)_g__Bacteroides_s__Bacteroides
PWY_7111__pyruvate_fermentation_to_isobutanol_(engineered)_g__Barnesiella_s__Barnesiella_i
PWY_7111__pyruvate_fermentation_to_isobutanol_(engineered)_g__Bilophila_s__Bilophila_wads
PWY_7111__pyruvate_fermentation_to_isobutanol_(engineered)_g__Blautia_s__Blautia_hydroge
PWY_7111__pyruvate_fermentation_to_isobutanol_(engineered)_g__Blautia_s__Blautia_sp_KLE_
PWY_7111__pyruvate_fermentation_to_isobutanol_(engineered)_g__Blautia_s__Ruminococcus_g
PWY_7111__pyruvate_fermentation_to_isobutanol_(engineered)_g__Blautia_s__Ruminococcus_c
PWY_7111__pyruvate_fermentation_to_isobutanol_(engineered)_g__Burkholderiales_noname_s_
PWY_7111__pyruvate_fermentation_to_isobutanol_(engineered)_g__Butyrivibrio_s__Butyrivibrio
PWY_7111__pyruvate_fermentation_to_isobutanol_(engineered)_g__Catenibacterium_s__Catenib
PWY_7111__pyruvate_fermentation_to_isobutanol_(engineered)_g__Citrobacter_s__Citrobacter_
PWY_7111__pyruvate_fermentation_to_isobutanol_(engineered)_g__Clostridiales_noname_s__Ba
PWY_7111__pyruvate_fermentation_to_isobutanol_(engineered)_g__Clostridiales_noname_s__Cl
PWY_7111__pyruvate_fermentation_to_isobutanol_(engineered)_g__Clostridium_s__Clostridium_
PWY_7111__pyruvate_fermentation_to_isobutanol_(engineered)_g__Clostridium_s__Clostridium_
PWY_7111__pyruvate_fermentation_to_isobutanol_(engineered)_g__Clostridium_s__Clostridium_
PWY_7111__pyruvate_fermentation_to_isobutanol_(engineered)_g__Clostridium_s__Clostridium_
PWY_7111__pyruvate_fermentation_to_isobutanol_(engineered)_g__Clostridium_s__Clostridium_
PWY_7111__pyruvate_fermentation_to_isobutanol_(engineered)_g__Clostridium_s__Clostridium_
PWY_7111__pyruvate_fermentation_to_isobutanol_(engineered)_g__Clostridium_s__Clostridium_
PWY_7111__pyruvate_fermentation_to_isobutanol_(engineered)_g__Collinsella_s__Collinsella_ta
PWY_7111__pyruvate_fermentation_to_isobutanol_(engineered)_g__Coprobacter_s__Coprobacte
PWY_7111__pyruvate_fermentation_to_isobutanol_(engineered)_g__Coprococcus_s__Coprococcu
PWY_7111__pyruvate_fermentation_to_isobutanol_(engineered)_g__Coprococcus_s__Coprococcu
PWY_7111__pyruvate_fermentation_to_isobutanol_(engineered)_g__Coprococcus_s__Coprococcu
PWY_7111__pyruvate_fermentation_to_isobutanol_(engineered)_g__Dorea_s__Dorea_formiciger
PWY_7111__pyruvate_fermentation_to_isobutanol_(engineered)_g__Eggerthella_s__Eggerthella_
PWY_7111__pyruvate_fermentation_to_isobutanol_(engineered)_g__Eggerthella_s__Eggerthella_
PWY_7111__pyruvate_fermentation_to_isobutanol_(engineered)_g__Enterobacter_s__Enterobact
PWY_7111__pyruvate_fermentation_to_isobutanol_(engineered)_g__Enterorhabdus_s__Enterorh
PWY_7111__pyruvate_fermentation_to_isobutanol_(engineered)_g__Erysipelotrichaceae_name
PWY_7111__pyruvate_fermentation_to_isobutanol_(engineered)_g__Erysipelotrichaceae_name
PWY_7111__pyruvate_fermentation_to_isobutanol_(engineered)_g__Erysipelotrichaceae_name
PWY_7111__pyruvate_fermentation_to_isobutanol_(engineered)_g__Erysipelotrichaceae_name
PWY_7111__pyruvate_fermentation_to_isobutanol_(engineered)_g__Eubacterium_s__Eubacteriu
PWY_7111__pyruvate_fermentation_to_isobutanol_(engineered)_g__Eubacterium_s__Eubacteriu
PWY_7111__pyruvate_fermentation_to_isobutanol_(engineered)_g__Eubacterium_s__Eubacteriu
PWY_7111__pyruvate_fermentation_to_isobutanol_(engineered)_g__Eubacterium_s__Eubacteriu
PWY_7111__pyruvate_fermentation_to_isobutanol_(engineered)_g__Eubacterium_s__Eubacteriu
PWY_7111__pyruvate_fermentation_to_isobutanol_(engineered)_g__Eubacterium_s__Eubacteriu
PWY_7111__pyruvate_fermentation_to_isobutanol_(engineered)_g__Fusobacterium_s__Fusobact

PWY_7111__pyruvate_fermentation_to_isobutanol_(engineered)_g__Klebsiella_s__Klebsiella_pne
PWY_7111__pyruvate_fermentation_to_isobutanol_(engineered)_g__Lachnospiraceae_noname_s
PWY_7111__pyruvate_fermentation_to_isobutanol_(engineered)_g__Lachnospiraceae_noname_s
PWY_7111__pyruvate_fermentation_to_isobutanol_(engineered)_g__Lachnospiraceae_noname_s
PWY_7111__pyruvate_fermentation_to_isobutanol_(engineered)_g__Lachnospiraceae_noname_s
PWY_7111__pyruvate_fermentation_to_isobutanol_(engineered)_g__Lachnospiraceae_noname_s
PWY_7111__pyruvate_fermentation_to_isobutanol_(engineered)_g__Lachnospiraceae_noname_s
PWY_7111__pyruvate_fermentation_to_isobutanol_(engineered)_g__Odoribacter_s__Odoribacter
PWY_7111__pyruvate_fermentation_to_isobutanol_(engineered)_g__Odoribacter_s__Odoribacter
PWY_7111__pyruvate_fermentation_to_isobutanol_(engineered)_g__Oxalobacter_s__Oxalobacte
PWY_7111__pyruvate_fermentation_to_isobutanol_(engineered)_g__Parabacteroides_s__Parabac
PWY_7111__pyruvate_fermentation_to_isobutanol_(engineered)_g__Parabacteroides_s__Parabac
PWY_7111__pyruvate_fermentation_to_isobutanol_(engineered)_g__Parabacteroides_s__Parabac
PWY_7111__pyruvate_fermentation_to_isobutanol_(engineered)_g__Parabacteroides_s__Parabac
PWY_7111__pyruvate_fermentation_to_isobutanol_(engineered)_g__Parabacteroides_s__Parabac
PWY_7111__pyruvate_fermentation_to_isobutanol_(engineered)_g__Parabacteroides_s__Parabac
PWY_7111__pyruvate_fermentation_to_isobutanol_(engineered)_g__Paraprevotella_s__Paraprev
PWY_7111__pyruvate_fermentation_to_isobutanol_(engineered)_g__Paraprevotella_s__Paraprev
PWY_7111__pyruvate_fermentation_to_isobutanol_(engineered)_g__Parasutterella_s__Parasutte
PWY_7111__pyruvate_fermentation_to_isobutanol_(engineered)_g__Peptostreptococcaceae_non
PWY_7111__pyruvate_fermentation_to_isobutanol_(engineered)_g__Phascolarctobacterium_s__
PWY_7111__pyruvate_fermentation_to_isobutanol_(engineered)_g__Propionibacterium_s__Prop
PWY_7111__pyruvate_fermentation_to_isobutanol_(engineered)_g__Ruminococcaceae_noname_
PWY_7111__pyruvate_fermentation_to_isobutanol_(engineered)_g__Ruminococcus_s__Ruminoc
PWY_7111__pyruvate_fermentation_to_isobutanol_(engineered)_g__Ruminococcus_s__Ruminoc
PWY_7111__pyruvate_fermentation_to_isobutanol_(engineered)_g__Streptococcus_s__Streptoco
PWY_7111__pyruvate_fermentation_to_isobutanol_(engineered)_g__Streptococcus_s__Streptoco
PWY_7111__pyruvate_fermentation_to_isobutanol_(engineered)_g__Streptococcus_s__Streptoco
PWY_7111__pyruvate_fermentation_to_isobutanol_(engineered)_g__Subdoligranulum_s__Subdo
PWY_7111__pyruvate_fermentation_to_isobutanol_(engineered)_g__Veillonella_s__Veillonella_d
PWY_7111__pyruvate_fermentation_to_isobutanol_(engineered)_g__Veillonella_s__Veillonella_p
PWY_7118__chitin_degradation_to_ethanol
PWY_7165__L_ascorbate_biosynthesis_VI_(engineered_pathway)
PWY_7184__pyrimidine_deoxyribonucleotides_de_novo_biosynthesis_I_g__Burkholderiales_nona
PWY_7184__pyrimidine_deoxyribonucleotides_de_novo_biosynthesis_I_g__Citrobacter_s__Citrob
PWY_7184__pyrimidine_deoxyribonucleotides_de_novo_biosynthesis_I_g__Eggerthella_s__Egger
PWY_7184__pyrimidine_deoxyribonucleotides_de_novo_biosynthesis_I_g__Klebsiella_s__Klebsiel
PWY_7184__pyrimidine_deoxyribonucleotides_de_novo_biosynthesis_I_g__Parasutterella_s__Par
PWY_7184__pyrimidine_deoxyribonucleotides_de_novo_biosynthesis_I_g__Veillonella_s__Veillon
PWY_7184__pyrimidine_deoxyribonucleotides_de_novo_biosynthesis_I_g__Veillonella_s__Veillon
PWY_7187__pyrimidine_deoxyribonucleotides_de_novo_biosynthesis_II_g__Klebsiella_s__Klebsie
PWY_7197__pyrimidine_deoxyribonucleotide_phosphorylation_g__Actinomyces_s__Actinomyces_
PWY_7197__pyrimidine_deoxyribonucleotide_phosphorylation_g__Adlercreutzia_s__Adlercreutzia
PWY_7197__pyrimidine_deoxyribonucleotide_phosphorylation_g__Akkermansia_s__Akkermansia
PWY_7197__pyrimidine_deoxyribonucleotide_phosphorylation_g__Bilophila_s__Bilophila_wadsw
PWY_7197__pyrimidine_deoxyribonucleotide_phosphorylation_g__Burkholderiales_noname_s__
PWY_7197__pyrimidine_deoxyribonucleotide_phosphorylation_g__Citrobacter_s__Citrobacter_fre

PWY_7197__pyrimidine_deoxyribonucleotide_phosphorylation_g__Collinsella_s__Collinsella_intes
PWY_7197__pyrimidine_deoxyribonucleotide_phosphorylation_g__Collinsella_s__Collinsella_tana
PWY_7197__pyrimidine_deoxyribonucleotide_phosphorylation_g__Dialister_s__Dialister_invisus
PWY_7197__pyrimidine_deoxyribonucleotide_phosphorylation_g__Eggerthella_s__Eggerthella_len
PWY_7197__pyrimidine_deoxyribonucleotide_phosphorylation_g__Eggerthella_s__Eggerthella_sp
PWY_7197__pyrimidine_deoxyribonucleotide_phosphorylation_g__Eggerthella_s__Eggerthella_sp
PWY_7197__pyrimidine_deoxyribonucleotide_phosphorylation_g__Enterorhabdus_s__Enterorhab
PWY_7197__pyrimidine_deoxyribonucleotide_phosphorylation_g__Klebsiella_s__Klebsiella_pneur
PWY_7197__pyrimidine_deoxyribonucleotide_phosphorylation_g__Lactobacillus_s__Lactobacillus_
PWY_7197__pyrimidine_deoxyribonucleotide_phosphorylation_g__Methanobrevibacter_s__Meth
PWY_7197__pyrimidine_deoxyribonucleotide_phosphorylation_g__Parasutterella_s__Parasuttere
PWY_7197__pyrimidine_deoxyribonucleotide_phosphorylation_g__Pediococcus_s__Pediococcus_
PWY_7197__pyrimidine_deoxyribonucleotide_phosphorylation_g__Phascolarctobacterium_s__Pha
PWY_7197__pyrimidine_deoxyribonucleotide_phosphorylation_g__Propionibacterium_s__Propion
PWY_7197__pyrimidine_deoxyribonucleotide_phosphorylation_g__Slackia_s__Slackia_piriformis
PWY_7197__pyrimidine_deoxyribonucleotide_phosphorylation_g__Streptococcus_s__Streptococc
PWY_7197__pyrimidine_deoxyribonucleotide_phosphorylation_g__Veillonella_s__Veillonella_disp
PWY_7197__pyrimidine_deoxyribonucleotide_phosphorylation_g__Veillonella_s__Veillonella_parv
PWY_7199__pyrimidine_deoxyribonucleosides_salvage_g__Alistipes_s__Alistipes_finegoldii
PWY_7199__pyrimidine_deoxyribonucleosides_salvage_g__Alistipes_s__Alistipes_sp_HGB5
PWY_7199__pyrimidine_deoxyribonucleosides_salvage_g__Citrobacter_s__Citrobacter_freundii
PWY_7199__pyrimidine_deoxyribonucleosides_salvage_g__Enterobacter_s__Enterobacter_cloaca
PWY_7199__pyrimidine_deoxyribonucleosides_salvage_g__Enterococcus_s__Enterococcus_faecal
PWY_7199__pyrimidine_deoxyribonucleosides_salvage_g__Enterococcus_s__Enterococcus_faeciu
PWY_7199__pyrimidine_deoxyribonucleosides_salvage_g__Lactococcus_s__Lactococcus_lactis
PWY_7199__pyrimidine_deoxyribonucleosides_salvage_g__Streptococcus_s__Streptococcus_mitis
PWY_7199__pyrimidine_deoxyribonucleosides_salvage_g__Streptococcus_s__Streptococcus_therı
PWY_7200__superpathway_of_pyrimidine_deoxyribonucleoside_salvage
PWY_7204__pyridoxal_5__phosphate_salvage_II_(plants)_unclassified
PWY_7208__superpathway_of_pyrimidine_nucleobases_salvage_g__Actinomyces_s__Actinomyce
PWY_7208__superpathway_of_pyrimidine_nucleobases_salvage_g__Adlercreutzia_s__Adlercreutz
PWY_7208__superpathway_of_pyrimidine_nucleobases_salvage_g__Akkermansia_s__Akkermansi
PWY_7208__superpathway_of_pyrimidine_nucleobases_salvage_g__Bilophila_s__Bilophila_wads
PWY_7208__superpathway_of_pyrimidine_nucleobases_salvage_g__Blautia_s__Blautia_sp_KLE_1
PWY_7208__superpathway_of_pyrimidine_nucleobases_salvage_g__Blautia_s__Ruminococcus_ob
PWY_7208__superpathway_of_pyrimidine_nucleobases_salvage_g__Burkholderiales_noname_s__
PWY_7208__superpathway_of_pyrimidine_nucleobases_salvage_g__Citrobacter_s__Citrobacter_f
PWY_7208__superpathway_of_pyrimidine_nucleobases_salvage_g__Clostridium_s__Clostridium_
PWY_7208__superpathway_of_pyrimidine_nucleobases_salvage_g__Collinsella_s__Collinsella_int
PWY_7208__superpathway_of_pyrimidine_nucleobases_salvage_g__Collinsella_s__Collinsella_tar
PWY_7208__superpathway_of_pyrimidine_nucleobases_salvage_g__Coprococcus_s__Coprococcu
PWY_7208__superpathway_of_pyrimidine_nucleobases_salvage_g__Coprococcus_s__Coprococcu
PWY_7208__superpathway_of_pyrimidine_nucleobases_salvage_g__Dialister_s__Dialister_invisus
PWY_7208__superpathway_of_pyrimidine_nucleobases_salvage_g__Enterobacter_s__Enterobacte
PWY_7208__superpathway_of_pyrimidine_nucleobases_salvage_g__Enterorhabdus_s__Enterorha
PWY_7208__superpathway_of_pyrimidine_nucleobases_salvage_g__Eubacterium_s__Eubacterium
PWY_7208__superpathway_of_pyrimidine_nucleobases_salvage_g__Gordonibacter_s__Gordoniba

PWY_7208__superpathway_of_pyrimidine_nucleobases_salvage_g__Klebsiella_s__Klebsiella_pneu
PWY_7208__superpathway_of_pyrimidine_nucleobases_salvage_g__Lactobacillus_s__Lactobacillu
PWY_7208__superpathway_of_pyrimidine_nucleobases_salvage_g__Methanobrevibacter_s__Met
PWY_7208__superpathway_of_pyrimidine_nucleobases_salvage_g__Nakaseomyces_s__Candida_
PWY_7208__superpathway_of_pyrimidine_nucleobases_salvage_g__Parasutterella_s__Parasutter
PWY_7208__superpathway_of_pyrimidine_nucleobases_salvage_g__Pediococcus_s__Pediococcus
PWY_7208__superpathway_of_pyrimidine_nucleobases_salvage_g__Phascolarctobacterium_s__P
PWY_7208__superpathway_of_pyrimidine_nucleobases_salvage_g__Propionibacterium_s__Propio
PWY_7208__superpathway_of_pyrimidine_nucleobases_salvage_g__Ruminococcus_s__Ruminoco
PWY_7208__superpathway_of_pyrimidine_nucleobases_salvage_g__Slackia_s__Slackia_piriformis
PWY_7208__superpathway_of_pyrimidine_nucleobases_salvage_g__Streptococcus_s__Streptococ
PWY_7208__superpathway_of_pyrimidine_nucleobases_salvage_g__Veillonella_s__Veillonella_di
PWY_7208__superpathway_of_pyrimidine_nucleobases_salvage_g__Veillonella_s__Veillonella_pa
PWY_7210__pyrimidine_deoxyribonucleotides_biosynthesis_from_CTP_g__Eggerthella_s__Eggert
PWY_7219__adenosine_ribonucleotides_de_novo_biosynthesis_g__Adlercreutzia_s__Adlercreutzi
PWY_7219__adenosine_ribonucleotides_de_novo_biosynthesis_g__Akkermansia_s__Akkermansia
PWY_7219__adenosine_ribonucleotides_de_novo_biosynthesis_g__Alistipes_s__Alistipes_finegol
PWY_7219__adenosine_ribonucleotides_de_novo_biosynthesis_g__Alistipes_s__Alistipes_indistin
PWY_7219__adenosine_ribonucleotides_de_novo_biosynthesis_g__Alistipes_s__Alistipes_onderd
PWY_7219__adenosine_ribonucleotides_de_novo_biosynthesis_g__Alistipes_s__Alistipes_putredi
PWY_7219__adenosine_ribonucleotides_de_novo_biosynthesis_g__Alistipes_s__Alistipes_senegal
PWY_7219__adenosine_ribonucleotides_de_novo_biosynthesis_g__Alistipes_s__Alistipes_shahii
PWY_7219__adenosine_ribonucleotides_de_novo_biosynthesis_g__Alistipes_s__Alistipes_sp_HGB
PWY_7219__adenosine_ribonucleotides_de_novo_biosynthesis_g__Anaerofustis_s__Anaerofustis_
PWY_7219__adenosine_ribonucleotides_de_novo_biosynthesis_g__Anaerostipes_s__Anaerostipes
PWY_7219__adenosine_ribonucleotides_de_novo_biosynthesis_g__Anaerotruncus_s__Anaerotrun
PWY_7219__adenosine_ribonucleotides_de_novo_biosynthesis_g__Bacteroides_s__Bacteroides_c
PWY_7219__adenosine_ribonucleotides_de_novo_biosynthesis_g__Bacteroides_s__Bacteroides_c
PWY_7219__adenosine_ribonucleotides_de_novo_biosynthesis_g__Bacteroides_s__Bacteroides_c
PWY_7219__adenosine_ribonucleotides_de_novo_biosynthesis_g__Bacteroides_s__Bacteroides_c
PWY_7219__adenosine_ribonucleotides_de_novo_biosynthesis_g__Bacteroides_s__Bacteroides_e
PWY_7219__adenosine_ribonucleotides_de_novo_biosynthesis_g__Bacteroides_s__Bacteroides_f
PWY_7219__adenosine_ribonucleotides_de_novo_biosynthesis_g__Bacteroides_s__Bacteroides_f
PWY_7219__adenosine_ribonucleotides_de_novo_biosynthesis_g__Bacteroides_s__Bacteroides_i
PWY_7219__adenosine_ribonucleotides_de_novo_biosynthesis_g__Bacteroides_s__Bacteroides_r
PWY_7219__adenosine_ribonucleotides_de_novo_biosynthesis_g__Bacteroides_s__Bacteroides_c
PWY_7219__adenosine_ribonucleotides_de_novo_biosynthesis_g__Bacteroides_s__Bacteroides_p
PWY_7219__adenosine_ribonucleotides_de_novo_biosynthesis_g__Bacteroides_s__Bacteroides_s
PWY_7219__adenosine_ribonucleotides_de_novo_biosynthesis_g__Bacteroides_s__Bacteroides_s
PWY_7219__adenosine_ribonucleotides_de_novo_biosynthesis_g__Bacteroides_s__Bacteroides_s
PWY_7219__adenosine_ribonucleotides_de_novo_biosynthesis_g__Bacteroides_s__Bacteroides_s
PWY_7219__adenosine_ribonucleotides_de_novo_biosynthesis_g__Bacteroides_s__Bacteroides_s
PWY_7219__adenosine_ribonucleotides_de_novo_biosynthesis_g__Bacteroides_s__Bacteroides_s
PWY_7219__adenosine_ribonucleotides_de_novo_biosynthesis_g__Bacteroides_s__Bacteroides_s
PWY_7219__adenosine_ribonucleotides_de_novo_biosynthesis_g__Bacteroides_s__Bacteroides_s
PWY_7219__adenosine_ribonucleotides_de_novo_biosynthesis_g__Bacteroides_s__Bacteroides_s
PWY_7219__adenosine_ribonucleotides_de_novo_biosynthesis_g__Bacteroides_s__Bacteroides_s

PWY_7219__adenosine_ribonucleotides_de_novo_biosynthesis_g__Bacteroides_s__Bacteroides_s
PWY_7219__adenosine_ribonucleotides_de_novo_biosynthesis_g__Bacteroides_s__Bacteroides_s
PWY_7219__adenosine_ribonucleotides_de_novo_biosynthesis_g__Bacteroides_s__Bacteroides_s
PWY_7219__adenosine_ribonucleotides_de_novo_biosynthesis_g__Bacteroides_s__Bacteroides_t
PWY_7219__adenosine_ribonucleotides_de_novo_biosynthesis_g__Bacteroides_s__Bacteroides_u
PWY_7219__adenosine_ribonucleotides_de_novo_biosynthesis_g__Bacteroides_s__Bacteroides_x
PWY_7219__adenosine_ribonucleotides_de_novo_biosynthesis_g__Barnesiella_s__Barnesiella_int
PWY_7219__adenosine_ribonucleotides_de_novo_biosynthesis_g__Bifidobacterium_s__Bifidobac
PWY_7219__adenosine_ribonucleotides_de_novo_biosynthesis_g__Bifidobacterium_s__Bifidobac
PWY_7219__adenosine_ribonucleotides_de_novo_biosynthesis_g__Bifidobacterium_s__Bifidobac
PWY_7219__adenosine_ribonucleotides_de_novo_biosynthesis_g__Bifidobacterium_s__Bifidobac
PWY_7219__adenosine_ribonucleotides_de_novo_biosynthesis_g__Bifidobacterium_s__Bifidobac
PWY_7219__adenosine_ribonucleotides_de_novo_biosynthesis_g__Bifidobacterium_s__Bifidobac
PWY_7219__adenosine_ribonucleotides_de_novo_biosynthesis_g__Bilophila_s__Bilophila_wadsw
PWY_7219__adenosine_ribonucleotides_de_novo_biosynthesis_g__Blautia_s__Blautia_hydrogeno
PWY_7219__adenosine_ribonucleotides_de_novo_biosynthesis_g__Blautia_s__Blautia_sp_KLE_17
PWY_7219__adenosine_ribonucleotides_de_novo_biosynthesis_g__Blautia_s__Ruminococcus_gna
PWY_7219__adenosine_ribonucleotides_de_novo_biosynthesis_g__Blautia_s__Ruminococcus_obe
PWY_7219__adenosine_ribonucleotides_de_novo_biosynthesis_g__Burkholderiales_noname_s__
PWY_7219__adenosine_ribonucleotides_de_novo_biosynthesis_g__Butyrivibrio_s__Butyrivibrio_c
PWY_7219__adenosine_ribonucleotides_de_novo_biosynthesis_g__Catenibacterium_s__Cateniba
PWY_7219__adenosine_ribonucleotides_de_novo_biosynthesis_g__Citrobacter_s__Citrobacter_fr
PWY_7219__adenosine_ribonucleotides_de_novo_biosynthesis_g__Clostridiales_noname_s__Bac
PWY_7219__adenosine_ribonucleotides_de_novo_biosynthesis_g__Clostridiales_noname_s__Clos
PWY_7219__adenosine_ribonucleotides_de_novo_biosynthesis_g__Clostridium_s__Clostridium_a
PWY_7219__adenosine_ribonucleotides_de_novo_biosynthesis_g__Clostridium_s__Clostridium_b
PWY_7219__adenosine_ribonucleotides_de_novo_biosynthesis_g__Clostridium_s__Clostridium_c
PWY_7219__adenosine_ribonucleotides_de_novo_biosynthesis_g__Clostridium_s__Clostridium_c
PWY_7219__adenosine_ribonucleotides_de_novo_biosynthesis_g__Clostridium_s__Clostridium_h
PWY_7219__adenosine_ribonucleotides_de_novo_biosynthesis_g__Clostridium_s__Clostridium_le
PWY_7219__adenosine_ribonucleotides_de_novo_biosynthesis_g__Clostridium_s__Clostridium_p
PWY_7219__adenosine_ribonucleotides_de_novo_biosynthesis_g__Clostridium_s__Clostridium_s
PWY_7219__adenosine_ribonucleotides_de_novo_biosynthesis_g__Clostridium_s__Clostridium_s
PWY_7219__adenosine_ribonucleotides_de_novo_biosynthesis_g__Clostridium_s__Clostridium_s
PWY_7219__adenosine_ribonucleotides_de_novo_biosynthesis_g__Collinsella_s__Collinsella_inte
PWY_7219__adenosine_ribonucleotides_de_novo_biosynthesis_g__Collinsella_s__Collinsella_tana
PWY_7219__adenosine_ribonucleotides_de_novo_biosynthesis_g__Coprobacter_s__Coprobacter_
PWY_7219__adenosine_ribonucleotides_de_novo_biosynthesis_g__Coprococcus_s__Coprococcus_
PWY_7219__adenosine_ribonucleotides_de_novo_biosynthesis_g__Coprococcus_s__Coprococcus_
PWY_7219__adenosine_ribonucleotides_de_novo_biosynthesis_g__Coprococcus_s__Coprococcus_
PWY_7219__adenosine_ribonucleotides_de_novo_biosynthesis_g__Dialister_s__Dialister_invisus
PWY_7219__adenosine_ribonucleotides_de_novo_biosynthesis_g__Dorea_s__Dorea_formicigene
PWY_7219__adenosine_ribonucleotides_de_novo_biosynthesis_g__Eggerthella_s__Eggerthella_le
PWY_7219__adenosine_ribonucleotides_de_novo_biosynthesis_g__Eggerthella_s__Eggerthella_sp
PWY_7219__adenosine_ribonucleotides_de_novo_biosynthesis_g__Eggerthella_s__Eggerthella_sp
PWY_7219__adenosine_ribonucleotides_de_novo_biosynthesis_g__Enterobacter_s__Enterobacter
PWY_7219__adenosine_ribonucleotides_de_novo_biosynthesis_g__Enterococcus_s__Enterococcu

PWY_7219__adenosine_ribonucleotides_de_novo_biosynthesis_g__Enterococcus_s__Enterococcu
PWY_7219__adenosine_ribonucleotides_de_novo_biosynthesis_g__Enterococcus_s__Enterococcu
PWY_7219__adenosine_ribonucleotides_de_novo_biosynthesis_g__Enterorhabdus_s__Enterorhab
PWY_7219__adenosine_ribonucleotides_de_novo_biosynthesis_g__Erysipelotrichaceae_noname_
PWY_7219__adenosine_ribonucleotides_de_novo_biosynthesis_g__Erysipelotrichaceae_noname_
PWY_7219__adenosine_ribonucleotides_de_novo_biosynthesis_g__Erysipelotrichaceae_noname_
PWY_7219__adenosine_ribonucleotides_de_novo_biosynthesis_g__Erysipelotrichaceae_noname_
PWY_7219__adenosine_ribonucleotides_de_novo_biosynthesis_g__Erysipelotrichaceae_noname_
PWY_7219__adenosine_ribonucleotides_de_novo_biosynthesis_g__Eubacterium_s__Eubacterium
PWY_7219__adenosine_ribonucleotides_de_novo_biosynthesis_g__Eubacterium_s__Eubacterium
PWY_7219__adenosine_ribonucleotides_de_novo_biosynthesis_g__Eubacterium_s__Eubacterium
PWY_7219__adenosine_ribonucleotides_de_novo_biosynthesis_g__Eubacterium_s__Eubacterium
PWY_7219__adenosine_ribonucleotides_de_novo_biosynthesis_g__Eubacterium_s__Eubacterium
PWY_7219__adenosine_ribonucleotides_de_novo_biosynthesis_g__Eubacterium_s__Eubacterium
PWY_7219__adenosine_ribonucleotides_de_novo_biosynthesis_g__Fusobacterium_s__Fusobacter
PWY_7219__adenosine_ribonucleotides_de_novo_biosynthesis_g__Gemella_s__Gemella_sanguin
PWY_7219__adenosine_ribonucleotides_de_novo_biosynthesis_g__Gordonibacter_s__Gordoniba
PWY_7219__adenosine_ribonucleotides_de_novo_biosynthesis_g__Holdemania_s__Holdemania_
PWY_7219__adenosine_ribonucleotides_de_novo_biosynthesis_g__Klebsiella_s__Klebsiella_pneu
PWY_7219__adenosine_ribonucleotides_de_novo_biosynthesis_g__Lachnospiraceae_noname_s__
PWY_7219__adenosine_ribonucleotides_de_novo_biosynthesis_g__Lachnospiraceae_noname_s__
PWY_7219__adenosine_ribonucleotides_de_novo_biosynthesis_g__Lachnospiraceae_noname_s__
PWY_7219__adenosine_ribonucleotides_de_novo_biosynthesis_g__Lachnospiraceae_noname_s__
PWY_7219__adenosine_ribonucleotides_de_novo_biosynthesis_g__Lachnospiraceae_noname_s__
PWY_7219__adenosine_ribonucleotides_de_novo_biosynthesis_g__Lachnospiraceae_noname_s__
PWY_7219__adenosine_ribonucleotides_de_novo_biosynthesis_g__Lachnospiraceae_noname_s__
PWY_7219__adenosine_ribonucleotides_de_novo_biosynthesis_g__Lachnospiraceae_noname_s__
PWY_7219__adenosine_ribonucleotides_de_novo_biosynthesis_g__Lactobacillus_s__Lactobacillus
PWY_7219__adenosine_ribonucleotides_de_novo_biosynthesis_g__Lactobacillus_s__Lactobacillus
PWY_7219__adenosine_ribonucleotides_de_novo_biosynthesis_g__Lactobacillus_s__Lactobacillus
PWY_7219__adenosine_ribonucleotides_de_novo_biosynthesis_g__Lactobacillus_s__Lactobacillus
PWY_7219__adenosine_ribonucleotides_de_novo_biosynthesis_g__Lactobacillus_s__Lactobacillus
PWY_7219__adenosine_ribonucleotides_de_novo_biosynthesis_g__Nakaseomyces_s__Candida_g
PWY_7219__adenosine_ribonucleotides_de_novo_biosynthesis_g__Odoribacter_s__Odoribacter_
PWY_7219__adenosine_ribonucleotides_de_novo_biosynthesis_g__Odoribacter_s__Odoribacter_
PWY_7219__adenosine_ribonucleotides_de_novo_biosynthesis_g__Oxalobacter_s__Oxalobacter_
PWY_7219__adenosine_ribonucleotides_de_novo_biosynthesis_g__Parabacteroides_s__Parabacte
PWY_7219__adenosine_ribonucleotides_de_novo_biosynthesis_g__Parabacteroides_s__Parabacte
PWY_7219__adenosine_ribonucleotides_de_novo_biosynthesis_g__Parabacteroides_s__Parabacte
PWY_7219__adenosine_ribonucleotides_de_novo_biosynthesis_g__Parabacteroides_s__Parabacte
PWY_7219__adenosine_ribonucleotides_de_novo_biosynthesis_g__Parabacteroides_s__Parabacte
PWY_7219__adenosine_ribonucleotides_de_novo_biosynthesis_g__Parabacteroides_s__Parabacte
PWY_7219__adenosine_ribonucleotides_de_novo_biosynthesis_g__Paraprevotella_s__Paraprevot
PWY_7219__adenosine_ribonucleotides_de_novo_biosynthesis_g__Paraprevotella_s__Paraprevot
PWY_7219__adenosine_ribonucleotides_de_novo_biosynthesis_g__Parasutterella_s__Parasuttere
PWY_7219__adenosine_ribonucleotides_de_novo_biosynthesis_g__Pediococcus_s__Pediococcus_
PWY_7219__adenosine_ribonucleotides_de_novo_biosynthesis_g__Pediococcus_s__Pediococcus_

PWY_7219__adenosine_ribonucleotides_de_novo_biosynthesis_g__Peptostreptococcaceae_nonar
PWY_7219__adenosine_ribonucleotides_de_novo_biosynthesis_g__Phascolarctobacterium_s__Ph
PWY_7219__adenosine_ribonucleotides_de_novo_biosynthesis_g__Prevotella_s__Prevotella_copr
PWY_7219__adenosine_ribonucleotides_de_novo_biosynthesis_g__Propionibacterium_s__Propio
PWY_7219__adenosine_ribonucleotides_de_novo_biosynthesis_g__Roseburia_s__Roseburia_hom
PWY_7219__adenosine_ribonucleotides_de_novo_biosynthesis_g__Roseburia_s__Roseburia_inuli
PWY_7219__adenosine_ribonucleotides_de_novo_biosynthesis_g__Ruminococcaceae_noname_s_
PWY_7219__adenosine_ribonucleotides_de_novo_biosynthesis_g__Ruminococcus_s__Ruminococ
PWY_7219__adenosine_ribonucleotides_de_novo_biosynthesis_g__Ruminococcus_s__Ruminococ
PWY_7219__adenosine_ribonucleotides_de_novo_biosynthesis_g__Ruminococcus_s__Ruminococ
PWY_7219__adenosine_ribonucleotides_de_novo_biosynthesis_g__Ruminococcus_s__Ruminococ
PWY_7219__adenosine_ribonucleotides_de_novo_biosynthesis_g__Slackia_s__Slackia_piriformis
PWY_7219__adenosine_ribonucleotides_de_novo_biosynthesis_g__Solobacterium_s__Solobacteri
PWY_7219__adenosine_ribonucleotides_de_novo_biosynthesis_g__Streptococcus_s__Streptococc
PWY_7219__adenosine_ribonucleotides_de_novo_biosynthesis_g__Streptococcus_s__Streptococc
PWY_7219__adenosine_ribonucleotides_de_novo_biosynthesis_g__Streptococcus_s__Streptococc
PWY_7219__adenosine_ribonucleotides_de_novo_biosynthesis_g__Subdoligranulum_s__Subdolig
PWY_7219__adenosine_ribonucleotides_de_novo_biosynthesis_g__Veillonella_s__Veillonella_disp
PWY_7219__adenosine_ribonucleotides_de_novo_biosynthesis_g__Veillonella_s__Veillonella_par
PWY_7220__adenosine_deoxyribonucleotides_de_novo_biosynthesis_II_g__Actinomyces_s__Actir
PWY_7220__adenosine_deoxyribonucleotides_de_novo_biosynthesis_II_g__Bacteroides_s__Bacte
PWY_7220__adenosine_deoxyribonucleotides_de_novo_biosynthesis_II_g__Bacteroides_s__Bacte
PWY_7220__adenosine_deoxyribonucleotides_de_novo_biosynthesis_II_g__Bacteroides_s__Bacte
PWY_7220__adenosine_deoxyribonucleotides_de_novo_biosynthesis_II_g__Bacteroides_s__Bacte
PWY_7220__adenosine_deoxyribonucleotides_de_novo_biosynthesis_II_g__Bacteroides_s__Bacte
PWY_7220__adenosine_deoxyribonucleotides_de_novo_biosynthesis_II_g__Bacteroides_s__Bacte
PWY_7220__adenosine_deoxyribonucleotides_de_novo_biosynthesis_II_g__Burkholderiales_nona
PWY_7220__adenosine_deoxyribonucleotides_de_novo_biosynthesis_II_g__Citrobacter_s__Citrob
PWY_7220__adenosine_deoxyribonucleotides_de_novo_biosynthesis_II_g__Clostridiales_noname
PWY_7220__adenosine_deoxyribonucleotides_de_novo_biosynthesis_II_g__Clostridium_s__Clostr
PWY_7220__adenosine_deoxyribonucleotides_de_novo_biosynthesis_II_g__Clostridium_s__Clostr
PWY_7220__adenosine_deoxyribonucleotides_de_novo_biosynthesis_II_g__Collinsella_s__Collins
PWY_7220__adenosine_deoxyribonucleotides_de_novo_biosynthesis_II_g__Collinsella_s__Collins
PWY_7220__adenosine_deoxyribonucleotides_de_novo_biosynthesis_II_g__Eggerthella_s__Egger
PWY_7220__adenosine_deoxyribonucleotides_de_novo_biosynthesis_II_g__Eggerthella_s__Egger
PWY_7220__adenosine_deoxyribonucleotides_de_novo_biosynthesis_II_g__Eggerthella_s__Egger
PWY_7220__adenosine_deoxyribonucleotides_de_novo_biosynthesis_II_g__Faecalibacterium_s__
PWY_7220__adenosine_deoxyribonucleotides_de_novo_biosynthesis_II_g__Holdemania_s__Hold
PWY_7220__adenosine_deoxyribonucleotides_de_novo_biosynthesis_II_g__Klebsiella_s__Klebsiel
PWY_7220__adenosine_deoxyribonucleotides_de_novo_biosynthesis_II_g__Oxalobacter_s__Oxalo
PWY_7220__adenosine_deoxyribonucleotides_de_novo_biosynthesis_II_g__Parasutterella_s__Par
PWY_7220__adenosine_deoxyribonucleotides_de_novo_biosynthesis_II_g__Slackia_s__Slackia_pi
PWY_7220__adenosine_deoxyribonucleotides_de_novo_biosynthesis_II_g__Solobacterium_s__So
PWY_7220__adenosine_deoxyribonucleotides_de_novo_biosynthesis_II_g__Streptococcus_s__Str
PWY_7220__adenosine_deoxyribonucleotides_de_novo_biosynthesis_II_g__Streptococcus_s__Str
PWY_7220__adenosine_deoxyribonucleotides_de_novo_biosynthesis_II_g__Veillonella_s__Veillor
PWY_7220__adenosine_deoxyribonucleotides_de_novo_biosynthesis_II_g__Veillonella_s__Veillor

PWY_7221__guanosine_ribonucleotides_de_novo_biosynthesis_g__Actinomyces_s__Actinomyces
PWY_7221__guanosine_ribonucleotides_de_novo_biosynthesis_g__Adlercreutzia_s__Adlercreutzi
PWY_7221__guanosine_ribonucleotides_de_novo_biosynthesis_g__Akkermansia_s__Akkermansia
PWY_7221__guanosine_ribonucleotides_de_novo_biosynthesis_g__Alistipes_s__Alistipes_finegold
PWY_7221__guanosine_ribonucleotides_de_novo_biosynthesis_g__Alistipes_s__Alistipes_indistin
PWY_7221__guanosine_ribonucleotides_de_novo_biosynthesis_g__Alistipes_s__Alistipes_onderd
PWY_7221__guanosine_ribonucleotides_de_novo_biosynthesis_g__Alistipes_s__Alistipes_putredi
PWY_7221__guanosine_ribonucleotides_de_novo_biosynthesis_g__Alistipes_s__Alistipes_senegal
PWY_7221__guanosine_ribonucleotides_de_novo_biosynthesis_g__Alistipes_s__Alistipes_shahii
PWY_7221__guanosine_ribonucleotides_de_novo_biosynthesis_g__Alistipes_s__Alistipes_sp_HGB
PWY_7221__guanosine_ribonucleotides_de_novo_biosynthesis_g__Anaerofustis_s__Anaerofustis_
PWY_7221__guanosine_ribonucleotides_de_novo_biosynthesis_g__Anaerotruncus_s__Anaerotru
PWY_7221__guanosine_ribonucleotides_de_novo_biosynthesis_g__Bacteroides_s__Bacteroides_c
PWY_7221__guanosine_ribonucleotides_de_novo_biosynthesis_g__Bacteroides_s__Bacteroides_c
PWY_7221__guanosine_ribonucleotides_de_novo_biosynthesis_g__Bacteroides_s__Bacteroides_c
PWY_7221__guanosine_ribonucleotides_de_novo_biosynthesis_g__Bacteroides_s__Bacteroides_f
PWY_7221__guanosine_ribonucleotides_de_novo_biosynthesis_g__Bacteroides_s__Bacteroides_f
PWY_7221__guanosine_ribonucleotides_de_novo_biosynthesis_g__Bacteroides_s__Bacteroides_i
PWY_7221__guanosine_ribonucleotides_de_novo_biosynthesis_g__Bacteroides_s__Bacteroides_n
PWY_7221__guanosine_ribonucleotides_de_novo_biosynthesis_g__Bacteroides_s__Bacteroides_o
PWY_7221__guanosine_ribonucleotides_de_novo_biosynthesis_g__Bacteroides_s__Bacteroides_p
PWY_7221__guanosine_ribonucleotides_de_novo_biosynthesis_g__Bacteroides_s__Bacteroides_s
PWY_7221__guanosine_ribonucleotides_de_novo_biosynthesis_g__Bacteroides_s__Bacteroides_s
PWY_7221__guanosine_ribonucleotides_de_novo_biosynthesis_g__Bacteroides_s__Bacteroides_s
PWY_7221__guanosine_ribonucleotides_de_novo_biosynthesis_g__Bacteroides_s__Bacteroides_s
PWY_7221__guanosine_ribonucleotides_de_novo_biosynthesis_g__Bacteroides_s__Bacteroides_s
PWY_7221__guanosine_ribonucleotides_de_novo_biosynthesis_g__Bacteroides_s__Bacteroides_s
PWY_7221__guanosine_ribonucleotides_de_novo_biosynthesis_g__Bacteroides_s__Bacteroides_s
PWY_7221__guanosine_ribonucleotides_de_novo_biosynthesis_g__Bacteroides_s__Bacteroides_s
PWY_7221__guanosine_ribonucleotides_de_novo_biosynthesis_g__Bacteroides_s__Bacteroides_s
PWY_7221__guanosine_ribonucleotides_de_novo_biosynthesis_g__Bacteroides_s__Bacteroides_s
PWY_7221__guanosine_ribonucleotides_de_novo_biosynthesis_g__Bacteroides_s__Bacteroides_s
PWY_7221__guanosine_ribonucleotides_de_novo_biosynthesis_g__Bacteroides_s__Bacteroides_t
PWY_7221__guanosine_ribonucleotides_de_novo_biosynthesis_g__Bacteroides_s__Bacteroides_x
PWY_7221__guanosine_ribonucleotides_de_novo_biosynthesis_g__Barnesiella_s__Barnesiella_int
PWY_7221__guanosine_ribonucleotides_de_novo_biosynthesis_g__Bifidobacterium_s__Bifidobac
PWY_7221__guanosine_ribonucleotides_de_novo_biosynthesis_g__Bifidobacterium_s__Bifidobac
PWY_7221__guanosine_ribonucleotides_de_novo_biosynthesis_g__Bifidobacterium_s__Bifidobac
PWY_7221__guanosine_ribonucleotides_de_novo_biosynthesis_g__Bifidobacterium_s__Bifidobac
PWY_7221__guanosine_ribonucleotides_de_novo_biosynthesis_g__Bifidobacterium_s__Bifidobac
PWY_7221__guanosine_ribonucleotides_de_novo_biosynthesis_g__Bilophila_s__Bilophila_wadsw
PWY_7221__guanosine_ribonucleotides_de_novo_biosynthesis_g__Blautia_s__Blautia_hydrogeno
PWY_7221__guanosine_ribonucleotides_de_novo_biosynthesis_g__Blautia_s__Blautia_sp_KLE_17
PWY_7221__guanosine_ribonucleotides_de_novo_biosynthesis_g__Blautia_s__Ruminococcus_gna
PWY_7221__guanosine_ribonucleotides_de_novo_biosynthesis_g__Blautia_s__Ruminococcus_obe
PWY_7221__guanosine_ribonucleotides_de_novo_biosynthesis_g__Blautia_s__Ruminococcus_tor
PWY_7221__guanosine_ribonucleotides_de_novo_biosynthesis_g__Burkholderiales_noname_s__

PWY_7221__guanosine_ribonucleotides_de_novo_biosynthesis_g__Butyrivibrio_s__Butyrivibrio_c
PWY_7221__guanosine_ribonucleotides_de_novo_biosynthesis_g__Citrobacter_s__Citrobacter_fr
PWY_7221__guanosine_ribonucleotides_de_novo_biosynthesis_g__Clostridiales_noname_s__Bac
PWY_7221__guanosine_ribonucleotides_de_novo_biosynthesis_g__Clostridium_s__Clostridium_a
PWY_7221__guanosine_ribonucleotides_de_novo_biosynthesis_g__Clostridium_s__Clostridium_b
PWY_7221__guanosine_ribonucleotides_de_novo_biosynthesis_g__Clostridium_s__Clostridium_c
PWY_7221__guanosine_ribonucleotides_de_novo_biosynthesis_g__Clostridium_s__Clostridium_c
PWY_7221__guanosine_ribonucleotides_de_novo_biosynthesis_g__Clostridium_s__Clostridium_h
PWY_7221__guanosine_ribonucleotides_de_novo_biosynthesis_g__Clostridium_s__Clostridium_le
PWY_7221__guanosine_ribonucleotides_de_novo_biosynthesis_g__Clostridium_s__Clostridium_p
PWY_7221__guanosine_ribonucleotides_de_novo_biosynthesis_g__Clostridium_s__Clostridium_s
PWY_7221__guanosine_ribonucleotides_de_novo_biosynthesis_g__Collinsella_s__Collinsella_tana
PWY_7221__guanosine_ribonucleotides_de_novo_biosynthesis_g__Coprobacter_s__Coprobacter_
PWY_7221__guanosine_ribonucleotides_de_novo_biosynthesis_g__Coprococcus_s__Coprococcus_
PWY_7221__guanosine_ribonucleotides_de_novo_biosynthesis_g__Coprococcus_s__Coprococcus_
PWY_7221__guanosine_ribonucleotides_de_novo_biosynthesis_g__Dialister_s__Dialister_invisus
PWY_7221__guanosine_ribonucleotides_de_novo_biosynthesis_g__Eggerthella_s__Eggerthella_le
PWY_7221__guanosine_ribonucleotides_de_novo_biosynthesis_g__Eggerthella_s__Eggerthella_sp
PWY_7221__guanosine_ribonucleotides_de_novo_biosynthesis_g__Eggerthella_s__Eggerthella_sp
PWY_7221__guanosine_ribonucleotides_de_novo_biosynthesis_g__Enterobacter_s__Enterobacter
PWY_7221__guanosine_ribonucleotides_de_novo_biosynthesis_g__Enterococcus_s__Enterococcu
PWY_7221__guanosine_ribonucleotides_de_novo_biosynthesis_g__Enterococcus_s__Enterococcu
PWY_7221__guanosine_ribonucleotides_de_novo_biosynthesis_g__Enterococcus_s__Enterococcu
PWY_7221__guanosine_ribonucleotides_de_novo_biosynthesis_g__Eubacterium_s__Eubacterium_
PWY_7221__guanosine_ribonucleotides_de_novo_biosynthesis_g__Eubacterium_s__Eubacterium_
PWY_7221__guanosine_ribonucleotides_de_novo_biosynthesis_g__Eubacterium_s__Eubacterium_
PWY_7221__guanosine_ribonucleotides_de_novo_biosynthesis_g__Eubacterium_s__Eubacterium_
PWY_7221__guanosine_ribonucleotides_de_novo_biosynthesis_g__Eubacterium_s__Eubacterium_
PWY_7221__guanosine_ribonucleotides_de_novo_biosynthesis_g__Gemella_s__Gemella_sanguin
PWY_7221__guanosine_ribonucleotides_de_novo_biosynthesis_g__Holdemania_s__Holdemania_
PWY_7221__guanosine_ribonucleotides_de_novo_biosynthesis_g__Klebsiella_s__Klebsiella_pneu
PWY_7221__guanosine_ribonucleotides_de_novo_biosynthesis_g__Lachnospiraceae_noname_s__
PWY_7221__guanosine_ribonucleotides_de_novo_biosynthesis_g__Lachnospiraceae_noname_s__
PWY_7221__guanosine_ribonucleotides_de_novo_biosynthesis_g__Lachnospiraceae_noname_s__
PWY_7221__guanosine_ribonucleotides_de_novo_biosynthesis_g__Lachnospiraceae_noname_s__
PWY_7221__guanosine_ribonucleotides_de_novo_biosynthesis_g__Lachnospiraceae_noname_s__
PWY_7221__guanosine_ribonucleotides_de_novo_biosynthesis_g__Lachnospiraceae_noname_s__
PWY_7221__guanosine_ribonucleotides_de_novo_biosynthesis_g__Lachnospiraceae_noname_s__
PWY_7221__guanosine_ribonucleotides_de_novo_biosynthesis_g__Lactobacillus_s__Lactobacillus
PWY_7221__guanosine_ribonucleotides_de_novo_biosynthesis_g__Lactobacillus_s__Lactobacillus
PWY_7221__guanosine_ribonucleotides_de_novo_biosynthesis_g__Lactococcus_s__Lactococcus_l
PWY_7221__guanosine_ribonucleotides_de_novo_biosynthesis_g__Methanobrevibacter_s__Meth
PWY_7221__guanosine_ribonucleotides_de_novo_biosynthesis_g__Nakaseomyces_s__Candida_g
PWY_7221__guanosine_ribonucleotides_de_novo_biosynthesis_g__Odoribacter_s__Odoribacter_l
PWY_7221__guanosine_ribonucleotides_de_novo_biosynthesis_g__Odoribacter_s__Odoribacter_s
PWY_7221__guanosine_ribonucleotides_de_novo_biosynthesis_g__Oxalobacter_s__Oxalobacter_
PWY_7221__guanosine_ribonucleotides_de_novo_biosynthesis_g__Parabacteroides_s__Parabacte

PWY_7221__guanosine_ribonucleotides_de_novo_biosynthesis_g__Parabacteroides_s__Parabacte
PWY_7221__guanosine_ribonucleotides_de_novo_biosynthesis_g__Parabacteroides_s__Parabacte
PWY_7221__guanosine_ribonucleotides_de_novo_biosynthesis_g__Parabacteroides_s__Parabacte
PWY_7221__guanosine_ribonucleotides_de_novo_biosynthesis_g__Parabacteroides_s__Parabacte
PWY_7221__guanosine_ribonucleotides_de_novo_biosynthesis_g__Paraprevotella_s__Paraprevot
PWY_7221__guanosine_ribonucleotides_de_novo_biosynthesis_g__Parasutterella_s__Parasuttere
PWY_7221__guanosine_ribonucleotides_de_novo_biosynthesis_g__Pediococcus_s__Pediococcus_
PWY_7221__guanosine_ribonucleotides_de_novo_biosynthesis_g__Pediococcus_s__Pediococcus_
PWY_7221__guanosine_ribonucleotides_de_novo_biosynthesis_g__Peptostreptococcaceae_nonar
PWY_7221__guanosine_ribonucleotides_de_novo_biosynthesis_g__Phascolarctobacterium_s__Ph
PWY_7221__guanosine_ribonucleotides_de_novo_biosynthesis_g__Prevotella_s__Prevotella_copr
PWY_7221__guanosine_ribonucleotides_de_novo_biosynthesis_g__Propionibacterium_s__Propior
PWY_7221__guanosine_ribonucleotides_de_novo_biosynthesis_g__Roseburia_s__Roseburia_inuli
PWY_7221__guanosine_ribonucleotides_de_novo_biosynthesis_g__Rothia_s__Rothia_mucilagino
PWY_7221__guanosine_ribonucleotides_de_novo_biosynthesis_g__Ruminococcaceae_noname_s_
PWY_7221__guanosine_ribonucleotides_de_novo_biosynthesis_g__Ruminococcus_s__Ruminococ
PWY_7221__guanosine_ribonucleotides_de_novo_biosynthesis_g__Ruminococcus_s__Ruminococ
PWY_7221__guanosine_ribonucleotides_de_novo_biosynthesis_g__Slackia_s__Slackia_piriformis
PWY_7221__guanosine_ribonucleotides_de_novo_biosynthesis_g__Solobacterium_s__Solobacteri
PWY_7221__guanosine_ribonucleotides_de_novo_biosynthesis_g__Streptococcus_s__Streptococc
PWY_7221__guanosine_ribonucleotides_de_novo_biosynthesis_g__Streptococcus_s__Streptococc
PWY_7221__guanosine_ribonucleotides_de_novo_biosynthesis_g__Veillonella_s__Veillonella_disp
PWY_7221__guanosine_ribonucleotides_de_novo_biosynthesis_g__Veillonella_s__Veillonella_par
PWY_7222__guanosine_deoxyribonucleotides_de_novo_biosynthesis_II_g__Actinomyces_s__Actir
PWY_7222__guanosine_deoxyribonucleotides_de_novo_biosynthesis_II_g__Bacteroides_s__Bacte
PWY_7222__guanosine_deoxyribonucleotides_de_novo_biosynthesis_II_g__Bacteroides_s__Bacte
PWY_7222__guanosine_deoxyribonucleotides_de_novo_biosynthesis_II_g__Bacteroides_s__Bacte
PWY_7222__guanosine_deoxyribonucleotides_de_novo_biosynthesis_II_g__Bacteroides_s__Bacte
PWY_7222__guanosine_deoxyribonucleotides_de_novo_biosynthesis_II_g__Bacteroides_s__Bacte
PWY_7222__guanosine_deoxyribonucleotides_de_novo_biosynthesis_II_g__Bacteroides_s__Bacte
PWY_7222__guanosine_deoxyribonucleotides_de_novo_biosynthesis_II_g__Burkholderiales_nona
PWY_7222__guanosine_deoxyribonucleotides_de_novo_biosynthesis_II_g__Citrobacter_s__Citrob
PWY_7222__guanosine_deoxyribonucleotides_de_novo_biosynthesis_II_g__Clostridiales_noname
PWY_7222__guanosine_deoxyribonucleotides_de_novo_biosynthesis_II_g__Clostridium_s__Clostr
PWY_7222__guanosine_deoxyribonucleotides_de_novo_biosynthesis_II_g__Clostridium_s__Clostr
PWY_7222__guanosine_deoxyribonucleotides_de_novo_biosynthesis_II_g__Collinsella_s__Collins
PWY_7222__guanosine_deoxyribonucleotides_de_novo_biosynthesis_II_g__Collinsella_s__Collins
PWY_7222__guanosine_deoxyribonucleotides_de_novo_biosynthesis_II_g__Eggerthella_s__Egger
PWY_7222__guanosine_deoxyribonucleotides_de_novo_biosynthesis_II_g__Eggerthella_s__Egger
PWY_7222__guanosine_deoxyribonucleotides_de_novo_biosynthesis_II_g__Eggerthella_s__Egger
PWY_7222__guanosine_deoxyribonucleotides_de_novo_biosynthesis_II_g__Faecalibacterium_s__
PWY_7222__guanosine_deoxyribonucleotides_de_novo_biosynthesis_II_g__Holdemania_s__Hold
PWY_7222__guanosine_deoxyribonucleotides_de_novo_biosynthesis_II_g__Klebsiella_s__Klebsiel
PWY_7222__guanosine_deoxyribonucleotides_de_novo_biosynthesis_II_g__Oxalobacter_s__Oxalo
PWY_7222__guanosine_deoxyribonucleotides_de_novo_biosynthesis_II_g__Parasutterella_s__Par
PWY_7222__guanosine_deoxyribonucleotides_de_novo_biosynthesis_II_g__Slackia_s__Slackia_pi
PWY_7222__guanosine_deoxyribonucleotides_de_novo_biosynthesis_II_g__Solobacterium_s__So

PWY_7222__guanosine_deoxyribonucleotides_de_novo_biosynthesis_II_g__Streptococcus_s__Str
PWY_7222__guanosine_deoxyribonucleotides_de_novo_biosynthesis_II_g__Streptococcus_s__Str
PWY_7222__guanosine_deoxyribonucleotides_de_novo_biosynthesis_II_g__Veillonella_s__Veillo
PWY_7222__guanosine_deoxyribonucleotides_de_novo_biosynthesis_II_g__Veillonella_s__Veillo
PWY_7228__superpathway_of_guanosine_nucleotides_de_novo_biosynthesis_I_g__Actinomyces_
PWY_7228__superpathway_of_guanosine_nucleotides_de_novo_biosynthesis_I_g__Akkermansia_
PWY_7228__superpathway_of_guanosine_nucleotides_de_novo_biosynthesis_I_g__Bacteroides_s
PWY_7228__superpathway_of_guanosine_nucleotides_de_novo_biosynthesis_I_g__Bacteroides_s
PWY_7228__superpathway_of_guanosine_nucleotides_de_novo_biosynthesis_I_g__Bacteroides_s
PWY_7228__superpathway_of_guanosine_nucleotides_de_novo_biosynthesis_I_g__Bacteroides_s
PWY_7228__superpathway_of_guanosine_nucleotides_de_novo_biosynthesis_I_g__Bacteroides_s
PWY_7228__superpathway_of_guanosine_nucleotides_de_novo_biosynthesis_I_g__Bacteroides_s
PWY_7228__superpathway_of_guanosine_nucleotides_de_novo_biosynthesis_I_g__Bacteroides_s
PWY_7228__superpathway_of_guanosine_nucleotides_de_novo_biosynthesis_I_g__Bacteroides_s
PWY_7228__superpathway_of_guanosine_nucleotides_de_novo_biosynthesis_I_g__Bacteroides_s
PWY_7228__superpathway_of_guanosine_nucleotides_de_novo_biosynthesis_I_g__Bilophila_s__
PWY_7228__superpathway_of_guanosine_nucleotides_de_novo_biosynthesis_I_g__Burkholderial
PWY_7228__superpathway_of_guanosine_nucleotides_de_novo_biosynthesis_I_g__Citrobacter_s
PWY_7228__superpathway_of_guanosine_nucleotides_de_novo_biosynthesis_I_g__Clostridium_s
PWY_7228__superpathway_of_guanosine_nucleotides_de_novo_biosynthesis_I_g__Collinsella_s_
PWY_7228__superpathway_of_guanosine_nucleotides_de_novo_biosynthesis_I_g__Dialister_s__
PWY_7228__superpathway_of_guanosine_nucleotides_de_novo_biosynthesis_I_g__Eggerthella_s
PWY_7228__superpathway_of_guanosine_nucleotides_de_novo_biosynthesis_I_g__Eggerthella_s
PWY_7228__superpathway_of_guanosine_nucleotides_de_novo_biosynthesis_I_g__Eggerthella_s
PWY_7228__superpathway_of_guanosine_nucleotides_de_novo_biosynthesis_I_g__Enterobacter_
PWY_7228__superpathway_of_guanosine_nucleotides_de_novo_biosynthesis_I_g__Klebsiella_s__
PWY_7228__superpathway_of_guanosine_nucleotides_de_novo_biosynthesis_I_g__Oxalobacter_
PWY_7228__superpathway_of_guanosine_nucleotides_de_novo_biosynthesis_I_g__Parabacteroid
PWY_7228__superpathway_of_guanosine_nucleotides_de_novo_biosynthesis_I_g__Parabacteroid
PWY_7228__superpathway_of_guanosine_nucleotides_de_novo_biosynthesis_I_g__Parabacteroid
PWY_7228__superpathway_of_guanosine_nucleotides_de_novo_biosynthesis_I_g__Parabacteroid
PWY_7228__superpathway_of_guanosine_nucleotides_de_novo_biosynthesis_I_g__Parabacteroid
PWY_7228__superpathway_of_guanosine_nucleotides_de_novo_biosynthesis_I_g__Parasutterella
PWY_7228__superpathway_of_guanosine_nucleotides_de_novo_biosynthesis_I_g__Pediococcus_
PWY_7228__superpathway_of_guanosine_nucleotides_de_novo_biosynthesis_I_g__Propionibacte
PWY_7228__superpathway_of_guanosine_nucleotides_de_novo_biosynthesis_I_g__Slackia_s__Sl
PWY_7228__superpathway_of_guanosine_nucleotides_de_novo_biosynthesis_I_g__Streptococcus
PWY_7228__superpathway_of_guanosine_nucleotides_de_novo_biosynthesis_I_g__Veillonella_s_
PWY_7228__superpathway_of_guanosine_nucleotides_de_novo_biosynthesis_I_g__Veillonella_s_
PWY_7229__superpathway_of_adenosine_nucleotides_de_novo_biosynthesis_I_g__Bacteroides_s
PWY_7229__superpathway_of_adenosine_nucleotides_de_novo_biosynthesis_I_g__Bacteroides_s
PWY_7229__superpathway_of_adenosine_nucleotides_de_novo_biosynthesis_I_g__Bacteroides_s
PWY_7229__superpathway_of_adenosine_nucleotides_de_novo_biosynthesis_I_g__Bacteroides_s
PWY_7229__superpathway_of_adenosine_nucleotides_de_novo_biosynthesis_I_g__Bacteroides_s
PWY_7229__superpathway_of_adenosine_nucleotides_de_novo_biosynthesis_I_g__Bacteroides_s
PWY_7229__superpathway_of_adenosine_nucleotides_de_novo_biosynthesis_I_g__Citrobacter_s
PWY_7229__superpathway_of_adenosine_nucleotides_de_novo_biosynthesis_I_g__Clostridiales_

PWY_7229__superpathway_of_adenosine_nucleotides_de_novo_biosynthesis_I_g__Clostridium_s
PWY_7229__superpathway_of_adenosine_nucleotides_de_novo_biosynthesis_I_g__Clostridium_s
PWY_7229__superpathway_of_adenosine_nucleotides_de_novo_biosynthesis_I_g__Collinsella_s_
PWY_7229__superpathway_of_adenosine_nucleotides_de_novo_biosynthesis_I_g__Collinsella_s_
PWY_7229__superpathway_of_adenosine_nucleotides_de_novo_biosynthesis_I_g__Eggerthella_s
PWY_7229__superpathway_of_adenosine_nucleotides_de_novo_biosynthesis_I_g__Eggerthella_s
PWY_7229__superpathway_of_adenosine_nucleotides_de_novo_biosynthesis_I_g__Eggerthella_s
PWY_7229__superpathway_of_adenosine_nucleotides_de_novo_biosynthesis_I_g__Faecalibacteri
PWY_7229__superpathway_of_adenosine_nucleotides_de_novo_biosynthesis_I_g__Holdemania_
PWY_7229__superpathway_of_adenosine_nucleotides_de_novo_biosynthesis_I_g__Klebsiella_s__
PWY_7229__superpathway_of_adenosine_nucleotides_de_novo_biosynthesis_I_g__Oxalobacter_
PWY_7229__superpathway_of_adenosine_nucleotides_de_novo_biosynthesis_I_g__Slackia_s__Sl
PWY_7229__superpathway_of_adenosine_nucleotides_de_novo_biosynthesis_I_g__Solobacteriun
PWY_7229__superpathway_of_adenosine_nucleotides_de_novo_biosynthesis_I_g__Streptococcus
PWY_7229__superpathway_of_adenosine_nucleotides_de_novo_biosynthesis_I_g__Veillonella_s_
PWY_7229__superpathway_of_adenosine_nucleotides_de_novo_biosynthesis_I_g__Veillonella_s_
PWY_7234__inosine_5__phosphate_biosynthesis_III_g__Citrobacter_s__Citrobacter_freundii
PWY_7234__inosine_5__phosphate_biosynthesis_III_g__Coprococcus_s__Coprococcus_catus
PWY_7234__inosine_5__phosphate_biosynthesis_III_g__Enterococcus_s__Enterococcus_faecium
PWY_7234__inosine_5__phosphate_biosynthesis_III_g__Lactobacillus_s__Lactobacillus_casei_para
PWY_7234__inosine_5__phosphate_biosynthesis_III_g__Lactobacillus_s__Lactobacillus_salivarius
PWY_7234__inosine_5__phosphate_biosynthesis_III_g__Lactococcus_s__Lactococcus_lactis
PWY_7234__inosine_5__phosphate_biosynthesis_III_g__Streptococcus_s__Streptococcus_mitis_o
PWY_7237__myo_,_chiro__and_scillo_inositol_degradation_g__Blautia_s__Blautia_sp_KLE_1732
PWY_7237__myo_,_chiro__and_scillo_inositol_degradation_g__Clostridium_s__Clostridium_perfr
PWY_7237__myo_,_chiro__and_scillo_inositol_degradation_g__Clostridium_s__Clostridium_sp_S
PWY_7237__myo_,_chiro__and_scillo_inositol_degradation_g__Klebsiella_s__Klebsiella_pneumon
PWY_7237__myo_,_chiro__and_scillo_inositol_degradation_g__Lachnospiraceae_noname_s__Lac
PWY_7237__myo_,_chiro__and_scillo_inositol_degradation_g__Ruminococcus_s__Ruminococcus_
PWY_7242__D_fructuronate_degradation_g__Citrobacter_s__Citrobacter_freundii
PWY_7242__D_fructuronate_degradation_g__Lactococcus_s__Lactococcus_lactis
PWY_724__superpathway_of_L_lysine,_L_threonine_and_L_methionine_biosynthesis_II_g__Anae
PWY_724__superpathway_of_L_lysine,_L_threonine_and_L_methionine_biosynthesis_II_g__Citrol
PWY_724__superpathway_of_L_lysine,_L_threonine_and_L_methionine_biosynthesis_II_g__Copro
PWY_724__superpathway_of_L_lysine,_L_threonine_and_L_methionine_biosynthesis_II_g__Copro
PWY_724__superpathway_of_L_lysine,_L_threonine_and_L_methionine_biosynthesis_II_g__Lacto
PWY_724__superpathway_of_L_lysine,_L_threonine_and_L_methionine_biosynthesis_II_g__Mega
PWY_724__superpathway_of_L_lysine,_L_threonine_and_L_methionine_biosynthesis_II_g__Oxalo
PWY_724__superpathway_of_L_lysine,_L_threonine_and_L_methionine_biosynthesis_II_g__Phaso
PWY_724__superpathway_of_L_lysine,_L_threonine_and_L_methionine_biosynthesis_II_g__Rothi
PWY_7254__TCA_cycle_VII_(acetate_producers)_g__Citrobacter_s__Citrobacter_freundii
PWY_7254__TCA_cycle_VII_(acetate_producers)_unclassified
PWY_7269__NAD_NADP_NADH_NADPH_mitochondrial_interconversion_(yeast)_unclassified
PWY_7279__aerobic_respiration_II_(cytochrome_c)_(yeast)_g__Nakaseomyces_s__Candida_glabr
PWY_7282__4_amino_2_methyl_5_phosphomethylpyrimidine_biosynthesis_(yeast)_g__Bacteroid
PWY_7282__4_amino_2_methyl_5_phosphomethylpyrimidine_biosynthesis_(yeast)_g__Bacteroid
PWY_7282__4_amino_2_methyl_5_phosphomethylpyrimidine_biosynthesis_(yeast)_g__Bacteroid

PWY_7282__4_amino_2_methyl_5_phosphomethylpyrimidine_biosynthesis_(yeast)_g__Bacteroid
PWY_7282__4_amino_2_methyl_5_phosphomethylpyrimidine_biosynthesis_(yeast)_g__Bacteroid
PWY_7282__4_amino_2_methyl_5_phosphomethylpyrimidine_biosynthesis_(yeast)_g__Bacteroid
PWY_7282__4_amino_2_methyl_5_phosphomethylpyrimidine_biosynthesis_(yeast)_g__Bacteroid
PWY_7282__4_amino_2_methyl_5_phosphomethylpyrimidine_biosynthesis_(yeast)_g__Citrobact
PWY_7283__wybutosine_biosynthesis
PWY_7288__fatty_acid_&beta__oxidation_(peroxisome,_yeast)
PWY_7288__fatty_acid_&beta__oxidation_(peroxisome,_yeast)_unclassified
PWY_7294__xylose_degradation_IV
PWY_7315__dTDP_N_acetylthomosamine_biosynthesis_g__Citrobacter_s__Citrobacter_freundii
PWY_7315__dTDP_N_acetylthomosamine_biosynthesis_unclassified
PWY_7323__superpathway_of_GDP_mannose_derived_O_antigen_building_blocks_biosynthesis_
PWY_7323__superpathway_of_GDP_mannose_derived_O_antigen_building_blocks_biosynthesis_
PWY_7323__superpathway_of_GDP_mannose_derived_O_antigen_building_blocks_biosynthesis_
PWY_7323__superpathway_of_GDP_mannose_derived_O_antigen_building_blocks_biosynthesis_
PWY_7323__superpathway_of_GDP_mannose_derived_O_antigen_building_blocks_biosynthesis_
PWY_7323__superpathway_of_GDP_mannose_derived_O_antigen_building_blocks_biosynthesis_
PWY_7345__superpathway_of_anaerobic_sucrose_degradation
PWY_7345__superpathway_of_anaerobic_sucrose_degradation_unclassified
PWY_7357__thiamin_formation_from_pyrithiamine_and_oxythiamine_(yeast)_g__Anaerofustis_s_
PWY_7357__thiamin_formation_from_pyrithiamine_and_oxythiamine_(yeast)_g__Anaerotruncus_
PWY_7357__thiamin_formation_from_pyrithiamine_and_oxythiamine_(yeast)_g__Bifidobacteriun
PWY_7357__thiamin_formation_from_pyrithiamine_and_oxythiamine_(yeast)_g__Blautia_s__Rur
PWY_7357__thiamin_formation_from_pyrithiamine_and_oxythiamine_(yeast)_g__Citrobacter_s_
PWY_7357__thiamin_formation_from_pyrithiamine_and_oxythiamine_(yeast)_g__Clostridium_s_
PWY_7357__thiamin_formation_from_pyrithiamine_and_oxythiamine_(yeast)_g__Coprococcus_s_
PWY_7357__thiamin_formation_from_pyrithiamine_and_oxythiamine_(yeast)_g__Enterococcus_s
PWY_7357__thiamin_formation_from_pyrithiamine_and_oxythiamine_(yeast)_g__Erysipelotricha
PWY_7357__thiamin_formation_from_pyrithiamine_and_oxythiamine_(yeast)_g__Erysipelotricha
PWY_7357__thiamin_formation_from_pyrithiamine_and_oxythiamine_(yeast)_g__Eubacterium_s_
PWY_7357__thiamin_formation_from_pyrithiamine_and_oxythiamine_(yeast)_g__Fusobacterium_
PWY_7357__thiamin_formation_from_pyrithiamine_and_oxythiamine_(yeast)_g__Klebsiella_s__K
PWY_7357__thiamin_formation_from_pyrithiamine_and_oxythiamine_(yeast)_g__Lachnospiracea
PWY_7357__thiamin_formation_from_pyrithiamine_and_oxythiamine_(yeast)_g__Pediococcus_s_
PWY_7357__thiamin_formation_from_pyrithiamine_and_oxythiamine_(yeast)_g__Pediococcus_s_
PWY_7357__thiamin_formation_from_pyrithiamine_and_oxythiamine_(yeast)_g__Phascolarctoba
PWY_7357__thiamin_formation_from_pyrithiamine_and_oxythiamine_(yeast)_g__Propionibacteri
PWY_7357__thiamin_formation_from_pyrithiamine_and_oxythiamine_(yeast)_g__Roseburia_s__I
PWY_7357__thiamin_formation_from_pyrithiamine_and_oxythiamine_(yeast)_g__Roseburia_s__I
PWY_7357__thiamin_formation_from_pyrithiamine_and_oxythiamine_(yeast)_g__Ruminococcus_
PWY_7357__thiamin_formation_from_pyrithiamine_and_oxythiamine_(yeast)_g__Streptococcus_
PWY_7371__1,4_dihydroxy_6_naphthoate_biosynthesis_II
PWY_7371__1,4_dihydroxy_6_naphthoate_biosynthesis_II_g__Alistipes_s__Alistipes_finegoldii
PWY_7371__1,4_dihydroxy_6_naphthoate_biosynthesis_II_g__Alistipes_s__Alistipes_sp_HGB5
PWY_7371__1,4_dihydroxy_6_naphthoate_biosynthesis_II_unclassified
PWY_7374__1,4_dihydroxy_6_naphthoate_biosynthesis_I
PWY_7374__1,4_dihydroxy_6_naphthoate_biosynthesis_I_unclassified

PWY_7384__anaerobic_energy_metabolism_(invertebrates,_mitochondrial)
PWY_7385__1,3_propanediol_biosynthesis_(engineered)
PWY_7388__octanoyl_[acyl_carrier_protein]_biosynthesis_(mitochondria,_yeast)_g__Bacteroides_
PWY_7388__octanoyl_[acyl_carrier_protein]_biosynthesis_(mitochondria,_yeast)_g__Bacteroides_
PWY_7388__octanoyl_[acyl_carrier_protein]_biosynthesis_(mitochondria,_yeast)_g__Citrobacter_
PWY_7388__octanoyl_[acyl_carrier_protein]_biosynthesis_(mitochondria,_yeast)_g__Haemophilu
PWY_7388__octanoyl_[acyl_carrier_protein]_biosynthesis_(mitochondria,_yeast)_g__Klebsiella_s_
PWY_7389__superpathway_of_anaerobic_energy_metabolism_(invertebrates)
PWY_7399__methylphosphonate_degradation_II
PWY_7400__L_arginine_biosynthesis_IV_(archaebacteria)_g__Burkholderiales_noname_s__Burkh
PWY_7400__L_arginine_biosynthesis_IV_(archaebacteria)_g__Coprococcus_s__Coprococcus_catus
PWY_7400__L_arginine_biosynthesis_IV_(archaebacteria)_g__Eubacterium_s__Eubacterium_sirae
PWY_7400__L_arginine_biosynthesis_IV_(archaebacteria)_g__Lactococcus_s__Lactococcus_lactis
PWY_7400__L_arginine_biosynthesis_IV_(archaebacteria)_g__Oxalobacter_s__Oxalobacter_formig
PWY_7400__L_arginine_biosynthesis_IV_(archaebacteria)_g__Parasutterella_s__Parasutterella_ex
PWY_7400__L_arginine_biosynthesis_IV_(archaebacteria)_g__Ruminococcus_s__Ruminococcus_c
PWY_7400__L_arginine_biosynthesis_IV_(archaebacteria)_g__Veillonella_s__Veillonella_dispar
PWY_7400__L_arginine_biosynthesis_IV_(archaebacteria)_g__Veillonella_s__Veillonella_parvula
PWY_7409__phospholipid_remodeling_(phosphatidylethanolamine,_yeast)_unclassified
PWY_7431__aromatic_biogenic_amine_degradation_(bacteria)
PWY_7446__sulfoglycolysis_g__Citrobacter_s__Citrobacter_freundii
PWY_7446__sulfoglycolysis_unclassified
PWY_7456__mannan_degradation_g__Bacteroides_s__Bacteroides_ovatus
PWY_7456__mannan_degradation_g__Coprococcus_s__Coprococcus_eutactus
PWY_7456__mannan_degradation_g__Coprococcus_s__Coprococcus_sp_ART55_1
PWY_7456__mannan_degradation_g__Eubacterium_s__Eubacterium_siraeum
PWY_7456__mannan_degradation_unclassified
PWY_7527__L_methionine_salvage_cycle_III
PWY_7527__L_methionine_salvage_cycle_III_unclassified
PWY_7539__6_hydroxymethyl_dihydropterin_diphosphate_biosynthesis_III_(Chlamydia)_g__Bact
PWY_7539__6_hydroxymethyl_dihydropterin_diphosphate_biosynthesis_III_(Chlamydia)_g__Bact
PWY_7539__6_hydroxymethyl_dihydropterin_diphosphate_biosynthesis_III_(Chlamydia)_g__Bact
PWY_7539__6_hydroxymethyl_dihydropterin_diphosphate_biosynthesis_III_(Chlamydia)_g__Bact
PWY_7539__6_hydroxymethyl_dihydropterin_diphosphate_biosynthesis_III_(Chlamydia)_g__Bact
PWY_7539__6_hydroxymethyl_dihydropterin_diphosphate_biosynthesis_III_(Chlamydia)_g__Bact
PWY_7539__6_hydroxymethyl_dihydropterin_diphosphate_biosynthesis_III_(Chlamydia)_g__Citro
PWY_7560__methylerythritol_phosphate_pathway_II_g__Citrobacter_s__Citrobacter_freundii
PWY_7560__methylerythritol_phosphate_pathway_II_g__Klebsiella_s__Klebsiella_pneumoniae
PWY_7560__methylerythritol_phosphate_pathway_II_g__Phascolarctobacterium_s__Phascolarcto
PWY_7663__gondoate_biosynthesis_(anaerobic)_g__Bacteroides_s__Bacteroides_eggerthii
PWY_7663__gondoate_biosynthesis_(anaerobic)_g__Bacteroides_s__Bacteroides_faecis
PWY_7663__gondoate_biosynthesis_(anaerobic)_g__Bacteroides_s__Bacteroides_finegoldii
PWY_7663__gondoate_biosynthesis_(anaerobic)_g__Bacteroides_s__Bacteroides_ovatus
PWY_7663__gondoate_biosynthesis_(anaerobic)_g__Bacteroides_s__Bacteroides_salyersiae
PWY_7663__gondoate_biosynthesis_(anaerobic)_g__Bacteroides_s__Bacteroides_sp_1_1_30
PWY_7663__gondoate_biosynthesis_(anaerobic)_g__Bacteroides_s__Bacteroides_sp_1_1_6
PWY_7663__gondoate_biosynthesis_(anaerobic)_g__Bacteroides_s__Bacteroides_sp_2_1_22

PWY_7663__gondoate_biosynthesis_(anaerobic)_g__Bacteroides_s__Bacteroides_sp_2_2_4
PWY_7663__gondoate_biosynthesis_(anaerobic)_g__Bacteroides_s__Bacteroides_sp_3_1_23
PWY_7663__gondoate_biosynthesis_(anaerobic)_g__Bacteroides_s__Bacteroides_stercoris
PWY_7663__gondoate_biosynthesis_(anaerobic)_g__Bacteroides_s__Bacteroides_thetaiotaomicro
PWY_7663__gondoate_biosynthesis_(anaerobic)_g__Bacteroides_s__Bacteroides_xylanisolvens
PWY_7663__gondoate_biosynthesis_(anaerobic)_g__Citrobacter_s__Citrobacter_freundii
PWY_7663__gondoate_biosynthesis_(anaerobic)_g__Enterobacter_s__Enterobacter_cloacae
PWY_7663__gondoate_biosynthesis_(anaerobic)_g__Klebsiella_s__Klebsiella_pneumoniae
PWY_7663__gondoate_biosynthesis_(anaerobic)_g__Lactococcus_s__Lactococcus_lactis
PWY_7663__gondoate_biosynthesis_(anaerobic)_g__Odoribacter_s__Odoribacter_splanchnicus
PWY_7664__oleate_biosynthesis_IV_(anaerobic)_g__Bacteroides_s__Bacteroides_sp_2_2_4
PWY_7664__oleate_biosynthesis_IV_(anaerobic)_g__Bacteroides_s__Bacteroides_xylanisolvens
PWY_7664__oleate_biosynthesis_IV_(anaerobic)_g__Citrobacter_s__Citrobacter_freundii
PWY_7664__oleate_biosynthesis_IV_(anaerobic)_g__Enterobacter_s__Enterobacter_cloacae
PWY_7664__oleate_biosynthesis_IV_(anaerobic)_g__Haemophilus_s__Haemophilus_parainfluenza
PWY_7664__oleate_biosynthesis_IV_(anaerobic)_g__Klebsiella_s__Klebsiella_pneumoniae
PWY_922__mevalonate_pathway_I_g__Lactococcus_s__Lactococcus_lactis
PWY0_1061__superpathway_of_L_alanine_biosynthesis_g__Citrobacter_s__Citrobacter_freundii
PWY0_1061__superpathway_of_L_alanine_biosynthesis_g__Enterococcus_s__Enterococcus_duran
PWY0_1061__superpathway_of_L_alanine_biosynthesis_g__Enterococcus_s__Enterococcus_faeciu
PWY0_1061__superpathway_of_L_alanine_biosynthesis_g__Klebsiella_s__Klebsiella_pneumoniae
PWY0_1061__superpathway_of_L_alanine_biosynthesis_g__Streptococcus_s__Streptococcus_miti
PWY0_1061__superpathway_of_L_alanine_biosynthesis_g__Streptococcus_s__Streptococcus_sang
PWY0_1241__ADP_L_glycero_&beta__D_manno_heptose_biosynthesis_g__Citrobacter_s__Citrob
PWY0_1241__ADP_L_glycero_&beta__D_manno_heptose_biosynthesis_g__Enterobacter_s__Ente
PWY0_1277__3_phenylpropanoate_and_3_(3_hydroxyphenyl)propanoate_degradation_g__Citrob
PWY0_1277__3_phenylpropanoate_and_3_(3_hydroxyphenyl)propanoate_degradation_g__Klebsi
PWY0_1296__purine_ribonucleosides_degradation_g__Anaerotruncus_s__Anaerotruncus_colihom
PWY0_1296__purine_ribonucleosides_degradation_g__Blautia_s__Blautia_sp_KLE_1732
PWY0_1296__purine_ribonucleosides_degradation_g__Blautia_s__Ruminococcus_gnavus
PWY0_1296__purine_ribonucleosides_degradation_g__Blautia_s__Ruminococcus_obeum
PWY0_1296__purine_ribonucleosides_degradation_g__Butyrivibrio_s__Butyrivibrio_crossotus
PWY0_1296__purine_ribonucleosides_degradation_g__Catenibacterium_s__Catenibacterium_mit
PWY0_1296__purine_ribonucleosides_degradation_g__Citrobacter_s__Citrobacter_freundii
PWY0_1296__purine_ribonucleosides_degradation_g__Clostridiales_noname_s__Bacteroides_pec
PWY0_1296__purine_ribonucleosides_degradation_g__Clostridiales_noname_s__Clostridiales_bac
PWY0_1296__purine_ribonucleosides_degradation_g__Clostridium_s__Clostridium_asparagiforme
PWY0_1296__purine_ribonucleosides_degradation_g__Clostridium_s__Clostridium_bolteae
PWY0_1296__purine_ribonucleosides_degradation_g__Clostridium_s__Clostridium_citroniae
PWY0_1296__purine_ribonucleosides_degradation_g__Clostridium_s__Clostridium_clostridioform
PWY0_1296__purine_ribonucleosides_degradation_g__Clostridium_s__Clostridium_hathewayi
PWY0_1296__purine_ribonucleosides_degradation_g__Clostridium_s__Clostridium_leptum
PWY0_1296__purine_ribonucleosides_degradation_g__Clostridium_s__Clostridium_perfringens
PWY0_1296__purine_ribonucleosides_degradation_g__Collinsella_s__Collinsella_intestinalis
PWY0_1296__purine_ribonucleosides_degradation_g__Collinsella_s__Collinsella_tanakaei
PWY0_1296__purine_ribonucleosides_degradation_g__Coprococcus_s__Coprococcus_catus
PWY0_1296__purine_ribonucleosides_degradation_g__Dorea_s__Dorea_formicigenerans

PWY0_1296__purine_ribonucleosides_degradation_g__Enterobacter_s__Enterobacter_cloacae
PWY0_1296__purine_ribonucleosides_degradation_g__Enterococcus_s__Enterococcus_durans
PWY0_1296__purine_ribonucleosides_degradation_g__Erysipelotrichaceae_noname_s__Clostridiu
PWY0_1296__purine_ribonucleosides_degradation_g__Erysipelotrichaceae_noname_s__Erysipelo
PWY0_1296__purine_ribonucleosides_degradation_g__Erysipelotrichaceae_noname_s__Erysipelo
PWY0_1296__purine_ribonucleosides_degradation_g__Erysipelotrichaceae_noname_s__Eubacteri
PWY0_1296__purine_ribonucleosides_degradation_g__Erysipelotrichaceae_noname_s__Eubacteri
PWY0_1296__purine_ribonucleosides_degradation_g__Eubacterium_s__Eubacterium_eligens
PWY0_1296__purine_ribonucleosides_degradation_g__Eubacterium_s__Eubacterium_ramulus
PWY0_1296__purine_ribonucleosides_degradation_g__Eubacterium_s__Eubacterium_sp_3_1_31
PWY0_1296__purine_ribonucleosides_degradation_g__Eubacterium_s__Eubacterium_ventriosum
PWY0_1296__purine_ribonucleosides_degradation_g__Holdemania_s__Holdemania_filiformis
PWY0_1296__purine_ribonucleosides_degradation_g__Klebsiella_s__Klebsiella_pneumoniae
PWY0_1296__purine_ribonucleosides_degradation_g__Lachnospiraceae_noname_s__Lachnospira
PWY0_1296__purine_ribonucleosides_degradation_g__Lachnospiraceae_noname_s__Lachnospira
PWY0_1296__purine_ribonucleosides_degradation_g__Lachnospiraceae_noname_s__Lachnospira
PWY0_1296__purine_ribonucleosides_degradation_g__Lachnospiraceae_noname_s__Lachnospira
PWY0_1296__purine_ribonucleosides_degradation_g__Lachnospiraceae_noname_s__Lachnospira
PWY0_1296__purine_ribonucleosides_degradation_g__Lachnospiraceae_noname_s__Lachnospira
PWY0_1296__purine_ribonucleosides_degradation_g__Lactobacillus_s__Lactobacillus_casei_parac
PWY0_1296__purine_ribonucleosides_degradation_g__Pediococcus_s__Pediococcus_acidilactici
PWY0_1296__purine_ribonucleosides_degradation_g__Peptostreptococcaceae_noname_s__Clostri
PWY0_1296__purine_ribonucleosides_degradation_g__Roseburia_s__Roseburia_hominis
PWY0_1296__purine_ribonucleosides_degradation_g__Roseburia_s__Roseburia_inulinivorans
PWY0_1296__purine_ribonucleosides_degradation_g__Streptococcus_s__Streptococcus_australis
PWY0_1296__purine_ribonucleosides_degradation_g__Streptococcus_s__Streptococcus_mitis_ora
PWY0_1296__purine_ribonucleosides_degradation_g__Streptococcus_s__Streptococcus_sanguinis
PWY0_1296__purine_ribonucleosides_degradation_g__Subdoligranulum_s__Subdoligranulum_sp_
PWY0_1297__superpathway_of_purine_deoxyribonucleosides_degradation_g__Anaerotruncus_s_
PWY0_1297__superpathway_of_purine_deoxyribonucleosides_degradation_g__Blautia_s__Rumin
PWY0_1297__superpathway_of_purine_deoxyribonucleosides_degradation_g__Citrobacter_s__Ci
PWY0_1297__superpathway_of_purine_deoxyribonucleosides_degradation_g__Clostridiales_nona
PWY0_1297__superpathway_of_purine_deoxyribonucleosides_degradation_g__Clostridium_s__Cl
PWY0_1297__superpathway_of_purine_deoxyribonucleosides_degradation_g__Clostridium_s__Cl
PWY0_1297__superpathway_of_purine_deoxyribonucleosides_degradation_g__Enterobacter_s__
PWY0_1297__superpathway_of_purine_deoxyribonucleosides_degradation_g__Holdemania_s__H
PWY0_1297__superpathway_of_purine_deoxyribonucleosides_degradation_g__Klebsiella_s__Kleb
PWY0_1297__superpathway_of_purine_deoxyribonucleosides_degradation_g__Lachnospiraceae_
PWY0_1297__superpathway_of_purine_deoxyribonucleosides_degradation_g__Lactobacillus_s__
PWY0_1297__superpathway_of_purine_deoxyribonucleosides_degradation_g__Streptococcus_s__
PWY0_1298__superpathway_of_pyrimidine_deoxyribonucleosides_degradation_g__Anaerostipes_
PWY0_1298__superpathway_of_pyrimidine_deoxyribonucleosides_degradation_g__Clostridium_s
PWY0_1298__superpathway_of_pyrimidine_deoxyribonucleosides_degradation_g__Lachnospirace
PWY0_1298__superpathway_of_pyrimidine_deoxyribonucleosides_degradation_g__Streptococcus
PWY0_1319__CDP_diacylglycerol_biosynthesis_II_g__Alistipes_s__Alistipes_finegoldii
PWY0_1319__CDP_diacylglycerol_biosynthesis_II_g__Alistipes_s__Alistipes_indistinctus
PWY0_1319__CDP_diacylglycerol_biosynthesis_II_g__Alistipes_s__Alistipes_sp_HGB5

PWY0_1319__CDP_diacylglycerol_biosynthesis_II_g__Anaerostipes_s__Anaerostipes_hadrus
PWY0_1319__CDP_diacylglycerol_biosynthesis_II_g__Bacteroides_s__Bacteroides_cellulosilyticus
PWY0_1319__CDP_diacylglycerol_biosynthesis_II_g__Bacteroides_s__Bacteroides_clarus
PWY0_1319__CDP_diacylglycerol_biosynthesis_II_g__Bacteroides_s__Bacteroides_coprocola
PWY0_1319__CDP_diacylglycerol_biosynthesis_II_g__Bacteroides_s__Bacteroides_eggerthii
PWY0_1319__CDP_diacylglycerol_biosynthesis_II_g__Bacteroides_s__Bacteroides_faecis
PWY0_1319__CDP_diacylglycerol_biosynthesis_II_g__Bacteroides_s__Bacteroides_finegoldii
PWY0_1319__CDP_diacylglycerol_biosynthesis_II_g__Bacteroides_s__Bacteroides_intestinalis
PWY0_1319__CDP_diacylglycerol_biosynthesis_II_g__Bacteroides_s__Bacteroides_ovatus
PWY0_1319__CDP_diacylglycerol_biosynthesis_II_g__Bacteroides_s__Bacteroides_plebeius
PWY0_1319__CDP_diacylglycerol_biosynthesis_II_g__Bacteroides_s__Bacteroides_salyersiae
PWY0_1319__CDP_diacylglycerol_biosynthesis_II_g__Bacteroides_s__Bacteroides_sp_1_1_30
PWY0_1319__CDP_diacylglycerol_biosynthesis_II_g__Bacteroides_s__Bacteroides_sp_1_1_6
PWY0_1319__CDP_diacylglycerol_biosynthesis_II_g__Bacteroides_s__Bacteroides_sp_2_1_22
PWY0_1319__CDP_diacylglycerol_biosynthesis_II_g__Bacteroides_s__Bacteroides_sp_2_2_4
PWY0_1319__CDP_diacylglycerol_biosynthesis_II_g__Bacteroides_s__Bacteroides_sp_3_1_19
PWY0_1319__CDP_diacylglycerol_biosynthesis_II_g__Bacteroides_s__Bacteroides_sp_3_1_23
PWY0_1319__CDP_diacylglycerol_biosynthesis_II_g__Bacteroides_s__Bacteroides_sp_D20
PWY0_1319__CDP_diacylglycerol_biosynthesis_II_g__Bacteroides_s__Bacteroides_stercoris
PWY0_1319__CDP_diacylglycerol_biosynthesis_II_g__Bacteroides_s__Bacteroides_thetaiotaomicr
PWY0_1319__CDP_diacylglycerol_biosynthesis_II_g__Bacteroides_s__Bacteroides_uniformis
PWY0_1319__CDP_diacylglycerol_biosynthesis_II_g__Bacteroides_s__Bacteroides_xylanisolvens
PWY0_1319__CDP_diacylglycerol_biosynthesis_II_g__Barnesiella_s__Barnesiella_intestinihominis
PWY0_1319__CDP_diacylglycerol_biosynthesis_II_g__Blautia_s__Blautia_hydrogenotrophica
PWY0_1319__CDP_diacylglycerol_biosynthesis_II_g__Blautia_s__Ruminococcus_gnavus
PWY0_1319__CDP_diacylglycerol_biosynthesis_II_g__Blautia_s__Ruminococcus_obeum
PWY0_1319__CDP_diacylglycerol_biosynthesis_II_g__Butyrivibrio_s__Butyrivibrio_crossotus
PWY0_1319__CDP_diacylglycerol_biosynthesis_II_g__Citrobacter_s__Citrobacter_freundii
PWY0_1319__CDP_diacylglycerol_biosynthesis_II_g__Clostridiales_noname_s__Clostridiales_bact
PWY0_1319__CDP_diacylglycerol_biosynthesis_II_g__Clostridium_s__Clostridium_asparagiforme
PWY0_1319__CDP_diacylglycerol_biosynthesis_II_g__Clostridium_s__Clostridium_bolteae
PWY0_1319__CDP_diacylglycerol_biosynthesis_II_g__Clostridium_s__Clostridium_clostridioforme
PWY0_1319__CDP_diacylglycerol_biosynthesis_II_g__Clostridium_s__Clostridium_hathewayi
PWY0_1319__CDP_diacylglycerol_biosynthesis_II_g__Clostridium_s__Clostridium_leptum
PWY0_1319__CDP_diacylglycerol_biosynthesis_II_g__Clostridium_s__Clostridium_sp_L2_50
PWY0_1319__CDP_diacylglycerol_biosynthesis_II_g__Clostridium_s__Clostridium_sp_SS2_1
PWY0_1319__CDP_diacylglycerol_biosynthesis_II_g__Coprobacter_s__Coprobacter_fastidiosus
PWY0_1319__CDP_diacylglycerol_biosynthesis_II_g__Coprococcus_s__Coprococcus_eutactus
PWY0_1319__CDP_diacylglycerol_biosynthesis_II_g__Dialister_s__Dialister_invisus
PWY0_1319__CDP_diacylglycerol_biosynthesis_II_g__Dorea_s__Dorea_formicigenerans
PWY0_1319__CDP_diacylglycerol_biosynthesis_II_g__Dorea_s__Dorea_longicatena
PWY0_1319__CDP_diacylglycerol_biosynthesis_II_g__Enterobacter_s__Enterobacter_cloacae
PWY0_1319__CDP_diacylglycerol_biosynthesis_II_g__Erysipelotrichaceae_noname_s__Clostridium
PWY0_1319__CDP_diacylglycerol_biosynthesis_II_g__Eubacterium_s__Eubacterium_eligens
PWY0_1319__CDP_diacylglycerol_biosynthesis_II_g__Eubacterium_s__Eubacterium_hallii
PWY0_1319__CDP_diacylglycerol_biosynthesis_II_g__Eubacterium_s__Eubacterium_ramulus
PWY0_1319__CDP_diacylglycerol_biosynthesis_II_g__Eubacterium_s__Eubacterium_siraeum

PWY0_1319__CDP_diacylglycerol_biosynthesis_II_g__Eubacterium_s__Eubacterium_ventriosum
PWY0_1319__CDP_diacylglycerol_biosynthesis_II_g__Gordonibacter_s__Gordonibacter_pamelaea
PWY0_1319__CDP_diacylglycerol_biosynthesis_II_g__Klebsiella_s__Klebsiella_pneumoniae
PWY0_1319__CDP_diacylglycerol_biosynthesis_II_g__Lachnospiraceae_noname_s__Lachnospirace
PWY0_1319__CDP_diacylglycerol_biosynthesis_II_g__Lachnospiraceae_noname_s__Lachnospirace
PWY0_1319__CDP_diacylglycerol_biosynthesis_II_g__Lachnospiraceae_noname_s__Lachnospirace
PWY0_1319__CDP_diacylglycerol_biosynthesis_II_g__Lachnospiraceae_noname_s__Lachnospirace
PWY0_1319__CDP_diacylglycerol_biosynthesis_II_g__Lachnospiraceae_noname_s__Lachnospirace
PWY0_1319__CDP_diacylglycerol_biosynthesis_II_g__Lachnospiraceae_noname_s__Lachnospirace
PWY0_1319__CDP_diacylglycerol_biosynthesis_II_g__Lachnospiraceae_noname_s__Lachnospirace
PWY0_1319__CDP_diacylglycerol_biosynthesis_II_g__Lachnospiraceae_noname_s__Lachnospirace
PWY0_1319__CDP_diacylglycerol_biosynthesis_II_g__Oxalobacter_s__Oxalobacter_formigenes
PWY0_1319__CDP_diacylglycerol_biosynthesis_II_g__Parabacteroides_s__Parabacteroides_distas
PWY0_1319__CDP_diacylglycerol_biosynthesis_II_g__Parabacteroides_s__Parabacteroides_johnso
PWY0_1319__CDP_diacylglycerol_biosynthesis_II_g__Parabacteroides_s__Parabacteroides_merda
PWY0_1319__CDP_diacylglycerol_biosynthesis_II_g__Parabacteroides_s__Parabacteroides_sp_D1
PWY0_1319__CDP_diacylglycerol_biosynthesis_II_g__Paraprevotella_s__Paraprevotella_clara
PWY0_1319__CDP_diacylglycerol_biosynthesis_II_g__Peptostreptococcaceae_noname_s__Clostric
PWY0_1319__CDP_diacylglycerol_biosynthesis_II_g__Propionibacterium_s__Propionibacterium_fr
PWY0_1319__CDP_diacylglycerol_biosynthesis_II_g__Roseburia_s__Roseburia_hominis
PWY0_1319__CDP_diacylglycerol_biosynthesis_II_g__Roseburia_s__Roseburia_inulinivorans
PWY0_1319__CDP_diacylglycerol_biosynthesis_II_g__Ruminococcus_s__Ruminococcus_champane
PWY0_1319__CDP_diacylglycerol_biosynthesis_II_g__Ruminococcus_s__Ruminococcus_sp
PWY0_1319__CDP_diacylglycerol_biosynthesis_II_g__Streptococcus_s__Streptococcus_mitis_orali
PWY0_1319__CDP_diacylglycerol_biosynthesis_II_g__Subdoligranulum_s__Subdoligranulum_sp_4
PWY0_1319__CDP_diacylglycerol_biosynthesis_II_g__Veillonella_s__Veillonella_dispar
PWY0_1319__CDP_diacylglycerol_biosynthesis_II_g__Veillonella_s__Veillonella_parvula
PWY0_1338__polymyxin_resistance_unclassified
PWY0_1415__superpathway_of_heme_biosynthesis_from_uroporphyrinogen_III_unclassified
PWY0_1479__tRNA_processing_g__Burkholderiales_noname_s__Burkholderiales_bacterium_1_1_
PWY0_1479__tRNA_processing_g__Parasutterella_s__Parasutterella_excrementihominis
PWY0_1479__tRNA_processing_unclassified
PWY0_1533__methylphosphonate_degradation_I_unclassified
PWY0_1586__peptidoglycan_maturation_(meso_diaminopimelate_containing)_g__Akkermansia_s
PWY0_1586__peptidoglycan_maturation_(meso_diaminopimelate_containing)_g__Bacteroides_s_
PWY0_1586__peptidoglycan_maturation_(meso_diaminopimelate_containing)_g__Citrobacter_s_
PWY0_1586__peptidoglycan_maturation_(meso_diaminopimelate_containing)_g__Eggerthella_s_
PWY0_1586__peptidoglycan_maturation_(meso_diaminopimelate_containing)_g__Eggerthella_s_
PWY0_1586__peptidoglycan_maturation_(meso_diaminopimelate_containing)_g__Eggerthella_s_
PWY0_1586__peptidoglycan_maturation_(meso_diaminopimelate_containing)_g__Enterobacter_s
PWY0_1586__peptidoglycan_maturation_(meso_diaminopimelate_containing)_g__Klebsiella_s__
PWY0_1586__peptidoglycan_maturation_(meso_diaminopimelate_containing)_g__Odoribacter_s_
PWY0_1586__peptidoglycan_maturation_(meso_diaminopimelate_containing)_g__Oxalobacter_s_
PWY0_1586__peptidoglycan_maturation_(meso_diaminopimelate_containing)_g__Propionibacter
PWY0_1586__peptidoglycan_maturation_(meso_diaminopimelate_containing)_g__Ruminococcus_
PWY0_1586__peptidoglycan_maturation_(meso_diaminopimelate_containing)_g__Streptococcus_
PWY0_1586__peptidoglycan_maturation_(meso_diaminopimelate_containing)_g__Streptococcus_

PWY0_1586__peptidoglycan_maturation_(meso_diaminopimelate_containing)_g__Veillonella_s__
PWY0_162__superpathway_of_pyrimidine_ribonucleotides_de_novo_biosynthesis_g__Citrobacter
PWY0_162__superpathway_of_pyrimidine_ribonucleotides_de_novo_biosynthesis_g__Klebsiella_
PWY0_162__superpathway_of_pyrimidine_ribonucleotides_de_novo_biosynthesis_g__Oxalobacte
PWY0_162__superpathway_of_pyrimidine_ribonucleotides_de_novo_biosynthesis_g__Propioniba
PWY0_166__superpathway_of_pyrimidine_deoxyribonucleotides_de_novo_biosynthesis_(E__coli)
PWY0_166__superpathway_of_pyrimidine_deoxyribonucleotides_de_novo_biosynthesis_(E__coli)
PWY0_166__superpathway_of_pyrimidine_deoxyribonucleotides_de_novo_biosynthesis_(E__coli)
PWY0_166__superpathway_of_pyrimidine_deoxyribonucleotides_de_novo_biosynthesis_(E__coli)
PWY0_166__superpathway_of_pyrimidine_deoxyribonucleotides_de_novo_biosynthesis_(E__coli)
PWY0_166__superpathway_of_pyrimidine_deoxyribonucleotides_de_novo_biosynthesis_(E__coli)
PWY0_321__phenylacetate_degradation_I_(aerobic)_g__Escherichia_s__Escherichia_coli
PWY0_321__phenylacetate_degradation_I_(aerobic)_unclassified
PWY0_42__2_methylcitrate_cycle_I
PWY0_42__2_methylcitrate_cycle_I_g__Citrobacter_s__Citrobacter_freundii
PWY0_42__2_methylcitrate_cycle_I_unclassified
PWY0_845__superpathway_of_pyridoxal_5__phosphate_biosynthesis_and_salvage_g__Bacteroide
PWY0_845__superpathway_of_pyridoxal_5__phosphate_biosynthesis_and_salvage_g__Bacteroide
PWY0_845__superpathway_of_pyridoxal_5__phosphate_biosynthesis_and_salvage_g__Bacteroide
PWY0_845__superpathway_of_pyridoxal_5__phosphate_biosynthesis_and_salvage_g__Bacteroide
PWY0_845__superpathway_of_pyridoxal_5__phosphate_biosynthesis_and_salvage_g__Bacteroide
PWY0_845__superpathway_of_pyridoxal_5__phosphate_biosynthesis_and_salvage_g__Bacteroide
PWY0_845__superpathway_of_pyridoxal_5__phosphate_biosynthesis_and_salvage_g__Bacteroide
PWY0_845__superpathway_of_pyridoxal_5__phosphate_biosynthesis_and_salvage_g__Bacteroide
PWY0_845__superpathway_of_pyridoxal_5__phosphate_biosynthesis_and_salvage_g__Bacteroide
PWY0_845__superpathway_of_pyridoxal_5__phosphate_biosynthesis_and_salvage_g__Bacteroide
PWY0_845__superpathway_of_pyridoxal_5__phosphate_biosynthesis_and_salvage_g__Bacteroide
PWY0_845__superpathway_of_pyridoxal_5__phosphate_biosynthesis_and_salvage_g__Bacteroide
PWY0_845__superpathway_of_pyridoxal_5__phosphate_biosynthesis_and_salvage_g__Citrobacte
PWY0_845__superpathway_of_pyridoxal_5__phosphate_biosynthesis_and_salvage_g__Parabacte
PWY0_862__(5Z)_dodec_5_enoate_biosynthesis_g__Bacteroides_s__Bacteroides_sp_2_2_4
PWY0_862__(5Z)_dodec_5_enoate_biosynthesis_g__Bacteroides_s__Bacteroides_xylanisolvens
PWY0_862__(5Z)_dodec_5_enoate_biosynthesis_g__Citrobacter_s__Citrobacter_freundii
PWY0_862__(5Z)_dodec_5_enoate_biosynthesis_g__Enterobacter_s__Enterobacter_cloacae
PWY0_862__(5Z)_dodec_5_enoate_biosynthesis_g__Klebsiella_s__Klebsiella_pneumoniae
PWY1F_823__leucopelargonidin_and_leucocyanidin_biosynthesis
PWY1F_823__leucopelargonidin_and_leucocyanidin_biosynthesis_g__Streptococcus_s__Streptoco
PWY3DJ_35471__L_ascorbate_biosynthesis_IV
PWY3O_19__ubiquinol_6_biosynthesis_from_4_hydroxybenzoate_(eukaryotic)
PWY4FS_7__phosphatidylglycerol_biosynthesis_I_(plastidic)_g__Citrobacter_s__Citrobacter_freun
PWY4FS_7__phosphatidylglycerol_biosynthesis_I_(plastidic)_g__Klebsiella_s__Klebsiella_pneumor
PWY4FS_8__phosphatidylglycerol_biosynthesis_II_(non_plastidic)_g__Citrobacter_s__Citrobacter_
PWY4FS_8__phosphatidylglycerol_biosynthesis_II_(non_plastidic)_g__Klebsiella_s__Klebsiella_pne
PWY66_367__ketogenesis
PWY66_375__leukotriene_biosynthesis
PWY66_375__leukotriene_biosynthesis_g__Nakaseomyces_s__Candida_glabrata
PWY66_375__leukotriene_biosynthesis_unclassified

PWY66_391__fatty_acid_&beta__oxidation_VI_(peroxisome)
PWY66_391__fatty_acid_&beta__oxidation_VI_(peroxisome)_unclassified
PWY66_400__glycolysis_VI_(metazoan)_g__Bacteroides_s__Bacteroides_ovatus
PWY66_400__glycolysis_VI_(metazoan)_g__Bacteroides_s__Bacteroides_sp_1_1_30
PWY66_400__glycolysis_VI_(metazoan)_g__Bacteroides_s__Bacteroides_sp_2_1_22
PWY66_400__glycolysis_VI_(metazoan)_g__Bacteroides_s__Bacteroides_sp_2_2_4
PWY66_400__glycolysis_VI_(metazoan)_g__Bacteroides_s__Bacteroides_sp_3_1_23
PWY66_400__glycolysis_VI_(metazoan)_g__Bacteroides_s__Bacteroides_xylanisolvens
PWY66_400__glycolysis_VI_(metazoan)_g__Lactococcus_s__Lactococcus_lactis
PWY66_400__glycolysis_VI_(metazoan)_g__Pediococcus_s__Pediococcus_acidilactici
PWY66_400__glycolysis_VI_(metazoan)_g__Pediococcus_s__Pediococcus_lolii
PWY66_400__glycolysis_VI_(metazoan)_g__Streptococcus_s__Streptococcus_australis
PWY66_400__glycolysis_VI_(metazoan)_g__Streptococcus_s__Streptococcus_mitis_oralis_pneumo
PWY66_409__superpathway_of_purine_nucleotide_salvage_g__Citrobacter_s__Citrobacter_freun
PWY66_409__superpathway_of_purine_nucleotide_salvage_g__Streptococcus_s__Streptococcus_
PWY66_422__D_galactose_degradation_V_(Leloir_pathway)_g__Anaerostipes_s__Anaerostipes_h
PWY66_422__D_galactose_degradation_V_(Leloir_pathway)_g__Blautia_s__Blautia_sp_KLE_1732
PWY66_422__D_galactose_degradation_V_(Leloir_pathway)_g__Blautia_s__Ruminococcus_obeun
PWY66_422__D_galactose_degradation_V_(Leloir_pathway)_g__Citrobacter_s__Citrobacter_freur
PWY66_422__D_galactose_degradation_V_(Leloir_pathway)_g__Clostridium_s__Clostridium_sp_S
PWY66_422__D_galactose_degradation_V_(Leloir_pathway)_g__Coprococcus_s__Coprococcus_ca
PWY66_422__D_galactose_degradation_V_(Leloir_pathway)_g__Enterococcus_s__Enterococcus_c
PWY66_422__D_galactose_degradation_V_(Leloir_pathway)_g__Enterococcus_s__Enterococcus_f
PWY66_422__D_galactose_degradation_V_(Leloir_pathway)_g__Erysipelotrichaceae_noname_s__
PWY66_422__D_galactose_degradation_V_(Leloir_pathway)_g__Eubacterium_s__Eubacterium_si
PWY66_422__D_galactose_degradation_V_(Leloir_pathway)_g__Fusobacterium_s__Fusobacteriu
PWY66_422__D_galactose_degradation_V_(Leloir_pathway)_g__Lachnospiraceae_noname_s__La
PWY66_422__D_galactose_degradation_V_(Leloir_pathway)_g__Lachnospiraceae_noname_s__La
PWY66_422__D_galactose_degradation_V_(Leloir_pathway)_g__Lactobacillus_s__Lactobacillus_ca
PWY66_422__D_galactose_degradation_V_(Leloir_pathway)_g__Lactobacillus_s__Lactobacillus_rh
PWY66_422__D_galactose_degradation_V_(Leloir_pathway)_g__Lactococcus_s__Lactococcus_lact
PWY66_422__D_galactose_degradation_V_(Leloir_pathway)_g__Propionibacterium_s__Propionib
PWY66_422__D_galactose_degradation_V_(Leloir_pathway)_g__Ruminococcus_s__Ruminococcus
PWY66_422__D_galactose_degradation_V_(Leloir_pathway)_g__Streptococcus_s__Streptococcus_
PWY66_422__D_galactose_degradation_V_(Leloir_pathway)_g__Streptococcus_s__Streptococcus_
PYRIDNUCSAL_PWY__NAD_salvage_pathway_I_g__Citrobacter_s__Citrobacter_freundii
PYRIDNUCSYN_PWY__NAD_biosynthesis_I_(from_aspartate)_g__Alistipes_s__Alistipes_finegoldii
PYRIDNUCSYN_PWY__NAD_biosynthesis_I_(from_aspartate)_g__Alistipes_s__Alistipes_senegalen
PYRIDNUCSYN_PWY__NAD_biosynthesis_I_(from_aspartate)_g__Alistipes_s__Alistipes_shahii
PYRIDNUCSYN_PWY__NAD_biosynthesis_I_(from_aspartate)_g__Anaerostipes_s__Anaerostipes_h
PYRIDNUCSYN_PWY__NAD_biosynthesis_I_(from_aspartate)_g__Citrobacter_s__Citrobacter_freur
PYRIDNUCSYN_PWY__NAD_biosynthesis_I_(from_aspartate)_g__Clostridium_s__Clostridium_sp_S
PYRIDNUCSYN_PWY__NAD_biosynthesis_I_(from_aspartate)_g__Coprococcus_s__Coprococcus_ca
PYRIDNUCSYN_PWY__NAD_biosynthesis_I_(from_aspartate)_g__Coprococcus_s__Coprococcus_eu
PYRIDNUCSYN_PWY__NAD_biosynthesis_I_(from_aspartate)_g__Coprococcus_s__Coprococcus_sp
PYRIDNUCSYN_PWY__NAD_biosynthesis_I_(from_aspartate)_g__Eubacterium_s__Eubacterium_si
PYRIDNUCSYN_PWY__NAD_biosynthesis_I_(from_aspartate)_g__Lachnospiraceae_noname_s__La

PYRIDNUCSYN_PWY__NAD_biosynthesis_I_(from_aspartate)_g__Odoribacter_s__Odoribacter_spla
PYRIDNUCSYN_PWY__NAD_biosynthesis_I_(from_aspartate)_g__Oxalobacter_s__Oxalobacter_for
PYRIDNUCSYN_PWY__NAD_biosynthesis_I_(from_aspartate)_g__Propionibacterium_s__Propionib
PYRIDOXSYN_PWY__pyridoxal_5__phosphate_biosynthesis_I_g__Bacteroides_s__Bacteroides_egg
PYRIDOXSYN_PWY__pyridoxal_5__phosphate_biosynthesis_I_g__Bacteroides_s__Bacteroides_fae
PYRIDOXSYN_PWY__pyridoxal_5__phosphate_biosynthesis_I_g__Bacteroides_s__Bacteroides_frag
PYRIDOXSYN_PWY__pyridoxal_5__phosphate_biosynthesis_I_g__Bacteroides_s__Bacteroides_ova
PYRIDOXSYN_PWY__pyridoxal_5__phosphate_biosynthesis_I_g__Bacteroides_s__Bacteroides_sp_
PYRIDOXSYN_PWY__pyridoxal_5__phosphate_biosynthesis_I_g__Bacteroides_s__Bacteroides_sp_
PYRIDOXSYN_PWY__pyridoxal_5__phosphate_biosynthesis_I_g__Bacteroides_s__Bacteroides_sp_
PYRIDOXSYN_PWY__pyridoxal_5__phosphate_biosynthesis_I_g__Bacteroides_s__Bacteroides_sp_
PYRIDOXSYN_PWY__pyridoxal_5__phosphate_biosynthesis_I_g__Bacteroides_s__Bacteroides_sp_
PYRIDOXSYN_PWY__pyridoxal_5__phosphate_biosynthesis_I_g__Bacteroides_s__Bacteroides_sp_
PYRIDOXSYN_PWY__pyridoxal_5__phosphate_biosynthesis_I_g__Bacteroides_s__Bacteroides_ster
PYRIDOXSYN_PWY__pyridoxal_5__phosphate_biosynthesis_I_g__Bacteroides_s__Bacteroides_the
PYRIDOXSYN_PWY__pyridoxal_5__phosphate_biosynthesis_I_g__Bacteroides_s__Bacteroides_uni
PYRIDOXSYN_PWY__pyridoxal_5__phosphate_biosynthesis_I_g__Bacteroides_s__Bacteroides_xyla
PYRIDOXSYN_PWY__pyridoxal_5__phosphate_biosynthesis_I_g__Citrobacter_s__Citrobacter_freu
PYRIDOXSYN_PWY__pyridoxal_5__phosphate_biosynthesis_I_g__Parabacteroides_s__Parabacterc
REDCITCYC__TCA_cycle_VIII_(helicobacter)_g__Citrobacter_s__Citrobacter_freundii
REDCITCYC__TCA_cycle_VIII_(helicobacter)_unclassified
RHAMCAT_PWY__L_rhamnose_degradation_I_g__Bacteroides_s__Bacteroides_faecis
RHAMCAT_PWY__L_rhamnose_degradation_I_g__Bacteroides_s__Bacteroides_finegoldii
RHAMCAT_PWY__L_rhamnose_degradation_I_g__Bacteroides_s__Bacteroides_ovatus
RHAMCAT_PWY__L_rhamnose_degradation_I_g__Bacteroides_s__Bacteroides_sp_1_1_30
RHAMCAT_PWY__L_rhamnose_degradation_I_g__Bacteroides_s__Bacteroides_sp_1_1_6
RHAMCAT_PWY__L_rhamnose_degradation_I_g__Bacteroides_s__Bacteroides_sp_2_1_22
RHAMCAT_PWY__L_rhamnose_degradation_I_g__Bacteroides_s__Bacteroides_sp_2_2_4
RHAMCAT_PWY__L_rhamnose_degradation_I_g__Bacteroides_s__Bacteroides_sp_3_1_23
RHAMCAT_PWY__L_rhamnose_degradation_I_g__Bacteroides_s__Bacteroides_thetaiotaomicron
RHAMCAT_PWY__L_rhamnose_degradation_I_g__Bacteroides_s__Bacteroides_xylanisolvens
RHAMCAT_PWY__L_rhamnose_degradation_I_g__Citrobacter_s__Citrobacter_freundii
RHAMCAT_PWY__L_rhamnose_degradation_I_g__Clostridium_s__Clostridium_hathewayi
RHAMCAT_PWY__L_rhamnose_degradation_I_g__Enterococcus_s__Enterococcus_faecalis
RHAMCAT_PWY__L_rhamnose_degradation_I_g__Eubacterium_s__Eubacterium_ventriosum
RHAMCAT_PWY__L_rhamnose_degradation_I_g__Holdemania_s__Holdemania_filiformis
RHAMCAT_PWY__L_rhamnose_degradation_I_g__Klebsiella_s__Klebsiella_pneumoniae
RHAMCAT_PWY__L_rhamnose_degradation_I_g__Lachnospiraceae_noname_s__Lachnospiraceae_
RHAMCAT_PWY__L_rhamnose_degradation_I_g__Pediococcus_s__Pediococcus_acidilactici
RHAMCAT_PWY__L_rhamnose_degradation_I_g__Pediococcus_s__Pediococcus_lolii
RHAMCAT_PWY__L_rhamnose_degradation_I_g__Roseburia_s__Roseburia_intestinalis
RHAMCAT_PWY__L_rhamnose_degradation_I_g__Streptococcus_s__Streptococcus_parasanguinis
RIBOSYN2_PWY__flavin_biosynthesis_I_(bacteria_and_plants)_g__Propionibacterium_s__Propioni
RUMP_PWY__formaldehyde_oxidation_I
RUMP_PWY__formaldehyde_oxidation_I_unclassified
SER_GLYSYN_PWY__superpathway_of_L_serine_and_glycine_biosynthesis_I_g__Anaerostipes_s__
SER_GLYSYN_PWY__superpathway_of_L_serine_and_glycine_biosynthesis_I_g__Citrobacter_s__C

SER_GLYSYN_PWY__superpathway_of_L_serine_and_glycine_biosynthesis_I_g__Clostridium_s__C
SER_GLYSYN_PWY__superpathway_of_L_serine_and_glycine_biosynthesis_I_g__Coprococcus_s__
SER_GLYSYN_PWY__superpathway_of_L_serine_and_glycine_biosynthesis_I_g__Dorea_s__Dorea_
SER_GLYSYN_PWY__superpathway_of_L_serine_and_glycine_biosynthesis_I_g__Enterobacter_s__
SER_GLYSYN_PWY__superpathway_of_L_serine_and_glycine_biosynthesis_I_g__Eubacterium_s__
SER_GLYSYN_PWY__superpathway_of_L_serine_and_glycine_biosynthesis_I_g__Gordonibacter_s_
SER_GLYSYN_PWY__superpathway_of_L_serine_and_glycine_biosynthesis_I_g__Klebsiella_s__Kle
SER_GLYSYN_PWY__superpathway_of_L_serine_and_glycine_biosynthesis_I_g__Lachnospiraceae_
SER_GLYSYN_PWY__superpathway_of_L_serine_and_glycine_biosynthesis_I_g__Phascolarctobact
SER_GLYSYN_PWY__superpathway_of_L_serine_and_glycine_biosynthesis_I_g__Propionibacteriu
SER_GLYSYN_PWY__superpathway_of_L_serine_and_glycine_biosynthesis_I_g__Ruminococcus_s_
SER_GLYSYN_PWY__superpathway_of_L_serine_and_glycine_biosynthesis_I_g__Streptococcus_s_
SO4ASSIM_PWY__sulfate_reduction_I_(assimilatory)_g__Akkermansia_s__Akkermansia_muciniph
SO4ASSIM_PWY__sulfate_reduction_I_(assimilatory)_g__Citrobacter_s__Citrobacter_freundii
SO4ASSIM_PWY__sulfate_reduction_I_(assimilatory)_g__Enterobacter_s__Enterobacter_cloacae
SO4ASSIM_PWY__sulfate_reduction_I_(assimilatory)_g__Klebsiella_s__Klebsiella_pneumoniae
SO4ASSIM_PWY__sulfate_reduction_I_(assimilatory)_g__Odoribacter_s__Odoribacter_laneus
SO4ASSIM_PWY__sulfate_reduction_I_(assimilatory)_g__Odoribacter_s__Odoribacter_splanchnicu
SULFATE_CYS_PWY__superpathway_of_sulfate_assimilation_and_cysteine_biosynthesis_g__Citrol
SULFATE_CYS_PWY__superpathway_of_sulfate_assimilation_and_cysteine_biosynthesis_g__Enter
SULFATE_CYS_PWY__superpathway_of_sulfate_assimilation_and_cysteine_biosynthesis_g__Klebs
TCA_GLYOX_BYPASS__superpathway_of_glyoxylate_bypass_and_TCA_g__Citrobacter_s__Citrobac
TCA_GLYOX_BYPASS__superpathway_of_glyoxylate_bypass_and_TCA_g__Enterobacter_s__Enterc
TCA__TCA_cycle_I_(prokaryotic)_g__Citrobacter_s__Citrobacter_freundii
TCA__TCA_cycle_I_(prokaryotic)_g__Enterobacter_s__Enterobacter_cloacae
TEICHOICACID_PWY__teichoic_acid_(poly_glycerol)_biosynthesis_unclassified
THISYNARA_PWY__superpathway_of_thiamin_diphosphate_biosynthesis_III_(eukaryotes)_g__Ana
THISYNARA_PWY__superpathway_of_thiamin_diphosphate_biosynthesis_III_(eukaryotes)_g__Ana
THISYNARA_PWY__superpathway_of_thiamin_diphosphate_biosynthesis_III_(eukaryotes)_g__Ana
THISYNARA_PWY__superpathway_of_thiamin_diphosphate_biosynthesis_III_(eukaryotes)_g__Bac
THISYNARA_PWY__superpathway_of_thiamin_diphosphate_biosynthesis_III_(eukaryotes)_g__Bac
THISYNARA_PWY__superpathway_of_thiamin_diphosphate_biosynthesis_III_(eukaryotes)_g__Bac
THISYNARA_PWY__superpathway_of_thiamin_diphosphate_biosynthesis_III_(eukaryotes)_g__Bac
THISYNARA_PWY__superpathway_of_thiamin_diphosphate_biosynthesis_III_(eukaryotes)_g__Bac
THISYNARA_PWY__superpathway_of_thiamin_diphosphate_biosynthesis_III_(eukaryotes)_g__Bac
THISYNARA_PWY__superpathway_of_thiamin_diphosphate_biosynthesis_III_(eukaryotes)_g__Bac
THISYNARA_PWY__superpathway_of_thiamin_diphosphate_biosynthesis_III_(eukaryotes)_g__Blau
THISYNARA_PWY__superpathway_of_thiamin_diphosphate_biosynthesis_III_(eukaryotes)_g__Citr
THISYNARA_PWY__superpathway_of_thiamin_diphosphate_biosynthesis_III_(eukaryotes)_g__Clos
THISYNARA_PWY__superpathway_of_thiamin_diphosphate_biosynthesis_III_(eukaryotes)_g__Klel
THISYNARA_PWY__superpathway_of_thiamin_diphosphate_biosynthesis_III_(eukaryotes)_g__Lach
THISYNARA_PWY__superpathway_of_thiamin_diphosphate_biosynthesis_III_(eukaryotes)_g__Pha
THRESYN_PWY__superpathway_of_L_threonine_biosynthesis_g__Anaerostipes_s__Anaerostipes_
THRESYN_PWY__superpathway_of_L_threonine_biosynthesis_g__Anaerotruncus_s__Anaerotrunc
THRESYN_PWY__superpathway_of_L_threonine_biosynthesis_g__Bifidobacterium_s__Bifidobacte
THRESYN_PWY__superpathway_of_L_threonine_biosynthesis_g__Bifidobacterium_s__Bifidobacte
THRESYN_PWY__superpathway_of_L_threonine_biosynthesis_g__Bifidobacterium_s__Bifidobacte

THRESYN_PWY__superpathway_of_L_threonine_biosynthesis_g__Citrobacter_s__Citrobacter_freu
THRESYN_PWY__superpathway_of_L_threonine_biosynthesis_g__Clostridium_s__Clostridium_sp_
THRESYN_PWY__superpathway_of_L_threonine_biosynthesis_g__Collinsella_s__Collinsella_intest
THRESYN_PWY__superpathway_of_L_threonine_biosynthesis_g__Coprococcus_s__Coprococcus_c
THRESYN_PWY__superpathway_of_L_threonine_biosynthesis_g__Coprococcus_s__Coprococcus_e
THRESYN_PWY__superpathway_of_L_threonine_biosynthesis_g__Coprococcus_s__Coprococcus_s
THRESYN_PWY__superpathway_of_L_threonine_biosynthesis_g__Enterobacter_s__Enterobacter_
THRESYN_PWY__superpathway_of_L_threonine_biosynthesis_g__Erysipelotrichaceae_noname_s_
THRESYN_PWY__superpathway_of_L_threonine_biosynthesis_g__Holdemania_s__Holdemania_fil
THRESYN_PWY__superpathway_of_L_threonine_biosynthesis_g__Klebsiella_s__Klebsiella_pneum
THRESYN_PWY__superpathway_of_L_threonine_biosynthesis_g__Lachnospiraceae_noname_s__La
THRESYN_PWY__superpathway_of_L_threonine_biosynthesis_g__Lactococcus_s__Lactococcus_lac
THRESYN_PWY__superpathway_of_L_threonine_biosynthesis_g__Megasphaera_s__Megasphaera_
THRESYN_PWY__superpathway_of_L_threonine_biosynthesis_g__Oxalobacter_s__Oxalobacter_fo
THRESYN_PWY__superpathway_of_L_threonine_biosynthesis_g__Phascolarctobacterium_s__Phas
THRESYN_PWY__superpathway_of_L_threonine_biosynthesis_g__Propionibacterium_s__Propionil
THRESYN_PWY__superpathway_of_L_threonine_biosynthesis_g__Rothia_s__Rothia_mucilaginosa
THRESYN_PWY__superpathway_of_L_threonine_biosynthesis_g__Streptococcus_s__Streptococcus
THRESYN_PWY__superpathway_of_L_threonine_biosynthesis_g__Veillonella_s__Veillonella_dispa
THRESYN_PWY__superpathway_of_L_threonine_biosynthesis_g__Veillonella_s__Veillonella_parvu
TRNA_CHARGING_PWY__tRNA_charging_g__Fusobacterium_s__Fusobacterium_nucleatum
TRPSYN_PWY__L_tryptophan_biosynthesis_g__Anaerostipes_s__Anaerostipes_hadrus
TRPSYN_PWY__L_tryptophan_biosynthesis_g__Bacteroides_s__Bacteroides_fragilis
TRPSYN_PWY__L_tryptophan_biosynthesis_g__Bacteroides_s__Bacteroides_xylanisolvens
TRPSYN_PWY__L_tryptophan_biosynthesis_g__Blautia_s__Blautia_sp_KLE_1732
TRPSYN_PWY__L_tryptophan_biosynthesis_g__Blautia_s__Ruminococcus_obeum
TRPSYN_PWY__L_tryptophan_biosynthesis_g__Citrobacter_s__Citrobacter_freundii
TRPSYN_PWY__L_tryptophan_biosynthesis_g__Clostridium_s__Clostridium_sp_SS2_1
TRPSYN_PWY__L_tryptophan_biosynthesis_g__Coprococcus_s__Coprococcus_eutactus
TRPSYN_PWY__L_tryptophan_biosynthesis_g__Coprococcus_s__Coprococcus_sp_ART55_1
TRPSYN_PWY__L_tryptophan_biosynthesis_g__Lactococcus_s__Lactococcus_lactis
TRPSYN_PWY__L_tryptophan_biosynthesis_g__Odoribacter_s__Odoribacter_splanchnicus
TRPSYN_PWY__L_tryptophan_biosynthesis_g__Oxalobacter_s__Oxalobacter_formigenes
TRPSYN_PWY__L_tryptophan_biosynthesis_g__Phascolarctobacterium_s__Phascolarctobacterium_
TRPSYN_PWY__L_tryptophan_biosynthesis_g__Ruminococcus_s__Ruminococcus_champanellensis
TRPSYN_PWY__L_tryptophan_biosynthesis_g__Ruminococcus_s__Ruminococcus_sp
UBISYN_PWY__superpathway_of_ubiquinol_8_biosynthesis_(prokaryotic)_g__Klebsiella_s__Klebsi
UBISYN_PWY__superpathway_of_ubiquinol_8_biosynthesis_(prokaryotic)_unclassified
UDPNAGSYN_PWY__UDP_N_acetyl_D_glucosamine_biosynthesis_I_g__Bifidobacterium_s__Bifido
UDPNAGSYN_PWY__UDP_N_acetyl_D_glucosamine_biosynthesis_I_g__Bifidobacterium_s__Bifido
UDPNAGSYN_PWY__UDP_N_acetyl_D_glucosamine_biosynthesis_I_g__Bifidobacterium_s__Bifido
UDPNAGSYN_PWY__UDP_N_acetyl_D_glucosamine_biosynthesis_I_g__Bifidobacterium_s__Bifido
UDPNAGSYN_PWY__UDP_N_acetyl_D_glucosamine_biosynthesis_I_g__Clostridium_s__Clostridiur
UDPNAGSYN_PWY__UDP_N_acetyl_D_glucosamine_biosynthesis_I_g__Coprococcus_s__Coprococ
UDPNAGSYN_PWY__UDP_N_acetyl_D_glucosamine_biosynthesis_I_g__Enterobacter_s__Enteroba
UDPNAGSYN_PWY__UDP_N_acetyl_D_glucosamine_biosynthesis_I_g__Enterococcus_s__Enterocc
UDPNAGSYN_PWY__UDP_N_acetyl_D_glucosamine_biosynthesis_I_g__Enterococcus_s__Enterocc

UDPNAGSYN_PWY__UDP_N_acetyl_D_glucosamine_biosynthesis_I_g__Fusobacterium_s__Fusoba
UDPNAGSYN_PWY__UDP_N_acetyl_D_glucosamine_biosynthesis_I_g__Klebsiella_s__Klebsiella_p
UDPNAGSYN_PWY__UDP_N_acetyl_D_glucosamine_biosynthesis_I_g__Lactobacillus_s__Lactobac
UDPNAGSYN_PWY__UDP_N_acetyl_D_glucosamine_biosynthesis_I_g__Lactococcus_s__Lactococc
UDPNAGSYN_PWY__UDP_N_acetyl_D_glucosamine_biosynthesis_I_g__Ruminococcus_s__Rumino
UDPNAGSYN_PWY__UDP_N_acetyl_D_glucosamine_biosynthesis_I_g__Streptococcus_s__Strepto
UDPNAGSYN_PWY__UDP_N_acetyl_D_glucosamine_biosynthesis_I_g__Streptococcus_s__Strepto
URSIN_PWY__ureide_biosynthesis
URSIN_PWY__ureide_biosynthesis_unclassified
VALSYN_PWY__L_valine_biosynthesis_g__Akkermansia_s__Akkermansia_muciniphila
VALSYN_PWY__L_valine_biosynthesis_g__Alistipes_s__Alistipes_indistinctus
VALSYN_PWY__L_valine_biosynthesis_g__Alistipes_s__Alistipes_senegalensis
VALSYN_PWY__L_valine_biosynthesis_g__Anaerofustis_s__Anaerofustis_stercorihominis
VALSYN_PWY__L_valine_biosynthesis_g__Anaerostipes_s__Anaerostipes_hadrus
VALSYN_PWY__L_valine_biosynthesis_g__Bacteroides_s__Bacteroides_cellulosilyticus
VALSYN_PWY__L_valine_biosynthesis_g__Bacteroides_s__Bacteroides_clarus
VALSYN_PWY__L_valine_biosynthesis_g__Bacteroides_s__Bacteroides_finegoldii
VALSYN_PWY__L_valine_biosynthesis_g__Bacteroides_s__Bacteroides_intestinalis
VALSYN_PWY__L_valine_biosynthesis_g__Bacteroides_s__Bacteroides_massiliensis
VALSYN_PWY__L_valine_biosynthesis_g__Bacteroides_s__Bacteroides_plebeius
VALSYN_PWY__L_valine_biosynthesis_g__Bacteroides_s__Bacteroides_salyersiae
VALSYN_PWY__L_valine_biosynthesis_g__Bacteroides_s__Bacteroides_sp_1_1_6
VALSYN_PWY__L_valine_biosynthesis_g__Bacteroides_s__Bacteroides_sp_2_2_4
VALSYN_PWY__L_valine_biosynthesis_g__Bacteroides_s__Bacteroides_sp_3_1_19
VALSYN_PWY__L_valine_biosynthesis_g__Bacteroides_s__Bacteroides_sp_3_1_23
VALSYN_PWY__L_valine_biosynthesis_g__Bacteroides_s__Bacteroides_stercoris
VALSYN_PWY__L_valine_biosynthesis_g__Bacteroides_s__Bacteroides_xylanisolvens
VALSYN_PWY__L_valine_biosynthesis_g__Barnesiella_s__Barnesiella_intestinihominis
VALSYN_PWY__L_valine_biosynthesis_g__Bifidobacterium_s__Bifidobacterium_angulatum
VALSYN_PWY__L_valine_biosynthesis_g__Bifidobacterium_s__Bifidobacterium_catenulatum
VALSYN_PWY__L_valine_biosynthesis_g__Bifidobacterium_s__Bifidobacterium_pseudocatenulatu
VALSYN_PWY__L_valine_biosynthesis_g__Bilophila_s__Bilophila_wadsworthia
VALSYN_PWY__L_valine_biosynthesis_g__Blautia_s__Blautia_hydrogenotrophica
VALSYN_PWY__L_valine_biosynthesis_g__Blautia_s__Blautia_sp_KLE_1732
VALSYN_PWY__L_valine_biosynthesis_g__Blautia_s__Ruminococcus_obeum
VALSYN_PWY__L_valine_biosynthesis_g__Burkholderiales_noname_s__Burkholderiales_bacterium
VALSYN_PWY__L_valine_biosynthesis_g__Butyrivibrio_s__Butyrivibrio_crossotus
VALSYN_PWY__L_valine_biosynthesis_g__Catenibacterium_s__Catenibacterium_mitsuokai
VALSYN_PWY__L_valine_biosynthesis_g__Citrobacter_s__Citrobacter_freundii
VALSYN_PWY__L_valine_biosynthesis_g__Clostridiales_noname_s__Bacteroides_pectinophilus
VALSYN_PWY__L_valine_biosynthesis_g__Clostridiales_noname_s__Clostridiales_bacterium_1_7_
VALSYN_PWY__L_valine_biosynthesis_g__Clostridium_s__Clostridium_asparagiforme
VALSYN_PWY__L_valine_biosynthesis_g__Clostridium_s__Clostridium_bolteae
VALSYN_PWY__L_valine_biosynthesis_g__Clostridium_s__Clostridium_citroniae
VALSYN_PWY__L_valine_biosynthesis_g__Clostridium_s__Clostridium_clostridioforme
VALSYN_PWY__L_valine_biosynthesis_g__Clostridium_s__Clostridium_hathewayi
VALSYN_PWY__L_valine_biosynthesis_g__Clostridium_s__Clostridium_leptum

VALSYN_PWY__L_valine_biosynthesis_g__Clostridium_s__Clostridium_sp_L2_50
VALSYN_PWY__L_valine_biosynthesis_g__Clostridium_s__Clostridium_sp_SS2_1
VALSYN_PWY__L_valine_biosynthesis_g__Collinsella_s__Collinsella_tanakaei
VALSYN_PWY__L_valine_biosynthesis_g__Coprobacter_s__Coprobacter_fastidiosus
VALSYN_PWY__L_valine_biosynthesis_g__Coprococcus_s__Coprococcus_catus
VALSYN_PWY__L_valine_biosynthesis_g__Coprococcus_s__Coprococcus_eutactus
VALSYN_PWY__L_valine_biosynthesis_g__Coprococcus_s__Coprococcus_sp_ART55_1
VALSYN_PWY__L_valine_biosynthesis_g__Dorea_s__Dorea_formicigenerans
VALSYN_PWY__L_valine_biosynthesis_g__Eggerthella_s__Eggerthella_sp_1_3_56FAA
VALSYN_PWY__L_valine_biosynthesis_g__Eggerthella_s__Eggerthella_sp_HGA1
VALSYN_PWY__L_valine_biosynthesis_g__Enterobacter_s__Enterobacter_cloacae
VALSYN_PWY__L_valine_biosynthesis_g__Enterorhabdus_s__Enterorhabdus_caecimuris
VALSYN_PWY__L_valine_biosynthesis_g__Erysipelotrichaceae_noname_s__Clostridium_spiroform
VALSYN_PWY__L_valine_biosynthesis_g__Erysipelotrichaceae_noname_s__Erysipelotrichaceae_ba
VALSYN_PWY__L_valine_biosynthesis_g__Erysipelotrichaceae_noname_s__Erysipelotrichaceae_ba
VALSYN_PWY__L_valine_biosynthesis_g__Erysipelotrichaceae_noname_s__Eubacterium_cylindroi
VALSYN_PWY__L_valine_biosynthesis_g__Eubacterium_s__Eubacterium_eligens
VALSYN_PWY__L_valine_biosynthesis_g__Eubacterium_s__Eubacterium_hallii
VALSYN_PWY__L_valine_biosynthesis_g__Eubacterium_s__Eubacterium_ramulus
VALSYN_PWY__L_valine_biosynthesis_g__Eubacterium_s__Eubacterium_siraeum
VALSYN_PWY__L_valine_biosynthesis_g__Eubacterium_s__Eubacterium_sp_3_1_31
VALSYN_PWY__L_valine_biosynthesis_g__Fusobacterium_s__Fusobacterium_nucleatum
VALSYN_PWY__L_valine_biosynthesis_g__Klebsiella_s__Klebsiella_pneumoniae
VALSYN_PWY__L_valine_biosynthesis_g__Lachnospiraceae_noname_s__Lachnospiraceae_bacteriu
VALSYN_PWY__L_valine_biosynthesis_g__Lachnospiraceae_noname_s__Lachnospiraceae_bacteriu
VALSYN_PWY__L_valine_biosynthesis_g__Lachnospiraceae_noname_s__Lachnospiraceae_bacteriu
VALSYN_PWY__L_valine_biosynthesis_g__Lachnospiraceae_noname_s__Lachnospiraceae_bacteriu
VALSYN_PWY__L_valine_biosynthesis_g__Lachnospiraceae_noname_s__Lachnospiraceae_bacteriu
VALSYN_PWY__L_valine_biosynthesis_g__Lachnospiraceae_noname_s__Lachnospiraceae_bacteriu
VALSYN_PWY__L_valine_biosynthesis_g__Odoribacter_s__Odoribacter_laneus
VALSYN_PWY__L_valine_biosynthesis_g__Odoribacter_s__Odoribacter_splanchnicus
VALSYN_PWY__L_valine_biosynthesis_g__Oxalobacter_s__Oxalobacter_formigenes
VALSYN_PWY__L_valine_biosynthesis_g__Parabacteroides_s__Parabacteroides_distasonis
VALSYN_PWY__L_valine_biosynthesis_g__Parabacteroides_s__Parabacteroides_goldsteinii
VALSYN_PWY__L_valine_biosynthesis_g__Parabacteroides_s__Parabacteroides_johnsonii
VALSYN_PWY__L_valine_biosynthesis_g__Parabacteroides_s__Parabacteroides_merdae
VALSYN_PWY__L_valine_biosynthesis_g__Parabacteroides_s__Parabacteroides_sp_20_3
VALSYN_PWY__L_valine_biosynthesis_g__Parabacteroides_s__Parabacteroides_sp_D13
VALSYN_PWY__L_valine_biosynthesis_g__Paraprevotella_s__Paraprevotella_clara
VALSYN_PWY__L_valine_biosynthesis_g__Paraprevotella_s__Paraprevotella_xylaniphila
VALSYN_PWY__L_valine_biosynthesis_g__Parasutterella_s__Parasutterella_excrementihominis
VALSYN_PWY__L_valine_biosynthesis_g__Peptostreptococcaceae_noname_s__Clostridium_bartle
VALSYN_PWY__L_valine_biosynthesis_g__Phascolarctobacterium_s__Phascolarctobacterium_succ
VALSYN_PWY__L_valine_biosynthesis_g__Propionibacterium_s__Propionibacterium_freudenreich
VALSYN_PWY__L_valine_biosynthesis_g__Roseburia_s__Roseburia_hominis
VALSYN_PWY__L_valine_biosynthesis_g__Roseburia_s__Roseburia_inulinivorans
VALSYN_PWY__L_valine_biosynthesis_g__Ruminococcaceae_noname_s__Ruminococcaceae_bacte

VALSYN_PWY__L_valine_biosynthesis_g__Ruminococcus_s__Ruminococcus_sp
VALSYN_PWY__L_valine_biosynthesis_g__Ruminococcus_s__Ruminococcus_sp_JC304
VALSYN_PWY__L_valine_biosynthesis_g__Streptococcus_s__Streptococcus_australis
VALSYN_PWY__L_valine_biosynthesis_g__Streptococcus_s__Streptococcus_mitis_oralis_pneumon
VALSYN_PWY__L_valine_biosynthesis_g__Streptococcus_s__Streptococcus_sanguinis
VALSYN_PWY__L_valine_biosynthesis_g__Subdoligranulum_s__Subdoligranulum_sp_4_3_54A2FA
VALSYN_PWY__L_valine_biosynthesis_g__Veillonella_s__Veillonella_dispar
VALSYN_PWY__L_valine_biosynthesis_g__Veillonella_s__Veillonella_parvula
PWY_6151__S_adenosyl_L_methionine_cycle_I_g__Haemophilus_s__Haemophilus_parainfluenzae
PWY_6387__UDP_N_acetylmuramoyl_pentapeptide_biosynthesis_I_(meso_diaminopimelate_con
PWY_6703__preQ0_biosynthesis_g__Bacteroides_s__Bacteroides_caccae
PWY_1269__CMP_3_deoxy_D_manno_octulosonate_biosynthesis_I
PWY0_1061__superpathway_of_L_alanine_biosynthesis_g__Haemophilus_s__Haemophilus_parai
PWY_1042__glycolysis_IV_(plant_cytosol)_g__Lactococcus_s__Lactococcus_lactis
PWY0_162__superpathway_of_pyrimidine_ribonucleotides_de_novo_biosynthesis
PWY66_400__glycolysis_VI_(metazoan)_unclassified
PWY_6121__5_aminoimidazole_ribonucleotide_biosynthesis_I
PWY_6396__superpathway_of_2,3_butanediol_biosynthesis
PWY_6147__6_hydroxymethyl_dihydropterin_diphosphate_biosynthesis_I_unclassified
1CMET2_PWY__N10_formyl_tetrahydrofolate_biosynthesis_g__Bacteroides_s__Bacteroides_cacca
FAO_PWY__fatty_acid_&beta__oxidation_I_unclassified
DTDPRHAMSYN_PWY__dTDP_L_rhamnose_biosynthesis_I_g__Veillonella_s__Veillonella_atypica
PWY_7013__L_1,2_propanediol_degradation_g__Escherichia_s__Escherichia_coli
THRESYN_PWY__superpathway_of_L_threonine_biosynthesis
PWY_5136__fatty_acid_&beta__oxidation_II_(peroxisome)_unclassified
P122_PWY__heterolactic_fermentation_unclassified
PWY_6122__5_aminoimidazole_ribonucleotide_biosynthesis_II_g__Lactococcus_s__Lactococcus_l
PWY_6277__superpathway_of_5_aminoimidazole_ribonucleotide_biosynthesis_g__Lactococcus_s
PWY_6545__pyrimidine_deoxyribonucleotides_de_novo_biosynthesis_III
ILEUSYN_PWY__L_isoleucine_biosynthesis_I_(from_threonine)_g__Lactococcus_s__Lactococcus_la
VALSYN_PWY__L_valine_biosynthesis_g__Lactococcus_s__Lactococcus_lactis
PWY0_845__superpathway_of_pyridoxal_5__phosphate_biosynthesis_and_salvage_g__Bacteroide
PWY_5686__UMP_biosynthesis_g__Bifidobacterium_s__Bifidobacterium_longum
PWY_7539__6_hydroxymethyl_dihydropterin_diphosphate_biosynthesis_III_(Chlamydia)_unclassi
PWY0_1586__peptidoglycan_maturation_(meso_diaminopimelate_containing)
PEPTIDOGLYCANSYN_PWY__peptidoglycan_biosynthesis_I_(meso_diaminopimelate_containing)_g
PWY_3841__folate_transformations_II_g__Bacteroides_s__Bacteroides_caccae
PWY_2941__L_lysine_biosynthesis_II_unclassified
PWY_6386__UDP_N_acetylmuramoyl_pentapeptide_biosynthesis_II_(lysine_containing)_g__Haem
FUC_RHAMCAT_PWY__superpathway_of_fucose_and_rhamnose_degradation_unclassified
PYRIDOXSYN_PWY__pyridoxal_5__phosphate_biosynthesis_I_g__Bacteroides_s__Bacteroides_cac
PWY_7187__pyrimidine_deoxyribonucleotides_de_novo_biosynthesis_II
PWY490_3__nitrate_reduction_VI_(assimilatory)_unclassified
HEMESYN2_PWY__heme_biosynthesis_II_(anaerobic)_g__Haemophilus_s__Haemophilus_parainfl
PWY_7197__pyrimidine_deoxyribonucleotide_phosphorylation_g__Haemophilus_s__Haemophilus
SALVADEHYPOX_PWY__adenosine_nucleotides_degradation_II
ARG+POLYAMINE_SYN__superpathway_of_arginine_and_polyamine_biosynthesis

PWY_6113__superpathway_of_mycolate_biosynthesis_unclassified
UNINTEGRATED_g__Bacteroides_s__Bacteroides_caccae
PWY_5989__stearate_biosynthesis_II_(bacteria_and_plants)_unclassified
PWY_6147__6_hydroxymethyl_dihydropterin_diphosphate_biosynthesis_I_g__Lactococcus_s__Lac
PWY_7219__adenosine_ribonucleotides_de_novo_biosynthesis_g__Lactococcus_s__Lactococcus_l
PWY_6737__starch_degradation_V_g__Haemophilus_s__Haemophilus_parainfluenzae
PWY_6284__superpathway_of_unsaturated_fatty_acids_biosynthesis_(E__coli)_unclassified
THISYNARA_PWY__superpathway_of_thiamin_diphosphate_biosynthesis_III_(eukaryotes)
PWY_7221__guanosine_ribonucleotides_de_novo_biosynthesis_g__Haemophilus_s__Haemophilu
GLCMANNANAUT_PWY__superpathway_of_N_acetylglucosamine,_N_acetylmannosamine_and_N
PWY_6897__thiamin_salvage_II
PWY_5188__tetrapyrrole_biosynthesis_I_(from_glutamate)
PWY0_1261__anhydromuropeptides_recycling_unclassified
PWY_5367__petroselinate_biosynthesis_unclassified
PWY_7211__superpathway_of_pyrimidine_deoxyribonucleotides_de_novo_biosynthesis
PWY0_1061__superpathway_of_L_alanine_biosynthesis_unclassified
PWY_6121__5_aminoimidazole_ribonucleotide_biosynthesis_I_g__Actinomyces_s__Actinomyces_
THISYN_PWY__superpathway_of_thiamin_diphosphate_biosynthesis_I_unclassified
GLCMANNANAUT_PWY__superpathway_of_N_acetylglucosamine,_N_acetylmannosamine_and_N
PWY_6125__superpathway_of_guanosine_nucleotides_de_novo_biosynthesis_II_g__Haemophilus
UNINTEGRATED_g__Actinomyces_s__Actinomyces_graevenitzii
PWY_6122__5_aminoimidazole_ribonucleotide_biosynthesis_II_g__Actinomyces_s__Actinomyces_
PWY_6277__superpathway_of_5_aminoimidazole_ribonucleotide_biosynthesis_g__Actinomyces_s
HISTSYN_PWY__L_histidine_biosynthesis
PWY_6317__galactose_degradation_I_(Leloir_pathway)
PWY_7228__superpathway_of_guanosine_nucleotides_de_novo_biosynthesis_I_g__Haemophilus_
POLYISOPRENSYN_PWY__polyisoprenoid_biosynthesis_(E__coli)_unclassified
PENTOSE_P_PWY__pentose_phosphate_pathway_unclassified
PWY_6901__superpathway_of_glucose_and_xylose_degradation_unclassified
PWY_3001__superpathway_of_L_isoleucine_biosynthesis_I
COA_PWY_1__coenzyme_A_biosynthesis_II_(mammalian)_g__Haemophilus_s__Haemophilus_par
GLUTORN_PWY__L_ornithine_biosynthesis_g__Lactococcus_s__Lactococcus_lactis
PWY_6936__seleno_amino_acid_biosynthesis_g__Haemophilus_s__Haemophilus_parainfluenzae
PWY_7220__adenosine_deoxyribonucleotides_de_novo_biosynthesis_II_g__Haemophilus_s__Hae
PWY_7222__guanosine_deoxyribonucleotides_de_novo_biosynthesis_II_g__Haemophilus_s__Hae
PWY_5840__superpathway_of_menaquinol_7_biosynthesis_unclassified
PWY_7199__pyrimidine_deoxyribonucleosides_salvage_g__Haemophilus_s__Haemophilus_parain
PWY_6519__8_amino_7_oxononanoate_biosynthesis_I_unclassified
PWY_6609__adenine_and_adenosine_salvage_III
PWY_6165__chorismate_biosynthesis_II_(archaea)
PWY_4981__L_proline_biosynthesis_II_(from_arginine)_unclassified
PANTO_PWY__phosphopantothenate_biosynthesis_I_g__Actinomyces_s__Actinomyces_graevenit
METHGLYUT_PWY__superpathway_of_methylglyoxal_degradation_unclassified
PWY_6859__all_trans_farnesol_biosynthesis_unclassified
COBALSYN_PWY__adenosylcobalamin_salvage_from_cobinamide_I
GLCMANNANAUT_PWY__superpathway_of_N_acetylglucosamine,_N_acetylmannosamine_and_N
PEPTIDOGLYCANSYN_PWY__peptidoglycan_biosynthesis_I_(meso_diaminopimelate_containing)_g

PWY_6387__UDP_N_acetylmuramoyl_pentapeptide_biosynthesis_I_(meso_diaminopimelate_con
PWY_5103__L_isoleucine_biosynthesis_III
PWY_7111__pyruvate_fermentation_to_isobutanol_(engineered)
FOLSYN_PWY__superpathway_of_tetrahydrofolate_biosynthesis_and_salvage_unclassified
COA_PWY_1__coenzyme_A_biosynthesis_II_(mammalian)_g__Lactococcus_s__Lactococcus_lactis
PWY_6151__S_adenosyl_L_methionine_cycle_I_g__Actinomyces_s__Actinomyces_graevenitzii
PWY_5695__urate_biosynthesis_inosine_5__phosphate_degradation_g__Haemophilus_s__Haemo
P4_PWY__superpathway_of_L_lysine,_L_threonine_and_L_methionine_biosynthesis_I
BIOTIN_BIOSYNTHESIS_PWY__biotin_biosynthesis_I_unclassified
SER_GLYSYN_PWY__superpathway_of_L_serine_and_glycine_biosynthesis_I_g__Lactococcus_s__L
BRANCHED_CHAIN_AA_SYN_PWY__superpathway_of_branched_amino_acid_biosynthesis
ARO_PWY__chorismate_biosynthesis_I_g__Haemophilus_s__Haemophilus_parainfluenzae
DAPLYSINESYN_PWY__L_lysine_biosynthesis_I_unclassified
P185_PWY__formaldehyde_assimilation_III_(dihydroxyacetone_cycle)_unclassified
PWY_6612__superpathway_of_tetrahydrofolate_biosynthesis_unclassified
COMPLETE_ARO_PWY__superpathway_of_aromatic_amino_acid_biosynthesis_g__Haemophilus_s
PWY_5667__CDP_diacylglycerol_biosynthesis_I_g__Haemophilus_s__Haemophilus_parainfluenza
PWY0_1319__CDP_diacylglycerol_biosynthesis_II_g__Haemophilus_s__Haemophilus_parainfluenz
THISYNARA_PWY__superpathway_of_thiamin_diphosphate_biosynthesis_III_(eukaryotes)_unclass
PWY_6151__S_adenosyl_L_methionine_cycle_I_g__Lactococcus_s__Lactococcus_lactis
PWY_6386__UDP_N_acetylmuramoyl_pentapeptide_biosynthesis_II_(lysine_containing)_g__Actin
PWY_7208__superpathway_of_pyrimidine_nucleobases_salvage_g__Haemophilus_s__Haemophil
PWY_1042__glycolysis_IV_(plant_cytosol)_g__Haemophilus_s__Haemophilus_parainfluenzae
RIBOSYN2_PWY__flavin_biosynthesis_I_(bacteria_and_plants)_unclassified
COA_PWY__coenzyme_A_biosynthesis_I_g__Lactococcus_s__Lactococcus_lactis
P124_PWY__Bifidobacterium_shunt
PWY_7391__isoprene_biosynthesis_II_(engineered)_unclassified
PWY_2942__L_lysine_biosynthesis_III_g__Haemophilus_s__Haemophilus_parainfluenzae
PWY_5695__urate_biosynthesis_inosine_5__phosphate_degradation
PWY_5097__L_lysine_biosynthesis_VI_g__Haemophilus_s__Haemophilus_parainfluenzae
PWY_1269__CMP_3_deoxy_D_manno_octulosonate_biosynthesis_I_g__Haemophilus_s__Haemop
PWY_6163__chorismate_biosynthesis_from_3_dehydroquinate_g__Haemophilus_s__Haemophilu
PWY_7184__pyrimidine_deoxyribonucleotides_de_novo_biosynthesis_I_g__Haemophilus_s__Hae
P163_PWY__L_lysine_fermentation_to_acetate_and_butanoate_unclassified
FUCCAT_PWY__fucose_degradation_unclassified
PWY_561__superpathway_of_glyoxylate_cycle_and_fatty_acid_degradation_unclassified
SER_GLYSYN_PWY__superpathway_of_L_serine_and_glycine_biosynthesis_I_g__Haemophilus_s__
PWY66_398__TCA_cycle_III_(animals)_unclassified
PWY_841__superpathway_of_purine_nucleotides_de_novo_biosynthesis_I_g__Haemophilus_s__H
PWY_6703__preQ0_biosynthesis
ILEUSYN_PWY__L_isoleucine_biosynthesis_I_(from_threonine)
VALSYN_PWY__L_valine_biosynthesis
PWY_6126__superpathway_of_adenosine_nucleotides_de_novo_biosynthesis_II_g__Haemophilus
PWY_5667__CDP_diacylglycerol_biosynthesis_I_g__Actinomyces_s__Actinomyces_odontolyticus
PWY0_1319__CDP_diacylglycerol_biosynthesis_II_g__Actinomyces_s__Actinomyces_odontolyticus
PRPP_PWY__superpathway_of_histidine,_purine,_and_pyrimidine_biosynthesis
PWY0_862__(5Z)_dodec_5_enoate_biosynthesis_g__Haemophilus_s__Haemophilus_parainfluenza

PWY_6700__queuosine_biosynthesis_g__Haemophilus_s__Haemophilus_parainfluenzae
PWY_7229__superpathway_of_adenosine_nucleotides_de_novo_biosynthesis_I_g__Haemophilus_
PWY_5188__tetrapyrrole_biosynthesis_I_(from_glutamate)_g__Haemophilus_s__Haemophilus_pa
PWY_7234__inosine_5__phosphate_biosynthesis_III_g__Haemophilus_s__Haemophilus_parainflu
PWY66_400__glycolysis_VI_(metazoan)_g__Haemophilus_s__Haemophilus_parainfluenzae
DTDPRHAMSYN_PWY__dTDP_L_rhamnose_biosynthesis_I_g__Lactococcus_s__Lactococcus_lactis
PWY_724__superpathway_of_L_lysine,_L_threonine_and_L_methionine_biosynthesis_II_g__Haem
PWY0_1296__purine_ribonucleosides_degradation_g__Dorea_s__Dorea_longicatena
PWY_6700__queuosine_biosynthesis_g__Bacteroides_s__Bacteroides_sp_3_2_5
VALSYN_PWY__L_valine_biosynthesis_g__Haemophilus_s__Haemophilus_parainfluenzae
PWY_6630__superpathway_of_L_tyrosine_biosynthesis_unclassified
PWY_6471__peptidoglycan_biosynthesis_IV_(Enterococcus_faecium)
PWY_3001__superpathway_of_L_isoleucine_biosynthesis_I_g__Haemophilus_s__Haemophilus_pa
ANAGLYCOLYSIS_PWY__glycolysis_III_(from_glucose)_g__Haemophilus_s__Haemophilus_parainflu
PWY_6121__5_aminoimidazole_ribonucleotide_biosynthesis_I_g__Haemophilus_s__Haemophilus
PWY_7219__adenosine_ribonucleotides_de_novo_biosynthesis_g__Haemophilus_s__Haemophilu
PWY_6168__flavin_biosynthesis_III_(fungi)
THISYNARA_PWY__superpathway_of_thiamin_diphosphate_biosynthesis_III_(eukaryotes)_g__Blau
HISTSYN_PWY__L_histidine_biosynthesis_g__Streptococcus_s__Streptococcus_thermophilus
P42_PWY__incomplete_reductive_TCA_cycle
PWY_6527__stachyose_degradation
HOMOSER_METSYN_PWY__L_methionine_biosynthesis_I_g__Blautia_s__Ruminococcus_torques
ASPASN_PWY__superpathway_of_L_aspartate_and_L_asparagine_biosynthesis_g__Haemophilus_
PWY_621__sucrose_degradation_III_(sucrose_invertase)_g__Blautia_s__Ruminococcus_torques
THRESYN_PWY__superpathway_of_L_threonine_biosynthesis_g__Haemophilus_s__Haemophilus_
PWY_7392__taxadiene_biosynthesis_(engineered)_unclassified
PWY_6122__5_aminoimidazole_ribonucleotide_biosynthesis_II_g__Haemophilus_s__Haemophilus
PWY_6277__superpathway_of_5_aminoimidazole_ribonucleotide_biosynthesis_g__Haemophilus_
PANTOSYN_PWY__pantothenate_and_coenzyme_A_biosynthesis_I
PWY_7219__adenosine_ribonucleotides_de_novo_biosynthesis_g__Dorea_s__Dorea_longicatena
PWY_7234__inosine_5__phosphate_biosynthesis_III_unclassified
PWY_6123__inosine_5__phosphate_biosynthesis_I_g__Haemophilus_s__Haemophilus_parainflue
UNMAPPED
PWY_5686__UMP_biosynthesis_g__Dorea_s__Dorea_longicatena
COA_PWY_1__coenzyme_A_biosynthesis_II_(mammalian)_g__Actinomyces_s__Actinomyces_odo
PWY_5367__petroselinate_biosynthesis
PWY_5100__pyruvate_fermentation_to_acetate_and_lactate_II_g__Blautia_s__Ruminococcus_tor
PWY_5347__superpathway_of_L_methionine_biosynthesis_(transsulfuration)
FASYN_INITIAL_PWY__superpathway_of_fatty_acid_biosynthesis_initiation_(E__coli)_g__Haemop
PWY_2942__L_lysine_biosynthesis_III_g__Bacteroides_s__Bacteroides_sp_3_2_5
THRESYN_PWY__superpathway_of_L_threonine_biosynthesis_g__Blautia_s__Ruminococcus_torqu
UNINTEGRATED_g__Streptococcus_s__Streptococcus_salivarius
PWY_7184__pyrimidine_deoxyribonucleotides_de_novo_biosynthesis_I_unclassified
PWY_6608__guanosine_nucleotides_degradation_III_g__Blautia_s__Ruminococcus_torques
PWY_7323__superpathway_of_GDP_mannose_derived_O_antigen_building_blocks_biosynthesis_
PWY_6151__S_adenosyl_L_methionine_cycle_I_g__Streptococcus_s__Streptococcus_thermophilu
PWY_6700__queuosine_biosynthesis

PWY_6385__peptidoglycan_biosynthesis_III_(mycobacteria)
METSYN_PWY__L_homoserine_and_L_methionine_biosynthesis_g__Blautia_s__Ruminococcus_to
HOMOSER_METSYN_PWY__L_methionine_biosynthesis_I_unclassified
PWY0_1241__ADP_L_glycero_&beta__D_manno_heptose_biosynthesis_g__Haemophilus_s__Hae
PWY_5347__superpathway_of_L_methionine_biosynthesis_(transsulfuration)_g__Blautia_s__Rum
NONOXIPENT_PWY__pentose_phosphate_pathway_(non_oxidative_branch)_g__Blautia_s__Rumi
PWY0_166__superpathway_of_pyrimidine_deoxyribonucleotides_de_novo_biosynthesis_(E__coli)
PWY_821__superpathway_of_sulfur_amino_acid_biosynthesis_(Saccharomyces_cerevisiae)
PWY_7210__pyrimidine_deoxyribonucleotides_biosynthesis_from_CTP_g__Haemophilus_s__Hae
MET_SAM_PWY__superpathway_of_S_adenosyl_L_methionine_biosynthesis_g__Blautia_s__Rumi
MET_SAM_PWY__superpathway_of_S_adenosyl_L_methionine_biosynthesis
SALVADEHYPOX_PWY__adenosine_nucleotides_degradation_II_g__Blautia_s__Ruminococcus_tor
PWY_7663__gondoate_biosynthesis_(anaerobic)_g__Bacteroides_s__Bacteroides_fragilis
TRNA_CHARGING_PWY__tRNA_charging
PWY_6305__putrescine_biosynthesis_IV_g__Blautia_s__Ruminococcus_torques
PWY_5695__urate_biosynthesis_inosine_5__phosphate_degradation_g__Blautia_s__Ruminococcu
PWY_7198__pyrimidine_deoxyribonucleotides_de_novo_biosynthesis_IV_g__Haemophilus_s__Ha
DTDPRHAMSYN_PWY__dTDP_L_rhamnose_biosynthesis_I
PWY_6126__superpathway_of_adenosine_nucleotides_de_novo_biosynthesis_II_unclassified
P4_PWY__superpathway_of_L_lysine,_L_threonine_and_L_methionine_biosynthesis_I_unclassifie
PWY_7219__adenosine_ribonucleotides_de_novo_biosynthesis_g__Blautia_s__Ruminococcus_tor
COA_PWY_1__coenzyme_A_biosynthesis_II_(mammalian)_g__Bacteroides_s__Bacteroides_fragili
PWY_6549__L_glutamine_biosynthesis_III
PWY_5367__petroselinate_biosynthesis_g__Blautia_s__Ruminococcus_torques
PWY0_1296__purine_ribonucleosides_degradation_g__Blautia_s__Ruminococcus_torques
PWY_7115__C4_photosynthetic_carbon_assimilation_cycle,_NAD_ME_type_unclassified
METSYN_PWY__L_homoserine_and_L_methionine_biosynthesis
PWY_7357__thiamin_formation_from_pyrithiamine_and_oxythiamine_(yeast)_g__Lactococcus_s_
PWY_7357__thiamin_formation_from_pyrithiamine_and_oxythiamine_(yeast)_g__Blautia_s__Ru
PWY_6124__inosine_5__phosphate_biosynthesis_II_g__Haemophilus_s__Haemophilus_parainflue
PWY_4984__urea_cycle
HEXITOLDEGSUPER_PWY__superpathway_of_hexitol_degradation_(bacteria)_unclassified
HEMESYN2_PWY__heme_biosynthesis_II_(anaerobic)_g__Actinomyces_s__Actinomyces_odontoly
PWY_6703__preQ0_biosynthesis_g__Blautia_s__Ruminococcus_torques
PWY_6386__UDP_N_acetylmuramoyl_pentapeptide_biosynthesis_II_(lysine_containing)_g__Strep
VALSYN_PWY__L_valine_biosynthesis_g__Dorea_s__Dorea_longicatena
PWY_7111__pyruvate_fermentation_to_isobutanol_(engineered)_g__Haemophilus_s__Haemophi
HOMOSER_METSYN_PWY__L_methionine_biosynthesis_I
PWY_6609__adenine_and_adenosine_salvage_III_g__Blautia_s__Ruminococcus_torques
PWY_5667__CDP_diacylglycerol_biosynthesis_I_g__Blautia_s__Ruminococcus_torques
PWY0_1319__CDP_diacylglycerol_biosynthesis_II_g__Blautia_s__Ruminococcus_torques
RHAMCAT_PWY__L_rhamnose_degradation_I_g__Blautia_s__Ruminococcus_torques
PWY_5686__UMP_biosynthesis_g__Blautia_s__Ruminococcus_torques
PWY_5686__UMP_biosynthesis_g__Bacteroides_s__Bacteroides_sp_3_2_5
P105_PWY__TCA_cycle_IV_(2_oxoglutarate_decarboxylase)_unclassified
PWY_1042__glycolysis_IV_(plant_cytosol)
PWY_2942__L_lysine_biosynthesis_III_unclassified

COLANSYN_PWY__colanic_acid_building_blocks_biosynthesis_unclassified
PWY0_1297__superpathway_of_purine_deoxyribonucleosides_degradation_g__Blautia_s__Rumin
PWY_2942__L_lysine_biosynthesis_III_g__Blautia_s__Ruminococcus_torques
PWY_5941__glycogen_degradation_II_(eukaryotic)_unclassified
PWY_6317__galactose_degradation_I_(Leloir_pathway)_g__Blautia_s__Ruminococcus_torques
PWY66_422__D_galactose_degradation_V_(Leloir_pathway)_g__Blautia_s__Ruminococcus_torque
PWY_6151__S_adenosyl_L_methionine_cycle_I_g__Blautia_s__Ruminococcus_torques
GLUTORN_PWY__L_ornithine_biosynthesis_g__Blautia_s__Ruminococcus_torques
PWY_5686__UMP_biosynthesis_g__Bacteroides_s__Bacteroides_fragilis
PANTO_PWY__phosphopantothenate_biosynthesis_I_g__Bacteroides_s__Bacteroides_fragilis
COMPLETE_ARO_PWY__superpathway_of_aromatic_amino_acid_biosynthesis_g__Blautia_s__Run
PWY_7111__pyruvate_fermentation_to_isobutanol_(engineered)_g__Dorea_s__Dorea_longicaten
PWY_6936__seleno_amino_acid_biosynthesis_g__Dorea_s__Dorea_longicatena
SER_GLYSYN_PWY__superpathway_of_L_serine_and_glycine_biosynthesis_I_g__Blautia_s__Rumir
PWY_5973__cis_vaccenate_biosynthesis_g__Blautia_s__Ruminococcus_torques
PWY_6737__starch_degradation_V_g__Blautia_s__Ruminococcus_torques
DENOVOPURINE2_PWY__superpathway_of_purine_nucleotides_de_novo_biosynthesis_II_g__Hae
ARO_PWY__chorismate_biosynthesis_I_g__Blautia_s__Ruminococcus_torques
PANTO_PWY__phosphopantothenate_biosynthesis_I_g__Dorea_s__Dorea_longicatena
PWY0_781__aspartate_superpathway_unclassified
PWY_7187__pyrimidine_deoxyribonucleotides_de_novo_biosynthesis_II_g__Haemophilus_s__Hae
PWY0_1296__purine_ribonucleosides_degradation
PWY_5097__L_lysine_biosynthesis_VI_g__Blautia_s__Ruminococcus_torques
PWY_241__C4_photosynthetic_carbon_assimilation_cycle,_NADP_ME_type
CITRULBIO_PWY__L_citrulline_biosynthesis
PWY_6163__chorismate_biosynthesis_from_3_dehydroquinate_g__Blautia_s__Ruminococcus_tor
VALSYN_PWY__L_valine_biosynthesis_g__Blautia_s__Ruminococcus_torques
1CMET2_PWY__N10_formyl_tetrahydrofolate_biosynthesis_g__Bacteroides_s__Bacteroides_sp_3_
PWY_7208__superpathway_of_pyrimidine_nucleobases_salvage_g__Blautia_s__Ruminococcus_to
PEPTIDOGLYCANSYN_PWY__peptidoglycan_biosynthesis_I_(meso_diaminopimelate_containing)_g
COA_PWY__coenzyme_A_biosynthesis_I_g__Blautia_s__Ruminococcus_torques
PWY_6385__peptidoglycan_biosynthesis_III_(mycobacteria)_g__Blautia_s__Ruminococcus_torque
PWY_6387__UDP_N_acetylmuramoyl_pentapeptide_biosynthesis_I_(meso_diaminopimelate_cont
PWY_1042__glycolysis_IV_(plant_cytosol)_g__Blautia_s__Ruminococcus_torques
UNINTEGRATED_g__Bifidobacterium_s__Bifidobacterium_adolescentis
COA_PWY_1__coenzyme_A_biosynthesis_II_(mammalian)_g__Blautia_s__Ruminococcus_torques
PWY_6124__inosine_5__phosphate_biosynthesis_II_g__Bacteroides_s__Bacteroides_fragilis
TRPSYN_PWY__L_tryptophan_biosynthesis_g__Blautia_s__Ruminococcus_torques
PWY_7111__pyruvate_fermentation_to_isobutanol_(engineered)_g__Blautia_s__Ruminococcus_t
PWY_5097__L_lysine_biosynthesis_VI_g__Bacteroides_s__Bacteroides_sp_3_2_5
PWY_7003__glycerol_degradation_to_butanol_unclassified
MET_SAM_PWY__superpathway_of_S_adenosyl_L_methionine_biosynthesis_unclassified
PWY_5154__L_arginine_biosynthesis_III_(via_N_acetyl_L_citrulline)_g__Blautia_s__Ruminococcus
PWY_6122__5_aminoimidazole_ribonucleotide_biosynthesis_II_g__Dorea_s__Dorea_longicatena
PWY_6277__superpathway_of_5_aminoimidazole_ribonucleotide_biosynthesis_g__Dorea_s__Dor
P441_PWY__superpathway_of_N_acetylneuraminate_degradation_unclassified
PWY_7400__L_arginine_biosynthesis_IV_(archaebacteria)_g__Blautia_s__Ruminococcus_torques

ARGSYN_PWY__L_arginine_biosynthesis_I_(via_L_ornithine)_g__Blautia_s__Ruminococcus_torque
PWY_6386__UDP_N_acetylmuramoyl_pentapeptide_biosynthesis_II_(lysine_containing)_g__Blaut
ARGSYNBSUB_PWY__L_arginine_biosynthesis_II_(acetyl_cycle)_g__Blautia_s__Ruminococcus_torc
PYRIDOXSYN_PWY__pyridoxal_5__phosphate_biosynthesis_I_g__Bacteroides_s__Bacteroides_sp_
PWY0_845__superpathway_of_pyridoxal_5__phosphate_biosynthesis_and_salvage_g__Bacteroide
UNINTEGRATED_g__Dorea_s__Dorea_longicatena
PWY_6121__5_aminoimidazole_ribonucleotide_biosynthesis_I_g__Dorea_s__Dorea_longicatena
CITRULBIO_PWY__L_citrulline_biosynthesis_unclassified
GLUCONEO_PWY__gluconeogenesis_I_unclassified
PWY_6606__guanosine_nucleotides_degradation_II_unclassified
PANTO_PWY__phosphopantothenate_biosynthesis_I_g__Blautia_s__Ruminococcus_torques
COA_PWY__coenzyme_A_biosynthesis_I_g__Actinomyces_s__Actinomyces_odontolyticus
GLYCOGENSYNTH_PWY__glycogen_biosynthesis_I_(from_ADP_D_Glucose)
PWY_6386__UDP_N_acetylmuramoyl_pentapeptide_biosynthesis_II_(lysine_containing)_g__Bacte
PWY0_166__superpathway_of_pyrimidine_deoxyribonucleotides_de_novo_biosynthesis_(E__coli)
PWY0_1319__CDP_diacylglycerol_biosynthesis_II
PWY_5667__CDP_diacylglycerol_biosynthesis_I
SER_GLYSYN_PWY__superpathway_of_L_serine_and_glycine_biosynthesis_I_g__Actinomyces_s__
PYRIDOXSYN_PWY__pyridoxal_5__phosphate_biosynthesis_I_unclassified
PWY_7219__adenosine_ribonucleotides_de_novo_biosynthesis_g__Bacteroides_s__Bacteroides_f
PWY_821__superpathway_of_sulfur_amino_acid_biosynthesis_(Saccharomyces_cerevisiae)_uncla
PWY0_781__aspartate_superpathway
PWY0_1298__superpathway_of_pyrimidine_deoxyribonucleosides_degradation_unclassified
PWY_6385__peptidoglycan_biosynthesis_III_(mycobacteria)_g__Bacteroides_s__Bacteroides_sp_3
PWY_5464__superpathway_of_cytosolic_glycolysis_(plants),_pyruvate_dehydrogenase_and_TCA_
PWY_6125__superpathway_of_guanosine_nucleotides_de_novo_biosynthesis_II_g__Actinomyces_
COMPLETE_ARO_PWY__superpathway_of_aromatic_amino_acid_biosynthesis
PWY_5097__L_lysine_biosynthesis_VI_g__Actinomyces_s__Actinomyces_odontolyticus
METSYN_PWY__L_homoserine_and_L_methionine_biosynthesis_unclassified
PWY_7282__4_amino_2_methyl_5_phosphomethylpyrimidine_biosynthesis_(yeast)_g__Bacteroid
PYRIDNUCSAL_PWY__NAD_salvage_pathway_I
PEPTIDOGLYCANSYN_PWY__peptidoglycan_biosynthesis_I_(meso_diaminopimelate_containing)_g
ASPASN_PWY__superpathway_of_L_aspartate_and_L_asparagine_biosynthesis_unclassified
PANTO_PWY__phosphopantothenate_biosynthesis_I
PWY_5686__UMP_biosynthesis_g__Collinsella_s__Collinsella_aerofaciens
PWY_7560__methylerythritol_phosphate_pathway_II_unclassified
UNINTEGRATED_g__Ruminococcus_s__Ruminococcus_lactaris
PWY0_845__superpathway_of_pyridoxal_5__phosphate_biosynthesis_and_salvage_unclassified
PWY_6168__flavin_biosynthesis_III_(fungi)_unclassified
SER_GLYSYN_PWY__superpathway_of_L_serine_and_glycine_biosynthesis_I
DAPLYSINESYN_PWY__L_lysine_biosynthesis_I_g__Actinomyces_s__Actinomyces_odontolyticus
PWY_6167__flavin_biosynthesis_II_(archaea)
PYRIDOXSYN_PWY__pyridoxal_5__phosphate_biosynthesis_I
UNINTEGRATED_g__Actinomyces_s__Actinomyces_odontolyticus
PWY_6387__UDP_N_acetylmuramoyl_pentapeptide_biosynthesis_I_(meso_diaminopimelate_con
PWY_7221__guanosine_ribonucleotides_de_novo_biosynthesis_g__Actinomyces_s__Actinomyces
PWY_7228__superpathway_of_guanosine_nucleotides_de_novo_biosynthesis_I_g__Actinomyces_

PWY0_1479__tRNA_processing
PWY_5686__UMP_biosynthesis_g__Coprococcus_s__Coprococcus_comes
PWY_7219__adenosine_ribonucleotides_de_novo_biosynthesis_g__Coprococcus_s__Coprococcus_
PWY_5188__tetrapyrrole_biosynthesis_I_(from_glutamate)_g__Actinomyces_s__Actinomyces_od
PWY4LZ_257__superpathway_of_fermentation_(Chlamydomonas_reinhardtii)
PWY_4984__urea_cycle_unclassified
PWY_7220__adenosine_deoxyribonucleotides_de_novo_biosynthesis_II_g__Actinomyces_s__Acti
PWY_7222__guanosine_deoxyribonucleotides_de_novo_biosynthesis_II_g__Actinomyces_s__Acti
PWY_7196__superpathway_of_pyrimidine_ribonucleosides_salvage
PWY_6385__peptidoglycan_biosynthesis_III_(mycobacteria)_g__Bacteroides_s__Bacteroides_fragi
PWY_6122__5_aminoimidazole_ribonucleotide_biosynthesis_II_g__Coprococcus_s__Coprococcus_
PWY_6277__superpathway_of_5_aminoimidazole_ribonucleotide_biosynthesis_g__Coprococcus_s
PWY_7282__4_amino_2_methyl_5_phosphomethylpyrimidine_biosynthesis_(yeast)_unclassified
PWY_5097__L_lysine_biosynthesis_VI_unclassified
PWY_7219__adenosine_ribonucleotides_de_novo_biosynthesis_g__Bacteroides_s__Bacteroides_s
PWY_6703__preQ0_biosynthesis_g__Bacteroides_s__Bacteroides_sp_3_2_5
PWY_621__sucrose_degradation_III_(sucrose_invertase)_unclassified
POLYAMSYN_PWY__superpathway_of_polyamine_biosynthesis_I
ARO_PWY__chorismate_biosynthesis_I
ARGININE_SYN4_PWY__L_ornithine_de_novo__biosynthesis_unclassified
PWY_6121__5_aminoimidazole_ribonucleotide_biosynthesis_I_g__Coprococcus_s__Coprococcus_
RHAMCAT_PWY__L_rhamnose_degradation_I
PWY_7199__pyrimidine_deoxyribonucleosides_salvage_unclassified
UNINTEGRATED_g__Blautia_s__Ruminococcus_torques
PWY_6125__superpathway_of_guanosine_nucleotides_de_novo_biosynthesis_II_unclassified
PWY_6470__peptidoglycan_biosynthesis_V_(&beta__lactam_resistance)
NONMEVIPP_PWY__methylerythritol_phosphate_pathway_I
PWY_6588__pyruvate_fermentation_to_acetone_unclassified
PWY_7197__pyrimidine_deoxyribonucleotide_phosphorylation_g__Actinomyces_s__Actinomyces_
COA_PWY_1__coenzyme_A_biosynthesis_II_(mammalian)_g__Collinsella_s__Collinsella_aerofacie
PANTO_PWY__phosphopantothenate_biosynthesis_I_g__Actinomyces_s__Actinomyces_odontolyt
PWY_3841__folate_transformations_II_g__Bacteroides_s__Bacteroides_sp_3_2_5
ARGININE_SYN4_PWY__L_ornithine_de_novo__biosynthesis_g__Bacteroides_s__Bacteroides_frag
PWY_5659__GDP_mannose_biosynthesis
PWY_7229__superpathway_of_adenosine_nucleotides_de_novo_biosynthesis_I_unclassified
PWY_6609__adenine_and_adenosine_salvage_III_g__Bacteroides_s__Bacteroides_fragilis
PWY_6270__isoprene_biosynthesis_I_unclassified
PWY_6386__UDP_N_acetylmuramoyl_pentapeptide_biosynthesis_II_(lysine_containing)_g__Bacte
PWY_7228__superpathway_of_guanosine_nucleotides_de_novo_biosynthesis_I_unclassified
PWY_5667__CDP_diacylglycerol_biosynthesis_I_g__Bacteroides_s__Bacteroides_sp_3_2_5
PWY0_1319__CDP_diacylglycerol_biosynthesis_II_g__Bacteroides_s__Bacteroides_sp_3_2_5
PWY_6737__starch_degradation_V_g__Coprococcus_s__Coprococcus_comes
UNINTEGRATED_g__Coprococcus_s__Coprococcus_comes
P162_PWY__L_glutamate_degradation_V_(via_hydroxyglutarate)
UNINTEGRATED_g__Bacteroides_s__Bacteroides_sp_3_2_5
PWY_6386__UDP_N_acetylmuramoyl_pentapeptide_biosynthesis_II_(lysine_containing)_g__Copr
COA_PWY_1__coenzyme_A_biosynthesis_II_(mammalian)_g__Coprococcus_s__Coprococcus_com

PEPTIDOGLYCANSYN_PWY__peptidoglycan_biosynthesis_I_(meso_diaminopimelate_containing)_g
COA_PWY_1__coenzyme_A_biosynthesis_II_(mammalian)_g__Dorea_s__Dorea_longicatena
PWY_7197__pyrimidine_deoxyribonucleotide_phosphorylation_unclassified
PWY_7663__gondoate_biosynthesis_(anaerobic)_g__Bacteroides_s__Bacteroides_sp_3_2_5
PWY_6151__S_adenosyl_L_methionine_cycle_I_g__Coprococcus_s__Coprococcus_comes
PEPTIDOGLYCANSYN_PWY__peptidoglycan_biosynthesis_I_(meso_diaminopimelate_containing)_g
PWY_5667__CDP_diacylglycerol_biosynthesis_I_g__Coprococcus_s__Coprococcus_comes
PWY0_1319__CDP_diacylglycerol_biosynthesis_II_g__Coprococcus_s__Coprococcus_comes
CALVIN_PWY__Calvin_Benson_Bassham_cycle
VALSYN_PWY__L_valine_biosynthesis_g__Coprococcus_s__Coprococcus_comes
PWY_6163__chorismate_biosynthesis_from_3_dehydroquinate
PWY_6387__UDP_N_acetylmuramoyl_pentapeptide_biosynthesis_I_(meso_diaminopimelate_cont
PWY_7221__guanosine_ribonucleotides_de_novo_biosynthesis_g__Bacteroides_s__Bacteroides_f
PWY_5695__urate_biosynthesis_inosine_5__phosphate_degradation_g__Bacteroides_s__Bacteroi
PWY_6588__pyruvate_fermentation_to_acetone
PWY_5100__pyruvate_fermentation_to_acetate_and_lactate_II_unclassified
PWY_6609__adenine_and_adenosine_salvage_III_g__Bacteroides_s__Bacteroides_sp_3_2_5
PWY66_399__gluconeogenesis_III_unclassified
PWY_7391__isoprene_biosynthesis_II_(engineered)
PWY_6121__5_aminoimidazole_ribonucleotide_biosynthesis_I_g__Actinomyces_s__Actinomyces_
PWY_7242__D_fructuronate_degradation
PWY_5347__superpathway_of_L_methionine_biosynthesis_(transsulfuration)_unclassified
P164_PWY__purine_nucleobases_degradation_I_(anaerobic)
PWY_5189__tetrapyrrole_biosynthesis_II_(from_glycine)_g__Actinomyces_s__Actinomyces_odon
PWY_5695__urate_biosynthesis_inosine_5__phosphate_degradation_g__Bacteroides_s__Bacteroi
PWY_6545__pyrimidine_deoxyribonucleotides_de_novo_biosynthesis_III_unclassified
PWY_7111__pyruvate_fermentation_to_isobutanol_(engineered)_g__Coprococcus_s__Coprococcu
ARGORNPROST_PWY__arginine,_ornithine_and_proline_interconversion
PWY_7221__guanosine_ribonucleotides_de_novo_biosynthesis_g__Coprococcus_s__Coprococcus_
PWY_724__superpathway_of_L_lysine,_L_threonine_and_L_methionine_biosynthesis_II_g__Meth
P161_PWY__acetylene_degradation_unclassified
PWY_2942__L_lysine_biosynthesis_III_g__Methanobrevibacter_s__Methanobrevibacter_smithii
PWY_5686__UMP_biosynthesis_g__Actinomyces_s__Actinomyces_odontolyticus
PWY_6387__UDP_N_acetylmuramoyl_pentapeptide_biosynthesis_I_(meso_diaminopimelate_cont
ARG+POLYAMINE_SYN__superpathway_of_arginine_and_polyamine_biosynthesis_unclassified
DTDPRHAMSYN_PWY__dTDP_L_rhamnose_biosynthesis_I_g__Bacteroides_s__Bacteroides_sp_3_
PWY_5097__L_lysine_biosynthesis_VI_g__Methanobrevibacter_s__Methanobrevibacter_smithii
SO4ASSIM_PWY__sulfate_reduction_I_(assimilatory)_unclassified
GLYCOLYSIS_E_D__superpathway_of_glycolysis_and_Entner_Doudoroff_unclassified
PWY_2941__L_lysine_biosynthesis_II_g__Methanobrevibacter_s__Methanobrevibacter_smithii
1CMET2_PWY__N10_formyl_tetrahydrofolate_biosynthesis_g__Streptococcus_s__Streptococcus_
PWY_6609__adenine_and_adenosine_salvage_III_g__Actinomyces_s__Actinomyces_odontolyticus
GLUCUROCAT_PWY__superpathway_of_&beta__D_glucuronide_and_D_glucuronate_degradation
THRESYN_PWY__superpathway_of_L_threonine_biosynthesis_g__Methanobrevibacter_s__Methan
PWY_7221__guanosine_ribonucleotides_de_novo_biosynthesis_g__Bacteroides_s__Bacteroides_s
GALACT_GLUCUROCAT_PWY__superpathway_of_hexuronide_and_hexuronate_degradation
ARGININE_SYN4_PWY__L_ornithine_de_novo__biosynthesis_g__Bacteroides_s__Bacteroides_sp_

PWY_5198__factor_420_biosynthesis
PWY_5198__factor_420_biosynthesis_g__Methanobrevibacter_s__Methanobrevibacter_smithii
PWY_2942__L_lysine_biosynthesis_III_g__Bacteroides_s__Bacteroides_fragilis
COA_PWY_1__coenzyme_A_biosynthesis_II_(mammalian)_unclassified
PWY_2942__L_lysine_biosynthesis_III_g__Actinomyces_s__Actinomyces_odontolyticus
PWY_6897__thiamin_salvage_II_g__Methanobrevibacter_s__Methanobrevibacter_smithii
PWY_3001__superpathway_of_L_isoleucine_biosynthesis_I_g__Methanobrevibacter_s__Methano
PWY_5101__L_isoleucine_biosynthesis_II_g__Methanobrevibacter_s__Methanobrevibacter_smith
PWY_5104__L_isoleucine_biosynthesis_IV_g__Methanobrevibacter_s__Methanobrevibacter_smit
PWY_6121__5_aminoimidazole_ribonucleotide_biosynthesis_I_g__Methanobrevibacter_s__Meth
UNINTEGRATED_g__Methanobrevibacter_s__Methanobrevibacter_smithii
PWY_5189__tetrapyrrole_biosynthesis_II_(from_glycine)_g__Methanobrevibacter_s__Methanobr
UNINTEGRATED_unclassified
PWY_7242__D_fructuronate_degradation_g__Eubacterium_s__Eubacterium_rectale
PWY_7211__superpathway_of_pyrimidine_deoxyribonucleotides_de_novo_biosynthesis_unclassif
PWY_6353__purine_nucleotides_degradation_II_(aerobic)_unclassified
PWY_6122__5_aminoimidazole_ribonucleotide_biosynthesis_II_g__Methanobrevibacter_s__Meth
PWY_6277__superpathway_of_5_aminoimidazole_ribonucleotide_biosynthesis_g__Methanobrevi
PWY_6123__inosine_5__phosphate_biosynthesis_I_g__Bacteroides_s__Bacteroides_sp_3_2_5
BRANCHED_CHAIN_AA_SYN_PWY__superpathway_of_branched_amino_acid_biosynthesis_g__Me
PWY_6122__5_aminoimidazole_ribonucleotide_biosynthesis_II_g__Actinomyces_s__Actinomyces_
PWY_6277__superpathway_of_5_aminoimidazole_ribonucleotide_biosynthesis_g__Actinomyces_
PWY_6151__S_adenosyl_L_methionine_cycle_I_g__Streptococcus_s__Streptococcus_parasanguini
PWY_6167__flavin_biosynthesis_II_(archaea)_g__Methanobrevibacter_s__Methanobrevibacter_s
PWY_5464__superpathway_of_cytosolic_glycolysis_(plants),_pyruvate_dehydrogenase_and_TCA_
DTDPRHAMSYN_PWY__dTDP_L_rhamnose_biosynthesis_I_g__Methanobrevibacter_s__Methanob
PWY_241__C4_photosynthetic_carbon_assimilation_cycle,_NADP_ME_type_unclassified
PWY_6124__inosine_5__phosphate_biosynthesis_II_g__Bacteroides_s__Bacteroides_sp_3_2_5
PWY_7219__adenosine_ribonucleotides_de_novo_biosynthesis_g__Methanobrevibacter_s__Meth
PWY_7357__thiamin_formation_from_pyrithiamine_and_oxythiamine_(yeast)_g__Methanobrevib
PWY_5103__L_isoleucine_biosynthesis_III_g__Methanobrevibacter_s__Methanobrevibacter_smitl
PWY_5121__superpathway_of_geranylgeranyl_diphosphate_biosynthesis_II_(via_MEP)_unclassifie
METHANOGENESIS_PWY__methanogenesis_from_H2_and_CO2_g__Methanobrevibacter_s__Meth
METHANOGENESIS_PWY__methanogenesis_from_H2_and_CO2
HSERMETANA_PWY__L_methionine_biosynthesis_III_g__Methanobrevibacter_s__Methanobrevib
ANAGLYCOLYSIS_PWY__glycolysis_III_(from_glucose)_unclassified
GLYCOGENSYNTH_PWY__glycogen_biosynthesis_I_(from_ADP_D_Glucose)_unclassified
PANTO_PWY__phosphopantothenate_biosynthesis_I_g__Bacteroides_s__Bacteroides_sp_3_2_5
PWY_5188__tetrapyrrole_biosynthesis_I_(from_glutamate)_g__Methanobrevibacter_s__Methano
PWY_6936__seleno_amino_acid_biosynthesis_g__Methanobrevibacter_s__Methanobrevibacter_s
PWY_5097__L_lysine_biosynthesis_VI_g__Bacteroides_s__Bacteroides_fragilis
NONOXIPENT_PWY__pentose_phosphate_pathway_(non_oxidative_branch)
PWY_6549__L_glutamine_biosynthesis_III_unclassified
PWY_5005__biotin_biosynthesis_II_unclassified
ILEUSYN_PWY__L_isoleucine_biosynthesis_I_(from_threonine)_g__Methanobrevibacter_s__Meth
VALSYN_PWY__L_valine_biosynthesis_g__Methanobrevibacter_s__Methanobrevibacter_smithii
HISTSYN_PWY__L_histidine_biosynthesis_g__Methanobrevibacter_s__Methanobrevibacter_smith

PWY_6305__putrescine_biosynthesis_IV
FERMENTATION_PWY__mixed_acid_fermentation_unclassified
PWY_7208__superpathway_of_pyrimidine_nucleobases_salvage_unclassified
COA_PWY__coenzyme_A_biosynthesis_I_g__Roseburia_s__Roseburia_intestinalis
PWY0_1296__purine_ribonucleosides_degradation_g__Streptococcus_s__Streptococcus_salivarius
PWY_5686__UMP_biosynthesis_unclassified
GALACTUROCAT_PWY__D_galacturonate_degradation_I
PWY_7286__7_(3_amino_3_carboxypropyl)_wyosine_biosynthesis
PWY_7111__pyruvate_fermentation_to_isobutanol_(engineered)_g__Methanobrevibacter_s__Me
PWY_6163__chorismate_biosynthesis_from_3_dehydroquinate_g__Methanobrevibacter_s__Meth
PPGPPMET_PWY__ppGpp_biosynthesis_unclassified
PWY_7357__thiamin_formation_from_pyrithiamine_and_oxythiamine_(yeast)_unclassified
PWY_5505__L_glutamate_and_L_glutamine_biosynthesis
PWY_6174__mevalonate_pathway_II_(archaea)
VALSYN_PWY__L_valine_biosynthesis_g__Streptococcus_s__Streptococcus_salivarius
PWY_5304__superpathway_of_sulfur_oxidation_(Acidianus_ambivalens)_unclassified
PWY_7187__pyrimidine_deoxyribonucleotides_de_novo_biosynthesis_II_unclassified
PWY_7111__pyruvate_fermentation_to_isobutanol_(engineered)_g__Streptococcus_s__Streptoco
PWY_6507__4_deoxy_L_threo_hex_4_enopyranuronate_degradation
PWY_6317__galactose_degradation_I_(Leloir_pathway)_unclassified
PWY_6124__inosine_5__phosphate_biosynthesis_II_g__Streptococcus_s__Streptococcus_salivariu
PWY_5505__L_glutamate_and_L_glutamine_biosynthesis_unclassified
PWY_6609__adenine_and_adenosine_salvage_III_g__Streptococcus_s__Streptococcus_salivarius
PWY_841__superpathway_of_purine_nucleotides_de_novo_biosynthesis_I_unclassified
PWY_3841__folate_transformations_II_g__Streptococcus_s__Streptococcus_salivarius
NONMEVIPP_PWY__methylerythritol_phosphate_pathway_I_g__Dorea_s__Dorea_longicatena
PWY_6609__adenine_and_adenosine_salvage_III_unclassified
PRPP_PWY__superpathway_of_histidine,_purine,_and_pyrimidine_biosynthesis_unclassified
TRPSYN_PWY__L_tryptophan_biosynthesis_g__Bacteroides_s__Bacteroides_sp_3_2_5
DENOVOPURINE2_PWY__superpathway_of_purine_nucleotides_de_novo_biosynthesis_II_unclass
ASPASN_PWY__superpathway_of_L_aspartate_and_L_asparagine_biosynthesis_g__Roseburia_s__
NONOXIPENT_PWY__pentose_phosphate_pathway_(non_oxidative_branch)_unclassified
PWY_5484__glycolysis_II_(from_fructose_6_phosphate)_unclassified
PWY_6527__stachyose_degradation_unclassified
PWY_5022__4_aminobutanoate_degradation_V
P162_PWY__L_glutamate_degradation_V_(via_hydroxyglutarate)_unclassified
UNINTEGRATED_g__Bacteroides_s__Bacteroides_fragilis
PWY_6123__inosine_5__phosphate_biosynthesis_I_g__Streptococcus_s__Streptococcus_salivarius
PWY_7219__adenosine_ribonucleotides_de_novo_biosynthesis_g__Streptococcus_s__Streptococc
SULFATE_CYS_PWY__superpathway_of_sulfate_assimilation_and_cysteine_biosynthesis_unclassifi
UDPNAGSYN_PWY__UDP_N_acetyl_D_glucosamine_biosynthesis_I_g__Streptococcus_s__Streptoc
PWY_6703__preQ0_biosynthesis_unclassified
PWY_5100__pyruvate_fermentation_to_acetate_and_lactate_II_g__Actinomyces_s__Actinomyces
PWY0_1297__superpathway_of_purine_deoxyribonucleosides_degradation_g__Streptococcus_s__
PWY_6936__seleno_amino_acid_biosynthesis_unclassified
PWY_7219__adenosine_ribonucleotides_de_novo_biosynthesis_g__Actinomyces_s__Actinomyces
PWY_1861__formaldehyde_assimilation_II_(RuMP_Cycle)

PYRIDNUCSYN_PWY__NAD_biosynthesis_I_(from_aspartate)
PANTO_PWY__phosphopantothenate_biosynthesis_I_unclassified
PWY_724__superpathway_of_L_lysine,_L_threonine_and_L_methionine_biosynthesis_II_unclassifi
PWY_7219__adenosine_ribonucleotides_de_novo_biosynthesis_g__Ruminococcus_s__Ruminococ
PWY_6151__S_adenosyl_L_methionine_cycle_I_g__Streptococcus_s__Streptococcus_salivarius
PWY66_422__D_galactose_degradation_V_(Leloir_pathway)_unclassified
GLYCOLYSIS__glycolysis_I_(from_glucose_6_phosphate)_unclassified
PANTOSYN_PWY__pantothenate_and_coenzyme_A_biosynthesis_I_g__Blautia_s__Ruminococcus_
PWY_5676__acetyl_CoA_fermentation_to_butanoate_II_unclassified
PWY_7219__adenosine_ribonucleotides_de_novo_biosynthesis_unclassified
PWY_5030__L_histidine_degradation_III_unclassified
PWY_5695__urate_biosynthesis_inosine_5__phosphate_degradation_unclassified
P108_PWY__pyruvate_fermentation_to_propanoate_I_unclassified
ANAEROFRUCAT_PWY__homolactic_fermentation_unclassified
PWY_5667__CDP_diacylglycerol_biosynthesis_I_g__Roseburia_s__Roseburia_intestinalis
PWY0_1319__CDP_diacylglycerol_biosynthesis_II_g__Roseburia_s__Roseburia_intestinalis
TRPSYN_PWY__L_tryptophan_biosynthesis
PWY_6305__putrescine_biosynthesis_IV_g__Methanobrevibacter_s__Methanobrevibacter_smithi
PWY_5100__pyruvate_fermentation_to_acetate_and_lactate_II_g__Ruminococcus_s__Ruminococ
PWY_7221__guanosine_ribonucleotides_de_novo_biosynthesis_g__Streptococcus_s__Streptococc
PWY_5384__sucrose_degradation_IV_(sucrose_phosphorylase)_g__Roseburia_s__Roseburia_intes
PWY_6737__starch_degradation_V_g__Ruminococcus_s__Ruminococcus_lactaris
NONMEVIPP_PWY__methylerythritol_phosphate_pathway_I_g__Roseburia_s__Roseburia_intestir
PWY0_162__superpathway_of_pyrimidine_ribonucleotides_de_novo_biosynthesis_unclassified
NONOXIPENT_PWY__pentose_phosphate_pathway_(non_oxidative_branch)_g__Roseburia_s__Ro
VALSYN_PWY__L_valine_biosynthesis_g__Ruminococcus_s__Ruminococcus_lactaris
PWY_7111__pyruvate_fermentation_to_isobutanol_(engineered)_g__Ruminococcus_s__Ruminoc
GOLPDLCAT_PWY__superpathway_of_glycerol_degradation_to_1,3_propanediol_unclassified
PWY_5345__superpathway_of_L_methionine_biosynthesis_(by_sulfhydrylation)_unclassified
COA_PWY_1__coenzyme_A_biosynthesis_II_(mammalian)_g__Roseburia_s__Roseburia_intestinal
COA_PWY_1__coenzyme_A_biosynthesis_II_(mammalian)_g__Bifidobacterium_s__Bifidobacteriur
PWY66_398__TCA_cycle_III_(animals)
PWY_6124__inosine_5__phosphate_biosynthesis_II_g__Streptococcus_s__Streptococcus_thermop
THRESYN_PWY__superpathway_of_L_threonine_biosynthesis_unclassified
PEPTIDOGLYCANSYN_PWY__peptidoglycan_biosynthesis_I_(meso_diaminopimelate_containing)_g
PWY_7234__inosine_5__phosphate_biosynthesis_III_g__Methanobrevibacter_s__Methanobreviba
PWY_6122__5_aminoimidazole_ribonucleotide_biosynthesis_II_g__Streptococcus_s__Streptococc
PWY_6277__superpathway_of_5_aminoimidazole_ribonucleotide_biosynthesis_g__Streptococcus_
SER_GLYSYN_PWY__superpathway_of_L_serine_and_glycine_biosynthesis_I_unclassified
PWY_724__superpathway_of_L_lysine,_L_threonine_and_L_methionine_biosynthesis_II_g__Blaut
P164_PWY__purine_nucleobases_degradation_I_(anaerobic)_unclassified
PWY_7196__superpathway_of_pyrimidine_ribonucleosides_salvage_unclassified
PWY_5659__GDP_mannose_biosynthesis_g__Roseburia_s__Roseburia_intestinalis
UDPNAGSYN_PWY__UDP_N_acetyl_D_glucosamine_biosynthesis_I_g__Bifidobacterium_s__Bifido
PWY_6123__inosine_5__phosphate_biosynthesis_I_g__Streptococcus_s__Streptococcus_thermop
PWY_6151__S_adenosyl_L_methionine_cycle_I_unclassified
PWY_6386__UDP_N_acetylmuramoyl_pentapeptide_biosynthesis_II_(lysine_containing)_g__Rumi

HISTSYN_PWY__L_histidine_biosynthesis_g__Streptococcus_s__Streptococcus_salivarius
VALSYN_PWY__L_valine_biosynthesis_g__Bifidobacterium_s__Bifidobacterium_adolescentis
PWY_6387__UDP_N_acetylmuramoyl_pentapeptide_biosynthesis_I_(meso_diaminopimelate_cont
PWY_6124__inosine_5__phosphate_biosynthesis_II_g__Methanobrevibacter_s__Methanobreviba
PWY_5100__pyruvate_fermentation_to_acetate_and_lactate_II_g__Roseburia_s__Roseburia_inte
P124_PWY__Bifidobacterium_shunt_unclassified
PWY_6387__UDP_N_acetylmuramoyl_pentapeptide_biosynthesis_I_(meso_diaminopimelate_cont
PWY_6386__UDP_N_acetylmuramoyl_pentapeptide_biosynthesis_II_(lysine_containing)_g__Bifido
PWY_6124__inosine_5__phosphate_biosynthesis_II_unclassified
PEPTIDOGLYCANSYN_PWY__peptidoglycan_biosynthesis_I_(meso_diaminopimelate_containing)_g
HISTSYN_PWY__L_histidine_biosynthesis_g__Bifidobacterium_s__Bifidobacterium_adolescentis
PWY_7221__guanosine_ribonucleotides_de_novo_biosynthesis_g__Ruminococcus_s__Ruminococ
PYRIDNUCSYN_PWY__NAD_biosynthesis_I_(from_aspartate)_unclassified
PWY_6121__5_aminoimidazole_ribonucleotide_biosynthesis_I_g__Streptococcus_s__Streptococcu
PWY4LZ_257__superpathway_of_fermentation_(Chlamydomonas_reinhardtii)_unclassified
PWY_7219__adenosine_ribonucleotides_de_novo_biosynthesis_g__Streptococcus_s__Streptococc
NONMEVIPP_PWY__methylerythritol_phosphate_pathway_I_g__Bifidobacterium_s__Bifidobacter
PWY_6163__chorismate_biosynthesis_from_3_dehydroquinate_g__Eubacterium_s__Eubacterium_
UNINTEGRATED_g__Eubacterium_s__Eubacterium_rectale
PWY_6122__5_aminoimidazole_ribonucleotide_biosynthesis_II_g__Streptococcus_s__Streptococc
PWY_6277__superpathway_of_5_aminoimidazole_ribonucleotide_biosynthesis_g__Streptococcus_
PWY_6737__starch_degradation_V_g__Roseburia_s__Roseburia_intestinalis
TRPSYN_PWY__L_tryptophan_biosynthesis_g__Roseburia_s__Roseburia_intestinalis
PWY_7221__guanosine_ribonucleotides_de_novo_biosynthesis_g__Roseburia_s__Roseburia_intes
PWY_6122__5_aminoimidazole_ribonucleotide_biosynthesis_II_g__Bifidobacterium_s__Bifidobact
PWY_6277__superpathway_of_5_aminoimidazole_ribonucleotide_biosynthesis_g__Bifidobacteriu
DTDPRHAMSYN_PWY__dTDP_L_rhamnose_biosynthesis_I_g__Eubacterium_s__Eubacterium_recta
GLYCOGENSYNTH_PWY__glycogen_biosynthesis_I_(from_ADP_D_Glucose)_g__Roseburia_s__Rose
THRESYN_PWY__superpathway_of_L_threonine_biosynthesis_g__Bifidobacterium_s__Bifidobacte
PWY_6123__inosine_5__phosphate_biosynthesis_I_unclassified
SALVADEHYPOX_PWY__adenosine_nucleotides_degradation_II_g__Roseburia_s__Roseburia_intes
PWY_5667__CDP_diacylglycerol_biosynthesis_I_g__Eubacterium_s__Eubacterium_rectale
PWY0_1319__CDP_diacylglycerol_biosynthesis_II_g__Eubacterium_s__Eubacterium_rectale
PWY_5097__L_lysine_biosynthesis_VI_g__Eubacterium_s__Eubacterium_rectale
PWY_6124__inosine_5__phosphate_biosynthesis_II_g__Streptococcus_s__Streptococcus_parasan
PWY_6151__S_adenosyl_L_methionine_cycle_I_g__Bifidobacterium_s__Bifidobacterium_adolesce
PWY66_422__D_galactose_degradation_V_(Leloir_pathway)_g__Eubacterium_s__Eubacterium_re
PWY_6700__queuosine_biosynthesis_unclassified
PWY_5695__urate_biosynthesis_inosine_5__phosphate_degradation_g__Roseburia_s__Roseburia
PWY_6608__guanosine_nucleotides_degradation_III_g__Roseburia_s__Roseburia_intestinalis
PWY_7219__adenosine_ribonucleotides_de_novo_biosynthesis_g__Streptococcus_s__Streptococc
ARO_PWY__chorismate_biosynthesis_I_g__Eubacterium_s__Eubacterium_rectale
PWY_6737__starch_degradation_V_g__Eubacterium_s__Eubacterium_rectale
PWY0_1296__purine_ribonucleosides_degradation_g__Roseburia_s__Roseburia_intestinalis
COBALSYN_PWY__adenosylcobalamin_salvage_from_cobinamide_I_g__Eubacterium_s__Eubacter
SALVADEHYPOX_PWY__adenosine_nucleotides_degradation_II_unclassified
DTDPRHAMSYN_PWY__dTDP_L_rhamnose_biosynthesis_I_unclassified

COMPLETE_ARO_PWY__superpathway_of_aromatic_amino_acid_biosynthesis_g__Eubacterium_s_
UNINTEGRATED_g__Ruminococcus_s__Ruminococcus_sp_5_1_39BFAA
PWY_7219__adenosine_ribonucleotides_de_novo_biosynthesis_g__Bifidobacterium_s__Bifidobac
PWY_7111__pyruvate_fermentation_to_isobutanol_(engineered)_g__Eubacterium_s__Eubacteriu
VALSYN_PWY__L_valine_biosynthesis_g__Eubacterium_s__Eubacterium_rectale
PWY_5104__L_isoleucine_biosynthesis_IV_g__Roseburia_s__Roseburia_intestinalis
GLYCOGENSYNTH_PWY__glycogen_biosynthesis_I_(from_ADP_D_Glucose)_g__Eubacterium_s__E
POLYAMSYN_PWY__superpathway_of_polyamine_biosynthesis_I_unclassified
PWY_6123__inosine_5__phosphate_biosynthesis_I_g__Streptococcus_s__Streptococcus_parasang
PWY_7234__inosine_5__phosphate_biosynthesis_III_g__Streptococcus_s__Streptococcus_parasar
PWY_7219__adenosine_ribonucleotides_de_novo_biosynthesis_g__Eubacterium_s__Eubacterium_
PWY_7221__guanosine_ribonucleotides_de_novo_biosynthesis_g__Bifidobacterium_s__Bifidobac
PWY_6595__superpathway_of_guanosine_nucleotides_degradation_(plants)_unclassified
VALSYN_PWY__L_valine_biosynthesis_g__Roseburia_s__Roseburia_intestinalis
PWY_6121__5_aminoimidazole_ribonucleotide_biosynthesis_I_g__Streptococcus_s__Streptococcu
PANTO_PWY__phosphopantothenate_biosynthesis_I_g__Eubacterium_s__Eubacterium_rectale
PWY_6151__S_adenosyl_L_methionine_cycle_I_g__Ruminococcus_s__Ruminococcus_lactaris
PWY_5367__petroselinate_biosynthesis_g__Eubacterium_s__Eubacterium_rectale
TRPSYN_PWY__L_tryptophan_biosynthesis_unclassified
PWY_5188__tetrapyrrole_biosynthesis_I_(from_glutamate)_g__Ruminococcus_s__Ruminococcus_
PWY_6608__guanosine_nucleotides_degradation_III_unclassified
PYRIDNUCSYN_PWY__NAD_biosynthesis_I_(from_aspartate)_g__Roseburia_s__Roseburia_intestir
PWY_7111__pyruvate_fermentation_to_isobutanol_(engineered)_g__Ruminococcus_s__Ruminoc
SER_GLYSYN_PWY__superpathway_of_L_serine_and_glycine_biosynthesis_I_g__Roseburia_s__Ro
PWY_5097__L_lysine_biosynthesis_VI_g__Roseburia_s__Roseburia_intestinalis
PWY_1269__CMP_3_deoxy_D_manno_octulosonate_biosynthesis_I_unclassified
PWY_7003__glycerol_degradation_to_butanol
PWY_7221__guanosine_ribonucleotides_de_novo_biosynthesis_g__Eubacterium_s__Eubacterium_
PEPTIDOGLYCANSYN_PWY__peptidoglycan_biosynthesis_I_(meso_diaminopimelate_containing)_u
PWY_7221__guanosine_ribonucleotides_de_novo_biosynthesis_unclassified
CALVIN_PWY__Calvin_Benson_Bassham_cycle_unclassified
PWY_6737__starch_degradation_V
NONMEVIPP_PWY__methylerythritol_phosphate_pathway_I_g__Eubacterium_s__Eubacterium_re
PWY_6609__adenine_and_adenosine_salvage_III_g__Ruminococcus_s__Ruminococcus_lactaris
PWY_6121__5_aminoimidazole_ribonucleotide_biosynthesis_I_g__Streptococcus_s__Streptococcu
PANTO_PWY__phosphopantothenate_biosynthesis_I_g__Roseburia_s__Roseburia_intestinalis
PWY_6387__UDP_N_acetylmuramoyl_pentapeptide_biosynthesis_I_(meso_diaminopimelate_cont
PWY_6386__UDP_N_acetylmuramoyl_pentapeptide_biosynthesis_II_(lysine_containing)_g__Euba
COA_PWY_1__coenzyme_A_biosynthesis_II_(mammalian)_g__Eubacterium_s__Eubacterium_rect
PWY_6163__chorismate_biosynthesis_from_3_dehydroquinate_g__Roseburia_s__Roseburia_intes
PWY_6122__5_aminoimidazole_ribonucleotide_biosynthesis_II_g__Streptococcus_s__Streptococc
PWY_6277__superpathway_of_5_aminoimidazole_ribonucleotide_biosynthesis_g__Streptococcus_
UNINTEGRATED_g__Roseburia_s__Roseburia_intestinalis
PWY0_1296__purine_ribonucleosides_degradation_g__Eubacterium_s__Eubacterium_rectale
PWY_5686__UMP_biosynthesis_g__Eubacterium_s__Eubacterium_rectale
VALSYN_PWY__L_valine_biosynthesis_g__Ruminococcus_s__Ruminococcus_sp_5_1_39BFAA
PWY_6386__UDP_N_acetylmuramoyl_pentapeptide_biosynthesis_II_(lysine_containing)_unclassif

PANTOSYN_PWY__pantothenate_and_coenzyme_A_biosynthesis_I_unclassified
OANTIGEN_PWY__O_antigen_building_blocks_biosynthesis_(E__coli)_g__Bifidobacterium_s__Bifi
HISTSYN_PWY__L_histidine_biosynthesis_unclassified
PWY_6151__S_adenosyl_L_methionine_cycle_I_g__Eubacterium_s__Eubacterium_rectale
PWY_6151__S_adenosyl_L_methionine_cycle_I_g__Roseburia_s__Roseburia_intestinalis
PWY_7219__adenosine_ribonucleotides_de_novo_biosynthesis_g__Roseburia_s__Roseburia_intes
PWY_7199__pyrimidine_deoxyribonucleosides_salvage_g__Streptococcus_s__Streptococcus_para
COMPLETE_ARO_PWY__superpathway_of_aromatic_amino_acid_biosynthesis_g__Roseburia_s__
PWY_7219__adenosine_ribonucleotides_de_novo_biosynthesis_g__Ruminococcus_s__Ruminococ
PWY_7221__guanosine_ribonucleotides_de_novo_biosynthesis_g__Streptococcus_s__Streptococc
PWY_6387__UDP_N_acetylmuramoyl_pentapeptide_biosynthesis_I_(meso_diaminopimelate_con
PWY_6121__5_aminoimidazole_ribonucleotide_biosynthesis_I_g__Roseburia_s__Roseburia_intes
PWY_2942__L_lysine_biosynthesis_III_g__Ruminococcus_s__Ruminococcus_sp_5_1_39BFAA
ARO_PWY__chorismate_biosynthesis_I_g__Roseburia_s__Roseburia_intestinalis
PWY_6163__chorismate_biosynthesis_from_3_dehydroquinate_g__Ruminococcus_s__Ruminococ
PWY_6737__starch_degradation_V_g__Ruminococcus_s__Ruminococcus_sp_5_1_39BFAA
PWY_5973__cis_vaccenate_biosynthesis_g__Eubacterium_s__Eubacterium_rectale
PWY_7111__pyruvate_fermentation_to_isobutanol_(engineered)_unclassified
HISDEG_PWY__L_histidine_degradation_I_unclassified
PWY_6122__5_aminoimidazole_ribonucleotide_biosynthesis_II_g__Roseburia_s__Roseburia_intes
PWY_6277__superpathway_of_5_aminoimidazole_ribonucleotide_biosynthesis_g__Roseburia_s__
ILEUSYN_PWY__L_isoleucine_biosynthesis_I_(from_threonine)_unclassified
VALSYN_PWY__L_valine_biosynthesis_unclassified
PWY_7208__superpathway_of_pyrimidine_nucleobases_salvage_g__Roseburia_s__Roseburia_inte
PWY_3001__superpathway_of_L_isoleucine_biosynthesis_I_unclassified
PWY_6317__galactose_degradation_I_(Leloir_pathway)_g__Roseburia_s__Roseburia_intestinalis
PWY66_422__D_galactose_degradation_V_(Leloir_pathway)_g__Roseburia_s__Roseburia_intestin
PWY_3841__folate_transformations_II_g__Streptococcus_s__Streptococcus_parasanguinis
PWY_5097__L_lysine_biosynthesis_VI_g__Ruminococcus_s__Ruminococcus_sp_5_1_39BFAA
BRANCHED_CHAIN_AA_SYN_PWY__superpathway_of_branched_amino_acid_biosynthesis_g__Str
PWY0_1296__purine_ribonucleosides_degradation_g__Ruminococcus_s__Ruminococcus_sp_5_1_
PWY_5189__tetrapyrrole_biosynthesis_II_(from_glycine)_unclassified
PWY_7229__superpathway_of_adenosine_nucleotides_de_novo_biosynthesis_I_g__Streptococcus
COMPLETE_ARO_PWY__superpathway_of_aromatic_amino_acid_biosynthesis_g__Ruminococcus_
COA_PWY__coenzyme_A_biosynthesis_I_unclassified
PWY_5100__pyruvate_fermentation_to_acetate_and_lactate_II_g__Streptococcus_s__Streptococc
PWY_5686__UMP_biosynthesis_g__Ruminococcus_s__Ruminococcus_sp_5_1_39BFAA
ARO_PWY__chorismate_biosynthesis_I_g__Ruminococcus_s__Ruminococcus_sp_5_1_39BFAA
NONOXIPENT_PWY__pentose_phosphate_pathway_(non_oxidative_branch)_g__Ruminococcus_s_
PWY_5103__L_isoleucine_biosynthesis_III_g__Streptococcus_s__Streptococcus_parasanguinis
NONMEVIPP_PWY__methylerythritol_phosphate_pathway_I_g__Ruminococcus_s__Ruminococcus
PWY66_399__gluconeogenesis_III
PWY0_1319__CDP_diacylglycerol_biosynthesis_II_unclassified
PWY_5667__CDP_diacylglycerol_biosynthesis_I_unclassified
COBALSYN_PWY__adenosylcobalamin_salvage_from_cobinamide_I_unclassified
UNINTEGRATED_g__Streptococcus_s__Streptococcus_parasanguinis
PWY_5667__CDP_diacylglycerol_biosynthesis_I_g__Ruminococcus_s__Ruminococcus_sp_5_1_39B

PWY0_1319__CDP_diacylglycerol_biosynthesis_II_g__Ruminococcus_s__Ruminococcus_sp_5_1_3
PWY_6609__adenine_and_adenosine_salvage_III_g__Streptococcus_s__Streptococcus_parasangu
PWY0_1296__purine_ribonucleosides_degradation_g__Streptococcus_s__Streptococcus_parasang
PWY_5659__GDP_mannose_biosynthesis_unclassified
ILEUSYN_PWY__L_isoleucine_biosynthesis_I_(from_threonine)_g__Streptococcus_s__Streptococc
VALSYN_PWY__L_valine_biosynthesis_g__Streptococcus_s__Streptococcus_parasanguinis
PWY_7357__thiamin_formation_from_pyrithiamine_and_oxythiamine_(yeast)_g__Roseburia_s__
PWY_6386__UDP_N_acetylmuramoyl_pentapeptide_biosynthesis_II_(lysine_containing)_g__Rumi
PWY_6609__adenine_and_adenosine_salvage_III_g__Streptococcus_s__Streptococcus_thermophi
PEPTIDOGLYCANSYN_PWY__peptidoglycan_biosynthesis_I_(meso_diaminopimelate_containing)_g
PWY_7221__guanosine_ribonucleotides_de_novo_biosynthesis_g__Ruminococcus_s__Ruminococ
PANTO_PWY__phosphopantothenate_biosynthesis_I_g__Ruminococcus_s__Ruminococcus_lactari
PWY_6317__galactose_degradation_I_(Leloir_pathway)_g__Streptococcus_s__Streptococcus_ther
PWY66_422__D_galactose_degradation_V_(Leloir_pathway)_g__Streptococcus_s__Streptococcus_
PWY_621__sucrose_degradation_III_(sucrose_invertase)_g__Streptococcus_s__Streptococcus_par
UNINTEGRATED_g__Streptococcus_s__Streptococcus_thermophilus
PWY_6387__UDP_N_acetylmuramoyl_pentapeptide_biosynthesis_I_(meso_diaminopimelate_con
PWY_7228__superpathway_of_guanosine_nucleotides_de_novo_biosynthesis_I_g__Streptococcus
NONMEVIPP_PWY__methylerythritol_phosphate_pathway_I_g__Bifidobacterium_s__Bifidobacter
PWY_5667__CDP_diacylglycerol_biosynthesis_I_g__Ruminococcus_s__Ruminococcus_lactaris
PWY0_1319__CDP_diacylglycerol_biosynthesis_II_g__Ruminococcus_s__Ruminococcus_lactaris
PWY_6386__UDP_N_acetylmuramoyl_pentapeptide_biosynthesis_II_(lysine_containing)_g__Strep
TCA__TCA_cycle_I_(prokaryotic)_unclassified
COMPLETE_ARO_PWY__superpathway_of_aromatic_amino_acid_biosynthesis_g__Streptococcus_
PWY_7229__superpathway_of_adenosine_nucleotides_de_novo_biosynthesis_I_g__Streptococcus
PWY_7208__superpathway_of_pyrimidine_nucleobases_salvage_g__Streptococcus_s__Streptococ
PWY0_1061__superpathway_of_L_alanine_biosynthesis_g__Streptococcus_s__Streptococcus_para
VALSYN_PWY__L_valine_biosynthesis_g__Streptococcus_s__Streptococcus_thermophilus
PWY_5177__glutaryl_CoA_degradation_unclassified
TRPSYN_PWY__L_tryptophan_biosynthesis_g__Streptococcus_s__Streptococcus_parasanguinis
PWY_6122__5_aminoimidazole_ribonucleotide_biosynthesis_II_unclassified
PWY_6277__superpathway_of_5_aminoimidazole_ribonucleotide_biosynthesis_unclassified
PWY_7197__pyrimidine_deoxyribonucleotide_phosphorylation_g__Streptococcus_s__Streptococc
PWY_6121__5_aminoimidazole_ribonucleotide_biosynthesis_I_unclassified
ARO_PWY__chorismate_biosynthesis_I_g__Streptococcus_s__Streptococcus_salivarius
PWY_7228__superpathway_of_guanosine_nucleotides_de_novo_biosynthesis_I_g__Streptococcus
COA_PWY_1__coenzyme_A_biosynthesis_II_(mammalian)_g__Ruminococcus_s__Ruminococcus_s
PWY_6387__UDP_N_acetylmuramoyl_pentapeptide_biosynthesis_I_(meso_diaminopimelate_con
PWY_5030__L_histidine_degradation_III_g__Streptococcus_s__Streptococcus_parasanguinis
PWY_7111__pyruvate_fermentation_to_isobutanol_(engineered)_g__Streptococcus_s__Streptoco
PWY_6386__UDP_N_acetylmuramoyl_pentapeptide_biosynthesis_II_(lysine_containing)_g__Strep
UDPNAGSYN_PWY__UDP_N_acetyl_D_glucosamine_biosynthesis_I_g__Streptococcus_s__Streptoc
LACTOSECAT_PWY__lactose_and_galactose_degradation_I_g__Streptococcus_s__Streptococcus_p
ARO_PWY__chorismate_biosynthesis_I_g__Streptococcus_s__Streptococcus_thermophilus
PWY_6163__chorismate_biosynthesis_from_3_dehydroquinate_g__Streptococcus_s__Streptococc
PWY_6151__S_adenosyl_L_methionine_cycle_I_g__Ruminococcus_s__Ruminococcus_sp_5_1_39E
COMPLETE_ARO_PWY__superpathway_of_aromatic_amino_acid_biosynthesis_g__Streptococcus_

PWY_6163__chorismate_biosynthesis_from_3_dehydroquinate_g__Streptococcus_s__Streptococc
PANTO_PWY__phosphopantothenate_biosynthesis_I_g__Ruminococcus_s__Ruminococcus_sp_5_
PWY_5188__tetrapyrrole_biosynthesis_I_(from_glutamate)_unclassified
PWY_7208__superpathway_of_pyrimidine_nucleobases_salvage_g__Streptococcus_s__Streptococ
PWY_1042__glycolysis_IV_(plant_cytosol)_unclassified
POLYAMINSYN3_PWY__superpathway_of_polyamine_biosynthesis_II
HISDEG_PWY__L_histidine_degradation_I_g__Streptococcus_s__Streptococcus_parasanguinis
PWY_6737__starch_degradation_V_g__Streptococcus_s__Streptococcus_parasanguinis
UNINTEGRATED_g__Faecalibacterium_s__Faecalibacterium_prausnitzii
BRANCHED_CHAIN_AA_SYN_PWY__superpathway_of_branched_amino_acid_biosynthesis_unclas
PWY_4981__L_proline_biosynthesis_II_(from_arginine)_g__Streptococcus_s__Streptococcus_para
PWY_7197__pyrimidine_deoxyribonucleotide_phosphorylation_g__Streptococcus_s__Streptococc
PWY0_1586__peptidoglycan_maturation_(meso_diaminopimelate_containing)_unclassified
PWY_3841__folate_transformations_II_g__Streptococcus_s__Streptococcus_thermophilus
HISDEG_PWY__L_histidine_degradation_I
COMPLETE_ARO_PWY__superpathway_of_aromatic_amino_acid_biosynthesis_g__Streptococcus_
ARO_PWY__chorismate_biosynthesis_I_g__Streptococcus_s__Streptococcus_parasanguinis
PWY_5177__glutaryl_CoA_degradation
TRNA_CHARGING_PWY__tRNA_charging_unclassified
PWY_6163__chorismate_biosynthesis_from_3_dehydroquinate_g__Streptococcus_s__Streptococc
PWY_6590__superpathway_of_Clostridium_acetobutylicum_acidogenic_fermentation
PWY_7237__myo_,_chiro__and_scillo_inositol_degradation
CRNFORCAT_PWY__creatinine_degradation_I_unclassified
CENTFERM_PWY__pyruvate_fermentation_to_butanoate
PWY_6527__stachyose_degradation_g__Dorea_s__Dorea_longicatena
PWY_5103__L_isoleucine_biosynthesis_III_unclassified
PWY0_1297__superpathway_of_purine_deoxyribonucleosides_degradation_g__Streptococcus_s__
PWY0_1296__purine_ribonucleosides_degradation_unclassified
PEPTIDOGLYCANSYN_PWY__peptidoglycan_biosynthesis_I_(meso_diaminopimelate_containing)_g
HSERMETANA_PWY__L_methionine_biosynthesis_III_unclassified
PWY_6703__preQ0_biosynthesis_g__Streptococcus_s__Streptococcus_salivarius
PWY_6737__starch_degradation_V_unclassified
COA_PWY_1__coenzyme_A_biosynthesis_II_(mammalian)_g__Streptococcus_s__Streptococcus_p
PWY_7209__superpathway_of_pyrimidine_ribonucleosides_degradation
UNINTEGRATED_g__Ruminococcus_s__Ruminococcus_bromii
GLUDEG_I_PWY__GABA_shunt
PWY_5101__L_isoleucine_biosynthesis_II_unclassified
PWY_6628__superpathway_of_L_phenylalanine_biosynthesis_g__Streptococcus_s__Streptococcus
PWY_6163__chorismate_biosynthesis_from_3_dehydroquinate_unclassified
HSERMETANA_PWY__L_methionine_biosynthesis_III
PWY_5686__UMP_biosynthesis_g__Streptococcus_s__Streptococcus_parasanguinis
PWY_7237__myo_,_chiro__and_scillo_inositol_degradation_unclassified
GLCMANNANAUT_PWY__superpathway_of_N_acetylglucosamine,_N_acetylmannosamine_and_N
CALVIN_PWY__Calvin_Benson_Bassham_cycle_g__Faecalibacterium_s__Faecalibacterium_prausni
PYRIDNUCSYN_PWY__NAD_biosynthesis_I_(from_aspartate)_g__Ruminococcus_s__Ruminococcus
NONOXIPENT_PWY__pentose_phosphate_pathway_(non_oxidative_branch)_g__Ruminococcus_s_
NONMEVIPP_PWY__methylerythritol_phosphate_pathway_I_unclassified

COMPLETE_ARO_PWY__superpathway_of_aromatic_amino_acid_biosynthesis_unclassified
PWY_5304__superpathway_of_sulfur_oxidation_(Acidianus_ambivalens)_g__Ruminococcus_s__Ru
PWY_7219__adenosine_ribonucleotides_de_novo_biosynthesis_g__Ruminococcus_s__Ruminococ
PWY_1042__glycolysis_IV_(plant_cytosol)_g__Faecalibacterium_s__Faecalibacterium_prausnitzii
PWY_1042__glycolysis_IV_(plant_cytosol)_g__Ruminococcus_s__Ruminococcus_bromii
ARO_PWY__chorismate_biosynthesis_I_unclassified
GLCMANNANAUT_PWY__superpathway_of_N_acetylglucosamine,_N_acetylmannosamine_and_N
PWY_5188__tetrapyrrole_biosynthesis_I_(from_glutamate)_g__Ruminococcus_s__Ruminococcus_
COA_PWY__coenzyme_A_biosynthesis_I_g__Streptococcus_s__Streptococcus_parasanguinis
PWY_6123__inosine_5__phosphate_biosynthesis_I_g__Faecalibacterium_s__Faecalibacterium_pra
PWY_4242__pantothenate_and_coenzyme_A_biosynthesis_III_g__Streptococcus_s__Streptococcu
PWY_6969__TCA_cycle_V_(2_oxoglutarate_ferredoxin_oxidoreductase)_unclassified
PWY_6124__inosine_5__phosphate_biosynthesis_II_g__Faecalibacterium_s__Faecalibacterium_pr
CRNFORCAT_PWY__creatinine_degradation_I
CALVIN_PWY__Calvin_Benson_Bassham_cycle_g__Ruminococcus_s__Ruminococcus_bromii
GALACTUROCAT_PWY__D_galacturonate_degradation_I_g__Faecalibacterium_s__Faecalibacteriu
PWY_5686__UMP_biosynthesis_g__Ruminococcus_s__Ruminococcus_bromii
SER_GLYSYN_PWY__superpathway_of_L_serine_and_glycine_biosynthesis_I_g__Ruminococcus_s_
PWY0_1296__purine_ribonucleosides_degradation_g__Streptococcus_s__Streptococcus_thermop
HISTSYN_PWY__L_histidine_biosynthesis_g__Ruminococcus_s__Ruminococcus_bromii
PWY_6151__S_adenosyl_L_methionine_cycle_I_g__Ruminococcus_s__Ruminococcus_bromii
GALACT_GLUCUROCAT_PWY__superpathway_of_hexuronide_and_hexuronate_degradation_g__F
PWY_6151__S_adenosyl_L_methionine_cycle_I_g__Dorea_s__Dorea_longicatena
PWY_6151__S_adenosyl_L_methionine_cycle_I_g__Bacteroides_s__Bacteroides_vulgatus
PWY_6609__adenine_and_adenosine_salvage_III_g__Faecalibacterium_s__Faecalibacterium_prau
PWY_621__sucrose_degradation_III_(sucrose_invertase)_g__Faecalibacterium_s__Faecalibacteriu
PWY_6737__starch_degradation_V_g__Ruminococcus_s__Ruminococcus_bromii
PWY_7242__D_fructuronate_degradation_g__Faecalibacterium_s__Faecalibacterium_prausnitzii
PWY_7282__4_amino_2_methyl_5_phosphomethylpyrimidine_biosynthesis_(yeast)
HOMOSER_METSYN_PWY__L_methionine_biosynthesis_I_g__Ruminococcus_s__Ruminococcus_br
GLUCUROCAT_PWY__superpathway_of_&beta__D_glucuronide_and_D_glucuronate_degradation_
ILEUSYN_PWY__L_isoleucine_biosynthesis_I_(from_threonine)_g__Faecalibacterium_s__Faecaliba
VALSYN_PWY__L_valine_biosynthesis_g__Faecalibacterium_s__Faecalibacterium_prausnitzii
PWY_7219__adenosine_ribonucleotides_de_novo_biosynthesis_g__Faecalibacterium_s__Faecalib
PWY_6737__starch_degradation_V_g__Faecalibacterium_s__Faecalibacterium_prausnitzii
BRANCHED_CHAIN_AA_SYN_PWY__superpathway_of_branched_amino_acid_biosynthesis_g__Fae
PWY_6317__galactose_degradation_I_(Leloir_pathway)_g__Ruminococcus_s__Ruminococcus_bro
PWY66_422__D_galactose_degradation_V_(Leloir_pathway)_g__Ruminococcus_s__Ruminococcus
DTDPRHAMSYN_PWY__dTDP_L_rhamnose_biosynthesis_I_g__Faecalibacterium_s__Faecalibacteri
COA_PWY_1__coenzyme_A_biosynthesis_II_(mammalian)_g__Bacteroides_s__Bacteroides_vulgat
PWY_5686__UMP_biosynthesis_g__Faecalibacterium_s__Faecalibacterium_prausnitzii
PWY_3001__superpathway_of_L_isoleucine_biosynthesis_I_g__Faecalibacterium_s__Faecalibacte
THRESYN_PWY__superpathway_of_L_threonine_biosynthesis_g__Faecalibacterium_s__Faecalibac
PWY_6317__galactose_degradation_I_(Leloir_pathway)_g__Faecalibacterium_s__Faecalibacteriun
PWY66_422__D_galactose_degradation_V_(Leloir_pathway)_g__Faecalibacterium_s__Faecalibacte
PWY_5103__L_isoleucine_biosynthesis_III_g__Faecalibacterium_s__Faecalibacterium_prausnitzii
NONOXIPENT_PWY__pentose_phosphate_pathway_(non_oxidative_branch)_g__Faecalibacterium_

PWY_7357__thiamin_formation_from_pyrithiamine_and_oxythiamine_(yeast)_g__Ruminococcus_
PWY_6386__UDP_N_acetylmuramoyl_pentapeptide_biosynthesis_II_(lysine_containing)_g__Faeca
PWY_5695__urate_biosynthesis_inosine_5__phosphate_degradation_g__Bacteroides_s__Bacteroi
PWY_7111__pyruvate_fermentation_to_isobutanol_(engineered)_g__Faecalibacterium_s__Faecal
PWY_3841__folate_transformations_II_g__Bacteroides_s__Bacteroides_vulgatus
PWY_5177__glutaryl_CoA_degradation_g__Faecalibacterium_s__Faecalibacterium_prausnitzii
PWY_6387__UDP_N_acetylmuramoyl_pentapeptide_biosynthesis_I_(meso_diaminopimelate_con
NONMEVIPP_PWY__methylerythritol_phosphate_pathway_I_g__Bacteroides_s__Bacteroides_vulg
PWY_7111__pyruvate_fermentation_to_isobutanol_(engineered)_g__Ruminococcus_s__Ruminoco
VALSYN_PWY__L_valine_biosynthesis_g__Ruminococcus_s__Ruminococcus_bromii
PWY_7111__pyruvate_fermentation_to_isobutanol_(engineered)_g__Streptococcus_s__Streptoco
PWY_7111__pyruvate_fermentation_to_isobutanol_(engineered)_g__Bacteroides_s__Bacteroides
VALSYN_PWY__L_valine_biosynthesis_g__Bacteroides_s__Bacteroides_vulgatus
PWY_6122__5_aminoimidazole_ribonucleotide_biosynthesis_II_g__Faecalibacterium_s__Faecaliba
PWY_6277__superpathway_of_5_aminoimidazole_ribonucleotide_biosynthesis_g__Faecalibacteri
PWY_6121__5_aminoimidazole_ribonucleotide_biosynthesis_I_g__Faecalibacterium_s__Faecaliba
PWY_6385__peptidoglycan_biosynthesis_III_(mycobacteria)_g__Faecalibacterium_s__Faecalibacte
PWY0_1586__peptidoglycan_maturation_(meso_diaminopimelate_containing)_g__Faecalibacteri
PEPTIDOGLYCANSYN_PWY__peptidoglycan_biosynthesis_I_(meso_diaminopimelate_containing)_g
PWY0_1296__purine_ribonucleosides_degradation_g__Faecalibacterium_s__Faecalibacterium_pr
ASPASN_PWY__superpathway_of_L_aspartate_and_L_asparagine_biosynthesis_g__Faecalibacteri
PWY_5695__urate_biosynthesis_inosine_5__phosphate_degradation_g__Faecalibacterium_s__Fa
SER_GLYSYN_PWY__superpathway_of_L_serine_and_glycine_biosynthesis_I_g__Faecalibacterium_
PWY_5097__L_lysine_biosynthesis_VI_g__Faecalibacterium_s__Faecalibacterium_prausnitzii
PWY_7221__guanosine_ribonucleotides_de_novo_biosynthesis_g__Ruminococcus_s__Ruminococ
PWY_7219__adenosine_ribonucleotides_de_novo_biosynthesis_g__Bacteroides_s__Bacteroides_v
COA_PWY_1__coenzyme_A_biosynthesis_II_(mammalian)_g__Faecalibacterium_s__Faecalibacteri
PWY_5659__GDP_mannose_biosynthesis_g__Faecalibacterium_s__Faecalibacterium_prausnitzii
PWY_7221__guanosine_ribonucleotides_de_novo_biosynthesis_g__Bacteroides_s__Bacteroides_v
PWY_7221__guanosine_ribonucleotides_de_novo_biosynthesis_g__Faecalibacterium_s__Faecaliba
PWY_6737__starch_degradation_V_g__Dorea_s__Dorea_longicatena
PEPTIDOGLYCANSYN_PWY__peptidoglycan_biosynthesis_I_(meso_diaminopimelate_containing)_g
PWY_7228__superpathway_of_guanosine_nucleotides_de_novo_biosynthesis_I_g__Bacteroides_s
PWY_5667__CDP_diacylglycerol_biosynthesis_I_g__Faecalibacterium_s__Faecalibacterium_prausn
PWY0_1319__CDP_diacylglycerol_biosynthesis_II_g__Faecalibacterium_s__Faecalibacterium_prau
UNINTEGRATED_g__Bacteroides_s__Bacteroides_vulgatus
PWY_6121__5_aminoimidazole_ribonucleotide_biosynthesis_I_g__Ruminococcus_s__Ruminococc
PWY_6387__UDP_N_acetylmuramoyl_pentapeptide_biosynthesis_I_(meso_diaminopimelate_con
PWY_6151__S_adenosyl_L_methionine_cycle_I_g__Faecalibacterium_s__Faecalibacterium_prausn
P42_PWY__incomplete_reductive_TCA_cycle_unclassified
PANTO_PWY__phosphopantothenate_biosynthesis_I_g__Bacteroides_s__Bacteroides_vulgatus
PWY_724__superpathway_of_L_lysine,_L_threonine_and_L_methionine_biosynthesis_II_g__Faeca
PWY0_845__superpathway_of_pyridoxal_5__phosphate_biosynthesis_and_salvage
PWY_7229__superpathway_of_adenosine_nucleotides_de_novo_biosynthesis_I_g__Bacteroides_s
PWY_6122__5_aminoimidazole_ribonucleotide_biosynthesis_II_g__Ruminococcus_s__Ruminococ
PWY_6277__superpathway_of_5_aminoimidazole_ribonucleotide_biosynthesis_g__Ruminococcus
PWY_4242__pantothenate_and_coenzyme_A_biosynthesis_III_unclassified

PWY_6507__4_deoxy_L_threo_hex_4_enopyranuronate_degradation_g__Faecalibacterium_s__Fa
PWY_6126__superpathway_of_adenosine_nucleotides_de_novo_biosynthesis_II_g__Bacteroides_
RHAMCAT_PWY__L_rhamnose_degradation_I_unclassified
PWY_6527__stachyose_degradation_g__Faecalibacterium_s__Faecalibacterium_prausnitzii
PWY_6703__preQ0_biosynthesis_g__Bacteroides_s__Bacteroides_vulgatus
PWY_5667__CDP_diacylglycerol_biosynthesis_I_g__Ruminococcus_s__Ruminococcus_bromii
PWY0_1319__CDP_diacylglycerol_biosynthesis_II_g__Ruminococcus_s__Ruminococcus_bromii
PWY_6125__superpathway_of_guanosine_nucleotides_de_novo_biosynthesis_II_g__Bacteroides_
PWY_2942__L_lysine_biosynthesis_III_g__Bacteroides_s__Bacteroides_vulgatus
PWY_6700__queuosine_biosynthesis_g__Bacteroides_s__Bacteroides_vulgatus
PWY_5690__TCA_cycle_II_(plants_and_fungi)_unclassified
COA_PWY__coenzyme_A_biosynthesis_I_g__Faecalibacterium_s__Faecalibacterium_prausnitzii
PWY_5104__L_isoleucine_biosynthesis_IV_unclassified
PWY_7357__thiamin_formation_from_pyrithiamine_and_oxythiamine_(yeast)_g__Faecalibacteriu
PWY_5097__L_lysine_biosynthesis_VI_g__Bacteroides_s__Bacteroides_vulgatus
PWY_7220__adenosine_deoxyribonucleotides_de_novo_biosynthesis_II_g__Bacteroides_s__Bacte
PWY_7222__guanosine_deoxyribonucleotides_de_novo_biosynthesis_II_g__Bacteroides_s__Bacte
RHAMCAT_PWY__L_rhamnose_degradation_I_g__Faecalibacterium_s__Faecalibacterium_prausni
PWY_6385__peptidoglycan_biosynthesis_III_(mycobacteria)_unclassified
COBALSYN_PWY__adenosylcobalamin_salvage_from_cobinamide_I_g__Faecalibacterium_s__Faec
PWY_5101__L_isoleucine_biosynthesis_II
PWY_7383__anaerobic_energy_metabolism_(invertebrates,_cytosol)_unclassified
THISYNARA_PWY__superpathway_of_thiamin_diphosphate_biosynthesis_III_(eukaryotes)_g__Fae
PWY_6151__S_adenosyl_L_methionine_cycle_I_g__Collinsella_s__Collinsella_aerofaciens
PWY_4242__pantothenate_and_coenzyme_A_biosynthesis_III_g__Faecalibacterium_s__Faecaliba
GALACTUROCAT_PWY__D_galacturonate_degradation_I_unclassified
PWY_7456__mannan_degradation
PWY_7383__anaerobic_energy_metabolism_(invertebrates,_cytosol)
ARGININE_SYN4_PWY__L_ornithine_de_novo__biosynthesis
PWY_7219__adenosine_ribonucleotides_de_novo_biosynthesis_g__Collinsella_s__Collinsella_aer
PWY_4981__L_proline_biosynthesis_II_(from_arginine)_g__Collinsella_s__Collinsella_aerofaciens
PWY_6387__UDP_N_acetylmuramoyl_pentapeptide_biosynthesis_I_(meso_diaminopimelate_con
UNINTEGRATED_g__Collinsella_s__Collinsella_aerofaciens
PWY_6386__UDP_N_acetylmuramoyl_pentapeptide_biosynthesis_II_(lysine_containing)_g__Collir
PWY_5030__L_histidine_degradation_III
PWY_6737__starch_degradation_V_g__Collinsella_s__Collinsella_aerofaciens
PEPTIDOGLYCANSYN_PWY__peptidoglycan_biosynthesis_I_(meso_diaminopimelate_containing)_g
PWY_5104__L_isoleucine_biosynthesis_IV
PWY_5304__superpathway_of_sulfur_oxidation_(Acidianus_ambivalens)
GALACT_GLUCUROCAT_PWY__superpathway_of_hexuronide_and_hexuronate_degradation_uncla
PWY_6147__6_hydroxymethyl_dihydropterin_diphosphate_biosynthesis_I_g__Collinsella_s__Colli
PWY_6121__5_aminoimidazole_ribonucleotide_biosynthesis_I_g__Collinsella_s__Collinsella_aero
PWY_6122__5_aminoimidazole_ribonucleotide_biosynthesis_II_g__Collinsella_s__Collinsella_aero
PWY_6277__superpathway_of_5_aminoimidazole_ribonucleotide_biosynthesis_g__Collinsella_s__
PWY_7242__D_fructuronate_degradation_unclassified
PWY_6305__putrescine_biosynthesis_IV_unclassified
GLUCUROCAT_PWY__superpathway_of_&beta__D_glucuronide_and_D_glucuronate_degradation_

PWY_621__sucrose_degradation_III_(sucrose_invertase)_g__Eubacterium_s__Eubacterium_rectal
PWY_6507__4_deoxy_L_threo_hex_4_enopyranuronate_degradation_unclassified
P108_PWY__pyruvate_fermentation_to_propanoate_I

Vanderbilt-patient_Post-FMT1	UNC-donor_Donor	UNC-patient_Pre-FMT
0.000042	0.000000157	0.000118668
0.0000335	9.24E-08	0.000103054
0.0000393	0.000000133	0.00011325
0.0000393	0.000000133	0.00011325
0.0000357	0.000000142	0.00011139
0.0000323	8.93E-08	0.0000844
0.0000366	0.000000152	0.000107571
0.0000368	0.000000119	0.000101853
0.000127998	0.000000458	0.000173713
0.0000363	0.000000162	0.000108013
0.0000345	0.000000143	0.000100397
0.0000378	0.000000172	0.0000823
0.0000313	0.000000172	0.0000919
0.0000365	0.000000163	0.000103687
0.000142484	0.000000602	0.000191611
0.0263132	0.000127845	0.0641757
0.0000409	0.000000219	0.00012425
0.000170954	0.00000104	0.000242941
0.000141437	0.00000062	0.00019046
0.000174824	0.000000856	0.000242033
0.0000361	0.00000018	0.000103203
0.000139831	0.000000626	0.000189723
0.000139837	0.000000626	0.000189893
0.0000497	0.000000254	0.000147517
0.0000343	0.000000192	0.0000957
0.0000422	0.000000224	0.000122176
0.000127368	0.000000621	0.000191745
0.000127368	0.000000621	0.000191745
0.0000324	0.00000021	0.0000945
0.0000344	0.000000196	0.0000967
0.0000437	0.000000239	0.00012748
0.0000418	0.000000284	0.000129199
0.0000418	0.000000284	0.000129199
0.0000347	0.000000209	0.0000981
0.0000339	0.000000174	0.0000876
0.000132679	0.000000666	0.00018458
0.0000433	0.000000253	0.00010629
0.0000409	0.000000246	0.000122776
0.0000409	0.000000246	0.000122776
0.0000353	0.0000002	0.000100711
0.0000353	0.0000002	0.000100711
0.000143964	0.000000944	0.000197398
0.00000201	0.000000151	0.000000191
0.00000656	0.000000117	0.000000288
0.000116581	0.000000678	0.000173393
0.000152796	0.000000966	0.0002101

	0.00000418	0.000000161	0.000000128
	0.000142358	0.0000008	0.000192559
	0.00000765	0.000000202	0.000000152
	0.00000259	0.000000191	0.000000245
	0.000032	0.000000243	0.0000912
	0.00000196	8.01E-08	8.08E-08
	0.00000559	0.000000211	0.000000201
	0.000144202	0.00000101	0.000195162
	0.00000978	0.000000381	0.000000338
	0.00000467	0.000000223	0.000000221
	0.00000773	0.0000003	0.000000179
	0.000174824	0.00000133	0.000242033
	0.000174824	0.00000133	0.000242033
	0.00000966	0.00000041	0.000000539
	0.0000075	0.000000281	0.000000427
	0.00000657	0.00000025	0.000000281
	0.00000692	0.000000177	0.000000109
	0.00000603	0.000000142	0.000000178
	0.000144202	0.00000116	0.000195162
	0.00000446	0.000000174	0.000000101
	0.00000566	0.000000146	0.000000212
	0.00000495	0.000000128	0.0000157
	0.00000495	0.000000128	0.0000157
	0.00000524	0.000000148	6.02E-08
	9.72E-08	0.000000183	0.000000227
	9.72E-08	0.000000183	0.000000227
	0.00000651	0.000000271	0.000000171
	0.00000681	0.000000167	0.000000144
	0.00000645	0.000000199	0.000000146
	0.0000159	0.000000329	0.0000272
	0.00000485	0.000000164	0.000000169
	0.00000518	0.000000122	0.00000016
	0.00000644	0.000000285	0.000000213
	0.00000665	0.000000208	0.000000177
	0.00000437	0.000000133	0.000000131
	0.00000762	0.000000305	0.000000328
	0.163006	0.00132921	0.230738
	0.00000461	0.00000026	0.000000247
	0.00000367	0.000000173	0.000000238
	0.00000607	0.000000162	0.000000131
	0.00000365	0.000000167	0.000000218
	0.0000036	0.000000112	7.96E-08
	0.00000565	0.000000178	0.000000118
	0.00000529	0.000000211	0.000000199
	0.00000613	0.000000249	0.000000334
	0.00000472	0.000000128	0.000000158
	0.00000472	0.000000128	0.000000158

	0.00000596	0.000000169	0.000000198
	0.00000472	0.000000178	0.000000219
	0.00000118	0.000000316	0.000000235
	0.00000756	0.000000344	0.000000334
	0.00000447	0.000000193	0.000000219
	0.00000407	0.000000198	0.000000257
	0.00000612	0.000000183	0.000000132
	0.00000612	0.000000183	0.000000132
	0.00000612	0.000000183	0.000000132
	0.000006	0.000000244	0.000000212
	0.00000616	0.000000264	0.000000203
	0.0000047	0.000000206	0.000000185
	0.00000876	0.000000327	0.00000025
	0.00000574	0.000000273	0.000000244
	0.00000565	0.000000225	0.000000343
	0.00000432	0.000000207	0.000000262
	0.00000877	0.000000322	0.000000412
	0.000000107	2.67E-08	0
	0.000000137	3.34E-08	0
	0.00000519	0.000000171	0.000000181
	0.00000016	3.86E-08	0
	0.00000838	0.000000322	0.000000186
	0.00000544	0.000000252	0.00000025
	0.00000221	0.000000213	0.000000164
	0.00000545	0.000000213	0.000000184
	0.00000545	0.000000213	0.000000184
	0.00000604	0.000000162	8.96E-08
	0.00000604	0.000000162	8.96E-08
	0.00000604	0.000000162	8.96E-08
	0.00000604	0.000000162	8.96E-08
	0.00000613	0.000000229	0.000000264
	0.0000061	0.000000235	0.000000256
	0.00000917	0.000000349	0.000000359
	0.00000865	0.000000394	0.000000384
	0.00000562	0.000000146	9.78E-08
	0.00000562	0.000000146	9.78E-08
	0.000006	0.000000221	0.000000261
	0.0000343	5.06E-08	0.0000114
	0.0000106	0.000000354	0.000000917
	0.00000475	0.000000194	4.09E-08
	0.00000614	0.000000245	0.000000248
	0.00000587	0.000000254	0.000000183
	0.00000705	0.000000321	0.000000261
	0.00000704	0.000000218	0.000000286
	0.00000528	0.000000363	0.000000358
	0.00000434	0.000000188	0.000000157
	0.00000424	0.000000378	0.0000135

	0.00000424	0.000000378	0.0000135
	0.00000546	0.00000017	0.000000151
	0.00000838	0.00000049	0.000000459
	0.00000392	0.000000253	0.000000335
	0.00000599	0.000000263	0.000000215
	0.00000469	0.000000228	0.000000202
	0.0000193	0.000000991	0.000000868
	0.00000794	0.000000317	0.000000357
	0.0000125	0.000000439	0.000000397
	0.00000597	0.00000018	0.000000156
	0.00000585	0.000000254	0.000000186
	0.0000107	0.000000459	0.000000398
	0.000007	0.000000223	0.000000112
	0.00000915	0.000000341	0.000000171
	0.00000577	0.000000271	0.000000236
	0.00000446	0.000000257	0.00000019
	0.000007	0.000000218	0.000000245
	0.00000804	0.000000288	0.00000013
	0.00000804	0.000000288	0.00000013
	0.00000804	0.000000288	0.00000013
	0.00000804	0.000000288	0.00000013
	0.00000553	0.000000249	0.000000191
	0.00000555	0.000000287	0.000000214
	0.00000852	0.000000316	0.000000267
	0.00000735	0.000000347	0.000000479
	0.0000113	0.000000487	0.0000135
	0.00000693	0.000000252	0.000000097
	0.00000378	0.000000213	0.000000197
	0.00000596	0.000000285	0.000000269
	0.00000623	0.00000015	0.000000105
	0.00000543	0.000000168	0.000000103
	0.00000507	0.000000238	0.0000181
	0.00000454	0.000000251	8.82E-08
	0.00000727	0.000000394	0.000000391
	0.00000429	0.00000021	0.00000026
	0.00000849	0.000000222	0.000000135
	0.0000124	0.000000361	0.000000493
	0.0000125	0.000000358	0.00000069
	0.00000983	0.000000408	0.000000665
	0.00000816	0.000000418	0.0000135
	0.00000924	0.000000369	0.000000274
	0.0000045	0.000000286	0.000000279
	0.00000727	0.000000453	0.000000247
	0.0000108	0.000000443	0.000000843
	0.00000282	4.31E-08	1.56E-08
	0.00000536	0.00000029	0.000000769
	0.00000499	0.000000261	0.000000151

	0.0000038	0.000000272	0.000000144
	0.00000531	0.000000306	0.000000915
	0.00000559	0.000000316	0.000000286
	0.00000321	0.000000187	0.000000224
	0.0000071	0.000000318	0.000000233
	0.00000277	4.54E-08	2.49E-08
	0.0000056	0.000000268	0.000000277
	0.00000816	0.000000412	0.000000321
	0.00000317	5.37E-08	2.92E-08
	0.00000368	0.000000189	0.000000134
	0.00000974	0.000000435	0.000000377
	0.00000974	0.000000435	0.000000364
	0.00000621	0.000000328	0.000000303
	0.00000644	0.000000311	0.000000545
	0.00000485	0.000000238	0.000000268
	0.00000137	0.000000121	0.000000111
	0.00000765	0.000000451	0.000000576
	0.00000504	0.000000207	0.000000419
	0.00000446	0.000000292	0.000000203
	0.00000482	0.000000207	0.000000185
	0.00000857	0.000000436	0.000000414
	0.00000912	0.000000426	0.00000027
	0.00000543	0.000000203	0.000000206
	0.0000186	0.000000907	0.000000708
	0.0000043	0.000000258	0
	0.00000598	0.000000311	0.000000215
	0.00000441	0.000000108	0.000000247
	0.00000446	0.000000227	0.000000125
	0.000003	0.000000148	0.000000221
	0.000003	0.000000148	0.000000221
	0.00000868	0.000000371	0.000000148
	0.00000868	0.000000371	0.000000148
	0.00000455	9.38E-08	5.45E-08
	0.00000626	0.000000112	6.43E-08
	0.00000626	0.000000112	6.43E-08
	0.0000118	0.000000476	0.000000418
	0.0000491	0.000000125	0
	0.00000889	0.000000435	0.000000323
	0.00000577	0.000000237	0.000000154
	0.0000052	0.000000196	0.000000112
	0.0140066	0.000370775	0.187459
	0.00000698	0.000000467	0.000000185
	0.0000106	0.000000271	0.0000196
	0.0000115	0.00000064	0.00000029
	0.000004	0.000000131	6.42E-08
	0.0278502	0.00134754	0.00741683
	0.0000194	0.000000189	0

	0.00000881	0.00000035	0.000000179
	0.00000881	0.00000035	0.000000179
	0.00000769	0.000000375	0.000000275
	0.00000515	0.000000287	5.59E-08
	0.0000073	0.000000331	0.00000018
	0.0000716	0.00000015	9.83E-08
	0.00000728	0.000000353	0.000000379
	0.00000314	0.000000254	0.000000197
	0.0000064	0.000000297	0.000000333
	0.0000576	0.000000147	0.000000191
	0.0000638	0.000000142	5.23E-08
	0.0000443	0.000000119	0.000000066
	0.00004	0.000000537	0.0000266
	0.00000614	0.000000345	0.000000326
	0.0000188	0.000000998	0.0000219
	0.00000648	0.000000347	0.000000307
	0.0000764	0.000000174	0.000000123
	0.00000177	0.000000135	7.99E-08
	0.0000503	0.000000107	5.88E-08
	0.0000193	0.000000193	0
	0.0000507	0.00000014	0
	0.00000521	0.000000183	0.000000104
	0.000133112	0.000000244	0.000000208
	0.00000536	0.000000216	3.46E-08
	0.00000546	0.000000219	0.000000823
	0.0548372	0.000142193	0.0009482
	0.0000784	0.00000013	5.22E-08
	0.000017	9.95E-08	0
	0.0000022	0.000000169	0.000000142
	0.0000185	6.35E-08	0
	0.0000201	7.96E-08	0
	0.000127978	0.000000263	0.000000188
	0.000127978	0.000000263	0.000000188
	0.00000396	0.000000546	0.000149077
	0.0000446	0.000000922	0.0000317
	0.0000222	4.85E-08	0
	0.000107419	0.000000222	0.000000144
	0.0000173	5.24E-08	0
	0.000284305	0.00000435	0.000372259
	0.0000613	0.000000196	5.84E-08
	0.00000472	6.31E-08	0.000000292
	0.0000333	0.000000175	5.92E-08
	0.000129312	0.000000194	0.000000215
	0.000129312	0.000000194	0.000000215
	0.0000114	0.000000858	0.0000301
	0.00000537	0.000000186	0.000000106
	0.00000537	0.000000186	0.000000106

	0.000106889	0.000000221	0.000000179
	0.000000197	3.16E-08	0
	0.0000901	0.000000207	0.000000179
	0.0000169	0.00000088	0.00000081
	0.0000925	0.00000021	0.000000203
	0.00000361	0.000000211	0.000000104
	0.0000771	0.000000212	0.000000154
	0.0000841	0.000000225	0.000000143
	0.0000223	5.23E-08	0
	0.0000691	0.0000002	0.000000171
	0.0000191	0.00000129	0.0000488
	0.000118037	0.000000214	0.000000211
	0.0000207	5.77E-08	0
	0.000127392	0.000000222	0.000000219
	0.00000626	0.000000186	9.59E-08
	0.0000177	8.93E-08	0
	0.000124301	0.000000252	0.000000218
	0.0000399	0.00000295	0.00018801
	0.000019	7.67E-08	0
	0.00000659	0.000000423	0.00000123
	0.00000659	0.000000423	0.00000123
	0.00164758	0.000143905	0.100464
	0.000116859	0.000000313	0.000000197
	0.00000439	0.000000263	0.000000173
	0.0000166	9.06E-08	0
	0.0000413	0.000000235	0.00000102
	0.0000219	0.00000144	0.0000129
	0.0000497	0.000000401	0.00000014
	0.00101787	0.0000597	0.0374877
	0.0000319	0.00000148	0.00000747
	0.00000379	0.000000736	0.0000332
	0.0000227	0.000000126	0
	0.000022	0.00000114	0.0000256
	0.0000247	0.00000137	0.0000335
	0.0000352	0.00000329	0.000105639
	0.0000352	0.00000329	0.000105639
	0.0000185	0.00000212	0.0000343
	0.0000037	0.000000346	0.000000832
	0.0000317	0.00000139	0.00000541
	0.0139074	0.0000728	0
	0.00000811	0.000000263	0.00000155
	0.00000843	0.000000746	0.0000159
	0.00000773	0.000000137	0.000013
	0.0000191	0.000000112	0
	0.00000864	0.00000073	0.0000159
	0.0000122	0.00000176	0.0000353
	0.00000431	0.000000459	0.000000162

	0.0000516	0.00000489	0.000141551
	0.0000516	0.00000489	0.000141551
	0.0000516	0.00000489	0.000141551
	0.00000785	0.000000266	0.00000115
	0.00000785	0.000000266	0.00000115
	0.00023537	0.00000552	0.000219723
	0.0000219	0.000000117	0
	0.0000199	0.000000102	0
	0.000000959	0.000000219	0.00017655
	0.0000031	0.000000323	0.000000631
	0.0000197	0.000000127	0
	0.0000197	0.000000127	0
	0.000153699	0.00000294	0.000179002
	0.00000129	0.000000214	0.000216702
	0.00000104	0.000000169	0.000168162
	0.0000352	0.00000329	0.000105639
	0.0000122	0.000000231	0.0000174
	0.0000503	0.00000325	0.000176942
	0.000000941	0.00000016	0.000147644
	0.0000354	0.00000285	0.0000128
	0.0000116	0.00000102	0.000018
	0.0000116	0.00000102	0.000018
	0.0000116	0.00000102	0.000018
	0.0000116	0.00000102	0.000018
	0.0000237	0.000000184	0
	0.0000214	0.000000141	0
	0.00000114	0.000000219	0.00018125
	0.0000199	0.000000167	0
	0.00000103	0.0000003	0.000176507
	0.000000586	0.000000216	7.72E-08
	0.00000101	0.000000206	0.000176275
	0.0000106	0.000000759	0.0000526
	0.0000211	0.00000163	0.0000381
	0.0000267	0.00000244	0.00000538
	0.0000516	0.00000489	0.000141551
	0.000000927	0.000000216	0.000170083
	0.00000139	0.000000374	0.000247666
	0.00000116	0.000000413	0.000220897
	0.00000113	0.000000449	0.000237565
	0.000000503	0.000000315	0.000155549
	0.000000826	0.000000217	0.000157125
	0.0000603	0.00000492	0.0000735
	0.0000014	0.00000039	0.000243525
	0.000000465	0.000000171	0.000136167
	0.0310167	0.000271427	0
	0.00000152	0.000000501	0.000263849
	0.000000789	0.000000259	0.000139932

	0.000000807	0.00000024	0.000159471
	0.00000287	0.000000578	0
	0.0000233	0.00000238	0.0000261
	0.000000851	0.000000256	0.000168997
	0.00000122	0.000000481	0.000200117
	0.000000778	0.000000229	0.000153235
	0.00000153	0.000000593	0.000242045
	0.000000739	0.000000248	0.000149348
	0.0000729	0.0000067	0.000174911
	0.0000729	0.0000067	0.000174911
	0.000000925	0.000000421	0.000191391
	0.00000124	0.000000459	0.000242108
	0.000000856	0.000000282	0.000176272
	0.000000743	0.000000358	0.000178942
	0.000000371	0	0
	0.000000778	0.000000378	0.000188292
	0.00000127	0.000000441	0.000248398
	0.00000018	0.000000039	0.0000609
	0.000000772	0.00000035	0.000175188
	0.000001	0.000000411	0.000225496
	0.000000972	0.0000004	0.000217908
	0.00000103	0.000000404	0.00022163
	0.00000113	0.00000063	0.00024343
	0.000000832	0.000000334	0.00016322
	0.00000134	0.00000055	0.000241877
	0.000000827	0.00000033	0.000163841
	0.00000103	0.000000428	0.00022163
	0.00000153	0.000000596	0.000255083
	0.000000498	0.000000279	0.000146099
	0.00000827	0.000000122	4.36E-08
	0.00000112	0.000000456	0.000198005
	0.00000153	0.000000573	0.000260547
	0.00000153	0.000000573	0.000260547
	0.00000115	0.000000528	0.000253872
	0.0000433	0.00000502	0.000115434
	0.000000355	0.00000012	0.000114693
	0.000000355	0.00000012	0.000114693
	0.000000502	0.000000307	0.000164497
	0.000000502	0.000000307	0.000164497
	0.000038	0.000000217	0
	0.00000117	0.00000058	0.000257021
	0.00000117	0.00000058	0.000257021
	0.00000141	0.000000582	0.000243669
	0.00000873	0.0000089	0.000103363
	0.0000011	0.000000503	0.00020159
	0.000000647	0.000000317	0.000238012
	0.000000666	0	0

0.000000322	0	0
0.000000385	0	0
0.00000823	0.000000494	0.0000399
0	0.000000297	0.000000164
0.000000376	0	0
0	5.54E-08	0.0000017
0.000221379	0.00000733	0.0000194
0.000000644	0	0
0.00000166	0.00000025	0
0.0000294	0.0000167	0.000235039
0.000419653	0	0.00000077
0.00000764	0.00000232	0.00000482
0.000519781	0	0.00000117
0.000000446	5.22E-08	0
0.000576684	0	0
0.000454264	0	0.000000984
0.00000631	8.24E-08	0.000000087
0.000000515	0.000000059	0
0.0000646	0.00000858	0.000100325
0.000000369	0	0
0.000422308	0	0.000000772
0.00042015	0	0.000000776
0.000000551	0	0
0.0000244	0	0
0.000481725	0	0.000000796
0.000503234	0	0.0000017
0.00000495	8.58E-08	7.75E-08
0.000630229	0	0.00000292
0.000000336	0	0
0.000000336	0	0
0.000402746	0	0.00000091
0.00000645	0.0000131	0.000149314
0.000000592	0	0
0.000000431	0	0
0.00000235	0.000000082	0
0.000000456	0	0
0.0000375	0.00000525	0.0000379
0.000240118	0.0000101	0.0000272
0.000017	0.0000273	0.000000673
0.000192996	0.00000856	0.0000669
0.00000228	0.000000106	7.89E-08
0.312088	0	0.00142448
0.000000309	5.31E-08	0
0	4.06E-08	0.0000114
0.00000799	0.000000188	9.14E-08
0.0000434	0.00000742	0.00017018
0.00000172	0	0

0.000000107	2.45E-08	0
0.0000121	0.0000112	0.0000355
0.0000267	0.00000785	0.000035
0.0000444	0.00000769	0.0000667
0.0000068	0.000000182	9.04E-08
0.000000852	0.000000074	0
0.000166854	0.0000189	0.000247765
0.000166854	0.0000189	0.000247765
0.000166854	0.0000189	0.000247765
0.00185629	0.000039	0
0.0000424	0.00000035	0
0.0000134	0.00000618	0.0000271
0.0000122	0.0000042	0.000000422
0	0.000000238	8.94E-08
0.00000627	0	0.000000158
0.000000702	0	0.0000324
0.0000197	0.0000091	0.0000402
0.0000971	0.0000174	0.000166523
0	0.000000218	7.69E-08
0.0000143	0.00000662	0.0000283
0.0000513	0.000000441	0
0.0000224	0.0000103	0.0000453
0.000000881	0.000000256	0
0.0000017	4.18E-08	0
0.00000158	4.23E-08	0
0.0000072	0.000000122	4.36E-08
0.00000165	3.26E-08	0
0.000000687	0	0.0000255
0.00000427	0.000000158	0.000000117
0.000000698	0	0.0000297
0.0000272	9.64E-08	0
0.00437229	0.000264561	0
0.0000104	0.000000363	0.00000142
0.000000653	0	0.0000288
0.000000653	0	0.0000288
0.0000248	6.97E-08	0
0.00000073	0	0.0000305
0.00000152	7.34E-08	0
0.0000405	0.0000119	0.00000144
0.000000947	0	0.0000287
0.000000842	0	0.0000327
0.000000752	0	0.0000289
0.0000261	0.000000102	0
0.000041	0.00000035	0
0.000000921	0.00000025	0
0.000000921	0.00000025	0
0.000000669	0	0.0000289

0.000024	0.0000113	0.0000349
0.000000539	0	0.0000271
0.00000182	0.000000492	0
0.0000419	0.000000363	0
0.00000166	0.00000026	0
0.0000429	0.000000371	0
0.00000094	0	0.0000308
0.0000688	0.0000115	0.0000824
0.00000191	0.000000536	0
0.000000547	0	0.0000267
0.0000055	0	0
0.000000709	0	0.0000273
0.000000519	0.000000175	0
0.000000873	0.000000256	0
0.000000803	0	0.0000307
0.000012	0.00000181	0.00000226
0.000145629	0.000042	0.000209398
0.000145629	0.000042	0.000209393
0.000015	0.00000915	0.0000288
0.000000456	0	0.0000287
0.000000456	0	0.0000287
0.00000144	0.000000454	0
0.000000886	0	0.0000306
0.000446542	0	0.018293
0.000000667	0	0.0000272
0.0000217	0.0000238	0.0000564
0.0000111	0.000000512	0.00000177
0.000000729	0	0.0000323
0.00000111	0.000000279	0
0.00000105	0.000000241	0
0.00000101	0.000000189	0
0.0000259	0.0000122	0.000045
0.00000142	0	0
0.000000996	0.000000276	0
0.00000212	0.000000546	0
0.000000895	0.000000243	0
0.000000716	0	0.0000323
0.000000855	0.000000255	0
0.00000592	0.00000032	0.000000038
0.000000666	0	0.000027
0.000109906	0.0000199	0.000141551
0.000000826	0.00000026	0
0.0000313	5.47E-08	0
0.00000101	0.000000254	0
0.000000646	0	0.0000275
0.0000305	0.000000059	0
0.00000119	0.000000317	0

0.000000713	0	0.0000257
0.00000189	0.000000452	0
0.00000189	0.000000452	0
0.000018	0.0000114	0.0000348
0.0000272	5.66E-08	0
0.00000294	0.00000185	0.0000387
0.000000923	0	0.000034
0.0000164	0.00000409	0.0000148
0.00000109	0	0.0000408
0.0000238	0.000000105	0
0.0000137	0.000000982	0.0000109
0.00000627	0.000000215	0.000000852
0.00000119	0.00000039	0
0.0000499	0.000036	0.0000414
0.000000574	0	0.0000264
0.000048	0.0000509	0.0000574
0.0000287	0.00000821	0.000016
0.00000438	0.000000309	0.000000196
0.00000114	0.000000404	0
0.00000651	0.000000504	0.000000817
0.000000346	0.000000297	0
0.00000101	0.000000278	0
0.0000118	0.00000495	0.0000305
0.000122493	0.0000239	0.000191754
0.0000461	0.0000501	0.0000553
0.000347609	0.0000783	0.000295301
0.00000676	0.000000204	0.0000217
0.00012507	0.0000662	0.000299957
0.00105717	0.000176736	0.000933803
0.0000196	0.00000947	0.0000274
0.00000587	0.000000207	0.000000155
0.0000296	0.000000122	0
0.0000296	0.000000122	0
0.00000558	0.000000057	0
0.0000128	0.0000133	0.0000243
0.000000156	0.000000184	0
0.0000313	0.000000132	0
0.0000274	0.000000103	0
0.0000269	9.56E-08	0
0.0000235	7.68E-08	0
0.000006	0.000000202	0.000000198
0.0000409	0.0000145	0.000117695
0.00000183	0.000000908	0.00000492
0.00000575	0.000000194	0.000000164
0.000413071	0.000128445	0.000425395
0.0000272	0.000000109	0
0.00000734	0	0

	0.0000168	5.64E-08	0
	0.00000584	0.0000087	0
	0.0000648	0.0000371	0.00000554
	0.0277844	0.000118528	0
	0.0000482	0.0000442	0.0000221
	0.00000604	0.000000123	0.000000118
	0	0.000000221	0.000000173
	0.0000248	0.000000137	0
	0.00000608	0.000000193	0.000000172
	0.0000246	0.000000122	0
	0.0000246	0.000000122	0
	0.00000811	0.000000249	0.000000364
	0.00000811	0.000000249	0.000000364
	0.000145815	0.0000439	0.000170321
	0.0000271	9.29E-08	0
	0.00000661	0.000000311	0.00000015
	0.0000276	0.000000137	0
	0.00000629	0.000000194	0.00000014
	0	0.00000018	0.00000467
	0.00000809	0.00000168	0.0000151
	0.0000291	0.0000125	0.00000969
	0.00000572	0.00000025	6.64E-08
	0.0000028	0.00000153	0
	0.0000226	0.0000294	0.0000363
	0.0000505	0.0000422	0.000116572
	0.0000203	0.000000105	0
	0.0000674	0.0000238	0.000212123
	0.000000451	0.000000227	0.00000149
	0.0000022	0.00000127	0.00000673
	0	3.03E-08	0.000000148
	0	2.45E-08	9.35E-08
	0	2.45E-08	9.35E-08
	0.0000066	0.000000288	0.000000159
	0.0000201	0.0000255	0.0000361
	0.000315709	0.000098	0.00039681
	0.0000259	0.00000015	0
	0.000243777	0.0000962	0.000343805
	0.00000394	0	0
	0.000376289	0.000122218	0.00046627
	0.000106333	0.000118885	0.00011531
	0.00000616	0.000000249	0.000000193
	0.000417765	0.000140259	0.000539597
	0.000000101	0.000000191	0.00000236
	0.000475033	0.000158231	0.000604395
	0.000137138	0.000059	0.0000762
	0.000424462	0.000138303	0.000548168
	0.000297867	0.0000901	0.000435074

0.000297867	0.0000901	0.000435074
0.00000199	0.00000205	0.000000595
0.00000199	0.00000205	0.000000595
0.0000622	0.0000425	0.0000761
0	0.00000021	0.000000142
0.000000161	6.91E-08	0.000000137
0.000770533	0.000168113	0.000558202
0.0000547	0.0000444	0.0000999
0.000026	0.000000225	0
0.0000336	0.0000382	0.0000592
0.0000214	0.0000441	0.00000259
0.0000194	0.0000108	0.00000822
0.000337359	0.000214711	0.000513475
0.0000346	0.0000214	0.00000505
0.00000298	0.0000366	0.0000276
0.000000214	0.000000108	0.000000436
0.000015	0.0000148	0.00002
0.0000416	0.0000973	0.000334668
0.000465518	0.000170838	0.000481681
0.000000259	0.000000112	0.000000227
0.000000259	0.000000112	0.000000227
0.000228748	0.000102848	0.000446619
0.000531927	0.000184768	0.000664413
0.00035258	0.0000646	0.0000766
0.000092	0.0000434	0.000141335
0.000016	0.000000164	0.000000835
0.000383956	0.000200795	0.000389885
0.00000085	0.0000101	0.0000753
0.00042519	0.000205845	0.000599165
0.000391305	0.000154231	0.000395244
0.00000454	0.00000574	0.0000165
0.000645235	0.000238181	0.000779923
0.000000381	0.000000878	0.00000213
0	2.43E-08	7.22E-08
0.000000673	0.00000156	0.00000327
0.000000573	0.000000311	0.000000763
0.00000313	0.00000308	0.00000105
0.000000616	0.000000333	0.00000083
0	0.000000183	0.00000016
0	0.000000183	0.00000016
0.000000735	0.000000396	0.000000958
0.000320516	0.000164624	0.000593285
0	2.65E-08	7.69E-08
0	3.44E-08	0.00000011
0.0000266	0.000000159	0
0	0.0000226	0.0000569
0.000000855	0.00000046	0.0000011

	0.000434515	0.000260522	0.000501325
	0.0000175	0.0000124	0.0000327
	0.000549019	0.000342333	0.000763714
	0.000000975	0.000000523	0.00000125
	0	0.000000022	7.17E-08
	0	2.77E-08	5.94E-08
	0	0.000000042	8.08E-08
	0.0000001	0.000000101	0
	0	5.23E-08	7.96E-08
	0.000373086	0.000185506	0.000462377
	0.00034418	0.0000728	0.0000816
	0	2.88E-08	0.0000001
	0.000000734	0.000000466	0.000000959
	0	0.0000135	0
	0.0000316	0.0000609	0.0000189
	0.000395829	0.000233633	0.000382841
	0.000435813	0.000285069	0.000502284
	0	5.23E-08	0.000000159
	0.0000115	0.0000236	0.0000181
	0.000388458	0.000223194	0.000326865
	0.000000263	0	0.000000449
	0	0.000000296	0
	0.0000448	0.0000822	0.0000278
	0	4.67E-08	9.75E-08
	0.000337359	0.000245142	0.000515207
	0.00025639	0.000218113	0.00049578
	0.0000988	0.0000312	0.0000241
	0.00000466	0.00000231	0.00000258
	0.000122223	0.00012165	0.000428176
	0	0.000000048	9.76E-08
	0	0.00000021	0.000000706
	0.00000235	0.000000544	0.000000765
	0.000000339	9.97E-08	0.000000511
	0	5.14E-08	9.78E-08
	0	0.00000061	0
	0.000000321	0.00000014	0.000000603
	0.0000597	0.0000538	0.00000799
	0.0000185	0.000014	0.0000144
	0	0.00000038	0.000000772
	0	7.83E-08	0.000000627
	0.0000562	0.000092	0.00003
	0	0.000000035	3.45E-08
	0.00113809	0.000625585	0.000936815
	0.000015	0.0000431	0.00000121
	0	4.98E-08	0.000000048
	0.0000222	0.0000176	0.0000489
	0.0000222	0.0000176	0.0000489

	0.0187819	0.0145977	0.015458
	0.0000169	0.000014	0.0000126
	0.000000141	0.000000704	0.000000313
	0.00046237	0.000314205	0.000417695
	0.00000273	0.000000286	0
	0.000125823	0.000168274	0.000510975
	0.00000753	0.00000887	0.00000215
	0.0000432	0.000113891	0.0000376
	0.0000363	0.0000472	0.0000596
	0.0000119	0.0000421	0.0000422
	0.000286031	0.000188335	0.000101081
	0.0000854	0.0000631	0.000098
	0	0.000000269	0
	0.000000113	0.00000403	0.000000154
	0.0000414	0.000078	0.000233718
	0	0	0
	0.0000189	0.0000178	0.0000178
	0.0000189	0.0000178	0.0000178
	0.00000475	0.00000908	0.000000148
	0	0.000000206	0.000000157
	0.00033217	0.000119044	0.000110555
	0	0.000000477	0
	0	0.00000015	0
	0.000000148	0.000000327	0
	0.000479121	0.000460562	0.000667741
	0	0.000000238	0
	0.0000014	0.00000604	0.00000628
	0.000805417	0.000469086	0.000660313
	0	2.85E-08	0
	0.0013689	0.000906902	0.00128861
	0.0000069	0.00000754	0.00000181
	0.719649	0.619134	0.707304
	0	0.000000451	0
	0	3.36E-08	0
	0.000007	0.00000169	0.00000153
	0.000974606	0.000629187	0.000767734
	0.0000705	0.000158703	0.000260276
	0	0	0
	0.0000107	0.0000288	0.000000775
	0	0.0000002	0.000000319
	0	0.000000425	0
	0	0.000000425	0
	0.0000432	0.0000648	0.0000865
	0.00000215	0.00000817	0.00000975
	0	0.000000382	0
	0.000000744	0.0000106	0.0000328
	0.0000232	0.0000913	0.0000255

0	2.85E-08	0
0.000947407	0.000607038	0.00068719
0.0000135	0.0000137	0.0000105
0	0.000000171	0
0	0.000000129	0
0	0.000000196	0
0	0.000000106	0.00000018
0	7.55E-08	0
0	0.000000106	0.000000176
0.0000141	0.0000147	0.0000102
0.0000614	0.000074	0.0000771
0	0.00000032	0
0.0000467	0.0000137	0.0000103
0.0000179	0.00000757	0.00000682
0.000165211	0.000179222	0.000307868
0.00000173	0.00000414	0.00000443
0.000817954	0.000705294	0.000935115
0	9.73E-08	0
0.0000561	0.0000166	0.0000126
0	0.00000226	0
0	0.000000208	0.000000369
0	0.000000526	0.000000325
0	0.000000526	0.000000325
0	0.000000526	0.000000325
0.000809797	0.000548805	0.000640975
0.000809259	0.000692772	0.000754962
0	0.000000263	0
0	0.0000023	0
0.0000242	0.0000127	0.0000234
0	0.00000221	0
0.0000134	0.0000187	0.0000105
0	0.00000233	0
0.0000129	0.000013	0.0000083
0.000497078	0.000359685	0.000374688
0.0000117	0.0000137	0.00000903
0	8.74E-08	0
0.0000127	0.0000136	0.0000072
0.0000136	0.0000137	0.00000757
0.0000136	0.0000137	0.00000757
0	0	0
0.000844915	0.000735836	0.000794358
0.00078939	0.000714654	0.000756751
0	0.000120879	0
0	0.00000269	0
0	0	0
0	0	0
0	0.000278899	0

0	0.00000242	0
0	0.00000242	0
0.0000119	0.0000143	0.00000751
0	0.00000243	0
0	0.00000331	0
0.000773851	0.000312381	0.000153709
0	0.000000391	0
0.0000411	0.000119903	0.000248834
0	0.00000267	0
0.000702189	0.000457394	0.000412092
0	0.00000253	0
0.00000199	0.00000482	0.00000455
0	0.00000259	0
0	0.00000124	0.000000428
0.0000111	0.0000146	0.00000824
0	0.00000305	0
0	5.12E-08	0
0.000000126	0.00000014	0.000000383
0	0.000000183	0
0	0.000000223	0
0	0.0000028	0
0	0.00000271	0
0.000015	0.0000203	0.0000123
0	0.00000269	0
0.0000107	0.0000146	0.00000785
0	6.49E-08	0
0	0.00000284	0
0.00000649	0.00000459	0.00000419
0	0.00000267	0
0	0.00000267	0
0	0	0
0	0	0
0.000286233	0.000371063	0.00051957
0.000584799	0.00063928	0.000659295
0.000584799	0.00063928	0.000659295
0.0000101	0.0000127	0.00000725
0.0000182	0.0000323	0.0000161
0	0.00000322	0
0.0000108	0.0000147	0.00000737
0.0000108	0.0000147	0.00000735
0	0.00226697	0
0	0.00391522	0
0	0.00209086	0
0	0.000190467	0
0	0.00624191	0
0	0.0169219	0
0	0.000725753	0

0	0.00366992	0
0	0.0021318	0
0	0	0
0	0.00187471	0
0	0	0
0	0.00502329	0
0	0.000226613	0
0	0.00284175	0
0	0.00488561	0
0	0.00274171	0
0	0.000906519	0
0	0.00293051	0
0	0.00248231	0
0	0.00166867	0
0	0.00733657	0
0	0.00444967	0
0	0.000533479	0
0	0.00257761	0
0	0.00179263	0
0	0.00259052	0
0	0.0036201	0
0	0.00140614	0
0	0.00217612	0
0	0.00277936	0
0	0.00949905	0
0	0.00947023	0
0	0.00412105	0
0	0.00885475	0
0	0.00493674	0
0	0.00117959	0
0	0.0175038	0
0	0.00286032	0
0	0.00272806	0
0	0.000125907	0
0	0.0000433	0
0	0.00275008	0
0	0.000956509	0
0.000662723	0	0.00293014
0	0.00162847	0
0	0.00161988	0
0	0.000611961	0
0	0.0122044	0
0.00099862	0.0015574	0
0	0.0153212	0
0	0.000851997	0
0	0.00079605	0
0	0.0000953	0

0.00174809	0	0
0	0.00115515	0
0	0.000367648	0
0	0.00073831	0
0	0.00209018	0
0	0.00043649	0
0	0.0022435	0
0	0.000853886	0
0	0.002975	0
0	0	0
0	0.00275952	0
0	0.0024497	0
0	0.00121257	0
0	0.0003027	0
0	0.000132584	0
0	0.000199352	0
0	0.00153863	0
0	0.00168184	0
0	0.00369082	0
0	0.00278855	0
0	0.00549777	0
0	0.000853823	0
0	0.000750284	0
0	0.000957559	0
0	0	0.00167848
0	0.000100005	0
0.00565867	0	0.00240917
0	0	0.000778311
0	0.0000709	0
0	0.000273648	0
0	0.000171195	0
0	0	0
0	0.00082551	0
0	0.000410833	0
0	0.0019538	0
0	0.00353875	0
0	0.00280409	0
0	0.00626886	0
0	0.00028024	0
0	0.00375562	0
0	0	0.000587702
0	0	0
0	0.000645441	0
0	0.000737711	0
0	0	0.0576032
0	0.000973507	0
0	0.000618574	0

0	0.000537345	0
0	0.000812082	0
0	0	0
0	0.00191045	0
0	0.000815602	0
0	0.00041613	0
0	0	0
0.00542572	0	0
0.000753412	0	0
0	0.0000883	0
0.000310288	0	0
0	0.000613186	0
0	0.00123698	0
0	0.0000671	0
0	0.00250648	0
0	0.000837324	0
0	0.000819953	0
0	0.00859277	0
0	0.00174244	0
0	0.00164903	0
0	0.000852202	0
0	0.00021853	0
0	0.00105129	0
0.000538061	0	0
0.00158047	0	0
0	0	0
0	0.000471418	0
0	0.00411886	0
0	0.0000769	0
0	0.00238824	0
0	0.00421208	0
0	0.00109441	0
0	0.000648298	0
0	0.000436926	0
0	0.00871396	0
0	0.000804642	0
0	0.000578805	0
0	0	0
0	0.0000823	0
0	0	0
0	0	0
0	0.00217604	0
0	0	0
0.0000957	0	0
0	0.00000572	0
0	0.0000014	0
0	7.28E-08	0

0	0.000000989	0
0	0.000000435	0
0	0.000000793	0
0	0.000000462	0
0	0.00000123	0
0	0.000000771	0
0	0.00000233	0
0	0.00000149	0
0	0.000000756	0
0	0.000000727	0
0	0.000000753	0
0	0.00000897	0
0	0.00000359	0
0	0.000000843	0
0	0.0000125	0
0	0.000000838	0
0.00000108	0	0
0	0	0.000014
0	0.000000143	0
0	0.00000091	0
0	0.000000353	0
0	0.000000155	0
0	0.00000561	0
0	0.000000825	0
0	0.00000065	0
0	5.73E-08	0
0	0.00000102	0
0.00000186	0	0.000000382
0	0	0
0	0.000000171	0
0	0.000000602	0
0	0.00000811	0
0	0.00000219	0
0	0.00000145	0
0	0.000000941	0
0	0.00000117	0
0	0.00000144	0
0	0.00000152	0
0.000000491	0	0
0	0	0.000001
0	0	0.0000117
0	0.000000209	0
0	0.0000011	0
0.00000111	0	0
0.0000022	0	0
0	0.00000018	0
0	0	0

0	0	0
0	0.0000018	0
0	7.78E-08	0
0	0.000000533	0
0	0.000000866	0
0	0.00000119	0
0	0.000000957	0
0	0.00000256	0
0	6.13E-08	0
0	0.000000744	0
0	0.00000128	0
0	0.000000181	0
0	0.00000147	0
0	0.0000019	0
0	0.00000644	0
0	0.00000416	0
0	0.0000005	0
0	0.0000123	0
0	0.000000273	0
0	0	0.000000199
0	0.00000104	0
0.00000127	0	0
0	0.000000927	0
0	0.00000386	0
0	0	0.000000265
0	0.000000221	0
0	5.34E-08	0
0	0.00000119	0
0	0.000000625	0
0	0	0
0.000000159	0	0
0	0.00000103	0
0	0.000000963	0
0	0.00000303	0
0	0.000000194	0
0	5.58E-08	0
0	0.00000124	0
0	0.000000584	0
0	0.000006	0
0	0.00000252	0
0	0.00000185	0
0	0.00000221	0
0	0	0
0	0.000000168	0
0	0.0000133	0
0.000000221	0.000000172	0
0	0.0000128	0

0	0.00000112	0
0	0.00000019	0
0.00000123	0	0
0	5.35E-08	0
0	0.000000162	0
0	5.37E-08	0
0	0.000000172	0
0	0.000000162	0
0	0.00000143	0
0	0.00000287	0
0	5.61E-08	0
0	0.00000345	0
0	0.0000112	0
0	0.000000416	0
0	0.00000305	0
0	0.00000659	0
0	0.000000114	0
0	0	0.000014
0	0.000000269	0
0	0.000000209	0
0	0.000000231	0
0	0.00000239	0
0	0.000000189	0
0	0.00000127	0
0	0	0
0	0.000000676	0
0	0.000000125	0
0	0.00000162	0
0	0.00000159	0
0	0.000000597	0
0	0.000000529	0
0	0.000000787	0
0	0.00000264	0
0	0.00000138	0
0	0.000000831	0
0	0.000000623	0
0	0.00000127	0
0	0.00000165	0
0	0.000000855	0
0	0.000000675	0
0.00000147	0	0
0	0	0.00000015
0.00000142	0	0
0	0.00000163	0
0	5.52E-08	0
0	0	0
0.000000985	0	0

0	0	0
0	0	0.000016
0	0.00000654	0
0	0	0
0	0.0000125	0
0	0.0000127	0
0	4.14E-08	0
0	0.00000144	0
0	0.00000104	0
0	0.00000153	0
0	0.00000423	0
0	0.00000622	0
0	0	0
0	0.000000641	0
0	0.00000148	0
0	0.000000143	0
0	0.0000117	0
0	5.82E-08	0
0	0	0
0	0	0
0	0.000000567	0
0	0.0000029	0
0	0.000000846	0
0.00000157	0	0
0	0.000000243	0
0	0.00000241	0
0	0.00000137	0
0	0	0.000000124
0	0	0.000000648
0	0	0.0000301
0	0.00000345	0
0.000000134	0	0
0	0.00000095	0
0	0	0
0	0	0.000000335
0	0.00000233	0
0	0	0
0	0.00000482	0
0	0.00000678	0
0	0.00000233	0
0	0.000000179	0
0	0.000026	0
0	0	0
0	0.00000141	0
0	0	0
0	0.00000159	0
0	0.000000739	0

0	0.000000637	0
0	0.00000146	0
0	0.000000548	0
0	0.000000717	0
0	0.00000094	0
0	0.00000249	0
0	0.000000103	0
0	0.00000148	0
0	0.000000783	0
0	0.000000807	0
0	0.00000125	0
0	0.000000785	0
0	0.00000131	0
0	0.000000531	0
0	0.00000493	0
0	0.000000353	0
0	0.00000514	0
0	0.000000465	0
0	0.00000849	0
0	0.00000071	0
0	0.0000114	0
0	0.000000985	0
0	0.00000247	0
0	0.000000227	0
0	0.00000449	0
0	0.00000106	0
0.00000105	0	0.00000465
0	0.0000029	0
0	6.33E-08	0
0	0.0000106	0
0	0.000000237	0
0	0.00000841	0
0	0.000000105	0
0.00000162	0	0
0	0.00000011	0
0	3.99E-08	0
0	3.19E-08	0
0	0.000000145	0
0	0	0
0	0.00000142	0
0	0	0
0	0.000000994	0
0	0.000000388	0
0	5.42E-08	0
0	0.000000161	0
0	0.000000818	0
0	0.0000007	0

0	0.00000371	0
0	0.00000316	0
0	0.000000613	0
0	0.000000628	0
0	0.000000725	0
0.00000302	0	0.000000551
0	0	5.06E-08
0	0.00000031	0
0	8.79E-08	0
0	0	0
0	0.000000289	0
0	0.000000386	0
0	0.00000317	0
0	0.00000615	0
0	0.000000102	0
0	0.000000198	0
0	0	0.000000935
0	0	0
0	0.00000101	0
0	0.000000176	0
0	0	0.0000126
0	0.000000132	0
0	0.000000116	0
0	0.000000276	0
0	7.39E-08	0
0	0	0
0	0.00000214	0
0	0.000000181	0
0	0.00000027	0
0	0	0
0.000000383	0	0
0	0.000000443	0
0	0.00000139	0
0	4.54E-08	0
0	0.000000686	0
0	0.0000004	0
0	0.000000309	0
0	0.00000461	0
0	0.000000624	0
0	0.00000108	0
0	0.000000152	0
0	0.000000635	0
0.000000794	0	0
0.00000191	0	0
0	0	0
0	0.000000566	0
0	0.00000146	0

0	0.0000012	0
0	0	0
0	0.000000162	0
0	0.00000115	0
0	0.00000486	0
0	0.000000401	0
0	0.000000104	0
0	0	0
0	0.0000011	0
0	0	0
0.000000113	0	0
0	0.00000717	0
0	0	0
0	0.00000136	0
0	0	0
0	0.00000235	0
0	0.00000146	0
0	0.0000011	0
0	0.00000164	0
0	0.00000147	0
0	0.00000102	0
0.0000006	0	0.00000465
0.00000191	0	0
0	0.00000011	0
0	0.000000962	0
0	0.000000684	0
0	0.000000502	0
0	0.000000589	0
0	0.00000071	0
0	0	3.45E-08
0	0.000000315	0
0	0.000000185	0
0	0.00000252	0
0	0.00000135	0
0	0.000000102	0
0.000000794	0	0
0.00000183	0	0
0	0.000000522	0
0	0.00000006	0
0	0	0
0	0	0
4.36E-08	0	0
0	0.00000822	0
0	0.000000773	0
0.00000095	0	0
0	0.00000144	0
0	0.000000317	0

0	0.00000143	0
0	0.00000802	0
0	0.00000648	0
0	0.00000236	0
0	0.000000185	0
0	0.0000135	0
0.000000237	0.000000189	0
0	0.0000133	0
0	0.000000202	0
0.000000867	0	0
0	5.61E-08	0
0	0.000000167	0
0	5.83E-08	0
0	0.000000178	0
0	0.000000173	0
0	0.00000153	0
0	0.00000293	0
0	5.89E-08	0
0	0.00000311	0
0	0	0.0000147
0	0.000000263	0
0	0.000000231	0
0	0.000000222	0
0	0.00000243	0
0	0.000000199	0
0	0.000000651	0
0	0.000000135	0
0	0.00000162	0
0	0.000000633	0
0	4.95E-08	0
0.00000121	0	0
0	0	0.000000347
0	0	0.0000139
0	6.49E-08	0
0	0.0000092	0
0	0.00000267	0
0	0.000000687	0
9.83E-08	0.000000437	0
0	0.00000508	0
0	0.00000249	0
0	0.00000156	0
0	0.00000191	0
0	0.00000233	0
0	0.00000144	0
0.000000697	0	0.00000437
0	0.0000124	0
0	0.0000126	0

0	0.00000172	0
0	0.000000173	0
0	0.000000115	0
0	0.000000902	0
0	0.00000216	0
0	0.000000648	0
0	7.78E-08	0
0	0.00000108	0
0	0.00000109	0
0	0	0.000000153
0	0.000000355	0
0	0	0.000000835
0	0.00000209	0
0	0.00000203	0
0	5.14E-08	0
0	9.65E-08	0
0	0.00000125	0
0	0.00000045	0
0	0.0000151	0
0	0.000000119	0
0.00000155	0	0
0	0	0
0	0	0
0.0000011	0	0
0	0.000000259	0
0	0.000000459	0
0.000000964	0	0
0	0	0.000000239
0	0	0
0	0	0.0000259
0	0.00000017	0
0	0.000000366	0
8.19E-08	0	0
0	0	0.0000148
0	0	0
0.000000893	0	0
0	0	0
0.00000112	0	0
0.00000111	0	0
0	0	0.0000147
0.000000727	0	0
0	0	0.000000272
0	0	0.000000118
0	0.00000645	0
0.00000142	0	0
0.00000103	0	0
0	0.00000114	0

0	0	0
0.00000142	0	0
0	0	0.000000209
0.000000727	0	0
0	0	0.000000272
0	0	0.000000118
0.00000121	0	0
0	0	0.000000172
0	0	0.00000878
0	0	0
0	0	0.000000219
0	0.00000096	0
0.00000123	0	0
0	0.00000113	0
0	0.00000245	0
0	0	0.0000208
0	3.93E-08	0
0	0.000000717	0
0	0.000000561	0
0	0	0
0.000000134	0	0
0	0	0
0	0.00000378	0
0	0.000015	0
0	0.0000131	0
0.000000952	0	0
0	0.00000359	0
0	0.00000104	0
0	0.00000137	0
0	0.00000736	0
0	0	0.000000406
0	0.000000743	0
0	0.00000618	0
0	0	0.000000832
0	0	0.0000113
0	0.00000271	0
0	0	0
0	0.00000964	0
0	0	0
0.000000831	0	0
0.00000108	0	0
0.000000994	0	0
0	0.000000148	0
0.00000139	0	0
0	0	0.000000302
0	0	0.0000153
0.000000245	0	0

0.00000162	0	0
0	0	0
0	0	0.0000287
0	0	0
0.000000235	0	0
0	0	0.0000147
0.00000115	0	0
0	0	0.000000293
0	0.000000134	0
0	0	0
0	0	0.00000886
0	0	0.000000325
0.00000102	0	0
0	0.00000731	0
0.000000981	0	0
0	2.46E-08	0
0	0	0.000000229
0	0	0.00000079
0	0	0.0000127
0	4.65E-08	0
0	0	0
0	0.00000178	0
0	0.000000467	0
0	0.000000705	0
0	0.000000674	0
0	0.00000331	0
0.00000054	0	0.00000409
0	0.00000218	0
0	0.00000118	0
0.000000275	0	0
0	0.00000157	0
0	0.000000223	0
0	0.00000495	0
0	0	0.00000888
0	0.00000185	0
0	5.27E-08	0
0	0	0
0	0	0
0	0.000000119	0
0	0.00000103	0
0	0.000000112	0
0	0.000000398	0
0	0	0
0	0	0
0	0.00000266	0
0	0.00000234	0
0	0.000000503	0

	0	0	0.000000217
	0	0	0.0000132
	0	0.00000218	0
	0	0	0
	0	0	0
	0	0.0000069	0
	0	0	0
	0	0.0000151	0
	0	0.0000133	0
	0	0.00000013	0
	0	0.00000151	0
	0	0.00000112	0
	0	0.00000188	0
	0	0.00000441	0
	0	0.00000656	0
	0	0	0
	0	0.000000653	0
	0	0.00000161	0
	0	0.000000163	0
	0	0.0000145	0
	0	8.24E-08	0
	0	0	0
	0	0	0
	0	0.000000587	0
	0.000000915	0	0
	0.000000848	0	0
	0	0.000000202	0
	0.000000143	0	0.000000924
	0	0	0
	0	0	0
	0	1.93E-08	0
	0	0.000000181	0
	0	0	0
	0	7.72E-08	0
	0	0	0
	0.000000133	0	0
	0	0.0000023	0
	0	0.00000202	0
	0	0	0.000000265
	0	0	0.0000169
	0	0.00000258	0
	0	0.000000148	0
	0	0	0
	0	0.0000023	0
	0	0.00000207	0
	0	0	0.000000326
	0	0	0.0000172

0	0.00000242	0
0	0.000000142	0
0	0	0
0.00000122	0	0
0	0	0.000000227
0	0	0.0000215
0	0	0
0.000000174	0	0.000000102
0	0.00000115	0
0.0000012	0	0
0	0	0.0000133
0	0.000000072	0
0	0.00000108	0
0	0	0.00000048
0	0.00000447	0
0	0.00000596	0
0	0.00000214	0
0	0.000000263	0
0	0.0000254	0
0	0.0000023	0
0	0.0000018	0
0	0	0
0	0.00000147	0
0	0.00000242	0
0	0.000000907	0
0	0.000000916	0
0	0.00000504	0
0	0.00000498	0
0	0.0000012	0
0	0.00000018	0
0.000000684	0	0.00000444
0	0.00000979	0
0	0.000000245	0
0	0.0000113	0
0	0.00000184	0
0	0.00000104	0
0	0.000000324	0
0.00000104	0	0
0	0.000000187	0
0	5.78E-08	0
0	0	0
0	0.00000186	0
0	0.00000237	0
0	0.000000511	0
0	0.000000158	0
0	0.000000246	0
0	0.0000036	0

0	0.000000541	0
0	0.000000579	0
0	0.000000573	0
0	0.000000208	0
0	0	0
0	0.000000906	0
0	0.000000159	0
0	0	0.000000131
0	0.000000186	0
0	0	0.0000124
0	0.000000315	0
0	0.000000322	0
0	0.000000248	0
0	0.000000149	0
0	0.000000217	0
0	0.000000597	0
0	0.00000121	0
0	0.000000471	0
0	0.00000083	0
0	0	0
0	0.000000452	0
0	0.00000014	0
0	0.00000133	0
0	0.00000117	0
0	0.000000457	0
0	0.00000755	0
0	0.00000367	0
0	0.000000691	0
0	0.00000187	0
0.000000229	0.000000297	0
0	0.0000141	0
0.00000152	0	0
0	8.59E-08	0
0	0.000000225	0
0	0.00000228	0
0	0.00000116	0
0	0	0.000000641
0	0.000000351	0
0	5.62E-08	0
0	0.00000351	0
0	0.0000113	0
0	0	0.000000551
0	0	0.0000299
0	0.000000344	0
0	0.0000059	0
0.00000064	0	0
9.48E-08	0	0

	0	0.000000154	0
	0	0.00000121	0
	0	2.87E-08	0
	0	0	0
	0	0.000000431	0
	0	0.000000213	0
	0	0	0
	0	0	0
	0	0.000000131	0
	0	0.000000462	0
	0	0	0
	0.00000143	0	0
	0	9.93E-08	0
	0	0.000000238	0
	0.000000238	0	3.61E-08
	0.000000838	0	0
	0	0	0.000000263
	0	0	0.0000108
	0	0	0.00000148
	0.00000112	0	0
	0	0	0
	0	0	0
	0.000001	0	0
	0	0.00000392	0
	0.00000166	0	0
	0	0.0000039	0
	0	0	0.000000289
	0	0	0.00000298
	0	0.00000455	0
	0	0	0
	0.000000301	0	0
	0	0.00000508	0
	0	0.00000516	0
	0	0.00000185	0
	0	0.000000342	0
	0	0.0000221	0
	0	0.00000154	0
	0	0	0
	0	0.00000224	0
	0	0.00000151	0
	0	7.86E-08	0
	0	0.00000111	0
	0	0.000000988	0
	0	0.00000147	0
	0	0.000000444	0
	0	0.00000094	0
	0	0.00000106	0

	0	0.00000105	0
	0	0.00000233	0
	0	0.00000234	0
	0	0.00000171	0
	0	0.00000072	0
	0	0.000000742	0
	0	0.00000141	0
	0	0.0000009	0
	0	0.00000144	0
	0	0.00000111	0
	0	0.00000653	0
	0	0.00000674	0
	0	0.00000126	0
	0	0.00000894	0
	0	0.00000421	0
	0	0.000000791	0
	0	0.000013	0
	0	0.00000113	0
	0	0.00000261	0
	0	0.0000022	0
	0	7.17E-08	0
	0	0.00000893	0
	0.000000222	0.000000311	0
	0	0.000000815	0
	0	0.000000307	0
	0.00000116	0	0
	0	0.00000185	0
	0	0.00000217	0
	0	0.000000152	0
	0	0.000000955	0
	0	0.00000554	0
	0	0.000000748	0
	0	0.000000808	0
	0	0.000000844	0
	0	0	0.000000268
	0.0000031	0	0
	0	0.000000139	0
	0	0.000004	0
	0	0.000000771	0
	0	0.000000212	0
	0	0	0.0000135
	0	0.000000889	0
	0	0.000000363	0
	0	0.000000543	0
	0	0.00000267	0
	0	0.000000295	0
	0	0.00000117	0

0	3.69E-08	0
0	0.000000657	0
0	0.000000432	0
0	0.000000307	0
0	0.00000454	0
0	0.00000064	0
0	0.000000707	0
0	0.000000456	0
0	0.00000136	0
0	0.000000513	0
0	0.00000131	0
0	6.78E-08	0
0	0.0000066	0
0	0	0.000000114
0	0.00000598	0
0	0	0
0	0.00000178	0
0	0.00000228	0
0	0.00000145	0
0	0.00000084	0
0	0.00000139	0
0	0.00000141	0
0	0.00000108	0
0.00000144	0	0
0	0.00000134	0
0	0.000000595	0
0	0.000000655	0
0	0.000000157	0
0	0.00000245	0
0	0.00000113	0
0	0.000000511	0
0.000000986	0	0
0	0	0.000000293
0	0	0.0000226
0	0.00000123	0
0	0.00000011	0
0	0.00000508	0
0	0.00000252	0
0	0.000000281	0
0	0.00000846	0
0	0.0000266	0
0	0	0
0	0.00000239	0
0	0	0
0	0.000000853	0
0	0.000000861	0
0	0.00000116	0

0	0.00000301	0
0	0.00000206	0
0	0.00000112	0
0	0.000000702	0
0	0.00000118	0
0	0.000000802	0
0	0.00000175	0
0	0.000000795	0
0	0.000007	0
0	0.00000672	0
0	0.000000869	0
0	0.00000863	0
0	0.0000125	0
0	0.000000696	0
0	0.00000293	0
0	0.000000162	0
0	0.00000136	0
0.000000785	0	0.00000448
0	0.00000181	0
0	0.000000108	0
0	0.0000129	0
0.000000259	0.000000203	0
0	0.00000591	0
0	0.00000117	0
0.0000014	0	0
0	0.000000122	0
0	0.00000129	0
0	0	0
0	0.00000116	0
0	0.00000219	0
0	0.00000041	0
0	0.000000143	0
0	0.000000207	0
0	0.000000946	0
0	0.00000343	0
0	0.000000667	0
0	0.000000645	0
0	0.000000834	0
0	0	0.000000235
0	0.000000084	0
0	0.000000248	0
0	2.98E-08	0
0	0	0
0	8.71E-08	0
0	0.00000104	0
0	0.000000146	0
0	0.000000118	0

0	0	0.000000124
0	0.000000228	0
0	0.000000182	0
0	0.000000243	0
0	0.000000246	0
0	0	0
0	0.00000216	0
0	0.0000002	0
0	0.000000248	0
0.00000175	0	0
0.000000514	0	0
0	0.000000203	0
0	0.00000059	0
0	0.0000013	0
0	4.41E-08	0
0	0.000000946	0
0	0.00000022	0
0	0.00000496	0
0	0.000000784	0
0	0.00000105	0
0	0.000000575	0
0	0.000000722	0
0.000000488	0	0
0.00000221	0	0
0	0	0
0	0.000000474	0
0	0.00000156	0
0	0.000000124	0
0	0.000000491	0
0	0.000000104	0
0	0	0
0	0.000000861	0
0	0	0
7.71E-08	0	0
0.00000145	0	0
0	0	0.000000219
0	0	0.0000148
0	0.000000436	0
0.000000096	0	0
0.00000144	0	0
0	0	0.000000383
0	0	0.0000197
0	0	0.0000249
0	0	0.0000192
0	0	0.000000455
0	0	0
0	0	0

	0	0.00000992	0
	0	0.000000746	0
	0	0.00000873	0
	0	0	0
	0	0.00000415	0
	0	0	0
	0	0.00000128	0
	0	0.000000631	0
	0	0.000001	0
	0	0.00000241	0
	0	0.00000145	0
	0	0.000000822	0
	0	0.00000129	0
	0	0.00000179	0
	0	0.00000125	0
	0	0.00000127	0
	0	0.00000149	0
	0	0.00000303	0
	0	0.0000139	0
	0	0.00000179	0
	0.00000145	0	0
	0	9.15E-08	0
	0	0.000000174	0
	0	0	0
	0	0.00000555	0
	0	0.00000131	0
	0	0.000000546	0
	0	0.000000731	0
	0	0.000000791	0
	0	0	5.25E-08
	0	0.000000316	0
	0	0	0.0000138
	0	0.000000424	0
	0	0.00000032	0
	0	0.00000536	0
	0	0.00000134	0
	0.000000797	0	0
	0.00000189	0	0
	0	0	0
	0	0.000000506	0
	0	0.0000158	0
	0	0.000000228	0
	0	0	0
	0	0.00000606	0
	0	0.000000464	0
	0	0.00000744	0
	0	0.000000685	0

0	0.000000469	0
0	0.00000124	0
0	0.000001	0
0	0.00000079	0
0	0.00000059	0
0	0.00000154	0
0	0.00000192	0
0	0.000000898	0
0	0.000000674	0
0.00000131	0	0
0	0.00000118	0
0	5.73E-08	0
0	0.000002	0
0	0.00000177	0
0	0.000000025	0
0	6.95E-08	0
0.00000261	0	0.00000039
0	0.00000021	0
0	0	0
0	0	0
0.000000394	0	0
0	0.000000173	0
0	0.00000158	0
0	0	0
0	0.00000459	0
0	0.00000569	0
0	0.000000449	0
0	0.00000195	0
0	0	0
0	0.0000011	0
0	9.16E-08	0
0	0.00000105	0
0	0.000000705	0
0	0.00000155	0
0	0.000000673	0
0	0.00000078	0
0	0.00000112	0
0	0.000000942	0
0	0.00000165	0
0	0.00000199	0
0	0.000000115	0
0	0.00000127	0
0	0.000000807	0
0	0.000000747	0
0	0.00000165	0
0	0.00000105	0
0	0.00000166	0

0	0.000000908	0
0	0.00000629	0
0	0.00000621	0
0	0.000000735	0
0	0.0000103	0
0	0.00000372	0
0	0.000000882	0
0	0.0000117	0
0	0.000000447	0
0	0.00000303	0
0	4.03E-08	0
0	0.00000171	0
0.000000169	0.000000316	0
0	0.0000113	0
0	0.000000845	0
0	2.34E-08	0
0.00000136	0	0
0	0.000000162	0
0	0.000000118	0
0	0.00000172	0
0	0.000000218	0
0	0.00000112	0
0	0.00000121	0
0	0.00000537	0
0	0.00000314	0
0	0.000000334	0
0	0.000000771	0
0	0.000000922	0
0	6.95E-08	0
0	0.000000114	0
0	0.000000255	0
0	0.000000104	0
0	0.00000471	0
0	0.000000167	0
0	0.0000123	0
0	0	0
0	0.000000906	0
0	0.000000162	0
0	0.000000342	0
0	0.00000212	0
0	0.000000111	0
0	0	0
0.000000418	0	0
0	0.000000164	0
0	0.000000677	0
0	0.00000148	0
0	5.88E-08	0

0	0.000000927	0
0	0.000000497	0
0	0.000000357	0
0	0.0000063	0
0	0.000000733	0
0	0.00000114	0
0	0.000000552	0
0	0.000000156	0
0	0	0
0	0.0000065	0
0	0.000000121	0
0	0.000000505	0
0	0.000000942	0
0	0	0
4.19E-08	0	0
0.00000179	0	0
0	0.00000102	0
0	0.00000135	0
0	0.00000441	0
0	0	0.000000236
0	0.000000173	0
0	0.000000135	0
0	0	0
0.000000409	0	0
0.00000167	0	0.00000326
0	0	0.000000561
0	0.00000624	0
0	0.00000141	0
0	8.06E-08	0
0	0.00000102	0
0	0.000000781	0
0	0.000000444	0
0	0.00000082	0
0	0.000000478	0
0	0.00000131	0
0	0.000000783	0
0	0.00000226	0
0	0.00000163	0
0	0.000000837	0
0	0.000000894	0
0	0.00000122	0
0	0.00000109	0
0	0.00000111	0
0	0.00000126	0
0	0.0000063	0
0	0.00000584	0
0	0.000000536	0

0	0.00000953	0
0	0.00000349	0
0	0.000000942	0
0	0.0000137	0
0	0.00000096	0
0.00000104	0	0
0	0	0.000000215
0.00000328	0	0.000000428
0	0	0.000000185
0	0	0.0000141
0.00000163	0	0
0	0	0
0.000000523	0	0
0	0.000000187	0
0	0.000000846	0
0	0.000000383	0
0	0.000000304	0
0	0.00000579	0
0	0.00000102	0
0	0.000000651	0
0	0	0
0	0	0
0.00000205	0	0
0	0	0.0000223
0	0	0.00000009
0	0.00000625	0
0	0	0
0	0	0
0	0.00000226	0
0	0.00000142	0
0	0.000000732	0
0	0.000000979	0
0	0.00000102	0
0	0.000000901	0
0.00000153	0	0
0	0.000000103	0
0	0.000000553	0
0	0.000000595	0
0	0.000000542	0
0	0.000000185	0
0	0.000000154	0
0.00000144	0	0
0	0.000000176	0
0	4.01E-08	0
0.000000554	0	0
0.00000186	0	0
0	0.000000472	0

0	4.64E-08	0
0	0	0
0	0	0
4.19E-08	0	0
0	0	0.000000395
0	0	0.00000028
0	0	0.000000433
0	0	0.000000328
0	0.00000685	0
0	0.00000111	0
0	0.00000591	0
0	0	0
0	8.44E-08	0
0	0.000000116	0
0	0.000000109	0
0	0	0
0	0.000000226	0
0	0.00000134	0
0	0.00000103	0
0	0.0000012	0
0	0	0.000000238
0	0	0.000000278
0	0.000000189	0
0	9.27E-08	0
0	0.000000946	0
0.00000103	2.34E-08	0
0.000000582	0	0
0	0.00000137	0
0.000000615	0	0
0.00000222	0	0
0	0.000000501	0
0	0.000000859	0
0	0.000000271	0
0	0	0
0	0	0
0	0	0
0	0	0.0000152
0	0	0.000000105
0.00000164	0	0
0	0	0.000000248
0	0	0.0000153
0	0.00000147	0
0	0.00000404	0
0	0.000005	0
0	0.000000306	0
0	0.00000218	0
0	0	0

	0	0.00000112	0
	0	0.00000013	0
	0	0.00000103	0
	0	0.00000148	0
	0	0.000000489	0
	0	0.000000859	0
	0	0.00000113	0
	0	0.00000122	0
	0	0.00000104	0
	0	0.00000203	0
	0	0.0000001	0
	0	0.00000107	0
	0	0.000000825	0
	0	0.000000672	0
	0	0.00000167	0
	0	0.00000108	0
	0	0.00000152	0
	0	0.00000601	0
	0	0.00000595	0
	0	0.00000956	0
	0	0.000003	0
	0	0.000000772	0
	0	0.000012	0
	0	0.000000756	0
	0	0.00000291	0
	0	4.32E-08	0
	0	0.00000168	0
	0.000000194	0.00000033	0
	0	0.0000121	0
	0	0.000000827	0
	0	2.49E-08	0
	0.00000142	0	0
	0	0.000000203	0
	0	0.00000018	0
	0	0.00000163	0
	0	0.000000217	0
	0	0.00000121	0
	0	0.00000121	0
	0	0.00000551	0
	0	0.00000296	0
	0	7.32E-08	0
	0	0.000000114	0
	0	0.000000254	0
	0	0	0
	0	0.00000482	0
	0	0.000000899	0
	0	0.000000145	0

0	0.000000358	0
0	0.00000179	0
0	0	0
0.000000429	0	0
0	0.00000063	0
0	0.00000147	0
0	5.49E-08	0
0	0.000000889	0
0	0.000000401	0
0	0.000000186	0
0	0.00000639	0
0	0.000000626	0
0	0.00000114	0
0	0.000000566	0
0	2.95E-08	0
0	0	0
0	0.000000412	0
0	0.00000651	0
0	0.00000164	0
0	0.000000106	0
0	0.000000498	0
0	0.00000103	0
0	0	0
0	0	0
0	0.00000285	0
0	0.000000149	0
0	0.0000018	0
0	0.00000305	0
0	0.000000454	0
0	0.000000189	0
0	0.0000011	0
0	0.00000139	0
0	0.000000626	0
0	0.000000612	0
0	0.000000561	0
0.00000374	0	0.000000538
0	0	0.000000212
0	0.00000156	0
0	4.08E-08	0
0	0.000000197	0
0	0	0
0	0	0.0000108
0	0.00000293	0
0	0.000000112	0
0	0.00000149	0
0	0	0
0	2.74E-08	0

0	0	0
0	0	0
0	0	0
0	0.00000629	0
0	0	0
0	0.000012	0
0	0.0000126	0
0.00000175	0	0
0	3.32E-08	0
0	0.00000136	0
0	0.00000102	0
0	0.00000136	0
0	0.00000394	0
0	0.00000607	0
0	0	0
0	0.000000629	0
0	0.00000147	0
0	0.000000117	0
0	0.0000108	0
0	5.25E-08	0
0	0	0
0	0	0
0	0.000000549	0
0	0.00000239	0
0	0.00000243	0
0	0.00000167	0
0	0.00000419	0
0	0.00000294	0
0	0.000000162	0
0.000000424	0	0
0.00000134	0	0
0.00000038	0	0.000000286
0	0.000000796	0
0	0.000000149	0
0.00000096	0	0
0	0	0.000000233
0	0	0.0000148
0.000000111	0	0
0	0	0
0	0	0
0	0.00000193	0
0	0	0
0	0	0
0	0	0
0	0.00000534	0
0	0.00000169	0
0	0	0

0	0.00000123	0
0	0.0000082	0
0	0.00000985	0
0	0.0000012	0
0.00000115	0	0
0	0.000000135	0
0	0	0
0	0.00000157	0
0	0.000000597	0
0	0.00000218	0
0	0.00000375	0
0	0.000000852	0
0	0	0.000000257
0	0.000000195	0
0	0	0.000000822
0	0	0.000012
0	0	0
0	0.00000192	0
0	0.000000138	0
0	0.00000104	0
0	0	0
0	0.000000122	0
0	6.07E-08	0
0	0.00000732	0
0	0.00000173	0
0	0	0
0.000000123	0	0
0	0.00000827	0
0	0.00000201	0
0.00000134	0	0
0	0.000000123	0
0	0	0.000000236
0.000000142	0	0
0	0.000000102	0
0	0.00000617	0
0	0	0
0	0.00000219	0
0	0.00000211	0
0	0.00000317	0
0	0.00000425	0
0	0.000000963	0
0	0.00000242	0
0	0.00000215	0
0	0	0.00000025
0	0	0.0000188
0	0.00000237	0
0	0.000000147	0

0	0.000000159	0
0	0	0.0000189
0	0	0.0000172
0.00000108	0	0
0	0	0.000000173
0	0	0.0000103
0	0.000000197	0
0.000000712	0	0
0	0	0
0	0.00000211	0
0	0.00000686	0
0	0.00000687	0
0	0.00000183	0
0	0.00000323	0
0	0.000000742	0
0	0.00000304	0
0	0.00000151	0
0	0.000000757	0
0	0.00000126	0
0	0.00000123	0
0	0.00000107	0
0	0.00000188	0
0	0.00000132	0
0	0.00000605	0
0	0.00000477	0
0	0.00000199	0
0.000000215	0	0
0	0.00000121	0
0	0.000000132	0
0	0.00000379	0
0	0.00000215	0
0	0.000000248	0
0	0.00000191	0
0	0.00000203	0
0	0.00000156	0
0	8.43E-08	0
0	0.00000117	0
0	0.00000128	0
0	0.000000465	0
0	0.000000816	0
0	0.00000123	0
0	0.00000185	0
0	0.00000192	0
0	4.39E-08	0
0	0.00000156	0
0	0.000000615	0
0	0.000000625	0

0	0.00000144	0
0	0.000000732	0
0	0.00000203	0
0	0.0000112	0
0	0.00000384	0
0	0.000000706	0
0	0.00000992	0
0	0.00000132	0
0	0.00000279	0
0	5.33E-08	0
0.000000104	0.000000411	0
0	0.0000024	0
0	0.000000176	0
0.00000153	0	0
0	3.12E-08	0
0	7.56E-08	0
0	6.46E-08	0
0	6.34E-08	0
0	0.000000108	0
0	0.00000074	0
0	0.00000108	0
0	0.00000225	0
0	0.00000017	0
0	0.00000085	0
0	0.0000041	0
0	0.00000326	0
0	0.00000898	0
0	0	0.000000306
0	0.000000182	0
0	0.00000274	0
0	0.00000288	0
0	0.000000786	0
0	0.00000559	0
0	0.000000203	0
0	0.000000967	0
0	0	0.0000176
0	0.00000028	0
0	0.0000002	0
0	0.00000032	0
0	0.000000324	0
0	0	0
0	0.00000235	0
0	5.29E-08	0
0	0.000000363	0
0	0.000000137	0
0	0.000000597	0
0	0.000000142	0

0	0.00000508	0
0	0.000000662	0
0	0.00000036	0
0	0	0
0	0.000000116	0
0	0.00000114	0
0	0.00000127	0
0	0.000000641	0
0	0.00000905	0
0	0	0
0	0.00000056	0
0	0	0
0.000000144	0	0
0	0.00000372	0
0	0.00000626	0
0	0.00000208	0
0	0.000000442	0
0	0.00000826	0
0	0.0000266	0
0	0.00000209	0
0	0.00000206	0
0	0.00000148	0
0	0.000000104	0
0	0.000000978	0
0	0.00000113	0
0	0.000000559	0
0	0.000000771	0
0	0.00000104	0
0	0.00000103	0
0	0.00000236	0
0	0.00000184	0
0	0.000000811	0
0	0.00000101	0
0	0.00000071	0
0	0.00000148	0
0	0.00000889	0
0	0.00000325	0
0	0.000000785	0
0	0.0000101	0
0	0.0000027	0
0	3.53E-08	0
0	0.00000107	0
0.000000749	0	0.00000423
0	0.00000296	0
0	0.00000175	0
0	0.00000958	0
0.000000105	0.000000263	0

0	0.00000298	0
0	0.000000228	0
0.000000965	0	0
0	7.17E-08	0
0	5.84E-08	0
0	0.00000105	0
0	0	0
0	0.00000255	0
0	2.97E-08	0
0	0.000000568	0
0	0.000000199	0
0	0.00000094	0
0	0.000000652	0
0	0.00000332	0
0	0.000000578	0
0	0.000000665	0
0	0.000000852	0
0	0	0.000000217
0	0.000000278	0
0	0.000000028	0
0	0	0
0	0.00000121	0
0	8.31E-08	0
0	0.000000954	0
0	0.00000629	0
0	0.00000013	0
0	0.000000156	0
0	0	0.00000112
0	0	0
0	0.000000297	0
0	0	0.000014
0	0.000000268	0
0	0.000000253	0
0	0	0
0	0.00000175	0
0	0.000000122	0
0.000000445	0	0
0	0.000000147	0
0	0.000000609	0
0	0.000000462	0
0	0.00000106	0
0	0.000000043	0
0	0.000000464	0
0	0.000000437	0
0	0.000000178	0
0	0.00000516	0
0	0.00000055	0

0	0.000000891	0
0.000000884	0	0
0.00000239	0	0
0	0	0
0	0.000000303	0
0	0.0000071	0
0	0.000000119	0
0	0.00000184	0
0	0.000000243	0
0	0.000000583	0
0	0.00000849	0
0	5.67E-08	0
0	0	0
0	0.000000878	0
0	0	0
0.000000084	0	0
0.00000123	0	0
0	0.000000216	0
0	0.00000721	0
0	0.000000144	0
0	0.00000108	0
0	0.000000734	0
0	0.00000122	0
0	0.000000586	0
0	0.000000661	0
0	0.00000107	0
0	0.000000807	0
0	0.0000018	0
0	0.00000105	0
0	0.000000918	0
0	0.00000109	0
0	0.00000132	0
0	0.000001	0
0	0.00000073	0
0	0.0000074	0
0	0.00000729	0
0	0.000000802	0
0	0.0000042	0
0	0.000000817	0
0	0.00000106	0
0	0.00000239	0
0	0.00000222	0
0.00000132	0	0
0	0.000000196	0
0	0.00000143	0
0	0	0.00000035
0	7.17E-08	0

	0	0	0.0000175
	0	0	0
	0	0.00000144	0
	0	0.000000595	0
	0	0.000000372	0
	0	0.00000015	0
	0	0.000000603	0
	0	0.000000743	0
	0	0	0
	0	0.00000162	0
	0	0.000000265	0
	0	0	0
	0.00000133	0	0
	0.00000205	0	0.000000282
	0.000000822	0	0
	0	0	0
	0	0.000000147	0
	0	0.000000147	0
	0	0	0
	0	0	0
	0	0	0
	0.000000957	0	0
	0	0	0.000000244
	0	0	0.0000134
	0	0	0
	0.000000957	0	0
	0	0	0.000000244
	0	0	0.0000134
	0	0	0
	0.000000957	0	0
	0	0	0.000000244
	0	0	0.0000134
	0	0	0
	0	0	0
	0	0	0
	0	0	0
	0	0	0
	0.000000887	0	0
	0.0000011	0	0
	0	0	0.000000262
	0	0.000000123	0
	0	0	0.000000806
	0	0.000000112	0
	0	0	0
	0	0.000000195	0
	0	0.000000389	0
	0.000000435	0	0

	0	0	0.000000199
	0	0	0
	0	0	0.000019
	0	0.000000542	0
	0	0.00000453	0
	0	0.00000722	0
	0	0.00000166	0
	0	0.00000813	0
	0	0.0000255	0
	0	0.0000024	0
	0	0	0
	0	0.00000184	0
	0	0	0
	0	0.00000109	0
	0	0.00000137	0
	0	2.02E-08	0
	0	0.0000102	0
	0.000000143	0.000000302	0
	0	0.0000099	0
	0	0.00000067	0
	0	0.000000874	0
	0	0.000000337	0
	0	4.21E-08	0
	0	6.86E-08	0
	0	2.64E-08	0
	0	9.54E-08	0
	0	0.00000013	0
	0	0.000000131	0
	0	0.0000013	0
	0	0.00000207	0
	0	0.00000243	0
	0	0.000000608	0
	0	0.000000226	0
	0	0.00000123	0
	0	0.00000126	0
	0	0.00000473	0
	0	0.00000356	0
	0	0.000000917	0
	0	0.000000935	0
	0	0.000000971	0
	0	0.000000129	0
	0.00000352	0	0.000000511
	0	0	3.09E-08
	0	7.36E-08	0
	0	0.00000137	0
	0	0.000000121	0
	0	0.00000375	0

0	0.00000108	0
0	0.00000583	0
0	0.00000017	0
0	0	0.000000934
0	0	0
0	0.000000289	0
0	0	0.0000149
0	0.000000259	0
0	0.000000319	0
0	0.000000289	0
0	0.00000157	0
0	0.000000262	0
0.000000602	0	0
0	5.64E-08	0
0	0.000000217	0
0	0.00000055	0
0	0.00000117	0
0	3.09E-08	0
0	0.00000122	0
0.000000959	0	0
0.00000181	0	0
0	0	0
0	0.000000456	0
0	0.000000158	0
0	0.00000164	0
0	0.00000125	0
0	0.000000243	0
0	0.000000193	0
0	0.000000604	0
0	0.00000828	0
0	0.000000546	0
0	0	0
0	0.00000101	0
0	0	0
0.000000086	0	0
0	0.00000535	0
0	0.00000752	0
0	0.00000219	0
0	0.00000804	0
0	0.0000249	0
0	0.00000242	0
0	0	0
0	0.00000171	0
0	0	0
0	0.000000984	0
0	0.000000134	0
0	5.54E-08	0

0	0.00000189	0
0.000000641	0	0.00000418
0	0.00000197	0
0	0.00000129	0
0	1.74E-08	0
0	0.0000104	0
0.000000123	0.000000304	0
0	0.00000994	0
0	0.000000546	0
0	0.000000883	0
0	0.000000357	0
0	4.62E-08	0
0	6.45E-08	0
0	0.000000022	0
0	0.000000131	0
0	0.00000012	0
0	0.000000162	0
0	0.00000132	0
0	0	0
0	0.00000209	0
0	0.00000233	0
0	0.00000062	0
0	0.000000232	0
0	0.00000122	0
0	0.00000179	0
0	0.00000481	0
0	0.00000377	0
0	0.00000104	0
0	0.000000892	0
0	0.000001	0
0	0	0.000000252
0	0.000000115	0
0.00000369	0	0.000000603
0	0	0.000000139
0	0.000000169	0
0	7.73E-08	0
0	0.00000131	0
0	0.000000115	0
0	0.00000365	0
0	0.00000106	0
0	0.00000612	0
0	0.000000178	0
0	0	0.000000875
0	0	0
0	0.00000027	0
0	0	0.0000162
0	0.000000364	0

0	0.000000299	0
0	0.000000308	0
0	0	0
0	0.00000216	0
0	0.000000309	0
0	0	0
0.000000616	0	0
0	5.51E-08	0
0	0.000000628	0
0	0.00000119	0
0	2.62E-08	0
0	0.00000119	0
0.000000867	0	0
0.00000236	0	0
0	0	0
0	0.000000532	0
0	0.000000148	0
0	0.00000159	0
0	0.00000124	0
0	0.0000003	0
0	0.000000298	0
0	0.000000588	0
0	0.0000116	0
0	0.00000063	0
0	0	0
0	0	0
0	0.00000102	0
0	0	0
8.13E-08	0	0
0	0.000000584	0
0	0.000000786	0
0	0.000000446	0
0	0.00000127	0
0	5.74E-08	0
0	0.00000184	0
0	0.000000977	0
0	0.00000132	0
0	0.00000145	0
0	0.00000151	0
0	0.000000844	0
0	0.00000138	0
0.00000114	0	0
0	0	0
0	0.00000102	0
0	0.00000108	0
0	0.00000443	0
0	0	0.000000215

0	0	0.000000176
0	0.00000533	0
0	0	0.0000129
0	0.00000186	0
0	0	0
0	0.000000147	0
0	0.000000368	0
0	0	0
0	0.00000056	0
0	0.000000781	0
0	0.00000113	0
0	7.12E-08	0
0	0.00000188	0
0	0.00000111	0
0	0.00000134	0
0	0.00000139	0
0	0.0000014	0
0	0.000000776	0
0	0.00000146	0
0.00000116	0	0
0	0	0
0	0.00000151	0
0	0.000000896	0
0	0.00000111	0
0	0.00000424	0
0	0	0.000000226
0	0	0.000000131
0	0.00000525	0
0	0	0.000000743
0	0	0.0000124
0	0.00000203	0
0	0	0
0.000000249	0	0
0	0.000000144	0
0	0.000000358	0
0	0	0
0	0	0
0	0.000000675	0
0	0.000000628	0
0	0.00000611	0
0	0.00000628	0
0	0.000000659	0
0	0.00000126	0
0.00000119	0	0
0	0	0
0	0.000000474	0
0	0.000000521	0

0	0.00000066	0
0	0	0.00000914
0	0.00000115	0
0	0	0
0	0	0
0	0	0
0	0	0
0	0.00000179	0
0	0.00000078	0
0	0.000000898	0
0	0.00000628	0
0	0.00000634	0
0	0.00000107	0
0.0000012	0	0
0	3.14E-08	0
0	0.000000202	0
0	0	0
0	0.000000527	0
0	0.00000015	0
0	0.000000554	0
0	0.000000403	0
0	0.00000048	0
0	0.00000102	0
0	0.000000215	0
0	0	0.00000813
0	0.000000102	0
0	0.000000656	0
0	0	0
0	0	0
0	0	0
0	0	0
0.000000234	0	0
0	0.00000209	0
0	0.00000074	0
0	0.000000617	0
0	0.000000825	0
0	0.00000113	0
0	0.000000624	0
0.000000602	0	0
0	0	0
0	0	0
0	0	0
0	0	4.92E-08
0	0.00000343	0
0	0	0
0	0.000000851	0
0	0.00000136	0

0	0.00000107	0
0	0.0000015	0
0	0.00000774	0
0	0.000008	0
0	0.000000605	0
0	0.00000707	0
0	0.00000463	0
0	0.0000138	0
0	0.00000322	0
0	6.58E-08	0
0	0.000000167	0
0.000000428	0	0.00000388
0	0.000000102	0
0	0.0000114	0
0.000000247	0.000000649	0
0	0.0000118	0
0	0.000000201	0
0	4.18E-08	0
0.000000982	0	0
0	0.000000177	0
0	7.92E-08	0
0	0.000000168	0
0	7.28E-08	0
0	0.000000113	0
0	0.000000208	0
0	0.00000118	0
0	0	0
0	0.00000104	0
0	0.00000244	0
0	0.000000285	0
0	0.000001	0
0	0.00000102	0
0	0.00000603	0
0	0.00000395	0
0	0.000000289	0
0	0.000000066	0
0	0.00000125	0
0	6.03E-08	0
0	0.00000372	0
0	0.00000296	0
0	0.000000132	0
0	6.66E-08	0
0	0	0.00000104
0	0	0
0	0	0.0000137
0	0.000000378	0
0	0.00000179	0

0	0.000000324	0
0	0	0
0.000000527	0	0
0.000000197	0	0
0	3.24E-08	0
0.0000202	0	0
0	0.000000425	0
0	0.00000121	0
0	0.000000638	0
0	0	0
0	0.00000745	0
0	8.75E-08	0
0	0.00000242	0
0	0.000000233	0
0	0.000000429	0
0	0.00000708	0
0	0.000000471	0
0	5.89E-08	0
0	0	0
0	0	0
0	0.000000667	0
0	0.00000537	0
0	0.00000278	0
0	0.00000802	0
0	0.00000166	0
0	0.00000215	0
0	0	0
0	0.00000027	0
0	0.00000165	0
0	0.0000139	0
0.000000198	0.000000172	0
0	0.0000125	0
0	0.000001	0
0	0.000000229	0
0.00000109	0	0
0	9.46E-08	0
0	0.00000016	0
0	9.26E-08	0
0	0.000000167	0
0	0.000000159	0
0	0.00000133	0
0	0.00000276	0
0	7.13E-08	0
0	0.000000989	0
0	0.00000341	0
0	0.0000119	0
0	8.58E-08	0

0	0.000000208	0
0	0.000000315	0
0	0.0000031	0
0	0.00000344	0
0	0.00000626	0
0	0.000000104	0
0	0	0.000000704
0	0	0.0000144
0	0.000000312	0
0	0.000000241	0
0	0.000000244	0
0	0	0
0	0.00000262	0
0	5.37E-08	0
0	0.000000162	0
0	0.00000128	0
0	0	0
0	0.000000633	0
0	0.000000126	0
0	0.00000163	0
0	0.00000174	0
0	0.000000134	0
0	0.000000635	0
0	0.000000933	0
0	6.02E-08	0
0	0	0
0	0.00000106	0
0	0.000000496	0
0	0.000000649	0
0	0.000000884	0
0.000000973	0	0
0	0.00000169	0
0	0.00000145	0
0	0	0
0	0.00000218	0
0.00000118	0	0
0	0.0000183	0
0	0.0000101	0
0	0.000000101	0
0	0.00000241	0
0	0.00000253	0
0	0.00000105	0
0	0.0000114	0
0	0	9.18E-08
0	0	0.0000252
0	0.00000423	0
0	0.0000207	0

0	0	0
0	0	0
0	0.000000402	0
0.000000557	0	0
0	0	0.00000209
0	0.000000385	0
0	0.00000535	0
0	0.00000752	0
0	0.00000219	0
0	0.00000804	0
0	0.0000249	0
0	0.00000242	0
0	0	0
0	0.00000171	0
0	0	0
0	0.000000984	0
0	0.000000134	0
0	5.54E-08	0
0	0.00000189	0
0.000000641	0	0.00000418
0	0.00000197	0
0	0.00000129	0
0	1.74E-08	0
0	0.0000104	0
0.000000123	0.000000304	0
0	0.00000994	0
0	0.000000546	0
0	0.000000883	0
0	0.000000357	0
0	4.62E-08	0
0	6.45E-08	0
0	0.000000022	0
0	0.000000131	0
0	0.00000012	0
0	0.000000162	0
0	0.00000132	0
0	0	0
0	0.00000209	0
0	0.00000233	0
0	0.00000062	0
0	0.000000232	0
0	0.00000122	0
0	0.00000179	0
0	0.00000481	0
0	0.00000377	0
0	0.00000104	0
0	0.000000892	0

0	0.000001	0
0	0	0.000000252
0	0.000000115	0
0.00000369	0	0.000000603
0	0	0.000000139
0	0.000000169	0
0	7.73E-08	0
0	0.00000131	0
0	0.000000115	0
0	0.00000365	0
0	0.00000106	0
0	0.00000612	0
0	0.000000178	0
0	0	0.000000875
0	0	0
0	0.00000027	0
0	0	0.0000162
0	0.000000364	0
0	0.000000299	0
0	0.000000308	0
0	0	0
0	0.00000216	0
0	0.000000309	0
0	0	0
0.000000616	0	0
0	5.51E-08	0
0	0.000000628	0
0	0.00000119	0
0	2.62E-08	0
0	0.00000119	0
0.000000867	0	0
0.00000236	0	0
0	0	0
0	0.000000532	0
0	0.000000148	0
0	0.00000159	0
0	0.00000124	0
0	0.0000003	0
0	0.000000298	0
0	0.000000588	0
0	0.0000116	0
0	0.00000063	0
0	0	0
0	0	0
0	0.00000102	0
0	0	0
8.13E-08	0	0

0	0.000000204	0
0	0.000000393	0
0.000000921	0	0
0	0	0.000000216
0	0	0
0	0	0.0000227
0	0	0
0	0.0000151	0
0.00000158	0	0
0	0	0.000000287
0	0.0000102	0
0	0	0.0000117
0	0.00000112	0
0	0.000000728	0
0	0.000000155	0
0	0.000000104	0
0	0.00000348	0
0	0.00000874	0
0	0.0000117	0
0.000000811	0	0
0	0.000000142	0
0	0.00000326	0
0	0.000000112	0
0	0	0.000000088
0	0.0000052	0
0	0	0.00000122
0	0	0
0	0.00000289	0
0	0.00000031	0
0	0.0000118	0
0	0.000000143	0
0	0	0
0	0	0
0	0.000000882	0
0	0.00000297	0
0	0.0000011	0
0	0.00000124	0
0	0.000000721	0
0	0.00000174	0
0	0.00000842	0
0	0.000000609	0
0.000000888	0	0.00000496
0.00000148	0	0
0	0	0
0	0.000000618	0
0	0.00000066	0
0	0.00000077	0

0	0	0.000000244
0	0.00000011	0
0.00000109	0	0
0	0.0000014	0
0	0.000000888	0
0	0.000000739	0
0	0.000000915	0
0	0.000000438	0
0	0	0
6.67E-08	0	0
0	0.00000503	0
0	0.00000245	0
0	0.000000298	0
0	0.00000863	0
0	0.0000269	0
0	0	0
0	0.00000238	0
0	0	0
0	0.000000953	0
0	0.00000122	0
0	0.00000289	0
0	0.0000022	0
0	0.00000116	0
0	0.0000007	0
0	0.00000128	0
0	0.000000849	0
0	0.00000162	0
0	0.00000833	0
0	0.0000131	0
0	0.000000733	0
0	0.00000284	0
0	0.000000171	0
0	0.00000461	0
0	0.00000166	0
0.000000711	0	0.00000429
0	0.00000185	0
0	0.000000107	0
0	0.0000129	0
0.000000263	0.000000203	0
0	0.0000088	0
0	0.00000119	0
0	0.000000296	0
0.00000137	0	0
0	0.000000133	0
0	0.00000134	0
0	0	0
0	0.00000112	0

0	0.0000022	0
0	0.000000456	0
0	0.000000165	0
0	0.000000229	0
0	0.00000103	0
0	0.00000353	0
0	0.000000772	0
0	0.000000761	0
0	0.000000805	0
0.00000397	0	0.00000045
0	0	5.19E-08
0	7.59E-08	0
0	3.45E-08	0
0	0	0
0	8.72E-08	0
0	0.00000298	0
0	0.00000108	0
0	0.000000144	0
0	0.000000112	0
0	0.000000277	0
0	0.00000023	0
0	0.000000302	0
0	0.000000258	0
0	0	0
0	0.0000025	0
0	0.000000208	0
0	0.000000263	0
0.00000192	4.38E-08	0
0.000000544	0	0
0	0.000000235	0
0	0.000000588	0
0	0.00000144	0
0	0.000000037	0
0	0.00000077	0
0	0.000000404	0
0	0.000000217	0
0	0.00000482	0
0	0.000000814	0
0	0.000000909	0
0	0.000000608	0
0	0.000000852	0
0.000000513	0	0
0.00000221	0	0
0	0	0
0	0.000000431	0
0	0.00000154	0
0	0.000000132	0

0	0.000000496	0
0	0.00000011	0
0	0	0
0	0	0
0	0.000000903	0
0	0	0
0.000000087	0	0
0	0.00000508	0
0	0.00000241	0
0	0.000000255	0
0	0.00000853	0
0	0.0000262	0
0	0	0
0	0.00000222	0
0	0	0
0	0.000000863	0
0	0.000000863	0
0	0.00000116	0
0	0.00000282	0
0	0.00000217	0
0	0.00000106	0
0	0.00000067	0
0	0.00000123	0
0	0.000000848	0
0	0.00000161	0
0	0.000000833	0
0	0.00000675	0
0	0.00000642	0
0	0.000000885	0
0	0.00000808	0
0	0.0000128	0
0	0.000000695	0
0	0.00000286	0
0	0.000000159	0
0	3.53E-08	0
0	0.00000458	0
0	0.00000143	0
0.000000819	0	0.00000472
0	0.00000183	0
0	0.000000103	0
0	0.0000124	0
0.000000252	0.000000185	0
0	0.0000052	0
0	0.00000112	0
0	0.000000286	0
0.00000141	0	0
0	0.000000121	0

0	0.00000127	0
0	0	0
0	0.00000114	0
0	0.00000211	0
0	0.000000412	0
0	0.000000134	0
0	0.000000207	0
0	0.000000947	0
0	0.00000352	0
0	0.000000756	0
0	0.000000732	0
0	0.000000834	0
0	0	0.000000217
0	7.74E-08	0
0	0.000000235	0
0	3.15E-08	0
0	0	0
0	0.000000076	0
0	0.0000029	0
0	0.00000108	0
0	0.000000145	0
0	0.000000109	0
0	0	0.000000102
0	0.000000259	0
0	0.000000193	0
0	0.000000238	0
0	0.00000024	0
0	0	0
0	0.00000217	0
0	0.000000204	0
0	0.000000248	0
0.00000182	0.000000042	0
0.000000528	0	0
0	0.000000222	0
0	0.00000057	0
0	0.00000134	0
0	3.77E-08	0
0	0.000000907	0
0	0.000000401	0
0	0.000000211	0
0	0.00000464	0
0	0.000000824	0
0	0.000000981	0
0	0.000000575	0
0	0.000000695	0
0.000000479	0	0
0.00000212	0	0

0	0	0
0	0.000000445	0
0	0.00000154	0
0	0.00000012	0
0	0.000000482	0
0	0.000000098	0
0	0	0
0	0.00000089	0
0	0	0
0.000000076	0	0
0	0	0
0	0.000000278	0
0	0.000000202	0
0	0	0
0.00000117	0	0
0	7.34E-08	0
0	0	0.000000115
0	0	0
0	0.000000242	0
0	0.000000435	0
0.000000971	0	0
0	0	0.000000144
0	0	0
0	0	0.0000209
0	0.0000108	0
0.00000106	0	0
0	0.00000187	0
0	0.00000607	0
0	0.00000764	0
0	0	0
0	0	0
0.000000382	0	0
0	0.00000116	0
0	0.0000144	0
0	0.000000236	0
0	0	0
0	0	0
0	0.00000041	0
0	0.000000496	0
0	0.000000771	0
0	7.01E-08	0
0	0.000000309	0
0	0.0000017	0
0	0.00000271	0
0	0.00000174	0
0	0.00000295	0
0	0.00000137	0

0	0.00000136	0
0	0.0000017	0
0	0.0000017	0
0	0	0
0	0.00000121	0
0	0.000000307	0
0	0.000000543	0
0	0.00000548	0
0	0	0
0	0	0
0	0.000000591	0
0	0.000000749	0
0	0.00000237	0
0	0.00000162	0
0	0.00000115	0
0	0.00000122	0
0	0.00000149	0
0	0.00000146	0
0	0.000000642	0
0	0.00000105	0
0	0.0000131	0
0.000000176	0.000000161	0
0.00000148	0	0
0	0.000000174	0
0	0.000000308	0
0	0	0
0	0.000000676	0
0	0.00000134	0
0	0.000000868	0
0	0.000000733	0
0	0.000000458	0
0	0	0.000000306
0	0.000000322	0
0	0.00000135	0
0	0.000000081	0
0	0.000000263	0
0	0	0.0000113
0	0.000000189	0
0	0.00000141	0
0	0	0
0	0.00000148	0
0.00000151	0	0
0	0.000000487	0
0	0.000000106	0
0	0	0
0	0	0
0	0	0

0.00000122	0	0
0	0	0.0000145
0	0.000000123	0
0.00000064	0	0
0	0	0
0.000000652	0	0
0	0	0.0000145
0	0.000000122	0
0.000000235	0	0
0	0	0.0000147
0	0.00000396	0
0	0.00000624	0
0	0.00000245	0
0	0.00000144	0
0	0.00000104	0
0	0.00000157	0
0	0.00000168	0
0	0.000000389	0
0	0.000000721	0
4.24E-08	0.000000326	0
0	0.000000937	0
0	0.00000244	0
0	0.00000278	0
0	0.00000195	0
0	0.000000762	0
0	0.000000166	0
0	0.00000108	0
0	0.000000973	0
0	0.00000194	0
0	0.00000196	0
0	0.00000635	0
0	0.00000606	0
0	0.00000166	0
0	0.000012	0
0	0.00000394	0
0	0.00000114	0
0	0.00000519	0
0	0.00000048	0
0	0.00000337	0
0	0.00000193	0
0	2.43E-08	0
0	0.00000947	0
0	0.000000582	0
0	0.000000964	0
0	0.000000308	0
0.00000103	0	0
0	0.00000175	0

0	0.00000149	0
0	0.000000371	0
0	0.000000789	0
0	0.00000115	0
0	0.00000583	0
0	0.00000342	0
0	0	0.000000236
0.00000346	0	0.000000515
0	0	0.000000144
0	7.28E-08	0
0	0.00000307	0
0	0	0.0000154
0	0.00000273	0
0.000000797	0	0.0000301
0	0.000000563	0
0	0.00000144	0
0	0.000000048	0
0	0.000000663	0
0	0.000000144	0
0	0.000000975	0
0	0.000000981	0
0	0.000000725	0
0	0.00000135	0
0	0	0
0	0.000000239	0
0	0.00000809	0
0	7.34E-08	0
0	0.00000921	0
0	0	0
0	0	0
0.000000151	0	0
0	0.00000526	0
0	0.00000629	0
0	0.0000139	0
0	0.00000128	0
0	0.00000136	0
0	7.79E-08	0
0	0.00000074	0
0	0.00000129	0
0	0.000000558	0
0	0.00000018	0
0	0.000000769	0
0	0.00000214	0
0	0.00000159	0
0	0.00000112	0
0	0.00000111	0
0	0.000000517	0

0	0.000000955	0
0	0.000000749	0
0	0.00000121	0
0	0.000000634	0
0	0.00000759	0
0	0.00000696	0
0	0.000000616	0
0	0.00000995	0
0	0.00000398	0
0	0.000000961	0
0	0.0000101	0
0	0.000000501	0
0	0.00000213	0
0	0.000002	0
0	0.0000115	0
0	0.000000279	0
0.000000777	0	0
0	0.00000183	0
0	0.0000015	0
0	5.77E-08	0
0	0.000000576	0
0	0.00000159	0
0	0.00000504	0
0	0	0.000000218
0	0.00000436	0
0	0.000000671	0
0	0	0.0000139
0	0.0000013	0
0	0.000000591	0
0	0.00000119	0
0	0.000000886	0
0	0.00000543	0
0	0.000000339	0
0	0.000000481	0
0	0	0
0	9.58E-08	0
0	0.00000528	0
0	0	0
0	0	0
6.43E-08	0	0
0.000000957	0	0
0	0	0.000000244
0	0	0.0000134
0	0	0
0	0.00000653	0
0	0.00000192	0
0	0.00000315	0

0	0.00000394	0
0	0	0
0.00000109	0	0.00000432
0	0.00000889	0
0.000000358	0.00000037	0
0	0.0000153	0
0	0.000000329	0
0	0.000000162	0
0	5.02E-08	0
0	0.000000258	0
0	0.000000103	0
0	9.62E-08	0
0	0.000000221	0
0	8.16E-08	0
0	0.0000026	0
0	0	0
0	0.00000238	0
0	4.95E-08	0
0	0.000000957	0
0	0.00000513	0
0	0.000000746	0
0	0.00000421	0
0	0.00000156	0
0	0.00000728	0
0	0.000000182	0
0	0	0.00000102
0	0.000000319	0
0	0.000000471	0
0	0.000000315	0
0	0	0
0	0.00000391	0
0	6.81E-08	0
0	0.000000261	0
0	0.00000187	0
0	0.00000239	0
0	0.000000157	0
0	0.0000121	0
0	0.000000138	0
0	0	0
0	0.00000151	0
0	0	0
0	0	0
0.00000118	0	0
0	0	0.000000323
0	0	0.0000153
0.000000146	0	0
0	0	0

0	0	8.14E-08
0	0	8.14E-08
0	0	0
0	0	0.0000112
0	0	0.000000347
0	0	0.0000128
0	0	0.00000138
0	2.63E-08	0
0.00000117	0	0
0	0	0.0000242
0	0.000000155	0
0	0	0.0000163
0.00000133	0	0
0	0.000000121	0
0	0.00000161	0
0	0.000000633	0
0	0.00000115	0
0	0.000000285	0
0	0.000000828	0
0	5.72E-08	0
0	0	0.000015
0	0.00000156	0
0	0	0
0	6.89E-08	0
0	0.000000146	0
0	0	3.41E-08
0	0	0
0.00000143	0	0
0.000000392	0	0
0	0	0
0.00000185	0	0
0	0	0
0	0	0.0000276
0	0	0.000000781
0	0	5.77E-08
0	0	5.16E-08
0	0.0000069	0
0	0.000000302	0
0	0.000000707	0
0	0	0
0	0.00000183	0
0	6.01E-08	0
0	0.000000733	0
0	0.000000859	0
0	0.00000119	0
0	0.000000835	0
0	0.00000208	0

	0	3.38E-08	0
	0	0.000000729	0
	0	0.00000065	0
	0	0.00000106	0
	0	0.000000837	0
	0	0.0000046	0
	0	0.00000135	0
	0	0.00000289	0
	0	0.00000153	0
	0	0.000000103	0
	0	0.0000117	0
	9.21E-08	0.000000293	0
	0	0.000013	0
	0	0.00000102	0
	0	0.000000342	0
	0	0.000000086	0
	0.00000198	0	0
	0	7.62E-08	0
	0	0.000000125	0
	0	0.000000363	0
	0	0.000000181	0
	0	6.91E-08	0
	0	6.05E-08	0
	0	0.000000135	0
	0	0.00000133	0
	0	0.00000177	0
	0	0.000000149	0
	0	0.000000255	0
	0	0.00000205	0
	0	0.00000219	0
	0	0.00000398	0
	0	0.0000034	0
	0	0.000000938	0
	0	0.000000855	0
	0	0	0.000000406
	0	0.00000013	0
	0	0.000000515	0
	0	2.76E-08	0
	0	0	0
	0	0.000000177	0
	0	0.00000375	0
	0	0.00000334	0
	0	0.00000125	0
	0	0.0000061	0
	0	0.000000142	0
	0	0.000000114	0
	0	0	0

	0	0	0.0000194
	0	0.000000311	0
	0	0.000000155	0
	0	0.000000332	0
	0	0.000000298	0
	0	0	0
	0	0.000000352	0
	0	0.000000633	0
	0	0.00000161	0
	0	9.49E-08	0
	0	0.000000885	0
	0	0.000000422	0
	0	0.000000221	0
	0	0.00000567	0
	0	0.000000856	0
	0	0.000000691	0
	0	0.000000646	0
	0	4.32E-08	0
	0	0.000000952	0
	0	0	0
	0	0.000000491	0
	0	0.000000186	0
	0	0.000000799	0
	0	0.0000163	0
	0	0.00000216	0
	0	8.24E-08	0
	0	0	0
	0	0	0
	0	0.000000545	0
	0	0	0
	6.46E-08	0	0
	0	0	0
	0	0	0
	0	0.0000013	0
	0.00000133	0	0
	0	0.000000445	0
	0	0	0.00000786
	0	0.000000977	0
	0	0	0
	0	0	0
	0	0	0.00000909
	0	0	0
	0	0.00000397	0
	0	0.00000524	0
	0	0.00000114	0
	0	0.00000115	0
	0.00000101	0	0

0	0.000000454	0
0	0.000000148	0
0	0.00000411	0
0	0.000000609	0
0	0.000000724	0
0	0.000000773	0
0	2.99E-08	0
0	0	0.0000109
0	0	0
0.000000107	0.000000713	0
0	0.000000925	0
0.00000306	0	0
0	0.000000509	0
0	0.000000106	0
0	0.000000464	0
0	0	0
0	0	0
0	0	0
0	0.00000174	0
0	0.00000266	0
0.000000154	0	0
0	0	0.000000103
0.00000436	0	0.0000006
0	0	0.000000284
0	0.000000061	0
0	0	0
0	0.000000334	0
0	0	0.000000583
0	0	0
0	0	0
0	0.00000388	0
0	0.00000688	0
0	0.00000102	0
0	0.0000112	0
0	0.0000044	0
0	0.000000799	0
0.000000975	0	0
0	0	0
0	0.00000057	0
0	0.000000176	0
0	0.00000113	0
0	0.000005	0
0	0.00000363	0
0	0	0.000000279
0	0.000000119	0
0	0.00000521	0
0	0.000000639	0

0	0	0.0000104
0	0	0
0.000000127	0.000000507	0
0.000000124	0	0
0	0.00000116	0
0.0000032	0	0
0	0.000000444	0
0	0.000000071	0
0	0.00000614	0
0	0.000000389	0
0	0	0
0	0	0
0	0	0
0	0.00000046	0
0	0.00000534	0
0	0.00000992	0
0	0.00000174	0
0	0.000000469	0
0	0.00000759	0
0	0.0000252	0
0	0.00000049	0
0	0.0000066	0
0	0.00000265	0
0	0	0
0	0.00000341	0
0	0	0
0	0.00000207	0
0	0.000000123	0
0	0.00000126	0
0	0.000000919	0
0	0.00000179	0
0	0.000000748	0
0	0.000000945	0
0	0.00000177	0
0	0.00000127	0
0	0.00000376	0
0	0.00000267	0
0	7.33E-08	0
0	0.00000261	0
0	0.00000136	0
0	0.00000149	0
0	0.00000164	0
0	0.000000884	0
0	0.00000155	0
0	0.00000117	0
0	0.00000736	0
0	0.0000071	0

0	0.0000015	0
0	0.00000886	0
0	0.00000528	0
0	0.000000829	0
0	0.0000187	0
0	0.00000116	0
0	0.0000032	0
0	0.000000635	0
0	6.96E-08	0
0	0.00000416	0
0	0.00000134	0
0.00000111	0	0.00000514
0	0.00000379	0
0	0.00000246	0
0	0.000000175	0
0	0.0000142	0
0.000000274	0.000000404	0
0	0.0000149	0
0	0.00000111	0
0	0.000000275	0
0	0.000000139	0
0.00000128	0	0
0	0.000000178	0
0	7.78E-08	0
0	0.000000206	0
0	4.37E-08	0
0	0.000000101	0
0	0.000000227	0
0	0.000000167	0
0	0.00000127	0
0	0	0
0	0.00000201	0
0	0.00000263	0
0	5.75E-08	0
0	0.000000719	0
0	0.000000162	0
0	0.000000281	0
0	0.00000127	0
0	0.00000346	0
0	0.00000507	0
0	0.00000541	0
0	0.00000366	0
0	0.00000114	0
0	0.00000077	0
0	0.000000731	0
0	0	0.000000373
0	5.14E-08	0

0.00000528	0	0.000000404
0	0	0.000000346
0	0.000000401	0
0	0.00000032	0
0	8.07E-08	0
0	0	0
0	0.00000186	0
0	8.66E-08	0
0	0.00000432	0
0	0.00000374	0
0	0.00000115	0
0	0.00000723	0
0	0.000000222	0
0	0.000000311	0
0	0	0.00000115
0	0	0
0	0.00000113	0
0	0.000000275	0
0	0	0.0000117
0	0.00000033	0
0	0.000000288	0
0	0.000000282	0
0	0.000000314	0
0	0	0
0	0.00000357	0
0	0.000000239	0
0	0.000000282	0
0	0	0
0.000000474	0	0
0	0	0.000105012
0.000000277	0	0
0	3.64E-08	0
0.000000206	0	0
0	0.000000846	0
0	0.0000012	0
0	0.000000128	0
0	0.000000995	0
0	0.000000423	0
0	0.00000031	0
0	0.00000698	0
0	0.00000104	0
0	0.00000101	0
0	0.000000354	0
0	6.22E-08	0
0	0.00000128	0
0.0000012	0	0
0.00000286	0	0

	0	0	0
	0	0.000000474	0
	0	0.00000774	0
	0	0.000000134	0
	0	0.0000019	0
	0	0.00000123	0
	0	0.000000269	0
	0	0.000000724	0
	0	0.000000787	0
	0	0.0000168	0
	0	0.00000102	0
	0	0.000000883	0
	0	0	0
	0	4.31E-08	0
	0	0	0
	0	0	0
	0	0.000000942	0
	0	0	0
	0.00000028	0	0
	0	0	0
	0	0.00000194	0
	0	0.000000651	0
	0	0.000000979	0
	0	0.00000536	0
	0	0.00000558	0
	0	0.000000976	0
	0	0.00000168	0
	0.00000154	0	0
	0	2.06E-08	0
	0	0.000000257	0
	0	0	0
	0	0.00000052	0
	0	0.000000157	0
	0	0.000000337	0
	0	0.000000412	0
	0	0.000000797	0
	0	0.000000441	0
	0	0.000000366	0
	0	0	0.00000592
	0	0.000000116	0
	0	0.00000149	0
	0	0.000000561	0
	0	0	0
	0	0	0
	0	0	0
	0	0	0
	0.00000036	0	0

0	0	0
0	0.00000467	0
0	0.00000853	0
0	0.00000163	0
0	0.000000191	0
0	0.00000733	0
0	0.0000225	0
0	0.000000643	0
0	0.00000704	0
0	0.00000225	0
0	0	0
0	0	0
0	0.00000194	0
0	0.000000116	0
0	0.000000722	0
0	0.000000754	0
0	0.000000719	0
0	0.00000097	0
0	0.00000079	0
0	0.00000249	0
0	0.00000216	0
0	8.43E-08	0
0	0.00000213	0
0	0.00000102	0
0	0.000000592	0
0	0.00000207	0
0	0.00000115	0
0	0.00000119	0
0	0.000000681	0
0	0.00000731	0
0	0.0000074	0
0	0.000000766	0
0	0.000000897	0
0	0.000000977	0
0	0.00000242	0
0	9.72E-08	0
0	0.000000028	0
0	0.00000355	0
0.00000104	0	0.00000564
0	0.0000025	0
0	0.00000188	0
0	0.000000112	0
0	0.0000124	0
0.000000164	0.000000204	0
0	0.0000103	0
0	0.000000607	0
0	0.00000117	0

0	0.000000287	0
0.00000113	0	0
0	0.000000111	0
0	6.75E-08	0
0	8.98E-08	0
0	3.36E-08	0
0	0.00000016	0
0	0.000000111	0
0	0.00000107	0
0	0	0
0	3.74E-08	0
0	7.18E-08	0
0	0.000000186	0
0	0.00000129	0
0	0.000000969	0
0	0.00000393	0
0	0.000000775	0
0	0.000000664	0
0	0.00000079	0
0	0	0.00000053
0	0.000000126	0
0.0000043	0	0.000000692
0	0	0.000000363
0	0.00000347	0
0	0.00000341	0
0	0.00000109	0
0	0.00000502	0
0	4.75E-08	0
0	0	0
0	0.00000017	0
0	0	0.0000159
0	0.000000202	0
0	0.000000242	0
0	0.000000171	0
0	0.000000261	0
0	0	0
0	0.000000101	0
0	0.000000204	0
0	0	0
0	4.11E-08	0
0	0.000000233	0
0.000000115	0.000000907	0
0.000000114	0	0
0	0.000000552	0
0	0.00000112	0
0	6.62E-08	0
0	0.00000103	0

0	0.000000572	0
0	0.000000284	0
0	0.00000109	0
0	0.00000145	0
0	0.000000492	0
0	0.00000107	0
0.00000048	0	0
0.000003	0	0
0	0	0
0	0.000000523	0
0	0.00000706	0
0	0.000000145	0
0	0.00000123	0
0	0	5.22E-08
0	0.000000146	0
0	0.000000347	0
0	0.00000045	0
0	0.000000669	0
0	0	0
0	8.82E-08	0
0	0	0
0	0	0
0.0000002	0	0
0	0	0
0	0.00000194	0
0	0.000000651	0
0	0.000000979	0
0	0.00000536	0
0	0.00000558	0
0	0.000000976	0
0	0.00000168	0
0.00000154	0	0
0	2.06E-08	0
0	0.000000257	0
0	0	0
0	0.00000052	0
0	0.000000157	0
0	0.000000337	0
0	0.000000412	0
0	0.000000797	0
0	0.000000441	0
0	0.000000366	0
0	0	0.00000592
0	0.000000116	0
0	0.00000149	0
0	0.000000561	0
0	0	0

0	0	0
0	0	0
0	0	0
0.00000036	0	0
0	0	0
0	0.0000071	0
0	0.000002	0
0	0.000000703	0
0	0.000000974	0
0	0.000000761	0
0	0.000000951	0
0	0.000000768	0
0	0.00000665	0
0	0.00000679	0
0	0.000000837	0
0	0.00000127	0
0	0.00000132	0
0.00000128	0	0
0	0	0
0	8.79E-08	0
0	0.00000346	0
0	0.000000589	0
0	0.000000633	0
0	0.000000797	0
0	0	0.000000502
0	0	0.0000144
0	7.84E-08	0
0	0.00000059	0
0	0.000000325	0
0	0.000000171	0
0	0.000000988	0
0	0.00000093	0
0	0.00000122	0
0.00000293	0	0
0	0.000000117	0
0	0.000000614	0
0	0	0
0	0	0
0.000000239	0	0
0	0.00000181	0
0	0.000000814	0
0	0.0000009	0
0	0.0000067	0
0	0.00000672	0
0	0.00000111	0
0.0000012	0	0
0	3.89E-08	0

0	0.000000216	0
0	0	0
0	0.000000546	0
0	0.000000149	0
0	0.000000659	0
0	0.000000419	0
0	0.000000451	0
0	0.00000174	0
0	0.000000253	0
0	0	0.00000928
0	0.000000102	0
0	0.000000684	0
0	0	0
0	0	0
0	0	0
0.000000225	0	0
0.000000989	0	0
0	0.000000962	0
0	0	0.000000147
0	0	0
0.0000179	0	0
0	8.82E-08	0
0	0	0
0	0.0000219	0
0	0	0
0	0.00000514	0
0	0	0.0000184
0	0.00000236	0
0	0.000019	0
0.000000935	0	0
0	0.000000185	0
0	0	0
0.00000153	0	0
0	0.00000109	0
0	0.00000111	0
0.000000406	0	0
0.000000726	0	0.0000276
0	6.31E-08	0
0	0.000000437	0
0	0	4.56E-08
0.000000927	0	0
0	0	0
0	0	0.000000107
0.000000409	0	0
0	0.000000248	0
0	0.00000258	0
0	0.00000121	0

0	0.000000342	0
0	0.00000127	0
0	0.00000101	0
0	0.000000449	0
0.000000858	0	0
8.99E-08	0	0
0.000000399	0	0
0	0	0
0	0.000000034	0
0.00000179	0	0
0	0	0
0	0.00000279	0
0	0.000000992	0
0	0.000000589	0
0	0.000000792	0
0	0.000000497	0
0	0.000000708	0
0.00000156	0	0.00000305
0	0	0.000000524
0	0	0
0	0	0
0	4.01E-08	0
0	0.00000416	0
0.00000106	0	0
0	0.000000249	0
0	0.00000138	0
0.00000405	0	0.000000616
0	0.000000381	0
0	0.000000548	0
0	0.00000492	0
0	0	0.000000563
0	0	0.0000246
0	0.00000188	0
0.000000136	0	0
0.00000336	0	0
0	0.000000527	0
0	0.000000153	0
0	0.00000174	0
0	0.00000158	0
0	0.0000173	0
0	0	0
0	0.0000101	0
0	0.00000169	0
0	0.00000276	0
0	0.000000463	0
0	0.000000592	0
0	0.000000342	0

0	0.000000606	0
0.00000156	0	0
0	0.000000159	0
0	0.000000326	0
0.00000011	0	0
0	0	0
0	0	0.0000173
0	0.000000988	0
0	0.000000608	0
0	0.00000104	0
0	0.000000929	0
0	0.00000396	0
0	0.000000221	0
0	5.34E-08	0
0	0.00000118	0
0	0.000000629	0
0	0	0
0.00000016	0	0
0	0	0
0	0.000000104	0
0.00000145	0	0
0	0	0
0	0.00000236	0
0	0.00000176	0
0	0.00000577	0
0	0.00000509	0
0	0.0000217	0
0	0	0.00000072
0	0	0.000000515
0	0.00000237	0
0	0.000000771	0
0	0.000000585	0
0	0.000000786	0
0	0.00000119	0
0	0.00000057	0
0.000000531	0	0
0.000000512	0	0
0	0	0.00000186
0	0.000000368	0
0	0.00000104	0
0	0.00000066	0
0	0.00000104	0
0	0.00000308	0
0	3.12E-08	0
0	0.00000136	0
0	0.000000798	0
0	0.00000107	0

0	0.00000125	0
0	0.00000126	0
0	0.00000351	0
0	0.000000654	0
0	0.000000815	0
0.00000104	0	0
0	0	0.000000326
0	0	0.0000431
0	0.00000024	0
0	0.00000146	0
0	0.000000232	0
0	0.000000428	0
0.000000929	0	0
0	0	0.000000248
0	0	0
0	0	0.0000242
0	0.000000164	0
0.0000014	0	0
0	5.98E-08	0
0	0	0.000000167
0	0	0.0000163
0	0	0
0	0	0
0.00000112	0	0
0	0	0.000000168
0.00000015	0	0
0	0	0.0000172
0	0	0
0	0.0000104	0
0.000000208	0.000000249	0
0	0.0000118	0
0	0.000000351	0
0	2.61E-08	0
0.00000217	0	0
0	0.00000012	0
0	0.000000159	0
0	0.000000211	0
0	0.000000185	0
0	0.000000135	0
0	0.000000171	0
0	0.000000187	0
0	0.00000162	0
0	0	0
0	0.000000551	0
0	0.000000344	0
0	0.00000117	0
0	0.00000296	0

	0	0	0.000000319
	0	0.000000139	0
	0	0.000000329	0
	0	8.36E-08	0
	0	0	0
	0	0.000000856	0
	0	6.41E-08	0
	0	0.0000035	0
	0	0.00000155	0
	0	0.000000212	0
	0	9.64E-08	0
	0	0.00000026	0
	0	0	0.0000146
	0	0.00000027	0
	0	0.000000233	0
	0	0.000000271	0
	0	0.000000246	0
	0	0.0000014	0
	0	0.000000182	0
	0	0	0
	0.000000357	0	0
	0	0	0
	0	0.00000153	0
	0	0.00000147	0
	0	0.000000155	0
	0	0	0
	0	0	0
	0	0.000000973	0
	0	0	0
	0.000000197	0.000000166	0
	0.00000144	0	0
	0	0.000000147	0
	0	0.000000241	0
	0	0	0
	0	0	0.00000032
	0	0.000000108	0
	0	0	0.000017
	0	0.000000206	0
	0	0	0
	0	0	0
	0	0.00000207	0
	0	0.00000189	0
	0	0.00000217	0
	0	0	0
	0	0.00000215	0
	0	0.000000248	0
	0	0.00000191	0

0	0.00000203	0
0	0.00000156	0
0	8.43E-08	0
0	0.00000117	0
0	0.00000128	0
0	0.000000465	0
0	0.000000816	0
0	0.00000123	0
0	0.00000185	0
0	0.00000192	0
0	4.39E-08	0
0	0.00000156	0
0	0.000000615	0
0	0.000000625	0
0	0.00000144	0
0	0.000000732	0
0	0.00000203	0
0	0.0000112	0
0	0.00000384	0
0	0.000000706	0
0	0.00000992	0
0	0.00000132	0
0	0.00000279	0
0	5.33E-08	0
0.000000104	0.000000411	0
0	0.0000024	0
0	0.000000176	0
0.00000153	0	0
0	3.12E-08	0
0	7.56E-08	0
0	6.46E-08	0
0	6.34E-08	0
0	0.000000108	0
0	0.00000074	0
0	0.00000108	0
0	0.00000225	0
0	0.00000017	0
0	0.00000085	0
0	0.0000041	0
0	0.00000326	0
0	0.00000898	0
0	0	0.000000306
0	0.000000182	0
0	0.00000274	0
0	0.00000288	0
0	0.000000786	0
0	0.00000559	0

0	0.000000203	0
0	0.000000967	0
0	0	0.0000176
0	0.00000028	0
0	0.0000002	0
0	0.00000032	0
0	0.000000324	0
0	0	0
0	0.00000235	0
0	5.29E-08	0
0	0.000000363	0
0	0.000000137	0
0	0.000000597	0
0	0.000000142	0
0	0.00000508	0
0	0.000000662	0
0	0.00000036	0
0	0	0
0	0.000000116	0
0	0.00000114	0
0	0.00000127	0
0	0.000000641	0
0	0.00000905	0
0	0	0
0	0.00000056	0
0	0	0
0.000000144	0	0
0	0	0
0	0	0
0	0.000000887	0
0	0.00000114	0
0	0	0
0	0	0
0	0.00000946	0
0	0.000000572	0
0.00000308	0	0
0	0.000000406	0
0	0.000000694	0
0	0.000000534	0
0	0	0.000000604
0	0	0.0000116
0	0.00000196	0
0	7.83E-08	0
0	8.04E-08	0
0	0.00000098	0
0	0	0
0	0	0

0.000000123	0	0
0.000000934	0	0
0	0	0.0000114
0	3.98E-08	0
0	0.000000106	0
0	0.00000122	0
0.00000127	0	0
0	0	0.0000107
0	0.000000856	0
0	0	0
0	0	0
0.00000431	0.000000035	0
0	0	0
0.0000024	0	0.00000032
0.000000882	0	0
0	0	0
0	0.000000478	0
0	0.000000559	0
0	0.00000283	0
0	0.00000114	0
0	0.000000927	0
0	0.00000117	0
0	0.000000721	0
0	0.00000137	0
0	0.0000111	0
0	0.000000855	0
0	0.00000856	0
0	0.000000419	0
0.000000676	0	0
0	0.000000577	0
0	0.000000203	0
0	0.000000392	0
0.000000886	0	0
0	0	0.000000322
0	0	0.0000223
0	0	0
0	0	0
0	0	0
0	0.00000487	0
0.00000119	0	0
0	0	0.0000104
0.00000119	0	0
0	0	0.0000104
0.0000117	0.000000359	0
5.87E-08	0	0
5.87E-08	0	0
0	0	0

0.00000076	0	0
0	0	0
0	0.00000246	0
0	0.00000162	0
0	0.000000893	0
0	0.000000983	0
0	0.00000125	0
0	0.00000161	0
0	0.000000108	0
0.000000749	0	0
0.00000185	0	0
0	0.000000165	0
0	0	0
0.00000123	0	0
0	0	0
0	0.00000367	0
0	0.0000111	0
0	0.0000117	0
0.000000824	0	0
0	0.00000326	0
0	0.000000831	0
0	0.000000112	0
0	0	0.000000088
0	0.00000109	0
0	0.0000055	0
0	0	0.00000122
0	0	0
0	0.00000272	0
0	0	0
0.000000246	0	0
0	0.00000031	0
0	0.000000125	0
0	0.0000124	0
0	0.000000143	0
0	0	0
0.000000825	0	0
0	0.00000173	0
0	0.000000839	0
0	0.00000747	0
0	0.00000233	0
0.000000888	0	0
0	0.0000023	0
0	0.00000109	0
0	0.00000108	0
0	0.0000033	0
0	0.0000053	0
0	0.00000203	0

0	0.00000108	0
0	4.22E-08	0
0	0.000000158	0
0	0.00000126	0
0	0.000000444	0
0	0.000000486	0
0	0.00000231	0
0	0.000000954	0
0	0.00000111	0
0	0.000000916	0
0	0.000000649	0
0	0.00000142	0
0	0.0000107	0
0	0.00000408	0
0	0.000000804	0
0	0.000009	0
0	0.000000781	0
0.000000627	0	0
0	0.000000594	0
0.00000079	0	0
0	0	0
0	0.000000645	0
0	0.000000826	0
0	0.00000477	0
0	0.000000885	0
0	0.00000101	0
0	0.000000498	0
0	0.00000201	0
0	0.000000652	0
0	0.000000825	0
0	0.000000732	0
0.00000144	0	0
0	0.000000143	0
0.00000398	0	0.000000629
0	4.36E-08	0
0	0	0
0	0	0.0000201
0	0	0
7.89E-08	0	0
0.000000576	0	0
0	0.00000039	0
0	0	0
0	7.13E-08	0
0	0.00000257	0
0	0.000000845	0
0	0.00000244	0
0.00000113	0	0

0	0.00000194	0
0	0.00000115	0
0	0.00000339	0
0	0	0.000000294
0	0.00000505	0
0	0.000000928	0
0	0	0.0000119
0	0.00000186	0
0	0.000000407	0
0	0.00000016	0
0	0.000000654	0
0	0	0
0	0.00000329	0
0.0000014	0	0
0	0	0.000000321
0	0	0.0000205
0	0.00000055	0
0	0.00000129	0
0.00000137	0	0
0	0	0.000000297
0	0	0.000015
0.00000118	0	0
0	0	0.000000281
0.00000123	0	0
0	0	0.000000295
0	0	0
0	0	0
0	0.00000179	0
0	0	0
0	0.00000218	0
0	0.000000957	0
0	0.00000125	0
0	0.000000979	0
0	0.00000142	0
0	0.000000982	0
0	0.000000791	0
0	0.00000174	0
0.00000112	0	0
0	0.00000229	0
0	0	0.0000243
0	0.0000022	0
0	0.000000415	0
0	0.00000226	0
0	0	0
0	4.37E-08	0
0	0.0000011	0
0.000000744	0	0.00000457

0.00000192	0	0
0	0.00000205	0
0	3.44E-08	0
0	0.000000971	0
0	0.00000123	0
0	0.00000488	0
0	0	0.000000289
0	9.39E-08	0
0	0.00000019	0
0	0	0.0000225
0	0.00000233	0
0	0.000000164	0
0.000000644	0	0.0000249
0	9.88E-08	0
0	0.00000039	0
0	0.000000136	0
0	0	3.41E-08
0	0	0
0	0	0
0	0	0
0	0	0
0	0.00000279	0
0	0.000000369	0
0	0.0000004	0
0	0.00000983	0
0	0.00000225	0
0.000000717	0	0
0	0.00000268	0
0	0.00000165	0
0	0.00000625	0
0	7.46E-08	0
0	0.00000124	0
0	5.77E-08	0
0	0.000000384	0
0	0.000000686	0
0	0.0000117	0
0	0	0.0000132
0	0	0
0	5.63E-08	0
0	0.000000861	0
0.000000859	0	0.00000399
0	0.0000029	0
0	0	0
0	0.0000032	0
0	0	0.000000325
0	0	0
0	0	0.000000124

0	0	0.00000103
0	0	0
0.000000693	0	0
0	0.000000179	0
0	0.000000481	0
0	0	0
0	0	0
0	0	0.000000356
0	0	0.000000309
0	0.0000069	0
0	0.000000302	0
0	0.000000707	0
0	0	0
0	0.00000209	0
0	0.00000183	0
0	6.01E-08	0
0	0.000000859	0
0	0.00000119	0
0	0.000000835	0
0	0.00000208	0
0	3.38E-08	0
0	0.000000729	0
0	0.00000065	0
0	0.00000106	0
0	0.000000837	0
0	0.0000046	0
0	0.00000135	0
0	0.00000289	0
0	0.000000283	0
0	0.00000161	0
0	0.00000374	0
0	0.00000153	0
0	0.000000103	0
0	0.0000151	0
0	0.0000133	0
0	0.00000102	0
0	0.000000207	0
0	0.000000086	0
0.00000196	0	0
0	7.62E-08	0
0	8.06E-08	0
0	0.00000013	0
0	0.000000103	0
0	6.91E-08	0
0	6.05E-08	0
0	8.19E-08	0
0	0.00000151	0

	0	0.00000177	0
	0	0.00000226	0
	0	0.000000149	0
	0	0.000000255	0
	0	0.00000112	0
	0	0.00000188	0
	0	0.00000441	0
	0	0.00000334	0
	0	0.000000938	0
	0	0.000000855	0
	0	0	0.000000309
	0	0.00000013	0
	0	0.000000404	0
	0	2.76E-08	0
	0	0	0
	0	6.24E-08	0
	0	0.00000375	0
	0	0.00000334	0
	0	0.00000125	0
	0	0.00000656	0
	0	0.000000142	0
	0	0	0
	0	0	0.0000256
	0	0.000000311	0
	0	0.000000155	0
	0	0.000000298	0
	0	0	0
	0	0.00000277	0
	0	0.000000352	0
	0	0.000000653	0
	0	0.00000161	0
	0	7.77E-08	0
	0	0.000000885	0
	0	0.000000422	0
	0	0.000000221	0
	0	0.00000567	0
	0	0.000000856	0
	0	0.000000691	0
	0	0.000000646	0
	0	4.32E-08	0
	0	0.000000952	0
	0	0	0
	0	0.000000427	0
	0	0.000000163	0
	0	0.00000153	0
	0	0.00000135	0
	0	0.000000799	0

0	0.0000145	0
0	0.00000216	0
0	8.24E-08	0
0	0	0
0	0	0
0	0.000000587	0
0	0	0
6.46E-08	0	0
0	9.77E-08	0
0	6.29E-08	0
0	0.00000274	0
0.0000235	0.0000573	0.0000335
0	6.27E-08	0
0	0.000000297	0
0.000405293	0.000197283	0.0000509
0.00000073	0.00000056	0.000000391
0.00053835	0.000632442	0.000644614
0	0.000000114	0
0.00000032	0.00000274	0.00000104
0	0.00000271	0
0.000000116	0.000000432	0.000000285
0.0000007	0	0
0.000000601	0.00000031	0.000000764
0.000604416	0.000357451	0.000269279
0.000000105	0.000000365	0.000000247
0	0	0.000000442
0	0.000000192	0
0	0.000000192	0
0.000235284	0.000117888	0.0000246
0	0.000000323	0
0	0.000000323	0
0	0.00000303	0
0.00000839	0.0000127	0.00000601
0.000000316	0.00000206	0.00000106
0.0000456	0.00018864	0.0000496
0	7.22E-08	0
0	0.00000285	0
0.0000357	0.0000342	0.000146532
0	7.27E-08	0
0	0.000000695	0.000000536
0	0.00000299	0
0.000149876	0.000162602	0.000192876
0.000000615	0.000000131	0
0	6.15E-08	0
0	0.000000115	0
0.0000394	0.0000543	0.00000373
0.000028	0.0000758	0.0000455

	0	0.0000006	0
	0	0.00397135	0
	0.000000271	0.000000697	0.000000384
	0	0.000000147	0
	0	0.000000253	0
	0	9.18E-08	0
	0	0.000000517	0
	0.000203933	0.000187064	0.000184405
	0	0.000000124	0
	0	7.11E-08	0
	0.000135175	0.000263022	0.000175569
	0.000102202	0.000205065	0.000387471
	0	0.00000127	0
	0	0.000000495	0
	0.00032265	0.0001458	0.0000391
	0.000000276	0.00000134	0.000000831
	0	0	0
	0	0.00000344	0.00000162
	0.0000902	0.000176449	0.00000173
	0	0.000000108	0
	0	0	0
	0	0	0
	0	0	0
	0.000313492	0.000288517	0.000270703
	0.000273534	0.000371063	0.000402343
	0	0.000000123	0
	0	0.000000364	0.000000294
	0.0000096	0.00000959	0.00000112
	0.00000106	0.000000864	0.000000505
	0.000465721	0.000408225	0.000336869
	0	0.000000111	0
	0	0.000000177	0
	0	0.000000116	0
	0	0.000000114	0
	0	0.000000114	0
	0	0.000000587	0
	0	0.00000012	0
	0	0.000000409	0
	0.000136081	0.000298409	0.000336239
	0.000000781	0.00000015	0.000000375
	0.00000525	0.0000237	0.0000191
	0	0	0
	0	0.000000156	0
	0	0.000000389	0.000000272
	0.0000747	0.000216096	0.00027733
	0	0.00000843	0
	0	0	0

0	0	0
0.000352224	0.000443319	0.000501396
0.000582177	0.000680838	0.000530234
0	0.00000147	0.00000135
0	0.0000002	0
0	0	0
0	0.000000146	0
0.000207542	0.000180913	0.0000895
0	0.000000515	0
0	0.00000023	0
0.000368937	0.000480847	0.000504951
0	0.000000122	0
0.00000107	0.00000237	0.00000159
0.000000538	0.000000447	0.000000282
0	0.00000108	0.000000935
0	0.000000125	0
0	0.000000154	0
0	0.000000154	0
0.0000189	0.0000116	0.00000287
0	0.000000193	0
0	0	0
0	0.000000134	0
0	0.000000161	0
0	0.000000683	0
0	0.000000222	0
0.00000217	0.00000531	0.0000341
0	0.00000014	0
0	0.000000145	0
0.000111899	0.000331666	0.000268862
0	0.000000153	0
0	0.000000109	0
0	0.00000014	0
0	0.000000132	0
0	5.47E-08	0
0.000000419	0.00000232	0.000000551
0	0.000000266	0
0	0.00000011	0
0	0.0000022	0
0	0.00000015	0
0.0000818	0.000254861	0.000192694
0.000419282	0.000680838	0.000550733
0.000419282	0.000680838	0.000550733
0	0.000000167	0
0	0.000000123	0.00000021
0	0.000000123	0.00000021
0.0000964	0.000174737	0.000120931
0	9.87E-08	0

0	0.000000168	0
0	0.000000186	0
0	0.000000157	0
0	0.000000141	0
0	0.000000169	0
0	0.000000307	0
0	0.000000103	0
0.00000635	0.0000145	0
0.000000123	0.000000558	0
0	0.000000193	0
0	0.000000736	0
0.000036	0.0000266	0.0000097
0	0.000000111	0
0	0.000000189	0
0	0.000000178	0
0	0.000000313	0
0.0000504	0.000190458	0.0000472
0.00000306	0.00000881	0
0.00000116	0.00000212	0
0.0000237	0.0000822	0.0000447
0.000169561	0.000325943	0.00018311
0.00000249	0.00000696	0
0	0.000000171	0
0.00000726	0.0000192	0
0	0.000000187	0
0	0.0000011	0.0000005
0	0.00000018	0
0	0.00000018	0
0.000114788	0.000237823	0.00021822
0.00000295	0.0000105	0
0.0000036	0.0000116	0.00000158
0	0.000000212	0
0.233267	0.338666	0.242221
0.0000027	0.0000105	0
0	0.00000015	0.000000192
0.000105322	0.0000809	0.00000335
0.00000236	0.00000645	0
0.000224978	0.000258275	0.0000533
0	0.000000274	0
0.000000208	0.000000468	0
0.00000239	0.00000704	0
0.00138671	0.00171447	0
0.0000183	0.0000176	0.00000606
0.00000274	0.00000768	0
0	0.000000277	0
0.00000197	0.00000328	0
0.000137936	0.000332795	0.000160552

	0.0000774	0.000376673	0.000262688
	0.00000272	0.00000788	0
	0.0000363	0.0000105	0.00000194
	0	0.000000104	0
	0.00000257	0.00000764	0
	0.00000346	0.0000128	0
	0	0.00000015	0
	0.00000109	0.0000132	0.00000343
	0	7.09E-08	0
	0.00000263	0.00000815	0
	0.000252968	0.000279961	0.0000613
	0.0000026	0.00000869	0
	0.000000173	0.00000031	0
	0.000337973	0.000411717	0.000286698
	0.00000241	0.00000787	0
	0.0000025	0.00000784	0
	0	6.03E-08	0
	0.00022171	0.000450772	0.000177182
	0.0000289	0.0000372	0.0000192
	0.00000158	0.00000367	0.00000206
	0.00000292	0.00000813	0
	8.85E-08	0.000000371	0
	0.00000208	0.0000123	0
	0.00000261	0.0000104	0
	0.00000325	0.00000875	0
	0.000000264	0.00000125	0.000000708
	0.000224178	0.000265068	0.000053
	0	3.97E-08	0
	0.00000315	0.0000127	0
	0	0.000000266	0
	0.0000042	0.0000327	0.000000231
	0.00000205	0.00000505	0.00000296
	0	0.000000175	0.000000182
	0.00000165	0.00000507	0
	0	0	0
	0.00000211	0.00000993	0
	0	0.000000141	0
	0.000129537	0.00017706	0.0000289
	0.00000277	0.00000885	0
	0.00000248	0.00000722	0
	0.00000248	0.00000722	0
	0.00000226	0.00000788	0
	0.00000186	0.0000075	0
	0.000000168	0.000000338	0
	0	0.000000438	0
	0.000234462	0.000530786	0.000393885
	0.0000426	0.0000851	0.000152615

0	0.000000458	0
0.00000259	0.00000737	0
0.00000238	0.0000076	0
0	0.0000249	0.00000694
0.00000185	0.00000578	0
0.00000185	0.00000578	0
0.00000187	0.00000776	0
0.00000214	0.0000068	0
7.99E-08	0.000000294	0
9.27E-08	0.000000455	0
0.00000254	0.00000819	0
0.00000211	0.0000105	0
0.0000015	0.00000919	0
0.00000196	0.00000654	0
0.00000243	0.0000105	0
0.00000233	0.0000072	0
0	0.000000108	0
0.00000239	0.0000077	0
0.00000447	0.0000116	0
0.00000135	0.0000038	0.00000214
0	0.000000116	0
0.000113641	0.000429887	0.000297602
0.00000225	0.00000782	0
0.00000375	0.00000985	0.00000255
0.00000503	0.0000382	0.000000277
0.00000228	0.00000744	0
0.00000199	0.00000708	0
0.000000145	0.000000393	0
0.00000239	0.00000832	0
0.00000225	0.00000795	0
0.00000147	0.00000644	0
0.00000224	0.00000783	0
0.00000223	0.00000796	0
0.00000244	0.00000998	0
0.0811398	0.0249312	0.00190843
0.00000151	0.00000653	0
0.000000106	0.000000472	0
0.00000215	0.00000801	0
0.00000237	0.00000876	0
0.00000021	0.000000495	0
0.000000605	0.000000554	0.000000381
0.000041	0.0000168	0.0000036
0.00000186	0.00000649	0
0.00000167	0.00000835	0
0.00000167	0.00000835	0
0.000000583	0.000000909	0.000000476
0.0000022	0.00000691	0

0.00000219	0.00000693	0
0.00000221	0.00000827	0
0.00000206	0.00000668	0
0.000000198	0.000000494	0
0.000000164	0.000000475	0
0.00250268	0.00848289	0
0.00000165	0.00000848	0
0	0.00000105	0.000000271
0.00000052	0.000000842	0.0000005
0.00000165	0.00000286	0.00000198
0.00000239	0.00000726	0
0	0.00000012	9.08E-08
0.000123786	0.000218746	0.0000378
0.000000181	0.000000425	0
0.0000286	0.0000309	0.0000114
0.000142835	0.000548751	0.000322548
0.000142835	0.000548751	0.000322507
0	0.000000245	0.000000178
0	0.0000034	0
9.41E-08	0.00000059	0
0	0.00000115	0
0.000101301	0.000178843	0.0000943
0.00000238	0.00000669	0.00000864
0.000000158	0.00000041	0
0.0000053	0.00000452	0
0	0.000000251	0.000000174
0.000300689	0.000616819	0.000199505
0	0.000000305	0.00000021
0.000045	0.0000195	0.00000339
0.000000128	0.000000414	0
0.0000207	0.000107816	0.00000476
0.000000189	0.000000496	0
0.00000946	0.0000147	0.00000718
0.000139745	0.00038361	0.000195215
0.00000684	0.0000378	0
0.0000153	0.0000133	0.00000382
0	0.0024674	0.0016595
0	0.00000481	0
0	0.00000767	0.00000108
0.000118506	0.000274243	0.0000976
0	0.000000322	0.000000203
0.00000105	0.00000168	0.00000106
0.0000162	0.000102345	0.00000225
0	0.000280053	0.000174742
0.000000179	0.000000477	0
0	0.000000524	0.00000032
0	0.000000569	0.000000343

0	0.00000961	0.00000113
0.0000015	0.00000278	0
0.00000189	0.00000403	0
0	0.000000626	0.000000375
0.0000069	0.0000102	0.0000124
0	0.000000878	0.000000225
0	0.000000641	0.000000381
0	0.000000641	0.000000381
0.000036	0.000136393	0.0000407
4.48E-08	0.000000384	0
0.00000194	0.00000401	0
0.00000194	0.00000401	0
0	0.0000112	0
0.0000458	0.000110548	0.000143834
0.000000199	0.00000059	0
8.44E-08	0.000000426	0
0.00000967	0.0000306	0.0000643
0.0000149	0.0000738	0.0000239
0.000288449	0.000653426	0.000184134
0	0.000000403	0
0.00000168	0.0000038	0
0.0000227	0.000151755	0.0000269
0.00000618	0.000017	0.0000114
0.00258156	0.0074474	0
0.0000165	0.0000254	0.00000868
0	0.000000602	0.000000331
0.000390508	0.000638682	0.000124353
0.000000437	0.000000561	0.00000084
0	0.000000578	0.000000315
0.00000253	0.0000139	0
0	0.000000324	0.000000174
0.00000018	0.000000399	0
4.83E-08	0.000000483	0
0.0000709	0.000167243	0.0000175
0.0000342	0.0000536	0.0000282
5.11E-08	0.000000421	0
0.0000153	0.0000151	0.00000369
3.76E-08	0.000000364	0
0.0000164	0.0000233	0.00000765
0.000000189	0.000000305	0
0.000000189	0.000000305	0
0.00000145	0.00000368	0
0.00281807	0.00607775	0
0	0.0000171	0.00000707
0.000177022	0.000565958	0
0.00000167	0.00000342	0
0.00000122	0.00000289	0

5.48E-08	0.000000424	0
0.00000152	0.00000779	0
0.0000121	0.0000156	0.00000384
0.000000108	0.000000309	0
0.00000183	0.00000384	0
0.00000155	0.00000317	0
0.00000152	0.00000324	0
0.00000152	0.00000324	0
0.000270167	0.000456067	0.000134717
0.0000014	0.00000366	0
0.000262981	0.000654834	0.000154716
0.00000151	0.00000309	0
7.57E-08	0.000000411	0
5.42E-08	0.000000423	0
0.00000273	0.0000157	0.00000249
0.0000273	0.0000396	0.0000364
0.000000148	0.000000377	0
0.000000625	0.000000875	0.000000338
0.0000011	0.00000197	0.000000589
0	0.000000822	0.000000385
0.0000466	0.000158164	0.00000393
0.0000211	0.0000177	0.00000337
0.00000955	0.0000228	0.00000702
0	0.000000514	0.000000237
0.000000152	0.000000383	0
0.0000148	0.0000171	0.00000258
0.00000149	0.0000038	0
0	0.00000287	0.000000501
0.00000123	0.00000313	0
0.000000232	0.000000581	0.000000255
0.000000311	0.00000479	0.00000801
0.000000223	0.000000532	0.000000229
0	0.000000384	0.000000165
5.13E-08	0.000000396	0
0	0.00000259	0.000000632
0.000000162	0.000000598	0
0.000000271	0.000000637	0.000000263
0.000000206	0.00000171	0.00000051
0	0.000000697	0.000000347
0.00000019	0.000000568	0.000000234
0	0.000000551	0
0	0.000000632	0.000000257
0.0000406	0.000155424	0.00000507
0.000000184	0.000000542	0.000000217
0.000000144	0.000000491	0
0.0000199	0.000142076	0.00000563
0.000000106	0.000000499	0

	0.000000191	0.000000816	0.000000311
	0.000000191	0.000000816	0.000000311
	6.32E-08	0.000000434	0
	0.0000436	0.000146973	0.000153067
	0	0.000000653	0.000000247
	0.000000168	0.000000726	0.000000272
	0.000000184	0.000000659	0.000000247
	0.000000171	0.000000837	0.000000313
	0.000000193	0.00000104	0.000000376
	0.000000249	0.000000856	0.000000309
	0.000136554	0.000424182	0.000148351
	0.00000019	0.000000666	0.000000233
	0.0318402	0.0895219	0.0469319
	0.0000329	0.00000443	5.16E-08
	0.0000109	0.0000164	0.00000271
	0.0000027	0.00000689	0.00000306
	0.000000286	0.000000991	0.00000034
	0.000000286	0.000000991	0.00000034
	0.000000128	0.000000481	0
	0.000000198	0.000000964	0.000000329
	0	0.00000117	0.000000401
	0	0.00000117	0.000000401
	0	0.000000568	0
	0.000000225	0.000000685	0.00000023
	0	0.000000938	0
	0.000000221	0.000000958	0.00000032
	0.000000279	0.00000361	0.000000763
	0.000000114	0.000000444	0
	0.000000272	0.00000105	0.000000345
	0.0000002	0.000000698	0.000000229
	0.000000172	0.000000893	0.000000293
	0	0.00000608	0.000000659
	0.000000314	0.00000097	0.000000318
	0.000000314	0.000000975	0.000000318
	0.000000326	0.000000747	0.000000242
	0.0000193	0.0000425	0.00000776
	0.00000958	0.0000221	0.00000591
	9.84E-08	0.000000273	0
	0.000000255	0.000000753	0.000000236
	0.000000259	0.000000475	0.000000149
	4.21E-08	0.000000407	0
	0.000305302	0.000411564	0.0000882
	0	0.00000557	0
	0	0.00000184	0
	0.000000293	0.00000108	0.000000322
	0.000000293	0.00000108	0.000000322
	0	0.00000068	0.0000002

0.00000834	0.000102291	0.0000158
0.000000708	0.00000934	0
0.0000128	0.0000233	0.00000508
0	0.000000709	0.000000195
0.000000463	0.00000172	0
0.0000356	0.00017152	0.000207822
0.0000138	0.000147344	0.00000604
8.14E-08	0.00000109	0.000000293
0.000000293	0.00000108	0.000000287
0.000000332	0.000000696	0.000000182
0.00000328	0.00000313	0.000000787
0.0000224	0.0000413	0.0000135
0.000000331	0.00000682	0
0.000000442	0.00000169	0.000000394
0.000000166	0.00000207	0
0.00000176	0.00000204	0.000000527
0.0000258	0.0000531	0.0000113
0.000000302	0.00000186	0
0.0000199	0.000152516	0.00000667
0.00000883	0.0000668	0.0000418
0.000000273	0.00000251	0
0	0.000000578	0
0.000000221	0.00000175	0
0.00000922	0.00003	0.00000325
0.000000187	0.00000163	0
0.000000303	0.0000094	0
0.0000187	0.0000305	0.00000679
0.00000182	0.0000215	0.00000302
5.56E-08	0.000000373	0
0.00000959	0.00003	0.00000396
0	0.000000884	0.000000166
0.0000374	0.0000481	0.00000483
0.00000175	0.00000992	0.00000331
0.00000805	0.0000685	0.000035
0.00000189	0.0000164	0.0000162
0	0.00000147	0.0000001
0.000245346	0.00428686	0
0.000000154	0.00000253	0
0.000000159	0.00000208	0
0.000000433	0.00000346	0.000000833
9.83E-08	0.00000138	0
0.0000135	0.0000319	0.0000105
0	0.00000043	7.13E-08
0.000000108	0.0000015	0
0.0000133	0.0000482	0.0000238
0	0.000000773	0.00000124
0	0.00000818	0

0.0000381	0.000218037	0.000012
0.0000415	0.0000666	0.00000521
0.0000308	0.0000879	0.0000196
0	0.0000013	0.000000192
0.000000172	0.00000155	0
0.00000948	0.0000668	0.0000249
0.00000179	0.0000106	0.00000359
0.000000313	0.0000024	0
0	0.00000653	0.00000194
0.0000884	0.000234303	0.00011941
0	0.00000255	0
0.00000695	0.0000289	0.00000923
0	0.0000011	0
0.00000424	0.0000204	0.00000491
0	0.000000732	9.38E-08
0	0.000000732	9.38E-08
0.000047	0.000273138	0.0000154
0.000000236	0.000000591	7.48E-08
0	0.000000927	0.000000112
8.42E-08	0.00000183	0
0	0.00000102	0.000000122
0	0.00000104	0.000000122
0	0.000000871	0.000000102
0.00000849	0.0000195	0.00000434
0	0.00000195	0.000000222
0	0.0000013	0.000000143
0	0.00000168	0.000000184
0.00000211	0.00000768	0.0000009
0	0.00000409	0.00000081
0	0.000000759	0.000000077
0.000147433	0.0000458	0.000000371
0	0.0000143	0
0.00000242	0.00000244	0.000000432
0.0000135	0.0000712	0.0000125
0	0.00000116	0.000000111
0.000000222	0.000000707	6.76E-08
0.00000012	0.00000228	0
0.00000012	0.00000228	0
0.00000577	0.0000186	0.00000236
0.000000123	0.000000806	0
0.00000199	0.0000076	0.000000404
0.00000627	0.0000162	0.00000384
0	0.00000146	0.000000134
0.00015308	0.0000486	0.000000383
0.00000247	0.00000255	0.0000004
0.0000253	0.000140052	0.0000315
0	0.00000131	0.000000116

0	0.000000666	0
0.000171509	0.0000509	0.000000412
0	0.00000123	0.000000106
0.000000239	0.000000678	5.88E-08
0	0.00000122	0.000000105
0.00000193	0.00000467	0.000000467
0.000153841	0.0000434	0.000000228
0.000156049	0.0000447	0.00000023
0.00000306	0.0000203	0.00000112
0.000151673	0.0000437	0.000000235
0.000133217	0.0000395	0.000000291
0	0.00000102	8.27E-08
0.00000882	0.0000257	0.00000292
0	0.00000221	0
0.000000376	0.00000556	0.00000303
0.00000241	0.00000297	0.000000423
0.000161176	0.0000535	0.000000311
0.0000402	0.0000366	0.000000579
0.0308487	0.0257696	0.000389167
0.000000197	0.000000804	0
0.000000197	0.000000804	0
0	0.00000205	0.000000149
0	0.000000959	6.98E-08
0	0.00000103	7.51E-08
0.000137473	0.0000433	0.000000253
0.000137473	0.0000433	0.000000253
0.0000333	0.0000311	0.00000056
0	0.00000148	0.000000106
0.000159978	0.0000513	0.000000296
0.0000036	0.0000232	0.00000167
0	0.00000108	7.48E-08
0.0000313	0.0000274	0.000000377
0.0000313	0.0000274	0.000000377
0.0000345	0.0000311	0.000000611
0.000000049	0.000000239	0
0.000152502	0.000051	0.000000271
0.0000318	0.0000313	0.000000484
0.0000156	0.0000579	0.00000488
0	0.00000109	7.36E-08
0	0.00000109	7.36E-08
0.000000383	0.00000142	0
0.0000368	0.0000342	0.000000519
0.0000432	0.0000428	0.000000548
0	0.000000931	6.24E-08
0.0000298	0.0000301	0.000000447
0.0000068	0.0000189	0.00000226
0.000106784	0.000127086	0.00000537

0.0000368	0.0000342	0.000000519
0	0.0128318	0.000340956
0.000192912	0.0000665	0.000000435
0.000034	0.0000342	0.000000549
0.000034	0.0000342	0.000000549
0	0.00000133	8.48E-08
0.0000447	0.0000422	0.000000643
0.000000152	0.00000729	0.000000625
7.16E-08	0.000000366	0
7.16E-08	0.000000366	0
0.0000517	0.0000463	0.000000646
0.000132478	0.0000374	0.000000148
0	0.00000246	0.00000228
0	0.00000135	8.24E-08
0.000000176	0.000000869	0
0.0000345	0.0000323	0.00000046
0	0.00000129	7.75E-08
0.0000377	0.0000352	0.000000554
0.00000807	0.0000276	0.00000531
0	0.00000989	0.000000457
0.00000751	0.0000198	0.00000199
0	0.000000848	4.93E-08
0	0.0000139	0.000000487
0	0.000000839	4.81E-08
0	0.000000949	5.43E-08
0	0.00000394	0.000000517
0.00000406	0.000024	0.00000562
0.0000307	0.0000299	0.000000432
0.0000354	0.000159053	0.0000243
0.0000198	0.000141441	0.000033
0.0000451	0.0000813	0.00000547
0.000108087	0.000773848	0.0000616
0.0000332	0.0000316	0.000000546
0	0.00000103	5.63E-08
0.00000235	0.00000315	0.000000331
0	0.00000104	5.57E-08
0.0000345	0.0000331	0.000000411
0.0000355	0.0000341	0.000000407
0.0000309	0.0000307	0.000000413
0	0.00000132	6.79E-08
0.00000234	0.00000307	0.000000314
0.00000234	0.00000307	0.000000314
0	0.00453673	0.000231002
0.0000374	0.0000377	0.000000423
0.0000328	0.0000321	0.000000359
0	0.0000139	0.000000487
0.0000428	0.000188272	0.0000255

0.00000763	0.0000493	0.00000568
0.000000187	0.00000576	7.53E-08
0.00000569	0.000053	0.0000116
0.0000342	0.0000308	0.000000442
0	0.00000125	5.76E-08
0	0.00000162	7.28E-08
0	0.000000294	0
0	0.0000013	5.63E-08
0	0.00002	0.000000612
0.00000199	0.00000261	0.000000204
0.0000348	0.000177552	0.0000224
0	0.00000124	5.05E-08
0	0.0000177	0.000000547
0	0.00000126	5.04E-08
0	0.0000186	0.000000509
0	0.0000172	0.000000464
0.0000364	0.0000354	0.000000353
0.0000187	0.000127555	0.0000155
0	0.000025	0
0	0.00000117	4.51E-08
0	0.00000117	4.51E-08
0.0000124	0.000127555	0.0000262
0.0000124	0.000127555	0.0000262
0	0.00000129	4.88E-08
0.0000114	0.0000823	0.0000152
0	0.00000151	5.74E-08
0	0.00000151	5.74E-08
0	0.000000617	0
0	0.000018	0.00000053
0.000000245	0.000000685	0
0	0.0000204	0.000000543
0.00000262	0.0000104	0.000000348
0	0.00000144	0
0	0.0000177	0.000000455
0.00000422	0.0000428	0.00000801
0.0000012	0.00000172	0.000000131
0	0.0000168	0.000000484
0	0.0000179	0.000000442
0	0.00000174	0.000000062
0.000000236	0.000000661	0
0	0.0000176	0.000000414
0.00000148	0.0000392	0.0000018
0.0000149	0.000108692	0.00000724
0.0000149	0.000108692	0.00000724
0.000000508	0.0000197	0.00000285
0.000121563	0.000490894	0
0	0.0000191	0.00000049

0	0.0000191	0.00000049
0.000000308	0.000000853	0
0.00000031	0.000000719	0
0.00000836	0.0000349	0.00000198
0.000000263	0.00000079	0
0.000000263	0.00000079	0
0	0.00000195	6.23E-08
0	0.000021	0.000000487
0.00000127	0.00000223	0.000000173
0	0.0000201	0.000000465
0	0.0000177	0.000000359
0	0.000000995	3.09E-08
0.000000981	0.00000116	8.24E-08
0.000000981	0.00000116	8.24E-08
0.000000108	0.000000558	0
0.00144349	0.00213801	0.000148041
0	0.0000201	0.000000451
0.00000164	0.00000241	0.000000148
0.00000274	0.00000275	0
0	0.00000122	3.59E-08
0	0.00000122	3.59E-08
0.000000176	0.000000659	0
0	0.00000644	0.000000343
0.000000097	0.00000134	0
0.00000178	0.00000274	0.00000018
0.0000023	0.00000303	0.000000161
0.000000153	0.000000584	0
0.00000195	0.00000302	0.000000188
0.000000431	0.00000767	0.000000302
0.000000163	0.000000762	0
0.0000454	0.000159899	0.00000553
0.0000454	0.000159899	0.00000553
0.0000019	0.00000221	0.000000115
0.0000418	0.000157976	0.00000586
8.77E-08	0.00000129	0
0	0.00000153	0
0	0.0000148	0.000000289
0.000000171	0.00000063	0
0.000000112	0.000000389	0
0.000000236	0.000000812	0
0.00000179	0.00000273	0.000000154
0.0000015	0.00000284	0.000000152
0.000000164	0.000000644	0
0.00000156	0.00000273	0.000000148
7.13E-08	0.00000113	0
0	0.0000173	0.000000298
0.00000163	0.00000293	0.000000162

0.00000153	0.00000281	0.00000014
0	0.0000142	0.000000201
0.00000942	0.0000595	0.00000408
0	0.00000164	0
0.0000252	0.000105333	0.00000774
0	0.0000033	6.69E-08
0.000000159	0.000000692	0
0.000000219	0.000000822	0
0	0.0739933	0.000764707
0.00000742	0.000102414	0.0000255
0.000000109	0.000000682	0
0	0.00000133	0
0.000000432	0.0000327	0.00000223
0.00000113	0.00000293	0.000000142
0.00000196	0.0000581	0.0000159
0	0.000000575	0
0	0.000000535	0
0.00000353	0.000096	0.00000202
0.0000222	0.000152065	0.0000125
0	0.000000498	0
0.00000154	0.0000194	0.00000274
0.0000033	0.0000577	0.0000211
0	0.00000679	0
0.0000012	0.0000155	0.00000213
0.00000149	0.00000916	0
0.00000654	0.0000943	0.0000188
0	0.000000645	0
0.0000168	0.000144549	0.00000578
0.000000181	0.000000617	0
0.000000219	0.0000125	0.00000112
0	0.000000899	0
0.0000165	0.000180853	0.0000141
0	0.000000642	0
0.00000474	0.0000419	0.00000101
0	0.0154167	0.0000819
0.000000223	0.0000164	0.000000264
0.000000958	0.0000048	0.000000376
0	0.00000054	0
0.0000163	0.000158334	0.00000604
0.0000118	0.000115948	0.00000614
0.000000157	0.00000054	0
0.0000033	0.000015	0.00000274
0.000000335	0.0000119	0.000000268
0	0.0000182	7.94E-08
0	0.0000322	0.000000196
0	0.0000435	0.000000254
0.0000184	0.000165656	0.00000386

	0.0000142	0.000148821	0.00000686
	0	0.0000396	0.000000213
	0	0.0000412	0.000000259
	0	0.0000778	0.000000418
	0	0.0000436	0.000000223
	0.0000146	0.000156137	0.00000633
	0	0.0000726	0.000000399
	0	0.000038	0.000000209
	0	0.000000793	0
	0	0.0000613	0.00000023
	0	0.000000549	0
	0	0.00000584	0
	0	0.0000601	0.000000224
	0	0.0000138	0
	0	0.0000419	0.000000217
	0	0.0000785	0.000000374
	0	0.0000366	0.000000149
	0	0.0000433	0.00000022
	0.00000112	0.00000218	0.000000039
	0	0.0000265	9.97E-08
	0	0.0000377	0.000000178
	0	0.0000696	0.000000277
	0	0.0000112	0
	0.00000104	0.00000726	0.000000683
	0	0.0000645	0.000000262
	0	0.0000834	0.000000312
	0	0.0000351	0.000000187
	0	0.0000875	0.000000372
	0.00000529	0.000209822	0
	0	0.0000419	0.000000155
	0	0.0000711	0.000000249
	0	0.000114208	0.000000399
	0	0.000114208	0.000000399
	0	0.0000979	0.000000316
	0	0.0000907	0.000000275
	0	0.0000984	0.000000336
	0	0.0000338	0.000000158
	0	0.0000338	0.000000158
	0	0.0000769	0.000000283
	0.00000095	0.00000862	0.000000822
	0	0.0000819	0.000000273
	0	0.0000704	0.000000226
	0	0.0000575	0.000000184
	0	0.0000602	0.000000196
	0	0.0000602	0.000000196
	0	0.0000984	0.000000305
	0	0.0000703	0.000000297

0	0.0000347	0.000000138
0	0.0000636	0.000000199
0.000000977	0.00000872	0.000000695
0	0.000114208	0.000000299
0.00000066	0.00000587	0.00000051
0	0.000058	0.000000142
0	0.0000676	0.000000208
0.000000837	0.00000693	0.000000662
0	0.0000488	0.000000152
0	0.0000488	0.000000152
0.000000105	0.00000136	0.000000107
0.000000745	0.00000793	0.000000689
0.000000745	0.00000793	0.000000689
0	0.000033	9.25E-08
0	0.000033	9.25E-08
0	0.0000366	0.00000011
0	0.0000624	0.000000183
0	0.0000554	0.00000011
0	0.0000624	0.000000183
0	0.0000649	0.000000278
0	0.0000618	0.000000146
0	0.000053	0.000000101
0	0.0000478	0.000000131
0	0.0000513	0.000000114
0	0.0000338	0.000000117
0.00000134	0.000014	0.000000857
0	0.0000572	0.00000014
0	0.000028	0.000000122
0.00000109	0.00000972	0.000000758
0	0.000052	0.000000115
0.00000012	0.0000108	0
0.00000103	0.0000103	0.000000724
0.000000884	0.00000867	0.00000075
0	0.0000667	0.000000099
0	0.0000667	0.000000099
0.00111625	0.0140039	0.000845115
0	0.0000272	0.000000068
0.000000994	0.0000104	0.000000721
0	0.0000468	0.000000121
0	0.00000617	0
0.000000712	0.00000858	0.000000693
0	0.000051	8.87E-08
0.00000229	0.000132055	0
0.000000881	0.0000103	0.000000703
0	0.0000278	6.88E-08
0	0.0000278	6.88E-08
0	0.0000408	0.00000405

	0	0.0000798	0.000000145
	0.000000759	0.00000956	0.000000604
	0.000000616	0.0000179	0.000000586
	0	0.0000284	8.78E-08
	0.000000839	0.00000868	0.00000046
	0	0.0000269	8.21E-08
	0	0.0000269	8.21E-08
	0.000000698	0.00000823	0.000000571
	0.00000065	0.00000744	0.000000349
	0.000000707	0.00000674	0.000000482
	0	0.00000467	0
	0	0.0000303	4.85E-08
	0.000000604	0.000024	0.00000349
	0	0.0000663	0.000000144
	0.00000061	0.00000713	0.000000373
	0.000000521	0.00000711	0.000000441
	0.000000521	0.00000711	0.000000441
	0	0.000042	2.99E-08
	0.00000106	0.0000962	0.00000569
	0	0.0000584	0.00000006
	0.00000809	0.0000673	0.000000704
	0.00000046	0.0000214	0.000000344
	0	0.0000587	8.08E-08
	0.000000177	0.0000263	4.44E-08
	0	0.0000256	0
	0.000000271	0.0000119	0.000000261
	0.00000364	0.000100627	0.00000254
	0.000000934	0.000113299	0.00000114
	0.000000224	0.0000838	0
	0.000011	0.0000493	0.00000021
	0.00000787	0.0000436	0.000000198
	0.00000326	0.0000302	0.000000138
	0.00341906	0.0185837	0.0000826
	0.00000353	0.000032	0.000000144
	0.000000472	0.0000602	0.00000274
	0.00000904	0.0000399	0.00000016
	0.00000364	0.0000305	0.000000131
	0.00000352	0.000109968	0.00000676
	0.00000176	0.0000543	0.00000074
	0	0.0000152	0.000000325
	0.00000337	0.0000239	0.000000066
	0.00000969	0.0000495	0.000000149
	0.0000107	0.0000521	0.000000151
	0.0000107	0.0000521	0.000000151
	0	0.0000122	0.000000227
	0	0.000024	0.000000409
	0	0.0000168	0.000000245

	0.000000707	0.0000162	7.11E-08
	0	0.0000112	0.000000128
	0	0.0000181	0.000000132

UNC-patient_Post-FMT1	UNC-patient_Post-FMT2	UNC-patient_Post-FMT3
0.0000154	0.00000535	0.000244369
0.0000124	0.00000508	0.000146467
0.0000149	0.00000541	0.00021776
0.0000149	0.00000541	0.00021776
0.0000143	0.00000499	0.000203101
0.0000111	0.00000384	0.000179464
0.0000129	0.00000493	0.000206115
0.0000138	0.00000498	0.00019299
0.000113447	0.000168306	0.0000964
0.0000145	0.000005	0.000199076
0.0000142	0.00000479	0.000189261
0.0000113	0.00000368	0.000219994
0.0000126	0.00000409	0.000180765
0.0000148	0.00000513	0.000194698
0.00012334	0.000179595	0.000110925
0.00876931	0.00310579	0.139375
0.0000165	0.00000567	0.00021974
0.000154234	0.000229607	0.0001463
0.000121739	0.000177675	0.000108742
0.000154048	0.000226173	0.000139357
0.0000151	0.0000051	0.000193322
0.000121622	0.000177043	0.000106699
0.000121725	0.000177217	0.000106813
0.0000201	0.00000725	0.000281167
0.0000129	0.00000454	0.000194953
0.0000178	0.00000603	0.000231162
0.000122501	0.000177042	0.00010924
0.000122501	0.000177042	0.00010924
0.0000132	0.00000434	0.000174173
0.0000131	0.00000453	0.000194443
0.0000184	0.0000063	0.000240251
0.0000164	0.00000671	0.000196067
0.0000164	0.00000671	0.000196067
0.0000132	0.00000466	0.000196719
0.000012	0.00000421	0.000183398
0.000116656	0.000167588	0.000103433
0.0000147	0.00000499	0.000245993
0.0000168	0.00000564	0.000216829
0.0000168	0.00000564	0.000216829
0.0000155	0.00000494	0.000189876
0.0000155	0.00000494	0.000189876
0.000125573	0.000190051	0.00011316
0.0000282	0.0000106	0.0000208
0.0000603	0.000028	0.000048
0.00011192	0.000166109	0.000103105
0.000135038	0.000195275	0.000117644

0.0000459	0.0000217	0.0000354
0.000125029	0.000177155	0.0001104
0.0000603	0.0000272	0.0000462
0.0000335	0.0000131	0.0000252
0.000012	0.00000386	0.000172358
0.0000233	0.000011	0.0000176
0.0000444	0.0000194	0.0000348
0.000127029	0.000192179	0.000121075
0.0000722	0.0000333	0.0000518
0.0000379	0.0000165	0.000029
0.0000565	0.0000269	0.0000443
0.000154048	0.000226173	0.000139357
0.000154048	0.000226173	0.000139357
0.0000912	0.0000418	0.0000698
0.0000787	0.0000372	0.0000607
0.000066	0.0000304	0.0000516
0.0000507	0.0000238	0.0000399
0.0000441	0.0000194	0.0000333
0.000127029	0.000192179	0.000121075
0.0000412	0.0000181	0.0000318
0.0000536	0.0000251	0.0000426
0.0000318	0.0000165	0.0000279
0.0000318	0.0000165	0.0000279
0.000041	0.0000195	0.0000324
0.0000647	0.0000302	0.000000058
0.0000647	0.0000302	0.000000058
0.0000751	0.0000373	0.0000623
0.0000509	0.0000248	0.0000406
0.0000512	0.0000236	0.0000385
0.000122708	0.000057	0.0000922
0.0000503	0.0000229	0.0000382
0.0000471	0.0000203	0.0000348
0.0000607	0.0000271	0.0000475
0.0000546	0.0000246	0.0000432
0.0000397	0.0000181	0.00003
0.0000872	0.0000431	0.0000663
0.14884	0.220364	0.142569
0.0000527	0.0000221	0.00004
0.0000627	0.0000283	0.0000476
0.0000461	0.0000226	0.0000376
0.0000589	0.0000267	0.0000447
0.0000222	0.0000099	0.0000167
0.000057	0.0000247	0.0000417
0.0000578	0.0000249	0.0000429
0.0000618	0.0000289	0.0000479
0.000031	0.0000163	0.0000274
0.000031	0.0000163	0.0000274

0.0000476	0.0000235	0.0000381
0.000048	0.0000221	0.0000378
0.0000612	0.0000339	0.0000515
0.00007	0.0000347	0.0000559
0.0000617	0.0000283	0.0000477
0.0000651	0.0000294	0.0000496
0.0000459	0.0000225	0.0000374
0.0000459	0.0000225	0.0000374
0.0000459	0.0000225	0.0000374
0.0000628	0.0000276	0.000047
0.0000667	0.0000293	0.00005
0.0000614	0.0000279	0.0000476
0.0000708	0.0000367	0.0000585
0.0000619	0.0000305	0.0000505
0.000057	0.0000263	0.000045
0.0000655	0.0000296	0.0000497
0.0000399	0.0000244	0.0000364
0.00000664	0.00000339	0.00000567
0.00000841	0.00000429	0.00000718
0.0000475	0.0000222	0.0000375
0.00000969	0.00000494	0.00000827
0.0000393	0.0000244	0.0000362
0.0000561	0.0000247	0.0000426
0.0000544	0.0000256	0.0000434
0.0000443	0.0000207	0.0000345
0.0000443	0.0000207	0.0000345
0.0000448	0.000022	0.0000365
0.0000448	0.000022	0.0000365
0.0000448	0.000022	0.0000365
0.0000448	0.000022	0.0000365
0.0000557	0.0000285	0.0000453
0.0000561	0.0000286	0.0000457
0.0000728	0.0000368	0.000061
0.000101174	0.0000453	0.0000757
0.0000507	0.0000241	0.0000406
0.0000507	0.0000241	0.0000406
0.0000558	0.0000282	0.0000449
0.0000146	0.00001	0.000000935
0.0000779	0.0000382	0.0000638
0.0000434	0.0000208	0.0000365
0.0000579	0.000026	0.0000436
0.0000518	0.0000232	0.0000398
0.0000796	0.0000385	0.0000615
0.0000498	0.0000234	0.0000436
0.0000487	0.0000209	0.0000371
0.0000421	0.0000176	0.00003
0.0000775	0.0000431	0.0000688

0.0000775	0.0000431	0.0000688
0.0000491	0.0000219	0.0000376
0.0000792	0.0000401	0.0000624
0.0000478	0.0000213	0.0000358
0.0000635	0.0000282	0.000047
0.0000476	0.0000212	0.0000363
0.00021564	0.0000992	0.000166254
0.0000593	0.0000322	0.0000503
0.000101862	0.0000481	0.0000781
0.0000444	0.0000229	0.0000377
0.0000597	0.000027	0.0000458
0.0000862	0.000043	0.0000692
0.000052	0.0000265	0.0000418
0.0000594	0.0000298	0.000047
0.0000678	0.0000313	0.0000509
0.0000565	0.0000268	0.0000436
0.000058	0.000028	0.0000456
0.0000497	0.0000243	0.0000397
0.0000497	0.0000243	0.0000397
0.0000497	0.0000243	0.0000397
0.0000497	0.0000243	0.0000397
0.0000623	0.0000277	0.0000462
0.0000616	0.0000299	0.0000496
0.0000579	0.000028	0.0000471
0.0000583	0.0000276	0.0000448
0.000100691	0.000051	0.000083
0.0000502	0.0000252	0.0000412
0.0000503	0.0000224	0.0000389
0.0000661	0.0000297	0.0000499
0.0000169	0.00000882	0.0000147
0.0000355	0.0000173	0.0000272
0.0000371	0.0000167	0.00003
0.0000363	0.0000177	0.0000293
0.0000779	0.0000396	0.0000641
0.0000468	0.0000209	0.0000361
0.0000421	0.0000226	0.0000366
0.0000961	0.0000451	0.0000733
0.000101862	0.0000481	0.0000781
0.000072	0.0000371	0.0000622
0.000072	0.0000334	0.0000562
0.000083	0.0000431	0.0000705
0.0000424	0.0000193	0.000034
0.0000699	0.0000319	0.0000555
0.000114563	0.0000535	0.0000911
0.00000417	0.0000019	0.00000322
0.0000428	0.0000214	0.0000348
0.0000454	0.0000196	0.0000335

0.0000468	0.0000208	0.0000352
0.0000451	0.0000222	0.0000367
0.0000674	0.0000308	0.0000523
0.0000407	0.0000182	0.0000312
0.0000525	0.0000245	0.0000427
0.00000677	0.00000309	0.00000523
0.0000626	0.0000277	0.0000469
0.0000759	0.000036	0.0000611
0.00000804	0.00000367	0.00000621
0.0000328	0.0000151	0.0000273
0.0000869	0.0000428	0.0000704
0.0000869	0.0000428	0.0000704
0.0000656	0.0000321	0.0000501
0.0000669	0.000032	0.0000502
0.0000371	0.0000167	0.0000297
0.0000254	0.000012	0.0000192
0.0000784	0.0000333	0.0000591
0.000033	0.0000154	0.0000269
0.0000448	0.000021	0.0000335
0.000033	0.0000154	0.0000265
0.0000784	0.0000371	0.0000629
0.0000739	0.0000357	0.0000567
0.0000348	0.0000165	0.0000277
0.00013232	0.0000813	0.000121824
0.0000344	0.0000162	0.0000267
0.0000578	0.0000256	0.0000436
0.0000426	0.0000182	0.0000339
0.0000407	0.0000171	0.0000306
0.0000506	0.0000224	0.0000398
0.0000506	0.0000224	0.0000398
0.0000678	0.0000295	0.000052
0.0000678	0.0000295	0.000052
0.0000143	0.00000661	0.0000111
0.0000215	0.0000109	0.0000171
0.0000215	0.0000109	0.0000171
0.0000285	0.0000105	0.0000454
0	0.000000283	0.0000176
0.0000784	0.000038	0.0000631
0.0000502	0.0000252	0.0000406
0.0000331	0.0000156	0.0000258
0.155382	0.256584	0.105269
0.0000468	0.0000221	0.0000454
0.000047	0.0000239	0.0000419
0.000106358	0.000055	0.0000893
0.0000194	0.00000918	0.0000149
0.225431	0.104437	0.167793
0	0.000000325	0.0000235

0.0000645	0.0000323	0.0000512
0.0000645	0.0000323	0.0000512
0.0000624	0.0000296	0.0000486
0.0000353	0.0000156	0.0000286
0.0000586	0.000032	0.0000499
0	0.000000321	0.0000178
0.0000586	0.0000285	0.0000485
0.0000387	0.0000169	0.0000297
0.0000553	0.0000268	0.0000458
0	0.000000321	0.0000174
0	0.000000296	0.0000178
0	0.000000153	0.0000143
0.0000442	0.0000335	0.0000389
0.0000609	0.0000287	0.0000472
0.0000952	0.0000472	0.0000978
0.0000575	0.0000279	0.0000452
0	0.000000362	0.0000185
0.0000204	0.00000961	0.0000157
0	0.000000131	0.0000129
0	0.000000305	0.0000228
0	0.000000186	0.0000156
0.0000255	0.0000123	0.0000187
0	0.000000575	0.0000236
0.0000195	0.00000977	0.0000158
0.000036	0.000016	0.0000281
0	0.00107825	0.0145849
0	0.000000374	0.0000164
0	0.00000017	0.00000418
0.0000246	0.0000118	0.0000188
0	0.000000168	0.00000452
0	6.13E-08	0.00000502
0	0.000000624	0.0000243
0	0.000000624	0.0000243
0	7.05E-08	0.000069
0.0000454	0.0000202	0.0000674
0	0.000000164	0.00000531
0	0.000000471	0.000019
0	0.000000102	0.00000445
0.000248637	0.000358303	0.0000827
0	0.000000319	0.0000166
0.0000049	0.00000227	0.00000429
0	0.00000026	0.0000173
0	0.000000299	0.0000193
0	0.000000299	0.0000193
0.0000705	0.0000345	0.0000748
0.0000229	0.000011	0.0000166
0.0000229	0.000011	0.0000166

0	0.000000326	0.0000186
0.0000215	0.00000814	0.000000371
0	0.000000309	0.000018
0.0000877	0.000054	0.0000746
0	0.000000325	0.0000178
0.0000277	0.0000131	0.0000216
0	0.000000328	0.0000171
0	0.000000332	0.0000187
0	0.000000183	0.00000535
0	0.000000298	0.0000185
0.000118488	0.0000542	0.0000782
0	0.000000313	0.0000183
0	0.000000147	0.00000489
0	0.000000322	0.0000186
0.0000245	0.0000124	0.0000198
0	0.000000104	0.00000402
0	0.000000325	0.0000183
0.0001881	0.000273539	0.000135893
0	0.000000179	0.00000438
0.000051	0.000023	0.000042
0.000051	0.000023	0.000042
0.0714279	0.106044	0.0995278
0	0.000000494	0.0000201
0.00000855	0.00000463	0.00000739
0	8.92E-08	0.00000423
0.0000814	0.0000388	0.0000944
0.000140657	0.0000819	0.000120211
0	0.00000042	0.0000249
0.0168554	0.0328678	0
0.000103909	0.0000546	0.000111573
0.0000575	0.0000302	0.000107456
0	0.000000224	0.00000625
0.000107534	0.000059	0.000126912
0.0000443	0.0000213	0.0000805
0.000114119	0.0000664	0.000121589
0.000114119	0.0000664	0.000121589
0.000127064	0.0000846	0.0000931
0.00000868	0.00000473	0.00000754
0.0000801	0.0000397	0.0000775
0	0.000385014	0.00349716
0.0000259	0.0000127	0.0000222
0.000051	0.000025	0.0000412
0.00000158	0.00000257	0.00000279
0	0.000000211	0.0000049
0.0000608	0.0000325	0.0000525
0.0000404	0.0000363	0.0000849
0.0000471	0.0000208	0.0000354

0.000143292	0.0000923	0.000165014
0.000143292	0.0000923	0.000165014
0.000143292	0.0000923	0.000165014
0.0000226	0.0000112	0.000019
0.0000226	0.0000112	0.000019
0.0001843	0.000210401	0.000178863
0	9.19E-08	0.00000537
0	0.000000104	0.00000486
0.000122969	0.0001891	0.000197093
0.00000461	0.00000251	0.00000401
0	0.000000212	0.00000492
0	0.000000212	0.00000492
0.000146839	0.000222071	0.000151344
0.000156874	0.000231011	0.000235055
0.000123035	0.000177197	0.000181992
0.000120812	0.0000737	0.000143513
0.00000159	0.00000261	0.00000281
0.000151492	0.000230385	0.000137584
0.000107788	0.00015968	0.000163063
0.000136521	0.0000716	0.000126702
0.0000497	0.0000245	0.0000402
0.0000497	0.0000245	0.0000402
0.0000497	0.0000245	0.0000402
0.0000497	0.0000245	0.0000402
0	0.000000115	0.00000631
0	0.000000135	0.00000528
0.000129485	0.000198766	0.000200631
0	0.00000014	0.00000476
0.000146677	0.000238835	0.0000986
0.0000197	0.00000831	0.0000149
0.000126774	0.000192747	0.000194275
0.000023	0.0000446	0.00000028
0.0000473	0.0000191	0.0000782
0.000116831	0.000062	0.000141533
0.000150261	0.000101561	0.000191479
0.000122654	0.000187137	0.000188278
0.000202761	0.000333441	0.000134556
0.000179519	0.000290649	0.000117335
0.000196414	0.000320239	0.000128584
0.00012709	0.000219964	0.000085
0.000127909	0.000217763	0.0000856
0.0000766	0.0000359	0.000214635
0.000199615	0.000327936	0.000132314
0.00011472	0.000192981	0.0000758
0	0.00287317	0.00237338
0.000215785	0.000353341	0.000137993
0.000118338	0.000202685	0.0000775

0.000129919	0.000221924	0.000086
0	0.000023	0.000000542
0.000112348	0.0000531	0.000111784
0.000140371	0.000239561	0.0000923
0.000164382	0.000266523	0.000109996
0.000128691	0.000222061	0.0000858
0.000198804	0.00032305	0.000130009
0.000125702	0.000218508	0.0000836
0.0000959	0.000049	0.000318221
0.0000959	0.000049	0.000318221
0.000152594	0.000249112	0.000102918
0.000196898	0.000324051	0.000128845
0.000127254	0.000191888	0.000197206
0.000146169	0.000243826	0.0000973
0.000000157	0.000000212	0.000000119
0.000154181	0.000256869	0.000101958
0.000208886	0.000341442	0.000137904
0.0000271	0.0000525	0
0.000142698	0.000238037	0.0000952
0.000182088	0.000293786	0.000120832
0.000176389	0.000292116	0.000112956
0.000177783	0.000296592	0.000114472
0.000198643	0.000329408	0.000132821
0.000132325	0.000226216	0.0000872
0.000196764	0.000321504	0.000130457
0.000132942	0.000226113	0.0000875
0.000179287	0.000297804	0.000114472
0.000209112	0.000340659	0.000138054
0.000122553	0.000212485	0.0000807
2.76E-08	9.14E-08	0.000000716
0.000146402	0.000219095	0.000223495
0.000213545	0.000349554	0.000142808
0.000213545	0.000349554	0.000142808
0.000203716	0.000337089	0.000131907
0.000104219	0.0000496	0.000251789
0.0000585	0.00010384	0.000000108
0.0000585	0.00010384	0.000000108
0.000135203	0.000226468	0.0000899
0.000135203	0.000226468	0.0000899
0	0.000000654	0.000000559
0.000208398	0.000342772	0.000134581
0.000208398	0.000342772	0.000134581
0.000201807	0.000326428	0.000132553
0.000152043	0.00014812	0.000128618
0.000163602	0.000266338	0.000106685
0.000105022	0.000195268	0
0.0000002	0.000000401	0.00000012

0.000000281	0.000000222	0.000000183
0.000000202	0.000000212	0.000000207
0.0000256	0.0000283	0.00000457
0.0000548	0.0000241	0.0000401
0.000000187	0.000000172	0.000000102
0	0.000000158	0.0000561
0.0000257	0.0000148	0.0000964
0.000000295	0.000000307	0.00000017
0	0.00000187	0.000000271
0.000227115	0.000226659	0.000219894
0.000000378	0.00000077	0.000000677
0.0000244	0.0000356	0.00000588
0.000000392	0.0000015	0.00000111
0.0000218	0.0000106	0.0000169
0.000522374	0.000807901	0.000224602
0.000000618	0.0000014	0.000000919
0.0000333	0.0000166	0.000026
0.0000237	0.0000116	0.0000184
0.000128663	0.000129388	0.000156764
0.000000223	0.000000317	0.000000213
0.000000388	0.000000762	0.000000684
0.000000394	0.00000084	0.000000681
0.000000385	0.000000403	0.000000215
0	4.69E-08	0
0.000000299	0.00000111	0.000000893
0.000000745	0.00000127	0.000000885
0.0000309	0.0000162	0.0000247
0.00000105	0.00000159	0.00000105
0.000000247	0.000000308	0.000000246
0.000000247	0.000000308	0.000000246
0.000000375	0.000000837	0.000000861
0.00016143	0.00013936	0.000170471
0.000000283	0.000000273	0.000000298
0.000000207	0.000000411	0.000000204
0	0	0
0.000000249	0.000000436	0.000000218
0.000170608	0.0000935	0.000134499
0.0000353	0.0000208	0.000124842
0.0000781	0.000051	0.0000552
0.000120341	0.0000642	0.000146837
0.0000331	0.0000159	0.0000256
0.000642433	0.00132315	0.00192224
0.0000154	0.00000783	0.0000129
0.00000547	0.00001	0
0.0000539	0.0000268	0.0000433
0.0000984	0.0000542	0.000187629
0	0	0

0.00000649	0.00000331	0.00000548
0.0000319	0.0000341	0.0000782
0.000122928	0.0000688	0.000105838
0.00004	0.0000169	0.0000951
0.0000466	0.0000231	0.0000375
0	0.000000198	0.000000106
0.000163609	0.000113777	0.000282701
0.000163609	0.000113777	0.000282701
0.000163609	0.000113777	0.000282701
0	0	0
0	0.000000706	0.000000522
0.0000893	0.0000406	0.000089
0.000089	0.0000419	0.000064
0.0000319	0.0000135	0.0000228
0.0000588	0.0000268	0.0000464
0	7.99E-08	0.0000691
0.000133952	0.0000781	0.000115791
0.0000998	0.0000511	0.0003391
0.0000292	0.0000123	0.0000208
0.0000837	0.0000373	0.0000916
0	0.000000717	0.000000921
0.000143612	0.0000868	0.000124577
0	0.000000215	0.000000131
0	0	0
0	0	0
2.76E-08	9.14E-08	5.74E-08
0	0	0
0	0.000000114	0.0000542
0.0000294	0.0000129	0.0000214
0	0.000000103	0.0000592
0	0	0
0	0.00159056	0.000141672
0.00000276	0.0000034	0.00000154
0	8.88E-08	0.0000566
0	8.88E-08	0.0000566
0	4.18E-08	0
0	9.39E-08	0.0000605
0	0	0
0.000180965	0.000107255	0.000180338
0	0.000000103	0.0000589
0	0.000000154	0.000067
0	0.000000162	0.0000588
0	0	0
0	0.000000403	0.000000359
0	0.000000327	0.000000164
0	0.000000327	0.000000164
0	9.58E-08	0.0000572

0.000121392	0.0000687	0.000103245
0	0	0.0000542
0	0.000000814	0.00000026
0	0.000000382	0.000000458
0	0.000000259	0.000000256
0	0.000000401	0.000000469
0	0.000000217	0.0000636
0.000113928	0.0000688	0.000227093
0	0.00000127	0.000000297
0	0	0.0000538
0.0000168	0.00000862	0.0000147
0	9.06E-08	0.0000544
0	0.00000063	0.00000032
0	0.000000136	0.000000149
0	0.000000148	0.0000624
0.0000176	0.00000809	0.0000156
0.000248957	0.000159515	0.000323326
0.000248929	0.000159511	0.000323303
0.000104488	0.0000553	0.0000949
0	0.000000141	0.0000543
0	0.000000141	0.0000543
0	0.000000339	0.000000387
0	0.000000124	0.0000618
0	0.0000605	0.0365693
0	9.13E-08	0.0000538
0.0000637	0.0000636	0.0001273
0.00000328	0.0000037	0.00000202
0	0.000000112	0.0000572
0	0.000000235	0.000000232
0	0.000000171	0.000000211
0	0.000000203	0.000000176
0.0000521	0.0000457	0.000120327
0	0	0
0	0.000000258	0.000000164
0	0.000000535	0.000000299
0	0.000000226	0.000000146
0	0.000000104	0.0000564
0	0.000000245	0.000000158
0	0	0.000000662
0	8.83E-08	0.0000579
0.000143292	0.0000923	0.000216617
0	0.000000208	0.000000165
0	8.09E-08	0
0	0.000000189	0.00000019
0	0.000000159	0.0000542
0	8.46E-08	0
0	0.00000022	0.000000155

0	0.000000117	0.000054
0	0.000000368	0.00000037
0	0.000000368	0.00000037
0.000102194	0.0000492	0.000112061
0	6.83E-08	0
0.000018	0.00000209	0.00000182
0	0.000000165	0.0000633
0.0000671	0.0000295	0.00006
0	0.000000152	0.0000813
0	9.13E-08	0
0.00000954	0.00000856	0.00000584
0	0	5.38E-08
0	0.000000279	0.000000195
0.00023868	0.000133849	0.000198105
0	6.78E-08	0.0000474
0.000236964	0.000127425	0.000196453
0.00000744	0.00000786	0.0000584
0.000000654	0	0.00000112
0	0.000000231	0.000000198
0.000000128	0.000000232	0.000000317
0	0.000000139	0.000000213
0	0.000000175	0.000000202
0.0000616	0.0000336	0.0000573
0.000151821	0.0000771	0.000329931
0.000208803	0.000104968	0.000187811
0.000261193	0.000295993	0.000394766
0.000000857	0	0.00000131
0.000215718	0.000121351	0.00038162
0.000642618	0.000937189	0.000644794
0.000124149	0.0000664	0
0	0	0.000000241
0	0.000000124	0
0	0.000000124	0
0	0	0.000000068
0.00006	0.000029	0.0000487
0	0.0000136	0.00000527
0	9.17E-08	0
0	7.14E-08	0
0	6.61E-08	0
0	7.27E-08	0
0	0	0.000000151
0.000155823	0.0000796	0.000127718
0.00000462	0.00000413	0.00000169
0	0	0.000000221
0.000429443	0.000506167	0.000537687
0	6.82E-08	0
0	0	0

0	0.000000161	0.00000436
0	0	0
0.000192707	0.000110752	0.000178642
0	0.0000676	0
0.000156693	0.0000839	0.000147126
0	0	9.41E-08
0.000000254	0.000000876	0.00000179
0	5.06E-08	0
0	0	9.07E-08
0	6.46E-08	0
0	6.46E-08	0
0	0	6.06E-08
0	0	6.06E-08
0.000193966	0.00018303	0.000201979
0	5.22E-08	0
0	0	0.000000241
0	7.39E-08	0
0	0	0.000000121
0.00000271	0.00000238	0.00000401
0.00000426	0.0000032	0.00000141
0.00000382	0.00000411	0.0000367
0	0	0.000000181
0.0000311	0.0000119	0.000006
0.000123934	0.0000623	0.000103928
0.000138005	0.000077	0.000133241
0	0.000000145	0.00000471
0.000121483	0.0000646	0.000259404
0.000000247	0.000000646	0.00000193
0.000000805	0.00000123	0.00000705
2.64E-08	0	0.00000116
0	0.000000973	0
0	0.000000973	0
0	0	0.000000141
0.0000718	0.000035	0.0000775
0.000232476	0.00035762	0.000368104
0	6.11E-08	0
0.00032105	0.000362563	0.00036116
0	0	0
0.000288895	0.000416136	0.000454916
0.00031447	0.000189321	0.000315201
0	0	8.07E-08
0.00031749	0.000483534	0.000485118
0.00000121	0.000000682	0.00000284
0.000367813	0.000545667	0.000538496
0.000275887	0.00017263	0.00032721
0.0003286	0.000490812	0.000497484
0.000259319	0.000391765	0.000422629

0.000259319	0.000391765	0.000422629
0.0000025	0.00000151	0.00000532
0.0000025	0.00000151	0.00000532
0.0000801	0.00004	0.000121593
0.000000127	0.000000524	0.00000139
0.000000388	0.000000487	0.00000113
0.000341551	0.000535147	0.000275542
0.000122175	0.0000637	0.000123773
0	4.97E-08	0
0.00021072	0.000128838	0.000196904
0.00011595	0.0000692	0.000106113
0.00000547	0.00000608	0.00000697
0.000376327	0.000552305	0.000390761
0.000000781	0.00000247	0.0000813
0.0000261	0.0000144	0.0000388
0.00000115	0.000000294	0.000000907
0.0000246	0.00000827	0.0000935
0.000283326	0.000356991	0.000271633
0.000306844	0.000424656	0.000471674
0.00000115	0.000000294	0.000000907
0.00000115	0.000000294	0.000000907
0.000308042	0.000378782	0.000369687
0.000398979	0.000612759	0.000596883
0.000126918	0.000130887	0.000173122
0.000138921	0.000158374	0.000176943
0	0.00000207	0.000000727
0.00029071	0.000388451	0.000273601
0.0000354	0.0000633	0.00000618
0.000383465	0.000566653	0.000517177
0.000311081	0.000428093	0.000288782
0.00000846	0.00000181	0.00000297
0.000477901	0.00073553	0.0006819
0.00000519	0.00000161	0.00000405
0	0.000000429	0
0.00000727	0.00000274	0.00000679
0.000000784	0.00000186	0.00000198
0.00000163	0.00000142	0.00000727
0.00000086	0.00000185	0.00000214
0.000000243	0.000000643	0.00000251
0.000000243	0.000000643	0.00000251
0.00000109	0.00000238	0.00000206
0.000290049	0.000451379	0.00059092
0	0.000000428	0
0	0.000000541	0
0	0.000000047	0
0	0.000362831	0
0.00000115	0.00000267	0.0000021

0.000378713	0.000476782	0.000515173
0.0000859	0.0000532	0.0000918
0.000491325	0.000755713	0.000576538
0.00000125	0.00000294	0.00000213
0	0.000000306	0
0	0.000000415	0
0	0.000000628	0
0.000000378	0.000000344	0.000000967
0	0.0000001	0.000000694
0.000316652	0.000506153	0.000204184
0.000115766	0.000104208	0.00018276
0	0.000000353	0
0.000000975	0.00000238	0.0000019
0	0	0
0.000174636	0.000140746	0.000192803
0.000254414	0.000353138	0.000285867
0.000370332	0.000466321	0.000511867
0	0.000000604	0
0.0000781	0.0000345	0.0000591
0.000298831	0.000444896	0.000426165
0.000000115	0.000000208	0.000000155
0	0	0
0.000213104	0.000185538	0.000243199
0	0.000000568	0
0.000376332	0.000560714	0.000390761
0.000366013	0.000541032	0.000360554
0.000101207	0.0000544	0.0000898
0.000000959	0.000000622	0.00000211
0.00030586	0.000405259	0.00052564
0	0.000000455	0
0	0	0.00000162
0.000000222	0.000000118	0.000000115
0	0	0.000000515
0	0.00000042	0
0.00000121	0.00000272	0.00000205
0	0	0.000000763
0.000155895	0.0000901	0.000148795
0.0000164	0.0000524	0
0.000000938	0.00000118	0.000000747
0	0	0.000000116
0.0000847	0.000109918	0.0000421
0	0.00000028	0
0.00060288	0.000837119	0.000701217
0.0000739	0.0000342	0.000059
0	0.000000396	0
0.0000269	0.0000448	0.00000798
0.0000269	0.0000448	0.00000798

0.0172544	0.0482583	0
0.0000153	0.0000515	0
0.000000825	0.000000693	0.00000132
0.000272513	0.000152403	0.000542496
0	0.000000488	0.000000398
0.000360745	0.000502237	0.000580306
0.00000967	0.00000433	0.00000823
0.000165838	0.00012241	0.000204319
0.000100221	0.0000506	0.000144416
0.00000968	0.00000762	0.00016173
0.000220989	0.000113584	0.000205212
0.000124	0.000063	0.000131553
0	0	0
0.0000134	0.0000529	0
0.000180845	0.000261976	0.000156618
0	8.48E-08	0
0.0000169	0.000056	0
0.0000169	0.000056	0
0.0000434	0.0000208	0.0000365
0	0.0000003	0.000000992
0.0000534	0.0000262	0.000106971
0.0000011	0.00000226	0
0	0	0
0.00000112	7.22E-08	0.00000108
0.000525705	0.000704986	0.000568748
0.000000193	0.00000013	0.00000131
0.00000488	0.00000263	0.00000497
0.00039151	0.00059236	0.000441263
0	0	0.000000193
0.000804766	0.00121769	0.000987015
0.00000806	0.00000364	0.00000694
0.73728	0.727023	0.731499
0	0	0
0.000000187	0	3.46E-08
0.00000116	0.000000752	0.000000811
0.000525123	0.000745681	0.000711206
0.00013862	0.0000699	0.000344526
0	5.27E-08	0
0.000045	0.0000178	0.0000458
0.000000296	0	0.000000649
0	0	0
0	0	0
0.000119071	0.0000647	0.000166914
0.00000445	0.00000346	0.00000654
0.000000337	0.000000237	0.00000181
0.0000282	0.0000113	0.00000679
0.0000956	0.0000489	0.0000813

0	0	0.000000175
0.000503237	0.000740909	0.000527965
0.0000136	0.0000501	0
0	0	0
0	0	0
0	0	0
0.000000265	0	0.000000176
0.000000324	0	0.000000107
0.000000253	0	0.000000173
0.0000108	0.0000418	0
0.000131139	0.0000724	0.000151331
0	0	0
0.00000602	0.00000825	0.00000258
0.0000053	0.00000487	0.00000669
0.00016744	0.000273998	0.0000297
0.00000982	0.00000321	0.00000778
0.000625102	0.000867859	0.000751718
0	0	0
0.0000073	0.0000101	0.00000226
0.00000902	0.00000246	0
0	0	0.000000675
0.000000154	0.00000034	0.00000182
0.000000154	0.00000034	0.00000182
0.000000154	0.00000034	0.00000182
0.00038301	0.000564264	0.00044991
0.000505776	0.00072095	0.000689641
0	0	0
0.00000893	0.00000228	0
0.0000104	0.00000802	0
0.00000807	0.0000026	0
0.0000132	0.0000535	0
0.00000878	0.00000249	0
0.00000786	0.0000296	0
0.000291772	0.000375408	0.000372657
0.0000102	0.0000368	0
0	0	0
0.00000927	0.0000376	0
0.00000937	0.0000379	0
0.00000937	0.0000379	0
0	9.68E-08	0
0.000526356	0.000756967	0.000713252
0.000509319	0.000723799	0.000693315
0.000313124	0	0.000244851
0.00000951	0.00000288	0
0	2.13E-08	0
0	2.13E-08	0
0	0	0

0.00000866	0.00000235	0
0.00000866	0.00000235	0
0.00000932	0.0000408	0
0.00000849	0.00000247	0
0.0000114	0.00000353	0
0.000138284	0.000123972	0.000143054
0	0	0
0.000182861	0.000253796	0.000243887
0.00000894	0.00000275	0
0.00029251	0.000405961	0.000379908
0.00000868	0.00000239	0
0.0000101	0.00000321	0.00000778
0.00000836	0.00000293	0
0.000002	0.0000012	0.00000177
0.00000987	0.0000402	0
0.00000988	0.00000325	0
8.99E-08	0	0.00000013
0	0	0.00000022
0	0	0
0	0	0
0.00000907	0.0000028	0
0.00000847	0.000003	0
0.0000146	0.0000532	0
0.00000893	0.00000237	0
0.00000983	0.0000393	0
0.000000163	0	0.000000106
0.00000887	0.00000285	0
0.00000279	0.00000269	0.00000202
0.00000831	0.00000266	0
0.00000831	0.00000266	0
0	6.03E-08	0
0	8.97E-08	0
0.00035974	0.000507092	0.000381392
0.000449323	0.000640466	0.00061044
0.000449323	0.000640466	0.00061044
0.0000079	0.0000328	0
0.0000132	0.00000982	0.0000311
0.00000985	0.00000313	0
0.00000902	0.0000393	0
0.00000902	0.0000393	0
0	0	0
0	0	0
0	0	0
0	0	0
0	0	0
0	0	0
0	0	0

0	0	0
0	0	0
0	0	0
0	0	0
0	0	0
0	0	0
0	0	0
0	0	0
0	0	0
0	0	0
0	0	0
0	0	0
0	0	0
0	0	0
0	0	0
0	0	0
0	0	0
0	0	0
0	0	0
0	0	0
0	0	0
0	0	0
0	0	0
0	0	0
0	0	0
0	0	0
0	0	0
0	0	0
0	0	0
0	0	0
0	0	0
0	0	0
0	0	0
0	0	0
0	0	0
0	0	0
0	0	0
0.00426989	0.00134401	0.0129033
0	0	0
0	0	0
0	0	0
0	0	0
0	0	0
0	0	0
0	0	0
0	0	0
0	0	0

0	0	0
0	0	0
0	0	0
0	0	0
0	0	0
0	0	0
0	0	0
0	0	0
0	0	0
0	0	0
0	0	0
0	0	0
0	0	0
0	0	0
0	0	0
0	0	0
0	0	0
0	0	0
0	0	0
0	0	0
0	0	0
0	0	0
0	0	0
0	0	0
0	0	0
0	0	0
0.000895381	0	0.00111138
0	0	0
0	0	0
0	0	0
0	0	0
0	0	0
0	0	0
0	0	0
0	0	0
0	0	0
0	0	0
0	0	0
0	0	0
0	0	0
0	0	0.0152714
0	0.0000456	0
0	0	0
0	0	0
0	0	0.000532038
0	0	0
0	0	0

0	0	0
0	0	0
0	0	0
0	0	0
0	0	0
0	0	0
0	0	0
0	0.00406381	0
0	0	0.000207189
0	0	0
0	0	0
0	0	0
0	0	0
0	0	0
0	0	0
0	0	0
0	0	0
0	0	0
0	0	0
0	0	0
0	0	0
0	0	0
0	0	0
0	0	0
0	0	0.000127036
0	0	0
0	0	0
0	0	0
0	0	0
0	0	0
0	0	0
0	0	0
0	0	0
0	0	0
0	0	0
0	0	0
0	0	0
0	0	0
0	0	0
0.000664364	0	0
0	0	0
0	0	0
0.000537335	0	0
0	0	0.00298744
0	0	0
0	0	0
0	0	0

0	0	0
0	0	0
0	0	0
0	0	0
0	0	0
0	0	0
0	0	0
0	0	0
0	0	0
0	0	0
0	0	0
0	0	0
0	0	0
0	0	0
0	0	0
0	0	0
0	0	0
0	0	0.000000114
0	0	0
0	0	0
0	0	0
0	0	0
0	0	0
0	0	0
0	0	0
0	0	0
0	0	0
0.000000663	0.000000282	0.000000252
0	0	0
0	0	0
0	0	0
0	0	0
0	0	0
0	0	0
0	0	0
0	0	0
0	0	0
0	0	0
0	0	0
0	0	0.0000333
0	0	8.07E-08
0	0	0
0	0	0
0	0	0
0	0	0.000000302
0	0	0
0.000000467	0	0

0	0	0.000000204
0	0	0
0	0	0
0	0	0
0	0	0
0	0	0
0	0	0
0	0	0
0	0	0
0	0	0
0	0	0
0	0	0
0	0	0
0	0	0
0	0	0
0	0	0
0	0	0
0	0	0
0	0	0
0	0	0
0	0	0
0	0	0
0	0	0
0	0	0
0	0	4.77E-08
0	0	0
0	0	0
0	0	0
0	0	0
0.000000959	0	0
0	0	0.00000499
0	0	0
0	0	0
0	0	0
0	0	0
0	0	0
0	0	0
0	0	0
0	0	0
0	0	0
0	0	0
0	0	0
0	0	0
0	0	0
0	0	0
0	0	0
0	0	0

0	0	0
0	0	0
0	0	0
0	0	0
0	0	0
0	0	0
0	0	0
0	0	0
0	0	0
0	0	0
0	0	0
0	0	0
0	0	0
0	0	0
0	0	0
0	0	0
0	0	0
0	0	8.41E-08
0	0	0
0	0	0
0	0	0
0	0	0
0	0	0
0	0	0
0	0	0
0	0	0
0	0	0
0	0	0
0	0	0
0	0	0
0	0	0
0	0	0
0	0	0
0	0	0
0	0	0
0	0	0
0	0	0
0	0	0
0	0	0
0	0	0
0	0	0
0	0	0
0	0.000000574	0.000000204
0	0	0
0	0	0
0	0	0.000000214
0	0	0

0	0	0.000000041
0	0	0
0	0	0
0	0	0
0	0	0
0	0	0
0	0	0
0	0	0
0	0	0
0	0	0
0	0	0
0	0	0
0	0	4.16E-08
0	0	0
0	0	0
0	0	0
0	0	0
0	0	0
0.000000347	0	0
0	0	0
0	0	0
0	0	0
0	0	0
0	0	0
0	0	0
0	0	0
0	0	0
0	0	0
0	0	0.0000311
0	0	0.000000236
0	0	0
0	0	8.74E-08
0	0	0
0	0	0
0	0	0
0	0	0
0	8.38E-08	0
0	0	0
0	0	0
0	0	0
0	0	0
0	0	0
0	0	0
0	0	0
0	0	0
0	0	0
0	0	0

0	0	0
0	0	0
0	0	0
0	0	0
0	0	0
0	0	0
0	0	0
0	0	0
0	0	0
0	0	0
0	0	0
0	0	0
0	0	0
0	0	0
0	0	0
0	0	0
0	0	0
0	0	0
0	0	0
0	0	0
0	0	0
0	0	0
0	0	0
0	0	0
0	0	0
0	0	0
0.00000647	0.00000215	0.0000202
0	0	0
0	0	0
0	0	0
0	0	0
0	0	0
0	0	0
0	0	0
0	0	0
0	0	0
0	0	0
0	0	0
0	0	0
0	0	0
0	0	0
0	0	0
0	0	0
0	0	0
0	0	0
0	0	0
0	0	0

0	0	0
0	0	0
0	0	0
0	0	0
0	0	0
0.000000145	0	0.000000113
0	0	0
0	0	0
0	0	0
0	0	0
0	0	0
0	0	0
0	0	0
0	0	0
0	0	0
0	0	0
0	0	0.0000313
0	7.27E-08	0
0	0	0
0	0	0
0	0	4.96E-08
0	0	0
0	0	0
0	0	0
0	0	0
0	0	0
0	0	0
0	0	0
0	0	0
0	0	0
0	0.000000291	0
0	0	0
0	0	0
0	0	0
0	0	0
0	0	0
0	0	0
0	0	0
0	0	0
0	0	0
0	0	0
0	0	0
0	0	0
0	0	0
0	0	0.000000247
0	0	0
0	0	0
0	0	0

0	0	0
0	0.000000452	0
0	0	0
0	0	0
0	0	0
0	0	0
0	0	0
0.000000325	0	0
0	0	0
0.000000324	0	0
0	0	0.00000309
0	0	0
0	0	0
0	0	0
0	0	0
0	0	0
0	0	0
0	0	0
0	0	0
0	0	0
0	0	0
0.00000647	0.00000215	0.000018
0	0	0
0	0	0
0	0	0
0	0	0
0	0	0
0	0	0
0	0	0
0	0	0
0	0	0
0	0	0
0	0	0
0	0	0
0	0	0
0	0	0
0	0	0.000000182
0	0	0
0	0	0
0.000000265	0	0
0.000000208	0	0
0	0	0.00000305
0	0	0
0	0	0
0	0	0
0	0	0
0	0	0

0	0	0
0	0	0
0	0	0
0	0	0
0	0	0
0	0	0
0	0	0
0	0	0
0	0	0
0	0	0
0	0	0
0	0	0
0	0	0
0	0	0
0	0	0
0	0	0
0	0	0
0	0	0
0	0	0
0	0	9.55E-08
0	0	0
0	0	0
0	0	0
0	0	0
0	0	0
0	0	0
0	0	0
0	0	0
0	0	0
0	0	0
0	0	0
0	0	0
0	0	0
0	0	0
0	0	0
0	0	0
0	0	0
0	0	0
0	0	0
0	0	0
0	0	0
0	0	0
0	0	0
0	0	0
0.0000063	0.00000195	0.0000214
0	0	0
0	0	0

0	0	0
0	0	0
0	0	0
0	0	0
0	0	0
0	0	0
0	0	0
0	0	0
0	0	0
0	0	0
0	0	0
0	0	0.000028
0	0	0
0	0	0
0	0	0
0	0	0
0	0	0
0	0	0
0	0	0
0	0	0
0	0	0
0	0	0.000000147
0.00000156	0	0
0	0	0
0	0	0
0	0	0
0	0	0
0	0	0
8.53E-08	0	9.93E-08
0	0	0.00000013
0	0	0
0	0	0
0	0	0
0	0	8.93E-08
0	0	0
0	0	0
0	0	0
0	0	0
0	0	0
0	0	0
0	0	0
0	0	0
0	0	1.94E-08
0	0	0
0	0	0
0	0	0
0	0	0

0	0	0.000000207
0	0	0
0	0	0
0	0	0
0	0	0
0	0	1.94E-08
0	0	0
0	0	0
0	0	3.32E-08
0.000000563	0.000000371	0.000000531
8.63E-08	0	0.000000128
0	0	0
0	0	0
0	0	0
0	0	0
0	0	8.08E-08
0	0	0
0	0	0
0	0	0
0.000000724	0	0
0	0	0.0000032
0	0	0
0	0	0
0	0	0
0	0	0
0	0	0
0	0	0
0	0	0
0	0	0
0	0	0
0	0	0
0	0	0
0	0	0
0	0	0.0000268
0	0	3.79E-08
0	0	0
0	0	0
0	0	0
0.00000106	0.000000403	0.000000648
0	0	0
0	0	0
0	0	0
0	0	0
0	0	0
0	0	0
0	0	0
0	0	0

0	0	0
1.25E-08	0	0
0	0	0.000000121
0	0	0
0	0	0
0	0	8.83E-08
0	0	0
0	0	0
0	0	0
0.000000168	0	0
0.00000194	0.00000242	0.00000244
0	0	0
0	0	0
0	0	0
0	0	0
0	0	0
0	0	0
0	0	0.0000275
0	0	8.01E-08
0	0	0
0	0	0
0	0	0
0	0	0
0	0	0
0	0	0
0	0	0
0.00000544	0.00000173	0.0000155
0	0	0
0	0	0
0	0	0
0	0	0
0	0	0
0	0	0
0	0	5.46E-08
0	0	0
0	0	0
0	0	0
0	0	0
0	0	0
0	0	0
0	0	0
0	0	0
0.000000117	0	0
0	0	0.00000356
0	0	0
0	0	0
0	0	0

0	0	0
0	0	0.0000001
0	0	0
0.000000295	0	0
0	0	0
0	0	0
0	0	0
0	0	0
0	0	0
0	0	0
0	0	0
0	0	0
0	0	0
0	0	0
0	0	0
0	0	0.000000143
0	0	0
0	0	0
0	0	0
0	0	0
0	0	0
0.000000388	0	0
0	0	0
0	0	0
0	0	0
0.00000192	0.00000195	0.00000079
0	0	0
0.000000328	0	0.00000018
0	0	0
0	0	0
0	0	0
0	0	0
0.000000406	0	0
0	0	0
0	0	0
0	0	0
0	0	0
0	0	0
0	0	0
0	0	8.49E-08
0	0	0
0	0	0
0.00000028	0	0
0	0	0
0	0	0
0	0	0
0	0	8.42E-08

0	0	0
0	0	0
0.000000285	0	0
0	0	0
0	0	0
0	0	9.65E-08
0	0	0
9.05E-08	7.24E-08	0.000000725
0	0	0
0	0	0
0	0	2.35E-08
0	0	0
0	0	0
0	0.000000356	0.00000161
0	0	0
0	0	0
0	0	0
0	0	0
0	0	0
0	0	0
0	0	0
0	0	0
0	0	0
0	0	0
0	0	0
0	0	0
0	0	0
0	0	0
0	0	0
0	0	0
0.00000679	0.00000196	0.0000193
0	0	0
0	0	0
0	0	0
0	0	0
0	0	0
0	0	0
0	0	0
0	0	0
0	0	0
0	0	0
0	0	0
0	0	0
0	0	0
0	0	0
0	0	0
0	0	0

	0	0	0
	0	0	0
	0	0	0
	0	0	0
	0	0	0
	0	0	0
	0	0	0
	0	0	0
	0	0	0
	0	0	0.000000039
	0	0	0
	0	0	0
	0	0	0
	0	0	0
	0	0	0
	0	0	0
	0	0	0
	0	0	0
	0	0	0
	0	0	0
	0	0	0
	0	0	0
	0	0	0
	0	0	0
	0	0	0
	0	0	0
	0	0	0
	0	0	0
	0	0	0
	0	0	0
	0	0	0
	0	0	0
	0	0	0
	0	0	0
	0	0	0
	0	0	0
	0	0	0
	0	0	0
	0	0	0
	0	0	0
	0	0	0
	0	0	0.0000147
	0	0	0.000000206
	0	0	0
	0	0	0
	0	0.000000292	0
	0	0	0.000000169

0	0	0
0	0	0
0	0	0
0	0	0
0	0	0
0	0	0
0.000000451	0	0
0	0	0.00000288
0	0	0
0	0	0
0	0	0.000000204
0	0	0
0	0	0
0	0	0
0	0	0.00000039
0	0	0
0	0	0
0	0	5.39E-08
0	0	0.0000458
0	0	0
1.33E-08	0	0
0	0	0.000000413
0	0	0
0	0	0
0	0	0
0	0	0
0	0	0
0	0	0
0	0	0
0	0	0
0	0	9.74E-08
0	0	0
0	0	0
0	0	0
0	0	0
0	0	0
0	0	0
0	0	0
0	0	0
0	0	0
0	0	0
0	0	0
0	0	0
0	0	0
0	0	0
0	0	0
0	0	0

	0	0	0
	0	0	0
	0	0	0
	0	0	0
	0	0	0
	0	0	0
	0	0	0
	0	0	0
	0	0	0
	0	0	0
	0	0	0
	0	0	0
	0	0	0
	0	0	0
	0	0	0
	0	0	0
	0	0	0
	0	0	0
	0	0	0
	0	0	0
	0	0	0
	0	0	0
	0	0	0
	0	0	0
	0	0	0
	0	0	0
	0	0	0
	0	0	0
	0	0	0
	0	0	0
	0	0	0
	0	0	0
	0	0	0
	0	0	0
	0	0	0
	0	0	0
	0	0	0
	0	0	0
	0	0	0
	0	0	0
	0	0	5.19E-08
	0	0	0
	0	0	0
	0	0	0
	0	0	0
	0	0	0
	0	0	0

0	0	0
0	0	0
0	0	0
0	0	0
0	0	0
0	0	0
0	0	0
0	0	0
0	0	0
0	0	0
0	0	0
0	0	0
0	0	0
0	0	0
0	0	0
0	0	0
0	0	0
0	0	0
0	0	0
0	0	0
0	0	0
0	0	0
0	0	0
0	0	0
0	0	0
0	0	0
0	0	0
0	0	0
0	0	0
0	0	0
0	0	0
0	0	0
0	0	0
0	0	0.000000126
0	0	0
0	0	0
0	0	0
0	0	0
0	0	0
0	0	0
0	0	0
0	0	0
0	0	0
0	0	0
0	0	0
0	0	0
0	0	0

0	0	0
0	0	0
0	0	0
0	0	0
0	0	0
0	0	0
0	0	0
0	0	0
0	0	0
0	0	0
0	0	0
0	0	0
0	0	0
0	0	0
0	0	0
0	0	0
0	0	0
0.00000685	0.00000204	0.0000203
0	0	0
0	0	0
0	0	0
0	0	0
0	0	0
0	0	0
0	0	0
0	0	0
0	0	0
0	0	0
0	0	0
0	0	0
0	0	0
0	0	0
0	0	0
0	0	0
0	0	0
0	0	0
0	0	0
0	0	0
0	0	0
0	0	0
0	0	0
0	0	0
0	0	0
0	0	0
0	0	0
0	0	0
0	0	0

0	0	0.00000226
0	0	0
0	0	0
0	0	0
0	0	0
0	0	0
0	0	0
0	0	0
0	0	0
0	0	0
0	0.000000299	0
0	0	0
0	0	0
0	0	0
0	0	0
0	0	0
0	0	0
0	0	0
0	0	0
0	0	0
0	0	0
0	0	0
0	0	0
0	0	0.000000191
0	0	0
0	0	0
0	0	0
0	0	0
0	0	0
0	0	0
0.000000294	0	0
0	0	0
0.000000458	0	0
0	0	0.00000293
0	0	0
0	0	0
0	0	0.000000104
0	0	0
0	0	0.00000334
0	0	0
0	0	0
0	0	0.000000179
0	0	9.05E-08
0	0	7.81E-08
0	0	0
0	0	0
0	0	0

0	0	0
0	0	0
0	0	0
0	0	0
0	0	0
0	0	0
0	0	0
0	0	0
0	0	0
0	0	0
0	0	0
0	0	0
0	0	0
0	0	0
0	0	0
0	0	0
0	0	0
0	0	0
0	0	0
0	0	0
0	0	0
0	0	0
0	0	0
0	0	0
0	0	0
0	0	0
0	0	0
0	0	0
0	0	0
0	0	0
0	0	0
0	0	0.000000115
0	0	0
0	0	0
0	0	0
0	0	0
0	0	0
0	0	0.00000028
0	0	0
0	0	0
0	0	0
0	0	0
0.000000378	0	0
0	0	0
0	0	0
0	0	0
0	0	0

0	0	0
0	0	0
0	0	0
0	0	0
0	0	0
0	0	0
0	0	0
0	0	0
0	0	0
0	0	0
0	0	0
0	0	0
0	0	0
0	0	0
0	0	0
0	0	0
0.000000132	0	8.71E-08
0	0	0
0	0	0
0	0	0
0	0.000000265	0
0	0	0
0	0	0
0	0	0
0	0	0
0	0	0
0	0	0
0	0	0
0	0	0
0	0	0
0	0	0
0	0	0
0	0	0
0	0	0
0	0	0
0	0	0
0	0	0
0	0	0
0	0	0
0	0	0
0	0	0
0	0	0
0	0	0
0	0	0
0	0	0
0	0	0
0	0	0

0	0	0
0	0	0
0	0	0
0	0	0
0	0	0
0	0	0
0	0	0
0	0	0
0	0	0
0	0	0
0	0	0
0	0	0
0	0	0
0	0	0
0	0	0
0	0	0
0	0	0
0	0	0
0	0	0
0	0	0
0	0	0
0	0	0
0	0	0
0	0	0
0	0	0
0	0	0
0	0	0
0	0	0
0	0	0
0	0	0
0	0	0
0	0	0
0	0	0
0	0	0
0	0	0
0	0.000000109	0
0	0	0
0	0	0
0	0	0
0	0	0
0	0	0
0	0	0
0	0.00000023	0
0	0	0
0	0	0
0	0	0
0	0	0

0	0	0
0	0	0
0	0	0
0	0	0
0	0	0
0	0	0
0	0	0
0	0	0
0	0	0
0	0	0
0	0	0
0	0	0
0	0	0
0.00000055	0	0
0	0	0.00000347
0	0	0
0	0	0
0	0	0
0	0	0
0	0	0
0	0	0
0	0	0
0.000000319	0	0
0	0	0
0	0	0
0	0	0
0	0	0
0	0	0
0	0	0
0	0	0
0	0	0
0	0	0
0	0	0
0	0	0
0	0	0
0	0	0
0	0	0
0	0	0
0	0	0
0	0	0
0	0	0
0	0	0
0	0	0
0	0	0
0	0	0
0	0	0
0	0	0

0	0	0
0	0	0
0	0	0
0	0	0
0	0	0
0	0	0
0	0	0
0.000000158	0	0.000000201
0	0	0
0	0	0.000000118
0	0	0
0	0	0
0	0.000000388	0
0	0	0
0	0	0
0	0	0
0	0	0
0	0	0
0	0	0
0	0	0
0.00000023	0	0
0	0	0
0	0	0
0	0	0.0000001
0	0	0
0	0	0
0	0	0
0	0	0
0	0	0
0	0	0
0	0	0
0	0	0
0	0	0
0	0	0
0	0	0
0	0	0
0	0	0
0	0	0
0	0	0
0	0	0
0	0	0
0	0	0
0	0	0
0	0	0
0	0	0
0	0	0.000000195
0	0	0

0	0	0
0.000000123	0	0
0.000000221	0	0
0	0	0.0000029
0	0	0.000000206
0	0	0.000000179
0	0	0.0000001
0	0	9.69E-08
0	0	0
0	0	0
0	0	0
0	0	0
0	0	0
0	0	0
0	0	0
0	0	0
0	0	0
0	0	0
0	0	0
0	0	0
0	0	0
0	0	0
0	0	0
0	0	0
0	0	0
0	0	0
0	0.000000346	0
0	0	0
0	0	0
0	0	0.000000304
0	0	0
0	0	0
0	0	0
0	0	0.000000252
0.000000373	0	0
0	0	0.000000699
0	0	0
0.00000015	0.000000123	0.000000113
0	0	0
0	0	0
0	0	7.88E-08
0	0	0
0	0	0
0	0	0
0	0	0
0	0	0
0	0	0

	0	0	0
	0	0	0
	0	0	0
	0	0	0
	0	0	0
	0	0	0
	0	0	0
	0	0	0
	0	0	0
	0	0	0
	0	0	0
	0	0	0
	0	0	0
	0	0	0
	0	0	0
	0	0	0
	0	0	0
	0	0	0
	0	0	0
	0	0	0
	0	0	0
	0	0	0
	0	0	0
	0	0	0
	0	0	0
	0	0	0
	0	0	0
	0	0	0
	0	0	0
	0	0	0
	0	0	0
	0	0	0
	0	0	0
	0	0	0
	0	0	0
	0	0	0
	0	0	0
	0	0	0
	0	0	0
	0	0	0
	0	0	0
	0	0	0
	0	0	0
	0	0	0
	0	0	0
	0	0	0
	0	0	0

0	0	0
0	0	0
0	0	0
0	0.000000254	0
0	0	0
0	0	0
0	0	0
0	0	0
0	0	0
0	0	0
0	0	0
0	0	0
0	0	0
0	0	0
0	0	0
0	0	0
0	0	0
0	0	0
0	0	0
0	0	0
0	0	0
0	0	0
0.000000407	0	0
0	0	0.00000291
0	0	0
0	0	0
0	0	0
0	0	0
0	0	0
0	0	0
0	0	0
0	0	0
0	0	0
0	0	0
0	0	0
0.000000246	0	0.000000297
0	0	0
0	0	0
0	0	0
0	0	0
0	0.000000146	0
0	0	6.34E-08
0	0	0
0	0	0
0	0	0
0	0	0
0	0	0

0.000000278	0	0
0	0	0
0.000000956	0	0
0	0	0
0	0	0
0	0	0
0	0	0
0	0	0
0	0	0
0	0	0
0	0	0
0	0	0
0	0	0
0	0	0
0	0	0.000000178
0	0	0
0	0	0
0	0	0
0	0	0
0	0	0
0.000000347	0	0
0	0	0
0	0	0
0	0	0
0	0	0
0	0	0
0	0	0
0	0	0
0	0	0
0	0	0
0	0	0
0.000000729	0.000000339	0.00000107
0	0	0
0	0	0
0	0	0
0	0	0
0	0	0.000000349
0	0	0
0	0	0
0.000000634	0.000000322	0.00000106
0	0	0
0.0000535	0	0
0.0000535	0	0
0	0.000000047	0
0	0	0
0	0	0
0	0	0

0	0	0
0	0	0
0	0	0
0	0	0
0	0	0
0	0	0
0	0	0
0	0	0
0	0	0
0	0	0
0	0	0
0	0	0
0	0	0
0	0	0
0	0	0.000028
0	0	3.35E-08
0	0	0
0	0	0
0	0	0
0	0	0
0	0	0
0	0	0
0	0	0
0	0	0
0	0	0
0.000000591	0	0
0	0	0.00000348
0	0	0
0	0	0
0	0	0
0	0	0
0	0	0
0	0	0
0	0	0
0	0	0
0	0	0
0	0	0
0	0	0
0	0	0
0	0	0
0	0	0
0	0	0
0	0	0
0	0	0
0	0	8.41E-08
0	0	0
0	0	0

0	0	0
0.000000213	0	0.000000142
0	0	6.67E-08
0	0	0
0	0	0
0	0	0.000000156
0	0	0
0	0	0
0	0	0.000000136
0	0	0
0	0	0
0	0	0
0	0	0
0	0	0
0	0	0
0	0	0
0	0	0
0	0	0
0	0	0
0	0	0
0	0	0
0	0	0
0	0	0
0	0	0
0	0	0
0	0	0
0	0	0
0	0	0
0	0	0
0	0	0
0	0	0
0	0	0
0	0	0
0	0	0
0	0	0
0	0	0
0	0	0
0	0	0
0	0	0
0	0	0
0	0	0
0	0	0
0	0	0
0	0	0
0	0	0
0	0	0
0	0	0

0	0	0
0	0	0
0	0	0
0	0	0
0	0	0
0	0	0
0	0	0
0	0	0
0	0	0
0	0	0
0	0	0
0	0	0
0	0	0
0	0	0
0	0	0
0	0	0
0	0	0
0	0	0
0	0	0
0	0	0
0	0	0
0	0	0
0	0	0
0	0	0
0	0	0
0	0	0
0	0	0
0	0	0
0	0	0
0	0	0
0	0	0
0	0	0
0	0	0
0	0	0
0	0	0
0	0	0.000000127
0	0	0
0	0	0
0	0	0
0	0	0
0	0	0
0	0	0
0	0	0
0	0	0
0	0	0
0	0	0
0	0	0

0	0	0
0	0	0
0	0	0
0	0	0
0	0	0
0	0	0
0	0	0
0	0	0
0	0	0
0.000000129	0	0
0	0	0
4.31E-08	0	0
0	0	0.00000283
0	0	0
0	0	0
0	0	0
0	0	0
0	0	0
0	0	0
0	0	0
0	0	0
0	0	0
0	0	0
0	0	0
0	0	0
0	0	0
0	0	0
0	0	0
0	0	0
0	0	0
0	0	0
0	0	0
0	0	0
0	0	0
0	0	0
0	0	0
0	0	0
0	0	0
0	0	0
0	0	0
0	0	0
0	0	0
0.00000589	0.0000018	0.000017
0	0	0
0	0	0
0	0	0
0	0	0

	0	0	0
	0	0	0
	0	0	0
	0	0	0
	0	0	0
	0	0	0
	0	0	0
	0	0	0
	0	0	0
	0	0	0
	0	0	0
	0	0	0
	0	0	0
	0	0	0
	0	0	0
	0	0	0
	0	0	0
	0	0	0
	0	0	0
	0	0	0
	0	0	0
	0	0	0
	0	0	0
	0	0	0
	0	0	0
	0	0	0
	0	0	0
	0	0	0.0000357
	0	4.63E-08	0
	0	0	0
	0	0	4.21E-08
	0	0	0
	0	0	0
	0	0	0
	0	0	0
	0	0	0
	0	0.000000485	0
	0	0	0
	0	0	0
	0	0	0
	0	0	0
	0	0	0
	0	0	0
	0	0	0
	0	0	0
	0	0	0
	0	0	0

0	0	0
0	0	0
0	0	0.000000357
0	0	0
0	0	0
0	0	0
0	0	0
0	0	0
0	0	0
0	0	0
0	0	0
0	0	0
0.000000283	0	0
0	0	0
0.00000045	0	0
0	0	0.00000359
0	0	0
0	0	0
0	0	0
0	0	0
0	0	0
0	0	0
0	0	0
0	0	0
0	0	0
0	0	0
0	0	0
0	0	0
0	0	0
0	0	0
0	0	0
0	0	0
0	0	0
0	0	0
0	0	0
0	0	0
0	0	0
0	0	0
0	0	0
0	0	0
0	0	0
0	0	0
0	0	0
0	0	0
0	0	0
0	0	0
0	0	0

0	0	0.000000142
0	0	0
0	0	0
0	0	0
0	0	0
0	0	0
0	0	0
0	0	0
0	0	0
0	0	0
0	0	0
0.000000191	0	0
0	0	0
0.0000015	0.000000537	0.00000165
0	0	0
0.000000113	0	7.92E-08
0	0	0
0	0	0
0	0.000000298	0.00000141
0	0.000000298	0.00000124
0	0.000000298	0.00000124
0	0	0
0	0	0
0	0	5.75E-08
0	0	0.000000359
0	0	0
0	0	0
0	0	5.75E-08
0	0	0.000000359
0	0	0
0	0	0
0	0	5.75E-08
0	0	0.000000359
0	0.000000204	0.00000084
0	0.000000204	0.00000096
0	0.000000204	0.00000084
0	0.000000298	0.00000124
0	0	0
0	0	0
0	0	0
0	0	0
0.00000102	0.00000171	0.00000213
0	0	0
0.000000463	0.000000582	0.00000115
0	0	0
0	0	0
0	0	0

0	0	0
6.55E-08	0	9.28E-08
0	0	9.97E-08
0	0	0
0	0	0
0	0	0
0	0	0
0	0	0
0	0	0
0	0	0
0	0	0
0	0	0
0	0	0
0	0	0
0	0	0
0	0	0
0	0	0
0	0	0
0	0	0
0	0	0
0	0	0
0	0	0
0	0	0
0	0	0
0	0	0
0	0	0
0	0	0
0	0	0
0	0	0
0	0	0
0	0	0
0	0	0
0	0	0
0	0	0
0	0	0
0	0	0
0	0	0
0	0	0
0	0	0
0	0	0
0	0	0
0.000000159	0	0.000000201
0	0	0
0	0	0
0	0	0
0	0	0
0	0	0

0	0	0
0	0	0
0	0	0
0	0	0.0000266
0	9.52E-08	0
0	0	0
0	0	0
0	0	0
0	0	0
0	0	0
0	0	0
0	0	0
0	0.000000512	0
0	0	0
0	0	0
0	0	0
0	0	0
0	0	0
0	0	0
0	0	0
0	0	0
0	0	0
0	0	0
0	0	0
0	0	0
0	0	0
0	0	0
0	0	0
0	0	0
0	0	0
0	0	0
0	0	0
0	0	0
0.00000033	0	0
0	0	0.00000303
0	0	0
0	0	0
0	0	0
0	0	0
0	0	0
0	0	0
0	0	0
0	0	0
0	0	0
0	0	0
0	0	0
0	0	0

0	0	0
0.00000662	0.00000183	0.0000177
0	0	0
0	0	0
0	0	0
0	0	0
0	0	0
0	0	0
0	0	0
0	0	0
0	0	0
0	0	0
0	0	0
0	0	0
0	0	0
0	0	0
0	0	0
0	0	0
0	0	0
0	0	0
0	0	0
0	0	0
0	0	0
0	0	0
0	0	0
0	0	0
0	0	0
0	0	0
0	0	0
0	0	0
0	0	0
0	0	0
0.000000225	0	0.000000234
0	0	0
0	0	0
0	0	0
0	0	0
0	0	0
0	0	0
0	0	0
0	0	0
0	0	0
0	0	0.0000264
0	8.35E-08	0
0	0	0
0	0	3.98E-08
0	0	0

0	0	0
0	0	0
0	0	0
0	0	0
0	0	0
0	0	0
0	0.00000053	0
0	0	0
0	0	0
0	0	0
0	0	0
0	0	0
0	0	0
0	0	0
0	0	0
0	0	0
0	0	0
0	0	0
0	0	0
0	0	0
0	0	0
0	0	0
0	0	0
0	0	0
0	0	0
0	0	0
0	0	0
0.00000029	0	0
0	0	0.00000303
0	0	0
0	0	0
0	0	0
0	0	0
0	0	0
0	0	0
0	0	0
0	0	0
0	0	0
0	0	0
0	0	0
0	0	0
0	0	0
0	0	0
0	0	0
0	0	0
0	0	0
0	0	0

0	0	0
0	0	0
0	0	2.97E-08
0	0	0
0	0	0
0	0	0
0	0	0
5.99E-08	0	0
0	0	0
0	0	0
0	0	0
0	0	0
0	0	0
0	0	0
0	0	0
0	0	0
0	0	0
0	0	0
0	0	0
0	0	0
0	0	0
0	0	0
0	0	0
0	0	0
0	0	0
0	0	0
0	0	0
0	0	0
0	0	0.0000269
0	0	2.93E-08
0	0	0
0	0	0
0	0	0.000000109
0	0	0
0	0	0
3.52E-08	0	0
0	6.32E-08	0
0	0	0
0	0	0
0	0	0
0	0	0
0	0	0
0	0	0
0	0	0
0	0	0
0	0	0
0	0	0

0	0	0
0	0	0.00000019
0	0	0
0.000000261	0	0
0	0	0
0.000000578	0	0
0	0	0.00000361
0	0	0
0	0	0
0	0	0
0	0	0
0	0	0
0	0	0
0	0	0
0	0	0
0	0	0
0	0	0
0	0	0
0	0	0
0	0	0
0	0	0
0	0	0
0	0	0
0	0	0
0	0	7.89E-08
0	0	0
0	0	0
0	0	0
0.000000312	0	0
0	0	0
0.000000645	0	0
0	0	0.00000427
0	0	0
0	0	0
0	0	0
0	0	0
0	0	0
0	0	0
0	0	0
0.000000343	0	0
0.000000739	0	0
0	0	0.00000386
0	0	0
0	0	0
0	0	0
0	0	0
0	0	0

0	0	0
0	0	0
0	0	0
0	0	0
0	0	0
0	0	0
0	0	0
0	0	0
0	0	0
0	0	0
0	0	0
0.00000604	0.00000182	0.0000191
0	0	0
0	0	0
0	0	0
0	0	0
0	0	0
0	0	0
0	0	0
0	0	0
0	0	0
0	0	0
0	0	0
0	0	0
0	0	0
0	0	0
0	0	0
0	0	0
0	0	0
0	0	0
0	0	0
0	0	0
0	0	0
0	0	0
0	0	0
0	0	0
0	0	0
0	0	0
0	0	0
0	0	0
0	0	0
0	0	0
0	0	0.0000281
0	0.000000095	0
0	0	0.00000006
0	0	0
0	0	0

0	0	0
0	0	0
0	0.000000171	0
0	0	0.000000119
0	0	0
0	0.000000124	0.00000483
0	0	0
0	0	0
0	0	0
0	0	0
0	0	0
0	0	0
0	0	0
0	0	0
0	0	0
0	0	0
0	0	0
0	0	0
0.000000345	0	0
0	0	0
0	0	0
0	0	0
0	0	0
0	0	0
0	0	0
0	0	0
0	0	0
0	0	0
0	0	0
0	0	0
0	0	0
0	0	0
0	0	0
0	0	0
0	0	0
0	0	0
0	0	0
0	0	0
0	0	0
0	0	0
0	0	0
0	0	0
0	0	0
0	0	0
0	0	0
0	0	0
0	0	0

0	0	0
0	0	0
0	0	0
0	0	0
0	0	0
0	0	0
0	0	0.000023
0	0	8.76E-08
0	0	0
0	0	0
0	0	0
0	0	0
0	0	0
0	0	0
0	0	0
0	0	0
0	0	0
0	0	0
0	0	0
0	0	0
0	0	0
0	0	0
0	0	0
0	0	0
0	0	0
0.000000327	0	0
0	0	0
0	0	0
0	0	0
0	0	0
0	0	0
0	0	0
0	0	0
0	0	3.32E-08
0	0	0
0	0	0
0	0	0
0	0	0
0	0	0
0	0	0
0	0	0
0	0	0
0	0	0
0	0	0
0	0	7.46E-08
0	0	0
0	0	0

0.00000017	0	0
0	0	0
0	0	0
0	0	0
0	0	0
0	0	0
0	0	0
0	0	0
0	0	0
0	0	0
0	0	0
0	0	0
0	0	0
0	0	0
0	0	0
0	0	0
0	0	0
0	0	0
0	0	0
0.00000662	0.00000183	0.0000177
0	0	0
0	0	0
0	0	0
0	0	0
0	0	0
0	0	0
0	0	0
0	0	0
0	0	0
0	0	0
0	0	0
0	0	0
0	0	0
0	0	0
0	0	0
0	0	0
0	0	0
0	0	0
0	0	0
0	0	0
0	0	0
0	0	0
0	0	0
0	0	0
0	0	0
0	0	0
0	0	0

0	0	0
0	0	0
0	0	0
0.000000225	0	0.000000234
0	0	0
0	0	0
0	0	0
0	0	0
0	0	0
0	0	0
0	0	0
0	0	0
0	0	0
0	0	0.0000264
0	8.35E-08	0
0	0	0
0	0	3.98E-08
0	0	0
0	0	0
0	0	0
0	0	0
0	0	0
0	0	0
0	0	0
0	0.00000053	0
0	0	0
0	0	0
0	0	0
0	0	0
0	0	0
0	0	0
0	0	0
0	0	0
0	0	0
0	0	0
0	0	0
0	0	0
0	0	0
0	0	0
0	0	0
0	0	0
0	0	0
0	0	0
0	0	0
0	0	0
0	0	0
0.00000029	0	0
0	0	0.00000303

0	0	0
0	0	0
0	0	0
0	0	0
6.81E-08	0	9.23E-08
0	0	0.000000116
0.00000057	0.000000941	0.00000116
0	0	0
0	0	0
0	0	0
0	0	0
0	0	8.62E-08
0	0	0
0	0	0
0	0	0
0	0	0
0	0	0
0	0	0
0	0	0
0	0	0
0	0	0
0	0	0
0	0	0
0	0	0
0	0	0
0	0	0.0000364
0	0	0
0	0	0
0	0	0
0	0	0
0	0	0
0	0	0
0	0	0
0	0	0
0	0	0
0	0	0
0	0	0
0	0	0
0	0	0
0	0	0
0	0	0
0.00000749	0.00000231	0.0000223
0	0	0
0	0	0
0	0	0
0	0	0
0	0	0

0	0	0
0	0	0
0	0.000000194	0.000000239
0	0	0
0	0	0
0	0	0
0	0	0
0	0	0
0.000000231	0	0
0	0	0.00000274
0	0	0
0	0	0
0	0	0
0	0	0
0	0	0
0	0	0
0	0	0
0	0	0
0	0	0
0	0	0
0	0	0
0	0	0
0	0	0
0	0	0
0	0	0
0	0	0
0	0	0
0	0	0
0	0	0
0	0	0
0	0	0
0	0	0
0	0	0
0	0	0
0.00000658	0.00000206	0.0000196
0	0	0
0	0	0
0	0	0
0	0	0
0	0	0
0	0	0
0	0	0
0	0	0
0	0	0
0	0	0
0	0	0
0	0	0

0	0	0
0	0	0
0	0	0
0	0	0
0	0	0
0	0	0
0	0	0
0	0	0
0	0	0
0.000000188	0	0.000000247
0	0	0
0	0	0
0	0	0
0	0	0
0	0	0
0	0	0
0	0	0
0	0	0
0	0	0
0	0	0
0	0	0
0	0	0
0	0	0
0	0	0
0	0	0
0	0	0
0	0	0
0	0	0
0	0.000000283	0
0	0	0
0	0	0
0	0	0
0	0	0
0	0	0
0	0	0
0	0	0
0	0	0
0	0	0
0	0	0
0	0	0
0	0	0
0	0	0
0	0	0.000000234
0	0	0
0	0	0
0	0	0
0	0	0

0	0	0
0	0	0
0.00000033	0	0
0	0	0
0	0	0
0.000000539	0	0
0	0	0.00000289
0	0	0
0	0	0
0	0	0
0	0	0
0	0	0
0	0	0
0	0	0
0	0	0
0	0	0
0	0	0
0	0	0
0	0	0
0	0	0
0	0	0
0	0	0
0	0	0
0	0	0
0	0	0
0	0	0
0	0	0
0	0	0
0	0	0
0	0	0
0	0	0
0	0	0
0	0	0
0	0	0
0	0	0
0	0	0
0	0	0
0.00000712	0.00000219	0.0000215
0	0	0
0	0	0
0	0	0
0	0	0
0	0	0
0	0	0
0	0	0
0	0	0
0	0	0

0	0	0
0	0	0
0	0	0
0	0	0
0	0	0
0	0	0
0	0	0
0	0	0
0	0	0
0	0	0
0	0	0
0	0	0
0	0	0
0	0	0
0	0	0
0	0	0
0	0	0
0	0	0
0	0	0
0	0	0
0	0	0
0	0	0
0	0	0.00000183
0	0	0
0	0	0
0	0	0
0	0	0
0	0	0
0	0	0
0	0	0
0	0	0
0	0	0
0	0.00000028	0
0	0	0
0	0	0
0	0	0
0	0	0
0	0	0
0	0	0
0	0	0
0	0	0
0	0	0
0	0	0
0	0	0
0	0	0
0	0	0
0	0	0.000000215

	0	0	0
	0	0	0
	0	0	0
	0	0	0
	0	0	0
	0	0	0
	0.000000291	0	0
	0	0	0
	0.000000493	0	0
	0	0	0.0000029
	0	0	0.000000224
	0	0	0
	0	0	0
	0.000000111	0	0
	0	0	0
	0	0	0
	0	0	0
	0	0	0
	0	0	0
	0	0	0
	0	0	0
	0	0	0
	0	0	7.01E-08
	0	0	0.000000104
	0	0	0
	0	0	0
	0	0	0
	0	0	0
	0	0	0
	0	0	0
	0	0	0
	0	0	8.73E-08
	0	0	0
	0	0	0
	0	0	0
	0	0	0
	0	0	0
	0	0	0
	0	0	0
	0	0	0
	0	0	0
	0	0	0
	0	0	0
	0	0	0
	0	0	0
	0	0	0
	0	0	0

0	0	0
0	0	0
0	0	0
0	0	0
0	0	0
0	0	0
0	0	0
0	0	0
0	0	0
0	0	0
0	0	0
0	0	0
0	0	0
0	0	0
0	0	0
0	0	0
0	0	0
0	0	0
0	0	0
0	0	0
0	0	0
0	0	0
0	0	0
0	0	0
0	0	0
0	0	0
0	0	0
0	0	0
0	0	0
0	0	0
0	0	0
0	0	0
0	0	0
0	0	0
0	0	0
0	0	0
0	0	4.89E-08
0	0	0
0	0	0
0	0	0
0	0	0
0	0	0
0	0	0
0	0	0
0.000000547	0	0
0	0	0
0	0	0

0	0	0
0	0	6.53E-08
0	0	0
0	0	0
0	0	0.000000654
0	0	0
0	0	6.02E-08
0	0	0
0	0	0
0	0	8.83E-08
0	0	0
0	0	0
0	0	0
0	0	0
0	0	0
0	0	0
0	0	0
0	0	0
0	0	0
0	0	0
0	0	0
0	0	0
0	0	0
0	0	0
0	0	0
0	0	0
0	0	0
0	0	0
0	0	0
0	0	0
0	0	0
0	0	0
0	0	0
0	0	0
0	0	0
0	0	0
0	0	0
0	0	0
0	0	0
0	0	0
0	0	0
0	0	0
0	0	0
0	0	0
0	0	0
0	0	0
0	0	0

0	0	0
0	0	0
0	0	0
0	0	0
0	0	0
0	0	0
0	0	0
8.21E-08	0	0.000000195
0	0	0
0	0	0
0	0	0
0	0	6.29E-08
0	0	0
0	9.73E-08	0.0000576
0	0	0
0	0	0
0	0	0
0	0	0
0	0	0
0	0	0
0	0	0
0	0	0
0	0	0
0	0	0
0	0	0
0	0	0
0	0	0
0	0	0
0.000000299	0	0
0.000000478	0	0
0	0	0.00000409
0	0	0
0	0	0
0	0	0
0	0	0
0	0	0
0	0	0
0	0	0
0	0	0
0	0	0
0	0	0
0	0	0
0	0	0
0	0	0
0	0	0
0	0	0
0	0	0

0	0	0
0	0	0
0	0	0
0	0	0
0	0	0
0	0	0
0	0	0
0	0	0
0	0	0
0	0	0
0	0	0
0	0	0
0	0	0
0	0	0
0	0	0
0	0	0
0	0	0
0	0	0
0	0	0
0	0	0
0	0	0
0	0	0
0	0	0
0	0	0
0	0	0
0	0	0
0	0	5.48E-08
0	0	0
0	0	0
0	0	0
0	0	0
0	0	0
0	0	0
0	0	0
0	0	0
0	0	0
0	0	0
0.000000205	0	0
0.00000025	0	0
0	0	0.0000031
0	0	0
0	0	0
0	0	5.75E-08
0	0	0.000000359
0	0	0
0	0	0
0	0	0

0	0	0
0	0	0
0.00000681	0.00000215	0.0000209
0	0	0
0	0	0
0	0	0
0	0	0
0	0	0
0	0	0
0	0	0
0	0	0
0	0	0
0	0	0
0	0	0
0	0	0
0	0	0
0	0	0
0	0	0
0	0	0
0	0	0
0	0	0
0	0	0
0	0	0
0	0	0
0	0	0.0000314
0	0	0
0	0	0
0	0	0
0	0	0
0	0	0
0	0	0
0	0	0
0	0	0
0	0	0
0	0	0
0	0	0
0	0	0
0	0	0
0	0	0
0	0	0
0	0	0
0	0	0
0	0	0
0	0	0.000000145
0	0	0
0	0	0.000000167

0.000000128	0	0.000000731
0	0	0.000000485
0	0	0
0	0	3.52E-08
0.00000173	0.00000104	0.00000155
0	0	0.000000047
0.00000466	0.00000151	0.00000198
0	0	0
0	0	0
0	0	8.89E-08
0	0	0
0	0	0.000000102
0	0	0
0	0	0
0	0	0
0	0	0
0	0	0
0	0	0
0	0	0
0	0	0
0	0	6.39E-08
0	0	0
0	0	0
0	0	0
0	0	0
0	0.000000227	0
0.000000328	0	0
0	0	0
0	0	0
0	0	0
0	0	0
0	0	4.57E-08
0	0	0.000000143
0	0	0.000000493
0	0	0.000000176
0	0	0.00000016
0	0	0
0	0	0
0	0	0
0	0	0
0	0	0
0	0	0
0	0	0
0	0	0
0	0	0
0	0	0
0	0	0

0	0	0
0	0	0
0	0	0
0	0	0
0	0	0
0	0	0
0	0	0
0	0	0
0	0	0
0	0	0
0	0	0
0	0	0
0	0	0
0	0	0
0	0	0
0	0	0
0	0	0
0	0	0
0	0	0
0	0	0
0	0	0
0	0	0
0	0	0
0	0	0
0	0	0
0	0	0
0	0	0
0	0	0
0	0	0
0	0	0
0	0	0
0	0	0
0	0	0
0	0	0
0	0	0
0	0	0
0	0	0
0	0	0
0	0	0
0	0	0
0	0	0
0	0	0
0	0	0
0	0	0
0	0	0
0	0	0
0	0	3.84E-08

0	0	0.000000101
0	0	0
0	0	0
0	0	0
0	0	0
0	0	0
0	0	0
0	0	0
0	0	0
0	0	0
0	0	0
0	0	0
0	0	0
0	0	0
0	0	0
0	0	0
0	0	0
0	0	0
0	0	0
0	0	0
0	0	0
0	0	0
0	0	0
0	0	0
0	0	0
0	0	0
0.000000388	0	0
0	0	0
0	0	0
0.00000124	0	0
0	0	0.00000403
0	0	0
0	0	0.000000261
0	0	0
0	0	0
0	0	0
0	0	0
0	0	0
0.000000525	0	0
0	0	0.00000352
0	0	0
0	0.00000013	0
0	0	0
0	0	0
0	0	0
0	0	0
0	0	0

0	0	0
0	0	0
0	0	0
0	0	0
0	0	0
0	0	0
0	0	0
0	0	8.11E-08
0	0	0
0	0	0
0	0	0
0	0	5.13E-08
0	0	0
0	0	0
0	0	0
0.000000262	0	0
0.000000744	0	0
0	0	0.0000027
0	0	0
0	0	0
0	0	0
0	0	0
0.000000187	0	0.000000185
0	0	0
0	0	0
0.000000313	0	0
0	0	0
0	0	0
0.000000261	0	0.000000801
0	5.52E-08	0
0	0	0
0	0	0
0	0	0
0	0	0
0	0	0
0	0	0
0	0	0
0	0	0
0	0	0
0	0	0
0	0	0
0	0	0
0	0	0
0	0	0
0	0	0
0	0	0
0	0	0

0	0	4.01E-08
0	0	0
0	0	0
0	0	0
0	0	0
0	0	0.000000073
0	0	0
0	0	0
0	0	0
0	0	0
0.000000235	0	0
0.000000664	0	0
0	0	0.00000294
0	0	0
0	0	0
0	0	0
0	0	0
0	0	0
0	0	0
0	0	0
0	0	0
0	0	0
0	0	0
0	0	0
0	0	0
0	0	0
0	0	0
0	0	0
0	0	0
0	0	0
0	0	0
0	0	0
0	0	0
0	0	0
0	0	0
0	0	0
0	0	0
0	0	0
0	0	0
0	0	0
0	0	0
0	0	0
0	0	0
0	0	0
0	0	0
0	0	0
0	0	0

0	0	0
0	0	0
0	0	0
0	0	0
0	0	0
0	0	0
0	0	0
0	0	0
0	0	0
0	0	0
0	0	0
0.00000798	0.00000287	0.0000238
0	0	0
0	0	0
0	0	0
0	0	0
0	0	0
0	0	0
0	0	0
0	0	0
0	0	0
0	0	0
0	0	0
0	0	0
0	0	0
0	0	0
0	0	0
0	0	0
0	0	0
0	0	0
0	0	0
0	0	0
0	0	0
0	0	0
0	0	0
0	0	0
0	0	0
0	0	0
0	0	0
0	0	0
0	0	0
0	0	0
0	0	0
0	0	0
0	0	0
0	0	0
0	0	0

0.000000202	0	0.000000548
0	0	0
0	0	0
0	0	0
0	0	0
0	0	0
0	0	0
0	0	0
0	0	0
0	0	0
0	0	0
0	0	0
0	0	0
0	0	0
0	0	0.0000374
0	0.000000134	0
0	0	0
0	0	0
0	0	0.000000283
0	0	0
0	0	0
0	0	0
0	0	0
0	0	0
0	0	0
0	0	0
0	0	0
0	0	0
0	0.000000411	0
0.0000475	0	0
0	0	0.000000133
0	0	0
0	0	0
0	0	0
0	0	0
0	0	0
0	0	0
0	0	0
0	0	0
0	0	0
0	0	0
0	0	0
0	0	0
0	0	0
0	0	0
0	0	0
0	0	0.000000253

0	0	0
0	0	0
0	0	0
0	0	0
0	0	0
0	0	0
0	0	0
0	0	0
0	0	0
0	0	0
0	0	0
0	0	0
0	0	0
0	0	0
0.000000509	0	0
0	0	0
0	0	0
0.00000117	0	0
0	0	0.00000505
0	0.000000097	0
0	0	0
0	0	0
0	0	0
0	0	0
0	0	0
0	0	0
0	0	0
0	0	0
0	0	0
0	0	0
0	0	0
0	0	0
0	0	0
0	0	0
0	0	0
0	0	0
0	0	0
0	0	0
0	0	0.000000257
0	0	0
0	0	0
0	0	0
0	0	0
0.000000216	0	0
0	0	0
0.000000417	0	0
0	0	0.00000372

0	9.58E-08	0
0	0	0
0	0	0
0	0	0
0	0	0
0	0	0
0	0	0
0	0	0
0	0	0
0	0	0
0	0	0
0	0	0
0	0	0
0	0	0
0	0	0
0	0	0
0	0	0
0	0	0
0	0	0
0	0	0
0	0	0
0	0	0
0	0	0
0	0	0
0	0	0
0	0	0
0	0	0
0	0	0
0	0	0
0	0	0
0	0	0
0	0	0
0	0	0
0	0	0
0	0	0
0	0	0
0	0	0
0	0	0
0.0000083	0.00000256	0.0000231
0	0	0
0	0	0
0	0	0
0	0	0
0	0	0
0	0	0
0	0	0
0	0	0

0	0	0
0	0	0
0	0	0
0	0	0
0	0	0
0	0	0
0	0	0
0	0	0
0	0	0
0	0	0
0	0	0
0	0	0
0	0	0
0	0	0
0	0	0
0	0	0
0	0	0
0	0	0
0	0	0
0	0	0
0	0	0
9.76E-08	0	0.000000284
0	0	0
0	0	0
0	0	0
0	0	0
0	0	0
0	0	0
0	0.000000116	0
0	0	0
0	0	0.000000203
0	0	0
0	0	0
0	0	0
0	0	0
0	0	0
0	0	0
0	0	0
0	0	0
0	0	0
0	0	0
0	0	0
0	0	0
0	0	0
0	0	0
0	0	0
0	0	0

0	0	0
0	0	0
0	0	0
0	0	0
0	0	0
0	0	0
0	0	0
0	0	0.000000274
0	0	0
0	0	0
0	0	0
0	0	0
0	0	0
0	0.000000294	0
0	0	0
0	0	0
0	0	0
0	0	0
0	0	0
0	0	0
0.00000032	0	0
0.000000831	0	0
0	0	0.00000362
0	0.000000097	0
0	0	0
0	0	0
0	0	0
0	0	0
0	0	0
0	0	0
0	0	0
0	0	0
0	0	0
0	0	0
0	0	0
0	0	0
0	0	0
0	0	0
0	0	0
0	0	0
0	0	0
0	0	0
0	0	0.000000257
0	0	0
0	0	0
0	0	0
0	0	0

0.000000216	0	0
0	0	0
0.000000417	0	0
0	0	0.00000372
0	0.0000001	0
0	0	0
0	0	0
0	0	0
0	0	0
0	0	0
0	0	0
0	0	0
0	0	0
0	0	0
0	0	0
0	0	0
0	0	0
0	0	0
0	0	0
0	0	0
0	0	0
0	0	0
0	0	0
0	0	0
0	0	0
0	0	0.000000221
0	0	0
0	0	0
0	0	0
0	0	0
0	0	0
0	0	0
0	0	0
0	0	0.000000072
0	0	0
0	0	0
0.000000287	0	0
0.000000778	0	0
0	0	0.00000386
0	0	0
0	0	0
0	0	0
0	0	0
0	0	0
0	0	0
0	0	0
0	0	0

0	0	0
0	0	0
0	0	0
0	0	0
0	0	0
0	0	0
0	0	0
0	0	0
0	0	0
0	0	7.11E-08
0	0	0
0	0	0
0	0	0
0.000000346	0	0
0.000000757	0	0
0	0	0.00000478
0	0	0
0	0	0
0	0	0
0	0	0
0	0.000000159	0.00000444
0	0	0
5.99E-08	0	0
0	0	0
0	0	0
0	0	0
0	0	0.000000137
0	0	0
0	0	0
0	0	0
0	0	0
0	0	0
0	0	0
0	0	0
0	0	0
0	0.000000257	0
0	9.08E-08	0.0000566
0	0	0
0	0	0
0	0.000000358	0
0	0	0
0	0	0.000000171
0	0	0.000000284
0	0	0
0	0	0
0	0	0
0	0	0

0	0	0
0	0	0
0	0	0
0	0	0
0	0	0
0	0	0
0	0	0
0	0	0
0	0	0
0	0	0
0.000000187	0	0.00000015
0	0	0
0	0	0
0	0	0
0	0	0
0	0	0
0	0	0
0	0	0
0	0	0
0	0	0
0	0	0
0	0	0
0	0	0
0	0	0
0	0	0
0	0	0
0.000000216	0	0.000000227
0	0	0
0	0	0
0	0	0
0	0	0.0000246
0	0	0.000000103
0	0	0
0	0	0
0	0	0.000000247
0	0	0
0	0	0
0	0	0
0	0	0
0	0	0
7.38E-08	0	0
0	0	0
0	0	0
0	0	0
0	0	0
0	0	0
0	0	0

0	0	0
0	0	0
0	0	0
0	0	0
0	0	0
7.87E-08	0	0.00000012
0	0	0.000000103
0	0	0
0	0	0
0	0	0
0	0	0
0	0	0
0	0	0
0	0	0
0	0	0
0	0	0
0.000000957	0	0
0	0	0.00000498
0	0	0.000000279
0	0	0
0	0	0
0	0	0.000000281
0	0	0
0	0	0
0	0	0
0	0	0
0	0	0
0	0	0.000000167
0	0	0.000000157
0	0	0
0	0	0
0	0	0
0	0	0
0	0	0
0	0	0
0	0	0
0	0	0
0	0	0
0	0	0
0	0	0
0	0	0
0	0	0
0	0	0
0	0	0
0	0	0
0	0	0
0	0	0

0	0	0
0	0	0
0	0	0
0	0	0
0	0	0
0	0	0
0	0	0
0	0	0.000000151
0	0	0
0	0	0
0	0	0
0	0	0
0	0	0
0	0	0
6.44E-08	0	0.000000104
0	0	0.000000129
0	0	0
0	0	0
0	0	0
0	0	0
0	0	8.36E-08
0.000000322	0	0
0	0	0
0	0	0
0	0	0
0	0	0
0	0	7.96E-08
0	0	0
0	0	0
0	0	0
0	0	0
0	0	0
0	0	0
0	0	0
0	0	0
0	0	0
0	0	0
0	0	0
0	0	0
0	0	0
0	0	0
0	0	0
0	0	0
0	0	0
0	0	0
0	0	0
0	0	0

0	0	0
0	0	0
0	0	0
0	0	0
0	0	0
0	0	0
0	0	0
0	0	0
0	0	0
0	0	0
0	0	0
0	0	0
0	0	0.000000234
0	0	0
0	0	0
0	0	0
0	0	0
0	0	0
0	0	0
0	0	0
0	0	0
0	0	0
0	0	0
0	0	0
0	0	0
0.000000459	0	0
0	0	0
0	0	0
0	0	0
0	0	0
0	0	0
0	0	0
0	0	0
0	0	0
0	0	0
0	0	0
0	0	0.000000181
0	0	0
0	0	0
0	0	0
0	0	0
0	0	0
0	0	0
0	0	0
0	0	0
0	0	0
0	0	0

0	0	0
0	0	0
0	0	0
0	0	0
0	0	0
0	0	0
0	0	0
0	0	0
0	0	0
0	0	0
0	0	0
0	0	0
0	0	0
0	0	0
0	0	0
0	0	0
0	0	0
0	0	0
0	0	0
0	0	0
0	0	0
0	0	0
0	0	0
0	0	0
0	0	0
0	0	0
0	0	0
0	0	0
0	0	0
0	0	0
0	0	0
0	0	0
0	0	0
0	0	0
0	0	0
0	0	0
0	0	0
0	0	0
0	0	0
0	0	0
0	0	0
0	0	0
0	0	0
0	0	0
0	0	0
0	0	0
0	0	0

0	0	0
0	0	0
0	0	0.000000127
0	0	0
0	0	0
0	0	0
0	0	0
0	0	0
0	0	0
0	0	0
0	0	0
0	0	0
0	0	0
0	0	0
0	0	0
0	0	0
0	0	0
0	0	0
0	0	0
0	0	0
0	0	0
0	0	0
0	0	0
0.000000254	0	0
0	0	0
4.31E-08	0	0
0	0	0.00000283
0	0	0.000000284
0.000000572	0.000000468	0.0000016
0	0	0
0	0	0
0.000000377	0.000000967	0.0000026
0	0	8.05E-08
0	0	0
0	0	0
0	0	0
0	0	0
0	0	0
0	0	0
0	0	0
0	0	0.000000114
0	0	0
0	0	0
0	0	0
0	0	0
4.82E-08	0	0
0	0	0

0	0	0.00000343
0	0	0
0	0	3.43E-08
0	0	0
0	0	0
0	0	0
0	0	0
0	0	0
0	0	0
0.000000639	0	0
0	0	0.00000326
0	0	0
0	0	5.56E-08
0.00000186	0.000000668	0.00000205
0	0	0
0.000000134	0	9.62E-08
0	0	0
0	0	0
0	0	0
0	0	0
0	0	0
0	0	0
0	0	0
0	0	0
0	0	0
0	0	0
0	0	0
0	0	0
0	0	0
0	0	0
0	0	0
0	0	0
0	0	0
0	0	0
0	0	0.000000128
0	0	0
0	0	0
0	0	0.000000624
0	0	0
0	0	0
0	0	4.25E-08
0	0	0
0	0	4.25E-08
0	0	0
0	0	0
0	0	0
0	0	0

0	0	0
0	0	0
0	0	0
0	0	0
0	0	0
0	0	0
0	0	0
0	0	0
0	0	0
0	0	0
0	0	0.00000022
0	0	0
0.00000044	0	0
0	0	0
0	0	0
0	0	0
0	0	0
0	0	0
0	0	0
0	0	0
0	0	0
0	0	0
0	0	0
0	0	0
0	0	0
0	0	0.0000364
0	0	0
0	0	0
0	0	0
0	0	0.000000131
0	0	0
0	0	0
0	0	0
0	0	0
0	0	0
0	0	0
0	0	0
0	0	0
0	0	0
0	0	0
0	0	0
0	0	0
0	0	0
0	0	0
0	0	0
0	0	0
0	0	0

0	0	0
0	0	0
0	0	0
0	0	0
0	0	0
0	0	0
0	0	0
0	0	0
0	0	0
0	0	0
0	0	0
0	0	0
0	0	0
0	0	0
0	0	0
0	0	0
0	0	0
0	0	0
0	0	0
0	0	0
0	0	0.000000223
0	0	0
0	0	0
0	0	0
0	0	0
0	0	0
0	0	0
0	0	0
0	0	0
0	0	0
0	0	0
0	0	0
0	0	0
0.000000196	0	0.000000242
0	0	0
0	0	0
0	0	0.000000111
0	0	0
0	0	0
0	0	0.000000309
0	0	0
0	0	0
0	0	0
0	0	0
0	0	0
0	0	0
0	0	0

0	0	0
0	0	0
0	0	0
0	0	0
0	0	0
0	0	0
0	0	6.95E-08
0	0	0
0	0	0
0	0	0
0	0	0
0	0	0
0	0	0
0	0	0
0	0	0
0	0	0.000000225
0	0	0
0	0	0
0	0	0
0	0	0
0	0	0.0000001
0	0	0
0	0	0
0	0	0
0	0	0
0	7.76E-08	0.00000014
0	0	0
0	0	0
0	0	0
0	0	0
0	0	0
0	0	0
0	0	0
0	0	0
0	0	0
0	0	0
0	0	0
0	0	0
0	0	0
0	0	0.000000121
0	0	0
0	0	0
0	0	0
0	0	0
0	0	0
0	0	0
0.00000667	0.00000213	0.0000184

0	0	0
0	0	0
0	0	0
0	0	0
0	0	0
0	0	0
0	0	0
0	0	0
0	0	0
0	0	0
0	0	0
0	0	0
0	8.82E-08	0.0000525
0	0	0
0	0	0
0	0	0
0	0.000000317	0
0.000000292	0	0
0.00000015	0	0
0	0	0.00000211
0	0	0.000000357
0	0	0
0	0	0
0	0	0
0	0	0
0	0	0
0	0	0
0	0	0
0	0	0
0	0	0
0	0	0
0	0	0
0	0	0
0	0	0
0	0	0
0	0	0
0	0	4.89E-08
0	0	0.000000368
0	0	0
0	0	0
0.0000056	0.00000182	0.0000168
0	0	0
0	0	0
0	0	0
0	0	0
0	0	4.68E-08
0	0	0

0	0	0.0000363
0	0	0.000000114
0	0.000000502	0
0	0	0
0	0	0
0.000000234	0	0
0	0	0
0	0	0
0	0	0
0	0	0
0	0	0
0	0	0
0	0	0
0	0	0
0	0	0
0	0	0
0	0	0
0	0	0
0	0	0
0	0	0
0	0	0
0	0	0
0	0	0
0	0	0
0	0	0
0	0	0
0	0	0
0	0	0
0	0	0
0	0	0
0	0	0
0	0	0
0	0	0
0	0	0
0	0	0
0	0	0
0	0	0
0	0	0
0	0	0
0	0	0
0	0	0
0	0	0
0	0	0
0	0	0
0	0	0
0	0	0
0	0	0

	0	0	0
	0	0	0
	0	0	0
	0	0	0
	0	0	0
	0	0	0
	0	0	0
	0	0	0
	0	0	0
	0	0	0
	0	0	0
	0	0	0
	0	0	0
	0	0	0
	0	0	0
	0	0	0
	0	0	0
	0	0	0
	0	0	0
	0	0	0
	0	0	0
	0	0	0.000000143
	0	0	0.000000159
	0	0	0
	0	0	0
	0	0	0
	0	0	0
	0	0	0
	0	0	0
	0	0	0
	0	0	0
	0	0	0
	0	0	0
	0	0	0
	0	0	0
	0	0	0
	0	0	0
	0	0	0
	0	0	0
	0	0	0
	0	0	0
	0	0	0
	0	0	0
	0	0	0
	0	0	0
	0	0	0
	0	0	0

0	0	0
0	0	0
0	0	0
0.000000388	0	0
0	0	0
0	0	0
0.00000124	0	0
0	0	0.00000403
0.000000233	0	0.000000154
0.000000142	0	0.000000107
0.00000824	0.00000258	0
0.0000676	0.0000278	0.000140824
0.00000014	0	0.000000106
0	0	0
0.000169727	0.0000861	0.000144802
0.000000827	0.000000998	0.00000225
0.000420345	0.00061002	0.000574406
0	0	0.000000435
0.00000106	0.00000106	0.00000384
0.00000791	0.00000238	0
0.000000236	0	0.00000111
0	0.000000387	0.000231694
0	7.04E-08	0.000000325
0.000215966	0.000142676	0.000338021
0.000000139	0	0.000000964
0.000000917	0.000000998	0.000000217
0	0	0
0	0	0
0.0000927	0.0000188	0.000117001
0	0	0
0	0	0
0.00000829	0.00000272	0
0.00000733	0.0000306	0
0.00000111	0.00000112	0.00000182
0.000316582	0.000168352	0.000296683
0.000000147	0	0.000000109
0.00000777	0.0000023	0
0.0000789	0.000124418	0.0000027
0.000000142	0	0.000000113
0.000000597	0.00000031	0.000000996
0.00000786	0.00000255	0
0.000118371	0.000176411	0.0000254
0.00000022	0.000000117	0.000000113
0.000000167	0	4.36E-08
0.000000224	0	0.000000169
0.0001191	0.0000667	0.000102195
0.0000788	0.0000615	0.000093

0.000000654	0.00000135	0
0.0102177	0.00305058	0
0.000000476	0.000000781	0.00000134
0	0	0
0	0	0
0.000000197	0	0.000000103
0.00000035	0.000000565	0.000000765
0.000138508	0.000190054	0.000222707
0.000000193	0	0.000000204
0.000000117	0	0.000000111
0.0000972	0.0000483	0.000310063
0.000258498	0.000334497	0.000439114
0.00000116	0.000000925	0.0000019
8.91E-08	0.000000192	0.000000227
0.0000571	0.0000335	0.000156398
0.000000847	0.00000116	0.00000214
0	0	0
0.00000502	0.00000157	0.00000204
0.000210529	0.000113968	0.000158182
0.00000018	0	0.000000138
0	0.0000759	0
0	6.05E-08	0
0	6.05E-08	0
0.000145027	0.000133972	0.000393412
0.000337556	0.00042977	0.000337981
0.000000191	0	0.000000154
0	0	0.000000726
0.00000166	0.00000274	0.00000429
0.000000853	0.00000108	0.00000225
0.000251719	0.00019481	0.000331702
0.00000023	0	7.75E-08
0	0	0
0.000000143	0	0.000000164
0.00000018	0	0.000000121
0.00000018	0	0.000000121
0	0.000000298	0.00000124
0.000000134	0	0.000000179
0.00000057	0	0.000000483
0.000378629	0.000423669	0.000364629
0	0	0
0.000016	0.0000103	0.00000164
0	5.48E-08	0
0	0.000000213	0.000000164
0	0	0.000000667
0.000150008	0.0000768	0.000358324
0.0000197	0.000000425	0
0	0	0

0	0	0
0.000292578	0.000377365	0.000299235
0.000444138	0.000549848	0.000578065
0.000000765	0.000000629	0.000000684
0	0	0
0	0.000000106	0
0.000000169	0	0.000000168
0.000130737	0.0000668	0.000146189
0.000000593	0	0.000000591
0	0	0
0.000306185	0.000400975	0.000323146
0.000000149	0	0.000000121
0.000002	0.00000253	0.00000353
0.000000508	0.000000679	0.000000779
0.000000517	0.000000428	0.000000477
0.000000151	0	0.00000012
0.000000227	0	0.000000105
0.000000227	0	0.000000105
0.00000226	0.00000143	0.00000201
0	0	0
0	0	0
0.000000185	0	9.69E-08
0.000000227	0	0.000000108
0.00000168	0.00000106	0.00000213
0	0	0
0.0000209	0.00000239	0.000000255
0	0	0.000000267
0.000000145	0	0.000000126
0.000308624	0.000231155	0.000385242
0.000000155	0	0.000000131
0.000000152	0	0.00000005
0.000000144	0	0.000000116
0.000000168	0	7.58E-08
0	0.000000101	0
0.00000082	0.000000284	0.000000959
0.000000181	0	0.000000302
0.000000134	0	6.19E-08
0	0	0
0.00000016	0	0.000000106
0.000106494	0.0000397	0.000367225
0.000383666	0.000518238	0.00053767
0.000383666	0.000518238	0.00053767
0.000000125	0	0.000000164
0	0	0
0	0	0
0.000148357	0.000158816	0.0000284
4.86E-08	0	0.000000118

0.000000191	0	9.04E-08
0.00000012	0	0.000000187
0.000000142	0	0.000000116
0.000000179	0	5.45E-08
0.00000018	0	9.43E-08
0	0	0
0.000000103	0	5.97E-08
0.0000222	0.000000547	0
0	0	0
0.000000157	0	0.000000142
0	0	0.00000114
0.00000674	0.00000154	0.00000376
0.000000109	0	6.25E-08
0.000000175	0	0.000000104
0.000000152	0	0.000000111
0.0000002	0	0.000000256
0.000171938	0.00013196	0.000188475
0	0	0
0	0	0
0.000037	0.0000131	0.00012376
0.00021487	0.000253454	0.000173024
0	0	0
0.000000139	0	9.81E-08
0	0	0
0.000000151	0	0.000000105
0	0	0.000000995
0.000000141	0	0.000000101
0.000000141	0	0.000000101
0.000149409	0.0000683	0.000242907
0.0000134	0.000000335	0
0.00000105	0.00000323	0.00000141
0.000000197	0	7.67E-08
0.215546	0.224667	0.20925
0.0000132	0.00000025	0
0	0	0
0.000000781	0.000000857	0.00000972
0	0	0
0.000124011	0.0000456	0.000164762
0.000000265	0	8.07E-08
0	0	0
0	0	0
0	0	0
0.00000348	0.00000476	0.00000766
0	0	0
0	0	0.000000331
0	0	0
0.000100597	0.0000358	0.000327077

0.000246597	0.000201074	0.000424271
0	0	0
0.00000467	0.00000153	0.00000203
0.000000069	0	5.44E-08
0	0	0
0	0	0
0.000000172	0	0
0.0000021	0.00000532	0.00000246
0	0	8.14E-08
0	0	0
0.000133121	0.0000465	0.000195568
0	0	0
0	0	0
0.000173987	0.000156603	0.000231421
0	0	0
0	0	0
0	0	6.69E-08
0.000188891	0.000276559	0.000334804
0.0000207	0.0000101	0.00000877
0.00000252	0.00000247	0.00000335
0	0	0
0	0	0
0	0	0
0	0	0
0	0	0
0.000000643	0.000000761	0.000000911
0.00012183	0.0000437	0.000174397
0	0	0
0	0	0
0.0000002	0	7.75E-08
0	0.00000148	0.00000123
0.00000174	0.00000108	0.00000254
0	0	0
0	0	0
0	0	0
0.0000101	0.000000173	0
6.82E-08	0	7.69E-08
0.00010455	0.0000399	0.000109094
0	0	0
0	0	0
0	0	0
0	0	0
0	0	0
0	0	0
0.000000172	0	0.000000272
0.00038235	0.000341672	0.000433688
0.0000778	0.000129632	0.00000745

0	0	0.000000461
0	0	0
0	0	0
0.00000668	0.00000494	0.000000108
0	0	0
0	0	0
0	0	0
0	0	0
0	0	0
0	0	0
0	0	0
0.0000101	0.000000173	0
0.00000882	0.000000169	0
0	0	0
0	0	0
0	0	0
0.000000103	0	0
0	0	0
0.0000107	0.000000204	0
0.00000224	0.00000241	0.00000346
0.000000108	0	0
0.000305617	0.000342979	0.000332251
0	0	0
0.00000157	0.00000153	0.00000113
0	0.00000178	0.00000147
0	0	0
0	0	0
0	0	0
0	0	0
0	0	0
0	0	0
0	0	0
0	0	0
0	0	0
0.00120316	0.00187096	0.00107531
0	0	0
0	0	0
0	0	0
0	0	0
0	0	0
0.000000481	0.000000664	0.000000667
0.00000742	0.00000298	0.00000345
0	0	0
0.00000687	0.000000139	0
0.00000687	0.000000139	0
0.00000249	0.00000054	0.00000196
0	0	0

0	0	0
0	0	0
0	0	0
0	0	0
0	0	0
0.00589356	0.00102947	0
0.00000672	0.000000139	0
0	0.000000362	0.000000196
0.00000103	0.00000113	0.00000143
0.00000163	0.00000113	0.000000923
0	0	0
0	0	0
0.0000841	0.0000343	0.000093
0	0	0
0.00000683	0.00000908	0.00000714
0.000366288	0.000397235	0.000346705
0.000366105	0.00039717	0.000346665
0	0	0
0.00000035	0.000000409	0.00000169
0	0	0
0	0	0.000000824
0.0000363	0.0000234	0.0000874
0.00000286	0.00000185	0.00000282
0	0	0
0.00000141	0.00000133	0.00000136
0	0	0
0.000181354	0.00010409	0.000346734
0	0	0
0.00000867	0.00000283	0.00000313
0	0	0
0.0000559	0.0000304	0.0000666
0	0	0
0.00000435	0.0000025	0.00000796
0.000110326	0.0000525	0.000237795
0	0	0
0.0000117	0.00000312	0.00000217
0	0	0
0.0000002	0.000000563	0.00000239
0.00000861	0.00000322	0.00000331
0.0000806	0.0000403	0.000156979
0	0	0
0	0	0
0.0000564	0.0000269	0.0000403
0	0	0
0	0	0
0	0	0
0	0	0

0.000000426	0.00000111	0.00000313
0	0	0
0	0	0
0	0	0
0.00000314	0.00000245	0.00000101
0	0.000000298	0
0	0	0
0	0	0
0.0000599	0.0000197	0.000148481
0	0	0
0	0	0
0	0	0
0.000000516	0.00000161	0.00000428
0.0000789	0.000121174	0.00000684
0	0	0
0	0	0
0.0000307	0.0000547	0.00000363
0.000044	0.0000329	0.0000515
0.000167721	0.000093	0.000337047
9.44E-08	0	0.000000132
0	0	0
0.0000681	0.0000314	0.0000522
0.00000794	0.00000664	0.0000133
0	0	0
0.0000047	0.00000676	0.00000537
0	0	0
0.000127226	0.000130887	0.000272775
0.000000251	0.000000386	0.000000316
0	0	0
0	0	0
0	0	0
0	0	0
0	0	0
0.0000679	0.000033	0.0000933
0.0000268	0.0000125	0.00000972
0	0	0
0.0000121	0.00000284	0.00000206
0	0	0
0.00000415	0.00000594	0.0000076
0	0	0
0	0	0
0	0	0
0	0	0
0.000000141	0.000000492	0.00000101
0	0	0
0	0	0
0	0	0

0	0	0
0.0000039	0	0
0.00000207	0.00000298	0.00000561
0	0	0
0	0	0
0	0	0
0	0	0
0	0	0
0.00010863	0.0000539	0.000190723
0	0	0
0.00014648	0.0000737	0.000306841
0	0	0
0	0	0
0	0	0
0.00000127	0.000000987	0.00001
0.0000154	0.0000246	0.00000477
0	0	0
0.000000679	0.00000088	0.00000115
0	0	0.000000338
0	0	0
0.00009	0.0000439	0.0000676
0.00000645	0.00000244	0.00000333
0.0000178	0.00000155	0.0000618
0	0	0
0	0	0
0.00000345	0.00000349	0.0000019
0	0	0
0	0	0.000000795
0	0	0
0	0	0
0.00000432	0.00000195	0.00000175
0	0	0
0	0	0
0	0	0
0.000000612	0.000000645	0.00000418
0	0	0
0	0	0
0.000000115	0.000000501	0.00000114
0.000000604	0.000000966	0.0000018
0	0	0
0	0	0
0	0	0
0.0000759	0.0000496	0.0000591
0	0	0
0	0	0
0.0000759	0.0000406	0.0000582
0	0	0

0	0	0
0	0	0
0	0	0
0.0000813	0.00012747	0.00000761
0	0	0
0	0	0
0	0	0
0	0	0
0	0	0
0	0	0
0	0	0
0	0	0
0.037626	0.0365606	0.0227863
1.64E-08	4.85E-08	3.43E-08
0.00000328	0.00000345	0.00000261
0.00000253	0.00000182	0.00000166
0	0	0
0	0	0
0	0	0
0	0	0
0	0	0
0	0	0
0	0	0
0	0	0
0	0	0.000000314
0	0	0
0.00000111	0.00000123	0.000000678
0	0	0
0	0	0
0	0	0
0	0	0
0	0	0.00000134
0	0	0
0	0	0
0	0	0
0.00000454	0.00000352	0.0000038
0.00000808	0.00000445	0.00000174
0	0	0
0	0	0
0	0	0
0	0	0
0.0000419	0.0000199	0.0000864
0	0	0
0.000000172	0.000000139	0.000000256
0	0	0
0	0	0
0	0	0

0.0000547	0.000026	0.0000472
0.000000743	0	0.00000132
0.000003	0.00000366	0.00000541
0	0	0
0	0	0
0.00010936	0.0000738	0.000017
0.0000697	0.0000324	0.0000517
0	0	0
0	0	0
0	0	0
0.00000121	0.000000362	0.00000151
0.0000115	0.00000171	0.0000112
0.00000786	0.0000025	0
0	0	0
0	0	0
0.000000242	0.000000221	0.000000237
0.00000675	0.00000902	0.00000491
0	0	0
0.0000582	0.0000257	0.0000444
0.000027	0.0000389	0.00000578
0	0	0
0.000000122	0	0
0	0	0
0.00000508	0.00000479	0.00000464
0	0	0
0.00000193	0	0
0.000008	0.00000562	0.0000089
0.00000279	0.00000278	0.00000245
0	0	0
0.00000496	0.00000507	0.00000341
0	0	0
0.00000563	0.00000371	0.00000376
0.00000284	0.00000173	0.00000356
0.0000179	0.0000311	0.00000485
0.0000143	0.00000463	0.000000507
3.91E-08	0.000000117	0
0	0	0
0	0	0
0	0	0
0.000000245	0.000000795	0.00000148
0	0	0
0.00000785	0.00000201	0.00000221
0	0	0
0	0	0
0.000017	0.00000986	0.00000547
0	0	0
0	0	0

0.0000892	0.0000375	0.0000572
0.00000357	0.00000174	0.000016
0.0000381	0.0000246	0.00000639
0	0	0
0	0	0
0.0000175	0.0000235	0.00000578
0.00000342	0.00000204	0.00000417
0	0	0
0.00000148	0.000000278	0.00000526
0.0000628	0.00001	0.0000101
0.000000177	0.000000172	0
0.0000089	0.0000072	0.00000457
0	0	0.000000146
0.00000361	0.00000234	0.00000366
0	0	0
0	0	0
0.0000726	0.0000332	0.000103814
0	0	0
0	0	0
0	0	0
0	0	0
0	0	0
0	0	0
0.0000031	0.00000282	0.00000258
0	0	0
0	0	0
0	0	0
0.000000738	0.00000107	0.00000163
0.000000273	0.000000922	0.00000119
0	0	0
0.000000222	0.0000004	0.000000304
0	0	0
0	0	0
0.0000283	0.0000162	0.00000656
0	0	0
0	0	0
0	0	0
0	0	0
0.00000499	0.00000132	0.00000206
0	0	0
0.00000177	0.000000454	0.00000041
0.00000244	0.00000156	0.00000125
0	0	0
0.000000219	0.000000349	0.000000314
0	0	0
0.0000328	0.0000289	0.00000448
0	0	0

0	0	0
0.000000218	0.000000268	0.000000337
0	0	0
0	0	0
0	0	0
0.000000676	0.000000801	0.00000024
0.000000192	0.000000342	0.000000255
0.000000191	0.000000351	0.000000268
0.00000354	0.00000324	0.00000205
0.000000192	0.000000339	0.000000249
0.000000156	0.00000026	0.000000213
0	0	0
0.00000331	0.00000166	0.000000998
0	0	0
0.00000159	0.00000118	0.0000007
0	0	0
0.000000195	0.000000381	0.000000274
0.000000201	5.61E-08	0.000000112
0.000648079	0.0000959	0.000092
0	0.000000564	0
0	0.000000564	0
0	0	0
0	0	0
0	0	0
0.000000208	0.000000276	0.000000155
0.000000208	0.000000276	0.000000155
9.51E-08	2.91E-08	0.000000139
0	0	0
0.000000167	0.000000316	0.000000195
0.00000355	0.00000323	0.00000147
0	0	0
0.00000014	8.13E-08	0.000000122
0.00000014	8.13E-08	0.000000122
4.39E-08	6.62E-08	9.65E-08
0	0.000000365	0
0.000000124	0.000000274	0.000000283
0.000000164	0.000000074	8.81E-08
0.00000846	0.00000296	0.00000957
0	0	0
0	0	0
0	0.000000674	0
0.000000179	5.73E-08	0.000000117
0.000000184	0.000000165	0.000000225
0	0	0
0.000000186	5.86E-08	0.0000001
0.00000182	0.00000133	0.00000123
0.00000513	0.00000426	0.0000117

0.000000182	6.13E-08	0.00000012
0.000509391	0	0
0.000000206	0.000000352	0.000000251
0.000000232	7.48E-08	0.000000124
0.000000232	7.48E-08	0.000000124
0	0	0
0.000000163	0.000000105	0.000000114
0.000000326	0.000000371	0.00000412
0	0.000000464	0
0	0.000000464	0
0.000000201	9.62E-08	0.000000145
0.000000129	0.000000212	0.000000115
0.00000184	0.00000135	0.00000102
0	0	0
0	0.000000583	0
0.000000124	6.63E-08	7.02E-08
0	0	0
0.000000141	0	8.01E-08
0.00000265	0.00000193	0.00000278
0.000000125	0	0
0.00000171	0.00000113	0.00000103
0	0	0
0.000000322	0	0
0	0	0
0	0	0
0.000000411	0.000000588	0.00000177
0.00000178	0.00000244	0.00000121
0.000000109	5.53E-08	8.21E-08
0.0000241	0.0000182	0.00000532
0.0000182	0.0000242	0.00000673
0.00000386	0.00000304	0.00000484
0.000128222	0.000112074	0.00012792
0	5.07E-08	0.000000106
0	0	0
0	0	0
0	0	0
0.000000108	6.94E-08	0.000000086
0.000000121	7.29E-08	0.000000102
0.00000014	0	3.82E-08
0	0	0
0	0	0
0	0	0
0	0	0
0.000000253	3.14E-08	0.000000154
0.000000118	4.69E-08	0.000000106
0.000000194	0	0
0.0000252	0.0000188	0.00000628

0.00000312	0.00000201	0.00000599
0.000000357	0.000000145	0
0.00000388	0.00000311	0.0000134
9.08E-08	0	5.55E-08
0	0	0
0	0	0
0	0.000000165	0
0	0	0
0.000000245	0	0
0	0	0
0.0000231	0.0000163	0.00000536
0	0	0
0.000000158	0	0
0	0	0
0.000000221	0	0
0.000000206	0	0
0.000000106	0	0.000000086
0.0000141	0.0000113	0.0000136
0.000000249	0.000000146	0.000000567
0	0	0
0	0	0
0.0000296	0.0000215	0.0000109
0.0000296	0.0000215	0.0000109
0	0	0
0.0000103	0.0000118	0.00000725
0	0	0
0	0	0
0	0.000000232	0
0.000000145	0	0
0	0.00000023	0
0.000000213	0	0
0.000000323	0.00000104	0.000000849
0	0	0
0.0000002	0	0
0.00000438	0.00000553	0.00000544
0	0	0
0.000000122	0	0
0.000000196	0	0
0	0	0
0	0.000000218	0
0.000000199	0	0
0.00000187	0.00000804	0.00000185
0.0000172	0.00000625	0.00000706
0.0000172	0.00000624	0.00000706
0.00000178	0.00000102	0.0000031
0	0.000177032	0
0.000000166	0	0

0.000000166	0	0
0	0.000000332	0
0	0.000000267	0
0.000000969	0.000001	0.000000963
0	0.000000254	0
0	0.000000254	0
0	0	0
0.000000179	0	0
0	0	0
0.000000162	0	0
0.00000019	0	0
0	0	0
0	0	0
0	0	0
0	0.000000185	0
0	0	0
0.000000151	0	0
0	0	0
0	9.11E-08	0
0	0	0
0	0	0
0	0.000000196	0
0.000000195	0.000000412	0.00000163
0	0	0
0	0	0
0	0	0
0	0.000000186	0
0	0	0
3.06E-08	0.000000297	0.00000167
0	0.000000216	0
0.0000147	0.00000432	0.0000086
0.0000147	0.00000432	0.0000086
0	0	0
0.0000154	0.00000459	0.00000847
0	0	0
0	0	0
9.07E-08	0	0
0	0.000000159	0
0	8.99E-08	0
0	0.000000237	0
0	0	0
0	0	0
0	0.000000139	0
0	0	0
0	0	0
0.000000102	0	0
0	0	0

0	0	0
0.000000112	0	0
0.00000344	0.00000327	0.00000369
0	0	0
0.00000607	0.00000268	0.00000336
0	0	0
0	0.000000121	0
0	0.000000188	0
0.000683203	0	0
0.00001	0.00000992	0.00000394
0	0.000000151	0
0	0	0
0.00000107	0.00000168	0.00000594
0	0	0
0.000000493	0.000000352	0.0000319
0	0.000000157	0
0	0.000000147	0
0.00000762	0.00000304	0.00000839
0.0000127	0.0000072	0.00000455
0	0.000000129	0
0.000000616	0.000000604	0.000000717
0.0000014	0.000000394	0.000000418
0.000000101	0	0
0.000000479	0.00000047	0.000000557
0.000000135	0	0
0.00000612	0.00000797	0.00000345
0	0.0000016	0
0.00000754	0.00000429	0.0000078
0	9.33E-08	0
0.000000585	0.000000987	0.000000715
0	0	0
0.0000203	0.00000897	0.0000159
0	0.000000155	0
0.00000171	0.000000715	0.00000647
0.0000978	0	0
0.0000146	0.00000453	0.000000137
5.36E-08	0.000000209	0.000000182
0	0.00000017	0
0.0000114	0.00000273	0.00000471
0.00000406	0.00000335	0.0000121
0	5.76E-08	0
0.00000115	0.000000394	0.000000281
0.00000176	0.000000211	0.00000131
9.51E-08	0	0
8.08E-08	0	0
0.000000119	0	0
0.0000137	0.00000328	0.00000314

0.00000555	0.00000248	0.00000532
0.000000111	0	0
7.22E-08	0	0
0.000000195	0	0
0.000000114	0	0
0.00000569	0.00000234	0.0000052
0.000000155	0	0
7.84E-08	0	0
0	0.000000111	0
0.000000227	0	0
0	6.61E-08	0
0.00000018	0.000000143	0.0000013
0.000000215	0	0
0.000000101	0	0
8.47E-08	0	0
0.000000191	0	0
0.000000106	0	0
7.89E-08	0	0
0	0	0
8.12E-08	0	0
7.66E-08	0	0
0.000000181	0	0
7.36E-08	0	0
0.000000307	0	0.000000187
0.000000151	0	0
0.000000221	0	0
3.77E-08	0	0
0.000000186	0	0
0.0000532	0.000022	0.0000107
9.85E-08	0	0
0.000000179	0	0
0.000000285	0	0
0.000000285	0	0
0.000000267	0	0
0.000000245	0	0
0.000000216	0	0
3.16E-08	0	0
3.16E-08	0	0
0.000000144	0	0
0.000000223	0	9.06E-08
0.000000176	0	0
0.000000159	0	0
0.00000013	0	0
0.000000128	0	0
0.000000128	0	0
0.000000216	0	0
7.44E-08	0	0

4.37E-08	0	0
0.000000133	0	0
0.000000257	0	0.000000136
0.000000285	0	0
0.000000318	0	0.000000174
0.000000152	0	0
0.000000129	0	0
0.000000262	0	0.000000104
8.97E-08	0	0
8.97E-08	0	0
0	0	0
0.000000287	0	0.000000161
0.000000287	0	0.000000161
6.96E-08	0	0
6.96E-08	0	0
6.91E-08	0	0
0.000000121	0	0
0.000000155	0	0
0.000000116	0	0
2.13E-08	0	0
0.000000138	0	0
0.000000143	0	0
8.71E-08	0	0
0.000000119	0	0
3.33E-08	0	0
0.000000365	0	0.000000219
0.000000111	0	0
0	0	0
0.000000204	0	0.000000194
0.000000106	0	0
4.57E-08	0	0
0.000000187	0	0.000000186
0.000000168	0	0.000000179
0.00000016	0	0
0.00000016	0	0
0.000556614	0	0.000233756
3.69E-08	0	0
0.00000017	0	0.000000169
0.000000056	0	0
0.000000457	0.000000189	0.000000521
0.00000029	0	0.000000217
0.000000101	0	0
0.0000239	0.00000922	0.00000465
0.000000232	0	0.00000018
3.11E-08	0	0
3.11E-08	0	0
0.00000171	0.00000244	0.00000437

0.000000133	0	0
0.000000207	0	0.000000174
0.000000445	0.00000046	0.000001
0	0	0
0.000000188	0	0.000000174
0	0	0
0	0	0
0.00000016	0	0.000000163
0.000000325	0	0.000000126
6.08E-08	0	0.000000131
0.000000134	0	0.000000172
3.25E-08	0	0
0.00000155	0.00000121	0.000000474
3.12E-08	0	0
0.000000246	0	0.000000106
0.000000136	0	0.000000151
0.000000136	0	0.000000151
7.09E-08	0	0
0.00000332	0.00000449	0.00000516
6.65E-08	0	0
5.37E-08	0.00000021	0.000000187
0.000000571	0.0000011	0.000000679
3.11E-08	0	0
0	0	0
3.33E-08	0	0
8.05E-08	0.000000231	0.000000847
0.00000199	0.00000122	0.000000627
0.00000118	0.00000611	0.00000115
0.0000158	0.00000483	0.000000135
0	0	0
0	0	0
0	0	0
0	0	0
0	0	0
0.00000023	0.000000309	0.00000229
0	0	0
0	0	0
0.00000159	0.00000124	0.000000506
0.000000353	0.000000221	0.000000237
0.00000013	0.000000171	0.000000773
0	0	0
0	0	0
0	0	0
0	0	0
6.45E-08	0.000000217	0.000000587
0.000000338	0.000000317	0.00000193
9.45E-08	0.000000194	0.000000634

0	0	0
0	0.000000157	0.000000537
5.41E-08	0	0.00000019

Vanderbilt-patient_Pre-FMT	Vanderbilt-patient_Post-FMT2	Vanderbilt-donor_Donor
0.0000409	0.00013429	0.000000614
0.0000334	0.00010209	0.000000578
0.0000374	0.000125777	0.000000704
0.0000374	0.000125777	0.000000704
0.0000347	0.000115519	0.000000585
0.0000312	0.0000991	0.000000707
0.0000357	0.000114832	0.000000618
0.0000333	0.000110098	0.000000717
0.000178974	0.0000539	0.00000067
0.0000342	0.000113027	0.000000608
0.0000328	0.000110807	0.000000729
0.0000377	0.000124941	0.000000726
0.0000307	0.0000992	0.000000545
0.0000333	0.000112095	0.000000734
0.00020206	0.0000581	0.00000085
0.0255422	0.0816828	0.000480156
0.0000394	0.000126368	0.000000738
0.000255985	0.0000672	0.000000807
0.000200912	0.0000571	0.000000848
0.00025195	0.0000691	0.00000101
0.0000332	0.000113072	0.000000753
0.000197526	0.000056	0.000000886
0.000197615	0.000056	0.000000891
0.0000495	0.000159374	0.00000113
0.0000336	0.000106085	0.000000691
0.0000401	0.00013553	0.000000911
0.00020146	0.0000544	0.000000923
0.00020146	0.0000544	0.000000923
0.0000317	0.000100434	0.000000565
0.0000336	0.00010641	0.000000698
0.0000423	0.000140636	0.000000947
0.0000402	0.000133519	0.000000675
0.0000402	0.000133519	0.000000675
0.0000341	0.000107366	0.000000682
0.0000321	0.000102132	0.000000722
0.000199596	0.0000522	0.000000867
0.0000409	0.000132119	0.000000841
0.0000389	0.000124868	0.000000811
0.0000389	0.000124868	0.000000811
0.0000322	0.000113787	0.000000776
0.0000322	0.000113787	0.000000776
0.000206459	0.0000589	0.000000769
0.0000477	0.00000307	5.94E-08
0.0000486	0.00000784	0.000000215
0.000192473	0.0000484	0.000000856
0.000216503	0.0000584	0.000000909

0.0000436	0.00000485	0.000000107
0.000201687	0.0000588	0.000000992
0.0000506	0.000009	0.000000155
0.0000502	0.00000384	8.04E-08
0.0000304	0.0000955	0.000000691
0.0000153	0.00000274	5.11E-08
0.0000683	0.0000069	0.000000153
0.000223666	0.0000584	0.00000108
0.0000635	0.0000111	0.000000142
0.0000456	0.00000525	8.54E-08
0.0000415	0.00000863	0.000000105
0.00025195	0.0000691	0.00000129
0.00025195	0.0000691	0.00000129
0.0000447	0.0000115	0.000000153
0.0000681	0.00000942	0.000000259
0.000057	0.00000765	0.000000206
0.000042	0.00000783	0.000000179
0.0000308	0.00000701	0.000000147
0.000223666	0.0000584	0.00000115
0.0000365	0.00000443	0.000000118
0.0000339	0.00000659	0.000000197
0.00000674	0.00000443	5.94E-08
0.00000674	0.00000443	5.94E-08
0.0000466	0.0000061	0.000000188
0.0000274	0.000000833	7.61E-08
0.0000274	0.000000833	7.61E-08
0.0000402	0.00000705	0.000000181
0.0000363	0.00000879	0.00000019
0.0000373	0.00000724	0.000000153
0.0000882	0.0000157	0.000000585
0.0000323	0.0000055	0.000000157
0.0000323	0.00000643	0.0000002
0.0000423	0.00000688	0.000000146
0.0000459	0.00000847	0.000000203
0.0000326	0.00000493	0.000000157
0.0000772	0.00001	0.000000351
0.242038	0.0662314	0.0015175
0.0000495	0.0000063	0.000000155
0.0000502	0.0000057	0.000000279
0.000028	0.00000698	0.000000158
0.0000471	0.00000563	0.000000262
0.0000273	0.00000403	9.05E-08
0.0000461	0.00000659	0.000000241
0.0000451	0.00000658	0.000000203
0.0000511	0.00000745	0.000000216
0.00000674	0.00000416	5.47E-08
0.00000674	0.00000416	5.47E-08

0.000031	0.00000713	0.000000171
0.0000292	0.00000553	0.000000152
0.0000449	0.00000238	0.000000143
0.00005	0.0000082	0.000000197
0.0000511	0.00000613	0.000000285
0.0000518	0.00000602	0.000000294
0.0000285	0.00000701	0.000000157
0.0000285	0.00000701	0.000000157
0.0000285	0.00000701	0.000000157
0.0000491	0.00000739	0.00000023
0.0000529	0.00000771	0.000000242
0.0000511	0.00000627	0.000000271
0.0000585	0.0000102	0.00000026
0.0000468	0.00000736	0.000000207
0.0000473	0.00000687	0.000000228
0.0000513	0.00000619	0.000000292
0.0000506	0.00000988	0.000000146
0.00000746	0.000000599	3.24E-08
0.00000911	0.000000754	4.02E-08
0.0000234	0.00000596	0.000000148
0.0000105	0.000000872	4.64E-08
0.0000506	0.00000987	0.000000146
0.00004	0.00000653	0.000000177
0.000032	0.00000342	0.000000154
0.0000517	0.00000657	0.000000211
0.0000517	0.00000657	0.000000211
0.0000209	0.00000682	0.000000153
0.0000209	0.00000682	0.000000153
0.0000209	0.00000682	0.000000153
0.0000209	0.00000682	0.000000153
0.0000359	0.00000751	0.0000002
0.000036	0.00000759	0.000000199
0.0000522	0.00000883	0.000000248
0.0000728	0.0000107	0.000000375
0.0000198	0.00000638	0.000000183
0.0000198	0.00000638	0.000000183
0.0000334	0.00000743	0.000000201
0.00000154	0.000018	0.000000289
0.0000755	0.0000125	0.000000373
0.000037	0.00000534	0.000000182
0.0000511	0.00000736	0.000000254
0.0000274	0.00000633	0.000000136
0.0000718	0.00000941	0.000000379
0.0000254	0.00000706	0.000000164
0.0000809	0.00000684	0.000000215
0.0000379	0.00000566	0.000000179
0.0000779	0.00000718	0.000000383

0.0000779	0.00000718	0.000000383
0.0000322	0.00000664	0.000000226
0.0000739	0.00000988	0.000000274
0.0000255	0.00000509	0.000000115
0.0000414	0.00000715	0.000000231
0.0000393	0.00000602	0.000000186
0.000168865	0.0000224	0.000000839
0.0000598	0.0000103	0.000000287
0.000059	0.0000142	0.000000372
0.00000707	0.00000515	0.000000089
0.0000466	0.00000705	0.000000263
0.0000735	0.000012	0.000000353
0.0000423	0.0000078	0.000000262
0.0000404	0.0000103	0.000000203
0.0000447	0.00000683	0.00000027
0.0000511	0.00000629	0.000000261
0.0000351	0.00000714	0.000000254
0.0000359	0.0000101	0.000000184
0.0000359	0.0000101	0.000000184
0.0000359	0.0000101	0.000000184
0.0000359	0.0000101	0.000000184
0.0000391	0.0000064	0.000000241
0.0000456	0.00000687	0.000000247
0.000058	0.00000894	0.000000276
0.0000483	0.00000782	0.000000208
0.0000809	0.000014	0.00000047
0.0000341	0.00000771	0.000000198
0.0000437	0.00000499	0.000000243
0.0000506	0.00000683	0.000000289
0.0000447	0.00000682	0.000000138
0.0000227	0.00000592	0.000000146
0.0000372	0.0000062	0.000000186
0.0000382	0.00000591	0.000000145
0.0000581	0.00000836	0.000000311
0.0000243	0.00000514	0.000000154
0.0000204	0.0000088	0.00000016
0.0000361	0.0000137	0.000000352
0.0000345	0.0000142	0.000000372
0.0000596	0.0000106	0.000000324
0.0000535	0.0000104	0.000000297
0.0000623	0.0000109	0.000000433
0.0000427	0.00000586	0.000000176
0.0000548	0.00000942	0.000000246
0.0000834	0.0000125	0.000000622
0.00000187	0.00000348	1.75E-08
0.0000457	0.0000069	0.000000199
0.0000351	0.00000478	0.00000017

0.0000388	0.00000451	0.00000018
0.0000464	0.00000707	0.000000204
0.0000559	0.00000673	0.000000329
0.0000134	0.00000415	0.000000102
0.0000552	0.00000882	0.000000273
0.00000208	0.0000034	2.84E-08
0.000041	0.00000701	0.000000288
0.0000664	0.00000997	0.000000372
0.00000249	0.00000388	3.36E-08
0.0000448	0.00000462	0.000000228
0.000071	0.000012	0.000000467
0.000071	0.000012	0.000000467
0.0000409	0.00000725	0.000000269
0.0000445	0.00000747	0.000000308
0.0000372	0.00000604	0.000000186
0.0000218	0.00000154	0.000000127
0.0000623	0.00000892	0.000000331
0.0000259	0.00000545	0.000000147
0.0000424	0.00000585	0.0000002
0.0000259	0.00000523	0.000000147
0.0000671	0.0000105	0.000000408
0.0000599	0.0000103	0.000000359
0.0000245	0.00000625	0.000000165
0.000100581	0.0000186	0.000000597
0.0000398	0.00000532	0.000000174
0.0000457	0.00000683	0.000000283
0.0000108	0.0000047	0.000000209
0.0000351	0.00000551	0.00000021
0.00000492	0.00000409	0.00000011
0.00000492	0.00000409	0.00000011
0.0000534	0.00000968	0.00000035
0.0000534	0.00000968	0.00000035
0.00000474	0.00000557	6.16E-08
0.00000813	0.00000632	0.000000112
0.00000813	0.00000632	0.000000112
0.0000549	0.0000136	0.000000178
0.0000912	0.0000312	0.000000305
0.0000671	0.0000113	0.00000045
0.0000142	0.00000627	0.000000161
0.0000221	0.00000581	0.000000158
0.00781364	0.00587106	0.000680286
0.0000469	0.00000724	0.000000194
0.00000834	0.00000661	0.000000162
0.0000865	0.000013	0.000000573
0.0000128	0.00000417	9.34E-08
0.161764	0.0295579	0.00111286
0.000116347	0.000025	0.000000274

0.0000502	0.0000102	0.000000402
0.0000502	0.0000102	0.000000402
0.0000433	0.00000839	0.000000311
0.0000475	0.00000595	0.000000228
0.0000484	0.00000823	0.000000388
0.0000948	0.0000419	0.000000364
0.0000589	0.00000859	0.000000415
0.0000455	0.00000423	0.000000261
0.0000446	0.00000758	0.000000377
0.000085	0.0000344	0.000000323
0.0000936	0.0000381	0.000000377
0.0000699	0.000029	0.000000269
0.0000763	0.0000255	0.000000586
0.0000472	0.00000736	0.000000379
0.0000599	0.0000215	0.000000414
0.0000331	0.0000066	0.000000274
0.0000983	0.0000441	0.00000038
0.0000149	0.00000222	0.000000102
0.000066	0.0000306	0.000000291
0.000113938	0.0000239	0.000000306
0.0000772	0.000032	0.000000298
0.0000308	0.00000588	0.000000218
0.000117495	0.0000667	0.000000556
0.0000227	0.00000604	0.000000126
0.0000461	0.00000527	0.000000373
0.0656986	0.0310652	0.000315326
0.0000863	0.0000419	0.000000496
0.0000375	0.0000118	5.42E-08
0.0000322	0.00000298	0.000000231
0.0000387	0.0000125	0.000000101
0.0000473	0.0000143	0.000000103
0.000120467	0.0000651	0.000000594
0.000120467	0.0000651	0.000000594
0.0000184	0.00000196	0.000000198
0.000101847	0.0000301	0.000000642
0.000047	0.0000148	0.000000195
0.000102024	0.000054	0.000000478
0.0000394	0.0000115	0.000000122
0.000498387	0.000166471	0.00000491
0.0000948	0.0000346	0.000000361
0.00000338	0.00000461	5.24E-08
0.0000865	0.0000273	0.000000333
0.0000989	0.0000651	0.000000676
0.0000989	0.0000651	0.000000676
0.0000471	0.0000118	0.000000393
0.0000281	0.00000583	0.000000246
0.0000281	0.00000583	0.000000246

0.0000976	0.0000547	0.000000521
0	0.00000156	3.92E-08
0.0000976	0.0000506	0.000000497
0.0000883	0.0000206	0.000000739
0.0000971	0.0000509	0.000000504
0.0000132	0.00000446	0.000000156
0.0000913	0.0000441	0.000000441
0.0000963	0.0000472	0.000000486
0.0000471	0.0000148	0.000000224
0.0000998	0.0000418	0.000000508
0.0000867	0.0000194	0.000000745
0.0000926	0.0000578	0.000000607
0.0000458	0.0000145	0.000000185
0.000095	0.0000604	0.000000649
0.00000852	0.00000687	0.000000176
0.0000401	0.0000122	9.12E-08
0.0000931	0.0000578	0.000000618
0.000110441	0.0000307	0.00000146
0.0000407	0.0000131	0.000000135
0.00000661	0.00000504	0.00000016
0.00000661	0.00000504	0.00000016
0.000658681	0.00221018	0.000374413
0.000111082	0.0000612	0.000000609
0.0000104	0.0000038	4.67E-08
0.0000375	0.000011	0.0000001
0.000053	0.0000349	0.00000392
0.000126945	0.0000234	0.00000141
0.000125479	0.0000367	0.000000477
0	0	0.0000547
0.0000909	0.0000288	0.00000105
0.0000393	0.00000777	0.000000596
0.000051	0.0000149	0.000000174
0.0000776	0.0000233	0.00000132
0.000130996	0.0000276	0.000000931
0.000117683	0.0000337	0.000000951
0.000117683	0.0000337	0.000000951
0.0000927	0.0000206	0.000000872
0.0000108	0.0000037	4.77E-08
0.0000826	0.0000254	0.000000849
0.030555	0.00908273	0.000124272
0.00000996	0.00000712	0.000000272
0.0000363	0.0000105	0.000000466
0.0000174	0.00000596	0.000000216
0.0000422	0.0000132	0.000000166
0.000049	0.00000849	0.000000773
0.000078	0.0000142	0.000000628
0.0000395	0.00000518	0.000000599

0.000133625	0.0000491	0.00000142
0.000133625	0.0000491	0.00000142
0.000133625	0.0000491	0.00000142
0.00000943	0.00000674	0.000000274
0.00000943	0.00000674	0.000000274
0.00031559	0.000123065	0.00000561
0.0000454	0.0000136	0.000000225
0.0000455	0.0000138	0.000000231
0.000000347	0.00000058	0.000000353
0.00000584	0.00000247	2.55E-08
0.0000429	0.0000134	0.000000196
0.0000429	0.0000134	0.000000196
0.000139077	0.0000845	0.0000059
0.000000583	0.000000874	0.000000736
0.000000196	0.000000726	0.000000517
0.000117683	0.0000415	0.00000191
0.0000183	0.0000074	0.000000298
0.0000888	0.0000337	0.00000276
0.000000178	0.000000587	0.000000473
0.000120115	0.0000532	0.00000197
0.000036	0.0000137	0.00000062
0.000036	0.0000137	0.00000062
0.000036	0.0000137	0.00000062
0.000036	0.0000137	0.00000062
0.0000496	0.0000158	0.000000262
0.000046	0.0000134	0.000000263
0.000000474	0.000000835	0.000000672
0.0000439	0.0000136	0.000000196
0.000000362	0.000000682	0.000000395
0.00000656	0.000000779	0.000000138
0.000000423	0.000000728	0.000000642
0.000000573	0.00000813	0.000000277
0.00006	0.0000214	0.00000106
0.0000924	0.0000267	0.00000188
0.000133625	0.0000601	0.00000284
0.000000416	0.000000696	0.000000612
0.000000511	0.000000826	0.000000692
0.000000452	0.000000888	0.000000513
0.000000418	0.000000885	0.000000505
0.000000179	0.000000481	0.000000243
0.000000365	0.000000513	0.000000527
0.0000788	0.0000791	0.00000204
0.000000457	0.00000071	0.000000694
0.000000215	0.000000609	0.000000456
0.114176	0.0156625	0.000364703
0.000000594	0.00000111	0.000000771
0.00000023	0.000000668	0.000000437

	0.000000193	0.000000517	0.00000049
	0.000174638	0.00000144	0.000000983
	0.0000872	0.0000266	0.00000237
	0.000000246	0.000000555	0.000000573
	0.000000325	0.000000728	0.000000497
	0.000000203	0.000000443	0.000000533
	0.000000631	0.00000104	0.000000725
	0.000000236	0.000000504	0.00000052
	0.000131655	0.000164148	0.00000546
	0.000131655	0.000164148	0.00000546
	0.000000346	0.000000749	0.000000523
	0.000000443	0.000000719	0.000000735
	0.000000505	0.00000074	0.000000821
	0.000000248	0.000000731	0.000000522
	0.000000114	0.000157254	0.000000104
	0.000000269	0.000000789	0.000000559
	0.000000617	0.000000833	0.000000951
	0	0	0.000000107
	0.000000257	0.000000692	0.000000529
	0.000000504	0.00000079	0.000000758
	0.000000373	0.000000702	0.000000677
	0.000000348	0.00000069	0.000000703
	0.000000231	0.000000746	0.00000051
	0.000000242	0.000000593	0.000000516
	0.000000445	0.000000619	0.000000672
	0.000000243	0.000000597	0.000000535
	0.000000385	0.000000797	0.000000738
	0.000000494	0.000000688	0.000000766
	0.000000175	0.000000588	0.000000474
	0.0000515	0.00000873	9.48E-08
	0.000000429	0.000000691	0.000000758
	0.000000365	0.000000492	0.000000806
	0.000000365	0.000000492	0.000000806
	0.000000422	0.000000787	0.000000867
	0.000115087	0.0000513	0.00000464
	5.06E-08	0.000000366	0.000000382
	5.06E-08	0.000000366	0.000000382
	0.000000241	0.000000498	0.000000541
	0.000000241	0.000000498	0.000000541
	0.000149166	0.0000178	0.000000258
	0.000000389	0.000000875	0.000000914
	0.000000389	0.000000875	0.000000914
	0.000000292	0.000000589	0.000000822
	0.000123443	0.00000874	0.00000202
	0.000000243	0.000000434	0.000000631
	0	0	0.00000276
	0.000000249	0.000225518	0.000000258

0.000000182	0.000204207	0.000000236
0.000000192	0.000157254	0.000000189
0.00000175	0.00000387	0.000000831
0.00000309	9.15E-08	0.000000394
0.000000103	0.000136791	0.000000174
0	0	0.000000145
0.000186338	0.000137126	0.0000038
0.000000137	0.000218464	0.000000296
0.000120959	0.000000406	0.000000358
0.000154634	0.0000311	0.00000403
0.000000483	0.000275932	0.000000967
0.0000685	0.00000101	0.000000819
0.000000902	0.000334466	0.00000119
0.0000178	0.0000014	0
0.0000343	0.213593	0.000303919
0.000000772	0.000300735	0.00000107
0.0000205	0.00000707	0
0.0000192	0.00000158	0
0.0000835	0.0000472	0.0000043
0.000000226	0.000207674	0.000000307
0.000000568	0.000278739	0.00000104
0.000000563	0.000274318	0.00000107
0.000000194	0.000247905	0.000000384
0	0.0000382	9.68E-08
0.000000544	0.000319695	0.00000125
0.000000644	0.000333509	0.00000132
0.000022	0.00000521	0
0.000000876	0.000433813	0.00000169
0.000000222	0.000213362	0.00000034
0.000000222	0.000213362	0.00000034
0.000000522	0.000266566	0.00000108
0.00014216	0.00000882	0.00000302
0.000000181	0.000245059	0.000000401
0.000000145	0.000217525	0.00000036
0.0000287	0	5.14E-08
0.000000216	0.000230261	0.000000384
0.000212572	0.0000369	0.00001
0.000196531	0.000162029	0.00000534
0.0000567	0.0000186	0.000000284
0.0000391	0.000131101	0.00000781
0.0000231	0.00000257	0
0.00448303	0.21046	0.000999054
0.0000145	0.00000142	0
0	0	0
0.0000344	0.00000884	0
0.0000828	0.0000406	0.00000606
0.0000195	0	4.54E-08

0.00000694	0.000000569	0
0.0000779	0.0000135	0.000000891
0.000123458	0.0000238	0.00000405
0.0000542	0.0000824	0.0000029
0.0000324	0.0000076	0
0.0000973	0.000000126	0.00000023
0.000172091	0.000268283	0.0000148
0.000172091	0.000268283	0.0000148
0.000172091	0.000268283	0.0000148
0.0178488	0	0.0000454
0.000165906	0.000021	0.000000478
0.00010349	0.0000145	0.00000319
0.0000721	0.0000145	0.00000282
0.00000123	3.57E-08	0.000000226
0.0000473	0.0000075	0.000000154
0.00000303	0.0000173	5.36E-08
0.0001356	0.0000214	0.0000047
0.0000866	0.000202728	0.0000101
0.00000106	3.06E-08	0.000000221
0.000102277	0.0000155	0.00000342
0.000205948	0.0000266	0.00000075
0.000146399	0.0000243	0.00000534
0.000099	0.00000011	0.000000101
0.0000194	0	6.41E-08
0.0000204	0	6.73E-08
0.0000515	0.00000873	9.48E-08
0.0000195	0	6.79E-08
0.00000266	0.0000164	6.45E-08
0.0000243	0.00000597	0
0.00000272	0.0000182	7.15E-08
0	0.000045	0.000000248
0.0927408	0.00101531	0.000554266
0.00000453	0.00000552	0.000000445
0.00000275	0.0000156	6.74E-08
0.00000275	0.0000156	6.74E-08
0	0.0000395	3.46E-08
0.00000287	0.0000182	7.88E-08
0.0000196	0	7.65E-08
0.000235581	0.0000451	0.0000116
0.00000319	0.0000172	7.77E-08
0.00000367	0.0000171	8.13E-08
0.00000286	0.0000173	7.95E-08
0	0.0000432	0.000000264
0.000161084	0.0000204	0.000000465
0.000112966	0.000000248	0.000000218
0.000112966	0.000000248	0.000000218
0.00000285	0.0000158	7.57E-08

0.000148796	0.0000228	0.00000558
0.00000286	0.0000164	7.91E-08
0.000198895	0.000000402	0.000000442
0.000164133	0.0000208	0.000000531
0.000126728	0.000000334	0.000000259
0.000167362	0.0000213	0.000000556
0.0000037	0.0000187	9.61E-08
0.0000841	0.0000919	0.000011
0.000210719	0.000000442	0.000000661
0.00000286	0.0000163	0.000000086
0.0000188	0.00000637	0.000000136
0.00000298	0.0000166	9.37E-08
0.0000395	0.00000356	0.000000274
0.0000952	8.61E-08	0.000000125
0.00000241	0.0000181	0.0000001
0.0000216	0.00000913	0.00000123
0.000123749	0.000109653	0.0000101
0.000123718	0.00010962	0.0000101
0.0000991	0.0000143	0.00000478
0.00000352	0.000019	0.00000011
0.00000352	0.000019	0.00000011
0.000153821	0.000000387	0.000000301
0.00000352	0.0000179	0.000000108
0.00191939	0.0107318	0.000064
0.00000293	0.0000166	0.000000101
0.0000835	0.000024	0.00000212
0.00000617	0.00000635	0.000000624
0.00000291	0.0000194	0.000000121
0.000126175	0.000000131	0.000000291
0.000118752	0.000000116	0.00000026
0.000107422	0.000000157	0.000000298
0.000158567	0.0000278	0.0000063
0.0000196	0	0.000000115
0.000112401	0.000000162	0.000000239
0.00015809	0.000000161	0.000000339
0.0000998	0.000000127	0.000000214
0.00000289	0.0000194	0.000000128
0.0000982	0.000000149	0.000000219
0.0000026	0.00000532	4.27E-08
0.00000321	0.0000196	0.000000134
0.000143237	0.000100633	0.0000154
0.000116382	0.000000119	0.000000246
0	0.0000507	0.000000177
0.000113693	0.000000123	0.000000264
0.00000286	0.0000168	0.000000127
0	0.0000497	0.000000181
0.000139112	0.000000295	0.000000263

0.0000029	0.0000162	0.000000126
0.000160237	0.000000382	0.000000426
0.000160237	0.000000382	0.000000426
0.0000987	0.0000178	0.000006
0	0.0000423	0.000000148
0.000000699	0.00000126	0.00000029
0.00000345	0.0000179	0.000000166
0.0000668	0.0000145	0.00000769
0.0000043	0.0000249	0.00000023
0	0.0000396	0.000000107
0.00000279	0.00000705	0.00000165
0.0000031	0.00000537	0.000000128
0.000140433	0.000000296	0.000000355
0.000156855	0.000281405	0.0000171
0.00000238	0.0000162	0.000000159
0.000187423	0.000395108	0.0000166
0.0000943	0.0000284	0.00000383
0.000007	0.00000436	0.000000239
0.000136482	0.000000307	0.00000036
0.0000126	0.0000047	0.000000178
0.0000655	0.00000208	0.000000208
0.000101703	0.000000146	0.000000365
0.0000628	0.0000125	0.00000867
0.000134592	0.000290974	0.0000481
0.000146178	0.000333535	0.0000163
0.000297243	0.000298122	0.0000421
0.00000567	0.00000494	0
0.000122448	0.00012597	0.0000166
0.000726175	0.000970219	0.000143665
0.0000495	0	0.00000489
0.000011	0.00000665	0.000000139
0	0.0000488	0.000000246
0	0.0000488	0.000000246
0.0000018	0.00000438	4.54E-08
0.0000588	0.0000103	0.00000324
0.00000293	0	0
0	0.0000508	0.000000192
0	0.000043	0.000000167
0	0.0000422	0.000000164
0	0.0000394	0.000000208
0.00000689	0.00000622	0.000000118
0.000109108	0.0000461	0.0000239
0.00000155	0.00000226	0.000000359
0.0000102	0.00000646	0.000000176
0.000392195	0.000420252	0.0000723
0	0.0000427	0.000000175
0.0000109	0.00000402	0.000000362

0.0000375	0.0000119	0
0	0	0.000000099
0.000119942	0.000134914	0.0000178
0	0.0445989	0.000183471
0.000144228	0.000294807	0.0000204
0.00000502	0.00000612	0.000000139
0.00000271	0.000000117	0.000000192
0	0.0000398	0.000000119
0.00000482	0.00000607	0.000000118
0	0.0000392	0.000000173
0	0.0000392	0.000000173
0.0000035	0.00000663	0.000000161
0.0000035	0.00000663	0.000000161
0.00016764	0.0000948	0.0000369
0	0.0000442	0.000000223
0.0000111	0.0000077	0.000000179
0	0.0000454	0.000000239
0.00000617	0.00000633	0.000000158
0.00000139	0.00000252	0.00000195
0.00000195	0.00000129	0.00000106
0.000156274	0.0000264	0.00000619
0.00000823	0.00000595	0.000000133
0.00000115	0	0.000000863
0.00010294	0.000064	0.0000145
0.0000981	0.0000356	0.0000142
0.0000467	0.0000137	0
0.0000456	0.000145541	0.0000516
0.000000918	0.000000744	0.000000294
0.000000243	0.00000654	0.000000834
6.15E-08	0	0
0	0	4.49E-08
0	0	4.49E-08
0.00000728	0.00000717	0.000000168
0.0000649	0.0000167	0.00000693
0.000211388	0.000313441	0.000092
0	0.0000428	0.000000226
0.000279427	0.000163227	0.0000829
0.00000573	0.00000213	0.000000408
0.000274739	0.000358901	0.000116203
0.000216513	0.000311637	0.0000436
0.00000452	0.00000602	0.000000162
0.000298475	0.000410685	0.000130842
0.000000808	0.000000153	0
0.000353399	0.000457256	0.000149811
0.000315344	0.000218085	0.0000918
0.000317431	0.000410947	0.000148545
0.000244694	0.000281545	0.000136618

0.000244694	0.000281545	0.000136618
0.000000441	0.00000418	0.000000323
0.00000044	0.00000418	0.000000323
0.000152147	0.000106162	0.0000244
0.0000014	5.89E-08	0.00000016
0.000000146	0	0
0.000336058	0.000696829	0.00019923
0.000102835	0.0000399	0.0000204
0	0.0000432	0.000000178
0.0000333	0.0000415	0.0000202
0.0000723	0.0000193	0.0000094
0.00000213	0.0000186	0.00000125
0.000478875	0.000404664	0.000130873
0.000142251	0.0000755	0.0000134
0.0000164	0.00000476	0.000000899
0.000000701	0.000000202	0
0.0000354	0.0000131	0.00000838
0.000226026	0.0000239	0.0000502
0.000311376	0.000452514	0.000173384
0.000000701	0.000000202	0
0.000000701	0.000000202	0
0.000398256	0.000155443	0.000167173
0.000397606	0.000509188	0.000261761
0.000100825	0.00033762	0.0000929
0.000100427	0.0000737	0.0000565
0.000000576	0.00000776	0
0.000314737	0.000454681	0.000116832
0.000000356	0.0000282	0.00000795
0.000413995	0.000471122	0.000203574
0.000238901	0.000245184	0.000126723
0.00000318	0.0000141	0.00000178
0.000478803	0.000610048	0.000310897
0.00000193	0.00000148	0.0000009
0	0	1.83E-08
0.00000311	0.000000832	0.000000962
0	0.00000022	0.000000225
0	0.00000577	0.000000539
0	0.000000236	0.000000241
0.00000032	0.000000416	0
0.00000032	0.000000416	0
0	0.000000283	0.000000286
0.000407557	0.000381321	0.00022324
0	0	1.81E-08
0	0	0
0	0.0000412	0.000000358
0	0	0.0000234
0	0.00000033	0.000000332

0.000345614	0.00047603	0.000165978
0.0000716	0.0000247	0.0000447
0.000500598	0.000909389	0.000284777
0	0.000000376	0.000000377
0	0	0
0	0	0.000000021
0	0	0
0.000000505	0.000000153	0
0	0	0
0.000179746	0.000599086	0.000184619
0.0000927	0.000342021	0.000103609
0	0	5.12E-08
0	0.000000283	0.000000292
0.0000065	0	0.000000564
0.0000775	0.0000269	0.0000256
0.000280516	0.000467474	0.00013995
0.000342718	0.000478937	0.000186101
0	0	7.46E-08
0.0000608	0.000011	0.0000153
0.000366521	0.000534997	0.000198692
0.000000358	0.000000399	9.42E-08
0.000000193	0	1.79E-08
0.000106614	0.0000396	0.0000363
0	0	0
0.000478875	0.000404664	0.000229938
0.000446947	0.0003062	0.000205986
0.000151117	0.000268773	0.000114648
0.000000184	0.0000107	0.00000109
0.000195549	0.000236347	0.000202449
0	0	0
0.000000845	0	0
0.000000071	0.00000757	0.000000694
0	0	0
0	0	0
0	0	0.000000437
0.00000015	0	0
0.0000721	0.0000591	0.0000493
0	0.0000141	0.00000672
0	0.00000053	0
0	0	0
0.000179941	0.000352283	0.0000618
0	0	0
0.000698247	0.00114401	0.000545969
0.0000671	0.0000141	0.000014
0	0	0
0.00000515	0.0000204	0.0000133
0.00000515	0.0000204	0.0000133

0	0.014186	0.00711322
0	0.0000134	0.00000672
0.0000031	0.000000657	0.000000688
0.000367702	0.000665422	0.000265528
0.000000566	0.000000376	0.000000458
0.000196049	0.000243674	0.000269095
0.0000188	0.00000539	0.00000358
0.000107118	0.0000623	0.0000407
0.0000848	0.0000343	0.0000481
0.0000626	0.0000512	0.0000274
0.000335087	0.000304923	0.000131859
0.000123417	0.0000648	0.0000757
0.000000186	0	3.18E-08
0	9.06E-08	0.000000146
0.0000273	0.0000776	0.000068
0	0.000000328	5.78E-08
0	0.0000148	0.00000791
0	0.0000148	0.00000791
0.000037	0.00000545	0.0000243
0	0.000000158	0
0.000216609	0.000259259	0.0000954
0	0	0.000000345
0.000000152	0	2.89E-08
0.000000388	0	0
0.000391419	0.00045162	0.000342587
0.000000376	0	0
0.0000016	0.0000027	0.00000087
0.000382858	0.000678814	0.000409127
0	0	0
0.000875405	0.00133848	0.00084449
0.0000104	0.00000479	0.000003
0.77497	0.716192	0.522069
0.000000345	0	6.96E-08
0	0	0
0.000000715	0.00000625	0.00000179
0.00043261	0.000959846	0.000534659
0.000154688	0.000269592	0.000131662
0	0.000000395	8.25E-08
0.0000221	0.0000113	0.00000875
0.000000106	0	0
0.000000119	0.00000103	0.000000243
0.000000119	0.00000103	0.000000243
0.0000977	0.0000451	0.0000668
0.00000191	0.00000418	0.00000131
0.000000539	0	0
0.000000476	0.0000257	0.0000107
0.000114941	0.0000165	0.0000231

0	0	0
0.000582017	0.000821662	0.000522733
0	0.0000107	0.00000724
0.000000157	0	3.51E-08
0.00000013	0	2.92E-08
0.000000175	0	3.97E-08
0	0	0
0	0	0
0	0	0
0	0.0000119	0.00000653
0.000107752	0.0000527	0.0000758
0.000000197	0	4.75E-08
0.00000234	0.0000323	0.00000971
0.00000112	0.0000228	0.00000799
0.000401007	0.000155518	0.000191507
0.00000394	0.00000572	0.00000427
0.000635168	0.000786584	0.000627122
0.000000137	0	3.45E-08
0.00000307	0.0000379	0.0000119
0	0	0
0	0	0
0.000000365	0.000000127	0
0.000000365	0.000000127	0
0.000000365	0.000000127	0
0.000375566	0.000703478	0.00046755
0.000570601	0.000900181	0.000592459
0.000000145	0	3.95E-08
0	0	0
0.00000475	0	0.0000065
0	0	0
0	0.0000128	0.00000747
0	0	0
0	0.0000106	0.00000581
0.000337919	0.000404016	0.000341301
0	0.0000101	0.00000626
0.000000126	0	3.55E-08
0	0.0000108	0.00000659
0	0.0000115	0.00000708
0	0.0000115	0.00000708
0	0.000000503	0.000000143
0.000589577	0.000909816	0.000632059
0.000570625	0.000891849	0.000608022
0	0	0
0	0	0
0	0.000000314	9.17E-08
0	0.000000314	9.17E-08
0.000185175	0	0.0000543

0	0	0
0	0	0
0	0.0000101	0.00000653
0	0	0
0	0	0
0.000395735	0.000619235	0.000237864
0.000000193	0	0.000000058
0.0000595	0.0000776	0.00010443
0	0	0
0.00039401	0.000599539	0.000420806
0	0	0
0.00000383	0.0000064	0.00000497
0	0	0
0	0	0.00000192
0	0.00000938	0.00000631
0	0	0
0	0	0
0	0	0
0.000000109	0	3.42E-08
0.000000156	0	0.000000049
0	0	0
0	0	0
0	0.0000129	0.0000087
0	0	0
0	0.00000946	0.00000629
0	0	0
0	0	0
0.00000146	0.00000894	0.00000347
0	0	0
0	0	0
0	0.000000417	0.000000136
0	0.00000037	0.000000121
0.000301917	0.000335384	0.000359728
0.000630167	0.000742389	0.000591877
0.000630167	0.000742389	0.000591877
0	0.00000806	0.00000564
0.0000197	0.0000101	0.00000858
0	0	0
0	0.00000929	0.00000633
0	0.00000929	0.00000634
0	0	0.00385725
0	0	0.0146864
0	0	0
0	0	0
0	0	0.00801873
0	0	0.0044382
0	0	0.000802798

0	0	0.00194242
0	0	0
0	0	0.0000685
0	0	0
0	0	0.00541951
0	0	0
0	0	0
0	0	0
0	0	0
0	0	0
0	0	0
0	0	0
0	0	0
0	0	0
0	0	0.00320651
0	0	0
0	0	0
0	0	0
0	0	0
0	0	0
0	0	0
0	0	0
0	0	0
0	0	0
0	0	0
0	0	0
0	0	0
0	0	0
0	0	0.00358436
0	0	0.00302967
0	0	0.00721597
0	0	0
0	0	0.00244165
0	0	0
0	0	0
0	0	0
0	0	0
0.00428342	0.000521065	0
0	0	0
0	0	0.000774627
0	0	0.0025781
0	0	0
0	0	0
0	0	0.0198963
0	0	0.000256669
0	0	0
0	0	0

0	0	0
0	0	0
0	0	0
0	0	0.00145504
0	0	0
0	0	0
0	0	0
0	0	0.000996222
0	0	0.00335598
0	0.00174243	0
0	0	0
0	0	0
0	0	0
0	0	0
0	0	0
0	0	0
0	0	0.00215791
0	0	0
0	0	0.0072218
0	0	0
0	0	0.00509231
0	0	0.00115843
0	0	0
0	0	0.00111799
0	0	0
0	0	0
0.0039958	0.00498704	0
0	0	0
0	0	0
0	0	0.00145309
0	0	0.000283903
0	0	0.00062609
0	0	0
0	0	0
0	0	0
0	0	0.017621
0	0	0.00234
0	0	0.00487814
0	0	0
0	0	0
0	0	0.0000464
0	0	0
0	0	0.00231185
0	0	0.00122133
0	0.00110703	0
0	0	0.0022888
0	0	0

0	0	0
0	0	0.00136372
0	0	0.00123356
0	0	0.0161232
0	0	0.00175794
0	0	0
0.00580072	0.000220811	0
0.00577471	0	0
0.00178755	0.000372375	0
0	0	0
0	0	0
0	0	0
0	0	0.000990634
0	0	0
0	0	0.00153154
0	0	0.00203633
0	0	0
0	0	0
0	0	0
0	0	0
0	0	0
0	0	0
0	0	0.000361498
0.000270089	0	0
0.000721455	0.00229613	0
0	0	0.000380371
0	0	0
0	0	0
0	0	0
0	0	0
0	0	0
0	0	0
0	0	0.00128167
0	0	0
0	0	0
0	0	0
0	0	0
0	0	0.0000498
0	0	0
0	0	0
0	0	0.0000817
0	0	0.00989144
0	0	0
0	0	0
0	0	0.0000201
0	0	0
0	0	0

0	0	0
0	0	0
0	0	0
0	0	0
0	0	0
0	0	0
0	0	0.000000548
0	0	0
0	0	0
0	0	0
0	0	0
0	0	0
0	0	0.000002
0	0	0.00000274
0	0	0.00000605
0	0	0
0	0	0
0	0.000000119	0
3.91E-08	0	0
0	0	9.57E-08
0	0	0.00000114
0	0	0
0	0	0
0	0	0
0	0	0
0	0	0
0	0	0.00000115
0.000000434	0.000000824	0
0	0.000000197	0
0.000000147	0	0
0	0	0.000000887
0	0	0.00000328
0	0	0.000000501
0	0	0
0	0	0
0	0	0
0	0	0
0	0	0
0	0	0
0	0	0
0	0.00000015	0
0.00000017	0	0
0	0	0.00000108
0.00000047	0	0
0.000000988	0.00000357	0
0	0	0
0	0	0

0	0	0
0	0	0
0	0	0
0	0	0
0	0	0
0	0	0
0	0	0
0	0	0.000000526
0	0	0
0	0	0
0	0	0
0	0	0
0	0	0
0	0	0
0	0	0
0	0	0.00000266
0	0	0.00000261
0	0	0.00000584
0	0	0
0	0	0
0	0	0.000000293
0	0	0
0	0	0.000000652
0	0	0
0	0	0
0.000000127	0	0
0	0	0
0	0	0.000000373
0	0	0
0	0	0
0	0	0
0	0	0.000000294
0	0	0.000000695
0	0	0
0.000000139	0	0
0	0	0
0	0	0.000000349
0	0	0
0	0	0.0000241
0	0	0
0	0	0
0	0	0
0	0	0.000000425
0	0	0
0	0	0
0	0	0
0	0	0.0000196

0	0	0.000000343
0	0	0
0	0	0
0	0	0
0	0	0
0	0	0
0	0	0
0	0	0
0	0	0
0	0	0
0	0	0
0	0	0.00000189
0	0	0
0	0	0
0	0	0
0	0	0
0	0	0
0	0.000000105	0
0	0	0.000000703
0	0	0
0	0	0.000000728
0	0	0.0000247
0	0	0
0	0	0.000000291
0	0	0.000000274
0	0	0
0	0	0
0	0	0
0	0	0
0	0	0
0	0	0
0	0	0
0	0	0.000000431
0	0	0
0	0	0
0	0	0
0	0	0
0	0	0
0	0	0.00000258
0	0	0
0	0	0
0	0	0
0.00000153	0.000000334	0
0	0	0.0000012
0	0	0
0	0	0
0	0	0

0	0	0
0	8.66E-08	0
0	0	0.0000226
0	0	9.55E-08
0	0	0
0	0	0.0000201
0	0	0
0	0	0.00000142
0	0	0.000000572
0	0	0
0	0	0.000008
0	0	0
0	0	0
0	0	0
0	0	0.000000924
0	0	0
0	0	0
0	0	0
0	0	0
0	0	3.43E-08
0	0	0.0000049
0	0	0
0	0	0.00000209
0	0	0
0	0	6.12E-08
0	0	0
0	0	0.000000105
0	0	0
0	0	0.00000005
0	0.000000178	0
0	0	0.0000287
0.0000002	0.000000114	0
0	0	0.00000094
0	0	0.000000226
0	0	0
0	0	0.00000111
0	0.000000309	0
0	0	0.00000776
0	0	0.000023
0	0	0
0	0	0
0	0	0.00000601
0	0	5.96E-08
0	0	0
0	0	0.000000478
0	0	0
0	0	0

0	0	0
0	0	0
0	0	0
0	0	0
0	0	0
0	0	0.000000525
0	0	0
0	0	0
0	0	0
0	0	0
0	0	0
0	0	0
0	0	0
0	0	0
0	0	0
0	0	0.000000477
0	0	0
0	0	0
0	0	0
0	0	0.00000282
0	0	0.00000539
0	0	0
0	0	0.00000183
0	0	0
0	0	0
0	0	0
0.00000684	0.000000667	0
0	0	0
0	0	6.74E-08
0	0	0
0	0	0
0	0	0.0000135
0	0	0
0	0	0
0	0	0
0	0	0
0	0	0
0	0	0
0	0	4.41E-08
0	0	0.000000834
0	0.00000118	0
0	0	0
0	0	0
0	0	0
0	0	0
0	0	0
0	0	0

0	0	0
0	0	0.00000169
0	0	0.000000794
0	0	0
0	0	0.00000104
0.00000231	0.00000274	0
0	0	0
0	0	0.00000146
0	0	0
0	0	5.54E-08
0	0	0
0	0	0
0	0	0.0000172
0	0	0
0	0	0
0	0	0
0	0	4.63E-08
0	0	0
0	0	0.00000367
0	0	0.000000067
0	0.000000105	0
0	0	0.00000106
0	0	0
0	0	0
0	0	0.000000671
0	0	6.74E-08
0	0	0.0000167
0	0	6.87E-08
0	0	0
0.00000018	4.66E-08	0
0.00000285	0	0
0	0	0
0	0	0.000000817
0	0	0
0	0	0.000000258
0	0	0.000001
0	0	0
0	0	0
0	0	0
0	0	0
0	0	0
0	0	0.000000307
5.58E-08	0	0
0.00000115	0.00000289	0
0	0	0.000000156
0	0	0
0	0	0

0	0	0
0	0	0
0	0	0
0	0	0.000000965
0	0	0
0	0	0
0	0	0
0	0	0
0	0	0.00000691
0	0	0
0	0	0
0	0	0.0000232
0	0	4.98E-08
0	0	0
0	0	0.000000333
0	0	0.000000622
0	0	0
0	0	0
0	0	0
0	0	0
0	0	0
0.00000631	0.000000667	0
0	0	0
0	0	0
0	0	0
0	0	0
0	0	0.000000593
0	0	0
0	0	0.000000984
0	0	0
0	0	0.00000145
0	0	0
0	0	0.0000178
0	0	0.000000719
0	0	0
0.000000246	0	0
0.00000104	0.00000263	0
0	0	0
0	0	0
0	0	0
0	0	0
0	0	0
0	0	0
0	0	0.000000364
0	0	0
0	0	0
0	0	0

0	0	0
0	0	0
0	0	0.0000258
0	0	0
0	0	0
0	0	0
0	0	0
0	0	0.0000205
0	0	0
0	0	0
0	0	0
0	0	0
0	0	0
0	0	0
0	0	0
0	0	0
0	0	0
0	0	0
0	0	0
0	0.000000115	0
0	0	0.000000704
0	0	0
0	0	0.000000731
0	0	0.000025
0	0	0
0	0	0
0	0	0
0	0	0
0	0	0
0	0	0
0	0	0
0	0	0
0	0.000000132	0
0	0	0
0	0	0.00000339
0	0	0
0	0	0
0	0	0
0	0	0.000000678
0	0	0
0	0	0
0	0	0
0	0	0
0	0	0
0.00000596	0.000000612	0
0	0	0
0	0	0.0000182

0	0	0
0	0	0
0	0	0
0	0	0
0	0	0
0	0	0
0	0	0
0	0	0
0	0	0.00000157
0	0	0
0	0	0
0	0	6.25E-08
0	0	0.0000303
0	0	0.00000229
0	0	0
0	0	0
0	0	0
0	0	0
0	0	0
0	0	0
0	0	0
0	0	0
0	0	0
0	0	0
0	0	0
0	0	0
0	0	0
0	0	0
0	0	0
0	0.000000288	0
0	0	0
0	0	0
0	0	0
0	0.000000224	0
0	0	3.82E-08
0	0	0
9.34E-08	0	0
0	0	0
0	0	0
0	0.000000127	0
0	0	0
0	0	0
0	0	0
0	0	0.00000257
0	0	0
0	0	0
0	0	0

0	0	0
0	0	0
0	0	0
0	0	0
0	0	0
0	0	0
0	0	0
0	0	0
0	0.00000013	0
0.000000516	0	0
0	0	0
0	0	0.0000002
0	0	0
0	0	0.000000726
0	0	0
0	2.94E-08	0
0	0	0
0	0	0.000000317
0	0	0
0	0	0
0	0	0
0.000000195	0	0
0	0	0
0	0	0
0	0	0.0000193
0	0	0
0	0	0
0	0	0
0	0	0
0	0	0.0000102
0	0	0
0	0	0
0	0	0
0	0	2.73E-08
0	0.000000146	0
0	0	0.0000386
0.000000146	6.46E-08	0
0	0	0
0.000000331	0	0
0	0	0
0	0	0
0	0	0
0.00000016	0	0
0	0	0
0	0	0
0	0.000000159	0
0	0	0

0	0	0
0	0	0
0	0.000000097	0
0	0	7.37E-08
0	0	0
0	0.000000174	0
0	0	0
0	0	0
0	0	0
0	0	0
0.000000297	0.000000231	0
0	0	0
0	0	0
0	0	0.00000296
0	0	0
0	0	0
0	0	0
0	0	3.94E-08
0	0.000000104	0
0	0	0
0	0	1.96E-08
0	0	0
0	0	0
0	0	0
0	0	0.00000273
0	0	0
0.00000553	0.000000453	0
0	0	0
0	0	0
0	0	0
0	0	0
0	0	0
0	0	0
0	0.00000012	0
0	0	0.0000186
0	0	0
0.000000201	0	0
0.00000241	0	0
7.58E-08	0	0
0	0	0.000000222
0	0	0
0	0	0
0	0	0
0	0	0
0	0	0
0	0	0
0	0	0

0	0	0
0	0.000000179	0
0	0	0.0000284
0	0	0
0	0	6.39E-08
0	0	0.0000235
0	0	9.42E-08
0	0	0
0	0	0.0000234
0	0	0
0	0	0.0000015
0	0	0.000000802
0	0	0
0	0	0.0000081
0	0	0
0	0	0
0	0	0
0	0	0.000000925
0	0	0
0	0	0
0	0	0
0	0	0
0	0	5.65E-08
0	0	0.00000499
0.00000085	0.00000271	0
0.00000224	0.000000153	0
0	0	0
1.87E-08	0.000000839	0
0.000000483	0.000000105	0
2.96E-08	0.000000117	0
0	0	0
0	0	0
0	0	0
0	6.31E-08	0
0	0	8.03E-08
0	0	0
0	0	0
0	0	0
0	0	0
0	0.000000186	0
0	0	0.000025
0.000000101	0	0
0	0	0
0	0	0
0	0	0
0	0	0
0	0.000000206	0

0	0	0.0000249
0.000000101	0	0
0	0	0
0	0	0
0	0	0
0	0.000000284	0
3.58E-08	0	0
0	0.000000103	0
0	0	0.000000218
0	0	0
0	0.000000148	0
0	0	0
0	0	0.000000242
9.89E-08	0	0
0	0	0.00000671
0	0	0.0000225
0	0	0
0	0	0
0	0	0.00000657
0	0	0
0	0	0
0	0	0.000000341
0	0	0
0	0	0.000000377
0	0	0
0	0	0
0	0	0
0	0	0
0	0	0
0	0	0
0.00000677	0.000000627	0
0	0	0
0	0	0
0	0	0.000017
0	0	0.0000019
0	0	0.000000311
0	0	0
0	0	0
0	0	0
0	0	0
0	0.00000128	0
0	0	0
0	0	0
0	0	0
0	0	0
0	0	0
0	0	0.00000227

0	0	0
0	0	0
0	0	0.00000139
0	0	0.00000175
0	0	3.76E-08
0	0	7.93E-08
0	0	0
0	0	0
0	0	0.000000117
0	0.000000133	0
0	0	0.000000607
0	0	0
0	0	0.000000543
0	0	0
0	0	0
0	0	0
0	0	0.000000991
0	0	0
0	0	0.000000267
0	0	0.000000245
0	0	0
0	0	0
0	0	0
0	0	0.00000142
0	0	0
0	0	0.00000318
0	0	0
0	0	0.00000273
0	0	0
0	0	0
0	0	0.0000214
0	0	0
0	0	0
0	0	7.25E-08
0	0	0
0	0	0.00000052
0	0	0
0	0	0.00000213
0	0	0
0	0	0
0	0	0.000000156
0	0	6.92E-08
0	0.000000208	0
0	0	0
0	0	0.0000363
0.00000344	0	0
5.41E-08	4.28E-08	0

0.00000013	0	0
0	0	0.00000108
0	0	0
0	0	0.000000286
0	0	0
0	0	0
0	0	0
0	0	0
6.06E-08	0	0
0	0	0
0	0	0
0	0	0
0	0	0
0.000000152	0	0
0.000000165	0.0000011	0
0	0	0
0	0	0
0	0.000000105	0
0	0	0
0	0	0
0	0	0
0	0	0
0	0	0
0	0	0
0	0	0
0	0	0
0	0	0
0	0	0
0	0	0.0000469
0.000000308	0.000000226	0
0.00000129	0.000000337	0
0	0	0.000009
0	0	0.0000228
0	0	0
0	0	0
0	0	0.00000617
0	0	0
0	0	0.000000102
0	0	0
0	0	0
0	0	0
0	0	0
0	0	0
0	0	0
0	0	0
0	0	0
0	0	0

0	0	0
0	0	0.000000366
0	0	0
0	0	0
0	0	0
0	0	0
0	0	0
0	0	0
0	0	0
0	0	0
0	0	0
0	0	0
0	0	0
0	0	0
0	0	0.00000236
0	0	0.00000287
0	0	0.00000605
0	0	0
0	0	0.00000221
0	0	0.000000772
0	0	3.61E-08
0	0	0
0	0	0
0	0	9.98E-08
0	0	0
0	0	0
0	0	0
0	0	0
0	0	0
0	0	0
0	0	0.00000849
0	0	0.00000273
0	0	0
0	0	0.00000169
0	0	0
0.00000206	0.00000223	0
0	0	0
0	0	0.0000228
0	0	0.000000212
0	0	0
0	0.000000071	0
0	0	0.00000327
0	0	0
0	0	0.00000138
0	0	0.0000281
0	0	0
0	0	0.00000111

0	0	0
0	0	0.000000251
0	0	0.00000105
0	0	0
0	0	0
0	0	0
0	0	0
0	0	0
0	0	0.000000254
0	0	0
0	0	0
0	0	0
0	0	0
0	0	0.00000018
0	0	0.0000234
0	0	5.81E-08
0	0	0
0	0	0.000000467
0	0	0
0	0	0
0	0	0
0	0	0
0	0	0
0	0	0
0	0	0
0	0	0.000000903
0	0	0
0	0	0
0	0	0.0000218
0	0	0.000000878
0	0	0
0	0	0
0	0	0
0	0.000000167	0
0	0	0.000000882
0	0	0
0	0	0.00000803
0	0	0
0	0	0
0	0	0.000011
0	0	0.00000618
0	0	3.73E-08
0	0	0
0	0	0.000000397
0	0	0
0	0	0
0	0	0

0	0	0.000000572
0	0	0
0	0	0
0	0	0
0	0	0
0	0	0
0	0	0
0	0	0
0	0	0
0	0	0
0	0	0
0	0	0
0	0	0.00000595
0	0	0
0	0	0.00000239
0	0	0
0	0	0
0.0000067	0.000000509	0
0	0	0.000000641
0	0	0
0	0	0
0	0	0
0	0	0.00000461
0	0	0.000000116
0	0	0
0	0	0
0	0	0.00000122
0	0.00000149	0
0	0	0
0	0	0
0	0	0
0	0	0
0	0	0
0	0	0
0	0	0.00000226
0	0	0.00000072
0	0	0
0	0	0.000000994
0	0	0
0	0	0
0	0	0.00000147
0	0	0
0	0	5.59E-08
0	0	0
0	0	0.000000218
0	0	0
0	0	0

0	0	0
0	0	0.000000675
0	0	0
0	0	0
0	0	0.000000707
0	0	4.95E-08
0	0	0.0000208
0	0	0
0	0	0
0.00002	0	0
0.00000285	0	0
6.08E-08	0	0
0	0	0
0	0	0.000000994
0	0	0
0	0	0.000000184
0	0	0
0	0	0
0	0	0
0	0	0
0	0	0
0	0	0.000000262
0.000000142	0	0
0.000000976	0.00000281	0
0	0	9.99E-08
0	0	0
0	0	0
0	0	0
0	0	0
0	0	0
0	0	0
0	0	0.00000569
0	0	0
0	0	0
0	0	0
0	0	0
0	0.000000154	0
0	0	0
0	0	0
0	0	0
0	0	0
0	0.000000171	0
0	0.000000388	0
0	0.000000305	0
0	0	0
0	0	4.65E-08
0	0	4.65E-08

0	0	0.0000298
0	0	0.000000914
0	0	0.00000341
0	0	8.65E-08
0	0	0
0	0	0.000000352
0	0	0
0	0	0
0	0	0
0	0	0.000000306
0	0	0
0	0	0
0	0	0
0	0	0
0	0	0
0	0	0.00000303
0	0	0
0	0	0.00000282
0	0	0
0	0	0.00000138
0	0	0
0	0	0
0	0	0
0	0.00000137	0
0	0	0
0	0	0.000000126
0	0	0.00000031
0	0	0
0	0	0.00000105
0	0	0
0	0	0.000000267
0	0.000000187	0
0	0	0.000000882
0	0	0.000000841
0	0	0.0000574
0	0	0.00000122
0.000000328	0	0
0.000000941	0.00000303	0
0	0	0.000000331
0	0	0
0	0	0
0	0	0
0	0	0
0	0	0.0000221
0	0	0.000000946
0	0	0.00000291
0	0	0

0	0	0
0	0	0.000000485
0	0	0
0	0	0
0	0	0
0	0	0
0	0	0
0	0	0.00000236
0	0	0
0	0	0
0	0	0.000000825
0	0	0
0	0	0.00000149
0	0	0.00000206
0	0	0
0	0	0
0.00000171	0.00000209	0
0	0	0.00000178
0	0	6.96E-08
0.00000026	9.27E-08	0
0.0000025	0	0
3.42E-08	0	0
0	0	0.00000119
0	0	0.0000018
0	0	0.00000711
0	0	0.0000225
0	0	0
0	0	0
0	0	0.000000339
0	0	0
0	0	0
0	0	0
0	0	0
0	0	0
0	0	0
0	0	0
0	0	0
0	0	0
0	0	0.000000421
0	0	0
0	0	0
0	0	0
0	0	0
0	0	0
0	0	0
0	0	0
0	0	0

0	0	0
0	0	0
0	0	0
0	0	0
0	0	0
0	0	0.00000246
0	0	0.00000313
0	0	0.00000608
0	0	0
0	0	0.00000269
0	0	0
0	0	0.000000885
0	0	0
0	0	0.000017
0	0	0.000000227
0	0	0
0	0	0
0	0	0
0	0	4.41E-08
0	0	0
0	0	0
0	0	0
0	0	0
0	0	0.00000934
0	0	0
0	0	0.000000849
0	0	0
0	0	0.00000149
0	0	0
0	0	0
0	0	0.00000213
0	0	6.69E-08
0	0	0
0	0	0
0	0	0.00000345
0	0	0
0	0	0.00000411
0	0	0
0	0	0
0	0	0.0000233
0	0	0
0.000000221	0.000000086	0
0.00000248	0	0
2.77E-08	0	0
0	0	0
0	0	0.00000112
0	0	0

0	0	0.000000212
0	0	0.00000128
0	0	0
0	0	0
0	0	0
0	0	0
0	0	0
0	0	0
0	0	0.000000158
0	0	0
0	0	0
0	0	0
0	0	0.00000108
0	0	0
0	0	0
0	0	0
0	0	0
0	0	0
0	0	0.00000843
0	0	0
0.000000139	0	0
0	0	0
0	0	0
0	0	0
0.000000767	0	0
0.000000439	0	0
0	0	0.0000205
0	0	0
0	0	0
0	0	0
0	0	0
0	0	0
0	0	0
0	0	0
0	0	0
0	0	0
0	0	0.000000544
0	0	0
0	0	0
0	0	0
0	0	0
0	0	0
0	0	0
0	0	0
0	0	0
0	0	0
0	0	0

0	0	0
0	0	0.00000218
0	0	0.00000308
0	0	0.00000623
0	0	0
0	0	0
0	0	0
0.00000207	0.00000262	0
0	0	0
0	0.0000001	0
0.0000189	0	0
0.000000239	6.39E-08	0
0.00000277	0	0
0.000000079	0	0
0	0	0.000000168
0	0	0.00000133
0	0	0
0	0	0
0	0	0
0	0	0
0	0	0
0	0	4.91E-08
0	0	0
0	0.000000216	0
0	0	0.000000137
0	0	0.0000231
0	0	4.94E-08
0	0	0.000000355
0	0	0.000000237
0	0	0
0	0	0
0	0	0
0	0	0
0	0	0
0	0	0
0	0	0
0	0	0.000000663
0	0	0
0	0	0.000000743
0	0	0.00000136
0	0	0
0.0000191	0	0
0.00000011	0	0
0	0	0
0	0	0
0.00000086	0.00000272	0
0	0	0

0	0	0
0	0	0
0	0	0
0	0	0
0	0	0
0	0	0
0	0	0
0	0	0
0	0	0.0000241
0	0.000000119	0
0	0	0.0000138
0	0	5.74E-08
0	0	0
0	0	7.86E-08
0	0	0
0	0.0000018	0
0	0	0
0	0	0.00000216
0	0	0
0	0	0.00000185
0	0	0
0	0	0
0	0	0
0	0	0
0	0	0.000000242
0.00000321	0	0
0.00000338	0	0
0	0	0.00000103
0.000000388	0	0
0.00000115	0.00000361	0
0	0	0
0	0	0.000000661
0	0	0
0	0	0
0	0	0
0	0	0
0	0	0
0	0	0
0	0	0
0	0	0
0	6.64E-08	0
0	0	0.00000127
0	0	0.00000591
0	0	0.0000219
0	0	0
0	0	0
0	0	0.000000341

	0	0	0
	0	0	0
	0	0	0
	0	0	0
	0	0	0
	0	0	0
	0	0	0
	0	0	0
	0	0	0.000000336
	0	0	0
	0	0	0
	0	0	0
	0	0	0
	0	0	0
	0	0	0
	0	0	0
	0	0	0
	0	0	0
	0	0	0
	0	0	0
	0	0	0.00000241
	0	0	0.00000272
	0	0	0.00000603
	0	0	0
	0	0	0.0000026
	0	0	0
	0	0	0.000000696
	0	0	0
	0	0	0.0000179
	0	0	0.000000192
	0	0	0
	0	0	0
	0	0	0
	0	0	4.77E-08
	0	0	0
	0	0	0
	0	0	0.000000366
	0	0	0
	0	0	0.00000933
	0	0	0
	0	0	0
	0	0	0
	0	0	0.00000203
	0	0	7.05E-08
	0	0	0
	0	0	0.00000384
	0	0	0

0	0	0
0	0	0.000024
0.000000231	9.55E-08	0
0.00000267	0	0
0	0	0
0	0	0.00000115
0	0	0
0	0	0.00000019
0	0	0.00000113
0	0	0
0	0	0
0	0	0
0	0	0
0	0	0
0	0	0
0	0	0.000000172
0	0	0
0	0	0
0	0	0
0	0	0
0	0	0
0	0	0.00000113
0	0	0
0	0	0
0	0	0
0	0	0
0	0	0
0	0	0
0	0	0
0	0	0
0	0	4.05E-08
0	0	0
0	0	0.000000934
0	0	0
0	0	0.000000614
0.00000225	0.00000313	0
0	0	0
0	0	0
0	0	0
0	0	0
0	0	0
0	0.000000115	0
0	0	0.0000345
0	0	0
0	0	0
0	0	0.000000119
0	0	0

0	0	0
0	0	2.31E-08
0	0	0
0	0	0.0000219
0	0	8.29E-08
0	0	0
0	0	0.0000201
0	0	0
0	0	0
0	0	0.00000139
0	0	0.000000572
0	0	0
0	0	0.00000784
0	0	0
0	0	0
0	0	0
0	0	0.00000085
0	0	0
0	0	0
0	0	0
0	0	0
0	0	3.37E-08
0	0	0.00000463
0	0	0
0	0	0
0	0	0
0	0	0.00000903
0	0	0.0000334
0	0	0
0	0	0
0	0	0
0.00000117	0.000000881	0
0	0	0.000000181
0	0	0
0	0	0
0	0	0
0	0.000000113	0
0	0	0
0	0	2.19E-08
0.000000429	0.000000506	0
0	0	0.000000761
0	0	0
0	0	0
0	0.000000399	0
0	0	0.0000208
0	0	0
0	0	0.000000353

0	0	0.000000693
0	0	0
0	0	0.0000138
0	0	0.000000317
0	0	0
0	0	0
0	0.000000906	0
0	0	0
0	0	0.000000235
0	0	0
0	0	0
0	0	0.000000538
0	0	0
0	0	0
0	0	0
0	0.000000118	0
0	0	7.54E-08
0	0	0.0000153
0	0	0
0	0	0.000000295
0	0	0.000000266
0	0	0
0	0	0
0	0	0
0	0	0
0	0	0
0	0	0
0	0	0
0	0	0.00000199
0	0	0
0	0	0
0	0	0
0	0	0
0	0	0
0	0	0
0	0	4.67E-08
0	0	0
0	0	0
0	0	0
0	0	0.0000106
0	0	0.0000178
0	0	0
0	0	0
0	0	0
0	0.000000219	0
0	0	0.0000254
0.000000101	0	0

0.000000085	0	0
0	0.000000233	0
0	0.00000015	0
0	0	0
0	0	0
0	0.000000133	0
0.000000159	0	0
0	0	0
0	0	0
0	0	0
0	0	0.0000098
0	0	0.00000318
0	0	0
0	0	0
0	0	0
0	0	0.000000739
0	0	0
0	0	0
0	0	0
0	0	0
0	0	0
0	0	0
0	0	0
0	0	0.0000112
0	0	0
0	0	0.0000207
0	0	0
0	0	0.000000783
0	0	0
0	0	0
0	0	0
0	0	0
0	0	0
0	0	0
0	0	0
0	0	0
0	0	0
0	0	0
0	0	0
0	0	0
0	0	0
0	0	0.000000404
0	0	0
0	0	0
0	0	0
0	0	0
0	0	0

	0	0	0
	0	0	0
	0	0	0
	0	0	0
	0	0	0.00000201
	0	0	0.00000248
	0	0	0.00000513
	0	0	0
	0	0	0.0000228
	0	0	0
	0	0	0
	0	0	0.00000187
	0	0	0
	0	0	0
	0	0	0
	0	0	0
	0	0	0
	0	0	0
	0	0	6.59E-08
	0	0	0.0000013
	0	0	0
	0	0	0
	0	0	0
	0	0	0
	0	0	0
	0	0	0.00000238
	0	0	0
	0	0	0
	0	0	0.00000156
	0	0	0
	0	0	0.0000182
	0	0	8.04E-08
	0	0	3.91E-08
	0	0	0
	0	0	0.00000335
	0	0.000000151	0
	0	0	0.000000566
	0	0	0
	0	0	0
	0	0	0.000000858
	0	0	7.83E-08
	0	0	0.0000188
	0	0	0
	0	0	0
	0	0	0
	0	0	0.000000203
	0	0	0

0	0	0
0	0	0
0	0	0
0	0	0.000000266
0	0	0
0	0	0
0	0	0
0	0	0
0	0	0
0	0	0
0	0	0.00000474
0	0	0
0	0	0
0	0	0.00000597
0	0	0.0000233
0	0	0
0	0	0
0	0	0.00000997
0	0	0.00000652
0	0	0
0	0	0
0	0	0
0	0	0
0	0	0
0	0	0
0	0	0
0	0	0
0	0	0
0	0	0
0	0	0.000000666
0	0	0
0	0	0
0	0	0
0	0	0
0	0	0
0	0	0
0	0	0.00000244
0	0	0.00000265
0	0	0.0000056
0	0	0.00000228
0	0	0
0	0	0
0.00000571	0.000000675	0
0	0	0
0	0	0.000000598
0	0	0
0	0	0

0	0	0.00000366
0	0	0
0	0	0
0	0	0
0	0	0
0	0	0.00000121
0	0.00000104	0
0	0	0
0	0	0
0	0	0
0	0	0
0	0	0.000000487
0	0	0
0	0	0
0	0	0.000000749
0	0	0
0	0	0.00000165
0	0	0
0	0	0.00000167
0	0	4.97E-08
0	0	5.19E-08
0	0	0
0	0	0
0	0	0.00000017
0	0	0
0	0	0
0	0	0
0	0	5.02E-08
0	0	0
0	0	0.000000125
0	0.0000001	0
0	0	0
0	0	0
0	0	4.78E-08
0	0	0.0000242
0	0	0
0.0000032	0	0
5.07E-08	0	0
0	0	0
0	0	0
0	0	0.00000104
0	0	0
0	0	0.000000145
0	0	0.000000917
0	0	0
0	0	0
0	0	0

0	0	0
0.000000307	0	0
0.00000115	0.00000402	0
0	0	0.000000192
0	0	0
0	0	0
0	0	0
0	0	0
0	0	0
0	0	0
0	0	0
0	0	0
0	0	0
0	0	0.00000561
0	0	0
0	0	0
0	0	0
0	0	0
0	0	0.00000309
0	0	0
0	0	0
0	0	0
0	0	0
0	0	0
0	0	0
0	0	0
0	0	0
0	0	0
0	0	0
0	0	0
0	0	0
0	0	0
0	0	0
0	0	0
0	0	0
0	0	0
0	0	0
0	0	0.00000229
0	0	0.00000228
0	0	0
0	0	0.00000239
0	0	0.00000107
0	0	0
0	0	0
0	0	0.000000374
0	0	0
0	0	0

0	0.000000202	0
3.46E-08	0	0
0	0	0.00000112
0	0	0.000000255
0	0	0.00000103
0	0	0
0	0	0
0	0	0
0	0	0.000000153
0	0	0
0	0	0
0	0.000000198	0
0	0	0
0.000000527	0.000000544	0
0	0	0
0	0	0
8.86E-08	0	0
8.86E-08	0	0
0	0	0
0	0	0
0	0	0
0	0	0
0	0	0
0	0	0
0	0	0
0	0	0
0	0	0
0	0	0
0	0	0
0	0	0
0	0	0
0	0	0
0	0	0
0	0	0
0	0	0
0	0	0
0	0	0
0	0	0
0	0	0
0	0	0
0	0	0
0.000000144	0	0
0	0	0
0	0	0
0	0	0
0	0	0
0	0	0

0	0	0
0	0	0
0	0.000000247	0
0	0	0
0	0	0.00000948
0	0	0.0000252
0	0	0
0	0	0.0000102
0	0	0.00000543
0	0	0
0	0	0.000000138
0	0	0
0	0	0.000000225
0	0	0
0	0	0.000000679
0	0	0.000000052
0	0	0
0	0	0
0	0	0.000014
0	0	0.00000151
0	0	0.000000312
0	0	0
0	0	0
0	0	0
0	0	0
0	0	0.00000005
0	0	0
0	0	0
0	0	0.00000102
0	0	0
0	0	0
0	0	0
0	0	0
0	0	0.000000989
0	0	0
0	0	0
0	0	0.00000226
0	0	0.00000178
0	0	0
0	0	0.00000125
0	0	0
0.0000022	0.00000281	0
0	0	0
0	0	0
0	0	0
0	0	0
0	0	0

0	0	0.000000174
0	0	5.31E-08
0	0	0
0	0	5.09E-08
0	0	0
0	0	0.000000118
0	0.000000181	0
0	0	0.00000089
0	0	0
0	0	0.000000608
0	0	0.000022
0	0	0
0.00000401	0	0
0	0	0
0.000000113	0	0
0	0	0
0	0	0.000000727
0	0	0
0	0	0.000000273
0.000000693	0	0
0.000000502	0.00000243	0
0	0	0.000000249
0	0	0
0	0	0
0	0	0
0	0	0
0	0	0
0	0	0
0	0	0
0	0	0
0	0	0
0	0	7.86E-08
0	0	0.00000647
0	0	0
0	0	0
0	0	0.0000102
0	0	0.0000257
0	0	0
0	0	0.0000103
0	0	0.00000544
0	0	0
0	0	0.000000122
0	0	0
0	0	0.000000211
0	0	0
0	0	0
0	0	0

0	0	0
0.00000632	0.000000376	0
0	0	0
0	0	0.000000657
0	0	4.85E-08
0	0	0
0	0	0
0	0	0.000014
0	0	0.00000135
0	0	0.000000289
0	0	0
0	0	0
0	0	0
0	0	0
0	0	4.38E-08
0	0	0
0	0	2.66E-08
0	0	0.000000971
0	0.00000116	0
0	0	0
0	0	0
0	0	0
0	0	0
0	0	0.000000999
0	0	0
0	0	0
0	0	0.00000233
0	0	0.0000017
0	0	0
0	0	0.00000143
0	0	0
0	0	0
0.00000231	0.00000291	0
0	0	0
0	0	0
0	0	7.85E-08
0	0	0
0	0	0
0	0	0
0	0	0.000000186
0	0	4.46E-08
0	0	0
0	0	5.26E-08
0	0	0
0	0	0.000000127
0	0.000000211	0
0	0	0.000000878

0	0	0
0	0	0.00000087
0	0	9.59E-08
0	0	0.0000234
0	0	0
0.000000221	0.000000137	0
0.00000431	0	0
0	0	0
0	0	0
0	0	0.000000923
0	0	0
0	0	0.000000312
0.00000069	0	0
0.000000471	0.00000267	0
0	0	0.000000243
0	0	0
0	0	0
0	0	0
0	0	0
0	0	0
0	0	0.000000375
0	0	0
0	0	0
0	0	0
0	0	9.47E-08
0	0	9.04E-09
0	0	0.00000639
0	0	0
0	0	0
0	0	0
0	0	0
0	0	0
0	0	0.000000754
0	0	0
0	0	0
0	0	0
0	0	0
0	0	0
0	0	0
0	0	0.00000304
0	0	0
0	0	0
0	0.00000138	0
0	0	0.000000762
0	0	0
0	0	0.00000839
0	0	0

0	0	0
0	0	0
0	0.000000116	0
0	0	0.0000258
0.000000204	8.39E-08	0
9.11E-08	0	0
0	0	0
0	0	0
0	0	0
0	0	0
0	0	0.000000734
0	0	0
0	0	0
0	0	0
0	0	0
0	0	0
0	0	0
0	0	0.00000301
0	0	0
0	0	0
0	0.00000134	0
0	0	0
0	0	0.000000591
0	0	0
0	0	0.00000826
0	0	0
0	0	0
0	0	0
0	0	3.15E-08
0	0.000000135	0
0	0	0.000027
0.000000195	8.38E-08	0
0.00000108	9.72E-08	0
8.37E-08	0	0
0	0	0
0	0	0
0	0.000000342	0
0	0	0
0	0	0
0	0	0
0	0	0
0	0	0
0	0	0.000000421
0	0	0
0	0.00000149	0
0	0	0.000000877
0	0	0

	0	0	0.000000612
	0	0.00000014	0
	0	0	0.000000309
	0	0	0
	0	0.000000084	0
	0	0	0
	0	0	0
	0	0	0
	0	0	0
	0	0	0
	0	0	0
	0	0	0
	0	0	0
	0	0	0
	0	0	0
	0	0	0
	0	0.00000172	0
	0	0	0
	0	0	0
	0	0	0.00000129
	0	0	0
	0	0	0.000000683
	0	0	0
	0	0	0.000000247
	0	0.000000123	0
	0	0	0
	0	0	0
	0	0	6.01E-08
	0	0	0
	0	9.06E-08	0
	0	0	0
	0	0	0
	0	0	0.000000551
	0	0	0
	0	0	0
	0	0	0
	0	0	0
	0	0	0
	0	0	0
	0	0	0
	0	0	0
	0	0	0
	0	0	5.58E-08
	0	0	0
	0	0	0.000000149
	0	0	0
	0	0	0

0	0	0
0	0	0
0	0	0
0	0	0
0	0	0
0	0	0
0	0	0.00000212
0	0	0.0000058
0	0	0.00000314
0	0	0
0	0	0
0.00000662	0.000000477	0
0	0	0
0	0	0
0	0	0
0	0	0.0000188
0	0	0
0	0	0
0	0	0
0	0	0
0	0	0
0	0	0
0	0	0
0	0	0
0	0	4.05E-08
0	0	0.00000136
0	0.0000017	0
0	0	0
0	0	0
0	0	0
0	0	0
0	0	0
0	0	0.0000103
0	0	0.00000265
0	0	0.00000174
0	0	0
0	0	0
0	0	0
0	0	0
0	0	0.0000195
0	0	0
0	0	0
0	0	0
0	0	0
0	9.81E-08	0
0	0	0
0	0	0.000028

0	0	0
0.000000108	8.14E-08	0
0.00000279	0	0
0.00000105	0.000000172	0
0	0	0
0.0000439	0.0000136	0
0	0	0
0	0	0.00000122
0	0	0
0	0	0.000000482
0	0	0
0	0	0
0	0	0
0	0	0
0	0	0
0	0	0
0	0	0
0	0	0
0	0	0
0	0	4.78E-08
0	0	0.00000546
0	0	0.0000218
0	0	0
0	0	0.00000331
0	0	0
0	0	0
0	0	0.000000469
0	0	0
0	0	0.000000811
0	0	0
0	0	0
0	0	0.0000194
0	0	0.000000293
0	0	0
0	0	0
0	0	0
0	0	0
0	0	0
0	0	0
0	0	0
0	0	0
0	0	0
0	0	0
0	0	0
0	0	0.00000172
0	0	0
0	0	0

0	0	0.00000186
0	0	0
0	0	0
0	0	0.0000218
0	0	0
0	0	0
0	0	3.52E-08
0	0.000000156	0
0	0	0.000000654
0	0	0
0	0	0.000000821
0	0	0.000000076
0	0	0.0000254
0	0	0
0	0	0
0	0	0.000000322
0	0	0.000000263
0	0	0
0	0	0
0	0	0
0	0	0
0	0	0
0	0	0
0	0	0
0	0	0
0	0	0
0	0	0
0	0	0
0	0	0
0	0	0.00000257
0	0	0
0	0	0
0	0	0.000016
0	0.000000163	0
0	0	0
0.0000075	0.00000773	0
0	0	0
0	0	0.0000144
0	0	0
0	0	0
0	0	0
0	0	0
0	0	0.0000182
0	0	0
0	0.000000255	0
0	0	0.0000259
0	0	0

0	0	0
0	0	0.000000142
0	0	0.00000153
0	0	0
0	0	0
0	0	0
0	0	0.0000102
0	0	0.0000257
0	0	0
0	0	0.0000103
0	0	0.00000544
0	0	0
0	0	0.000000122
0	0	0
0	0	0.000000211
0	0	0
0	0	0
0	0	0
0	0	0
0.00000632	0.000000376	0
0	0	0
0	0	0.000000657
0	0	4.85E-08
0	0	0
0	0	0
0	0	0.000014
0	0	0.00000135
0	0	0.000000289
0	0	0
0	0	0
0	0	0
0	0	0
0	0	4.38E-08
0	0	0
0	0	2.66E-08
0	0	0.000000971
0	0.00000116	0
0	0	0
0	0	0
0	0	0
0	0	0
0	0	0.000000999
0	0	0
0	0	0
0	0	0.00000233
0	0	0.0000017
0	0	0

0	0	0.00000143
0	0	0
0	0	0
0.00000231	0.00000291	0
0	0	0
0	0	0
0	0	7.85E-08
0	0	0
0	0	0
0	0	0
0	0	0.000000186
0	0	4.46E-08
0	0	0
0	0	5.26E-08
0	0	0
0	0	0.000000127
0	0.000000211	0
0	0	0.000000878
0	0	0
0	0	0.00000087
0	0	9.59E-08
0	0	0.0000234
0	0	0
0.000000221	0.000000137	0
0.00000431	0	0
0	0	0
0	0	0
0	0	0.000000923
0	0	0
0	0	0.000000312
0.00000069	0	0
0.000000471	0.00000267	0
0	0	0.000000243
0	0	0
0	0	0
0	0	0
0	0	0
0	0	0
0	0	0.000000375
0	0	0
0	0	0
0	0	0
0	0	9.47E-08
0	0	9.04E-09
0	0	0.00000639
0	0	0
0	0	0

0	0	0
0	0	0
0	0	0
0	0	0
0	0	0
0	0.000000274	0
0	0	0
0	0	0.0000243
0	0	0
0	0	0
0	0	0.0000024
0	0	0
0	0	0.00000083
0	0	0
0	0	0
0	0	0
0	0	0
0	0	0
0	0	0.0000189
0	0	0
0	0	0
0	0	0
0	0	0
0	0	0
0	0	0
0	0	4.52E-08
0	0	0.000000079
0	0	0.0000324
0.000000157	0	0
0	0	0
0	0	0.000000503
0	0	0.000000044
0	0	3.73E-08
0	0	0
0	0	0.000000611
0	0	0
0	0	0
0	0	0
0	0	0
0	0	0
0	0	0
0.0000073	0.000000563	0
0	0	0
0	0.00000147	0
0	0	0.000000744
0	0	0
0	0	0.00000101

0	0	0
0	0	0
0.000023	0.000000134	0
0	0	0.00000106
0	0	0.000000172
0	0	0
0	0	0
0	0	0
0	0	0
0	0	0
0	0	0.00000827
0	0	0
0	0	0
0	0	0.0000114
0	0	0.0000065
0	0	4.39E-08
0	0	0
0	0	0.000000453
0	0	0
0	0	0
0	0	0.000000568
0	0	0
0	0	0
0	0	0
0	0	0
0	0	0
0	0	0
0	0	0
0	0	0.00000587
0	0	0
0	0	0.00000227
0	0	0
0	0	0
0	0	0
0.00000647	0.000000506	0
0	0	0.000000865
0	0	0
0	0	0
0	0	0
0	0	0.00000834
0	0	0.000000252
0	0	0
0	0	0
0	0	0
0	0	0.00000125
0	0.00000161	0
0	0	0

0	0	0
0	0	0
0	0	0
0	0	0
0	0	0
0	0	0.00000231
0	0	0.000000948
0	0	0
0	0	0.000000934
0.00000221	0.00000275	0
0	0	0
0	0	0
0	0	0
0	0	5.39E-08
0	0	0
0	0	0
0	0	0.000000241
0	0	0
0	0	0
0	0	0.000000704
0	0	0
0	0	0
0	0	0.000000738
0	0	6.12E-08
0	0	0.0000234
0	0	0
0	0	0
0.0000215	0	0
0.00000294	0	0
8.58E-08	0	0
0	0	0
0	0	0.00000104
0	0	0
0	0	0.000000168
0	0	0.00000108
0	0	0
0	0	0
0	0	0
0	0	0
0	0	0
0	0	0.000000291
0.000000148	0	0
0.00000105	0.00000308	0
0	0	9.73E-08
0	0	0
0	0	0
0	0	0

0	0	0
0	0	0
0	0	0
0	0	4.23E-08
0	0	0.00000667
0	0	0
0	0	0
0	0	0.00000841
0	0	0
0	0	0
0	0	0.0000113
0	0	0.00000648
0	0	5.45E-08
0	0	0
0	0	0.000000436
0	0	0
0	0	0
0	0	0
0	0	0.000000561
0	0	0
0	0	0
0	0	0
0	0	0
0	0	0
0	0	0
0	0	0
0	0	0
0	0	0
0	0	0
0	0	0
0	0	0.00000589
0	0	0
0	0	0.00000226
0	0	0
0	0	0
0	0	0
0	0	0
0.000007	0.00000054	0
0	0	0.000000821
0	0	0
0	0	0
0	0	0
0	0	0.00000392
0	0	0.000000235
0	0	0
0	0	0
0	0	0

0	0	0.00000117
0	0.00000156	0
0	0	0
0	0	0
0	0	0
0	0	0
0	0	0
0	0	0
0	0	0.00000224
0	0	0.000000887
0	0	0
0	0	0.000000965
0	0	0
0	0	0
0	0	0.00000144
0	0	0
0	0	5.07E-08
0	0	0
0	0	0
0	0	0.00000024
0	0	0
0	0	0
0	0	0
0	0	0.000000639
0	0	0
0	0	0
0	0	0.000000708
0	0	4.83E-08
0	0	0.00002
0	0	0
0	0	0
0.0000203	0	0
0.00000289	0	0
6.98E-08	0	0
0	0	0
0	0	0.000000977
0	0	0
0	0	0.000000177
0	0	0.00000106
0	0	0
0	0	0
0	0	0
0	0	0
0	0	0
0	0	0.000000273
0.00000013	0	0
0.00000101	0.00000302	0

0	0	8.73E-08
0	0	0
0	0	0
0	0	0
0	0	0
0	0	0
0	0	0
0	0	0.00000654
0	0	0
0	0	0
0	4.12E-08	0
0	0	0
0	0	0
0	0	0
0	0	0
0	0	0
0	0	0
0.000000288	0.000000063	0
0	0	0
0	0	0
0	0	0
0	0	0
0	0	0
0	0.000000178	0
0	0	0
0	0	0
0	0	0
0	0	0.0000145
0	0	0
0	0	6.93E-08
0.000000312	0.000000149	0
0.00000127	0.000000123	0
0	0	0
0	0	0
0	0	0.00000044
0.0000062	0	0
0.000000148	0	0
0	0	0.000000943
0	0	0
0	0	0.000000636
0	0	0
0	0	0
0	0	0
0	0	0
0	0	0.0000298
0	0	0.00000142
0	0	0.000000876

0	0	0.0000008
0	0	0.00000092
0	0	0.000000772
0	0.000000238	0
0	0	0.00000112
0	0	0
0	0	0.000000617
0	0	0.00000252
0	0	7.52E-08
0	0	0.000000346
0	0	0
0	0	0
0	0	0.000000452
0	0	0
0	0	0
0	0	0
0	0	0
0	0	0
0	0	0.00000312
0	0	0
0	0	0
0	0	0
0	0	0
0	0	0
0	0	0
0	0.00000189	0
0	0	0
0	0	0.000000409
0	0	0.000000564
0	0	0
0	0	0.000000539
0	0	0
0	0	0.00000182
0	0	0
0	0	0
0	0	0.000000242
0	0.000000157	0
0	0	0
0	0	0.0000152
0.000000202	0.000000126	0
0	0	0.000000943
0.000000918	0	0
0	0	0
0	0	0
0	0	0
0	0	9.82E-08
9.32E-08	0	0

	0	0	0
	0	0.000000123	0
	0	0	0
	0	0	0
	0	0	0.00000245
	0	0	0
	0	0.000000119	0
	0	0	0
	0	0	0
	0	0.000000174	0
	0	0	0.00000647
	0	0	0.0000213
	0	0	0
	0	0	0
	0	0	0
	0	0	0
	0	0	0
	0	0	0
	0	0	0
	0	0	0
	0	0	0
	0	0	0.000000393
	0	0	0
	0	0	0
	0	0	0
	0	0	0
	0	0	0
	0	0	0
	0	0	0
	0	0	0
	0	0	0
	0	0	0
	0	0	0
	0	0	0
	0	0	0.0000024
	0	0	0.00000215
	0	0	0.00000558
	0	0	0
	0	0	0.00000285
	0	0	0.000000814
	0	0	0
	0	0	0
	0	0	0.00000118
	0	0	0.000000163
	0	0	0
	0	0	0
	0	0	0

0	0	0
0	0	0
0	0	0.000000163
0	0	0
0	0	0.00000988
0	0	0
0	0	0
0.00000209	0.00000259	0
0	0	0
0	0	0
0	0	0.0000174
0	0.000000126	0
0	0	0.0000182
0.00000289	0.0000164	0
0	0	0
0	0	0.000000872
0	0	0
0	0	0.000000141
0	0	0
0	0	0
0	0	0
0	0	0
0	0	0.0000002
0	0	0.000000342
0	0	0
0	0	0
0	0	0
0	0	0
0	0	0
0	0	0
0	0	0
0	0	0.00000902
0	0	0.0000231
0	0	0.0000091
0	0	0
0	0	0
0	0	0
0	0	0
0	0	0
0	0	0
0	0	0.000000107
0	0	0
0	0	0.000000687
0	0	0
0	0	0
0	0	0
0	0	0

	0	0	0
	0	0	0
	0	0	0
	0	0	0
	0	0	0
	0	0	0
	0	0	0
	0	0	0
	0	0	0.00000174
	0	0	0.00000277
	0	0	0.00000456
	0	0	0
	0	0	0.00000207
	0	0	0.000000619
	0	0	0
	0	0	0
	0	0	0
	0	0	0
	0	0	0
	0	0	0
	0	0	0
	0	0	0
	0	0	0
	0	0	0
	0	0	0.0000233
	0	0	0.000000226
	0	8.33E-08	0
	0	0	0.0000209
	0	0	0
	0	0	0.000000727
	0	0	0
	0	0	0
	0	0	0
	0	0	0
	0	0	0.000000311
	0	0	0
	0	0	0.00000111
	0	0	0
	0	0	0
	0	0	0
	0	0	0
	0	0	0
	0	0	0
	0	0	0
	0	0	0.0000218
	0	0	0
	0	0	0

0	0	0
0	0	0.000000337
0.0000065	0.000000533	0
0	0	0
0	0	0
0	0	0.000023
0	0	0
0	0	0
0	0	0
0	0	4.78E-08
0	0	0
0	0	0
0	0	0
0	0	0.000000147
0	0	0.00000252
0	0.00000129	0
0	0	0
0	0	0
0	0	0.000000914
0	0	0.00000292
0	0	0
0	0	0.0000266
0	0	0.00000035
0	0	0
0	0	0
0	0	2.12E-08
0	0	0.000000764
0	0	0
0	0	0.000000635
0	0	0.000000095
0	0	0.0000391
0	0	1.96E-08
0	0	0
0	0	0
0	0	0
0	0	0
0	0	0
0	0	0
0	0	6.18E-08
0	0	0.00000747
0	0	8.24E-08
0	0	7.52E-08
0	0	0
0	0	0
0	0.00000026	0
0	0	0
0	0	0

8.08E-08	0	0
7.24E-08	0	0
0	0	0.000000105
0	9.92E-08	0
0	0	0
0	0.000000106	0
0	0	0
0	0	0
0	0	0
0	0.000000114	0
0	0	0
0	0.000000176	0
0	0	0
0	0	0.00000011
0	0	0
0	0	0
0	0	0.00000177
0	0	0.00000157
0	0	0
0	0	0
0	0.000000124	0
0	0	0.0000246
0.000000299	0.000000107	0
0	0	0
0	0	0
0	0	0
0	0	0
0	0	0
0.00000017	0	0
0.000000161	0	0
0	0	0
0	0	0
0	0.000000194	0
0.0000001	0.000000197	0
0	0	0
0	0	0
0	0	0.0000217
0	0	0
0	0	0.00000117
0	0	0.000000113
0	0	0
0	0	0
0	0	0
0	0	0
0	0	0
0	0	0
0	0	0

0	0	0
0	0	0
0	0	0
0	0	0
0	0	0
0	0	0.00000232
0	0	0
0	0	0.00000284
0	0	0.000000465
0	0	0.000000069
0	0	0
0	0	0
0	0	0.0000234
0	0	0.000000368
0	0	0
0	0	0
0	0	0
0	0	0
0	0	0
0	0	0.00000012
0	0	0
0	0	0
0	0	0
0	0	6.01E-08
0	0	0.0000015
0	0	0
0	0	0
0	0	0
0	0	0.0000011
0	0	0
0	0	0.0000077
0	0	0.00000191
0	0	0
0	0	0.00000219
0	0	0
0	0	0
0	0	0.00000323
0	0	7.72E-08
0	0	7.54E-08
0	0	0
0	0	0
0	0	0.0000193
0	0	0.000000184
0	0	0
0	0	0
0	0	0
0	0	0

	0	0.000000172	0
	0	0	0.000000664
	0	0	0
	0	0	0
	0	0	0.000000646
	0	0	8.61E-08
	0	0	0.000000227
	0	0	0
	0	0	0.000000858
	0	0	0
	0	0	0.00000018
	0	0	0.00000108
	0	0	0
	0	0	0
	0	0	0
	0	0	0
	0	0	0
	0	0	0
	0	0	0.000000293
	0	0	0.00000028
	0	0	0
	0	0	0
	0	0	0
	0	0	0
	0	0	0
	0	0	0
	0	0	0
	0	0	5.65E-08
	0	0	0.00000448
	0	0	0
	0	0	0
	9.77E-08	0	0
	0	0	0
	0	0	0.000000367
	0	0	0
	0	0	0.000000589
	0	7.98E-08	0
	0	0	0.000000163
	0	0	0
	0	0	0
	0	8.21E-08	0
	0	0.000000402	0
	0	0	0.00000712
	0	0	0.0000223
	0	0	0.000000952
	0	0	0.000000334
	0	0	0

0	0	0
0	0	0
0	0	0
0	0	0.00000102
0	0	0
0	0	0.00000057
0	0	0
0	7.34E-08	0
0.000000226	0.000000192	0
0	0	0
0	0	0.000000311
0.00000133	0.00000307	0
0	0	0
0	0	0
0	0	0
0	0	0
0	0	0
0	0	0
0	0	0
0	0	0
0	0	0
0	0	0
0.0000025	0.0000034	0
0	0	0
6.52E-08	0	0
0	0	0
0	0	0
0	0	0
0	0	0
0	0.000000363	0
0	0	0.00000739
0	0	0.0000213
0	0	0.000000335
0	0	0
0	0	0.00000299
0	0	0.000000165
0	0	0
0	0.00000161	0
0	0	0
0	0	0
0	0	0.000000165
0	0	0.00000902
0	0	0
0	0	0
0	0	0
0	0	0
0	0	0.00000344

0	0.000000121	0
0.000000204	0.00000011	0
0	0	0
0	0	0
0	0	0.000000324
0.00000136	0.0000033	0
0	0	0
0	0	0
0	0	0
0	0	0
0	0	0
0	0	0
0	0	0
0	0	0.000000604
0	0	0.00000919
0	0	0.0000279
0	0	0
0	0	0
0	0	0.0000101
0	0	0.00000636
0	0	0.000000712
0	0	0.00000304
0	0	0
0	0	0.000000124
0	0	0
0	0	0.000000481
0	0	0
0	0	0
0	0	0
0	0	0
0	0	0
0	0	0
0	0	0
0	0	0
0	0	0
0	0	0.00000076
0	0	0
0	0	0
0	0	0
0	0	0
0	0	0
0	0	0
0	0	0
0	0	0
0	0	0
0	0	0
0	0	0

0	0	0
0	0	0
0	0	0.00000321
0	0	0.00000372
0	0	0.00000767
0	0	0
0	0	0.00000286
0	0	0
0	0	0
0	0	0
0	0	0
0.0000077	0.000000681	0
0	0	0
0	0	0.00000104
0	0	8.61E-08
0	0	0
0	0	0
0	0	0.0000256
0	0	0.000000224
0	0	0
0	0	0
0	0	0
0	0	0
0	0	0
0	0	0.000000119
0	0	0
0	0	0
0	0	0
0	0	4.87E-08
0	0	0.00000131
0	0.00000241	0
0	0	0
0	0	0
0	0	0
0	0	0
0	0	0
0	0	0
0	0	0.000000774
0	0	0
0	0	0.0000114
0	0	0
0	0	0.00000212
0	0	0.00000232
0	0	0
0	0	0.000000984
0	0	0
0	0	0

0.00000343	0.00000391	0
0	0	0
0	0	0
0	0	0.00000187
0	0	0.000000157
0	0	5.09E-08
0	0	0
0	0	0
0	0	0
0	0	0.0000226
0	0	0.000000268
0	0	0.000000025
0	0	0
0	0	0
0	0	0.000000146
0	0	0
0	0	0.00000478
0	0	0.0000003
0	0.00000012	0
0	0	0.000000795
0	0	0
0	0	0
0	0	0.00000083
0	0	5.39E-08
0	0	0.0000394
0	0	0.000000101
0	0	0
0.000000378	8.81E-08	0
0.00000334	0	0
0	0	0
0.00000112	0.000000158	0
0	0	0
0	0	0
0	0	0
0	0	0.00000117
0	0	0
0	0	0.000000215
0	0	0.00000156
0	0	0
0	0	0
0	0	0
0	0	0
0	0	0
0	0	0
0	0	0.00000032
0.00000053	0	0
0.000000981	0.00000325	0

	0	0	0.000000347
	0	0	0
	0	0	0
	0	0	0
	0	0	0
	0	0	0
	0	0	0
	0	0	0.000000994
	0	0	0
	0	0	0
	0	0	0
	0	0	0
	0	0	6.62E-08
	0	0	0
	0	0	0
	0	0	0.000000105
	0	0	0.0000075
	0	0	0
	0	0	0
	0	0.00000032	0
	0	0	0
	0	0	0
	0	0	0
	0	0	0
	0	0	0
	0	0	0
	0	0	0.000000581
	0	0	0
	0	0	0
	0	0	0.000000143
	0	0.00000149	0
	0	0	0
	0	0	0
	0	0	0.000000909
	0	0	0
	0	0	0.00000057
	0	0	0
	0	0	0.000000208
	0	0.000000137	0
	0	0	0
	0	0	0.000000452
	0	0	0
	0	0	0.00000014
	0	0	0
	0	5.83E-08	0
	0	0	0
	0	0	0

	0	0.000000371	0
	0	0	0.00000839
	0	0	0.0000246
	0	0	0
	0	0	0
	0	0	0.0000105
	0	0	0.00000597
	0	0	0.000000334
	0	0	0.00000233
	0	0	0
	0	0	2.34E-08
	0	0	0.000000256
	0	0	0
	0	0	0
	0	0	0
	0	0	0
	0	0	0
	0	0	0
	0	0	0
	0	0	0.000000867
	0	0	0
	0	0	0
	0	0	0
	0	0	0
	0	0	0
	0	0	0
	0	0	0
	0	0	0
	0	0	0
	0	0	0
	0	0	0
	0	0	0
	0	0	0.00000323
	0	0	0
	0	0	0.00000214
	0	0	0
	0	0	0
	0	0	0
	0.00000843	0.000000457	0
	0	0	0
	0	0	0.000000881
	0	0	0
	0	0	0
	0	0	0
	0	0	0.0000167
	0	0	0.00000108
	0	0	0.000000407

0	0	0
0	0	0
0	0	0
0	0	7.33E-08
0	0	0
0	0	0
0	0	0
0	0	6.34E-08
0	0	0.00000127
0	0.00000151	0
0	0	0
0	0	0
0	0	0
0	0	0.000000121
0	0	0
0	0	0
0	0	0.00000169
0	0	0
0	0	0.000000901
0	0	0
0	0	0
0.00000303	0.00000328	0
0	0	0
0	0	0
0	0	0.0000194
0	0	0.00000018
0	0	0
0	0	0
0	0	0
0	0	0.000000193
0	0.000000167	0
0	0	0.000000867
0	0	0
0	0	0
0	0	0.000000697
0	0	0.000000189
0	0	0
0	0	0
0.000000397	7.81E-08	0
0	0	0
6.86E-08	0	0
0	0	0
0	0	0
0	0	0
0	0	0.00000074
0	0	0
0	0	0.000000321

0	0	0.00000159
0	0	0
0	0	0
0	0	0
0	0	0
0	0	0.000000321
0.000000316	0	0
0.00000114	0.0000032	0
0	0	0.000000201
0	0	0
0	0	0
0	0	0
0	0	0
0	0	0
0	0	0
0	0	0
0	0	0
0	0	0
0	0	9.48E-08
0	0	0
0	0	0
0	0	0
0	0	0
0	0.00000032	0
0	0	0
0	0	0
0	0	0
0	0	0
0	0	0
0	0	0
0	0	0.000000581
0	0	0
0	0	0
0	0	0.000000143
0	0.00000149	0
0	0	0
0	0	0
0	0	0.000000909
0	0	0
0	0	0.00000057
0	0	0
0	0	0.000000208
0	0.000000137	0
0	0	0
0	0	0.000000452
0	0	0
0	0	0.00000014

0	0	0
0	5.83E-08	0
0	0	0
0	0	0
0	0.000000359	0
0	0	0.0000241
0	0	0
0	0	0
0	0	0
0	0	0
0	0	0
0	0	0
0	0	0
0	0	0
0	0	0
0	0	0.000000742
0	0	0.000000454
0	0	0
0	0.00000151	0
0	0	0
0	0	0
0	0	0.00000126
0	0	0
0	0	0.000000762
0	0	0
0	0.000000144	0
0	0	0
0	0	0.000000272
0	0	0.00000125
0	0	0
0	0	0
0	0	0
0	0	0.000000365
0.00000115	0.00000301	0
0	0	0
0	0	0
0	0	0
0	0	0
0	0	0
0	0	0
0	0	0
0	0	0
0	0	0
0	0	0
0	0	0
0	0	0
0	0	0

0	0	0
0	0.0000018	0
0	0	0
0	0	0
0	0	0.00000141
0	0	0
0	0	0.000000713
0	0	0
0	0	0.000000275
0	0.000000128	0
0	0	0
0	0	0
0	0	7.14E-08
0	0	0
0	0	0
0	0	0
0	0	0
0	0	0.000000625
0	0	0
0.000000196	0.000000077	0
0.0000385	0.0000123	0
9.11E-08	0	0
0	0	0
0	0	0
0	0.00000198	0
0	0	0
0	0.00000019	0
0	0	0.0000153
0	0	0
0	0	0
0.000000107	0	0
0	0	0.000000344
0	0	0
0	0	0
0	0	0
0.00000272	0	0
0.00000268	0.000017	0
0	0	0
0	0	0
0	0	0
0	0	0
0.000000322	0.000000305	0
0	0	0
0	0	0
0	0	0
0	0	0.000000401
0	0	0

0	0	0
0	0	0
0	0	0
0	0	0
0	0	0
0	0	0
0.000000172	0	0
9.34E-08	0	0
0	0	0
0	0	0
0	0	0
0	0	0.000000543
0	0	0
0	0	0
0	0	0
0	0	0
0	0	0
0.000000716	0	0
0.000000417	0	0
0	0	4.99E-08
0	0	0.000000359
0	0	0
0	0	0.00000366
0	0	0
0	0	4.49E-08
0	0	0.000000604
0.00000207	0.00000293	0
0	0	0.00000192
0	0	0
0	0	0
0	0	0
0	0.000000411	0
0	0	0.000018
6.33E-08	0	0
0.0000015	0.00000389	0
0	0	0
0	0	0
0	0	0
0	0	0
0	0	0
0	0	0
0	0	0.00000162
0	0	0
0	0	0
0	0	0.000000399
0	0	0
0	0	0

	0	0	0.000000105
	0	0	0
	0	0	0
	0	0	0
	0	0	0
	0	0	0
	0	0.000000296	0
	0	0	0.000000179
	0	0	0.000000535
	0	0	0.000000292
	0	0	0.000000655
	0	0	0
	0	0	0
	0	0	0
	0	0	0.000000375
	0	0	0
	0	0	0
	0	0	0
	0	0	0
	0	0	0
	0	0	0
	0	0	0
	0	0	0
	0	0	0
	0	0	0.0000113
	0	0	0
	0	0	0.0000299
	0	0	0
	0	0	0
	0	0	0.000000371
	0	0	0
	0	0	0
	0	0	0
	0	0	0
	0	0	0
	0	0	0
	0	0	0
	0	0	0
	0	0	0
	0	0	0
	0	0	0
	0	0	0
	0	0	0.000000552
	0	0	0
	0	0	0
	0	0	0
	0	0	0

0	0	0
0	0	0
0	0	0.00000188
0	0	0.00000246
0	0	0
0	0	0
0	0	0
0	0.000000289	0
9.58E-08	0	0
0	0	0.00000104
0	0	0
0	0	0
0	0	0
0	0	0
0	0	0
0	0.00000029	0
0.000000115	0	0
0	0	0
0	0	0
0	0	0
0	0.000000111	0
0	0	0
0	0	2.31E-08
0	0	0
0	0	0
0	0	0
0	0.000000222	0
0	0	0.000000333
0	0	0
0	0	0
0	0	0.0000188
0	0	0
0	0	0
0	0	0
0	0	0
0	0	0
0	0	0
0	0	0
0	0	0
0	0	0
0	0	3.33E-08
0	0	0.00000142
0	0.00000196	0
0	0	0
0	0	0
0	0	0
0	0	0.00000193

0	0	0
0	0	0
0	0	0.00000163
0	0	8.76E-08
0	0	6.63E-08
0	0	0
0	0	0
0	0	0
0	0	0.000000286
0	0	0
0	0	0
0	0	0.000000217
0	0.00000013	0
0	0	0.000000602
0	0	0
0	0	0
0	0	0.000000546
0	0	0.0000143
0	0	0
0.000000211	0.000000203	0
0.000000604	0	0
0	0	0.000000142
0	0	0
0	0	0
0	0	0
0	0	0
0	0	5.54E-08
0	0	0.00000764
0	0	0.00000035
0	0	0
0	0	0
0	0	0
0	0	0
0	0.00000166	0
0	0	0
0	0	0.000000218
0	0.000000197	0
0	0	0
0.000000223	0.00000013	0
0	0	4.88E-08
0	0	0
0	0	0
0	0	0.0000186
0	0	3.83E-08
0	0	0
0	0	0
0	0	0

	0	0	0
	0	0	0
	0	0	0
	0	0	0
	0	0	0
	0	0	0
	0	0	0
	0	0	0
	0	0	0.000000404
	0	0	0
	0	0	0
	0	0	0
	0	0	0
	0	0	0
	0	0	0
	0	0	0
	0	0	0
	0	0	0
	0	0	0.00000201
	0	0	0.00000248
	0	0	0.00000513
	0	0	0
	0	0	0.00000228
	0	0	0
	0	0	0
	0	0	0.00000187
	0	0	0
	0	0	0
	0	0	0
	0	0	0
	0	0	0
	0	0	0
	0	0	6.59E-08
	0	0	0.0000013
	0	0	0
	0	0	0
	0	0	0
	0	0	0
	0	0	0
	0	0	0.00000238
	0	0	0
	0	0	0
	0	0	0.00000156
	0	0	0
	0	0	0.0000182
	0	0	8.04E-08
	0	0	3.91E-08

	0	0	0
	0	0	0.00000335
	0	0.000000151	0
	0	0	0.000000566
	0	0	0
	0	0	0
	0	0	0.000000858
	0	0	7.83E-08
	0	0	0.0000188
	0	0	0
	0	0	0
	0	0	0
	0	0	0.000000203
	0	0	0
	0	0	0
	0	0	0
	0	0	0
	0	0	0.000000266
	0	0	0
	0	0	0
	0	0	0
	0	0	0
	0	0	0
	0	0	0
	0	0	0.00000474
	0	0	0
	0	0	0
	0	0	0
	8.08E-08	0	0
	0	0	0.000000267
	0	0	0.000000304
	0	0	0
	0	0	0
	0	0	0.0000319
	0	0	0
	0	0	0
	0	0	0.0000006
	0	0	0
	0	0	0.000000875
	0	0	0
	0	6.38E-08	0
	0	0	0.0000015
	0	0	0
	0	0	0
	0	0	0
	0	0	0
	0	0	0.000000137

0	0	0
0	0	0
0	8.91E-08	0
0	0	0
0	0	0
0	0	0.000000332
0	0	0
0	0.000000076	0
0	0	0.000000164
0	0	0
0	0	0
0.00000163	0.0000042	0
0	0	0
0.000000657	0.000000668	0
0	0	0
0	0	0
0	0	0
0	0	0.000000123
0	0	0.000000408
0	0	0
0	0	0
0	0	0
0	0	0
0	0	0
0	0	0
0	0	0.00000257
0	0	0.00000511
0	0	0
0	0	0
0	0	0
0	0	0
0	0	0
0	0	0
0	0	0
0	0.000000293	0
0	0	0.000000058
0	0	0.000000058
0	0	0
0	0	0.000000755
0	0	0
0	8.37E-08	0
0	0	0
0	8.37E-08	0
8.18E-08	0.00000548	0
0.000000112	0	0
0	0	0
7.41E-08	0	0

0.000000339	0	0
0.00000013	0	0
0	0	0
0	0	0
0	0	0
0	0	0
0	0	0
0	0	0
0.000000155	0	0
0.000000357	0	0
0.000000842	0.00000287	0
0	0	0
0	0	0
0	0	0
0	0.000000108	0
0	0	0
0	0	0
0	0	0.0000189
0	0	0
0	0	0
0	0	0
0	0	0
0	0	0
0	0	0
0	0	0
0	0	4.52E-08
0	0	0.000000079
0	0	0.0000357
0.000000312	0.000000119	0
0.000000918	8.26E-08	0
0.000000136	0	0
0	0	0
0	0	0
0	0	0.000000503
0	0	0.000000044
0	0	0
0	0	0
0	0	0.000000802
0	0	0.00000333
0	0	0
0	0	0
0	0	0
0	0	7.66E-08
0	0	0
0	0	0.00000857
0	0	0
0	0	0.0000244

0	0	0.00000116
0	0	0
0	0	0
0	0	0
0	0	0
0	0	8.86E-08
0	0	0.000000501
0	0	0
0	0	0
0	0	0
0	0	0
0	0	0
0	0	0
0	0	0.00000246
0	0	0.00000252
0	0	0.00000543
0	0	0
0	0	0
0	0	0
0	0	0
0	0.000000346	0
0	0	0
0	0	0
0	0	0.000000621
0	0	0
0	0	0
0	0	0
0	0	0
0	0	0
0	0	0.00000276
0	0	0
0	0	0
0	0	0
0.00000246	0.00000301	0
0	0	0
0	0	0.000000115
0	0.000000128	0
0	0	3.67E-08
0.000000394	0	0
0.00000092	9.15E-08	0
0	0	0
0	0	0.00000013
0	0	0
0	0	0.00000125
0	0	0.000000766
0	0	0
0	0	0

0	0	0
0	0	0.000000378
0	0	0.00000239
0	0	0
0	0	0
0	0	0.00000431
0	0.000000158	0
0	0	0.0000269
0	0	0
0	0	0
0	0	0
0	0	4.47E-08
0	0	0
0	0	0
0	0	0
0	0.000000291	0
0	0	0
0	0	0.00000121
0	0	0
0	0	0
0	0.000000124	0
0	0	0
0	0	0
0	0	0
0	0	0
0	0	0
0	0	0.000000078
0	0	0
0	0	0.000000386
0	0	0.00000048
0	0	0
0	0	0
0	0	0
0	0	0
0	0	0
0	0	0
0	0	0.00000184
0	0	0
0	0	0
0	0.00000013	0
0	0	0.000018
0	0	0
0	0	0
0	0	0.000000302
0	0	0
0	0	0
0.00000634	0.000000585	0

0	0	0
0	0	0
0	0	0
0	0	0
0	0	0
0	0	0.00000888
0	0	0
0	0	0
0	0	0.000000157
0	0.000000179	0
0	0	0.0000224
0.000000124	0	0
0.00000244	0.0000157	0
0	0	0
0	0	0
0	0	0
0	0	0
0	0	0
0	0	0
0	0	0
0	0	0
0	0	0
0	0	0.000000118
0	0	0
0	0	0
0	0	0.00000145
0	0	0
0	0	0
0	0	0
0	0	0.0000113
0.000000118	0	0
0	0	0.00000123
0	0	0
0	0	0
0	0	0
0	0	0
0	0	0
0	0	0
0	0	0
0	0	0
0.00000609	0.000000538	0
0	0	0
0	0.0000016	0
0	0	0.0000102
0	0	0
0	0	0
0	0	0

0	0	5.51E-08
0	8.41E-08	0
0.0000036	0	0
0.000000134	0	0
0	0	0
0	0	0
0	0	7.74E-08
0	0	0
0	0	0
0	0	0.0000235
0	0	0
0	0	0.00000117
0	0	9.42E-08
0	0	0
0	0	0
0	0	0
0	0	0
0	0	0
0	0	0
0	0	0
0	0	0
0	0	0
0	0	0
0	0	0
0	0	0
0	0	0.00000232
0	0	0
0	0	0.00000284
0	0	0
0	0	0
0	0	0
0	0	0.000000465
0	0	0.000000069
0	0	0
0	0	0.0000234
0	0	0.000000368
0	0	0
0	0	0
0	0	0
0	0	0
0	0	0
0	0	5.21E-08
0	0	0
0	0	0
0	0	0
0	0	0
0	0	0.0000015

	0	0	0
	0	0	0
	0	0	0
	0	0	0
	0	0	0.000000802
	0	0	0
	0	0	0.0000081
	0	0	0.00000167
	0	0	0
	0	0	0.00000219
	0	0	0
	0	0	0
	0	0	0.00000286
	0	0	7.72E-08
	0	0	7.54E-08
	0	0	0
	0	0	0
	0	0	0.0000193
	0	0	0.000000184
	0	0	2.05E-08
	0	0	0
	0	0	0
	0	0.00000029	0
	0	0	0.000000664
	0	0	0
	0	0	0.000000646
	0	0	8.61E-08
	0	0	0.000032
	0	0	0.000000227
	0	0	0
	0	0	0.000000925
	0	0	0
	0	0	0.00000018
	0	0	0.00000108
	0	0	0
	0	0	0
	0	0	0
	0	0	0
	0	0	0
	0	0	0
	0	0	0.000000293
	0	0	0.00000028
	0	0	0
	0	0	0
	0	0	0
	0	0	0
	0	0	0

0	0	0
0	0	0
0	0	0
0	0	0
0	0	5.65E-08
0	0	0.00000499
0	0	0
0	0	0
0	0	0
0	0	0
0	0	0
0.0000732	0.0000283	0.0000498
0	0	0
0.000000188	0	6.45E-08
0.0000553	0.000440013	0.000177523
0.00000104	0.00000197	0.00000258
0.000604415	0.000727168	0.000577921
0	0.000000692	0
0.000000352	0.000000759	0.00000032
0	0	0
0.000000438	0.00000029	0
0.000000281	0.000000505	0.000000527
0.0000166	0.000000665	0
0.000398264	0.000500626	0.000363885
0.000000166	0.000000232	0
0.000000237	0.000000507	0.000000269
0.000000121	0	4.39E-08
0.000000121	0	4.39E-08
0.000201944	0.000153824	0.000115312
0.000000186	0	6.76E-08
0.000000186	0	6.76E-08
0	0	0
0	0.00000763	0.00000511
0.000000262	0.000000751	0.000000308
0.000245528	0.000242168	0.00022025
0	0	0
0	0	0
0.00000205	0.0000196	0.0000557
0	0	0
0.000000301	0.000000169	0
0	0	0
0.000285082	0.000138357	0.000174502
7.02E-08	0.000000491	0
0	0	0
0	0	0
0.0000431	0.0000419	0.0000665
0.0000772	0.0000141	0.0000437

0	0	0.000000413
0	0	0
0.00000022	0.000000213	0.000000301
0.000000103	0	4.11E-08
0.000000186	0	7.53E-08
0	0	0
0	0	0.000000324
0.000171635	0.00012039	0.000200347
0	0	0
0	0	0
0.0000903	0.00024661	0.000119363
0.0000878	0.000222478	0.000302148
0	0	0.00000212
0.00000187	0	0.000000208
0.0000901	0.000239598	0.000139403
0.000000988	0.000000566	0.000000753
0	0.000000362	0.000000161
0	0	0.0000019
0.000224423	0.0000683	0.000117553
0	0	0
0	0.000247479	0.00011448
0	0.000000462	0.000000216
0	0.000000462	0.000000216
0.000169282	0.000375634	0.000327445
0.000301917	0.000179237	0.000359728
0	0	0
0	0	0
0.00000182	0.00000746	0.00000231
0.000000584	0.0000025	0.0000027
0.000411261	0.000412773	0.000422601
0	0	0
0.000000106	0	5.31E-08
0	0	0
0	0	0
0	0	0
0.000000193	0.000000122	0
0	0	0
0	0.000000177	0
0.000174504	0.0000836	0.000261242
0	0	0
0.00000289	0.00000297	0.00000299
0	0.000000331	0.000000181
0.000000166	0.000000297	0
0	0	0
0.0000699	0.0000892	0.000129282
0	0	0
0	0.000000221	0.000000125

0	0.000000214	0.000000121
0.000380045	0.000307957	0.000488439
0.00055743	0.000424431	0.000689746
0	0	0
0.000000117	0	6.75E-08
0	0.000000291	0.000000168
0	0	0
0.000106943	0.000145922	0.000159558
0	0.000000212	0
0.000000134	0	7.89E-08
0.000395308	0.000328186	0.000518261
0	0	0
0.00000175	0.000000281	0.00000191
0.000000682	0.00000147	0.00000206
0	0	0
0	0	0
0	0	0
0	0	0
0.000000557	0.0000137	0.00000377
0.00000013	0	8.12E-08
0	0.00000023	0.000000145
0	0	0
0	0	0
0	0.00000141	0.00000164
0.000000129	0	8.66E-08
0.000000778	0.00000435	0.0000133
0	0	0
0	0	0
0.000182858	0.0000854	0.00024425
0	0	0
0	0	0
0	0	0
0	0	0
0	0	0
0.000000752	0.00000014	0.000000268
0.000000215	0	0
0	0	0
0.000000223	0	0.000000168
0	0	0
0.0000913	0.000203687	0.00019815
0.00047939	0.000343185	0.000689746
0.00047939	0.000343185	0.000689746
0	0	0
0	0	0.000000159
0	0	0.000000159
0.000142326	0.000100771	0.00017491
0	0	0

0	0	0
0	0	0
0	0	0
0	0	0
0	0	0
7.76E-08	0	0.000000064
0	0	0
0	0	0
0	0	0.000000105
0	0	0
0	0	0.00000198
0.0000228	0.0000236	0.0000145
0	0	0
0	0	0
0	0	0
0	0	0
0.000107218	0.0000514	0.000135831
0	0	0.00000284
0.000000242	0	0.0000013
0.0000571	0.000029	0.000068
0.000180269	0.0000974	0.000267584
0	0	0.00000239
0	0	0
0	0	0.00000706
0	0	0
0	0	0
0	0	0
0	0	0
0.0000991	0.000167714	0.000273696
0	0	0
0.000000525	0.0000137	0.00000284
0	0	0
0.17682	0.233487	0.439063
0	0	0
0	0	0.000000259
0.000221336	0.0000294	0.0000169
0	0	0.00000247
0.00024594	0.000176052	0.00025656
0	0	0
0	0	0.000000224
0	0	0.00000261
0.000823082	0.00140928	0.00395834
0.00000584	0.0000145	0.0000132
0	0	0.00000301
0.000000175	0	0
0.000000087	0	0.0000023
0.00010942	0.000236844	0.000253638

0.000251842	0.000115107	0.000374015
0	0	0.00000304
0.000000865	0.0000364	0.00002
0	0	0
0	0	0.00000291
0	0	0.00000396
0	0	0
0.00000115	0.0000025	0.00000139
0	0	0
0	0	0.00000307
0.000266831	0.00018886	0.000322301
0	0	0.00000305
0	0	0.000000204
0.0000352	0.000404391	0.000475569
0	0	0.00000288
0	0	0.000003
0	0	0
0.000246489	0.000388569	0.00055814
0.00000542	0.0000273	0.0000292
0.00000139	0.000000495	0.00000295
0	0	0.00000354
0	0	0.000000107
0.000000387	0	0.00000302
0	0	0.0000032
0	0	0.00000402
0.000000276	0.000000295	0.000000627
0.000241606	0.000167372	0.000291151
3.46E-08	0	4.32E-08
0	0	0.00000396
0	0	0
0.000274216	0.00000113	0.00000804
0.000000822	0.0000022	0.00000267
0	0	0.0000027
0	0	0.00000211
0	0.000000146	0.000000188
0	0	0
0	0	0
0.000175965	0.0000953	0.000207526
0	0	0.00000361
0	0	0.00000325
0	0	0.00000325
0	0	0.00000297
0	0	0.00000245
0	0	0.0000022
0.000000285	0	0
0.00018091	0.000283454	0.000672844
0.00000248	0.0000286	0.000104725

0.000000225	0	0
0	0	0.00000343
0	0	0.00000316
0.000000518	0.00000302	0.00000089
0	0	0.00000248
0	0	0.00000248
0	0	0.00000251
0	0	0.00000288
0	0	0.000000108
0	0	0.000000126
0	0	0.00000345
0	0	0
0	0	0
0	0	0.00000268
0	0	0.00000334
0	0	0.00000322
0	0	0
0	0	0.00000338
0	0	0
0.00000139	0.000000514	0.0000031
0	0	0
0.00014842	0.0000724	0.000357363
0	0	0.00000329
0.000000795	0.00000495	0.00000241
0.00027037	0.00000136	0.00000953
0	0	0.00000339
0	0	0.00000298
0	0	0.000000218
0	0	0.00000363
0	0	0.00000342
0	0	0.00000225
0	0	0.00000342
0	0	0.00000341
0	0	0.00000374
0.000595089	0.0894854	0.0159819
0	0	0.00000232
0	0	0.000000163
0	0	0.00000332
0	0	0.00000369
0	0	0.000000329
0	0.000000865	0.00000229
0.00000154	0.0000332	0.0000311
0	0	0.00000295
0	0	0
0	0	0
0	0.000000879	0.0000035
0	0	0.00000351

0	0	0.00000352
0	0	0.00000355
0	0	0.00000334
0	0	0.000000321
0	0	0.000000267
0	0	0
0	0	0
0.00000039	0	0
0.000000741	0.00000046	0.00000357
0.000000815	0.00000662	0.0000077
0	0	0.00000411
0	0	0.000000209
0.000144703	0.0000991	0.000186233
0	0	0.000000324
0.00000493	0.0000235	0.0000287
0.000103054	0.000106396	0.00042168
0.000103021	0.00010635	0.00042168
0	0	0.000000443
0.000000726	8.93E-08	0
0	0	0.000000175
0.000000375	0	0
0.0000468	0.000111454	0.000164791
0.00000116	0.00000497	0.00000984
0	0	0.000000303
0.000000958	0.00000127	0.00000329
0	0	0.000000787
0.00016548	0.000427412	0.000582472
0	0	0.000000594
0.00000143	0.000034	0.000036
0	0	0.000000248
0.000105745	0.0000211	0.000105008
0	0	0.000000368
0.0000016	0.0000134	0.0000179
0.000113039	0.000141827	0.000299865
0	0.000011	0.0000352
0.000000589	0.0000127	0.000022
0	0	0.00369438
0	0	0
0.000000724	0.0000013	0.00000218
0.0000712	0.000295679	0.000343255
0	0	0.000000638
0	0	0
0.0000572	0.0000188	0.00006
0	0	0.000472554
0	0	0.000000387
0	0	0.00000124
0	0	0.00000112

0	0	0.00000289
0	0	0.00000332
0	0	0.0000042
0	0	0.000000946
0.0000014	0.000000941	0.00000963
0.000000343	0	0
0	0	0.000000985
0	0	0.000000985
0.0000568	0.0000205	0.000129055
0	0	0.000000104
0	0	0.00000453
0	0	0.00000453
0	0	0
0.00000261	0.0000301	0.000145855
0	0	0.000000465
0	0	0.000000198
0.00000112	0.00000793	0.0000552
0.0000437	0.00000735	0.0000806
0.000156227	0.000427904	0.00061826
0	0	0
0	0	0.00000402
0.0000774	0.0000165	0.0000821
0.00000571	0.00000861	0.0000284
0	0	0.00621788
0.00000405	0.0000159	0.0000221
0	0	0
0.00011369	0.000415978	0.000573787
0	0.000000369	0.00000157
0	0	0.000000836
0	0.000005	0.0000184
0	0	0.000000378
0	0	0.000000449
0	0	0.000000121
0.0000918	0.0000206	0.000146279
0.00000577	0.00003	0.0000638
0	0	0.00000013
0.000000553	0.0000126	0.0000248
0	0	9.67E-08
0.00000426	0.0000156	0.0000253
0	0	0.000000489
0	0	0.000000489
0	0	0.00000375
0	0	0.00732317
0	0	0.00000222
0	0	0.000467781
0	0	0.00000442
0	0	0.00000323

0	0	0.000000146
0	0	0
0.00000576	0.00000996	0.0000174
0	0	0.000000294
0	0	0.00000497
0	0	0.00000423
0	0	0.00000417
0	0	0.00000417
0.000238701	0.000240559	0.000549389
0	0	0.00000384
0.000130206	0.000453151	0.000605932
0	0	0.00000415
0	0	0.000000209
0	0	0.00000015
0.0000614	0.00000878	0.0000478
0.00000168	0.0000211	0.0000722
0	0	0.000000417
0.00000076	0	0.00000342
0	0	0
0	0	0.00000143
0.0000849	0.0000146	0.000135621
0.00000149	0.0000235	0.000029
0	0	0.0000266
0	0	0.00000062
0	0	0.000000441
0.00000584	0.00000627	0.0000131
0	0	0.00000437
0	0	0.00000319
0	0	0.00000368
0	0	0
0	0.00000166	0.00000509
0	0	0
0	0	0.000000467
0	0	0.00000016
0.000000429	0.000000735	0.00000215
0	0	0.000000519
0	0	0
0.000000653	0.000000114	0.00000104
0.000000501	0	0.00000216
0	0	0
0.000000307	0.000000287	0.00000194
0	0	0.000000682
0.0000936	0.0000153	0.000150418
0	0	0
0	0	0.000000483
0.0000753	0.0000128	0.000117332
0	0	0.000000368

0	0	0
0	0	0
0	0	0.000000221
0.00000494	0.0000334	0.000180219
0	0	0.000000644
0	0	0
0	0	0
0	0	0
0	0	0
0	0	0
0	0	0
0	0	0
0.0186954	0.035811	0.13257
0	0	0.00000107
0.000000867	0.00000884	0.0000146
0.00000112	0.0000095	0.000017
0	0	0
0	0	0
0	0	0.000000501
0	0	0
0	0	0.00000149
0	0	0.00000149
0.000000301	0.000000226	0.00000207
0	0	0
0.000000352	0	0
0	0	0
0.000000549	0.000000177	0.00000105
0	0	0.000000456
0	0	0
0	0	0
0	0	0
0	0	0.00000457
0	0	0
0	0	0
0	0	0
0.00000215	0.0000257	0.0000226
0.000000385	0.000016	0.0000249
0	0	0.000000415
0	0	0
0	0	0
0	0	0.00000018
0.000223173	0.000215386	0.000458701
0.00000027	0	0.00000116
8.16E-08	0.000000118	0
0	0	0
0	0	0
0	0	0

0.0000645	0.00000422	0.000124006
0.00000101	0.00000417	0.00000155
0.00000583	0.0000106	0.000026
0	0	0
0.000000195	0.00000021	0.00000423
0.0000037	0.0000327	0.000209617
0.0000571	0.0000104	0.000108135
0	0	0
0	0	0
0	0	0
0	0.00000886	0.0000193
0.00000437	0.0000164	0.0000542
0	0	0.000000694
0	0	0
0.00000034	0.00000036	0.00000506
8.74E-08	0.00000508	0.00000614
0.00000428	0.0000225	0.0000475
0.00000015	0.00000036	0.00000491
0.0000563	0.000012	0.000118291
0.00000115	0.0000115	0.0000564
0.000000277	0.0000004	0.00000588
0	0	0
0.000000181	0.00000021	0.00000387
0.00000151	0.0000178	0.0000268
0.000000233	0.000000227	0.00000416
0	0	0
0.00000211	0.0000211	0.0000659
0.000000489	0.0000104	0.0000107
0	0	0.000000367
0.00000149	0.0000175	0.0000277
0	0	0
0.00000238	0.0000476	0.0000586
0.000000957	0.00000172	0.00000934
0.00000176	0.0000095	0.0000461
0	0	0.00000761
0	0	0
0	0	0.00187644
0.000000277	0.000000311	0.0000057
0.000000261	0.000000355	0.00000601
0.000000508	0.000000226	0.00000221
0.000000192	0.000000219	0.000004
0.000000608	0.0000214	0.0000492
0	0	0.00000062
0.000000155	0.000000203	0.00000375
0.00000187	0.0000242	0.0000931
0	0	0.0000011
0.00000064	0.000000111	0.00000632

	0.0000435	0.0000399	0.000233143
	0.000022	0.0000248	0.0000785
	0.00000238	0.0000277	0.000138075
	0	0	0.00000156
	0.000000179	0.000000134	0.00000448
	0.000000818	0.0000123	0.0000564
	0.00000119	0.00000174	0.0000138
	0	0	0.000003
	0.000000878	0.000000204	0.00000357
	0.00000743	0.0000514	0.000309441
	0	0	0.00000345
	0.00000174	0.0000147	0.0000597
	0	0	0
	0.00000127	0.00000433	0.000018
	0	0	0
	0	0	0
	0.0000625	0.0000369	0.000315977
	0	0	0
	0	0	0.00000153
	0.000000185	0.000000211	0.00000531
	0	0	0
	0	0	0.00000127
	0	0	0
	0.000000471	0.0000103	0.0000362
	0	0	0
	0	0	0.00000111
	0	0	0.00000162
	0.000000797	0.00000616	0.0000162
	0.000000707	0.000000283	0.0000025
	0	0	0
	0.000000221	0.000165875	0.0000295
	0.000000621	0	0.00000834
	0.000000227	0	0.00000158
	0.00000293	0.0000249	0.000126199
	0	0	0.00000138
	0	0	0
	0.000000111	0.00000017	0.0000056
	0.000000111	0.00000017	0.0000056
	0.00000103	0.0000155	0.0000452
	0	0	0.00000173
	0	0	0.00000282
	0.000000522	0.0000062	0.0000261
	0	0	0
	0.000000234	0.000165435	0.0000304
	0.000000237	0	0.00000159
	0.00000436	0.0000253	0.000143615
	0	0	0.00000146

9.58E-08	5.84E-08	0.00000234
0.000000278	0.000189212	0.0000336
0	0	0.00000141
0	0	0
0	0	0
0.000000621	0.00000373	0.0000114
0.000000249	0.000172099	0.0000305
0.000000258	0.000174917	0.0000308
0.00000051	0.0000128	0.0000322
0.000000252	0.000168475	0.00003
0.000000288	0.000145996	0.0000265
0	0	0.00000146
0.00000036	0.0000154	0.0000354
0.000000129	0.000000189	0.00000546
0.000000151	0.000000712	0.00000625
0.000000275	0	0.00000167
0.000000196	0.000181887	0.0000326
0	0	0.00000472
0	0	0.00668026
0.000000357	0.000000361	0.00000293
0.000000357	0.000000361	0.00000293
0	0	0
0	0	0
0	0	0
0.000000235	0.000153393	0.0000275
0.000000235	0.000153393	0.0000275
0	0	0.00000405
0	0	0
0.000000255	0.000179378	0.0000306
0.000000525	0.0000137	0.0000367
0	0	0
0	0	0.00000398
0	0	0.00000398
0	0	0.0000044
2.72E-08	6.17E-08	0.00000102
0.000000307	0.000177131	0.00003
0	0	0.00000401
0.00000158	0.0000228	0.0000877
0	0	0
0	0	0
0.000000604	0.000000651	0.00000386
0	0	0.00000466
0	0	0.00000563
0	0	0
0	0	0.0000039
0.000000821	0.0000129	0.000036
0.0000631	0.0000771	0.000257003

0	0	0.00000477
0	0	0.0179281
0.000000214	0.000223775	0.0000404
0	0	0.00000507
0	0	0.00000507
0	0	0
0	0	0.00000571
0.000000227	0.000000417	0.0000032
4.62E-08	0.000000101	0.0000015
4.62E-08	0.000000101	0.0000015
0	0	0.00000661
0.00000019	0.000143964	0.0000244
0	0.000000313	0.00000451
0	0	0
0.000000259	0.000000395	0.00000305
0	0	0.00000425
0	0	0.00000143
0	0	0.00000468
0.00000189	0.00001	0.0000517
0	0	0.0000215
0.00000125	0.0000124	0.0000358
0	0	0
0	0	0.0000225
0	0	0
0	0	0
0.000000477	0.000000056	0.0000026
0.00000168	0.00000111	0.0000186
0	0	0.00000411
0.00000241	0.0000293	0.000180573
0.00000372	0.0000233	0.000162358
0.00000139	0.0000403	0.000110085
0.0000605	0.0000629	0.000771118
0	0	0.00000444
0	0	0.00000146
0.000000101	0	0.00000159
0	0	0
0	0	0.00000443
0	0	0.00000462
0	0	0.00000383
0	0	0
9.42E-08	0	0.00000163
9.42E-08	0	0.00000163
0	0	0
0	0	0.00000562
0	0	0.00000435
0	0	0.0000225
0.00000262	0.0000349	0.000224558

0.00000243	0.0000168	0.0000649
0	0.00000015	0.00000024
0.00000224	0.000015	0.000099
0	0	0.00000467
0	0	0
0	0	0
0.000000226	0.000000312	0.00000226
0	0	0
0	0	0.000028
0.000000202	0	0.00000136
0.00000256	0.0000307	0.000206441
0	0	0
0	0	0.0000246
0	0	0
0	0	0.000026
0	0	0.0000245
0	0	0.00000484
0.0000206	0.0000201	0.000219509
0	0	0.0000323
0	0	0
0	0	0
0.00000837	0.0000157	0.000219509
0.00000837	0.0000157	0.000219509
0	0	0
0.00000303	0.0000167	0.000147742
0	0	0
0	0	0
0.000000325	0.000000398	0.00000239
0	0	0.0000269
0.000000284	0.000000356	0.00000265
0	0	0.0000241
0	0	0.00000582
0	0.000000123	0.00000443
0	0	0.0000255
0.00000166	0.0000134	0.0000959
0.000000116	0	0.000000916
0	0	0.0000224
0	0	0.0000257
0	0	0.00000243
0.000000267	0.000000349	0.00000265
0	0	0.0000241
0.00000287	0.00000258	0.000023
0.00000135	0.0000122	0.0000952
0.00000133	0.0000122	0.0000952
0.000000142	0.0000033	0.0000169
0.000179855	0.000251062	0.0019316
0	0	0.0000243

0	0	0.0000243
0.000000355	0.000000348	0.00000387
0.000000319	0.000000349	0.00000364
0.000000963	0.0000142	0.0000334
0.000000284	0.000000392	0.00000311
0.000000284	0.000000392	0.00000311
0	0	0
0	0	0.0000274
0.000000123	0	0.00000114
0	0	0.0000255
0	0	0.000022
0	0	0.0000013
0.000000101	0	0.000000837
0.000000101	0	0.000000837
0.000000455	0.000000225	0.00000287
0.000159237	0	0.00122486
0	0	0.0000257
0.000000166	0	0.00000125
0	0.00000279	0.00000208
0	0	0.00000122
0	0	0.00000122
0.000000298	0.000000291	0.00000258
0.00000041	0.000000444	0.00000388
0	0	0.00000446
0.000000241	0	0.00000152
0.000000212	0	0.00000154
0.000000199	0.000000272	0.00000234
0.000000253	0	0.00000165
0.00000134	0.000000863	0.00000958
0.000000363	0.000000351	0.00000307
0.00000334	0.0000322	0.000206985
0.00000334	0.0000322	0.000206985
7.59E-08	0	0.00000128
0.00000243	0.00003	0.000201565
0	0	0.00000443
0	8.43E-08	0.00000428
0	0	0.0000191
0.000000287	0.000000282	0.00000243
0.000000104	0.000000158	0.00000114
0.000000325	0.000000352	0.00000363
0.000000235	0	0.00000157
0.000000165	0	0.00000126
0.00000039	0.000000301	0.00000261
0.00000021	0	0.00000137
0	0	0.00000408
0	0	0.0000235
0.000000226	0	0.00000152

0.000000248	0	0.00000132
0	0	0.0000206
0.00000218	0.0000154	0.000100875
0	8.92E-08	0.0000055
0.00000118	0.0000316	0.000172771
0	0	0.00000144
0.000000332	0.000000411	0.00000266
0.000000276	0.000000313	0.00000312
0	0	0.0784451
0.00000281	0.0000121	0.000190159
0.000000174	0.00000031	0.00000235
0	6.27E-08	0.00000453
0.000000818	0.00000624	0.0000453
0.000000222	0	0.0000012
0.000000419	0.00000102	0.0000525
0.000000224	0.00000018	0.00000212
0.00000021	0.000000167	0.000002
0.0000188	0.0000046	0.000115493
0.00000262	0.0000221	0.000227645
0.000000204	0.000000163	0.00000197
0.000000192	0.000000665	0.0000125
0	0.00000198	0.0000479
0	0	0.000000714
0.000000149	0.000000518	0.00000986
0	0	0
0.00000281	0.0000112	0.000183472
0.000000198	0.0000003	0.00000295
0.00000209	0.0000215	0.000170648
0.000000238	0.000000274	0.00000252
0.000000505	0.00000189	0.0000176
3.51E-08	0	0.0000035
0.000000832	0.0000174	0.000288448
0.000000201	0.000000236	0.00000281
0	0.000000291	0.0000331
0	0	0.0305147
0	0	0.0000077
0	0	0.00000477
0.000000213	0	0.00000205
0.00000128	0.0000207	0.000183684
0.00000662	0.00000437	0.000160146
0.000000224	0.000000197	0.00000198
0	0	0.000033
0.000000716	0.000000358	0.0000142
0	0	0.00000607
0	0	0.0000649
0	0	0.0000817
0.00000117	0.000018	0.000216972

0.00000138	0.0000199	0.000194513
0	0	0.0000701
0	0	0.0000826
0	0	0.0000669
0	0	0.0000861
0.00000123	0.0000204	0.000197537
0	0	0.0000542
0	0	0.0000758
0.000000168	0.000000286	0.00000281
0	0	0.0000424
0.00000015	0.000000253	0.0000022
0.000000344	0	0.00000432
0	0	0.0000401
0	0	0.00000386
0	0	0.0000854
0	0	0.0000608
0	0	0.0000675
0	0	0.0000781
9.32E-08	0	0.00000105
0	0	0.0000536
0	0	0.0000715
0	0	0.0000557
0	0	0
0	0	0.00000863
0	0	0.0000355
0	0	0.0000658
0	0	0.0000624
0	0	0.0000973
0.00000205	0	0.000160144
0	0	0.0000808
0	0	0.0000618
0	0	0.000114283
0	0	0.000114283
0	0	0.00008
0	0	0.0000698
0	0	0.0000812
0	0	0.0000608
0	0	0.0000608
0	0	0.0000643
0	0	0.00000755
0	0	0.0000561
0	0	0.0000552
0	0	0.0000436
0	0	0.0000434
0	0	0.0000434
0	0	0.0000812
0	0	0.0000512

0	0	0.0000748
0	0	0.0000456
0	0	0.00000792
0	0	0.000114283
0	0	0.00000738
0	0	0.0000511
0	0	0.0000488
0	0	0.00000834
0	0	0.0000841
0	0	0.0000841
0.000000166	0	0.00000145
0	0	0.00000723
0	0	0.00000723
0	0	0.0000316
0	0	0.0000316
0	0	0.0000339
0	0	0.0000454
0	0	0.0000395
0	0	0.0000454
0	0	0.0000486
0	0	0.0000418
0	0	0.000037
0	0	0.0000338
0	0	0.0000326
0	0	0.0000603
0	0	0.0000103
0	0	0.0000483
0	0	0.00000351
0	0	0.0000101
0	0	0.0000425
0	0	0
0	0	0.00000906
0	0	0.00000926
0	0	0.0000531
0	0	0.0000531
0	0	0.0111892
0	0	0.000054
0	0	0.00000874
0	0	0.0000353
0.000000275	0	0.00000466
0	0	0.00000891
0	0	0.0000353
0.000000896	0	0.000082
0	0	0.00000872
0	0	0.0000527
0	0	0.0000527
0.00000175	0.00000133	0.0000882

0	0	0.0000644
0	0	0.00000821
0.000000175	0.000000167	0.0000143
0	0	0
0	0	0.00000863
0	0	0.0000552
0	0	0.0000552
0	0	0.00000829
0	0	0.00000816
0	0	0.00000844
0.000000336	0	0.0000027
0	0	0.0000293
0.000000634	0.000000463	0.0000598
0	0	0.0000529
0	0	0.00000806
0	0	0.00000702
0	0	0.00000702
0	0	0.0000197
0.00000144	0.00000216	0.000133552
0	0	0.000043
0	0	0.0000223
0.000000551	0.00000037	0.0000282
0	0	0.0000478
0	0	0
0	0	0.0000297
0.000000223	0.000000285	0.0000266
0	0	0.0000774
0.00000182	0.00000158	0.000136704
0	0	0.0000225
0	0.0000167	0.0000508
0	0.0000129	0.0000407
0	0.00000502	0.0000169
0	0.00555284	0.0178875
0	0.00000537	0.0000181
0.000000314	0.000000492	0.0000535
0	0.0000145	0.0000447
0	0.00000558	0.0000188
0.000000709	0.000000473	0.000212258
8.74E-08	0.00000508	0.000104604
0.000000283	0.000000338	0.0000329
0	0.00000554	0.0000155
0	0.0000151	0.0000475
0	0.0000156	0.0000488
0	0.0000156	0.0000488
0.00000019	0.000000239	0.0000283
0.000000146	0.000000441	0.0000542
0.000000271	0.000000316	0.0000384

0	0	0.00000383
0.000000185	0	0.0000242
0.000000168	0.000000121	0.0000355

log10_vanderbiltRatio	log10_uncRatio	averageRatio
2.071209823	2.786766601	2.428988212
1.989078505	2.858640857	2.423859681
1.982061477	2.821268388	2.401664932
1.982061477	2.821268388	2.401664932
2.025233516	2.768201331	2.396717424
1.884764677	2.892626191	2.388695434
2.004157691	2.737938713	2.371048202
1.923165832	2.818288973	2.370727402
2.253885055	2.479384102	2.366634579
2.002961273	2.70329937	2.353130321
1.911072735	2.730766592	2.320919663
1.963707807	2.664318978	2.314013393
1.99406361	2.624145196	2.309104403
1.917121122	2.688177783	2.302649452
2.198576132	2.400125323	2.299350727
1.967101903	2.624553976	2.295827939
1.970315073	2.621072618	2.295693845
2.310027366	2.270257006	2.290142186
2.196029013	2.382791915	2.289410464
2.21178584	2.347347624	2.279566732
1.907173324	2.644099702	2.275636513
2.170159382	2.376245252	2.273202317
2.168096235	2.376218714	2.272157475
1.882450567	2.652130576	2.267290572
1.923927977	2.604127437	2.264027707
1.901705916	2.623773429	2.262739672
2.141096976	2.383274683	2.262185829
2.141096976	2.383274683	2.262185829
1.987283695	2.532992216	2.260137956
1.920563019	2.596084682	2.25832385
1.902150462	2.612691995	2.257421228
2.02701267	2.486246334	2.256629502
2.02701267	2.486246334	2.256629502
1.934841382	2.572634612	2.253737997
1.889802963	2.614596654	2.252199809
2.169940358	2.332315199	2.251127779
1.933311766	2.565796547	2.249554156
1.925007364	2.566058292	2.245532828
1.925007364	2.566058292	2.245532828
1.891626169	2.590338535	2.240982352
1.891626169	2.590338535	2.240982352
2.248984223	2.219475412	2.234229818
2.471385415	1.996620785	2.2340031
1.990079653	2.465981281	2.228030467
2.143618562	2.310677853	2.227148208
2.195688983	2.231013347	2.213351165

	2.214851733	2.203224237	2.209037985
	2.131573695	2.276454048	2.204013871
	2.161183678	2.219136769	2.190160223
	2.370745089	1.974830731	2.17278791
	1.881745131	2.459213547	2.170479339
	2.115483905	2.20952241	2.162503158
	2.245578517	2.067784169	2.156681343
	2.117200947	2.195562897	2.156381922
	2.295934802	2.014282276	2.155108539
	2.336081807	1.972633994	2.1543579
	2.266144229	2.027062073	2.146603151
	2.106130523	2.15537445	2.130752487
	2.106130523	2.15537445	2.130752487
	2.155964975	2.092776619	2.124370797
	2.039571736	2.196578548	2.118075142
	2.062585024	2.171488013	2.117036518
	2.023375955	2.209876946	2.116626451
	1.997738753	2.232763635	2.115251194
	2.090178394	2.138120256	2.114149325
	2.107416613	2.117723231	2.112569922
	1.893073526	2.317380197	2.105226862
	1.956555001	2.253022885	2.104788943
	1.956555001	2.253022885	2.104788943
	2.012776013	2.195362079	2.104069046
	2.094143151	2.113555585	2.103849368
	2.094143151	2.113555585	2.103849368
	1.996802676	2.207780767	2.102291722
	1.959268825	2.241801864	2.100535345
	2.045491205	2.153198203	2.099344704
	1.833739646	2.357086263	2.095412954
	1.957619286	2.231633895	2.094626591
	1.864952643	2.321701422	2.093327032
	2.10293604	2.075688039	2.089312039
	2.000947264	2.168576253	2.084761759
	1.949249073	2.217330035	2.083289554
	1.954411367	2.207399627	2.080905497
	2.015024816	2.145049301	2.080037058
	2.113908298	2.043585171	2.078746734
	1.85302956	2.300919948	2.076974754
	1.936526209	2.214532289	2.075529249
	1.855873484	2.290965464	2.073419474
	2.109940357	2.036447724	2.073194041
	1.907011378	2.239113838	2.073062608
	1.971532389	2.17339535	2.07246387
	1.999060336	2.14429406	2.071677198
	1.978252965	2.164001513	2.071127239
	1.978252965	2.164001513	2.071127239

	1.933078569	2.208489503	2.070784036
	1.937973223	2.180300732	2.059136978
	2.05194578	2.06508936	2.05851757
	2.046140265	2.06823051	2.057185387
	1.858781706	2.252589206	2.055685456
	1.846274178	2.261704521	2.05398935
	1.946282114	2.161165608	2.053723861
	1.946282114	2.161165608	2.053723861
	1.946282114	2.161165608	2.053723861
	1.957211671	2.148240179	2.052725925
	1.964227164	2.140669489	2.052448327
	1.882854909	2.220032431	2.05144367
	1.997264522	2.103579466	2.050421994
	1.983210882	2.117002254	2.050106568
	1.941951558	2.154701344	2.048326451
	1.849190029	2.243778281	2.046484155
	2.197448846	1.894734934	2.04609189
	1.924642742	2.167062496	2.045852619
	1.918695764	2.172251261	2.045473513
	1.891823783	2.196655986	2.044239885
	1.91668579	2.171297229	2.04399151
	2.194980958	1.890264896	2.042622927
	1.990811722	2.089520126	2.040165924
	1.911558892	2.162015245	2.036787069
	2.002351435	2.067505321	2.034928378
	2.002351435	2.067505321	2.034928378
	1.866085822	2.201939749	2.034012786
	1.866085822	2.201939749	2.034012786
	1.866085822	2.201939749	2.034012786
	1.866085822	2.201939749	2.034012786
	1.916646751	2.151148318	2.033897535
	1.921779944	2.143752968	2.032766456
	1.975742482	2.088204581	2.031973531
	1.912593198	2.1493654	2.030979299
	1.761358477	2.295357229	2.028357853
	1.761358477	2.295357229	2.028357853
	1.890354429	2.164118023	2.027236226
	1.792719182	2.261341212	2.027030197
	1.945276494	2.106376333	2.025826414
	1.935788253	2.112516585	2.024152419
	1.927966884	2.114765332	2.021366108
	1.98633489	2.053662563	2.019998727
	1.890506773	2.146694674	2.018600723
	1.904558264	2.129016885	2.016787574
	2.1584208	1.868141685	2.013281243
	1.949962846	2.076581077	2.013271961
	1.891151849	2.128368333	2.009760091

	1.891151849	2.128368333	2.009760091
	1.815775143	2.203010451	2.009392797
	2.049759876	1.968491386	2.009125631
	2.000052065	2.01799357	2.009022817
	1.8965668	2.121456359	2.00901158
	1.952611488	2.062353055	2.007482272
	1.922155405	2.08476261	2.003459008
	1.956763128	2.049373106	2.003068117
	1.885415587	2.114256514	1.99983605
	1.833260892	2.164631895	1.998946394
	1.877151876	2.116257404	1.99670464
	1.958163199	2.035181273	1.996672236
	1.860226121	2.13163721	1.995931665
	1.99219738	1.999546256	1.995871818
	1.849159834	2.141535517	1.995347676
	1.897737565	2.092025826	1.994881696
	1.810652582	2.178688863	1.994670723
	1.992016104	1.99507656	1.993546332
	1.992016104	1.99507656	1.993546332
	1.992016104	1.99507656	1.993546332
	1.992016104	1.99507656	1.993546332
	1.849129841	2.135775173	1.992452507
	1.89383772	2.090858819	1.992348269
	1.960536256	2.023015134	1.991775695
	2.006650182	1.975521154	1.991085668
	1.876826197	2.105277125	1.991051661
	1.913479467	2.063162949	1.988321208
	1.856909354	2.117646518	1.987277936
	1.864962735	2.107458567	1.986210651
	2.143521156	1.828720067	1.986120612
	1.891956917	2.077321321	1.984639119
	1.939652822	2.029331475	1.984492148
	2.047600741	1.920332688	1.983966714
	1.898350971	2.062375134	1.980363052
	1.863050208	2.094083956	1.978567082
	1.895700654	2.057247783	1.976474219
	1.77071072	2.172998625	1.971854673
	1.7392463	2.203446144	1.971346222
	1.915768047	2.023272086	1.969520066
	1.907533387	2.019748103	1.963640745
	1.802255727	2.124819242	1.963537485
	2.002331904	1.924216145	1.963274024
	1.985807117	1.93900996	1.962408539
	1.757763668	2.166605731	1.962184699
	2.191933386	1.732253941	1.962093663
	1.987542122	1.935098973	1.961320547
	1.945738766	1.974534365	1.960136566

	1.941553798	1.976682318	1.959118058
	1.983134216	1.933321836	1.958228026
	1.839397462	2.076436328	1.957916895
	1.832931274	2.081885537	1.957408405
	1.938718563	1.97421636	1.956467461
	1.986538728	1.920126582	1.953332655
	1.792793263	2.10887406	1.950833661
	1.878898568	2.021916396	1.950407482
	1.976557014	1.922443872	1.949500443
	1.890040121	1.997806408	1.943923265
	1.820960993	2.061358468	1.941159731
	1.820960993	2.06132957	1.941145281
	1.829360854	2.052117929	1.940739391
	1.800779442	2.080539444	1.940659443
	1.936197127	1.944166797	1.940181962
	1.810263178	2.069443469	1.939853323
	1.899584022	1.97800947	1.938796746
	1.916161515	1.961303469	1.938732492
	1.94501404	1.929726048	1.937370044
	1.911012158	1.957355449	1.934183804
	1.847699527	2.010384042	1.929041785
	1.867101816	1.990282838	1.928692327
	1.86430072	1.989700287	1.927000503
	1.885940794	1.966825727	1.92638326
	1.976674078	1.874576616	1.925625347
	1.837699462	2.010041315	1.923870389
	1.502386072	2.342726218	1.922556145
	1.854371737	1.988475906	1.921423822
	1.56003503	2.280946655	1.920490843
	1.56003503	2.280946655	1.920490843
	1.834971391	2.003721023	1.919346207
	1.834971391	2.003721023	1.919346207
	1.905202266	1.932799142	1.919000704
	1.790584278	2.044757626	1.917670952
	1.790584278	2.044757626	1.917670952
	2.177709095	1.648898465	1.91330378
	2.272097076	1.553876397	1.912986736
	1.810765865	2.014689275	1.91272757
	1.736761812	2.087742604	1.912252208
	1.844841771	1.979430122	1.912135946
	1.132530557	2.676830139	1.904680348
	2.021832614	1.787487066	1.90465984
	1.720042416	2.086933742	1.903488079
	1.809790965	1.991502317	1.900646641
	1.874047356	1.91929078	1.896669068
	1.817223481	1.971756649	1.894490065
	2.291025471	1.497024473	1.894024972

	1.759694645	2.02521315	1.892453898
	1.759694645	2.02521315	1.892453898
	1.80338258	1.972821468	1.888102024
	1.933223177	1.839940894	1.886582035
	1.740248682	2.026197103	1.883222892
	2.280356352	1.483073462	1.881714907
	1.778732639	1.984150206	1.881441422
	1.828777587	1.925070328	1.876923957
	1.714777299	2.032983755	1.873880527
	2.261641248	1.483012992	1.87232712
	2.237552088	1.504306004	1.870929046
	2.249747901	1.485626689	1.867687295
	1.906728248	1.823964768	1.865346508
	1.727710046	1.9966528	1.862181423
	1.907049892	1.817267524	1.862158708
	1.7498212	1.974340243	1.862080721
	2.283411321	1.435341355	1.859376338
	1.788317253	1.927033649	1.857675451
	2.226137991	1.485797433	1.855967712
	2.233061255	1.477384852	1.855223053
	2.252822709	1.449052505	1.850937607
	1.805268859	1.887635839	1.846452349
	2.279421685	1.397708991	1.838565338
	1.955815818	1.718431986	1.837123902
	1.705289413	1.96597993	1.835634671
	2.20482103	1.46546672	1.835143875
	2.142533296	1.511514192	1.827023744
	2.610472597	1.038989052	1.824730825
	1.731762982	1.912754503	1.822258743
	2.360489435	1.266102309	1.813295872
	2.422057152	1.203036325	1.812546739
	2.245107525	1.377523353	1.811315439
	2.245107525	1.377523353	1.811315439
	1.612717417	1.999385584	1.806051501
	1.962209839	1.649693281	1.80595156
	2.158056682	1.450979924	1.804518303
	2.263756112	1.344679364	1.804217738
	2.271383921	1.336422016	1.803902968
	1.809359237	1.785213323	1.79728628
	2.245352714	1.334521331	1.789937022
	1.907115102	1.667829889	1.787472496
	2.168880248	1.401260415	1.785070331
	2.160467027	1.405648856	1.783057942
	2.160467027	1.405648856	1.783057942
	1.775476064	1.786318976	1.78089752
	1.726716155	1.832237695	1.779476925
	1.726716155	1.832237695	1.779476925

	2.219716665	1.335492702	1.777604684
	1.17563608	2.376492841	1.77606446
	2.203985845	1.347735776	1.775860811
	1.754025445	1.790387464	1.772206455
	2.201520469	1.338921258	1.770220864
	1.658039739	1.869558983	1.763799361
	2.205749725	1.317196218	1.761472971
	2.193456393	1.329069032	1.761262713
	2.097771927	1.422544635	1.760158281
	2.140741821	1.374427132	1.757584477
	1.74844191	1.764058454	1.756250182
	2.168697846	1.341217208	1.754957527
	2.163206144	1.338778897	1.75099252
	2.16216095	1.332700647	1.747430799
	1.611956811	1.881658302	1.746807557
	2.408487631	1.062927505	1.735707568
	2.171243555	1.270711089	1.720977322
	1.616027172	1.823094589	1.719560881
	2.256160015	1.172581822	1.714370919
	1.580604034	1.840601792	1.710602913
	1.580604034	1.840601792	1.710602913
	0.604324167	2.816739318	1.710531742
	2.199350129	1.219952189	1.709651159
	2.122969653	1.294827822	1.708898737
	2.335413573	1.075668415	1.705540994
	1.040314347	2.361429673	1.70087201
	1.608642341	1.791093968	1.699868155
	2.170514407	1.200822595	1.685668501
	0.792369418	2.562740911	1.677555164
	1.683229628	1.669895633	1.67656263
	1.453788648	1.889992527	1.671890587
	2.229960066	1.107303093	1.668631579
	1.491862032	1.843137206	1.667499619
	1.816988824	1.516701222	1.666845023
	1.815404721	1.491483567	1.653444144
	1.815404721	1.491483567	1.653444144
	1.702081033	1.602476662	1.652278848
	2.104042836	1.197200824	1.65062183
	1.739090436	1.561731493	1.650410964
	2.157225435	1.124645068	1.640935252
	1.489916942	1.772858697	1.63138782
	1.596791479	1.648830721	1.6228111
	1.679954884	1.561238252	1.620596568
	2.175391112	1.057858565	1.616624838
	1.455411742	1.743332582	1.599372162
	1.743502439	1.446301901	1.59490217
	1.435383868	1.750972357	1.593178113

1.740220421	1.443121615	1.591671018
1.740220421	1.443121615	1.591671018
1.740220421	1.443121615	1.591671018
1.465552548	1.704786524	1.585169536
1.465552548	1.704786524	1.585169536
1.602975259	1.555412386	1.579193823
2.078668847	1.067528528	1.573098688
2.0582215	1.086588075	1.572404787
0.250625191	2.893677307	1.572151249
2.173560019	0.958689796	1.566124907
2.111847539	1.004974334	1.558410936
2.111847539	1.004974334	1.558410936
1.328474085	1.774901523	1.551687804
0.094742318	2.992323135	1.543532726
0.102472909	2.982010675	1.542241792
1.530106953	1.528109556	1.529108255
1.626527108	1.422074197	1.524300653
1.319054169	1.728843791	1.52394898
0.079919921	2.954849109	1.517384515
1.548695359	1.484101178	1.516398268
1.518409278	1.509979056	1.514194167
1.518409278	1.509979056	1.514194167
1.518409278	1.509979056	1.514194167
1.518409278	1.509979056	1.514194167
2.053465468	0.940487834	1.496976651
2.010811514	0.982571577	1.496691545
0.084089909	2.908977782	1.496533845
2.119795593	0.865982027	1.49288881
0.243055239	2.740209476	1.491632357
1.281811287	1.695203012	1.48850715
0.050337487	2.921974724	1.486156106
1.366839557	1.599094018	1.482966788
1.509560264	1.447962742	1.478761503
1.412153186	1.524215135	1.46818416
1.459179436	1.476049414	1.467614425
0.045502483	2.888263021	1.466882752
0.118110334	2.787714676	1.452912505
0.211849125	2.690134991	1.450992058
0.20517931	2.691357769	1.448268539
0.202323511	2.668194206	1.435258858
0.032620805	2.832099587	1.432360196
1.55151158	1.308750744	1.430131162
0.091549807	2.762494634	1.42702222
-0.026519326	2.879585111	1.426532892
2.167374514	0.684160778	1.425767646
0.144205159	2.68567924	1.4149422
0.109508255	2.715974747	1.412741501

0.013879325	2.794333364	1.404106345
1.782902917	1.008073184	1.395488051
1.284103678	1.503288324	1.393696001
-0.017480572	2.796369379	1.389444403
0.182555906	2.58584044	1.384198173
-0.050517409	2.809404492	1.379443541
0.168453635	2.576523955	1.372488795
-0.023514384	2.764444669	1.370465143
1.352390117	1.377008004	1.36469906
1.352390117	1.377008004	1.36469906
0.109846848	2.616244534	1.363045691
0.037411159	2.686590196	1.362000678
-0.069248666	2.78812741	1.359439372
0.041485035	2.668072945	1.35477899
2.705494198	0	1.352747099
0.039034467	2.666209604	1.352622035
-0.021106805	2.725471465	1.35218233
-0.25110023	2.954553331	1.351726551
0.035564116	2.667397798	1.351480957
0.003860657	2.698991133	1.351425895
0.003145023	2.698548068	1.350846545
-0.007299584	2.700469219	1.346584817
0.138265331	2.554861941	1.346563636
0.032589228	2.658637246	1.345613237
0.076702678	2.607130716	1.341916697
0.016433817	2.665553762	1.34099379
0.000185	2.676725387	1.338455329
0.071843999	2.597503497	1.334673748
-0.052549776	2.701252212	1.324351218
2.381689639	0.256871458	1.319280549
-0.007165648	2.63520642	1.314020386
-0.006182802	2.624857446	1.309337322
-0.006182802	2.624857446	1.309337322
-0.042724641	2.642190883	1.299733121
1.177847209	1.414449943	1.296148576
-0.171689674	2.761364691	1.294837508
-0.171689674	2.761364691	1.294837508
-0.115955336	2.701020614	1.292532639
-0.115955336	2.701020614	1.292532639
2.42245384	0.146131044	1.284292442
-0.051893002	2.608561668	1.278334333
-0.051893002	2.608561668	1.278334333
-0.032458017	2.589047661	1.278294822
1.365841317	1.174710944	1.270276131
-0.026175099	2.564476326	1.269150614
-0.107344897	2.628231555	1.260443329
2.466347269	0	1.233173635

2.460555374	0	1.230277687
2.445633418	0	1.222816709
0.744844749	1.696849798	1.220847273
0.430524771	2.001461793	1.215993282
2.419495634	0	1.209747817
0	2.417727293	1.208863647
1.678814864	0.726955664	1.202885264
2.392364384	0	1.196182192
2.058957823	0.329227036	1.19409243
1.250519601	1.134772311	1.192645956
2.380244576	0	1.190122288
1.496559561	0.882400904	1.189480232
2.377894326	0	1.188947163
0	2.372982649	1.186491324
2.370948499	0	1.185474249
2.370268689	0	1.185134345
0	2.363350869	1.181675435
0	2.357125127	1.178562564
1.179629772	1.176624063	1.178126917
2.354241859	0	1.17712093
2.35054329	0	1.175271645
2.335746515	0	1.167873257
2.334184883	0	1.167092441
2.333629004	0	1.166814502
2.331515514	0	1.165757757
2.323749975	0	1.161874988
0	2.321450606	1.160725303
2.32126602	0	1.16063301
2.321064004	0	1.160532002
2.321064004	0	1.160532002
2.315707735	0	1.157853868
1.240458635	1.073148157	1.156803396
2.309919324	0	1.154959662
2.305556391	0	1.152778196
2.304791429	0	1.152395714
2.30213714	0	1.15106857
0.97931991	1.318035522	1.148677716
1.572795186	0.711281949	1.142038567
2.033846371	0.228524416	1.131185393
1.190350316	1.065693074	1.128021695
0	2.248237846	1.124118923
2.245125968	0	1.122562984
0	2.231157624	1.115578812
0	2.219055196	1.109527598
0	2.217731819	1.10886591
0.962359542	1.23557557	1.098967556
2.193276541	0	1.09663827

0	2.192654213	1.096327106
1.587165188	0.602804193	1.094984691
1.155712536	1.024954167	1.090333351
1.318759422	0.85168648	1.085222951
0	2.16973538	1.08486769
2.15408934	0.011327437	1.082708389
1.135150886	1.029644932	1.082397909
1.135150886	1.029644932	1.082397909
1.135150886	1.029644932	1.082397909
2.160730509	0	1.080365254
2.203723252	-0.057300442	1.073211405
1.137792053	0.998077927	1.06793499
1.067812275	1.065193512	1.066502894
0.27296484	1.856336608	1.064650724
2.120004641	0	1.06000232
2.116191435	0	1.058095717
1.097936421	1.004824569	1.051380495
1.10374118	0.974490342	1.039115761
0.217083082	1.855002978	1.03604303
1.110315488	0.959142732	1.03472911
2.10053684	-0.032309333	1.034113753
1.08071622	0.986147148	1.033431684
2.519880642	-0.470762209	1.024559217
2.04004551	0	1.020022755
2.036837125	0	1.018418563
2.37484092	-0.344573468	1.015133726
2.016927893	0	1.008463946
2.008464859	0	1.004232429
0	2.004361389	1.002180695
2.003601358	0	1.001800679
1.986592148	0	0.993296074
1.770955224	0.214020242	0.992487733
1.184782854	0.798014499	0.991398676
1.973397905	0	0.986698953
1.973397905	0	0.986698953
2.792688377	-0.824139954	0.984274212
1.964221118	0	0.982110559
1.963692825	0	0.981846413
0.96551155	0.995534319	0.980522935
1.960891775	0	0.980445888
1.9483405	0	0.97417025
1.94357727	0	0.971788635
1.941659642	0	0.970829821
2.202707109	-0.264048227	0.969329441
2.242583636	-0.308264529	0.967159553
2.242583636	-0.308264529	0.967159553
1.929924595	0	0.964962298

	1.067884503	0.861493046	0.964688775
	1.921462854	0	0.960731427
	2.181175694	-0.262986625	0.959094535
	2.153553863	-0.237781856	0.957886003
	2.220052293	-0.30478532	0.957633486
	2.142750665	-0.232897196	0.954926735
	1.908091365	0	0.954045682
	0.868663127	1.028766312	0.948714719
	2.031146414	-0.135471525	0.947837445
	1.881787425	0	0.940893712
	1.877039552	0	0.938519776
	1.859050157	0	0.929525078
	1.724147362	0.133434834	0.928791098
	2.409289769	-0.555604509	0.92684263
	1.84975231	0	0.924876155
	1.065181276	0.780247874	0.922714575
	1.095305706	0.748631446	0.921968576
	1.095232369	0.748603759	0.921918064
	0.951819477	0.889213084	0.92051628
	1.840053932	0	0.920026966
	1.840053932	0	0.920026966
	2.236503318	-0.397946586	0.919278366
	1.837798587	0	0.918899293
	1.83413312	0	0.91706656
	1.823569192	0	0.911784596
	1.307189027	0.514138536	0.910663781
	1.100579605	0.720511043	0.910545324
	1.801355315	0	0.900677657
	2.164779248	-0.37829584	0.893241704
	2.187141931	-0.402534453	0.892303739
	2.084987796	-0.301625434	0.891681181
	1.050678627	0.732279422	0.891479025
	1.782677413	0	0.891338707
	2.199261989	-0.417401455	0.890930267
	2.198127827	-0.418104013	0.890011907
	2.195809747	-0.417436733	0.889186507
	1.776519319	0	0.888259659
	2.178026971	-0.403886534	0.887070219
	2.033718346	-0.261833353	0.885942496
	1.765757843	0	0.882878922
	0.885377091	0.873554477	0.879465784
	2.201095849	-0.444965538	0.878065155
	2.187940102	-0.431939841	0.87800013
	2.160901765	-0.429290637	0.865805564
	1.727600594	0	0.863800297
	2.168571256	-0.445393042	0.861589107
	2.251413284	-0.529122328	0.861145478

1.720263607	0	0.860131803
2.104652979	-0.389046981	0.857802999
2.104652979	-0.389046981	0.857802999
0.873791618	0.81562115	0.844706384
2.196154233	-0.520889357	0.837632438
0.750579096	0.913277452	0.831928274
1.651093899	0	0.825546949
0.626603067	1.020213347	0.823408207
1.642367523	0	0.821183762
2.294196484	-0.664072675	0.815061904
0.676216796	0.947864324	0.81204056
1.585740621	0.023239308	0.804489965
2.124979445	-0.517034946	0.80397225
0.977534852	0.628104626	0.802819739
1.604785577	0	0.802392789
1.102086544	0.482401507	0.792244026
1.120183494	0.436229691	0.778206592
1.340652574	0.202448617	0.771550596
2.106575209	-0.57576333	0.765405939
1.650249317	-0.130543363	0.759852977
2.037486197	-0.528349161	0.754568518
1.972323846	-0.470015523	0.751154161
0.524832252	0.965451708	0.74514198
0.579979226	0.895579905	0.737779566
1.03159067	0.443698981	0.737644826
0.87328106	0.599894368	0.736587714
0	1.466583597	0.733291799
0.873785505	0.584804944	0.729295224
0.805422943	0.650082668	0.727752806
0.672838414	0.760072807	0.71645561
1.752258555	-0.320061055	0.71609875
2.026480279	-0.597021818	0.714729231
2.026480279	-0.597021818	0.714729231
1.936422825	-0.524973257	0.705724784
0.925894225	0.483708601	0.704801413
0	1.40958262	0.70479131
2.153231365	-0.761091603	0.696069881
2.147590529	-0.762177183	0.692706673
2.146775025	-0.762479099	0.692147963
2.002488681	-0.625651038	0.688418822
1.731471699	-0.365757952	0.682856874
0.437231535	0.918482119	0.677856827
0.719913574	0.626669456	0.673291515
1.629221325	-0.303811704	0.662704811
0.752304089	0.567677326	0.659990707
2.123761138	-0.805127795	0.659316671
1.310652727	0	0.655326363

0	1.301776346	0.650888173
1.293762892	0	0.646881446
0.776469517	0.516843043	0.64665628
2.118949696	-0.846235664	0.636357016
0.900236539	0.365584565	0.632910552
1.614452987	-0.365685607	0.62438369
0.690861592	0.542574537	0.616718065
2.256253084	-1.034539449	0.610856817
1.68217658	-0.467063557	0.607556512
2.090658073	-0.876370015	0.607144029
2.090658073	-0.876370015	0.607144029
1.576220993	-0.370659329	0.602780832
1.576220993	-0.370659329	0.602780832
0.567009025	0.62989945	0.598454237
2.028684886	-0.852951113	0.587866886
1.675159775	-0.502609048	0.586275363
2.007893519	-0.8684766	0.569708459
1.597822714	-0.474419938	0.561701388
-0.175101523	1.280817576	0.552858026
0.551226534	0.553104565	0.552165549
1.057122218	0.0352843	0.546203259
1.696220936	-0.607116455	0.54455224
0.183052431	0.904531969	0.5437922
0.640736762	0.443562245	0.542149504
0.63517754	0.43991339	0.537545465
0	1.063682357	0.531841178
0.222988871	0.838712806	0.530850839
0.379157357	0.676232049	0.527694703
0.555104101	0.492039925	0.523572013
0	1.041480877	0.520740438
0	1.036804005	0.518402003
0	1.036804005	0.518402003
1.620510287	-0.584261647	0.51812432
0.689360385	0.333741962	0.511551174
0.483451466	0.538612563	0.511032015
2.006616111	-0.992296359	0.507159876
0.440822625	0.55733612	0.499079373
0.984290838	0	0.492145419
0.461952248	0.521983333	0.49196779
0.685682774	0.293300225	0.4894915
1.536658633	-0.560075446	0.488291593
0.458026585	0.512447963	0.485237274
0	0.96830743	0.484153715
0.456470633	0.511749906	0.48411027
0.386587538	0.557752643	0.47217009
0.412789744	0.527802147	0.470295946
0.303353898	0.621795916	0.462574907

	0.303353898	0.621795916	0.462574907
	0.833034657	0.083492863	0.45826376
	0.832971277	0.083484413	0.458227845
	0.640902744	0.271745171	0.456323958
	0.481916176	0.416191405	0.449053791
	0	0.889554469	0.444777234
	0.479620892	0.405447063	0.442533977
	0.507961616	0.36283274	0.435397178
	2.112493282	-1.257184638	0.427654322
	0.252434366	0.591396142	0.421915254
	0.602798362	0.22137997	0.412089166
	1.030960444	-0.207499663	0.411730391
	0.49270807	0.329216903	0.410962486
	0.796399637	0.020624618	0.408512127
	0.95279966	-0.136796222	0.408001719
	0	0.807302616	0.403651308
	0.402737001	0.392884447	0.397810724
	0.286416975	0.505707434	0.396062205
	0.373565762	0.391918061	0.382741912
	0	0.760790891	0.380395446
	0	0.760790891	0.380395446
	0.193167539	0.562740701	0.37795412
	0.262950387	0.487919183	0.375434785
	0.453026127	0.292880635	0.372953381
	0.196099585	0.549450742	0.372775164
	0	0.742394802	0.371197401
	0.517287091	0.22314896	0.370218026
	0.090598105	0.648866576	0.369732341
	0.331529496	0.399626689	0.365578093
	0.362224881	0.363034269	0.362629575
	0.611161729	0.112873163	0.362017446
	0.269332846	0.448397916	0.358865381
	0.147649818	0.567615451	0.357632635
	0	0.712833296	0.356416648
	0.204219631	0.50826078	0.356240205
	0.070967894	0.636800305	0.3538841
	0.740620793	-0.034931257	0.352844768
	0.071656721	0.629952564	0.350804642
	0	0.687159207	0.343579604
	0	0.687159207	0.343579604
	0.074416702	0.612464518	0.34344061
	0.219192544	0.466020379	0.342606461
	0	0.678389608	0.339194804
	0	0.674242964	0.337121482
	1.800016528	-1.13051658	0.334749974
	0	0.66716553	0.333582765
	0.075609692	0.582304923	0.328957308

0.401872832	0.254399826	0.328136329
-0.071292227	0.724060927	0.32638435
0.360409601	0.276336353	0.318372977
0.076796208	0.559099994	0.317948101
0	0.632761636	0.316380818
0	0.631374371	0.315687185
0	0.624986882	0.312493441
0	0.621373216	0.310686608
0	0.620763593	0.310381797
0.318023916	0.302583483	0.310303699
0.398964417	0.220879295	0.309921856
0	0.595060585	0.297530292
0.064644084	0.523825032	0.294234558
0.584214884	0	0.292107442
0.248545612	0.335484768	0.29201519
0.435263149	0.135344981	0.285304065
0.35262698	0.210350446	0.281488713
0	0.562004412	0.281002206
0.258253829	0.302623523	0.280438676
0.335279994	0.224408791	0.279844393
0.557706968	0	0.278853484
0.556635507	0	0.278317754
0.244599279	0.30875962	0.27667945
0	0.551149508	0.275574754
0.247947378	0.274050918	0.260999148
0.213163282	0.305604122	0.259383702
0.178399958	0.334624355	0.256512157
0.676973298	-0.169174073	0.253899612
-0.039833849	0.534225178	0.247195665
0	0.458995719	0.229497859
0	0.441170539	0.220585269
0.681355179	-0.251618879	0.21486815
0	0.410488326	0.205244163
0	0.401110601	0.200555301
0	0.389247491	0.194623745
0	0.385585405	0.192792702
0.111023837	0.272354536	0.191689186
0.208623643	0.172426814	0.190525228
0	0.378566641	0.18928332
0	0.375598382	0.187799191
0.501480364	-0.139992452	0.180743956
0	0.351983491	0.175991745
0.259977593	0.089926671	0.174952132
0.359000786	-0.010785367	0.174107709
0	0.348091873	0.174045936
0.079744634	0.260768654	0.170256644
0.079744634	0.260768654	0.170256644

0.188903789	0.141983468	0.165443628
0.176095425	0.152183866	0.164139646
0.276266774	0.049648194	0.162957484
0.273553096	0.042210241	0.157881668
0.427234241	-0.111601963	0.157816139
-0.154558948	0.462906	0.154173526
0.470632119	-0.163121292	0.153755413
0.241167595	0.065885739	0.153526667
0.032408051	0.27427118	0.153339616
0.1839075	0.11842787	0.151167685
0.369399183	-0.070223796	0.149587693
0.08122318	0.21778701	0.149505095
0.289535967	0	0.144767984
-0.332722423	0.615941935	0.141609756
-0.144417209	0.426668032	0.141125411
0.276619098	0	0.138309549
0.151946063	0.106467668	0.129206866
0.151946063	0.106467668	0.129206866
-0.188808843	0.443527877	0.127359517
0	0.246220734	0.123110367
0.450937457	-0.20483547	0.123050993
0	0.245253788	0.122626894
0.243013829	0	0.121506915
0	0.238173331	0.119086665
0.10939188	0.126852742	0.118122311
0	0.235037152	0.117518576
0.338860856	-0.109944388	0.114458234
0.182185624	0.045886486	0.114036055
0	0.227897031	0.113948515
0.150504981	0.073654245	0.112079613
0.390355669	-0.16830541	0.11102513
0.149702456	0.069018248	0.109360352
0.218188807	0	0.109094403
0	0.217081315	0.108540658
0.415918637	-0.20223929	0.106839674
0.169011497	0.038452531	0.103732014
0.097867298	0.107641047	0.102754172
0.203451177	0	0.101725588
0.225820182	-0.022852394	0.101483894
0	0.19929506	0.09964753
0.197423861	0	0.098711931
0.197423861	0	0.098711931
-0.032264863	0.2267541	0.097244619
0.323461888	-0.130601798	0.096430045
0	0.192741747	0.096370873
-0.078207312	0.270480327	0.096136508
0.349654434	-0.162188963	0.093732736

0	0.186397208	0.093198604
0.175867343	0.005535817	0.09070158
0.048285694	0.130106578	0.089196136
0.173857714	0	0.086928857
0.169215506	0	0.084607753
0.167863722	0	0.083931861
0	0.166494642	0.083247321
0	0.154046881	0.07702344
0	0.154016917	0.077008458
0.123594537	0.029823187	0.076708862
-0.010858914	0.16399867	0.076569878
0.141671396	0	0.070835698
0.446032008	-0.304563257	0.070734375
0.242717529	-0.106620232	0.068048649
0.099072461	0.036086091	0.067579276
-0.051393534	0.182495993	0.06555123
0.075717722	0.051968805	0.063843264
0.119764258	0	0.059882129
0.43360184	-0.315130981	0.05923543
0	0.103889069	0.051944535
0	0.099426399	0.0497132
0	0.098378688	0.049189344
0	0.098378688	0.049189344
0	0.098378688	0.049189344
0.129246085	-0.03223999	0.048503048
0.108163101	-0.015922939	0.046120081
0.088858598	0	0.044429299
0	0.086366047	0.043183024
0.170701302	-0.08528288	0.042709211
0	0.082714434	0.041357217
0.067829684	0.014673043	0.041251363
0	0.081637284	0.040818642
0.129981066	-0.054013734	0.037983666
0.082817048	-0.007371691	0.037722679
0.064010799	0.010226357	0.037118578
0.07345702	0	0.03672851
0.075255433	-0.002011505	0.036621964
0.07154107	0.000874	0.036207484
0.07154107	0.000874	0.036207484
0.069069698	0	0.034534849
0.092135795	-0.023091612	0.034522092
0.09150161	-0.027505365	0.031998123
0	0.062203891	0.031101945
0	0.061934156	0.030967078
0.056777239	0	0.028388619
0.056777239	0	0.028388619
0.056009324	0	0.028004662

0	0.055560144	0.027780072
0	0.055560144	0.027780072
0.050396597	0.002693016	0.026544807
0	0.051806901	0.025903451
0	0.0517933	0.02589665
0.399116912	-0.349318033	0.024899439
0.044705949	0	0.022352974
-0.244975473	0.287302347	0.021163437
0	0.039249275	0.019624638
0.128155574	-0.089026924	0.019564325
0	0.038189842	0.019094921
-0.086561008	0.124392022	0.018915507
0	0.037267785	0.018633892
0	0.035675545	0.017837773
0.033609524	-0.000308	0.016650941
0	0.032211492	0.016105746
0	0.031578203	0.015789102
0	0.030506878	0.015253439
0.026104955	0	0.013052478
0.025798989	0	0.012899494
0	0.025531132	0.012765566
0	0.024161586	0.012080793
0.028783917	-0.00584136	0.011471278
0	0.020461159	0.010230579
0.029237766	-0.011846662	0.008695552
0	0.015455986	0.007727993
0	0.014227671	0.007113835
0.20993719	-0.19621213	0.00686253
0	0.011568579	0.00578429
0	0.011568579	0.00578429
0.009354097	0	0.004677049
0.009319114	0	0.004659557
-0.067642609	0.075924013	0.004140702
0.042316881	-0.03492671	0.003695086
0.042316881	-0.03492671	0.003695086
0.0307205	-0.024358079	0.003181211
0.270326921	-0.264784414	0.002771254
0	0.003336312	0.001668156
0.025133112	-0.023737712	0.000698
0.024580552	-0.0243932	0.0000937
0	0	0
0	0	0
0	0	0
0	0	0
0	0	0
0	0	0
0	0	0

	0	0	0
	0	0	0
	0	0	0
	0	0	0
	0	0	0
	0	0	0
	0	0	0
	0	0	0
	0	0	0
	0	0	0
	0	0	0
	0	0	0
	0	0	0
	0	0	0
	0	0	0
	0	0	0
	0	0	0
	0	0	0
	0	0	0
	0	0	0
	0	0	0
	0	0	0
	0	0	0
	0	0	0
	0	0	0
	0	0	0
	0	0	0
	0	0	0
	0	0	0
	0	0	0
	0	0	0
	0	0	0
	0	0	0
	0	0	0
	0	0	0
	0	0	0
	0	0	0
	0	0	0
	0	0	0
	0	0	0
	0	0	0
	0	0	0
	0	0	0
	0	0	0
	0	0	0
	0	0	0
	0	0	0

	0	0	0
	0	0	0
	0	0	0
	0	0	0
	0	0	0
	0	0	0
	0	0	0
	0	0	0
	0	0	0
	0	0	0
	0	0	0
	0	0	0
	0	0	0
	0	0	0
	0	0	0
	0	0	0
	0	0	0
	0	0	0
	0	0	0
	0	0	0
	0	0	0
	0	0	0
	0	0	0
	0	0	0
	0	0	0
	0	0	0
	0	0	0
	0	0	0
	0	0	0
	0	0	0
	0	0	0
	0	0	0
	0	0	0
	0	0	0
	0	0	0
	0	0	0
	0	0	0
	0	0	0
	0	0	0
	0	0	0
	0	0	0
	0	0	0
	0	0	0
	0	0	0
	0	0	0
	0	0	0
	0	0	0

	0	0	0
	0	0	0
	0	0	0
	0	0	0
	0	0	0
	0	0	0
	0	0	0
	0	0	0
	0	0	0
	0	0	0
	0	0	0
	0	0	0
	0	0	0
	0	0	0
	0	0	0
	0	0	0
	0	0	0
	0	0	0
	0	0	0
	0	0	0
	0	0	0
	0	0	0
	0	0	0
	0	0	0
	0	0	0
	0	0	0
	0	0	0
	0	0	0
	0	0	0
	0	0	0
	0	0	0
	0	0	0
	0	0	0
	0	0	0
	0	0	0
	0	0	0
	0	0	0
	0	0	0
	0	0	0
	0	0	0
	0	0	0
	0	0	0
	0	0	0
	0	0	0
	0	0	0
	0	0	0
	0	0	0

	0	0	0
	0	0	0
	0	0	0
	0	0	0
	0	0	0
	0	0	0
	0	0	0
	0	0	0
	0	0	0
	0	0	0
	0	0	0
	0	0	0
	0	0	0
	0	0	0
	0	0	0
	0	0	0
	0	0	0
	0	0	0
	0	0	0
	0	0	0
	0	0	0
	0	0	0
	0	0	0
	0	0	0
	0	0	0
	0	0	0
	0	0	0
	0	0	0
	0	0	0
	0	0	0
	0	0	0
	0	0	0
	0	0	0
	0	0	0
	0	0	0
	0	0	0
	0	0	0
	0	0	0
	0	0	0
	0	0	0
	0	0	0
	0	0	0
	0	0	0
	0	0	0
	0	0	0
	0	0	0
	0	0	0

	0	0	0
	0	0	0
	0	0	0
	0	0	0
	0	0	0
	0	0	0
	0	0	0
	0	0	0
	0	0	0
	0	0	0
	0	0	0
	0	0	0
	0	0	0
	0	0	0
	0	0	0
	0	0	0
	0	0	0
	0	0	0
	0	0	0
	0	0	0
	0	0	0
	0	0	0
	0	0	0
	0	0	0
	0	0	0
	0	0	0
	0	0	0
	0	0	0
	0	0	0
	0	0	0
	0	0	0
	0	0	0
	0	0	0
	0	0	0
	0	0	0
	0	0	0
	0	0	0
	0	0	0
	0	0	0
	0	0	0
	0	0	0
	0	0	0
	0	0	0
	0	0	0
	0	0	0
	0	0	0
	0	0	0

	0	0	0
	0	0	0
	0	0	0
	0	0	0
	0	0	0
	0	0	0
	0	0	0
	0	0	0
	0	0	0
	0	0	0
	0	0	0
	0	0	0
	0	0	0
	0	0	0
	0	0	0
	0	0	0
	0	0	0
	0	0	0
	0	0	0
	0	0	0
	0	0	0
	0	0	0
	0	0	0
	0	0	0
	0	0	0
	0	0	0
	0	0	0
	0	0	0
	0	0	0
	0	0	0
	0	0	0
	0	0	0
	0	0	0
	0	0	0
	0	0	0
	0	0	0
	0	0	0
	0	0	0
	0	0	0
	0	0	0
	0	0	0
	0	0	0
	0	0	0
	0	0	0
	0	0	0
	0	0	0
	0	0	0

	0	0	0
	0	0	0
	0	0	0
	0	0	0
	0	0	0
	0	0	0
	0	0	0
	0	0	0
	0	0	0
	0	0	0
	0	0	0
	0	0	0
	0	0	0
	0	0	0
	0	0	0
	0	0	0
	0	0	0
	0	0	0
	0	0	0
	0	0	0
	0	0	0
	0	0	0
	0	0	0
	0	0	0
	0	0	0
	0	0	0
	0	0	0
	0	0	0
	0	0	0
	0	0	0
	0	0	0
	0	0	0
	0	0	0
	0	0	0
	0	0	0
	0	0	0
	0	0	0
	0	0	0
	0	0	0
	0	0	0
	0	0	0
	0	0	0
	0	0	0
	0	0	0
	0	0	0
	0	0	0
	0	0	0

	0	0	0
	0	0	0
	0	0	0
	0	0	0
	0	0	0
	0	0	0
	0	0	0
	0	0	0
	0	0	0
	0	0	0
	0	0	0
	0	0	0
	0	0	0
	0	0	0
	0	0	0
	0	0	0
	0	0	0
	0	0	0
	0	0	0
	0	0	0
	0	0	0
	0	0	0
	0	0	0
	0	0	0
	0	0	0
	0	0	0
	0	0	0
	0	0	0
	0	0	0
	0	0	0
	0	0	0
	0	0	0
	0	0	0
	0	0	0
	0	0	0
	0	0	0
	0	0	0
	0	0	0
	0	0	0
	0	0	0
	0	0	0
	0	0	0
	0	0	0
	0	0	0
	0	0	0
	0	0	0
	0	0	0

	0	0	0
	0	0	0
	0	0	0
	0	0	0
	0	0	0
	0	0	0
	0	0	0
	0	0	0
	0	0	0
	0	0	0
	0	0	0
	0	0	0
	0	0	0
	0	0	0
	0	0	0
	0	0	0
	0	0	0
	0	0	0
	0	0	0
	0	0	0
	0	0	0
	0	0	0
	0	0	0
	0	0	0
	0	0	0
	0	0	0
	0	0	0
	0	0	0
	0	0	0
	0	0	0
	0	0	0
	0	0	0
	0	0	0
	0	0	0
	0	0	0
	0	0	0
	0	0	0
	0	0	0
	0	0	0
	0	0	0
	0	0	0
	0	0	0
	0	0	0
	0	0	0
	0	0	0
	0	0	0
	0	0	0

	0	0	0
	0	0	0
	0	0	0
	0	0	0
	0	0	0
	0	0	0
	0	0	0
	0	0	0
	0	0	0
	0	0	0
	0	0	0
	0	0	0
	0	0	0
	0	0	0
	0	0	0
	0	0	0
	0	0	0
	0	0	0
	0	0	0
	0	0	0
	0	0	0
	0	0	0
	0	0	0
	0	0	0
	0	0	0
	0	0	0
	0	0	0
	0	0	0
	0	0	0
	0	0	0
	0	0	0
	0	0	0
	0	0	0
	0	0	0
	0	0	0
	0	0	0
	0	0	0
	0	0	0
	0	0	0
	0	0	0
	0	0	0
	0	0	0
	0	0	0
	0	0	0
	0	0	0
	0	0	0
	0	0	0

	0	0	0
	0	0	0
	0	0	0
	0	0	0
	0	0	0
	0	0	0
	0	0	0
	0	0	0
	0	0	0
	0	0	0
	0	0	0
	0	0	0
	0	0	0
	0	0	0
	0	0	0
	0	0	0
	0	0	0
	0	0	0
	0	0	0
	0	0	0
	0	0	0
	0	0	0
	0	0	0
	0	0	0
	0	0	0
	0	0	0
	0	0	0
	0	0	0
	0	0	0
	0	0	0
	0	0	0
	0	0	0
	0	0	0
	0	0	0
	0	0	0
	0	0	0
	0	0	0
	0	0	0
	0	0	0
	0	0	0
	0	0	0
	0	0	0
	0	0	0
	0	0	0
	0	0	0
	0	0	0
	0	0	0

0	0	0
0	0	0
0	0	0
0	0	0
0	0	0
0	0	0
0	0	0
0	0	0
0	0	0
0	0	0
0	0	0
0	0	0
0	0	0
0	0	0
0	0	0
0	0	0
0	0	0
0	0	0
0	0	0
0	0	0
0	0	0
0	0	0
0	0	0
0	0	0
0	0	0
0	0	0
0	0	0
0	0	0
0	0	0
0	0	0
0	0	0
0	0	0
0	0	0
0	0	0
0	0	0
0	0	0
0	0	0
0	0	0
0	0	0
0	0	0
0	0	0
0	0	0
0	0	0
0	0	0
0	0	0
0	0	0
0	0	0

	0	0	0
	0	0	0
	0	0	0
	0	0	0
	0	0	0
	0	0	0
	0	0	0
	0	0	0
	0	0	0
	0	0	0
	0	0	0
	0	0	0
	0	0	0
	0	0	0
	0	0	0
	0	0	0
	0	0	0
	0	0	0
	0	0	0
	0	0	0
	0	0	0
	0	0	0
	0	0	0
	0	0	0
	0	0	0
	0	0	0
	0	0	0
	0	0	0
	0	0	0
	0	0	0
	0	0	0
	0	0	0
	0	0	0
	0	0	0
	0	0	0
	0	0	0
	0	0	0
	0	0	0
	0	0	0
	0	0	0
	0	0	0
	0	0	0
	0	0	0
	0	0	0
	0	0	0
	0	0	0
	0	0	0

	0	0	0
	0	0	0
	0	0	0
	0	0	0
	0	0	0
	0	0	0
	0	0	0
	0	0	0
	0	0	0
	0	0	0
	0	0	0
	0	0	0
	0	0	0
	0	0	0
	0	0	0
	0	0	0
	0	0	0
	0	0	0
	0	0	0
	0	0	0
	0	0	0
	0	0	0
	0	0	0
	0	0	0
	0	0	0
	0	0	0
	0	0	0
	0	0	0
	0	0	0
	0	0	0
	0	0	0
	0	0	0
	0	0	0
	0	0	0
	0	0	0
	0	0	0
	0	0	0
	0	0	0
	0	0	0
	0	0	0
	0	0	0
	0	0	0
	0	0	0
	0	0	0
	0	0	0
	0	0	0
	0	0	0

	0	0	0
	0	0	0
	0	0	0
	0	0	0
	0	0	0
	0	0	0
	0	0	0
	0	0	0
	0	0	0
	0	0	0
	0	0	0
	0	0	0
	0	0	0
	0	0	0
	0	0	0
	0	0	0
	0	0	0
	0	0	0
	0	0	0
	0	0	0
	0	0	0
	0	0	0
	0	0	0
	0	0	0
	0	0	0
	0	0	0
	0	0	0
	0	0	0
	0	0	0
	0	0	0
	0	0	0
	0	0	0
	0	0	0
	0	0	0
	0	0	0
	0	0	0
	0	0	0
	0	0	0
	0	0	0
	0	0	0
	0	0	0
	0	0	0
	0	0	0
	0	0	0
	0	0	0
	0	0	0
	0	0	0

	0	0	0
	0	0	0
	0	0	0
	0	0	0
	0	0	0
	0	0	0
	0	0	0
	0	0	0
	0	0	0
	0	0	0
	0	0	0
	0	0	0
	0	0	0
	0	0	0
	0	0	0
	0	0	0
	0	0	0
	0	0	0
	0	0	0
	0	0	0
	0	0	0
	0	0	0
	0	0	0
	0	0	0
	0	0	0
	0	0	0
	0	0	0
	0	0	0
	0	0	0
	0	0	0
	0	0	0
	0	0	0
	0	0	0
	0	0	0
	0	0	0
	0	0	0
	0	0	0
	0	0	0
	0	0	0
	0	0	0
	0	0	0
	0	0	0
	0	0	0
	0	0	0
	0	0	0
	0	0	0
	0	0	0

	0	0	0
	0	0	0
	0	0	0
	0	0	0
	0	0	0
	0	0	0
	0	0	0
	0	0	0
	0	0	0
	0	0	0
	0	0	0
	0	0	0
	0	0	0
	0	0	0
	0	0	0
	0	0	0
	0	0	0
	0	0	0
	0	0	0
	0	0	0
	0	0	0
	0	0	0
	0	0	0
	0	0	0
	0	0	0
	0	0	0
	0	0	0
	0	0	0
	0	0	0
	0	0	0
	0	0	0
	0	0	0
	0	0	0
	0	0	0
	0	0	0
	0	0	0
	0	0	0
	0	0	0
	0	0	0
	0	0	0
	0	0	0
	0	0	0
	0	0	0
	0	0	0
	0	0	0
	0	0	0
	0	0	0

	0	0	0
	0	0	0
	0	0	0
	0	0	0
	0	0	0
	0	0	0
	0	0	0
	0	0	0
	0	0	0
	0	0	0
	0	0	0
	0	0	0
	0	0	0
	0	0	0
	0	0	0
	0	0	0
	0	0	0
	0	0	0
	0	0	0
	0	0	0
	0	0	0
	0	0	0
	0	0	0
	0	0	0
	0	0	0
	0	0	0
	0	0	0
	0	0	0
	0	0	0
	0	0	0
	0	0	0
	0	0	0
	0	0	0
	0	0	0
	0	0	0
	0	0	0
	0	0	0
	0	0	0
	0	0	0
	0	0	0
	0	0	0
	0	0	0
	0	0	0
	0	0	0
	0	0	0
	0	0	0
	0	0	0

	0	0	0
	0	0	0
	0	0	0
	0	0	0
	0	0	0
	0	0	0
	0	0	0
	0	0	0
	0	0	0
	0	0	0
	0	0	0
	0	0	0
	0	0	0
	0	0	0
	0	0	0
	0	0	0
	0	0	0
	0	0	0
	0	0	0
	0	0	0
	0	0	0
	0	0	0
	0	0	0
	0	0	0
	0	0	0
	0	0	0
	0	0	0
	0	0	0
	0	0	0
	0	0	0
	0	0	0
	0	0	0
	0	0	0
	0	0	0
	0	0	0
	0	0	0
	0	0	0
	0	0	0
	0	0	0
	0	0	0
	0	0	0
	0	0	0
	0	0	0
	0	0	0
	0	0	0
	0	0	0
	0	0	0

	0	0	0
	0	0	0
	0	0	0
	0	0	0
	0	0	0
	0	0	0
	0	0	0
	0	0	0
	0	0	0
	0	0	0
	0	0	0
	0	0	0
	0	0	0
	0	0	0
	0	0	0
	0	0	0
	0	0	0
	0	0	0
	0	0	0
	0	0	0
	0	0	0
	0	0	0
	0	0	0
	0	0	0
	0	0	0
	0	0	0
	0	0	0
	0	0	0
	0	0	0
	0	0	0
	0	0	0
	0	0	0
	0	0	0
	0	0	0
	0	0	0
	0	0	0
	0	0	0
	0	0	0
	0	0	0
	0	0	0
	0	0	0
	0	0	0
	0	0	0
	0	0	0
	0	0	0
	0	0	0
	0	0	0

	0	0	0
	0	0	0
	0	0	0
	0	0	0
	0	0	0
	0	0	0
	0	0	0
	0	0	0
	0	0	0
	0	0	0
	0	0	0
	0	0	0
	0	0	0
	0	0	0
	0	0	0
	0	0	0
	0	0	0
	0	0	0
	0	0	0
	0	0	0
	0	0	0
	0	0	0
	0	0	0
	0	0	0
	0	0	0
	0	0	0
	0	0	0
	0	0	0
	0	0	0
	0	0	0
	0	0	0
	0	0	0
	0	0	0
	0	0	0
	0	0	0
	0	0	0
	0	0	0
	0	0	0
	0	0	0
	0	0	0
	0	0	0
	0	0	0
	0	0	0
	0	0	0
	0	0	0
	0	0	0
	0	0	0

	0	0	0
	0	0	0
	0	0	0
	0	0	0
	0	0	0
	0	0	0
	0	0	0
	0	0	0
	0	0	0
	0	0	0
	0	0	0
	0	0	0
	0	0	0
	0	0	0
	0	0	0
	0	0	0
	0	0	0
	0	0	0
	0	0	0
	0	0	0
	0	0	0
	0	0	0
	0	0	0
	0	0	0
	0	0	0
	0	0	0
	0	0	0
	0	0	0
	0	0	0
	0	0	0
	0	0	0
	0	0	0
	0	0	0
	0	0	0
	0	0	0
	0	0	0
	0	0	0
	0	0	0
	0	0	0
	0	0	0
	0	0	0
	0	0	0
	0	0	0
	0	0	0
	0	0	0
	0	0	0
	0	0	0

	0	0	0
	0	0	0
	0	0	0
	0	0	0
	0	0	0
	0	0	0
	0	0	0
	0	0	0
	0	0	0
	0	0	0
	0	0	0
	0	0	0
	0	0	0
	0	0	0
	0	0	0
	0	0	0
	0	0	0
	0	0	0
	0	0	0
	0	0	0
	0	0	0
	0	0	0
	0	0	0
	0	0	0
	0	0	0
	0	0	0
	0	0	0
	0	0	0
	0	0	0
	0	0	0
	0	0	0
	0	0	0
	0	0	0
	0	0	0
	0	0	0
	0	0	0
	0	0	0
	0	0	0
	0	0	0
	0	0	0
	0	0	0
	0	0	0
	0	0	0
	0	0	0
	0	0	0
	0	0	0
	0	0	0

	0	0	0
	0	0	0
	0	0	0
	0	0	0
	0	0	0
	0	0	0
	0	0	0
	0	0	0
	0	0	0
	0	0	0
	0	0	0
	0	0	0
	0	0	0
	0	0	0
	0	0	0
	0	0	0
	0	0	0
	0	0	0
	0	0	0
	0	0	0
	0	0	0
	0	0	0
	0	0	0
	0	0	0
	0	0	0
	0	0	0
	0	0	0
	0	0	0
	0	0	0
	0	0	0
	0	0	0
	0	0	0
	0	0	0
	0	0	0
	0	0	0
	0	0	0
	0	0	0
	0	0	0
	0	0	0
	0	0	0
	0	0	0
	0	0	0
	0	0	0
	0	0	0
	0	0	0
	0	0	0
	0	0	0

	0	0	0
	0	0	0
	0	0	0
	0	0	0
	0	0	0
	0	0	0
	0	0	0
	0	0	0
	0	0	0
	0	0	0
	0	0	0
	0	0	0
	0	0	0
	0	0	0
	0	0	0
	0	0	0
	0	0	0
	0	0	0
	0	0	0
	0	0	0
	0	0	0
	0	0	0
	0	0	0
	0	0	0
	0	0	0
	0	0	0
	0	0	0
	0	0	0
	0	0	0
	0	0	0
	0	0	0
	0	0	0
	0	0	0
	0	0	0
	0	0	0
	0	0	0
	0	0	0
	0	0	0
	0	0	0
	0	0	0
	0	0	0
	0	0	0
	0	0	0
	0	0	0
	0	0	0
	0	0	0
	0	0	0

	0	0	0
	0	0	0
	0	0	0
	0	0	0
	0	0	0
	0	0	0
	0	0	0
	0	0	0
	0	0	0
	0	0	0
	0	0	0
	0	0	0
	0	0	0
	0	0	0
	0	0	0
	0	0	0
	0	0	0
	0	0	0
	0	0	0
	0	0	0
	0	0	0
	0	0	0
	0	0	0
	0	0	0
	0	0	0
	0	0	0
	0	0	0
	0	0	0
	0	0	0
	0	0	0
	0	0	0
	0	0	0
	0	0	0
	0	0	0
	0	0	0
	0	0	0
	0	0	0
	0	0	0
	0	0	0
	0	0	0
	0	0	0
	0	0	0
	0	0	0
	0	0	0
	0	0	0
	0	0	0
	0	0	0

	0	0	0
	0	0	0
	0	0	0
	0	0	0
	0	0	0
	0	0	0
	0	0	0
	0	0	0
	0	0	0
	0	0	0
	0	0	0
	0	0	0
	0	0	0
	0	0	0
	0	0	0
	0	0	0
	0	0	0
	0	0	0
	0	0	0
	0	0	0
	0	0	0
	0	0	0
	0	0	0
	0	0	0
	0	0	0
	0	0	0
	0	0	0
	0	0	0
	0	0	0
	0	0	0
	0	0	0
	0	0	0
	0	0	0
	0	0	0
	0	0	0
	0	0	0
	0	0	0
	0	0	0
	0	0	0
	0	0	0
	0	0	0
	0	0	0
	0	0	0
	0	0	0
	0	0	0
	0	0	0
	0	0	0

	0	0	0
	0	0	0
	0	0	0
	0	0	0
	0	0	0
	0	0	0
	0	0	0
	0	0	0
	0	0	0
	0	0	0
	0	0	0
	0	0	0
	0	0	0
	0	0	0
	0	0	0
	0	0	0
	0	0	0
	0	0	0
	0	0	0
	0	0	0
	0	0	0
	0	0	0
	0	0	0
	0	0	0
	0	0	0
	0	0	0
	0	0	0
	0	0	0
	0	0	0
	0	0	0
	0	0	0
	0	0	0
	0	0	0
	0	0	0
	0	0	0
	0	0	0
	0	0	0
	0	0	0
	0	0	0
	0	0	0
	0	0	0
	0	0	0
	0	0	0
	0	0	0
	0	0	0
	0	0	0
	0	0	0

	0	0	0
	0	0	0
	0	0	0
	0	0	0
	0	0	0
	0	0	0
	0	0	0
	0	0	0
	0	0	0
	0	0	0
	0	0	0
	0	0	0
	0	0	0
	0	0	0
	0	0	0
	0	0	0
	0	0	0
	0	0	0
	0	0	0
	0	0	0
	0	0	0
	0	0	0
	0	0	0
	0	0	0
	0	0	0
	0	0	0
	0	0	0
	0	0	0
	0	0	0
	0	0	0
	0	0	0
	0	0	0
	0	0	0
	0	0	0
	0	0	0
	0	0	0
	0	0	0
	0	0	0
	0	0	0
	0	0	0
	0	0	0
	0	0	0
	0	0	0
	0	0	0
	0	0	0
	0	0	0
	0	0	0

	0	0	0
	0	0	0
	0	0	0
	0	0	0
	0	0	0
	0	0	0
	0	0	0
	0	0	0
	0	0	0
	0	0	0
	0	0	0
	0	0	0
	0	0	0
	0	0	0
	0	0	0
	0	0	0
	0	0	0
	0	0	0
	0	0	0
	0	0	0
	0	0	0
	0	0	0
	0	0	0
	0	0	0
	0	0	0
	0	0	0
	0	0	0
	0	0	0
	0	0	0
	0	0	0
	0	0	0
	0	0	0
	0	0	0
	0	0	0
	0	0	0
	0	0	0
	0	0	0
	0	0	0
	0	0	0
	0	0	0
	0	0	0
	0	0	0
	0	0	0
	0	0	0
	0	0	0
	0	0	0
	0	0	0

	0	0	0
	0	0	0
	0	0	0
	0	0	0
	0	0	0
	0	0	0
	0	0	0
	0	0	0
	0	0	0
	0	0	0
	0	0	0
	0	0	0
	0	0	0
	0	0	0
	0	0	0
	0	0	0
	0	0	0
	0	0	0
	0	0	0
	0	0	0
	0	0	0
	0	0	0
	0	0	0
	0	0	0
	0	0	0
	0	0	0
	0	0	0
	0	0	0
	0	0	0
	0	0	0
	0	0	0
	0	0	0
	0	0	0
	0	0	0
	0	0	0
	0	0	0
	0	0	0
	0	0	0
	0	0	0
	0	0	0
	0	0	0
	0	0	0
	0	0	0
	0	0	0
	0	0	0
	0	0	0
	0	0	0

	0	0	0
	0	0	0
	0	0	0
	0	0	0
	0	0	0
	0	0	0
	0	0	0
	0	0	0
	0	0	0
	0	0	0
	0	0	0
	0	0	0
	0	0	0
	0	0	0
	0	0	0
	0	0	0
	0	0	0
	0	0	0
	0	0	0
	0	0	0
	0	0	0
	0	0	0
	0	0	0
	0	0	0
	0	0	0
	0	0	0
	0	0	0
	0	0	0
	0	0	0
	0	0	0
	0	0	0
	0	0	0
	0	0	0
	0	0	0
	0	0	0
	0	0	0
	0	0	0
	0	0	0
	0	0	0
	0	0	0
	0	0	0
	0	0	0
	0	0	0
	0	0	0
	0	0	0
	0	0	0
	0	0	0

	0	0	0
	0	0	0
	0	0	0
	0	0	0
	0	0	0
	0	0	0
	0	0	0
	0	0	0
	0	0	0
	0	0	0
	0	0	0
	0	0	0
	0	0	0
	0	0	0
	0	0	0
	0	0	0
	0	0	0
	0	0	0
	0	0	0
	0	0	0
	0	0	0
	0	0	0
	0	0	0
	0	0	0
	0	0	0
	0	0	0
	0	0	0
	0	0	0
	0	0	0
	0	0	0
	0	0	0
	0	0	0
	0	0	0
	0	0	0
	0	0	0
	0	0	0
	0	0	0
	0	0	0
	0	0	0
	0	0	0
	0	0	0
	0	0	0
	0	0	0
	0	0	0
	0	0	0
	0	0	0
	0	0	0

	0	0	0
	0	0	0
	0	0	0
	0	0	0
	0	0	0
	0	0	0
	0	0	0
	0	0	0
	0	0	0
	0	0	0
	0	0	0
	0	0	0
	0	0	0
	0	0	0
	0	0	0
	0	0	0
	0	0	0
	0	0	0
	0	0	0
	0	0	0
	0	0	0
	0	0	0
	0	0	0
	0	0	0
	0	0	0
	0	0	0
	0	0	0
	0	0	0
	0	0	0
	0	0	0
	0	0	0
	0	0	0
	0	0	0
	0	0	0
	0	0	0
	0	0	0
	0	0	0
	0	0	0
	0	0	0
	0	0	0
	0	0	0
	0	0	0
	0	0	0
	0	0	0
	0	0	0
	0	0	0
	0	0	0

	0	0	0
	0	0	0
	0	0	0
	0	0	0
	0	0	0
	0	0	0
	0	0	0
	0	0	0
	0	0	0
	0	0	0
	0	0	0
	0	0	0
	0	0	0
	0	0	0
	0	0	0
	0	0	0
	0	0	0
	0	0	0
	0	0	0
	0	0	0
	0	0	0
	0	0	0
	0	0	0
	0	0	0
	0	0	0
	0	0	0
	0	0	0
	0	0	0
	0	0	0
	0	0	0
	0	0	0
	0	0	0
	0	0	0
	0	0	0
	0	0	0
	0	0	0
	0	0	0
	0	0	0
	0	0	0
	0	0	0
	0	0	0
	0	0	0
	0	0	0
	0	0	0
	0	0	0
	0	0	0
	0	0	0

	0	0	0
	0	0	0
	0	0	0
	0	0	0
	0	0	0
	0	0	0
	0	0	0
	0	0	0
	0	0	0
	0	0	0
	0	0	0
	0	0	0
	0	0	0
	0	0	0
	0	0	0
	0	0	0
	0	0	0
	0	0	0
	0	0	0
	0	0	0
	0	0	0
	0	0	0
	0	0	0
	0	0	0
	0	0	0
	0	0	0
	0	0	0
	0	0	0
	0	0	0
	0	0	0
	0	0	0
	0	0	0
	0	0	0
	0	0	0
	0	0	0
	0	0	0
	0	0	0
	0	0	0
	0	0	0
	0	0	0
	0	0	0
	0	0	0
	0	0	0
	0	0	0
	0	0	0
	0	0	0
	0	0	0

	0	0	0
	0	0	0
	0	0	0
	0	0	0
	0	0	0
	0	0	0
	0	0	0
	0	0	0
	0	0	0
	0	0	0
	0	0	0
	0	0	0
	0	0	0
	0	0	0
	0	0	0
	0	0	0
	0	0	0
	0	0	0
	0	0	0
	0	0	0
	0	0	0
	0	0	0
	0	0	0
	0	0	0
	0	0	0
	0	0	0
	0	0	0
	0	0	0
	0	0	0
	0	0	0
	0	0	0
	0	0	0
	0	0	0
	0	0	0
	0	0	0
	0	0	0
	0	0	0
	0	0	0
	0	0	0
	0	0	0
	0	0	0
	0	0	0
	0	0	0
	0	0	0
	0	0	0
	0	0	0
	0	0	0

	0	0	0
	0	0	0
	0	0	0
	0	0	0
	0	0	0
	0	0	0
	0	0	0
	0	0	0
	0	0	0
	0	0	0
	0	0	0
	0	0	0
	0	0	0
	0	0	0
	0	0	0
	0	0	0
	0	0	0
	0	0	0
	0	0	0
	0	0	0
	0	0	0
	0	0	0
	0	0	0
	0	0	0
	0	0	0
	0	0	0
	0	0	0
	0	0	0
	0	0	0
	0	0	0
	0	0	0
	0	0	0
	0	0	0
	0	0	0
	0	0	0
	0	0	0
	0	0	0
	0	0	0
	0	0	0
	0	0	0
	0	0	0
	0	0	0
	0	0	0
	0	0	0
	0	0	0
	0	0	0
	0	0	0

	0	0	0
	0	0	0
	0	0	0
	0	0	0
	0	0	0
	0	0	0
	0	0	0
	0	0	0
	0	0	0
	0	0	0
	0	0	0
	0	0	0
	0	0	0
	0	0	0
	0	0	0
	0	0	0
	0	0	0
	0	0	0
	0	0	0
	0	0	0
	0	0	0
	0	0	0
	0	0	0
	0	0	0
	0	0	0
	0	0	0
	0	0	0
	0	0	0
	0	0	0
	0	0	0
	0	0	0
	0	0	0
	0	0	0
	0	0	0
	0	0	0
	0	0	0
	0	0	0
	0	0	0
	0	0	0
	0	0	0
	0	0	0
	0	0	0
	0	0	0
	0	0	0
	0	0	0
	0	0	0
	0	0	0

	0	0	0
	0	0	0
	0	0	0
	0	0	0
	0	0	0
	0	0	0
	0	0	0
	0	0	0
	0	0	0
	0	0	0
	0	0	0
	0	0	0
	0	0	0
	0	0	0
	0	0	0
	0	0	0
	0	0	0
	0	0	0
	0	0	0
	0	0	0
	0	0	0
	0	0	0
	0	0	0
	0	0	0
	0	0	0
	0	0	0
	0	0	0
	0	0	0
	0	0	0
	0	0	0
	0	0	0
	0	0	0
	0	0	0
	0	0	0
	0	0	0
	0	0	0
	0	0	0
	0	0	0
	0	0	0
	0	0	0
	0	0	0
	0	0	0
	0	0	0
	0	0	0
	0	0	0
	0	0	0
	0	0	0

	0	0	0
	0	0	0
	0	0	0
	0	0	0
	0	0	0
	0	0	0
	0	0	0
	0	0	0
	0	0	0
	0	0	0
	0	0	0
	0	0	0
	0	0	0
	0	0	0
	0	0	0
	0	0	0
	0	0	0
	0	0	0
	0	0	0
	0	0	0
	0	0	0
	0	0	0
	0	0	0
	0	0	0
	0	0	0
	0	0	0
	0	0	0
	0	0	0
	0	0	0
	0	0	0
	0	0	0
	0	0	0
	0	0	0
	0	0	0
	0	0	0
	0	0	0
	0	0	0
	0	0	0
	0	0	0
	0	0	0
	0	0	0
	0	0	0
	0	0	0
	0	0	0
	0	0	0
	0	0	0
	0	0	0

	0	0	0
	0	0	0
	0	0	0
	0	0	0
	0	0	0
	0	0	0
	0	0	0
	0	0	0
	0	0	0
	0	0	0
	0	0	0
	0	0	0
	0	0	0
	0	0	0
	0	0	0
	0	0	0
	0	0	0
	0	0	0
	0	0	0
	0	0	0
	0	0	0
	0	0	0
	0	0	0
	0	0	0
	0	0	0
	0	0	0
	0	0	0
	0	0	0
	0	0	0
	0	0	0
	0	0	0
	0	0	0
	0	0	0
	0	0	0
	0	0	0
	0	0	0
	0	0	0
	0	0	0
	0	0	0
	0	0	0
	0	0	0
	0	0	0
	0	0	0
	0	0	0
	0	0	0
	0	0	0
	0	0	0

	0	0	0
	0	0	0
	0	0	0
	0	0	0
	0	0	0
	0	0	0
	0	0	0
	0	0	0
	0	0	0
	0	0	0
	0	0	0
	0	0	0
	0	0	0
	0	0	0
	0	0	0
	0	0	0
	0	0	0
	0	0	0
	0	0	0
	0	0	0
	0	0	0
	0	0	0
	0	0	0
	0	0	0
	0	0	0
	0	0	0
	0	0	0
	0	0	0
	0	0	0
	0	0	0
	0	0	0
	0	0	0
	0	0	0
	0	0	0
	0	0	0
	0	0	0
	0	0	0
	0	0	0
	0	0	0
	0	0	0
	0	0	0
	0	0	0
	0	0	0
	0	0	0
	0	0	0
	0	0	0
	0	0	0

	0	0	0
	0	0	0
	0	0	0
	0	0	0
	0	0	0
	0	0	0
	0	0	0
	0	0	0
	0	0	0
	0	0	0
	0	0	0
	0	0	0
	0	0	0
	0	0	0
	0	0	0
	0	0	0
	0	0	0
	0	0	0
	0	0	0
	0	0	0
	0	0	0
	0	0	0
	0	0	0
	0	0	0
	0	0	0
	0	0	0
	0	0	0
	0	0	0
	0	0	0
	0	0	0
	0	0	0
	0	0	0
	0	0	0
	0	0	0
	0	0	0
	0	0	0
	0	0	0
	0	0	0
	0	0	0
	0	0	0
	0	0	0
	0	0	0
	0	0	0
	0	0	0
	0	0	0
	0	0	0
	0	0	0

	0	0	0
	0	0	0
	0	0	0
	0	0	0
	0	0	0
	0	0	0
	0	0	0
	0	0	0
	0	0	0
	0	0	0
	0	0	0
	0	0	0
	0	0	0
	0	0	0
	0	0	0
	0	0	0
	0	0	0
	0	0	0
	0	0	0
	0	0	0
	0	0	0
	0	0	0
	0	0	0
	0	0	0
	0	0	0
	0	0	0
	0	0	0
	0	0	0
	0	0	0
	0	0	0
	0	0	0
	0	0	0
	0	0	0
	0	0	0
	0	0	0
	0	0	0
	0	0	0
	0	0	0
	0	0	0
	0	0	0
	0	0	0
	0	0	0
	0	0	0
	0	0	0
	0	0	0
	0	0	0
	0	0	0

	0	0	0
	0	0	0
	0	0	0
	0	0	0
	0	0	0
	0	0	0
	0	0	0
	0	0	0
	0	0	0
	0	0	0
	0	0	0
	0	0	0
	0	0	0
	0	0	0
	0	0	0
	0	0	0
	0	0	0
	0	0	0
	0	0	0
	0	0	0
	0	0	0
	0	0	0
	0	0	0
	0	0	0
	0	0	0
	0	0	0
	0	0	0
	0	0	0
	0	0	0
	0	0	0
	0	0	0
	0	0	0
	0	0	0
	0	0	0
	0	0	0
	0	0	0
	0	0	0
	0	0	0
	0	0	0
	0	0	0
	0	0	0
	0	0	0
	0	0	0
	0	0	0
	0	0	0
	0	0	0
	0	0	0

	0	0	0
	0	0	0
	0	0	0
	0	0	0
	0	0	0
	0	0	0
	0	0	0
	0	0	0
	0	0	0
	0	0	0
	0	0	0
	0	0	0
	0	0	0
	0	0	0
	0	0	0
	0	0	0
	0	0	0
	0	0	0
	0	0	0
	0	0	0
	0	0	0
	0	0	0
	0	0	0
	0	0	0
	0	0	0
	0	0	0
	0	0	0
	0	0	0
	0	0	0
	0	0	0
	0	0	0
	0	0	0
	0	0	0
	0	0	0
	0	0	0
	0	0	0
	0	0	0
	0	0	0
	0	0	0
	0	0	0
	0	0	0
	0	0	0
	0	0	0
	0	0	0
	0	0	0
	0	0	0
	0	0	0

	0	0	0
	0	0	0
	0	0	0
	0	0	0
	0	0	0
	0	0	0
	0	0	0
	0	0	0
	0	0	0
	0	0	0
	0	0	0
	0	0	0
	0	0	0
	0	0	0
	0	0	0
	0	0	0
	0	0	0
	0	0	0
	0	0	0
	0	0	0
	0	0	0
	0	0	0
	0	0	0
	0	0	0
	0	0	0
	0	0	0
	0	0	0
	0	0	0
	0	0	0
	0	0	0
	0	0	0
	0	0	0
	0	0	0
	0	0	0
	0	0	0
	0	0	0
	0	0	0
	0	0	0
	0	0	0
	0	0	0
	0	0	0
	0	0	0
	0	0	0
	0	0	0
	0	0	0
	0	0	0
	0	0	0

	0	0	0
	0	0	0
	0	0	0
	0	0	0
	0	0	0
	0	0	0
	0	0	0
	0	0	0
	0	0	0
	0	0	0
	0	0	0
	0	0	0
	0	0	0
	0	0	0
	0	0	0
	0	0	0
	0	0	0
	0	0	0
	0	0	0
	0	0	0
	0	0	0
	0	0	0
	0	0	0
	0	0	0
	0	0	0
	0	0	0
	0	0	0
	0	0	0
	0	0	0
	0	0	0
	0	0	0
	0	0	0
	0	0	0
	0	0	0
	0	0	0
	0	0	0
	0	0	0
	0	0	0
	0	0	0
	0	0	0
	0	0	0
	0	0	0
	0	0	0
	0	0	0
	0	0	0
	0	0	0
	0	0	0

	0	0	0
	0	0	0
	0	0	0
	0	0	0
	0	0	0
	0	0	0
	0	0	0
	0	0	0
	0	0	0
	0	0	0
	0	0	0
	0	0	0
	0	0	0
	0	0	0
	0	0	0
	0	0	0
	0	0	0
	0	0	0
	0	0	0
	0	0	0
	0	0	0
	0	0	0
	0	0	0
	0	0	0
	0	0	0
	0	0	0
	0	0	0
	0	0	0
	0	0	0
	0	0	0
	0	0	0
	0	0	0
	0	0	0
	0	0	0
	0	0	0
	0	0	0
	0	0	0
	0	0	0
	0	0	0
	0	0	0
	0	0	0
	0	0	0
	0	0	0
	0	0	0
	0	0	0
	0	0	0
	0	0	0

	0	0	0
	0	0	0
	0	0	0
	0	0	0
	0	0	0
	0	0	0
	0	0	0
	0	0	0
	0	0	0
	0	0	0
	0	0	0
	0	0	0
	0	0	0
	0	0	0
	0	0	0
	0	0	0
	0	0	0
	0	0	0
	0	0	0
	0	0	0
	0	0	0
	0	0	0
	0	0	0
	0	0	0
	0	0	0
	0	0	0
	0	0	0
	0	0	0
	0	0	0
	0	0	0
	0	0	0
	0	0	0
	0	0	0
	0	0	0
	0	0	0
	0	0	0
	0	0	0
	0	0	0
	0	0	0
	0	0	0
	0	0	0
	0	0	0
	0	0	0
	0	0	0
	0	0	0
	0	0	0
	0	0	0

0	0	0
0	0	0
0	0	0
0	0	0
0	0	0
0	0	0
0	0	0
0	0	0
0	0	0
0	0	0
0	0	0
0	0	0
0	0	0
0	0	0
0	0	0
0	0	0
0	0	0
0	0	0
0	0	0
0	0	0
0	0	0
0	0	0
0	0	0
0	0	0
0	0	0
0	0	0
0	0	0
0	0	0
0	0	0
0	0	0
0	0	0
0	0	0
0	0	0
0	0	0
0	0	0
0	0	0
0	0	0
0	0	0
0	0	0
0	0	0
0	0	0
0	0	0
0	0	0
0	0	0
0	0	0
0	0	0
0	0	0

	0	0	0
	0	0	0
	0	0	0
	0	0	0
	0	0	0
	0	0	0
	0	0	0
	0	0	0
	0	0	0
	0	0	0
	0	0	0
	0	0	0
	0	0	0
	0	0	0
	0	0	0
	0	0	0
	0	0	0
	0	0	0
	0	0	0
	0	0	0
	0	0	0
	0	0	0
	0	0	0
	0	0	0
	0	0	0
	0	0	0
	0	0	0
	0	0	0
	0	0	0
	0	0	0
	0	0	0
	0	0	0
	0	0	0
	0	0	0
	0	0	0
	0	0	0
	0	0	0
	0	0	0
	0	0	0
	0	0	0
	0	0	0
	0	0	0
	0	0	0
	0	0	0
	0	0	0
	0	0	0
	0	0	0

	0	0	0
	0	0	0
	0	0	0
	0	0	0
	0	0	0
	0	0	0
	0	0	0
	0	0	0
	0	0	0
	0	0	0
	0	0	0
	0	0	0
	0	0	0
	0	0	0
	0	0	0
	0	0	0
	0	0	0
	0	0	0
	0	0	0
	0	0	0
	0	0	0
	0	0	0
	0	0	0
	0	0	0
	0	0	0
	0	0	0
	0	0	0
	0	0	0
	0	0	0
	0	0	0
	0	0	0
	0	0	0
	0	0	0
	0	0	0
	0	0	0
	0	0	0
	0	0	0
	0	0	0
	0	0	0
	0	0	0
	0	0	0
	0	0	0
	0	0	0
	0	0	0
	0	0	0
	0	0	0
	0	0	0

	0	0	0
	0	0	0
	0	0	0
	0	0	0
	0	0	0
	0	0	0
	0	0	0
	0	0	0
	0	0	0
	0	0	0
	0	0	0
	0	0	0
	0	0	0
	0	0	0
	0	0	0
	0	0	0
	0	0	0
	0	0	0
	0	0	0
	0	0	0
	0	0	0
	0	0	0
	0	0	0
	0	0	0
	0	0	0
	0	0	0
	0	0	0
	0	0	0
	0	0	0
	0	0	0
	0	0	0
	0	0	0
	0	0	0
	0	0	0
	0	0	0
	0	0	0
	0	0	0
	0	0	0
	0	0	0
	0	0	0
	0	0	0
	0	0	0
	0	0	0
	0	0	0
	0	0	0
	0	0	0
	0	0	0

	0	0	0
	0	0	0
	0	0	0
	0	0	0
	0	0	0
	0	0	0
	0	0	0
	0	0	0
	0	0	0
	0	0	0
	0	0	0
	0	0	0
	0	0	0
	0	0	0
	0	0	0
	0	0	0
	0	0	0
	0	0	0
	0	0	0
	0	0	0
	0	0	0
	0	0	0
	0	0	0
	0	0	0
	0	0	0
	0	0	0
	0	0	0
	0	0	0
	0	0	0
	0	0	0
	0	0	0
	0	0	0
	0	0	0
	0	0	0
	0	0	0
	0	0	0
	0	0	0
	0	0	0
	0	0	0
	0	0	0
	0	0	0
	0	0	0
	0	0	0
	0	0	0
	0	0	0
	0	0	0
	0	0	0

	0	0	0
	0	0	0
	0	0	0
	0	0	0
	0	0	0
	0	0	0
	0	0	0
	0	0	0
	0	0	0
	0	0	0
	0	0	0
	0	0	0
	0	0	0
	0	0	0
	0	0	0
	0	0	0
	0	0	0
	0	0	0
	0	0	0
	0	0	0
	0	0	0
	0	0	0
	0	0	0
	0	0	0
	0	0	0
	0	0	0
	0	0	0
	0	0	0
	0	0	0
	0	0	0
	0	0	0
	0	0	0
	0	0	0
	0	0	0
	0	0	0
	0	0	0
	0	0	0
	0	0	0
	0	0	0
	0	0	0
	0	0	0
	0	0	0
	0	0	0
	0	0	0
	0	0	0
	0	0	0
	0	0	0

	0	0	0
	0	0	0
	0	0	0
	0	0	0
	0	0	0
	0	0	0
	0	0	0
	0	0	0
	0	0	0
	0	0	0
	0	0	0
	0	0	0
	0	0	0
	0	0	0
	0	0	0
	0	0	0
	0	0	0
	0	0	0
	0	0	0
	0	0	0
	0	0	0
	0	0	0
	0	0	0
	0	0	0
	0	0	0
	0	0	0
	0	0	0
	0	0	0
	0	0	0
	0	0	0
	0	0	0
	0	0	0
	0	0	0
	0	0	0
	0	0	0
	0	0	0
	0	0	0
	0	0	0
	0	0	0
	0	0	0
	0	0	0
	0	0	0
	0	0	0
	0	0	0
	0	0	0
	0	0	0
	0	0	0

	0	0	0
	0	0	0
	0	0	0
	0	0	0
	0	0	0
	0	0	0
	0	0	0
	0	0	0
	0	0	0
	0	0	0
	0	0	0
	0	0	0
	0	0	0
	0	0	0
	0	0	0
	0	0	0
	0	0	0
	0	0	0
	0	0	0
	0	0	0
	0	0	0
	0	0	0
	0	0	0
	0	0	0
	0	0	0
	0	0	0
	0	0	0
	0	0	0
	0	0	0
	0	0	0
	0	0	0
	0	0	0
	0	0	0
	0	0	0
	0	0	0
	0	0	0
	0	0	0
	0	0	0
	0	0	0
	0	0	0
	0	0	0
	0	0	0
	0	0	0
	0	0	0
	0	0	0
	0	0	0
	0	0	0

0	0	0
0	0	0
0	0	0
0	0	0
0	0	0
0	0	0
0	0	0
0	0	0
0	0	0
0	0	0
0	0	0
0	0	0
0	0	0
0	0	0
0	0	0
0	0	0
0	0	0
0	0	0
0	0	0
0	0	0
0	0	0
0	0	0
0	0	0
0	0	0
0	0	0
0	0	0
0	0	0
0	0	0
0	0	0
0	0	0
0	0	0
0	0	0
0	0	0
0	0	0
0	0	0
0	0	0
0	0	0
0	0	0
0	0	0
0	0	0
0	0	0
0	0	0
0	0	0
0	0	0
0	0	0
0	0	0
0	0	0

	0	0	0
	0	0	0
	0	0	0
	0	0	0
	0	0	0
	0	0	0
	0	0	0
	0	0	0
	0	0	0
	0	0	0
	0	0	0
	0	0	0
	0	0	0
	0	0	0
	0	0	0
	0	0	0
	0	0	0
	0	0	0
	0	0	0
	0	0	0
	0	0	0
	0	0	0
	0	0	0
	0	0	0
	0	0	0
	0	0	0
	0	0	0
	0	0	0
	0	0	0
	0	0	0
	0	0	0
	0	0	0
	0	0	0
	0	0	0
	0	0	0
	0	0	0
	0	0	0
	0	0	0
	0	0	0
	0	0	0
	0	0	0
	0	0	0
	0	0	0
	0	0	0
	0	0	0
	0	0	0
	0	0	0

	0	0	0
	0	0	0
	0	0	0
	0	0	0
	0	0	0
	0	0	0
	0	0	0
	0	0	0
	0	0	0
	0	0	0
	0	0	0
	0	0	0
	0	0	0
	0	0	0
	0	0	0
	0	0	0
	0	0	0
	0	0	0
	0	0	0
	0	0	0
	0	0	0
	0	0	0
	0	0	0
	0	0	0
	0	0	0
	0	0	0
	0	0	0
	0	0	0
	0	0	0
	0	0	0
	0	0	0
	0	0	0
	0	0	0
	0	0	0
	0	0	0
	0	0	0
	0	0	0
	0	0	0
	0	0	0
	0	0	0
	0	0	0
	0	0	0
	0	0	0
	0	0	0
	0	0	0
	0	0	0
	0	0	0

	0	0	0
	0	0	0
	0	0	0
	0	0	0
	0	0	0
	0	0	0
	0	0	0
	0	0	0
	0	0	0
	0	0	0
	0	0	0
	0	0	0
	0	0	0
	0	0	0
	0	0	0
	0	0	0
	0	0	0
	0	0	0
	0	0	0
	0	0	0
	0	0	0
	0	0	0
	0	0	0
	0	0	0
	0	0	0
	0	0	0
	0	0	0
	0	0	0
	0	0	0
	0	0	0
	0	0	0
	0	0	0
	0	0	0
	0	0	0
	0	0	0
	0	0	0
	0	0	0
	0	0	0
	0	0	0
	0	0	0
	0	0	0
	0	0	0
	0	0	0
	0	0	0
	0	0	0
	0	0	0
	0	0	0

	0	0	0
	0	0	0
	0	0	0
	0	0	0
	0	0	0
	0	0	0
	0	0	0
	0	0	0
	0	0	0
	0	0	0
	0	0	0
	0	0	0
	0	0	0
	0	0	0
	0	0	0
	0	0	0
	0	0	0
	0	0	0
	0	0	0
	0	0	0
	0	0	0
	0	0	0
	0	0	0
	0	0	0
	0	0	0
	0	0	0
	0	0	0
	0	0	0
	0	0	0
	0	0	0
	0	0	0
	0	0	0
	0	0	0
	0	0	0
	0	0	0
	0	0	0
	0	0	0
	0	0	0
	0	0	0
	0	0	0
	0	0	0
	0	0	0
	0	0	0
	0	0	0
	0	0	0
	0	0	0
	0	0	0

	0	0	0
	0	0	0
	0	0	0
	0	0	0
	0	0	0
	0	0	0
	0	0	0
	0	0	0
	0	0	0
	0	0	0
	0	0	0
	0	0	0
	0	0	0
	0	0	0
	0	0	0
	0	0	0
	0	0	0
	0	0	0
	0	0	0
	0	0	0
	0	0	0
	0	0	0
	0	0	0
	0	0	0
	0	0	0
	0	0	0
	0	0	0
	0	0	0
	0	0	0
	0	0	0
	0	0	0
	0	0	0
	0	0	0
	0	0	0
	0	0	0
	0	0	0
	0	0	0
	0	0	0
	0	0	0
	0	0	0
	0	0	0
	0	0	0
	0	0	0
	0	0	0
	0	0	0
	0	0	0
	0	0	0

	0	0	0
	0	0	0
	0	0	0
	0	0	0
	0	0	0
	0	0	0
	0	0	0
	0	0	0
	0	0	0
	0	0	0
	0	0	0
	0	0	0
	0	0	0
	0	0	0
	0	0	0
	0	0	0
	0	0	0
	0	0	0
	0	0	0
	0	0	0
	0	0	0
	0	0	0
	0	0	0
	0	0	0
	0	0	0
	0	0	0
	0	0	0
	0	0	0
	0	0	0
	0	0	0
	0	0	0
	0	0	0
	0	0	0
	0	0	0
	0	0	0
	0	0	0
	0	0	0
	0	0	0
	0	0	0
	0	0	0
	0	0	0
	0	0	0
	0	0	0
	0	0	0
	0	0	0
	0	0	0
	0	0	0

	0	0	0
	0	0	0
	0	0	0
	0	0	0
	0	0	0
	0	0	0
	0	0	0
	0	0	0
	0	0	0
	0	0	0
	0	0	0
	0	0	0
	0	0	0
	0	0	0
	0	0	0
	0	0	0
	0	0	0
	0	0	0
	0	0	0
	0	0	0
	0	0	0
	0	0	0
	0	0	0
	0	0	0
	0	0	0
	0	0	0
	0	0	0
	0	0	0
	0	0	0
	0	0	0
	0	0	0
	0	0	0
	0	0	0
	0	0	0
	0	0	0
	0	0	0
	0	0	0
	0	0	0
	0	0	0
	0	0	0
	0	0	0
	0	0	0
	0	0	0
	0	0	0
	0	0	0
	0	0	0
	0	0	0

	0	0	0
	0	0	0
	0	0	0
	0	0	0
	0	0	0
	0	0	0
	0	0	0
	0	0	0
	0	0	0
	0	0	0
	0	0	0
	0	0	0
	0	0	0
	0	0	0
	0	0	0
	0	0	0
	0	0	0
	0	0	0
	0	0	0
	0	0	0
	0	0	0
	0	0	0
	0	0	0
	0	0	0
	0	0	0
	0	0	0
	0	0	0
	0	0	0
	0	0	0
	0	0	0
	0	0	0
	0	0	0
	0	0	0
	0	0	0
	0	0	0
	0	0	0
	0	0	0
	0	0	0
	0	0	0
	0	0	0
	0	0	0
	0	0	0
	0	0	0
	0	0	0
	0	0	0
	0	0	0
	0	0	0

	0	0	0
	0	0	0
	0	0	0
	0	0	0
	0	0	0
	0	0	0
	0	0	0
	0	0	0
	0	0	0
	0	0	0
	0	0	0
	0	0	0
	0	0	0
	0	0	0
	0	0	0
	0	0	0
	0	0	0
	0	0	0
	0	0	0
	0	0	0
	0	0	0
	0	0	0
	0	0	0
	0	0	0
	0	0	0
	0	0	0
	0	0	0
	0	0	0
	0	0	0
	0	0	0
	0	0	0
	0	0	0
	0	0	0
	0	0	0
	0	0	0
	0	0	0
	0	0	0
	0	0	0
	0	0	0
	0	0	0
	0	0	0
	0	0	0
	0	0	0
	0	0	0
	0	0	0
	0	0	0
	0	0	0

	0	0	0
	0	0	0
	0	0	0
	0	0	0
	0	0	0
	0	0	0
	0	0	0
	0	0	0
	0	0	0
	0	0	0
	0	0	0
	0	0	0
	0	0	0
	0	0	0
	0	0	0
	0	0	0
	0	0	0
	0	0	0
	0	0	0
	0	0	0
	0	0	0
	0	0	0
	0	0	0
	0	0	0
	0	0	0
	0	0	0
	0	0	0
	0	0	0
	0	0	0
	0	0	0
	0	0	0
	0	0	0
	0	0	0
	0	0	0
	0	0	0
	0	0	0
	0	0	0
	0	0	0
	0	0	0
	0	0	0
	0	0	0
	0	0	0
	0	0	0
	0	0	0
	0	0	0
	0	0	0
	0	0	0

0	0	0
0	0	0
0	0	0
0	0	0
0	0	0
0	0	0
0	0	0
0	0	0
0	0	0
0	0	0
0	0	0
0	0	0
0	0	0
0	0	0
0	0	0
0	0	0
0	0	0
0	0	0
0	0	0
0	0	0
0	0	0
0	0	0
0	0	0
0	0	0
0	0	0
0	0	0
0	0	0
0	0	0
0	0	0
0	0	0
0	0	0
0	0	0
0	0	0
0	0	0
0	0	0
0	0	0
0	0	0
0	0	0
0	0	0
0	0	0
0	0	0
0	0	0
0	0	0
0	0	0
0	0	0
0	0	0
0	0	0

	0	0	0
	0	0	0
	0	0	0
	0	0	0
	0	0	0
	0	0	0
	0	0	0
	0	0	0
	0	0	0
	0	0	0
	0	0	0
	0	0	0
	0	0	0
	0	0	0
	0	0	0
	0	0	0
	0	0	0
	0	0	0
	0	0	0
	0	0	0
	0	0	0
	0	0	0
	0	0	0
	0	0	0
	0	0	0
	0	0	0
	0	0	0
	0	0	0
	0	0	0
	0	0	0
	0	0	0
	0	0	0
	0	0	0
	0	0	0
	0	0	0
	0	0	0
	0	0	0
	0	0	0
	0	0	0
	0	0	0
	0	0	0
	0	0	0
	0	0	0
	0	0	0
	0	0	0
	0	0	0
	0	0	0

	0	0	0
	0	0	0
	0	0	0
	0	0	0
	0	0	0
	0	0	0
	0	0	0
	0	0	0
	0	0	0
	0	0	0
	0	0	0
	0	0	0
	0	0	0
	0	0	0
	0	0	0
	0	0	0
	0	0	0
	0	0	0
	0	0	0
	0	0	0
	0	0	0
	0	0	0
	0	0	0
	0	0	0
	0	0	0
	0	0	0
	0	0	0
	0	0	0
	0	0	0
	0	0	0
	0	0	0
	0	0	0
	0	0	0
	0	0	0
	0	0	0
	0	0	0
	0	0	0
	0	0	0
	0	0	0
	0	0	0
	0	0	0
	0	0	0
	0	0	0
	0	0	0
	0	0	0
	0	0	0
	0	0	0

0	0	0
0	0	0
0	0	0
0	0	0
0	0	0
0	0	0
0	0	0
0	0	0
0	0	0
0	0	0
0	0	0
0	0	0
0	0	0
0	0	0
0	0	0
0	0	0
0	0	0
0	0	0
0	0	0
0	0	0
0	0	0
0	0	0
0	0	0
0	0	0
0	0	0
0	0	0
0	0	0
0	0	0
0	0	0
0	0	0
0	0	0
0	0	0
0	0	0
0	0	0
0	0	0
0	0	0
0	0	0
0	0	0
0	0	0
0	0	0
0	0	0
0	0	0
0	0	0
0	0	0
0	0	0
0	0	0
0	0	0

	0	0	0
	0	0	0
	0	0	0
	0	0	0
	0	0	0
	0	0	0
	0	0	0
	0	0	0
	0	0	0
	0	0	0
	0	0	0
	0	0	0
	0	0	0
	0	0	0
	0	0	0
	0	0	0
	0	0	0
	0	0	0
	0	0	0
	0	0	0
	0	0	0
	0	0	0
	0	0	0
	0	0	0
	0	0	0
	0	0	0
	0	0	0
	0	0	0
	0	0	0
	0	0	0
	0	0	0
	0	0	0
	0	0	0
	0	0	0
	0	0	0
	0	0	0
	0	0	0
	0	0	0
	0	0	0
	0	0	0
	0	0	0
	0	0	0
	0	0	0
	0	0	0
	0	0	0
	0	0	0
	0	0	0

	0	0	0
	0	0	0
	0	0	0
	0	0	0
	0	0	0
	0	0	0
	0	0	0
	0	0	0
	0	0	0
	0	0	0
	0	0	0
	0	0	0
	0	0	0
	0	0	0
	0	0	0
	0	0	0
	0	0	0
	0	0	0
	0	0	0
	0	0	0
	0	0	0
	0	0	0
	0	0	0
	0	0	0
	0	0	0
	0	0	0
	0	0	0
	0	0	0
	0	0	0
	0	0	0
	0	0	0
	0	0	0
	0	0	0
	0	0	0
	0	0	0
	0	0	0
	0	0	0
	0	0	0
	0	0	0
	0	0	0
	0	0	0
	0	0	0
	0	0	0
	0	0	0
	0	0	0
	0	0	0
	0	0	0

	0	0	0
	0	0	0
	0	0	0
	0	0	0
	0	0	0
	0	0	0
	0	0	0
	0	0	0
	0	0	0
	0	0	0
	0	0	0
	0	0	0
	0	0	0
	0	0	0
	0	0	0
	0	0	0
	0	0	0
	0	0	0
	0	0	0
	0	0	0
	0	0	0
	0	0	0
	0	0	0
	0	0	0
	0	0	0
	0	0	0
	0	0	0
	0	0	0
	0	0	0
	0	0	0
	0	0	0
	0	0	0
	0	0	0
	0	0	0
	0	0	0
	0	0	0
	0	0	0
	0	0	0
	0	0	0
	0	0	0
	0	0	0
	0	0	0
	0	0	0
	0	0	0
	0	0	0
	0	0	0
	0	0	0

	0	0	0
	0	0	0
	0	0	0
	0	0	0
	0	0	0
	0	0	0
	0	0	0
	0	0	0
	0	0	0
	0	0	0
	0	0	0
	0	0	0
	0	0	0
	0	0	0
	0	0	0
	0	0	0
	0	0	0
	0	0	0
	0	0	0
	0	0	0
	0	0	0
	0	0	0
	0	0	0
	0	0	0
	0	0	0
	0	0	0
	0	0	0
	0	0	0
	0	0	0
	0	0	0
	0	0	0
	0	0	0
	0	0	0
	0	0	0
	0	0	0
	0	0	0
	0	0	0
	0	0	0
	0	0	0
	0	0	0
	0	0	0
	0	0	0
	0	0	0
	0	0	0
	0	0	0
	0	0	0
	0	0	0

	0	0	0
	0	0	0
	0	0	0
	0	0	0
	0	0	0
	0	0	0
	0	0	0
	0	0	0
	0	0	0
	0	0	0
	0	0	0
	0	0	0
	0	0	0
	0	0	0
	0	0	0
	0	0	0
	0	0	0
	0	0	0
	0	0	0
	0	0	0
	0	0	0
	0	0	0
	0	0	0
	0	0	0
	0	0	0
	0	0	0
	0	0	0
	0	0	0
	0	0	0
	0	0	0
	0	0	0
	0	0	0
	0	0	0
	0	0	0
	0	0	0
	0	0	0
	0	0	0
	0	0	0
	0	0	0
	0	0	0
	0	0	0
	0	0	0
	0	0	0
	0	0	0
	0	0	0
	0	0	0
	0	0	0

	0	0	0
	0	0	0
	0	0	0
	0	0	0
	0	0	0
	0	0	0
	0	0	0
	0	0	0
	0	0	0
	0	0	0
	0	0	0
	0	0	0
	0	0	0
	0	0	0
	0	0	0
	0	0	0
	0	0	0
	0	0	0
	0	0	0
	0	0	0
	0	0	0
	0	0	0
	0	0	0
	0	0	0
	0	0	0
	0	0	0
	0	0	0
	0	0	0
	0	0	0
	0	0	0
	0	0	0
	0	0	0
	0	0	0
	0	0	0
	0	0	0
	0	0	0
	0	0	0
	0	0	0
	0	0	0
	0	0	0
	0	0	0
	0	0	0
	0	0	0
	0	0	0
	0	0	0
	0	0	0
	0	0	0

	0	0	0
	0	0	0
	0	0	0
	0	0	0
	0	0	0
	0	0	0
	0	0	0
	0	0	0
	0	0	0
	0	0	0
	0	0	0
	0	0	0
	0	0	0
	0	0	0
	0	0	0
	0	0	0
	0	0	0
	0	0	0
	0	0	0
	0	0	0
	0	0	0
	0	0	0
	0	0	0
	0	0	0
	0	0	0
	0	0	0
	0	0	0
	0	0	0
	0	0	0
	0	0	0
	0	0	0
	0	0	0
	0	0	0
	0	0	0
	0	0	0
	0	0	0
	0	0	0
	0	0	0
	0	0	0
	0	0	0
	0	0	0
	0	0	0
	0	0	0
	0	0	0
	0	0	0
	0	0	0
	0	0	0

	0	0	0
	0	0	0
	0	0	0
	0	0	0
	0	0	0
	0	0	0
	0	0	0
	0	0	0
	0	0	0
	0	0	0
	0	0	0
	0	0	0
	0	0	0
	0	0	0
	0	0	0
	0	0	0
	0	0	0
	0	0	0
	0	0	0
	0	0	0
	0	0	0
	0	0	0
	0	0	0
	0	0	0
	0	0	0
	0	0	0
	0	0	0
	0	0	0
	0	0	0
	0	0	0
	0	0	0
	0	0	0
	0	0	0
	0	0	0
	0	0	0
	0	0	0
	0	0	0
	0	0	0
	0	0	0
	0	0	0
	0	0	0
	0	0	0
	0	0	0
	0	0	0
	0	0	0
	0	0	0
	0	0	0

	0	0	0
	0	0	0
	0	0	0
	0	0	0
	0	0	0
	0	0	0
	0	0	0
	0	0	0
	0	0	0
	0	0	0
	0	0	0
	0	0	0
	0	0	0
	0	0	0
	0	0	0
	0	0	0
	0	0	0
	0	0	0
	0	0	0
	0	0	0
	0	0	0
	0	0	0
	0	0	0
	0	0	0
	0	0	0
	0	0	0
	0	0	0
	0	0	0
	0	0	0
	0	0	0
	0	0	0
	0	0	0
	0	0	0
	0	0	0
	0	0	0
	0	0	0
	0	0	0
	0	0	0
	0	0	0
	0	0	0
	0	0	0
	0	0	0
	0	0	0
	0	0	0
	0	0	0
	0	0	0
	0	0	0

	0	0	0
	0	0	0
	0	0	0
	0	0	0
	0	0	0
	0	0	0
	0	0	0
	0	0	0
	0	0	0
	0	0	0
	0	0	0
	0	0	0
	0	0	0
	0	0	0
	0	0	0
	0	0	0
	0	0	0
	0	0	0
	0	0	0
	0	0	0
	0	0	0
	0	0	0
	0	0	0
	0	0	0
	0	0	0
	0	0	0
	0	0	0
	0	0	0
	0	0	0
	0	0	0
	0	0	0
	0	0	0
	0	0	0
	0	0	0
	0	0	0
	0	0	0
	0	0	0
	0	0	0
	0	0	0
	0	0	0
	0	0	0
	0	0	0
	0	0	0
	0	0	0
	0	0	0
	0	0	0
	0	0	0

	0	0	0
	0	0	0
	0	0	0
	0	0	0
	0	0	0
	0	0	0
	0	0	0
	0	0	0
	0	0	0
	0	0	0
	0	0	0
	0	0	0
	0	0	0
	0	0	0
	0	0	0
	0	0	0
	0	0	0
	0	0	0
	0	0	0
	0	0	0
	0	0	0
	0	0	0
	0	0	0
	0	0	0
	0	0	0
	0	0	0
	0	0	0
	0	0	0
	0	0	0
	0	0	0
	0	0	0
	0	0	0
	0	0	0
	0	0	0
	0	0	0
	0	0	0
	0	0	0
	0	0	0
	0	0	0
	0	0	0
	0	0	0
	0	0	0
	0	0	0
	0	0	0
	0	0	0
	0	0	0
	0	0	0

	0	0	0
	0	0	0
	0	0	0
	0	0	0
	0	0	0
	0	0	0
	0	0	0
	0	0	0
	0	0	0
	0	0	0
	0	0	0
	0	0	0
	0	0	0
	0	0	0
	0	0	0
	0	0	0
	0	0	0
	0	0	0
	0	0	0
	0	0	0
	0	0	0
	0	0	0
	0	0	0
	0	0	0
	0	0	0
	0	0	0
	0	0	0
	0	0	0
	0	0	0
	0	0	0
	0	0	0
	0	0	0
	0	0	0
	0	0	0
	0	0	0
	0	0	0
	0	0	0
	0	0	0
	0	0	0
	0	0	0
	0	0	0
	0	0	0
	0	0	0
	0	0	0
	0	0	0
	0	0	0
	0	0	0

0	0	0
0	0	0
0	0	0
0	0	0
0	0	0
0	0	0
0	0	0
0	0	0
0	-0.003714588	-0.001857294
0	-0.005124746	-0.002562373
0	-0.00623503	-0.003117515
-0.077138901	0.070729676	-0.003204613
0	-0.008292596	-0.004146298
-0.013059405	0	-0.006529703
0.228177518	-0.242488245	-0.007155363
-0.316823183	0.299663819	-0.008579682
0.032836315	-0.0510169	-0.009090292
0	-0.021707951	-0.010853975
0.173455077	-0.19560684	-0.011075882
0	-0.022758278	-0.011379139
0	-0.025374401	-0.012687201
-0.026870007	0	-0.013435003
0	-0.030052387	-0.015026193
0.138961983	-0.170325458	-0.015681738
0	-0.033783991	-0.016891995
-0.036202504	0	-0.018101252
-0.036488118	0	-0.018244059
-0.036488118	0	-0.018244059
0.232629932	-0.270153718	-0.018761893
-0.038494147	0	-0.019247073
-0.038494147	0	-0.019247073
0	-0.042426817	-0.021213408
0.019505004	-0.062365371	-0.021430183
0.158354467	-0.206231786	-0.023938659
-0.093056956	0.042044932	-0.025506012
0	-0.052164935	-0.026082467
0	-0.053796925	-0.026898463
-0.464924459	0.411020712	-0.026951874
0	-0.056733614	-0.028366807
0	-0.056959523	-0.028479761
0	-0.060598126	-0.030299063
0.039471641	-0.10298843	-0.031758395
0	-0.067361639	-0.033680819
0	-0.068380839	-0.034190419
0	-0.069734294	-0.034867147
-0.20513509	0.1284421	-0.038346495
-0.041531527	-0.036138019	-0.038834773

0	-0.077955875	-0.038977938
0	-0.078183527	-0.039091764
-0.10842849	0.02894913	-0.03973968
-0.079655113	0	-0.039827556
-0.084727675	0	-0.042363838
0	-0.087982513	-0.043991257
0	-0.090083014	-0.045041507
-0.083459198	-0.0073648	-0.045411999
0	-0.095743262	-0.047871631
0	-0.096858117	-0.048429059
0.12001395	-0.221943702	-0.050964876
-0.341973356	0.238208338	-0.051882509
0	-0.106853573	-0.053426786
0.478249038	-0.590159619	-0.05595529
0.193079236	-0.309323187	-0.058121975
-0.091618561	-0.033824826	-0.062721693
-0.12570427	0	-0.062852135
0	-0.127311648	-0.063655824
0.035791979	-0.163465178	-0.0638366
0	-0.131251037	-0.065625519
-0.142312523	0	-0.071156261
-0.146011892	0	-0.073005946
-0.146011892	0	-0.073005946
-0.058565862	-0.087667207	-0.073116534
-0.155328053	0.006792889	-0.074267582
0	-0.151949208	-0.075974604
0	-0.15510533	-0.077552665
0.435412119	-0.59213184	-0.078359861
-0.290191819	0.132783373	-0.078704223
0.007455445	-0.165645773	-0.079095164
0	-0.158690132	-0.079345066
-0.176693971	0	-0.088346985
0	-0.178852155	-0.089426077
0	-0.181822273	-0.090911136
0	-0.181822273	-0.090911136
0	-0.184838233	-0.092419117
0	-0.185864436	-0.092932218
0	-0.191314661	-0.09565733
-0.298507241	0.100135035	-0.099186103
0	-0.204160465	-0.102080232
0.092635257	-0.304346797	-0.10585577
-0.215038779	0	-0.10751939
0	-0.217865688	-0.108932844
0	-0.219300381	-0.109650191
-0.219722128	-0.000961	-0.110341645
0	-0.223419056	-0.111709528
-0.228570208	0	-0.114285104

-0.228690632	0	-0.114345316
-0.148803884	-0.081289453	-0.115046668
-0.121563145	-0.112412187	-0.116987666
0	-0.23407789	-0.117038945
-0.238202072	0	-0.119101036
-0.239243173	0	-0.119621587
0	-0.23940711	-0.119703555
-0.016897921	-0.222800618	-0.119849269
0	-0.240822275	-0.120411137
-0.247884785	0	-0.123942393
-0.153275768	-0.097885816	-0.125580792
0	-0.257445664	-0.128722832
-0.268548027	0.007851577	-0.130348225
-0.362053727	0.099755775	-0.131148976
0	-0.263226838	-0.131613419
0	-0.266250032	-0.133125016
0	-0.26778713	-0.133893565
0	-0.26778713	-0.133893565
0.46666696	-0.735087843	-0.134210441
-0.274277265	0	-0.137138632
-0.275707552	0	-0.137853776
0	-0.279904569	-0.139952284
0	-0.282742156	-0.141371078
-0.543720967	0.251635365	-0.146042801
-0.303098529	0	-0.151549265
-0.737318552	0.432995634	-0.152161459
0	-0.322972867	-0.161486433
0	-0.330694716	-0.165347358
-0.285018083	-0.045799176	-0.16540863
0	-0.332237533	-0.166118767
0	-0.334015448	-0.167007724
0	-0.334947265	-0.167473633
0	-0.335802307	-0.167901153
0	-0.335901939	-0.167950969
0.212492523	-0.54968181	-0.168594644
0	-0.343648874	-0.171824437
0	-0.352491141	-0.17624557
-0.353528373	0	-0.176764186
0	-0.354884918	-0.177442459
-0.198079131	-0.159475329	-0.17877723
-0.221738856	-0.136183708	-0.178961282
-0.221738856	-0.136183708	-0.178961282
0	-0.36459946	-0.18229973
0	-0.369487031	-0.184743516
0	-0.369487031	-0.184743516
-0.189037787	-0.184967935	-0.187002861
0	-0.374640412	-0.187320206

0	-0.380101558	-0.190050779
0	-0.383636153	-0.191818076
0	-0.384290362	-0.192145181
0	-0.384917821	-0.192458911
0	-0.392613166	-0.196306583
-0.393319619	0	-0.196659809
0	-0.401979552	-0.200989776
0	-0.407235995	-0.203617998
-0.40730317	0	-0.203651585
0	-0.4116433	-0.20582165
0	-0.411771441	-0.20588572
0.277838363	-0.690368826	-0.206265232
0	-0.414088468	-0.207044234
0	-0.431495964	-0.215747982
0	-0.432687667	-0.216343834
0	-0.438660582	-0.219330291
-0.289903637	-0.149786626	-0.219845132
-0.44445786	0	-0.22222893
-0.445376491	0	-0.222688246
-0.26931525	-0.177477694	-0.223396472
-0.254038912	-0.199033113	-0.226536013
-0.460022267	0	-0.230011133
0	-0.460405595	-0.230202797
-0.465038253	0	-0.232519127
0	-0.465549166	-0.232774583
0	-0.469602399	-0.234801199
0	-0.473619064	-0.236809532
0	-0.473619064	-0.236809532
-0.332739004	-0.146553073	-0.239646039
0	-0.485315383	-0.242657692
0.319079727	-0.805730074	-0.243325174
0	-0.490946349	-0.245473175
-0.311049605	-0.181620616	-0.24633511
0	-0.493277643	-0.246638821
0	-0.494540693	-0.247270346
0.847293932	-1.342745872	-0.24772597
-0.49653114	0	-0.24826557
-0.075426059	-0.425683192	-0.250554625
0	-0.501172943	-0.250586472
-0.507741907	0	-0.253870954
-0.514384141	0	-0.25719207
-0.516037126	0	-0.258018563
-0.011642454	-0.505697228	-0.258669841
-0.517687965	0	-0.258843983
0	-0.524971359	-0.262485679
-0.52514479	0	-0.262572395
-0.19631079	-0.329053795	-0.262682292

-0.402272482	-0.12317022	-0.262721351
-0.525733857	0	-0.262866928
0.089505767	-0.617761242	-0.264127738
0	-0.529792007	-0.264896003
-0.530899425	0	-0.265449713
-0.536253309	0	-0.268126655
0	-0.541016441	-0.270508221
0.054845025	-0.596882871	-0.271018923
0	-0.542078324	-0.271039162
-0.543385992	0	-0.271692996
-0.134945601	-0.409169897	-0.272057749
-0.546482547	0	-0.273241274
-0.550472005	0	-0.275236003
-0.263613001	-0.287899983	-0.275756492
-0.554930848	0	-0.277465424
-0.556034167	0	-0.278017083
0	-0.556371258	-0.278185629
-0.291001165	-0.265928593	-0.278464879
-0.15354754	-0.40359097	-0.278569255
-0.408332329	-0.149403687	-0.278868008
-0.560043825	0	-0.280021913
-0.560622057	0	-0.280311028
-0.565638704	0	-0.282819352
-0.565693911	0	-0.282846955
-0.568385418	0	-0.284192709
-0.352755822	-0.217652604	-0.285204213
-0.139728814	-0.43106939	-0.285399102
-0.573203462	0	-0.286601731
-0.575685675	0	-0.287842837
0	-0.58223427	-0.291117135
1.064306332	-1.647828843	-0.291761256
-0.198365083	-0.385561704	-0.291963394
0	-0.584288198	-0.292144099
-0.585446754	0	-0.292723377
-0.586022327	0	-0.293011164
0	-0.588111248	-0.294055624
0	-0.588300048	-0.294150024
-0.191301058	-0.399255786	-0.295278422
-0.592671926	0	-0.296335963
-0.594438073	0	-0.297219037
-0.594438073	0	-0.297219037
-0.595098828	0	-0.297549414
-0.595149897	0	-0.297574948
-0.595247971	0	-0.297623985
0	-0.59616804	-0.29808402
-0.460666584	-0.136201079	-0.298433832
-0.630105027	0.033029871	-0.298537578

0	-0.59882403	-0.299412015
-0.598855634	0	-0.299427817
-0.599469057	0	-0.299734529
0.122777562	-0.726555956	-0.301889197
-0.604537758	0	-0.302268879
-0.604537758	0	-0.302268879
-0.605395519	0	-0.30269776
-0.606299428	0	-0.303149714
-0.608617656	0	-0.304308828
-0.608987208	0	-0.304493604
-0.610201281	0	-0.30510064
0	-0.610895858	-0.305447929
0	-0.61186767	-0.305933835
-0.61341065	0	-0.306705325
-0.615862799	0	-0.307931399
-0.618116651	0	-0.309058325
0	-0.625928671	-0.312964336
-0.627471683	0	-0.313735841
0	-0.627844457	-0.313922228
-0.457225778	-0.171248868	-0.314237323
0	-0.633543174	-0.316771587
-0.505867048	-0.128730896	-0.317298972
-0.641604223	0	-0.320802112
0.118849644	-0.76422501	-0.322687683
0.985725759	-1.63601944	-0.32514684
-0.650414384	0	-0.325207192
-0.651744342	0	-0.325872171
-0.652390866	0	-0.326195433
-0.658408325	0	-0.329204162
-0.65859769	0	-0.329298845
-0.659951849	0	-0.329975925
-0.660466436	0	-0.330233218
-0.662771907	0	-0.331385954
-0.662953549	0	-0.331476774
0.552805562	-1.216483942	-0.33183919
-0.663709379	0	-0.33185469
-0.66503101	0	-0.332515505
-0.665503233	0	-0.332751617
-0.669227496	0	-0.334613748
-0.672157668	0	-0.336078834
-0.669698931	-0.004157495	-0.336928213
-0.090767891	-0.585196029	-0.33798196
-0.677713252	0	-0.338856626
0	-0.678201018	-0.339100509
0	-0.678201018	-0.339100509
-0.856679408	0.177036778	-0.339821315
-0.680991685	0	-0.340495842

-0.682484357	0	-0.341242178
-0.683066804	0	-0.341533402
-0.686964371	0	-0.343482185
-0.687547137	0	-0.343773568
-0.687631732	0	-0.343815866
0	-0.690307613	-0.345153807
0	-0.694054681	-0.347027341
0	-0.702637561	-0.351318781
-0.794368987	0.084771604	-0.354798692
-0.404771763	-0.305442832	-0.355107297
-0.712390496	0	-0.356195248
0	-0.721725026	-0.360862513
-0.181837954	-0.545567808	-0.363702881
-0.731328402	0	-0.365664201
-0.179233078	-0.554954012	-0.367093545
-0.555210106	-0.185257018	-0.370233562
-0.555307508	-0.185356754	-0.370332131
0	-0.740728784	-0.370364392
0	-0.744066162	-0.372033081
-0.746363108	0	-0.373181554
0	-0.747883786	-0.373941893
-0.279781843	-0.471820219	-0.375801031
-0.540083647	-0.218573506	-0.379328576
-0.760130207	0	-0.380065103
-0.117124756	-0.644503056	-0.380813906
0	-0.762245423	-0.381122712
-0.291262382	-0.47225991	-0.381761146
0	-0.763946142	-0.381973071
-0.12871466	-0.635368944	-0.382041802
-0.765709101	0	-0.382854551
-0.329473308	-0.436758009	-0.383115659
-0.767308484	0	-0.383654242
-0.340661265	-0.426804148	-0.383732707
-0.357877846	-0.410802982	-0.384340414
-0.771994419	0	-0.38599721
-0.36439139	-0.409927266	-0.387159328
0	-0.774322295	-0.387161147
0	-0.784929655	-0.392464827
-0.508707076	-0.276761695	-0.392734386
-0.326624576	-0.465720247	-0.396172412
0	-0.801958379	-0.400979189
0	-0.802358536	-0.401179268
-0.290578611	-0.512371246	-0.401474928
0	-0.806902919	-0.403451459
-0.81107296	0	-0.40553648
0	-0.816146995	-0.408073498
0	-0.821342257	-0.410671129

0	-0.822156661	-0.41107833
-0.822213739	0	-0.411106869
-0.823588071	0	-0.411794036
0	-0.824339107	-0.412169553
-0.495020928	-0.330677928	-0.412849428
0	-0.827169772	-0.413584886
0	-0.828721353	-0.414360677
0	-0.828721353	-0.414360677
-0.533221381	-0.307411176	-0.420316278
-0.843652896	0	-0.421826448
-0.844906453	0	-0.422453226
-0.844906453	0	-0.422453226
0	-0.845337294	-0.422668647
-0.745970978	-0.100629546	-0.423300262
-0.846755631	0	-0.423377816
-0.846904351	0	-0.423452175
-0.946787294	0.09800526	-0.424391017
-0.563630187	-0.287283388	-0.425456788
-0.32748715	-0.524122575	-0.425804862
0	-0.85191155	-0.425955775
-0.855374216	0	-0.427687108
-0.324224329	-0.531503715	-0.427864022
-0.619141572	-0.239600448	-0.42937101
-0.858881368	0	-0.429440684
-0.259973252	-0.600208941	-0.430091096
0	-0.861416895	-0.430708448
-0.272001057	-0.590943603	-0.43147233
-0.767830076	-0.097532241	-0.432681159
0	-0.865452214	-0.432726107
-0.86620161	0	-0.433100805
0	-0.871969688	-0.435984844
-0.874639313	0	-0.437319656
-0.876088348	0	-0.438044174
-0.379171575	-0.499609134	-0.439390354
-0.437035107	-0.443608423	-0.440321765
-0.881300818	0	-0.440650409
-0.417168754	-0.464250956	-0.440709855
-0.887268081	0	-0.443634041
-0.321957842	-0.565782541	-0.443870192
-0.888989235	0	-0.444494617
-0.888989235	0	-0.444494617
-0.890578796	0	-0.445289398
-0.891868594	0	-0.445934297
0	-0.894534861	-0.44726743
-0.89913659	0	-0.449568295
-0.90018689	0	-0.450093445
-0.901117982	0	-0.450558991

-0.902038285	0	-0.451019143
0	-0.902832912	-0.451416456
-0.273460322	-0.63230364	-0.452881981
-0.911755653	0	-0.455877826
-0.911948359	0	-0.45597418
-0.912429641	0	-0.456214821
-0.914818629	0	-0.457409315
-0.914818629	0	-0.457409315
-0.342271879	-0.57267754	-0.457474709
-0.91506788	0	-0.45753394
-0.332001303	-0.58457269	-0.458286997
-0.917518798	0	-0.458759399
-0.918736275	0	-0.459368137
-0.919651669	0	-0.459825835
-0.293958016	-0.628175612	-0.461066814
-0.63616004	-0.290581195	-0.463370618
-0.927196757	0	-0.463598378
-0.869703741	-0.060249221	-0.464976481
0	-0.93026952	-0.46513476
0	-0.931836518	-0.465918259
-0.444387067	-0.488566431	-0.466476749
-0.276758302	-0.65691552	-0.466836911
-0.921446761	-0.015427457	-0.468437109
0	-0.938000137	-0.469000068
-0.939140672	0	-0.469570336
-0.166169431	-0.776642024	-0.471405727
-0.945292965	0	-0.472646483
0	-0.947456407	-0.473728203
-0.954121092	0	-0.477060546
0	-0.960378414	-0.480189207
-0.889931018	-0.077530677	-0.483730848
0	-0.96803954	-0.48401977
0	-0.968351793	-0.484175896
-0.970804244	0	-0.485402122
-0.742627708	-0.231725032	-0.48717637
-0.982198204	0	-0.491099102
0	-0.985922284	-0.492961142
-0.50618689	-0.480371953	-0.493279421
-1.112278847	0.125215835	-0.493531506
0	-0.987829943	-0.493914971
-0.990990681	0	-0.495495341
0	-0.993403951	-0.496701975
-0.479560601	-0.515625592	-0.497593097
0	-0.999709418	-0.499854709
-1.003707226	0	-0.501853613
-0.512983302	-0.49832273	-0.505653016
-1.019171938	0	-0.509585969

0	-1.020828247	-0.510414124
0	-1.020828247	-0.510414124
-1.02093524	0	-0.51046762
-0.819362062	-0.201774642	-0.510568352
0	-1.024793877	-0.512396938
0	-1.028241256	-0.514120628
0	-1.028714897	-0.514357448
0	-1.028795423	-0.514397711
0	-1.043730376	-0.521865188
0	-1.044441501	-0.52222075
0	-1.058321749	-0.529160874
0	-1.058700823	-0.529350412
-0.66332127	-0.395914002	-0.529617636
1.009623273	-2.070547261	-0.530461994
-0.329836792	-0.735106073	-0.532471432
-0.582618248	-0.483607484	-0.533112866
0	-1.06679452	-0.53339726
0	-1.06679452	-0.53339726
-1.068022169	0	-0.534011085
0	-1.068866234	-0.534433117
0	-1.069005017	-0.534502509
0	-1.069005017	-0.534502509
-1.072510703	0	-0.536255352
0	-1.075262896	-0.537631448
0	-1.077240089	-0.538620044
0	-1.078593925	-0.539296962
-0.497127068	-0.583065869	-0.540096468
-1.081061848	0	-0.540530924
0	-1.083949754	-0.541974877
0	-1.085363967	-0.542681983
0	-1.085807201	-0.542903601
0	-1.086041446	-0.543020723
0	-1.086708286	-0.543354143
0	-1.088827107	-0.544413554
0	-1.09115988	-0.54557994
-0.158695901	-0.937793129	-0.548244515
-0.458626424	-0.642434869	-0.550530647
-1.10258045	0	-0.551290225
0	-1.106298836	-0.553149418
0	-1.107262604	-0.553631302
-1.1087805	0	-0.55439025
-0.267159152	-0.842844571	-0.555001862
-1.110744937	0	-0.555372468
0	-1.114966875	-0.557483437
0	-1.129159246	-0.564579623
0	-1.129159246	-0.564579623
0	-1.133486313	-0.566743157

-0.683607937	-0.454457572	-0.569032754
0.103134203	-1.257023209	-0.576944503
-0.426987652	-0.735563935	-0.581275793
0	-1.163718256	-0.581859128
-1.165441439	0	-0.582720719
-0.941099945	-0.225743444	-0.583421695
-0.6012321	-0.566915778	-0.584073939
0	-1.174194115	-0.587097058
0	-1.179413187	-0.589706594
0	-1.184727896	-0.592363948
-0.678999075	-0.5105541	-0.594776587
-0.575548556	-0.639841438	-0.607694997
-0.798012032	-0.420389588	-0.60920081
0	-1.233817362	-0.616908681
-1.243914436	0	-0.621957218
-0.424564701	-0.823065171	-0.623814936
-0.432122038	-0.821959986	-0.627041012
-1.258912951	0	-0.629456476
-0.604458155	-0.655099024	-0.62977859
-0.896789314	-0.371563925	-0.63417662
-1.268641995	0	-0.634320998
0	-1.276982475	-0.638491238
-1.277781568	0	-0.638890784
-0.450069102	-0.830212238	-0.64014067
-1.285138767	0	-0.642569383
0	-1.290207567	-0.645103783
-0.673150216	-0.619151663	-0.64615094
-0.403804681	-0.89165353	-0.647729106
-1.296128193	0	-0.648064096
-0.463540437	-0.839051443	-0.65129594
0	-1.327498981	-0.663749491
-0.304198068	-1.030376836	-0.667287452
-0.801340241	-0.540046103	-0.670693172
-0.855343892	-0.48955492	-0.672449406
-1.082548954	-0.265173668	-0.673861311
0	-1.360152924	-0.680076462
-1.360676955	0	-0.680338478
-1.363011393	0	-0.681505697
-1.366532956	0	-0.683266478
-0.754638195	-0.615436044	-0.685037119
-1.372920746	0	-0.686460373
-0.619715549	-0.753954392	-0.686834971
0	-1.382489265	-0.691244633
-1.382755394	0	-0.691377697
-0.851692388	-0.536511458	-0.694101923
0	-1.396162489	-0.698081244
-1.401958954	0	-0.700979477

-0.760142337	-0.648538462	-0.704340399
-0.426343401	-1.001129291	-0.713736346
-0.832931405	-0.598117747	-0.715524576
0	-1.432497535	-0.716248768
-1.442996441	0	-0.721498221
-0.873612192	-0.57137634	-0.722494266
-0.943958323	-0.50482367	-0.724390996
-1.458725738	0	-0.729362869
-0.996059521	-0.464635742	-0.730347632
-0.799683095	-0.665775413	-0.732729254
0	-1.466052883	-0.733026442
-0.884547553	-0.587678035	-0.736112794
0	-1.479207551	-0.739603775
-0.74081772	-0.7494541	-0.74513591
0	-1.494436773	-0.747218386
0	-1.494436773	-0.747218386
-0.811170985	-0.686275426	-0.748723205
0	-1.499653367	-0.749826684
0	-1.519906968	-0.759953484
-1.520627025	0	-0.760313513
0	-1.521378246	-0.760689123
0	-1.529954169	-0.764977085
0	-1.532741433	-0.766370717
-0.750786913	-0.783246782	-0.767016847
0	-1.545260489	-0.772630245
0	-1.561349708	-0.780674854
0	-1.56462621	-0.782313105
-0.730207263	-0.850081067	-0.790144165
-0.878833394	-0.708452992	-0.793643193
0	-1.595631286	-0.797815643
0.549967491	-2.150237086	-0.800134798
-1.605239936	0	-0.802619968
-0.253327388	-1.354528347	-0.803927867
-0.962468306	-0.651643631	-0.807055969
0	-1.620912109	-0.810456055
0	-1.621602148	-0.810801074
-1.622978377	0	-0.811489188
-1.622978377	0	-0.811489188
-0.784218624	-0.841046871	-0.812632747
-1.625359736	0	-0.812679868
-0.628463202	-0.999960694	-0.814211948
-0.779925743	-0.853726402	-0.816826073
0	-1.639163964	-0.819581982
0.54380897	-2.186251601	-0.821221316
-0.244984337	-1.407547246	-0.826265792
-0.894588631	-0.75850422	-0.826546426
0	-1.657157971	-0.828578986

-1.657949349	0	-0.828974674
0.554182722	-2.217596988	-0.831707133
0	-1.663643676	-0.831821838
0	-1.664059213	-0.832029606
0	-1.66640015	-0.833200075
-0.736269696	-0.93263189	-0.834450793
0.552461907	-2.232023524	-0.839780808
0.554168738	-2.235451602	-0.840641432
-0.77064519	-0.911361089	-0.84100314
0.551482973	-2.235825728	-0.842171378
0.546454464	-2.234490417	-0.844017977
0	-1.693340677	-0.846670339
-0.635029079	-1.06318689	-0.849107984
-1.71247214	0	-0.85623607
-1.180057434	-0.534033067	-0.85704525
-0.27033483	-1.4488751	-0.859604965
0.545356118	-2.266287294	-0.860465588
0.453134341	-2.188756229	-0.867810944
0.187322247	-1.924967664	-0.868822709
-0.982364121	-0.756055107	-0.869209614
-0.982364121	-0.756055107	-0.869209614
0	-1.740007955	-0.870003977
0	-1.740036854	-0.870018427
0	-1.741432415	-0.870716207
0.547554084	-2.289092753	-0.870769335
0.547554084	-2.289092753	-0.870769335
0.437167431	-2.178826802	-0.870829686
0	-1.749303944	-0.874651972
0.568512179	-2.324086181	-0.877787001
-0.79155039	-0.970829633	-0.881190012
0	-1.76258972	-0.88129486
0.417915155	-2.181573267	-0.881829056
0.417915155	-2.181573267	-0.881829056
0.417221099	-2.182633812	-0.882706357
-1.348071368	-0.418213027	-0.883142197
0.564258024	-2.331302249	-0.883522112
0.421750314	-2.189143213	-0.88369645
-0.817837552	-0.951667992	-0.884752772
0	-1.773271686	-0.886635843
0	-1.773271686	-0.886635843
-0.849003281	-0.925525962	-0.887264621
0.419923916	-2.194638354	-0.887357219
0.408013854	-2.183292979	-0.887639563
0	-1.776050215	-0.888025107
0.405309368	-2.182451302	-0.888570967
-0.720926826	-1.056219838	-0.888573332
-0.49435845	-1.283653548	-0.889005999

0.410674149	-2.1895225	-0.889424176
0	-1.780751389	-0.890375695
0.536735619	-2.330233296	-0.896748838
0.348501705	-2.145266028	-0.898382162
0.348501705	-2.145266028	-0.898382162
0	-1.796977378	-0.898488689
0.416934954	-2.216794886	-0.899929966
-1.080794245	-0.728680141	-0.904737193
-1.312531564	-0.499103017	-0.905817291
-1.312531564	-0.499103017	-0.905817291
0.41637185	-2.230497513	-0.907062831
0.577358551	-2.393435795	-0.908038622
-1.635655347	-0.181061918	-0.908358632
0	-1.81702788	-0.90851394
-1.042256068	-0.775386388	-0.908821228
0.43260106	-2.253514585	-0.910456763
0	-1.823231733	-0.911615866
0.428683765	-2.259000307	-0.915158271
-0.890543034	-0.940095344	-0.915319189
0	-1.832257293	-0.916128646
-0.70490397	-1.130731756	-0.917817863
0	-1.837835306	-0.918917653
0	-1.83881283	-0.919406415
0	-1.843490154	-0.921745077
0	-1.8446211	-0.92231055
-1.165747565	-0.680019983	-0.922883774
-0.910522983	-0.938304069	-0.924413526
0.39612875	-2.246527393	-0.925199322
-0.907194157	-0.947210808	-0.927202483
-1.017158437	-0.838136659	-0.927647548
-0.580543023	-1.276568199	-0.928555611
-0.999754138	-0.8574061	-0.928580119
0.396806142	-2.254503613	-0.928848735
0	-1.865179058	-0.932589529
-0.288657784	-1.579986269	-0.934322026
0	-1.875350783	-0.937675391
0.414891471	-2.29259306	-0.938850794
0.40858275	-2.287319963	-0.939368607
0.429311834	-2.316337093	-0.94351263
0	-1.889776592	-0.944888296
-0.302484853	-1.591976532	-0.947230692
-0.302484853	-1.591976532	-0.947230692
0	-1.895187185	-0.947593592
0.346324188	-2.242929193	-0.948302502
0.400601317	-2.309253611	-0.954326147
0	-1.913279736	-0.956639868
-0.923807044	-0.99747685	-0.960641947

-0.86094177	-1.069711092	-0.965326431
-0.329584889	-1.601339522	-0.965462205
-1.112490667	-0.821863208	-0.967176938
0.386630484	-2.321452998	-0.967411257
0	-1.939626911	-0.969813456
0	-1.949066399	-0.974533199
-1.10018404	-0.851805367	-0.975994703
0	-1.964987696	-0.982493848
0	-1.969232925	-0.984616463
-0.269001057	-1.709469068	-0.989235062
-0.95885824	-1.024165278	-0.991511759
0	-1.990977512	-0.995488756
0	-2.001062585	-1.000531292
0	-2.001083305	-1.000541652
0	-2.008509394	-1.004254697
0	-2.010696523	-1.005348262
0.399756146	-2.414671404	-1.007457629
-1.045221653	-0.970583131	-1.007902392
0	-2.016936334	-1.008468167
0	-2.017322309	-1.008661154
0	-2.017322309	-1.008661154
-1.256662354	-0.762487867	-1.009575111
-1.256662354	-0.762487867	-1.009575111
0	-2.022316596	-1.011158298
-1.153583268	-0.869060075	-1.011321671
0	-2.023302681	-1.011651341
0	-2.023302681	-1.011651341
-0.996521509	-1.027515286	-1.012018397
0	-2.027142921	-1.01357146
-0.952755889	-1.07541418	-1.014085035
0	-2.032462035	-1.016231018
-0.823702157	-1.20943127	-1.016566713
-2.03401789	0	-1.017008945
0	-2.034386721	-1.017193361
-1.173895137	-0.865185514	-1.019540325
-0.320825824	-1.718402621	-1.019614223
0	-2.044012847	-1.022006423
0	-2.050239701	-1.02511985
0	-2.050536043	-1.025268022
-0.969606665	-1.084322154	-1.026964409
0	-2.059302873	-1.029651436
-0.99747626	-1.063646857	-1.030561559
-1.000924242	-1.061798851	-1.031361546
-1.001268482	-1.061818775	-1.031543628
-1.109396106	-0.955079564	-1.032237835
-1.02072189	-1.044995939	-1.032858915
0	-2.06711942	-1.03355971

0	-2.06711942	-1.03355971
-1.059632406	-1.011786714	-1.03570956
-1.047554634	-1.031669677	-1.039612156
-0.629148833	-1.45341475	-1.041281791
-0.997527841	-1.094792869	-1.046160355
-0.997527841	-1.094792869	-1.046160355
0	-2.098249736	-1.049124868
0	-2.101999839	-1.05099992
-0.391344717	-1.712797302	-1.052071009
0	-2.108820393	-1.054410196
0	-2.109374892	-1.054687446
0	-2.109387534	-1.054693767
-0.365901431	-1.752562964	-1.059232198
-0.365901431	-1.752562964	-1.059232198
-1.03843513	-1.080589714	-1.059512422
-0.360348154	-1.761687713	-1.061017934
0	-2.125052085	-1.062526043
-0.319587744	-1.811810599	-1.065699172
-0.051822345	-2.081677004	-1.066749675
0	-2.133836853	-1.066918426
0	-2.133836853	-1.066918426
-1.006261197	-1.128469484	-1.067365341
-1.134910782	-1.000256918	-1.06758385
-2.139663202	0	-1.069831601
-0.354853258	-1.785893397	-1.070373327
-0.263784254	-1.877430531	-1.070607393
-1.05067705	-1.098920789	-1.074798919
-0.351591138	-1.806714772	-1.079152955
-1.03763016	-1.125761822	-1.081695991
-1.021065248	-1.150168179	-1.085616713
-0.885303458	-1.285976203	-1.085639831
-0.885303458	-1.285976203	-1.085639831
-0.288548727	-1.886128034	-1.087338381
-0.911185211	-1.265052452	-1.088118832
-2.180577346	0	-1.090288673
-2.182853306	0	-1.091426653
0	-2.193806711	-1.096903355
-0.993888832	-1.200210144	-1.097049488
-0.960462758	-1.238269037	-1.099365897
-1.076027273	-1.136076562	-1.106051917
-0.366612345	-1.851000591	-1.108806468
-0.355733688	-1.875263738	-1.115498713
-0.962150975	-1.26902649	-1.115588732
-0.364355149	-1.868124904	-1.116240026
-2.235315533	0	-1.117657766
0	-2.236997379	-1.11849869
-0.390744371	-1.860363379	-1.125553875

-0.348375367	-1.904115893	-1.12624563
0	-2.259676125	-1.129838063
-1.049622597	-1.216076707	-1.132849652
-2.267329524	0	-1.133664762
-0.951195034	-1.326969402	-1.139082218
0	-2.295196064	-1.147598032
-0.946446232	-1.357774678	-1.152110455
-1.06370754	-1.24212838	-1.15291796
0	-2.31051082	-1.15525541
-1.40809495	-0.919268765	-1.163681858
-1.074560175	-1.256337816	-1.165448996
-2.335918454	0	-1.167959227
-1.258453116	-1.077943457	-1.168198286
-0.425825472	-1.917650458	-1.171737965
-1.665580447	-0.679455285	-1.172517866
-1.196299718	-1.164702635	-1.180501176
-1.200897063	-1.163504741	-1.182200902
-1.108968628	-1.260381384	-1.184675006
-1.16313912	-1.216702306	-1.189920713
-1.206611608	-1.188390235	-1.197500922
-1.195927111	-1.220885889	-1.2084065
-1.434499072	-0.995098681	-1.214798876
0	-2.431598273	-1.215799136
-1.200807249	-1.231660128	-1.216233688
0	-2.433015589	-1.216507795
-1.426938532	-1.016382067	-1.2216603
-1.248997936	-1.206627894	-1.227812915
-1.103028718	-1.357157935	-1.230093326
-1.037924822	-1.422494764	-1.230209793
-1.305185742	-1.166570807	-1.235878274
-2.476452143	0	-1.238226071
-1.396569708	-1.086694935	-1.241632321
-1.285588205	-1.219248005	-1.252418105
-1.295264901	-1.228533164	-1.261899033
0	-2.535512278	-1.267756139
-2.015278412	-0.526148801	-1.270713606
-1.174704045	-1.369238935	-1.27197149
-1.460995438	-1.103048741	-1.28202209
-1.158431499	-1.406663052	-1.282547276
-1.324666831	-1.256479364	-1.290573097
-1.011909721	-1.573630165	-1.292769943
-1.477391204	-1.119413987	-1.298402595
-1.48077409	-1.126543319	-1.303658705
0	-2.62020703	-1.310103515
0	-2.667761401	-1.333880701
0	-2.668669953	-1.334334977
-1.238810579	-1.441345727	-1.340078153

-1.215534211	-1.46926385	-1.342399031
0	-2.688889773	-1.344444887
0	-2.697058434	-1.348529217
0	-2.705539179	-1.352769589
0	-2.714900628	-1.357450314
-1.21423285	-1.504095293	-1.359164072
0	-2.719865735	-1.359932868
0	-2.723069503	-1.361534751
-1.268623439	-1.454543447	-1.361583443
0	-2.729606004	-1.364803002
-1.213214819	-1.521394989	-1.367304904
-1.57641939	-1.159117868	-1.367768629
0	-2.738577594	-1.369288797
0	-2.740561208	-1.370280604
0	-2.744871772	-1.372435886
0	-2.744926196	-1.372463098
0	-2.757637301	-1.378818651
0	-2.762468431	-1.381234216
-0.413843369	-2.349014794	-1.381429081
0	-2.767352773	-1.383676387
0	-2.772045984	-1.386022992
0	-2.784477586	-1.392238793
0	-2.785348661	-1.39267433
-1.395965651	-1.39285618	-1.394410916
0	-2.79545261	-1.397726305
0	-2.795917649	-1.397958825
0	-2.796193724	-1.398096862
0	-2.797259799	-1.3986299
-1.816215822	-0.989426197	-1.40282101
0	-2.820925647	-1.410462824
0	-2.823014051	-1.411507025
0	-2.824998937	-1.412499469
0	-2.824998937	-1.412499469
0	-2.827062521	-1.413531261
0	-2.844250008	-1.422125004
0	-2.852612448	-1.426306224
0	-2.854156031	-1.427078015
0	-2.854156031	-1.427078015
0	-2.857369201	-1.428684601
-1.377305142	-1.482376794	-1.429840968
0	-2.863540479	-1.431770239
0	-2.863973515	-1.431986758
0	-2.865124519	-1.432562259
0	-2.871445367	-1.435722683
0	-2.871445367	-1.435722683
0	-2.877553256	-1.438776628
0	-2.878889062	-1.439444531

0	-2.882261723	-1.441130862
0	-2.884577167	-1.442288583
-1.385817654	-1.506032445	-1.44592505
0	-2.893543016	-1.446771508
-1.52525611	-1.369721079	-1.447488595
0	-2.897614915	-1.448807458
0	-2.903733207	-1.451866604
-1.475327916	-1.430491319	-1.452909618
0	-2.907963979	-1.453981989
0	-2.907963979	-1.453981989
-1.205880451	-1.703307163	-1.454593807
-1.464061114	-1.44550375	-1.454782432
-1.464061114	-1.44550375	-1.454782432
0	-2.91052692	-1.45526346
0	-2.91052692	-1.45526346
0	-2.912068033	-1.456034017
0	-2.914376635	-1.457188318
0	-2.922018224	-1.461009112
0	-2.922212746	-1.461106373
0	-2.938382462	-1.469191231
0	-2.938990458	-1.469495229
0	-2.940671942	-1.470335971
0	-2.943055733	-1.471527867
0	-2.945456531	-1.472728266
0	-2.95320048	-1.47660024
-1.363083881	-1.590857698	-1.476970789
0	-2.959029273	-1.479514636
0	-2.962907043	-1.481453522
-1.444702304	-1.527058841	-1.485880573
0	-2.972970257	-1.486485128
0	-2.976134561	-1.48806728
-1.422981778	-1.574020445	-1.498501111
-1.497241342	-1.500134895	-1.498688119
0	-3.012438051	-1.506219025
0	-3.012438051	-1.506219025
-1.478158819	-1.534662427	-1.506410623
0	-3.015640226	-1.507820113
-1.421144252	-1.595882227	-1.508513239
0	-3.024173796	-1.512086898
-1.705821125	-1.325645758	-1.515733441
-1.574795824	-1.456709898	-1.515752861
0	-3.03270791	-1.516353955
-1.888134897	-1.145894894	-1.517014895
-1.472643618	-1.567713518	-1.520178568
0	-3.046572347	-1.523286174
0	-3.046572347	-1.523286174
-1.934475807	-1.113437787	-1.523956797

0	-3.060187445	-1.530093723
-1.511503555	-1.588901284	-1.550202419
-1.651662832	-1.458585609	-1.555124221
0	-3.111625068	-1.555812534
-1.4891768	-1.626039551	-1.557608176
0	-3.116904556	-1.558452278
0	-3.116904556	-1.558452278
-1.551654749	-1.565852083	-1.558753416
-1.576091166	-1.570079995	-1.57308558
-1.554238777	-1.602113993	-1.578176385
-1.381720001	-1.785209646	-1.583464824
0	-3.175217784	-1.587608892
-2.023763648	-1.153720239	-1.588741944
0	-3.181027722	-1.590513861
-1.597810388	-1.594848237	-1.596329313
-1.606425797	-1.591860226	-1.599143011
-1.606425797	-1.591860226	-1.599143011
0	-3.222075436	-1.611037718
-1.933974933	-1.31420391	-1.624089422
0	-3.266813693	-1.633406847
-0.916617011	-2.36760496	-1.642110986
-1.786636641	-1.501907551	-1.644272096
0	-3.321891787	-1.660945894
0	-3.375259029	-1.687629515
0	-3.486753501	-1.743376751
-2.01006776	-1.525446271	-1.767757016
-1.804773434	-1.799839017	-1.802306225
-1.976534956	-1.674877041	-1.825705999
-2.479537272	-1.207183644	-1.843360458
-0.740405573	-2.972108495	-1.856257034
-0.769603471	-2.944069844	-1.856836657
-0.786664754	-2.941520107	-1.864092431
-0.776786477	-2.954072821	-1.865429649
-0.785048765	-2.947492831	-1.866270798
-2.099069228	-1.635924045	-1.867496636
-0.755356402	-2.999934693	-1.877645547
-0.787498643	-2.968496056	-1.877997349
-2.132167166	-1.638948962	-1.885558064
-1.656054271	-2.146257523	-1.901155897
-2.201416637	-1.638960377	-1.920188507
-0.71823746	-3.160074768	-1.939156114
-0.759894082	-3.124766802	-1.942330442
-0.746034184	-3.140914863	-1.943474524
-0.746034184	-3.140914863	-1.943474524
-2.297134678	-1.649156828	-1.973145753
-2.442623568	-1.506412397	-1.974517982
-2.293261972	-1.760836336	-2.027049154

	-1.211262629	-2.960357833	-2.085810231
	-2.593743353	-1.735690942	-2.164717148
	-2.566734322	-2.283206043	-2.424970183
